# Guidelines for laparoscopic treatment of ventral and incisional abdominal wall hernias (International Endohernia Society (IEHS)—Part 1

**DOI:** 10.1007/s00464-013-3170-6

**Published:** 2013-10-11

**Authors:** R. Bittner, J. Bingener-Casey, U. Dietz, M. Fabian, G. S. Ferzli, R. H. Fortelny, F. Köckerling, J. Kukleta, K. LeBlanc, D. Lomanto, M. C. Misra, V. K. Bansal, S. Morales-Conde, B. Ramshaw, W. Reinpold, S. Rim, M. Rohr, R. Schrittwieser, Th. Simon, M. Smietanski, B. Stechemesser, M. Timoney, P. Chowbey

**Affiliations:** 1 Hernia Center Rottenburg am Neckar, Winghofer Medicum, Röntgenstr. 38, 72108 Rottenburg, Germany; 2 Division of Gastroenterological and General Surgery, Mayo Clinic, 200 First Street SW, Rochester, MN 55905 USA; 3 Department of General, Visceral, Vascular, and Pediatric Surgery (Department of Surgery I), University Hospital of Wuerzburg, Oberduerrbacher Strasse 6, 97080 Wurzburg, Germany; 4 Department of General Surgery, Halifax Health, Daytona Beach, FL USA; 5 Department of Surgery, Lutheran Medical Center, SUNY Health Science Center, 65 Cromwell Avenue, Brooklyn, Staten Island, NY USA; 6 Department of General, Visceral and Oncological Surgery, Wilhelminenspital, 1171 Vienna, Austria; 7 Department of Surgery and Center for Minimally Invasive Surgery, Vivantes Hospital, Neue Bergstrasse 6, 13585 Berlin, Germany; 8 General, Visceral, Abdominal Wall Surgery, Klinik Im Park, Grossmuensterplatz 9, 8001 Zurich, Switzerland; 9 Minimally Invasive Surgery Institute and the Fellowship Program, Surgeons Group of Baton Rouge of Our Lady of the Lake Physician Group, Baton Rouge, LA USA; 10 Minimally Invasive Surgical Center, KTP Advanced Surgical Training Center, YYL School of Medicine, National University Hospital, Kent Ridge Wing 2, 5 Lower Kent Ridge Road, Singapore, 119074 Singapore; 11 Division of Minimally Invasive Surgery, J P N Apex Trauma Centre, All India Institute of Medical Sciences, Angari Nagar, New Delhi, 110029 India; 12 Unit of Innovation in Minimally Invasive Surgery, University Hospital, Virgen del Rocío, Seville, Spain; 13 Department of Surgery, Gross-Sand Hospital Hamburg, Gross-Sand 3, 21107 Hamburg, Germany; 14 Department of General Surgery, Katutura State Hospital, P.O. Box 81233, Olympia, Windhoek Namibia; 15 Department of Surgery, LKH, Tragösserstrasse 1 und 1a, Muerzzuschlag, 8600 Bruck/Mur, Austria; 16 Department of Surgery, GRN‐Klinik Sinsheim, Weinheim, Germany; 17 Ceynowa Hospital, Wejherowo, Poland; 18 Hernienzentrum Köln, Zeppelinstrasse 1, 50667 Cologne, Germany; 19 Minimal Access, Metabolic and Bariatric Surgery, Max Healthcare Institute Ltd., 2 Press Enclave Road, Saket, New Delhi, India

**Keywords:** Evidence-based medicine, Guidelines, Laparoscopic ventral hernia repair, Indication for surgery, Perioperative management

## Abstract

Guidelines are increasingly determining the decision process in day-to-day clinical work. Guidelines describe the current best possible standard in diagnostics and therapy. They should be developed by an international panel of experts, whereby alongside individual experience, above all, the results of comparative studies are decisive. According to the results of high-ranking scientific studies published in peer-reviewed journals, statements and recommendations are formulated, and these are graded strictly according to the criteria of evidence-based medicine. Guidelines can therefore be valuable in helping particularly the young surgeon in his or her day-to-day work to find the best decision for the patient when confronted with a wide and confusing range of options. However, even experienced surgeons benefit because by virtue of a heavy workload and commitment, they often find it difficult to keep up with the ever-increasing published literature. All guidelines require regular updating, usually every 3 years, in line with progress in the field. The current Guidelines focus on technique and perioperative management of laparoscopic ventral hernia repair and constitute the first comprehensive guidelines on this topic. In this issue of *Surgical Endoscopy,* the first part of the Guidelines is published including sections on basics, indication for surgery, perioperative management, and key points of technique. The next part (Part 2) of the Guidelines will address complications and comparisons between open and laparoscopic techniques. Part 3 will cover mesh technology, hernia prophylaxis, technique-related issues, new technologic developments, lumbar and other unusual hernias, and training/education.

**Electronic supplementary material:**

The online version of this article (doi:10.1007/s00464-013-3170-6) contains supplementary material, which is available to authorized users.

Guidelines describe the current, best possible standard in diagnostics and therapy. Critics against the implementation of guidelines in clinical practice argue that they restrict the physician’s freedom to manage patients in accordance with personal experience and may restrict medical therapeutic freedom.

 However, failure to follow guidelines may disadvantage patient outcome, and for this reason, the benefit and importance of guidelines need to be explained to all practitioners. In this context, guidelines have to be evidence based and should be formulated by an international panel of experts who are able to grade the recommendations (level of evidence) according to established criteria of evidence-based medicine (EBM).

Incisional and ventral abdominal wall hernias are common. Their operative repair forms a part of the daily routine practiced by every general and visceral surgeon. In Germany alone, 50,000 of these operations are performed each year. Although operations for abdominal wall hernia are comparatively unspectacular, they still can be invasive in a major way to the individual patient, incurring a long and painful period of illness and even leading in some cases to a lethal outcome.

The operation for an abdominal wall hernia is plastic reconstructive in nature. Findings and operation procedures can be extremely complex, for example, with respect to the size of the defect or hernia sac, the extent of intraabdominal adhesions, the required operative competence, the length of the operation, and the costs for the materials.

A surgeon who has not been trained in this specific area finds it increasingly difficult to determine the best treatment option. Guidelines can solve this problem. The fundamental precondition for reliable guidelines is the availability of quality published studies ranking high in the classification of the EBM.

At the beginning of the guidelines process, critics expressed fears that evidence from studies was not yet sufficient to answer many important questions. This argument deserves to be taken seriously, but a PubMed search on “ventral hernias” produced 8,000 papers, and a search on “incisional hernias” resulted in 2,700 publications. The preconditions for the development of reliable guidelines are

1. An international panel of experts qualified by their publications in peer-review journals
2. Two experts from the assembled group to address one specific topic
3. Complete transparency of the process used in formulating the guidelines and clear communication between the assembled group of experts
4. A final consensus conference to confirm the final version of the guidelines.

The development process for the current guidelines was conducted in a form similar to the development of the “Guidelines for laparoscopic (TAPP) and endoscopic (TEP) treatment of inguinal hernia (International Endohernia Society [IEHS])” (Surg Endosc 2011;25:2773–2843).

The process was started in January 2011 by collection of relevant published data and recruiting of qualified experts in laparoscopic ventral hernia repair. Approximately 40 experts from three continents were invited to participate in a consensus conference. The conference was set up within the framework of the 5th Meeting of the International Endohernia Society (IEHS) organized for October 2011 in Suzhou, China by Professor Z. L. Ji (Nanjing), Professor Q. Y. Yao (Shanghai), and Professor H. R. Wu (Suzhou). The assembled experts were asked several key questions about their willingness to participate, the most important issues related to laparoscopic surgery, what topics the individual experts would address, and the like. On the basis of the answers received, 37 topics were identified, and 22 surgeons declared their willingness to formulate drafts for the respective Guidelines. This constituted the first stage of the guidelines development process.

In the second stage of the process, the experts were asked to (1) search the literature available on the topic and (2) to grade the papers according to the Oxford hierarchy of evidence (following the advice of Dr. S. Sauerland) as outlined later, consisting of the following five levels:

**Table:** 

1A. Systematic review of randomized clinical trials (RCTs) (with consistent results from individual studies)
1B. RCTs (of good quality)
2A. Systematic review of 2B studies (with consistent results from individual studies)
2B. Prospective and comparative studies (or RCTs of poorer quality)
2C. Outcome studies (e.g., analyses of large registries, population-based data)
3. Retrospective and comparative studies, case–control studies
4. Case series (i.e., studies without a control group)
5. Expert opinion, animal or lab experiments.

For the recommendations, the following grading scale was used:

**Table:** 

A Consistent level 1 studies: strict recommendations (“standard,” “surgeons *must* do it”)
B Consistent level 2 or 3 studies or extrapolations from level 1 studies: less strict wording (“recommended,” “surgeons *should* do it”)
C Level 4 studies or extrapolations from level 2 or 3 studies: vague wording (“option,” “surgeons *can* do it”)
D Level 5 evidence or worryingly inconsistent or inconclusive studies at any level (no recommendation at all, described options).

In the third stage of the process, the experts were requested to prepare a document for presentation at the Consensus Conference in Suzhou at the 5th Meeting of the International Endohernia Society (IEHS), 13–16 October 2011. All the papers were discussed first among the experts and then 1 day later during the plenary session attended by several hundred participants.

In the fourth stage of the process, during the following months, the authors drafted the first version of their specific sections including all the suggestions they had received during the Conference. These first versions were distributed to all the other experts for criticisms, comments, and supplements, leading to the formulation of the agreed-upon Guidelines.

## Basics

### How comparable are incisional and ventral hernias in terms of operative technique and outcomes?

#### Bruce Ramshaw

The following search terms were used: “variability of incisional hernia” (3/5), “variability of ventral hernia” (2/8), “laparoscopic ventral hernia variability” (0/0), “laparoscopic incisional hernia repair variability” (0/1), “complexity of ventral hernia repair” (2/14), “complexity of laparoscopic ventral hernia repair” (2/8), “complexity of incisional hernia repair” (0/7), and “complexity of laparoscopic incisional hernia repair” (0/5).

The search, performed in October 2011, resulted in four publications, all of which were clinical studies. A secondary search resulted in an additional 22 publications pertinent to this topic, 10 of which were clinical. The remainder were nonclinical studies.

##### Statements

**Table:** 

Level 4	The level of complexity and variability for ventral/incisional hernia patients and repair techniques is high.
Level 5	The degree of complexity is growing higher at an increasing rate of change. The techniques and outcomes, therefore, cannot be considered comparable using current methods of analysis due to the many complex ever-changing variables as well as the relationships between variables, which are not controllable.

##### Recommendations

**Table:** 

Grade C	Due to the increasing pace of change and the complexity of ventral/incisional hernia patients and techniques, use of traditional human subjects clinical research, evidence-based methods and guidelines in health care should be considered a starting point rather than a goal.
Grade C	The application of principles of complex adaptive systems science, particularly real-world clinical quality-improvement methods, likely will be required to improve the value of care (e.g., quality outcomes measures, satisfaction, patient experience, costs) for the patient with a ventral/incisional hernia.

Abdominal wall hernia disease clearly is more complex than previously thought. In addition, the patient groups presenting with incisional and ventral hernias are becoming more complex as the treatment options, including the varieties of mesh, continue to grow. This increasing complexity as well as the variability of outcomes leads us to challenge the traditional application of EBM, which to date has not included knowledge generated from clinical quality-improvement studies. This is not to say that this understanding of EBM does not have value for complex problems, such as abdominal wall hernia disease. However, it is incomplete and represents a starting point rather than a goal toward understanding how to improve the value of care for both the patient who presents with a ventral/incisional hernia and the system in which that care is provided.

This chapter describes the current evidence for the variability of ventral/incisional hernia patients and presents a brief framework for understanding how to apply new thinking to the study of complex problems such as ventral/incisional hernia disease.

During the past 150 years, traditional clinical research methods have been based on reductionist scientific approaches, in which the scientific method is applied to the study of one part or variable (e.g., a drug or device) within a complex system (e.g., a patient’s cycle of care). This approach to medical research has led to significant improvements in health care. Without the ability to perform prospective, RCTs, many improvements in health care would not have been achieved. However, a closer look at advances in health care shows that many significant innovations did not come from well-planned studies based on the traditional application of the scientific method. They often were discovered by accident or by innovators outside the traditional scientific community [1, 2].

Many treatments approved through rigorous scientific scrutiny have later been proven to cause unexpected and unintended harm or have been found to offer unexpected benefits for other unrelated diseases [3, 4]. Even major medical initiatives, such as the human genome project, have emerged through loose collaborations and relationships between various individuals and often between various types of experts [5]. More recently, many health care research initiatives are being initiated by patients and family members who have been frustrated by the lack of medical knowledge generated by our traditional research mechanisms (e.g., the women who started studies on spontaneous coronary artery dissection because none were available, and the two mothers from Old Lyme, CT, who initiated the studies elucidating the cause of Lyme disease) [6, 7].

A new field of medicine is forming, referred to as complex adaptive systems research [8]. Complex adaptive systems describe any biologic organism (e.g., the human body) and any grouping of biologic organisms (e.g., our health care system). Research conducted to generate evidence based on the study of complex adaptive systems includes clinical quality improvement methods, participatory research (sometimes led by patients and family members), and documentation of data throughout the entire cycle of patient care including psychosocial and other nontraditional outcomes measures. This field recognizes that humans likely belong to many subgroups that must be identified for better prediction of outcomes and improvement of value. These subgroups may be based on genetics, environment, disease states, age, sex, and the like.

Many researchers are realizing that the traditional application of reductionist research methods often is inadequate in the search to improve the value of patient care [9]. One reason these traditional research methods are inadequate is that as our medical knowledge increases exponentially, an almost infinite number variables appear, with an almost infinite number of complex relationships between them. These relational interactions can have an impact on the outputs, leading to an escalating degree of complexity in health care and our world in general [10]. In addition, these variables and relationships are constantly changing and are not controllable. In light of this increasing complexity, traditional research methods alone are not sufficient to improve the value of care for the patient or the value of the overall health care system [11].

##### Research

This knowledge of complex adaptive systems and increasing complexity has an impact on our understanding of the variability we see for the patient with a ventral/incisional hernia. Variability that can have an impact on outcomes for ventral/incisional hernia repair may include patient factors, technique variability, surgeon skill, and variability in mesh characteristics, as well as variability in both the environmental conditions of the patient’s home living conditions and the facility in which treatment occurs.

Studies on the variability of ventral/incisional hernias are few, but a comparison of studies analyzing different types of ventral/incisional hernias clearly shows a large variety of outcomes based on many complex factors. One study within the U.S. Veterans Affairs (VA) system showed significant variation in the use of mesh for ventral/incisional hernia repair, which correlated with less recurrence at the facilities in which mesh was used more often (up to a fourfold increase in mesh use) [12]. A study using similar VA data showed that the location of mesh placement also had an impact on outcomes, with laparoscopic and underlay mesh placement leading to lower recurrence rates than onlay and inlay mesh placement [13].

One prospective clinical study attempted to define some of the complex variables involved in laparoscopic ventral/incisional hernia repair. In that study, Jenkins et al. [14] documented significant variation for a number of variables from a group of 180 patients, with data collected prospectively. Significant variation was documented for patient age, body mass index (BMI), number of previous open abdominal procedures (range 0–13), previous laparoscopic procedures (range 0–6), number of prior hernia repairs (range 0–8), and many other patient factors. Significant variation also was documented for the actual operative procedure, with wide variation in the time required for adhesiolysis, mesh placement, and overall operative time. The variables that increased the time required for adhesiolysis included the history of chronic obstructive pulmonary disease (COPD), the presence of bowel adhesions, and a suprapubic hernia location. A suprapubic hernia location and incarceration of hernia contents significantly increased the time for mesh placement and the total operative time. The presence of bowel adhesions also significantly increased the total operative time.

Another study investigating laparoscopic ventral/incisional hernia repair for hernias in a suprapubic location resulted in increased complication and recurrence rates compared with a large study of laparoscopic ventral/incisional hernia repair that included all locations [15, 16]. Other location variability such as flank, subcostal, parastomal variations and the like also would be expected to have an impact on surgical outcomes, especially if the surgeon has had little experience performing ventral/incisional hernia repair for hernias in these atypical locations.

BMI also can be a variable with an impact on the outcomes of laparoscopic ventral/incisional hernia repair. In one study of more than 1,000 patients by Tsereteli et al. [17], morbidly obese patients had a fourfold increase in recurrence compared with non-morbidly obese patients. In addition to obesity, another patient factor that can have a significant impact on outcomes is the size of the defect and the amount or volume of the herniated contents. Outcomes such as operative time, complications, and recurrence rates for laparoscopic ventral/incisional hernia repair of small defects differ greatly from those for loss-of-domain hernias [18, 19].

A variety of factors also can be seen as having an impact on the postoperative course of patients undergoing ventral/incisional hernia repair. Studies evaluating factors related to the need for mesh removal showed that postoperative complications, recurrence rates, surgical-site infection (SSI), resource use, patient demographics (e.g., male sex, history of smoking), hernia characteristics (e.g., size of defect, incarceration), and technique factors (e.g., laparoscopic, open) all had the potential to contribute to outcome differences [20–24].

Another complex variable that can have a potential impact on outcomes of ventral/incisional hernia repair is the choice of mesh material. Although most synthetic meshes used currently produce good short-term results, any mesh could contribute to complications in a given subgroup of patients. A partial list of mesh-related complications includes infection requiring mesh removal, mesh mechanical failure, mesh bulging, chronic pain, chronic inflammatory reaction, and mesh erosion into abdominal viscera [25, 26]. With the number and variety of hernia meshes available for ventral/incisional hernia repair, this variable alone is sufficient to demonstrate that traditional research mechanisms (i.e., prospective RCTs) are inadequate to determine the mesh or meshes that have the best value for various patient groups, hernia types, techniques, surgeon skill levels, and so forth.

With an understanding of complexity science, complex systems, continuous learning, and continuous clinical quality improvement, we can begin to understand and improve value for patients who present with a ventral/incisional hernia. The starting point for this endeavor is the best current available evidence, much of which is contained in the remaining chapters of this document.

##### Summary

The traditional human subjects clinical research approach to generating EBM guidelines alone is unable to produce improved value for patient care that will be significant and sustainable for our increasingly complex health care system. Specifically, the increasing variability in ventral/incisional hernia patients and technique options minimizes the value of applying traditional research methods to improve outcomes. We need to change our thinking and learn how to understand and implement research methods designed to address this increasing complexity so we can fully address health care challenges such as ventral/incisional hernia disease. This includes not only an evolution of traditional/current EBM but also an evolution of evidenced-based management in health care.

Because complex systems research is most often applied in the real world of patient care in the community, hospital, clinic, and even the academic medical center, we need to apply the principles of continuous learning and continuous clinical quality improvement to our regular patient care in addition to using traditional clinical research methods. As we apply these new principles (new to health care, although currently used in other industries) and learn how to use complexity science–driven data analytics, the patient clusters that emerge will guide our treatment options and lead to improved value for our entire system. We should do this by including the patient in a shared decision process as well as the entire medical team caring for the person who is the patient. Our focus on improving value for the patient should be our uncompromising purpose.

### Is the routine application of computed tomography (CT) and magnetic resonance imaging (MRI) recommended for the diagnosis of ventral hernias before laparoscopic ventral hernia repair?

#### R. Schrittwieser

The Pubmed search used the following search terms: “CT-scan” AND “ventral hernia” AND “laparoscopy”; “MRI” AND “ventral hernia” AND “laparoscopy.” The search was performed in August 2011. The first search detected 53 articles. In addition, the search found 21 articles for the pre- and postoperative use of a CT scan and three articles for the use of MRI.

##### Statements

**Table:** 

Level 5	The evidence for the use of CT/MRI in the daily routine is insufficient. In some cases, especially those involving posttraumatic hernias, obese patients, large hernias with loss of domain, or special rare entities such as lumbar hernias, a CT scan or MRI can be helpful.

##### Recommendation

**Table:** 

Grade D	In special cases, such as those involving posttraumatic hernias, rare entities such as lumbar hernias or Spieghelian hernias, and connections with obesity, a CT scan or MRI may be considered.

### How important are CT and MRI in postoperative diagnosis?

#### Statement

**Table:** 

Level 2b	In the postoperative diagnosis of recurrent hernia, a CT scan is superior to a clinical examination.

#### Recommendations

**Table:** 

Grade B	A CT scan should be performed to find a recurrence or associated pathologies.
Grade D	Functional cine MRI can be used to find postoperative adhesions.

Clinical investigation ranks first for the diagnosis of ventral hernia. However, CT or MRI can be recommended in some cases for a more precise preoperative diagnosis. The available literature is most concerned with investigations involving specific entities [27–39]. Data on the use of CT and MRI are lacking for all ventral hernia types. In cases with abdominal trauma, a CT scan is recommended, among other things, to identify potential traumatic ventral hernias.

Killeen et al. [27] investigated the CT scan results for patients with blunt abdominal trauma and traumatic lumbar hernias, which showed that 9 of 14 patients had concomitant injuries and that only 1 of the 14 patients had clinical signs of a hernia. Likewise, in a retrospective series of 15 traumatic abdominal wall hernias, all correctly diagnosed by a CT scan and subsequently confirmed intraoperatively, Hickey et al. [29] reported on the high frequency of associated mesenteric and intestinal injuries. The CT scan can therefore provide valuable information concerning concomitant injuries, hernia condition, or potential hematoma.

The importance of the CT scan for diagnosing uncommon abdominal wall hernias has been demonstrated by some case reports and retrospective series [31, 33–39]. Gough and Vella [35] described the discovery of an incarcerated Spieghelian hernia as the cause of an acute abdominal pain by a CT scan. Skrekas et al. [31] highlighted the case of a patient who had swelling in the left lumbar region without trauma or previous surgery. The CT scan showed a superior lumbar hernia (Grynfeltt hernia).

For obese patients, a CT scan also can be helpful. Rose et al. [30] reported on three obese patients for whom clinical examination failed to detect a hernia. The CT scan showed a ventral hernia as the cause of the complaint. Currently, no reported studies have described the preoperative use of MRI in the diagnosis of ventral hernias. The current view is against performing a CT scan for all ventral hernias. Instead, CT is recommended for cases of obesity, repeated previous operations, large hernias with possible loss of domain, and traumatic hernias, and for the diagnosis of uncommon ventral hernias.

Currently, a number of studies [40–47] describe the use of CT scans after laparoscopic ventral hernia repair (LVHR). Gutierrez de la Pena et al. [40] reported on 50 patients with LVHR who underwent clinical investigations 1 year after surgery, including a CT scan and diagnostic laparoscopy. Recurrences were correctly diagnosed in 98 % of the cases by CT and in 88 % of the cases by clinical investigation. Wagenblast et al. [41], in a prospective study of 35 patients with LVHR, reported four patients with subsequent swelling for whom CT scan was able to differentiate between a seroma and a recurrence. Currently, reports of MRI describe only the diagnosis of adhesions after LVHR by cine MRI [40–50]. The CT scan is the method of choice for the postoperative differential diagnosis of recurrences, seroma, and bulging or residual hernias. An ultrasound investigation can be helpful in detecting seromas but does not yield the necessary anatomic details as does the CT scan to enable a firm diagnosis of recurrence [47].

### Classification

#### U. A. Dietz, F. Muysoms, M. Rohr

The following search terms were used: “incisional_hernia” AND “classification”; “ventral_hernia” AND “classification”; “incisional_hernia”; “randomized_controlled_trial.” A systematic search of the available literature was performed in January 2012 using Embase, PubMed, and the Cochrane Library, as well as a manual search of relevant references using the listed search terms. The first search detected 70 articles in Embase, 112 articles in Pubmed, and 14 articles by manual search relating to classification criteria. After excluding duplicates and articles not relevant to the key questions, 30 articles were included in the review.

##### Statements

**Table:** 

Level 5	A consensus exists among experts that it is necessary to classify ventral and incisional hernias prospectively, to create a useful data set to improve understanding of the disease, to allow comparability of results, to substantiate patient counseling, and to optimize therapeutic algorithms.

##### Recommendations

**Table:** 

Grade D	It is recommended that ventral and incisional hernias be classified before surgical therapy.
	The European Hernia Society (EHS) classification for ventral and incisional hernias is recommended.

#### Are the classification criteria included in the EHS classification consistent?

##### Statements

**Table:** 

Level 2B	Numbers of previous repairs and reducibility have been demonstrated to increase the risk of postoperative seroma.
Level 2C	Risk factors have been shown to influence the incidence of repeat recurrences.
Level 3	The incidence of SSI is increased in patients with recurrent incisional hernias and chronic steroid use as well as among smokers.
	The morphology and size of the hernia may influence the type of procedure.
	Findings show the width of the hernia gap to be a predictive factor for postoperative complications and the length of the hernia to be an independent prognostic factor for repeat recurrences.
Level 4	Risk factors, hernia gap size, and morphology can influence the time needed for the surgical procedure.
	Smoking, male gender, BMI, age, SSI, and postoperative wound complications are risk factors for the development of an incisional hernia.

##### Recommendations

**Table:** 

Grade B	Number of previous repairs, morphology, size of the hernia gap, risk factors and reducibility should be part of any classification system and should be recorded in the patient files.
Grade C	Risk factors, hernia gap size, and morphology should be part of any classification. They should be considered in planning (tailoring) the surgical procedure.
	No known algorithm exists that reduces the incidence of SSI in patients with risk factors. These patients should be informed about the increased risk during preoperative counseling.

### Is it necessary to classify ventral and incisional hernias as well as which classification should be recommended?

Classification systems are necessary to structure the way scientific knowledge is collected and analyzed. Because the documented benefit flowing from the introduction and use of the tumor-node-metastasis tumor classification and the International Classification of Diseases, the value of other classification systems for diagnostic, therapeutic, and prognostic decision has been confirmed, aside from their benefits in patients counseling.

Classifications for ventral and incisional hernias were first proposed by Chevrel and Rath [52], followed by Korenkov et al. [63], Ammaturo et al. [51], Chowbey et al. [53], Dietz et al. [56], Muysoms et al. [70], and Hadeed et al. [58]. Some agreement exists regarding the basic criteria of morphology and size of the hernia gap, although none has gained widespread acceptance in the literature. The classification proposed by the European Hernia Society (EHS) [70] is the result of a comprehensive discussion of the criteria to be included and also of how to precise and define them. The EHS classification generally is regarded as an improvement on the previous classifications.

### Are the classification criteria included in the EHS classification consistent?

**Fig. 1:**
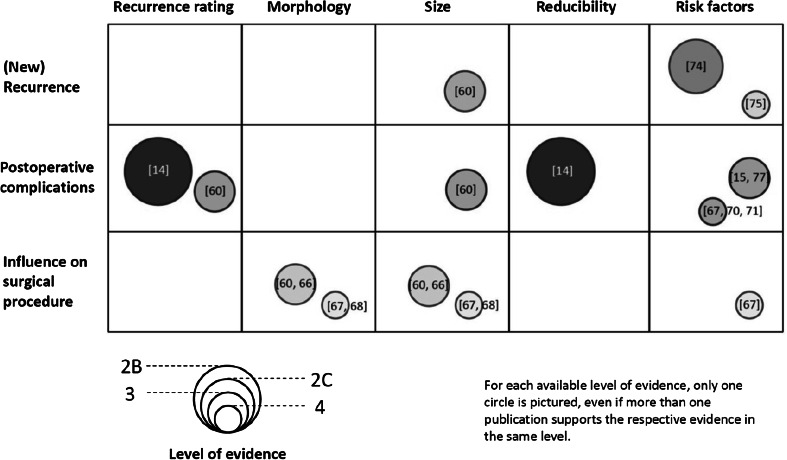
Correlation between the classification criteria, the incidence of a repeat recurrence, and postoperative complications as well as the influence on decision making regarding the surgical approach.* Circles* are sized proportionally to the available level of evidence, with respective references cited in each* circle*

The following discussion illustrates the clinical importance of the classification criteria [61, 77]. The scarcity of evidence is illustrated in Fig. 1. Recurrence rating provides important information on the patient’s hernia history. The term “recurrence rating” comprises first the distinction between ventral and incisional hernias and second the designation of incisional hernias in the subcategory of recurrent incisional hernias. The number of previous repairs has been demonstrated to increase the risk of postoperative seroma [60]. The incidence of SSI is increased in patients with recurrent incisional hernias [57] and is related to the surgical technique [62]. The incidence of postoperative complications is twice as high among patients with incisional hernias as among those with ventral hernias [57].

In morphologic terms, the EHS classification defines median and lateral hernias. Morphology may influence the type of procedure, for example, in the subxiphoidal area [54, 55, 57, 66] or in the suprapubic region [15, 57]. In a nonrandomized clinical trial involving 199 patients, lateral incisional hernias had a different clinical presentation than medial hernias, with more preoperative pain and more postoperative complications [69].

The location of the hernia is of importance for the surgical strategy. Proximity to bony structures, tension in closing the gap, and the composition of the fascia layers need to be considered [14, 57, 65]. The location of the hernia correlates with the operative time [14]. In the future, comparison of data regarding surgical approach, layer of mesh insertion, quality of life, and morphology will be included in comparative studies [71, 72].

The EHS classification requires measurement of the gap size during the surgical procedure. There is a consensus that the length of the hernia gap should be the greatest longitudinal distance between the proximal and distal margins of the hernia gaps, as it also should be for the width in the transversal axis [70, 71].

Hernia width is a useful intraoperative variable in tailoring surgical procedures [15, 57, 72, 73]. Findings have shown the width of the hernia gap to be a predictive factor for postoperative complications and the length of the hernia to be an independent prognostic factor for repeat recurrences [57]. Hernia gap size also can influence the time needed for the surgical procedure and serves as a marker of operative complexity [14, 64]. Related to the hernia gap is the reducibility of the sac contents. Nonreducible incisional hernias have been shown to correlate significantly with a seroma [21, 60].

Risk factors were studied in large cohort series [59, 76] and potential risk groups [73]. Smoking, male gender, BMI, age, SSI, and postoperative wound complications are risk factors for the development of an incisional hernia [14, 57, 67, 72–75]. According to experimental evidence, patients with incisional hernias have an imbalance in the collagen metabolism [62]. Risk factors have been shown to influence the incidence of repeat recurrences [57]. Because risk factors and comorbidities are not understood to date, the working group of the European Registry of Abdominal Wall Hernias (EuraHS at www.eurahs.eu) introduced the definition of the severity of comorbidity (SOC) score to refine further the influence of risk factors on the course of ventral and incisional hernias [71]. Risk factors should be considered in tailoring the surgical procedure and in counseling the patient regarding the expected postoperative course and prognosis of recurrence during late follow-up evaluation (Table 1).

**Table 1:** Literature overview of classification systems and the corresponding evidence of each criterion

Author	Years	Type of study	Oxford	New classification	Use of a classification	Recurrence rating	Morphology	Size	Risk factors	Surgical procedure
Ammaturo and Bassi [51]	2005	Case series	4	X			X	X		
Chevrel and Rath [52]	2000	Expert opinion	5	X						
Chowbey et al. [53]	2006	Expert opinion	5	X						
Conze et al. [54]	2005	Experimental	5				X			X
Conze et al. [55]	2007	Case series	4				X			X
Dietz et al. [56]	2007	Expert opinion	5	X						
Dietz et al. [57]	2012	Retrospective case–control	3		X	X	X	X	X	
Hadeed et al. [58]	2011	Case series	4	X						
Höer et al. [59]	2002	Outcome study	2c						X	
Jenkins et al. [14]	2010	Case series	4				X	X	X	
Kaafarani et al. [60]	2009	RCT	2B			X				
Kaafarani et al. [21]	2010	RCT	2B			X		X		
Kingsnorth [61]	2006	Review	5							X
Klinge et al. [62]	2001	Experimental	5						X	
Korenkov et al. [63]	2001	Expert opinion	5	X						
Leblanc et al. [64]	2001	Retrospective cohort	4					X		
Licheri et al. [65]	2008	Case series	4		X		X			
Losanoff et al. [66]	2007	Review	5				X			X
Martínez-Serrano et al. [67]	2010	Retrospective cohort	3						X	
Moreno-Egea et al. [68]	2007	Review	5				X			
Moreno-Egea et al. [69]	2008	NR-controlled trial	3				X			
Muysoms et al. [70]	2009	Expert opinion	5	X	X	X	X	X	X	
Muysoms et al. [71]	2012	Expert opinion	5		X				X	
Parker et al. [72]	2011	Retrospective cohort	4					X		
Piardi et al. [73]	2010	Retrospective cohort	4				X	X	X	
Sanchez et al. [74]	2011	Review	5						X	
Sørensen et al. [75]	2005	Retrospective cohort	3						X	
Varnell et al. [15]	2008	Case series	4			X	X	X		
Veljkovic et al. [76]	2009	Case series	4						X	
Winkler et al. [77]	2008	Review	5		X	X	X	X		X

*RCT* randomized controlled trial, *NR* nonrandomized

## Indication for surgery

### Indications for treatment: dependence on size of defect or hernia sac, hernia type, symptoms, and age

#### Thomas Simon

A systematic search was performed in PubMed, Medline, the Cochrane Library, and the Study Register, as well as a search of relevant journals and reference lists including publications until 6 June 2012. The following search terms were used in the search strategy: “operation” AND “watchful waiting” AND (“Hernia” [Mesh]) OR “Hernia; Abdominal” [Mesh] OR “Hernia; Ventral” [Mesh] OR “Hernia; Umbilical” [Mesh] OR (“Abdominal wall hernias”) OR (“ventral hernia”) OR (“epigastric hernia”) OR (“incisional hernia”) AND (randomized controlled trial[pt] OR controlled clinical trial[pt]). The search produced 462 hits including inguinal hernias. Of the 42 relevant papers found, 28 were selected for this analysis. The only two level 1b trials addressed inguinal hernias and were included with intention to discuss the existing evidence in a related field. Regarding data addressing ventral and incisional hernias, only one level 3 study and 15 level 4 uncontrolled studies were found.

##### Statements

**Table:** 

Level 4	Symptoms develop for 33–78 % of patients with a ventral or incisional hernia.
Level 4	Surgery is performed for 5–15 % of patients with a ventral or incisional hernia because of an acute complication (obstruction/strangulation).
	Emergency repairs are associated with high morbidity.
	Umbilical hernias obstruct five times more often than other ventral and incisional hernias.
Level 4	The defect size of incisional hernias predicts recurrence rates.
Level 4	Findings seem to indicate no difference in terms of morbidity or mortality regarding laparoscopic surgery for ventral hernias in advanced age.
	The reduced risk of SSI in laparoscopic techniques has an impact especially for elderly patients.

##### Recommendations

**Table:** 

Grade D	Symptomatic ventral and incisional hernias should be treated surgically.
Grade D	The laparoscopic technique for ventral and incisional hernias should preferably be reserved for defect sizes smaller than 10 cm in diameter.
Grade D	The laparoscopic technique for ventral and incisional hernia repair can be used even for patients advanced in age.

No precise data on the incidence and prevalence of ventral and incisional hernias are available. An epidemiologic study showed an increasing proportion of midline abdominal wall hernias, with a relative frequency of 19 % for umbilical/par umbilical hernias, 8.6 % for epigastric hernias, and 4.8 % for incisional hernias [78]. The incidence for incisional hernia is 10–20 % [79, 80], making it one of the most common surgical complication after laparotomy.

Ventral and incisional hernias are treated with surgery to relieve symptoms (pain and discomfort), to prevent complications (strangulation, respiratory dysfunction, or skin problems), or to resolve acute complications (incarceration and strangulation) [95].

##### Symptoms

The study found seven publications dealing with symptoms, including two database studies [84] and one a questionnaire study [82]. A large study with a long-term follow-up period (≤10 years) including 564 patients, showed that 11 % of patients experience an incisional hernia, with 33 % having symptoms and 14 % experiencing obstruction [80]. In a retrospective review of 959 patients after liver transplantation, Vardanian et al. [83] found an incisional hernia rate of 4.6 %, with 78 % of the hernias being symptomatic (pain and discomfort) and 5 % presenting with incarceration or strangulation. In the series of Courtney et al. [86], 78 % of the patients underwent surgery because of pain, whereas 10 % presented acutely [87], and in the series published by Hjaltason, umbilical hernias were incarcerated five times more often than incisional hernias.

##### Acute hernia

Emergency repairs of acute abdominal hernia are associated with a high morbidity rate [81, 88, 93]. In patients managed by a “watchful waiting” strategy, Davies et al. reported a significant proportion who presented with acute hernia. The series of Alani et al. comprised an interestingly high rate of acute ventral hernia amounting to almost 50 % of the prospectively reviewed population. As a percentage of all the hernias managed by surgery during the study period, the 12.2 % incidence of acute ventral hernias still is high [89]. Regarding pediatric umbilical hernia, a retrospective review of 489 children showed a 7 % rate of presentation with acute hernia [90]. Earlier studies showed an incarceration rate of 14.6 % and a strangulation rate of 2.4 % [91].

##### Age

Only one retrospective study providing level 4 evidence and involving 155 patients addressed the question whether advanced age is a contraindication for laparoscopic ventral hernia repair. The study population was divided in two groups based on a threshold at 65 years. The authors did not find any significant difference regarding morbidity and mortality [92]. The Cochrane Review [106] comparing laparoscopic and open surgical techniques for ventral and incisional hernia repair showed a clearcut and consistent reduced risk for SSI in the laparoscopic group, and this review has had a great impact on hernia surgery among the elderly.

##### Indication related to size

The systematic search provided only one article on defect size and outcome [96]. Moreno-Egea et al. performed a prospective study without a control group that excluded hernias smaller than 5 cm in diameter and those with “Swiss cheese” defects. The data analysis showed that size correlates with recurrence, and these authors recommended restriction of the laparoscopic approach to a hernia size of 10 cm or smaller (level 4). A retrospective single-center study of 302 patients who underwent open repair of primary incisional hernia analyzed several risk factors for recurrence and demonstrated that the size of the hernia is a significant risk factor for recurrence [97].

##### Asymptomatic hernias

The search found no publications on the natural history of the condition. One long-term prospective study and one review showed 60 % of patients with incisional hernias to be symptom free [80, 81]. An international questionnaire among hernia specialists showed a rate of 23 % for asymptomatic cases, and more than 20 % of the patients did not undergo operative repair. The strangulation/incarceration rate was 5 % [82].The group concluded that there are no hard data describing the natural course of an incisional hernia. Their current view is that patients with asymptomatic incisional hernias should be undergo surgery to avoid complications.

Precise data on the strangulation rate or the risk for acute incarceration of incisional hernias are unavailable. One small prospective case study reported an emergency operation rate of 3.2 % [103].The data from the Danish Ventral Hernia Database published by Helgstrand et al. [84] showed an acute hernia rate of 10 %, with umbilical hernias showing the highest rate (57 %). No controlled studies have investigated the increase in size of incisional hernias over time, the risk factors for strangulation, or the development of discomfort and pain.

A prospective case study involving consecutive patients investigated whether patients obtain pain relief from surgery [103]. This study found no benefit regarding pain for patients with minimal symptoms. Two prospective trials have been launched to address this question relating to the indication for surgery among asymptomatic and minimally symptomatic incisional patients. The multicenter AWARE trial of Lauscher et al. [104] is the multicenter study in the recruiting phase and the second trial that has completed intake and data collection but has not been published to date [105]. Hence, no conclusive data exist currently, but the issue is likely to be resolved with publication of the results from the two trials.

### Is there still a place for open suture repair depending on defect size?

#### J. Kukleta, Th. Simon, S. Morales-Conde

In August 2011 and April 2012, a systematic search of the literature was performed using Pubmed, Medline, and the Cochrane Library, as well as a search of other relevant journals and reference lists. The following search terms were used: “small hernia” AND “non-mesh repair” AND “suture repair” AND “recurrence” AND “infection” AND “umbilical hernia” AND “incisional hernia” AND “ventral hernia.” The search yielded 277 papers, metaanalyses, RCTs, and reviews on umbilical hernia (UB). These publications included, 100 UB and suture repair articles, 54 UB and recurrence articles, and 21 UB and infection articles. For epigastric hernia (EH), we found 26 publications (metaanalyses, RCTs, and reviews). For small hernia (SM), we found 433 articles with filter metaanalysis, RCT, and reviews. From this material, 45 relevant papers were chosen for this review, including 19 papers with an evidence level of la or lb, four papers with an evidence level of 2, 14 papers with and evidence level of 3, and six papers with an evidence level of 4.

##### Statements

**Table:** 

Level 1B	Suture herniorrhaphy is the simplest procedure among the open repair techniques.
	Suture repair is associated with a high recurrence rate.
	Suture repair is accomplished in a shorter operative time than mesh repair.
	Mesh repair reduces the recurrence rate significantly compared with suture repair.
	Mesh repair seems to be a safe method even in the presence of nonviable bowel loops in an incarcerated umbilical hernia.
	Wound complication rates can be slightly higher in mesh repair or similar in the two groups.
Level 3	Independent risk factors for recurrence of small hernias are not clearly defined. Hernia size, BMI, or wound infection in one study, and smoking, obesity, size of hernia, type of repair, and COPD in another study do not seem to predict recurrence in small hernia repair.
Level 4	Not every “small hernia” requires mesh repair.
	Suture repair of hernias smaller than 2 cm shows an acceptable recurrence rate and low wound morbidity.
Level 5	Despite the existing evidence, suture repair still is very popular in the surgical community

##### Recommendations

**Table:** 

Grade A	For repair of primary defects larger than 2 cm or recurrent hernias of any size, mesh repair should be considered as the first choice.
Grade C	Suture repair should be used only for very small primary defects of the abdominal wall in the absence of any possible recurrence risk factors.
Grade D	In terms of recurrence, the available evidence is sufficiently strong to recommend that all defects of the abdominal wall, whether inguinal, incisional, or umbilical hernias, and of whatever size should be repaired with the use of prosthetic mesh.

Most studies investigating the treatment of small abdominal wall hernias published between 2000 and 2012 recommend mesh for the repair due to the unacceptable high recurrence rate after suture repair. The term “small hernia” often is used, although it has never been precisely defined. The consensus is that it involves a defect 2 cm in size or smaller. The vast majority of surgeons worldwide continue to repair the small hernia by suture despite the clear message of Burger et al. [148] in 2004 that “suture repair should be abandoned.”

In 2001, Arroyo et al. [108] reported an RCT comparing suture and mesh repair of umbilical hernia in adults. The recurrence rate for suture repair was 11 %, significantly higher than the 1 % for mesh repair (*p* = 0.0015). In 2010, Aslani and Brown [110] published a metaanalysis of RCTs together with an extensive review. All the RCTs favored mesh repair in terms of recurrence, as did 8 of 10 cohort studies.

Wound complication rates are slightly higher for mesh repair in RCTs but equal between the two groups in cohort studies. The retrospective comparison of mesh and suture repair by Sanjay et al. [126] showed recurrence rates for mesh of 0 versus 11.5 % for suture repair. The infection rate for mesh repair was 0 versus 11.5 % for suture repair.

In 2009, Stabilini et al. [130] reported the 10-year recurrence rate of 14.7 % for suture repair versus 3.1 % for mesh repair (*p* = 0.0475). Eryilmaz et al. [124] demonstrated in a prospective comparison that all umbilical hernias regardless of the size should be repaired by polypropylene (PP) mesh. However, in contrast to the aforementioned studies, Dur et al. [145] reported a low recurrence rate after suture repair and advised that not every small hernia needs a mesh repair.

##### Risk factors

The independent risk factors for recurrence in small hernia repair are not well defined. Asolati et al. [135] reported that smoking, obesity, size of hernia, type of repair, and COPD do not seem to predict recurrence of hernias. Halm et al. [137] could not establish a relationship between a BMI higher than 30 kg/m^2^ and an increased recurrence rate but did establish a relationship between a BMI higher than 25 kg/m^2^ and a recurrence increase from 5 to 18 %. Arroyo et al. [108] did not find any significant relationship between recurrence rate and hernia size. The recurrence rates were similar for defects larger and smaller than 3 cm. A BMI higher than 30 kg/m^2^ was a risk factor for umbilical hernia recurrence. In their retrospective analysis of recurrence rate after mesh-free Spitzy’s repair, Schumacher et al. [149] reported a clear correlation between hernia size or a BMI higher than 30 kg/m^2^ and the recurrence rate (Table 2). According to their results, a patient with BMI higher than 30 kg/m^2^ or a hernia larger than 3 cm needs a mesh repair.

**Table 2:** Umbilical hernia repair: published data on patients and results

Author	Study	No. of patients	OM/Rec *n* (%)	LM/Rec *n* (%)	ONM/Rec *n* (%)	Wound infection OM/LM/ONM (%)
Abdel-Baki	RCT	42	21 (0)		21 (19)	
Arroyo	RCT	200	(1)		(11)	Similar
Polat	RCT	50	17 PHS	15 Onlay	18 Mayo	
Aslani	Sys rev		(1)		(11)	
Asolati	Retrosp	229	132 (3)		97 (7.7)	
Bowley		473	80 (2.5)		393 (4)	
Ergul	Case series	10 + Lapchol	(0)			
Eryilmaz	Prosp	111	48 (2)		63 (14)	
Farrow	Retrosp	152	(1.5)		(9.2)	19
Gonzales	Retrosp	76	20 (20)	32 (0)	24 (8)	15/0/0
Halm	Retrosp	131	12 (0)		119 (13)	
Kamer	Retrosp	64	14		50	
Lau	Retrosp	102	9 (0)	26 (0)	43 + 24 (8.7)	
Malik	Retrosp	236	(7.4)		(22.7)	
Solomon	Retrosp	724	227 (1.8)	301 (1.0)	146 (30)	1.3/2.2/5.5
Sanjay	Retrosp	100	39 (0.0)		61 (11.5)	0.0/11.5
Stabilini	Retrosp	98	64 (3.1)		34 (14.7)	1.4
Venclauskas	Retrosp	97	5		92	
Wright	Retrosp	116	20	30	66	

*OM* open mesh repair, *Rec* recurrence, *LM* laparoscopic mesh repair, *ONM* open nonmesh repair, *RCT* randomized controlled trial, *PHS* polypropylen hernia system, Mayo, *Lapchol* laparoscopic cholecystectomy, *Sys rev* systematic review, *Retrosp* retrospective, *Prosp* prospective

### Limitations of laparoscopic intraperitoneal onlay mesh (IPOM) repair in relation to defect size or body habitus

#### J. Bingener, M. Rohr

The following search terms were used: “hernia” AND “ventral” AND “laparoscopy” AND “laparoscopic surgery” AND “postoperative complications or recurrence or pain” AND “postoperative or surgical wound infection” AND “prosthesis” AND “design/failure/implantation/device removal” AND “seroma” AND “pain” AND “limitations.” The search resulted in a total of 946 citations identified in Ovid Medliner from 1948 to August 2011, PubMed including prepublication, Embase from 1988 to the 33rd week of 2011, EBM reviews and the Cochrane Register, and the Web of Science from 1993 to 2011. Of these, 17 full papers were relevant to the topic and included in the review.

##### Feasibility with regard to obesity: statements

**Table:** 

Level 3	Laparoscopic IPOM is feasible for obese patients (BMI > 30 kg/m^2^).
Level 3	Laparoscopic IPOM is feasible for morbidly obese patients (BMI > 40 kg/m^2^).
Level 3	Laparoscopic IPOM is feasible for super morbidly obese patients (BMI > 50 kg/m^2^).
Level 4	Laparoscopic IPOM is feasible for patients with a BMI up to 82 kg/m^2^.

##### IPOM feasibility in relation to hernia size: statements

**Table:** 

Level 3	Laparoscopic IPOM is feasible for defects larger than 15 cm.
Level 2B	Hernia recurrence is more likely with defects wider than 10 cm.
Level 3	The operating time is longer with defects larger than 15 cm.
Level 2B	Mesh sizes up to 1,250 cm^2^ can be used.
Level 4	Mesh sizes up to 2,400 cm^2^ can be used.
Level 4	LVHR is feasible for defects of up to 880 cm^2^.

##### Morbidity and obesity: statements

**Table:** 

Level 3	Complication rates in patients with a BMI ≥ 40 kg/m^2^ undergoing LVHR are higher than for patients with a BMI < 40 kg/m^2^.
Level 2B	The recurrence rate is increased with BMI > 30 kg/m^2^.

##### Recommendations

**Table:** 

Grade B	Obese patients should be informed that LVHR is feasible.
Grade B	Patients should be informed that the risk of complications and hernia recurrence increases with BMI.
Grade B	Patients should be informed that complications and wound infections are less likely with LVHR for obese patients than with the open approach.

##### LVHR versus open repair for large hernia: statements

**Table:** 

Level 2B	LVHR requires the use of larger mesh sizes than open hernia repair.
Level 2B	LVHR results in fewer superficial SSIs than open repair of large hernias.
Level 2B	LVHR results in less blood loss than open repair of large hernias.
Level 3	LVHR is associated with less use of postoperative narcotics than open repair.
Level 3	LVHR is associated with a shorter hospital stay than open repair.
Level 3	LVHR of large hernias is associated with less ileus than open repair.

##### Recommendations

**Table:** 

Grade B	Patients should be informed that LVHR for large hernia defects is feasible.
Grade B	Patients should be informed that LVHR for large hernias reduces the incidence of superficial SSIs compared with open repair.
Grade B	Patients should be informed that LVHR for large hernias is accompanied by less blood loss than open repair.
Grade B	Patients should be informed that LVHR for large hernias results in a shorter hospital stay than open repair.

This issue is hampered by the limited quality and number of retrospective studies [15, 43, 150–168]. Large hernia is poorly defined. Existing classifications (EHS) are not used consistently. Some studies consider a large hernia to be greater than 5 cm in diameter, whereas others consider a diameter greater than 10 or 15 cm as large, and one study defined a hernia larger than 20 cm to be a giant hernia.

It is important to stress that the level of recommendation in statements on SSI is extrapolated from metaanalyses and RCTs for overall infection outcomes of LVHR versus open ventral hernia repairs.

### Obese patients and incisional hernia

#### F. Köckerling, P. Chowbey

The following search terms were used: “incisional hernia”; “ventral hernia”; “incisional hernia and obesity”; “ventral hernia and obesity”; “laparoscopic incisional hernia repair”; “laparoscopic ventral hernia repair (LVHR)”; “LVHR and obesity”; “LVHR and complications”; “LVHR and wound infections”; “LVHR and defect Size.”

A systematic search of the available literature was performed in July 2012 using Medline, PubMed, and the Cochrane Library, as well as a search of relevant journals and reference lists using the aforementioned search terms. The first search yielded 35 articles. The review is based on nine key publications.

##### Statements

**Table:** 

Level 1A	Laparoscopic ventral and incisional hernia repair is associated with fewer wound infections.
Level 2A	Laparoscopic ventral and incisional hernia repair is associated with significantly fewer wound complications.
Level 2B	Obese patients (BMI > 30 kg/m^2^) have significantly larger defect sizes in laparoscopic incisional hernia repair.
Level 3	A BMI higher than 30 kg/m^2^ or a defect larger than 8–10 cm significantly increases the risk of recurrence.
	The early outcome of LVHR does not differ significantly between non-morbidly obese (BMI < 35 kg/m^2^) and morbidly obese (BMI ≥ 35 kg/m^2^) patients.

##### Recommendations

**Table:** 

Grade A	For obese patients presenting with a ventral or incisional hernia, the laparoscopic approach is preferred because it reduces the wound infection rate and complications.
Grade B	For patients with a BMI of 35 kg/m^2^ or higher, laparoscopic ventral and incisional hernia repairs may be preferred.
	In obese patients, the defect sizes are significantly larger, something that must be considered when the laparoscopic approach is advised.
	For obese patients (BMI ≥ 30 kg/m^2^) with a defect size greater than 8–10 cm, there may be a need for additional technical steps (greater mesh fixation, more overlap, suture closure of the defect) when the laparoscopic approach is indicated.

Obesity is a risk factor for occurrence of incisional hernias and leads to higher perioperative complication and recurrence rates after open repair. There are multifactorial reasons for this, such as delayed wound healing, impaired pulmonary function, and higher intraabdominal pressure [163]. Metaanalyses of prospective randomized studies comparing laparoscopic repair of incisional and ventral hernias with open repair have shown a significantly lower rate of wound infections, with no removal of the mesh, for the laparoscopic IPOM technique (level 1A) and a trend toward lower infection rates with mesh removal (level 1A) using the laparoscopic technique [113].

In the metaanalysis of Sauerland et al. [106], the local infection rate in the laparoscopic group was 3.1 versus 13.4 % in the open group (*p* < 0.00001). A local infection requiring mesh removal was found in 0.7 % of the laparoscopic group and 3.5 % of the open group (*p* = 0.09).

In an analysis of pooled data on 4,582 laparoscopic and 758 open repairs of incisional and ventral hernias, Pierce et al. [169] found a wound complication rate of 3.8 % for the laparoscopic procedure and 16.8 % for the open technique (*p* < 0.0001) (level 2A). The significantly lower rate of wound complications attests to the benefits of using the laparoscopic technique, especially for obese persons, who are at higher risk for wound complications. In a metaanalysis of cohort studies, Mavros et al. [170] observed a trend toward higher mesh infection rates for obese patients after open ventral hernia repair.

However, a larger abdominal wall defect must be expected in obese patients with an incisional hernia. A study by Moreno-Egea et al. [96] demonstrated that in patients with a BMI higher than 30 kg/m^2^, the proportion of defects larger than 10 cm was 35.1 %. However, 60 % of the patients with a defect size of 10–12 cm had a BMI higher than 30 kg/m^2^, and the proportion of patients with defects larger 12 cm rose to 73.5 % (level 2B). Accordingly, a larger defect for an incisional hernia must always be expected in obese persons.

During a mean follow-up period of 5 years after laparoscopic IPOM repair of incisional hernias, the study by Moreno-Egea et al. saw recurrences in 0.4 % of the patients who had defects smaller than 10 cm, in 20 % of those with defects 10–12 cm in size, and in 41.2 % of those with defects larger than 12 cm. Accordingly, significant differences were noted in the defect sizes, the BMI, and the proportion of patients with a BMI higher than 30 kg/m^2^ between the recurrence and the nonrecurrence groups. In the former group, the mean BMI was 36.3 ± 6.3 versus 29.5 ± 5.9 kg/m^2^ in the nonrecurrence group (*p* < 0.001). The proportion of patients with a BMI higher than 30 kg/m^2^ was 90 % in the recurrence group and 37.9 % in the nonrecurrence group (*p* < 0.001). The mean defect size was 14.4 ± 2.9 cm in the recurrence group and 7.9 ± 2.9 cm in the nonrecurrence group (*p* < 0.001).

Thus, patients with a BMI higher than 30 kg/m^2^ have significantly larger defects and higher recurrence rates, especially patients with a defects larger than 8–10 cm. Accordingly, additional technical steps are needed to prevent recurrence in these patients, such as the use of a larger mesh to ensure more extensive mesh overlap and stronger fixation of the mesh or even suture closure of the defect.

A comparison of early postoperative outcomes between patients with a BMI lower than 35 kg/m^2^ and those with a BMI of 35 kg/m^2^ or higher (level 3) showed no significant differences in the rate for enterotomies, hematomas, seromas, enterocutaneous fistulas, or postoperative infections [171]. For 163 patients with a BMI higher than 30 kg/m^2^, Novitsky et al. [172] reported a mortality rate of 0 % after laparoscopic repair of incisional and ventral hernias, a conversion rate of 3.1 %, a postoperative complication rate of 12.3 %, a wound infection rate of 1.2 %, and a mesh-related infection rate of 1.2 %. Raftopoulos and Courcoulas [43] reported no mortality in their patients with a BMI of 35 kg/m^2^ or higher, but the wound infection rate was 3.7 %, the bladder injury rate was 3.7 %, and the postoperative ileus rate was 11.1 %.

### Recurrence after open surgery: redo better laparoscopically?

#### R. Schrittwieser

The following search terms were used: (open[All Fields] AND (“hernia, ventral”[MeSH Terms] OR (“hernia”[All Fields] AND “ventral”[All Fields]) OR “ventral hernia”[All Fields] OR (“ventral”[All Fields] AND “hernia”[All Fields]) AND (“recurrence”[MeSH Terms] OR “recurrence”[All Fields]). The first search detected 270 articles, but only five articles could be used for the review.

##### Statement

**Table:** 

Level 4	Some evidence indicates that reoperation for recurrence after open repair is better performed laparoscopically.

##### Recommendation

**Table:** 

Grade C	Some cases of recurrence after open repair are better managed laparoscopically provided the surgeon has sufficient experience in laparoscopic ventral hernia repair.

Reoperations for recurrence of ventral hernia are challenging. Currently no evidence-based recommendations of optimal management exist. In cases of recurrence after previous open suture repair, the decision concerning the approach (open or laparoscopic surgery) is similar to that for the primary incisional hernia [106, 168, 173].

After open mesh repair, reoperation by the laparoscopic approach has certain advantages. First, the repeat operation is performed at a different site/level of the abdominal wall. Second, in all instances, the entire incisional scar can be covered by a mesh. Usually, it is not necessary to remove the previously inserted mesh, hence avoiding an extensive dissection of the abdominal wall.

Uranues et al. [174] demonstrated that with sufficient expertise, laparoscopic reoperation can be performed with moderate recurrence rates, even after multiple previous repairs. A possible advantage of laparoscopic reoperation is the identification of previously undiscovered recurrent hernias, which can be undertaken during the same intervention. Sharma et al. [42] reported 203 occult hernias (16.3 %) in their series of 1,242 laparoscopic ventral hernia repairs during a period of 13 years.

## Perioperative management

### Evidence for antibiotic and thromboembolic prophylaxis in laparoscopic ventral hernia surgery

#### Rudolf Schrittwieser

The following search terms were used: “ventral hernia” AND “antibiotic prophylaxis”; “ventral hernia” AND “antibiotic prophylaxis” AND “laparoscopy”; “ventral hernia” AND “antibiotic prophylaxis” AND “randomized studies”; “abdominal wall hernia” AND “antibiotic prophylaxis”; “ventral hernia” AND “thromboembolic prophylaxis”; “hernia” AND “thromboembolic prophylaxis” AND “laparoscopy”; “ventral hernia” AND “thromboembolic prophylaxis” AND “randomized studies”; “abdominal wall hernia” AND “thromboembolic prophylaxis.” The search was performed in August 2011. The first search yielded 24 articles, 13 of which were relevant for this review.

##### Statements

**Table:** 

Level 2b	Antibiotic prophylaxis in ventral hernia repair is associated with significantly fewer local infections.
Level 5	The evidence for routine thromboembolic prophylaxis in laparoscopic ventral hernia repair is insufficient.

##### Recommendations

**Table:** 

Grade B	Routine antibiotic prophylaxis in ventral hernia repair is recommended.
Grade D	Thromboembolic prophylaxis should be given in accordance with the presence of risk factors for the individual patient.

##### Antibiotic prophylaxis

Antibiotic prophylaxis in hernia surgery remains a subject for debate. Both grade D [175] and grade B [98] recommendations can be applied to laparoscopic inguinal hernia surgery. However, there is significantly more literature on antibiotic prophylaxis for inguinal hernia surgery than for ventral hernia. The rate of infection with LVHR in specific studies can be as high as 16 %, but it usually is much lower, ranging from 0.5 to 4 % [182].

Two level 2b studies are available. Ríos et al. [176] reported a significant difference between hernia surgery with and without the use of prophylactic antibiotics (*p* = 0.00991). However, this was a nonrandomized investigation of patients who had undergone open repair with mesh implantation, in which the two patient groups differed in numbers (140 with prophylaxis and 76 without prophylaxis), and the rate of infection (18.1 %) was on the high side. Abromov et al. [177] concluded from their series of open repairs that single-dose antibiotic prophylaxis has a positive effect on the wound infection rate after repair of umbilical and incisional hernia. The wound infection rate was 1 of 17 in the antibiotic prophylaxis group compared with 8 of 18 in the nonantibiotic prophylaxis group.

Three level four studies are available. White et al. [179] reported on 250 hernia operations in 206 patients over a period of 14 years. Neither antibiotics nor drainage had any influence on the rate of wound complications. Deysine [180] in a retrospective study of more than 4,000 inguinal and 350 clean ventral hernia operations reported an infection rate of 0.11 %. The antibiotic prophylaxis involved 1 g of cefazolin given intravenously 1 h before the operation, and the protocol included additional frequent intraoperative wound flushing with a solution comprising 80 mg of gentamicin in 250 ml of NaCl. A further retrospective study by Edwards et al. [178] reported on 65 cases of laparoscopic ventral hernia repair designed to establish the rate of seroma-associated cellulitis. Before surgery, all the patients had received a third-generation cephalosporin, and in addition, 45 of the 65 patients received either cephalosporin or fluoroquinolone orally during 7 days after the operation. The rate of seromas was equal in the two groups, but all the patients who received only antibiotics preoperatively experienced cellulitis, whereas in the pre- and postoperative group, the rate was only 40 %. However, the study dealt with a small and a very heterogeneous sample of patients.

Some studies advocate the routine use of prophylactic antibiotics ranging from administration of amoxicillin (1 g) and clavulanic acid (200 mg) before surgery and then 8 h after the operation [181] to administration of a second-generation cephalosporin at the start of the anesthesia and then 24 h after the operation [183] to administration of a first-generation cephalosporin at the time of the skin incision and then again for operations lasting longer than 2 h [16].

From the available studies, a clear recommendation for or against the use of antibiotic prophylaxis cannot be drawn. It appears advisable, however, to consider administering a prophylactic antibiotic for patients with risk factors (advanced age, administration of corticosteroids, immunosuppressive therapy, obesity, diabetes, or malignant tumor) and for cases with surgical risk factors (contamination, long operation duration, drainage, urinary catheter).

##### Thromboembolic prophylaxis

Some studies seem to suggest a higher risk after laparoscopic interventions [184]. The increased intraperitoneal pressure and the reversed Trendelenburg position possibly account for this. No RCTs on the efficacy of thrombosis prophylaxis in LVHR and available. In terms of thromboembolic prophylaxis and incidences of thromboembolic complications after laparoscopic surgery, a prospective study [185] investigating a total of 2,384 patients reported 8 cases of deep vein thrombosis (DVT). However, there were no cases of pulmonary embolism. In six of the cases, pneumoperitoneum lasted for more than 2 h, and for more than 3 h in two cases. The authors concluded that heparin prophylaxis should be continued at least until discharge for these patients.

## Key-points of the technique

### Positioning of the trocars and creating the capnopneumoperitoneum

#### M. Rohr, Y. Trommer

The following search terms were used: “laparoscopic hernia repair” AND “LVHR” AND “incisional hernia” AND “ventral hernia” AND “capno/peritoneum” AND “trocar position” AND “laparoscopic insufflation” AND “CO_2_ insufflations laparoscopic.” In August 2011, a systemic search of the available literature was performed using Medline, PubMed, the Cochrane Library, as well as a search of relevant journals and reference lists using the aforementioned search terms. The search detected 13 relevant articles.

##### Statements

**Table:** 

Level 4	A safe area for Veress needle insertion usually is in the right or left upper quadrant. However, most surgeons prefer an open access (Hasson) in the left or right subcostal region but modify the insertion site depending on previous surgery and expected adhesions.
	The location of the trocars is influenced by the location of the hernia defect or defects.
	The use of 30° and 45° scopes provides better visualization of the inner aspect of the abdominal wall.

##### Recommendations

**Table:** 

Grade D	The left or right upper quadrant subcostally is recommended for the first access port to the peritoneal cavity.
	The use of a 30° laparoscope is recommended.
	The trocar entry points should be as far as possible from the site of expected adhesions and the size, site, and number of wall defects, and they should be placed to achieve triangulation of the hernia site.

Many articles report on the placements of the trocars [188], which should be placed in dependence on the suspected presence of adhesions and the size, the site, and number of wall defects [187, 189]. A three-trocar technique is mostly preferred, with placement of a 10- or 12-mm trocar first and then, depending on the intraabdominal anatomic situation, placement of one or two additional 5- or 10-mm trocars [190]. These also can be positioned along the subcostal line on the left side crossing the rectus muscle or on the right side [191].

It frequently is necessary to place and manipulate instruments from the side of the patient directly opposite the viewing laparoscope to produce a mirror image that enables better viewing of all the adhesions [187]. Moreover, an opposite 5-mm trocar may provide better fixation for the parts of the mesh near the optic trocar [192]. In a few cases, despite the left subcostal area, a subumbilical insertion may be chosen, but no firm recommendations exist for this decision.

The use of a 30° scope is necessary to provide a good view of the inner aspect of the abdominal wall [187]. In contrast to groin hernia operations, for most patients, the capnopneumoperitoneum is not created using a Veress needle [193, 194]. The left subcostal position is used to insert the first trocar (mostly 10–12 mm) using the open technique (Hasson) and to insufflate CO_2_ to a pressure of 12–14 mmHg [195, 196]. When the mesh is later inserted into the abdomen, the pneumoperitoneum is reduced to 9 mmHg until the mesh is fixed by suture, and then after application of the tacks, the pressure is restored again to 12–14 mmHg [197].

### Port type, positions, and number in laparoscopic ventral hernia repair

#### Sean Rim, Danny Yakoub, George Ferzli

The following search terms were used: “laparoscopic” AND “ventral” AND “incisional” AND “abdominal wall” AND “hernia” AND “technique.” A systematic search of the literature was performed in January 2012 using PubMed, and the Cochrane Library, as well a search of reference lists. A total of 58 articles were found and analyzed, with four articles added. Six articles were used for this review.

##### Statements

**Table:** 

Level 2	Visually guided insertion of trocars can minimize the size of the entry wound but does not decrease the incidence of visceral or vascular injury.
Level 4	Placement of trocars is dictated by the size and location of the defect.
	Placement of additional trocars may be necessary.

##### Recommendations

**Table:** 

Grade B	Visually guided entry of trocars is recommended because these decrease the size of the wound.
Grade D	When additional trocars are needed, the principles of triangulation and maintenance of optimal distance should be taken into consideration.

As with the traditional open approach, the key components of the repair with laparoscopy include tension-free mesh placement, wide coverage of the defect, and meticulous adhesiolysis [106].

##### Port type

Visually guided insertion of trocars does not decrease the incidence of visceral or vascular injury but does decrease the size of the port-site wounds [198].

##### Port positions and number

The fundamental principles of laparoscopic surgery, namely, triangulation around the operative field and optimal distance (16–18 cm) from the target, apply to laparoscopic ventral hernia surgery [199]. The first trocar should always be placed as far as possible laterally from the defect to provide clear visualization of the defect margin. In dealing with midline and right-sided abdominal wall defects, three inline trocars in the left abdomen are ideal. Left-sided abdominal defects are approached via three trocars on the right [199, 200].

Small subxiphoid defects can be managed with the patient in a modified lithotomic position and with the surgeon between the patient’s legs. The camera port is placed at the umbilicus, and a 5-mm trocar on each side provides excellent triangulation around the hernia. For larger subxiphoid defects, the umbilical port is not used. Instead, three trocars are used in the left flank, with the inferiormost port closer to the midline [199, 201]. Suprapubic defects can be managed in a similar fashion.

For smaller defects, the umbilicus can be used as the camera port with two small working ports on either side. Larger suprapubic hernias also can be repaired using three left-flank trocars, with the uppermost port closer to the midline [199, 202]. Additional ports should always be placed as needed. This certainly will be of benefit for difficult cases in which extensive adhesiolysis is required or a large hernia sac is encountered.

### Principles of adhesiolysis

#### M. Rohr, J. Lang

The following search terms were used: “hernia” AND “adhesiolysis” (*n* = 98), “abdominal” AND “adhesiolysis” (*n* = 353), and “abdominal” AND “adhesiolysis” AND “treatment” (*n* = 316). In August 2011, a systemic search of the available literature was performed using Medline, PubMed, the Cochrane Library, as well as a search of relevant journals and reference lists using the aforementioned search terms. A total of 385 papers were found, but only 73 were relevant to the topic.

##### Statements

**Table:** 

Level 1b	Adhesiolysis offers no additional benefit in itself.
Level 3	Adhesiolysis increases the risk of iatrogenic enterotomy, which increases mortality.
Level 4	Greater age and number of previous operations increase the risk of iatrogenic enterotomy during adhesiolysis.
Level 5	Monopolar coagulation has a larger collateral damage zone surrounding the coagulated tissue and produces higher temperatures.
	Currently, there is no reliable prevention of adhesions in abdominal surgery.
	Use of monopolar electrocoaguation increases the risk of enterotomy.

##### Recommendations

**Table:** 

Grade B	Adhesiolysis should be limited to freeing the abdominal wall to enable adequate overlapping of the defect by the mesh.
Grade C	Cold and sharp adhesiolysis is preferred to ultrasonic dissection.
	Bipolar coagulation is allowed, but monopolar coagulation should be avoided.
Grade D	Adhesiolysis should be performed near the abdominal wall away from the adherent bowel.

Although up to 25 % of the individuals have adhesions without previous operations [203, 204], adhesions form after nearly every invasive abdominal procedure [205]. Adhesions are a major health problem [206–215]. In a hernia operation, adhesiolysis is a basic part of the procedure because nearly all hernias exhibit adhesions to the abdominal wall.

An important issue is to decide how much adhesiolysis is needed. For hernia operations, use of a mesh adhesiolysis is needed to free the abdominal wall around the overlapping zone of the mesh. In the FINHYST trial, adhesiolysis was the strongest single risk factor for major complications as a whole [odds ratio (OR), 2.41; 95 % confidence interval (CI) 1.38–4.21] [204]. Most important, an increased extent of adhesiolysis may lead to an increased frequency of enterotomies with life-threatening sequelae [175, 212, 216]. On the other hand, surgical adhesiolysis offers no additional benefit (e.g., less chronic abdominal pain) [217]. Evidence points in the direction of a strategy that favors sufficient adhesiolysis to enable optimal overlapping of the defect by the mesh on all sides. Adhesiolysis should be performed away from the adherent tissue and near the abdominal wall [193].

Adhesiolysis can be performed using several methods, and reformation of adhesions is unaffected by the method of adhesiolysis used [218]. According to existing level 4 evidence, ultrasonic dissection is safe for adhesiolysis [219], and a level 2c study showed fewer gallbladder perforations during laparoscopic cholecystectomy when the harmonic scalpel was used instead of monopolar cautery) [220]. Additionally, the harmonic scalpel has a smaller collateral damage zone and reaches lower temperatures than monopolar cautery [221].

The search showed no study directly comparing different methods of adhesiolysis and their risks, although an Italian consensus conference recommended cold and sharp adhesiolysis [193]. In an animal model, ultrasonic coagulating shears, electrothermal bipolar vessel sealer, titanium laparoscopic clips, and plastic laparoscopic clips all show sufficient hemostasis [222]. Therefore, to avoid enterotomy, it is safer to use cold and sharp adhesiolysis or ultrasonic dissection.

### Laparoscopic ventral or incisional hernia repair: importance of defining hernia defect margins and gauging the size of the hernia pre- and postoperatively

#### P. Chowbey

A systematic search and review of the literature was performed in Pubmed, Medline, the Cochrane Library, EMBASE, the *British Journal of Surgery* database, UK Pubmed Central, Google, Google scholar, Scirus, Ovid, and the Directory of Open Journal Access (DOAJ). The following search terms were used: “hernial defect size,” “hernial defect margins,” “hernial defect diameter,” “hernial defect area,” “laparoscopic contraindications,” “mesh size,” “measuring hernial defect size,” “incisional hernia,” and “ventral hernia.” A total of 28 publications that covered the topic were found, 8 of which were found useful for the review.

##### Statements

**Table:** 

Level 2B	Size of the hernia defect is a significant risk factor for recurrence in laparoscopic ventral/incisional hernia repair.
Level 3	Accurate measurement of the hernia defect size is important to the choice of an appropriate surgical technique.
Level 3	Accurate measurement of the defect is important to the choice of an appropriate-sized mesh.
Level 3	The laparoscopic approach affords the surgeon the ability to define the margins of the hernia defect clearly and definitively and to identify additional defects that may not have been clinically apparent preoperatively.

##### Recommendations

**Table:** 

Grade B	Accurate measurement of the hernia defect size should be performed.
Grade B	The intracorporeal method of measuring the size of the hernia defect should be used.

In open surgery, the size of the defect may play a minor role [223], whereas in laparoscopic repair, its accurate measurement seems to be essential for estimating the proper size of the mesh to be used [96, 224]. Laparoscopic procedure is performed for patients with larger defects (i.e., > 15 cm) [158], but this will not work without sufficient overlapping. The more overlapping the surgeon achieves, the lower the recurrence rate will be [225]. Precise measurement of defect size and the correspondingly choice of an appropriate mesh size are indispensable preconditions for the success of the repair.

To determine the size of the hernia defect, a transverse and vertical dimension of 6 to 10 cm is added, and a prosthesis slightly larger than these measurements is used to ensure at least a 3- to 5-cm overlap [226].

Currently, no standard and accurate method exists for measuring the size of the hernia defect. Most commonly, measurement of the hernia defect size is estimated by physical examination, which lacks accuracy [42]. Other methods include extracorporeal palpation of the hernia defect, with it marked in the distended abdominal cavity and then measured after deflation [226]. Intracorporeal measurement is possible by placing spinal needles through the abdominal wall or by placing an intraperitoneal ruler after adhesiolysis. In addition, the size of the hernia can be reported as the largest diameter of the hernia defect when measured directly intraperitoneally by a laparoscope [42, 226].

Intracorporeal methods are more accurate and advantageous than extracorporeal methods. The laparoscopic approach defines the margins of the hernia defect clearly and helps in identifying additional defects that may not have been apparent preoperatively. In addition, it prevents distortion of abdominal wall contour and the hernia sac [42, 226, 227].

### Bridging, augmentation, and reconstruction of the linea alba: closure of the defect before IPOM

#### J. F. Kukleta, E. Chelala, P. Chowbey

In August 2011 and April 2012, a systematic search of the available literature was performed using Pubmed, Medline, and the Cochrane Library, as well as a search of other relevant journals and reference lists. The following search terms were used: “augmentation repair” AND “incisional hernia” AND “bridging repair” AND “defect closure”; “hybrid repair” AND “linea alba reconstruction” AND “incisional hernia.” The search found 53 articles on defect closure, 9 articles on augmentation repair, 3 articles on bridging repair, 1 article on hybrid repair, 18 articles on linea alba reconstruction, and 21 articles on linea alba and incisional hernia. A total of 27 articles were relevant but were of a low evidence level (levels 3, 4, and 5).

Laparoscopic repair of ventral and incisional hernias was introduced by Karl LeBlanc [228] in 1993. The IPOM procedure consists of reducing the hernia content and patching the abdominal wall defect with an overlapping nonabsorbable synthetic mesh, which is tacked to the abdominal wall. In LeBlanc’s original technique, the tacks were metallic. The experience with the first 100 patients led to reinforcement of the tacked mesh with several additional transfascial mesh-fixing sutures, decreasing the recurrence rate from 9 to 4 % in the next 100 patients [152].

Such a “bridging repair” may lead in larger hernias to a functional problem. The major goal of any open abdominal wall repair is not only reduction of hernia content and prevention of further herniation but also restoration of the integrity and restitution of abdominal wall functionality, especially restoration of the linea alba. Mimicking open repair, laparoscopic operation should combine the transfascial transabdominal closure of the defect with the IPOM placement. Such procedure is called “augmentation repair” (or IPOM-Plus) in contrast to “bridging repair” (the classical IPOM). The laparoscopically assisted transfascial suturing is achieved either transabdominally with multiple interrupted sutures [229, 235, 237] or intraabdominally with a running suture [232].

The bridged area in IPOM is formed by mesh only (with no musculo-aponeurotic coverage) and as such is functionally adynamic. This creates the well-known phenomenon of bulging and leaves space for seroma formation. The sutured repair in IPOM-Plus may reduce the hernia size to zero, eliminating bulging, and decreasing the seroma size and incidence, hence keeping the potential infection risk low.

Although the straight defect closure is not feasible for every hernia due to unacceptable tension, a combination with the endoscopic components separation technique may lower the tension and enable the closure. Hybrid techniques (combination of different approaches) can combine minilaparatomy for hernia closure and a following laparoscopic IPOM reinforcement with or without components separation.

##### Statements

**Table:** 

Level 3	Reconstruction of the linea alba in laparoscopic incisional hernia repair improves the functionality of the abdominal wall.
	Reconstruction of the midline (even using open procedure) and laparoscopic reinforcement through IPOM decrease the rate of wound complications.
	Laparoscopically assisted transfascial repair of the midline defects often is feasible under “physiologic tension.”
	Although not “tension free,” the augmentation repair causes less pain in the early postoperative period than bridging repair.
	Augmentation repair (due to combined defect closure and extended mesh overlap) is a stronger repair than bridging repair if technically feasible. The usual overlap of 5 cm can be extended to 8 cm, for example, without an increase of technical difficulty.
	The IPOM-Plus technique reduces the recurrence rate compared with classical IPOM.
Level 4	Closing hernia defects in IPOM-Plus repair minimizes seroma incidence and prevents bulging, thus reducing the patient’s discomfort.
	The augmentation repair decreases the recurrence rate and the incidence of chronic pain.
	Reconstruction of the linea alba without mesh reinforcement leads to high recurrence rates.

##### Recommendations

**Table:** 

Grade B	The suture material for defect closure in IPOM-Plus should be nonabsorbable.
Grade C	Reconstruction of the linea alba (or any defect closure) in laparoscopic ventral or incisional hernia repair combined with IPOM is recommended for hernias of limited size. Additional components separation facilitates the closure and should be used for larger defects.
Grade D	The anterior transfascial suture technique should involve the hernia sac to obliterate the dead space as much as possible with the aim of preventing seroma formation.

In 2003, Chelala et al. [234] presented his “suturing concept for laparoscopic mesh fixation in ventral and incisional hernias.” An essential component of his technique is closure of the defect with the U reverse stitches. The same author [235, 237] reported improved outcomes with growing experience (733 patients), longer follow-up periods, and experience with 85 redo surgeries [237].

Palanivelu et al. [232] retrospectively analyzed 721 patients with laparoscopic incisional hernia repair. During a mean follow-up period of 4.2 years, only four recurrences (0.55 %) were noted. The repair consisted of defect closure with running suture of polyamide and reinforcement using intraabdominal Parietex composite mesh or Dualmesh.

In 2004, Franklin et al. [143] published a retrospective analysis of 384 patients with laparoscopic abdominal wall hernia repair. During a mean follow-up period of 47.1 months, 11 recurrences (2.9 %) were found. Their repair involved closure of large defects with nonabsorbable interrupted sutures, even if only a limited closure was possible, and reinforcement with nonabsorbable mesh. Banerjee et al. [231] reported on a retrospective comparative study of 193 patients. His IPOM-Plus of interrupted nonabsorbable sutures and intraperitoneal mesh reinforcement achieved better recurrence rates than IPOM used to manage primary and recurrent abdominal wall hernias (3 vs 4.8 and 4.8 vs 10.5 %, respectively).

Agarwal et al. [230] described a defect-suturing technique using spinal needles as threader and snare needles. He introduced the mesh through a 10-mm port placed through the hernia defect, which was consecutively covered by the prosthetic mesh. Sharma et al. [244] proposed interrupted nonabsorbable sutures with far-near-near-far stitching. This results in a double-layered suture repair augmented with intraperitoneal mesh.

In 2011, Orenstein et al. [241] described the shoe-lacing technique for physiologic abdominal wall reconstruction. “Figure eight stitches” with nonabsorbable sutures close the defect. Nonabsorbable cardinal sutures and additional absorbable transfascial sutures around the defect support the circumferential fixation of the mesh margins with metallic or absorbable tacks. A systematic review of the outcomes for correction of diastasis of the recti by Hickey et al. [242] demonstrated that the resuturing without adequate support of mesh and sufficient fixation leads to unsatisfactory results. To enable defect closure in large hernias, some additional operative steps may become necessary (hybrid procedures) [247, 251, 253].

### How much overlap is necessary?

#### Salvador Morales-Conde

A Medline search was performed until November 2011 using the following terms: “laparoscopic repair,” “ventral hernia,” “ventral defect,” “overlapping,” “overlap,” and “mesh size.” The number of papers identified was 78 (following the flow indicated in Fig. 1). The number of papers analyzed was 23, and 55 were excluded because they were unrelated to the topic (*n* = 3), were experimental studies not related to overlap during LVHR (*n* = 2), only analyzed mesh size related to the size of the defect (*n* = 41), described overlap during open repair ((*n* = 4), or did not establish size in centimeters when describing overlap (*n* = 5). Of the 23 papers included in the final analysis, none had an evidence level of 1 or 2, only 2 had an evidence level of 3a, 2 had an evidence level of 3b, 14 had an evidence level of 4, and 5 had an evidence level of 5.

##### Statements

**Table:** 

Level 3	Recurrence is increased if overlap of the fascial defect by the prosthesis is inadequate.
	Large meshes with substantial overlap are associated with a low recurrence rate.
Level 4	Structures such as the falciform ligament, the ligamentum teres, and the prevesical fatty tissue require dissection to enable proper fixation and incorporation of the mesh in the area that has mesh overlap of the fascial defect.
	A larger overlap of the prosthesis (5 vs 3 cm) is necessary if sutures are not used and is more important for securing the overlap than the use of transfascial sutures for fixation of the mesh.
	Recurrence after incisional hernia repair appears to be due primarily to disregard for the principle that the whole incision (not only the hernia) must be repaired.

##### Recommendations

**Table:** 

*Grade B*	The mesh used for laparoscopic repair of a ventral hernia should overlap the hernia defect by at least 3 to 4 cm in all directions
*Grade C*	For proper fixation and incorporation of the mesh dissection of anatomic structures such as the falciform ligament, the ligamentum teres and the prevesical fatty tissue should be done.
	A large overlap of the defect by mesh is necessary, with a minimum of 5 cm if the mesh is fixed without transfascial sutures.
	A larger overlap is recommended for larger hernias than the overlap used for small hernias.
	To avoid recurrences, the entire incisional scar should be covered by the mesh, even if the defect is overlapped 3 to 5 cm in all directions.

Initially, surgeons related recurrences to the method of fixation. In fact, LeBlanc [182] in 2004 established that the main reason for recurrence after LVHR was related to cases in which transfascial sutures were not used. Studies with an evidence level 3a [182, 255] showed that a larger overlap of the defect by the prosthesis (5 vs 3 cm) was necessary if sutures were not used. Findings have shown that technical reasons, together with a small overlap of the defect in all directions, are among the key factors related to recurrences and are more important than the method of fixation.

Tsimoyiannis et al. [225] studied 78 patients who underwent 80 LVHR procedures with expanded polytetrafluoroethylene (ePTFE) dual mesh placed intraperitoneally and fixed by full-thickness sutures and endoscopic tacks. They concluded that the combination of a large patch to overlap the defect by at least 4 cm and the surgeon’s experience were important in the prevention of recurrences. However, it is difficult to draw reliable conclusions based on 14 studies with an evidence level of 4, especially because three of the studies were based on fewer than ten cases [256, 261, 266].

Summarizing the literature, the mesh should overlap the hernia defect at least 3–5 cm in all directions, and the extent of this overlap should be larger as the defect gets larger. However, whereas the evidence for the first recommendation is strong, the evidence for the second recommendation is weak. The need for a large overlap is related to three factors: intraabdominal pressure because this will tend to fold the mesh better against the abdominal if its surface is sufficiently large; a large mesh because it will have more surface to interact with the abdominal wall, increasing the ingrowth and hence the biologic fixation; and a large mesh to compensate for any shrinkage of the mesh. Another important issue relates to the need for covering the entire previous scar to avoid a weak area in the abdominal wall through which a new hernia or a recurrence can occur [44].

### Fixation

#### R. H. Fortelny, M. Misra, F. Köckerling, J. Kukleta

The following search terms were used: “laparoscopic hernia repair” AND “LVHR” AND “incisional hernia” AND “ventral hernia” AND “fixation” AND “sutures” AND “tacks” AND “staples” AND “recurrences” AND “pain” AND “long-term results.” In August 2011, a systemic search of the available literature was performed using Medline, PubMed, and the Cochrane Library, as well as a search of relevant journals and reference lists using the aforementioned search terms. The first search found 64 relevant articles. In a second-level search, 14 articles were added. Hence, 78 publications were used for this review.

##### Statements

**Table:** 

Level 1B	The method used for mesh fixation (sutures and/or tacks) has no influence on acute postoperative pain.
	Suture fixation of the mesh incurs a significantly longer operation time than fixation by tacks.
	The absorbability of the suture material used for mesh fixation is not related to the incidence of postoperative pain.
	Tacks-only fixation is associated with a significantly higher grade of mesh shrinkage in the horizontal direction than transfascial suture fixation.
	In umbilical hernias with a defect size up to 5 cm, mesh fixation by glue results in less acute postoperative pain than fixation by tacks.
Level 3	The incidence of acute postoperative pain correlates significantly with the number of tacks used for mesh fixation.
Level 4	The recurrence rates do not differ between the different fixation techniques.
	Application intervals of 1.5 cm for the staples/tacks in the single- or double-crown technique are associated with a low recurrence rate.
	The type of mesh fixation technique does not influence the incidence of postoperative chronic pain.
	The use of resorbable penetrating fixation devices achieves sufficient tensile strength and low recurrence rates.
	The use of additional glue fixation increases the efficacy of fixation and postoperative pain.
Level 5	Penetrating fixation devices (e.g., transfascial sutures, protruding tacks) can cause incisional hernias and in the pericardial region may result in a cardiac tamponade.

##### Recommendations

**Table:** 

Grade B	Suture fixation alone or a combination with tacks should be performed.
Grade C	The tacks-only fixation can be considered the technique of choice, taking into account the increased risk of postoperative pain due to the number of devices and the need for an additional overlap of mesh (at least 5 cm) to prevent recurrence caused by shrinkage.
	Additional glue fixation reduces the need for penetrating fixation devices and hence decreases postoperative pain and device-induced hernia.

Since the introduction of laparoscopic surgery for ventral and incisional hernia (LVHR) by LeBlanc and Booth [228] in 1993, one of the most controversially discussed topics relates to the technique used for mesh fixation. The majority of reports describe the use of transfascial sutures and tacks fixation (e.g., Heniford et al. [6] and LeBlanc et al. [64]), achieving low recurrence rates of 4.7 and 4 %, respectively. LeBlanc et al. [152] demonstrated that additional transfascial suture fixation and an increased mesh overlap reduces the recurrence rate from 9 to 4 %.

On the other hand, several studies have testified to the efficacy of tacks-only fixation. Frantzides et al. [281] and Carbajo et al. [159] reported very low recurrence rates of 1.4 and 4.4 %, respectively, with this technique. The discussion concerning the increased recurrence risk due to fewer fixation devices (e.g., transfascial sutures) still is going on [326]. Finally, new absorbable fixation devices such as tacks, staples, and glues have been developed to reduce the risk of chronic postoperative pain.

##### Recurrences related to fixation techniques

The usually performed fixation techniques (transfascial sutures with tacks, sutures only, and tacks only) have been compared. For this review we used a modification of the recommendation by Kapischke et al. [116] and included only studies with a minimum of 100 patients and a follow-up period of at least of 24 months. A total of 23 studies were selected and grouped by procedures as follows: transfascial sutures and tacks (10 studies) [42, 44, 143, 152, 165, 265, 273, 274, 275, 276], sutures only (2 studies) [232, 277], and tacks only (11 studies) [42, 44, 159, 278–285].

The median recurrence rate for all three groups comprising 5,884 patients in the 23 publications was 3.95 % (2–5, 6) during a cumulative follow-up period of 35.5 months [29–48]. The recurrence rates for the three groups were as follows: 3.65 % (range 2.45–5.75 %) for the suture and tacks fixation group comprising 2,211 patients, 1.05 % (range 0.82–1.27 %) for the sutures only fixation comprising 1,121 patients, and 4.5 % (range 2.4–6.17 %) for the tacks only fixation group comprising 3,473 patients (Table 3). The three groups did not differ significantly in terms of recurrence rates or follow-up periods (by Kruskal–Wallis and ANOVA tests) (Table 3).

**Table 3:** Recurrence rates and chronic pain in dependence on the type of fixation (systematic review of the literature)

Type of fixation	No. of studies	Total no. of patients	Recurrence rate median % (IQR)	Chronic pain follow-up median %/month (IQR)
Sutures+tacks	10	2,211	3.65 (2.45–5.75)^a,b^	2.75 (1.72–13.22)^a,b^
				31.5 (27.75–38.25)
Sutures only	2	1,121	1.05 (0.82–1.27)^a,b^	3.75 (3.12–4.37)^a,b^
				39 (33.5–44.5)
Tacks only	11	2,473	4.5 (2.4–6.17)^a,b^	6,35 (2.17–13.22)^a,b^
				40 (30.5–49.5)

*IQR* interquartile range

^a^ *p* = 0.17 (Kruskal–Wallis test)

^b^ *p* = 0.535 (ANOVA)

The two studies [232, 277] using suture only repair based on the principle of suture closure of the defect and mesh reinforcement of the abdominal wall in contrast to the usual IPOM technique obtained the lowest recurrence rate (1.05 %) but failed to show a statistically significant difference compared with the other groups. Due to the variability of patient characteristics and the nonstandardized technique of using different fixations and mesh types, these results are likely to contain bias and need confirmation by RCTs (currently lacking).

##### Acute postoperative pain

The incidence of acute postoperative pain was analyzed from the data of four RCT 1B studies [181, 254, 286, 287] and one prospective 2B study [288] (Table 4). In the study of Wassenaar et al. [286] 172 patients were included and randomized into three procedure groups: absorbable sutures with tacks (*n* = 56), tacks in double-crown technique (*n* = 60), and nonabsorbable sutures with tacks (*n* = 56). No significant differences among the different fixation techniques in terms of pain were detected at any time point.

**Table 4:** Comparison of the incidence of acute postoperative pain in relation to different types of fixation in RCT’s

Authors	Study	Total patients (groups)	Type of fixation	Assessment (weeks or days^a^)	Acute pain Sut/FS/tack	*p* Value	Level of evidence
Wassenaar et al. [286]	RCT	172 (56/60/56)	SR+T vs T vs SN+T	2/6/18	NS/NS/NS	>0.05	1b
Bansal et al. [181]	RCT	68 (32/36)	SN vs T	1^a^/1/12	S/S/S	<0.05	1b
Beldi et al. [254]	RCT	40 (20/20)	SN vs T	6/24	S/NS	0.020	1b
Eriksen et al. [287]	RCT	38 (19/19)	FS vs T	2^a^/10^a^	S/S	0.025	1b
Nguyen et al. [288]	Prospective comparative.	50 (29/21)	SN vs T	1/4/8	NS/NS/NS	>0.05	2b

*Sut* suture, *FS* fibrin sealant, *RCT* randomized controlled trial, *SR* resorbable suture, *T* tacks, *SN* nonresorbable suture, *NS* nonsignificant, *S* significant

The study of Bansal et al. [181] enrolled 68 patients and randomized them into two procedure groups: tacks (*n* = 36) and nonabsorbable sutures (*n* = 32). Tacks fixation resulted in significantly higher pain scores than suture fixation at 1.6 and 24 h and also at 1 week and 3 months postoperatively.

Beldi et al. [254] randomized 40 patients into two procedure groups: nonabsorbable sutures (*n* = 20) and tacks (*n* = 20). The transfascial suture group experienced significantly higher pain scores than the tacks group at 6 weeks, but these became nonsignificant at 6 months.

Nguyen et al. [288] reported on another RCT. In their study, pain assessment was studied in two procedure groups: sutures (*n* = 29) and tacks (*n* = 21). The two groups showed no significant difference at 1 week, 1 month, and 2 months postoperatively.

Eriksen et al. [287] reported on 40 patients with an umbilical hernia defect (1.5–5 cm) at three Danish hernia centers. The patients were assigned randomly (20/20) to either fibrin sealant fixation (4 U/ml thrombin) or titanium tacks fixation (double crown). The assessment of acute pain (postoperative days 0–2) by visual analog score (VAS 0–10) showed significantly less pain in the fibrin sealant group than in the tacks group at rest (median 19 vs 47 mm; *p* = 0.025) and during activity (38 vs 60 mm; *p* = 0.014).

Bansal et al. [181] attributed the reduced pain in the suture group to the technique of “loose tying of the sutures.” Despite the significant difference compared with the tacks group, the pain scores in both groups were very low: 2.5/1.6 at 1 week, 1.5/0.6 at 1 month, and 0.6/0.14 at 3 months.

##### Chronic postoperative pain

Chronic pain is defined as pain lasting at least 3 months postoperatively. To find any possible correlation between different fixation techniques and the incidence of chronic postoperative pain, the three different groups (transfascial sutures and tacks [42, 44, 143, 152, 165, 265, 273–276], sutures only [232, 277] tacks only [42, 44, 159, 278–285]) were analyzed. The median incidences of chronic pain in the suture and tack fixation group were 2.75, 3.75, and 6.35 % respectively (nonsignificant difference) (Table 4).

##### Number of tacks and postoperative pain

A comparative study correlating postoperative pain and number of tacks used for mesh fixation was reported by Schoenmaeckers et al. [289]. The assessment of pain by VAS showed significantly less pain (*p* = 0.001) 3 months postoperatively in the group with 55 % fewer tacks used for fixation, but this difference became nonsignificant at 6 months.

##### Intervals of tacker fixation

Nine studies have analyzed the correlation between intervals of tacks fixation and recurrence (Franzidis et al. [281], Baccari et al. [260], Carbajo et al. [159], Ceccarelli et al. [290], Ferrari et al. [158], Morales et al. [283], Olmi et al. [285], Sharma et al. [42], Wassenaar et al. [44] (see Table in electronic version). Mean tacks fixation intervals of 1.5 cm (range 1–2) correlated with a recurrence rate of 2.85 % (range 2.1–3.8 %) during a follow-up period of 37 months (range 29–40 months). The analysis of the different mesh overlaps showed a mean of 4 cm (range 3.1–4.5 cm).

##### Operation time: suture fixation versus tacks fixation

The correlation between type and time of fixation was investigated in studies by Wassenaar et al. [286], Bansal et al. [181] and Nguyen et al. [288]. The operation time in the suture group was significantly longer in the RCTs of Wassenaar et al. [286] (50.6 vs 41.1 min; *p* = 0.002) and Bansal et al. [181] (77.5 vs 52.6 min; *p* < 0.0001). However, the prospective study reported by Nguyen et al. [288] showed no significant difference between the two groups.

##### Type of suture used for fixation and pain

Only one study, an RCT, reported by Wassenaar et al. [286] investigated the influence that type of suture material used for transfascial suture fixation had on postoperative pain by comparing absorbable (Vicryl) and nonabsorbable (Mersilene) sutures. Their study showed no significant difference in postoperative pain assessed by VAS 2 weeks, 6 weeks, and 3 months after surgery.

In another randomized study by Bellows et al. [183], patients were randomized to receive local infiltration anesthesia (0.25 % bupivacaine with epinephrine) in all layers of the abdominal wall to the level of the parietal peritoneum at suture fixation sites (nonabsorbable Gore-Tex sutures) immediately before suture placement compared with a control group that received no local anesthesia. The treated group experienced a statistically significant decrease in postoperative pain scores (VAS, 0–10) 1 h postoperatively (2.2 vs 6.4; *p* < 0.05). At the other time points (4 and 24 h), the mean pain scores, although lower in the treated group, were not significantly different.

##### Fixation-associated complications

*Mesh shrinkage*. The RCT reported by Beldi et al. [254] investigated tacks (Protack; single-crown technique, 2-cm intervals) versus suture (polypropylene, 2- to 3-cm intervals) fixation of a composite polyester mesh with an overlap of at least 5 cm using conventional abdominal x-ray examination with the patient in prone position on postoperative day 2 and then after 6 weeks and 6 months. In the tacks fixation group, a significant decrease in mesh size was detected in the horizontal direction, whereas no significant differences were found in the vertical direction or the mesh surface area.

In another study by Schoenmaeckers et al. [292], mesh shrinkage after double-crown fixation of ePTFE meshes was investigated by CT measurements. A shrinkage rate of 7.5 % was found at 17.9 months postoperatively.

*Incisional hernia*. Several case reports on fixation device-induced incisional hernias have been published. The first report in 2003 published by LeBlanc [293] concerned the development of an incisional hernia at the site of a penetrating tack, described as a “tack hernia.” Further reports by Muysoms et al. [294], Khandelwal et al. [296] and Barzana et al. [297] describe incisional hernias after suture fixation. The most severe complication of tacks fixation, reported by Malmstroem et al. [295], consisted of a fatal cardiac tamponade.

##### New fixation devices

*Resorbable fixation devices*. Although resorbable devices for mesh fixation in LVHR have been available for some years, only one prospective multicenter clinical trial study by Lepere et al. [298] investigating these devices has been published. In this study, 29 patients in 11 centers were treated for incisional and umbilical hernia by LVHR. The mesh fixation was performed by I-Clip (10-mm disposable instrument), which is resorbable within 1 year. Pain assessment by VAS (0–10) at 1 and 12 months showed no pain at any time points. The recurrence rate during a follow-up period of 1 year was 0 %. Meanwhile, the I-Clip device (Ethicon Endo-Surgery, Inc., Somerville, NJ) was replaced by new resorbable tacks devices, achieving higher tensile strength, as reported by Hollinsky et al. [299]. New absorbable fixation devices (e.g., SorbaFix (C. R. Bard, Inc., Murray Hill, NJ), PermaFix (Davol Inc. Bard, Murray Hill, NJ), AbsorbaTack (Covidien, Mansfield, MA), Securestrap (Ethicon Endo-Surgery, Inc., Somerville, NJ) have been developed that achieve a sufficient tensile fixation strength compared with conventional nonresorbable tacks (Protack) and transfascial suture repair [299, 300], but randomized trials are required to verify these experimental results.

##### Glue fixation

*Clinical studies*. The first clinical report published by Olmi et al. [301] described a prospective study of 40 patients with a defect 2–7 cm in diameter using diluted Tissucol (One Baxter Parkway Deerfield, IL) (50 U/ml thrombin) by Duplotip application (One Baxter Parkway Deerfield, IL) and temporary suture fixation. During a median follow-up period of 16 months, no hematoma, seroma, or recurrence was detected. The pain score (VAS) after 7 days postoperatively was 0 for all the patients.

Another case–control study by Olmi et al. [302] included 19 patients with a defects smaller than 6 cm in diameter. Again, mesh fixation was performed by diluted Tissucol applied by Duplotip. In two cases, transfascial suture fixation was added. No complications or recurrences were detected during a mean follow-up period of 20 months. The pain score (VAS 0–10) was 1 at 7, 15 and 30 days postoperatively.

Recently, Erikson et al. [287] published a multicenter RCT that included 40 patients with an umbilical hernia defect 1.5–5 cm in size. The patients were assigned randomly to fibrin sealant (4 U/ml thrombin) or titanium tacks fixation (double-crown technique). The fibrin sealant group had significantly less pain (VAS 0–100 mm) on postoperative days 0–2, resumed normal daily activity earlier (after a median of 7 vs 18 days; *p* = 0.027), and reported significantly less discomfort.

In conclusion, mesh fixation in LVHR by fibrin sealant for small umbilical hernias (≤5 cm) was associated with less acute postoperative pain, less discomfort, and a shorter convalescence than tacks fixation in the very short follow-up period of 10 days. The results reported by Olmi et al. [301, 302] confirm the feasibility of glue fixation for small ventral hernias with a defect size up to 7 cm during follow-up periods of 16 and 20 months, respectively, without any recurrences. These clinical results are very promising but need confirmation by larger prospective studies with longer follow-up periods.

### Fixation in suprapubic and subxiphoidal hernia repair

#### R. H. Fortelny, M. Misra, F. Köckerling

The following search terms were used: “laparoscopic hernia repair” AND “LVHR” AND “incisional hernia” AND “suprapubic hernia” AND “parapubic hernia” AND “subxiphoidal hernia” AND “fixation” AND “tacks” AND “staples” AND “recurrences” AND “pain” AND “long term results.” In August 2011, a systematic search of the available literature was performed using Medline, PubMed, and the Cochrane Library, as well as a search of relevant journals and reference lists. The first search yielded 19 relevant articles, and the second-level search yielded 2 articles. Hence, the review was based on 21 articles.

The specification of the term “suprapubic hernia” is defined by Carbonell et al. [202] and Palanivelu et al. [332] as a hernia defect located 3–4 cm above the symphysis pubis and by the EHS classification [70] as hernia M5. The most common cause of suprapubic hernia is a postoperative incisional hernia (e.g., after suprapubic radical prostatectomy [338]). Congenital malformations of the pelvis are very rare [337].

##### Statements

**Table:** 

Level 4	A retropubic dissection is necessary to achieve sufficient and safe mesh overlap of the suprapubic defect as well as an effective fixation.
	A combination of mesh fixation by sutures and tacks, including fixation at Cooper’s ligament and a sufficient mesh overlap, is associated with a low recurrence rate.

##### Recommendations

**Table:** 

Grade C	For safe positioning and sufficient overlap of mesh, the retropubic space should be dissected.
	The mesh fixation should include Cooper’s ligament, preferably by penetrating devices.

The first report describing a mesh-enforced repair of an incisional parapubic hernia by the open approach was published by Bendavid [328] in 1990.

##### Fixation in suprapubic hernia

In 1999, Matuszewski et al. [329] reported the first laparoscopic repair of an incisional suprapubic hernia after suprapubic radical prostatectomy using a polypropylene mesh and fixation by clips. By August 2011, other publications included reports of three case series [202, 330, 331] and four retrospective studies [15, 332–334] on laparoscopic repair of suprapubic hernia. Hirasa et al. [330] treated suprapubic hernias laparoscopically in seven patients without dissection of the space of Retzius using dual-surface mesh with an overlap of 2–3 cm and fixation by tacks. They reported one recurrence during a mean follow-up period of 5.8 months. All other studies have described the need for a complete dissection of the retropubic space for appropriate mesh positioning with good overlap of at least 4–5 cm.

Most fixation techniques are based on a combination of sutures and tacks. A new technique described by Palanivelu et al. [332] involves complete closure of the hernia defect by running sutures and mesh fixation (overlap of 5 cm) using pre-tied intracorporal sutures with 4- to 5-cm intervals circumferentially. Postoperative pain was reported by Carbonell et al. [202], Palanivelu et al. [332], Varnell et al. [15], and Sharma et al. [334], possibly due to tight transfascial sutures, but of varying incidences, from 2.7 to 9.7 %. The largest series (72 patients) was reported retrospectively by Sharma et al. [334]. In this series a combination of devices (transfascial sutures and tacks) for mesh fixation with an overlap of 5 cm was used. During the longest follow-up period of all the studies (4.9 years), a recurrence rate of 0 % and a postoperative pain incidence of 9.7 % occurred. Carbonell et al. [335] reported a novel method of mesh fixation using a bone anchor for fixation to the pubic bone in suprapubic hernia repair [336]. The median recurrence rate for a total of 215 patients was 5.5 % (range 2.7–6.0 %) during a follow-up period of 21.1 months. The median incidence of postoperative pain was 4.9 % (range 3.8–6.6 %; level of evidence, 4).

##### Fixation in subxiphoid hernia

Subxiphoid hernia is defined by the EHS classification [70] as hernia M1. Its reported incidence after median sternotomy varies between 1 and 4.2 % [66]. Different types of open repair techniques (onlay mesh, sublay) are described [66], and laparoscopic repair was first reported in 2000 [343].

In the technical repair of subxyphoidal hernia, Conze et al. [54] stressed the importance of the appropriate landmarks for dissection of the retroxiphoidal space. Starting from the dorsal aspect of the xiphoid process, fatty tissue is mobilized by blunt dissection followed by detachment of the diaphragm’s sternal portion and finally separation of the pericardium from the sternum. This technique is mandatory, independent of the approach (open or laparoscopic), to achieve adequate opening of the retroxiphoidal space for safe and effective mesh positioning with sufficient overlap.

##### Statements

**Table:** 

Level 4	Dissection of the extended retroxiphoidal space up to 5 cm behind the xiphoid process is mandatory for appropriate mesh positioning and overlap.
	Fixation in the cephalad portion of the mesh carries a high risk of injury to the pericardium.

##### Recommendations

**Table:** 

Grade C	The overlap of the mesh should be sufficient, especially in the proximal retroxiphoidal space. The proximal part of the mesh should not be fixed.

Only four studies (1 retrospective comparative [341] and 3 retrospective [201, 333, 342]) deal with this topic. In 2000, Muscarella et al. [343] published the first report describing laparoscopic repair of a subxiphoidal hernia using a bilayer permanent composite mesh and four transmural corner stitches and tacks for fixation to the posterior rectus sheath. The first case series of Landau et al. [201] included 10 patients repaired laparoscopically. For mesh fixation, three pre-tied stay sutures and tacks were used. In this series, one patient experienced a recurrence at 20 to 24 months.

In a retrospective comparative study, Mackey et al. [341] reported on the risk of incisional hernia after median sternotomy for cardiothoracic procedures. The cohort of 45 patients who experienced hernia were treated by the open approach (*n* = 35) with suture repair (*n* = 14), mesh repair (*n* = 21), and laparoscopic repair (*n* = 10). During a mean follow-up period of 48 months, three patients experienced recurrence, after a sternal wound in one patient.

In a case study published by Eisenberg et al. [342] four patients (3 with recurrence after open repair) were included. A mesh repair with overlap of 3 cm fixed by six to eight sutures and tacks (omitting fixation of the proximal part) gave good results with no recurrence at 6 months.

In another retrospective study by Ferrari et al. [333] 15 patients (3 with recurrent hernia) underwent repair using mesh fixation performed with only intracorporal sutures to the peritoneal layer or xiphoidal periostium and omitting fixation of the cephalad part of the mesh. The recurrence rate was 6.6 % (1 patient) during a follow-up period of 37 months. Analysis of all 39 patients in the entire cohort showed a median recurrence rate of 8.3 % (range 4.95–15 %) during a follow-up period of 29.5 months (range 18–39.75 months).

### Mesh insertion

#### M. C. Misra, V. K. Bansal, Pradeep Prakash, D. Babu, P. Singhal, R. Fortelny

A systematic search was performed in Pubmed, the Cochrane Library, and Medline, as well as a search of relevant journals and reference lists in the English language. The following search terms were used: “mesh introduction/insertion” AND “laparoscopic” AND “incisional hernia” AND “ventral hernia repair.” Whereas 86 studies (levels 3, 4, and 5) described the technique of mesh insertion, only 12 concerned mesh insertion techniques. In 76 studies (>6,000 patients), mesh was inserted through 10- and 12-mm ports.

Theodoropoulou et al. [344] described mesh insertion through the 10-mm balloon port or balloon port site. Hussain et al. [345] used a separate 10- to 15-mm port for mesh insertion at the center of the hernia after reduction of the contents. Perry et al. [346] used a 2- to 3-cm incision over the hernia site for cases with an incarcerated omentum that could not be reduced safely. An appropriate-sized piece of prosthetic mesh was prepared and inserted into the abdomen via the opened hernia sac. Perrone et al. [153], Nimeri et al. [347] and Agrawal et al. [230] also used a similar skin incision over the defect for mesh insertion.

Carlson et al. [348] described a technique for introducing a large mesh with stay sutures slid into a plastic sleeve and through the 10-mm trocar site, avoiding contact of the mesh with the skin. The mesh itself should be treated in the same fashion as a vascular graft in that any contact with the skin should be avoided [230, 348, 349]. To ensure this, the mesh could be inserted inside a plastic sleeve [348]. Leiberman et al. [350] rolled the mesh along its long axis and after every one-third roll placed a 4-0 chromic catgut suture. The mesh then was inserted through a 10-mm trocar site or 10-mm port site if the mesh was too large.

##### Rolling techniques and mesh introduction

Walter et al. [351] compared four specified insertion techniques. They documented the optimal insertion technique and the minimum port sizes realistically needed for insertion of different types and sizes of mesh. They noted that the roll-and-bind technique allows optimal maximum mesh width (cm) to a minimum port size (mm) ratio (M:P ratio) to be obtained from biologic meshes because it overcomes their tendency to lose their roll. No advantage in using the roll-and-bind insertion technique was found.

##### Statements

**Table:** 

Level 3	Mesh insertion (up to 30 × 30 cm) through a 10- to 12-mm port is possible in the majority of laparoscopic incisional/ventral hernia repairs of varying sizes.
	Mesh insertion through a 2- to 3-cm skin incision at the center of the defect directly (inside a plastic sleeve) or through a 15-mm port may be a viable alternative for larger defects requiring larger meshes (>30 cm).
Level 5	Mesh–skin contact can contaminate the mesh with bacteria.
	The largest lightweight mesh can be inserted safely through a 10- to 12-mm port.

##### Recommendations

**Table:** 

Grade B	Large meshes should be rolled up tightly for safe and effective insertion.
Grade C	For very large meshes (35 × 30 cm), a 15-mm port may be used.
	Mesh–skin contact should be avoided.

## Electronic supplementary material

Below is the link to the electronic supplementary material.

Supplementary material 1 (DOCX 1095 kb)

Supplementary material 2 (DOCX 87 kb)

