# Supplementary material for: Guidelines for laparoscopic treatment of ventral and incisional abdominal wall hernias (International Endohernia Society (IEHS)—Part 1
Source: Surg Endosc. 2013 Oct 11;28(1):2–29. doi: 10.1007/s00464-013-3170-6 (PMC3872300; doi:10.1007/s00464-013-3170-6)
Supplement: Supplementary file 1 — Supplementary material 1 (DOCX 1095 kb) [file 464_2013_3170_MOESM1_ESM.docx]

**Guidelines for laparoscopic treatment of ventral and incisional abdominal wall hernias (International Endohernia Society (IEHS)).**

R. Bittner, D. Berger, J. Bingener-Casey, E. Chelala, U. Dietz, M. Fabian, G. Ferzli, R. Fortelny, U. Klinge, F. Köckerling, J. Kukleta, K. LeBlanc, D. Lomanto, M. Misra, S. Morales-Conde, F. Muysoms, B. Ramshaw, W. Reinpold, S. Rim, M. Rohr, R. Schrittwieser, Th. Simon, B. Stechemesser, D. Weyhe, P. Chowbey.

R. Bittner

Hernia Center Rottenburg am Neckar, Winghofer Medicum, Röntgenstr.38, 72108 Rottenburg. Germany

e-mail: [bittnerfamilie@web.de](mailto:bittnerfamilie@web.de)

D. Berger

Klinik für Viszeral-, Gefäß- und Kinderchirurgie Klinikum Mittelbaden gGmbH, Stadtklinik Baden-Baden, Balger Straße 50, 76532 Baden-Baden, Germany.

J. Bingener-Casey

Division of Gastroenterologic and General Surgery, Mayo Clinic, 200 First Street SW, Rochester, MN  55905. USA.

E. Chelala

Digestive surgery at CHU of Tivoli, Avenue des Cavaliers, 9

1640 Rhode St Genèse, Belgium.

U. Dietz

Department of General, Visceral, Vascular and Pediatric Surgery (Department of Surgery I), University Hospital of Wuerzburg, Oberduerrbacher Strasse 6, 97080 Wuerzburg . Germany.

G. S. Ferzli, M. Timoney, S. Rim

Department of Surgery, Lutheran Medical Center, SUNY Health Science Center, Brooklyn, 65 Cromwell Avenue, Staten Island, NY, USA

R. H. Fortelny

Department of General, Visceral and Oncological Surgery, Wilhelminenspital, 1171 Vienna, Austria

U. Klinge

Surgical Department, University of Aachen, and Institut for Applied Medical Engineering AME Helmholtz, Pauwelstrasse, 52074 Aachen, Germany

F. Köckerling

Department of Surgery and Center for Minimally Invasive Surgery, Vivantes Hospital, Neue Bergstr. 6, 13585 Berlin,

Germany

J. Kukleta

General-, Visceral-, Abdominal Wall Surgery, Klinik Im Park, Grossmuensterplatz 9, 8001 Zürich, Switzerland

K. LeBlanc

Minimally Invasive Surgery Institute and the Fellowship Program,

Baton Rouge, Managing Partner, Surgeons Group of Baton Rouge of Our Lady of the

Lake Physician Group, Baton Rouge, LA, USA

D. Lomanto

Minimally Invasive Surgical Center, KTP Advanced Surgical Training Center, YYL School of Medicine, National University Hospital, Kent Ridge Wing 2, 5 Lower Kent Ridge Road, Singapore 119074, Singapore

M. C. Misra, V.K. Bansal

Division of Minimally Invasive Surgery, J P N Apex Trauma Centre, All India Institute of Medical Sciences, Angari Nagar, New Delhi 110029, India

S. Morales-Conde

Unit of Innovation in Minimally Invasive Surgery. University Hospital ‘‘Virgen del Rocı´o’’, Sevilla, Spain

F. Muysoms

Head of the Department for General and Abdominal Surgery, AZ Maria Middelares, Kortrijksesteenweg 1026, 9000 Ghent – Belgium

B. Ramshaw

Department of General Surgery, Halifax Health, Daytona Beach, Florida, USA

W. Reinpold

Department of Surgery, Gross-Sand Hospital Hamburg, Gross-Sand 3, 21107 Hamburg, Germany

M. Rohr

Chief of the Department of General Surgery , Katutura State Hospital, PO Box 81233 Olympia, Windhoek – Namibia

R. Schrittwieser

Head of the Department of Surgery, LKH, Muerzzuschlag, Tragösserstrasse 1 und 1a,8600 Bruck/Mur, Austria.

Th. Simon

Department of Surgery, GRN‐Klinik Sinsheim, Weinheim, Germany.

B. Stechemesser

Hernienzentrum Köln, Zeppelinstr.1, 50667 Köln, Germany.

D. Weyhe

Department of Surgery, Pius Hospital, Georgstrasse 12, 26121 Oldenburg, Germany.

P. Chowbey

Minimal Access, Metabolic, and Bariatric Surgery, Max Healthcare Institute Ltd., 2 Press Enclave Road, Saket, New Delhi, India.

**Table of contents:**

**Introduction**

**Section 1. Basics:**

How comparable are incisional and ventral hernias in terms of operative technique and outcomes?

Is the routine application of CT and MRI recommended for the diagnosis of ventral/incisional hernias prior to laparoscopic ventral hernia repair?

Classification

**Section 2. Indication for surgery:**

Indications for treatment in dependence on size of defect or hernia sac, hernia type, symptoms, age.

Is there still any place for open suture repair in dependence on defect size?

Limitations of laparoscopic intraperitoneal onlay mesh repair in terms of defect size or body habitus.

Obese patient and ventral/incisional hernia.

Recurrence after open surgery: Re-do better laparoscopically?

**Section 3: Perioperative Management:**

What is the evidence for antibiotic and thromboembolic prophylaxis in laparoscopic ventral/incisional hernia surgery?

**Section 4: Key-points of technique:**

Positioning of the trocars and creating the capnopneumoperitoneum.

Port type, positions, and number in laparoscopic ventral/incisional hernia repair.

Principles of adhesiolysis.

Importance of defining hernial defect margins and gauging size of the hernia preoperatively and intraoperatively

Bridging or augmentation? Reconstruction of the linea alba – yes or no? Is it necessary to close the defect before IPOM?

How much overlap is necessary?

Fixation - best type of fixation? Are permanent sutures needed?

Suture vs. Tacker – what is better?

Fixation in suprapubic and subxiphoidal hernias**.**

Mesh insertion.

**Section 5: Complications:**

Management of bowel injury during laparoscopic ventral /incisional hernia repair.

Unrecognized enterotomy.

Risk factors for infection in laparoscopic incisional / ventral hernia repair.

Mesh infection

Postoperative Seroma: Risk factors, prevention and best treatment.

Postoperative bulging.

Chronic Pain – Risk Factors, Prevention and Treatment

Recurrence after laparoscopic ventral/incisional hernia repair- risk factors, mechanism and prevention.

**Section 6: Technique – special questions:**

Is laparoscopic preperitoneal ventral and incisional hernia repair possible?

The role of Endoscopic Component Separation (ECS) in the treatment of large abdominal wall hernias.

Laparoscopic parastomal hernia repair.

**Section 7: Comparison open vs. laparoscopic repair:**

Comparison of open vs. laparoscopic hernia repair: OR time, bowel lesion, seroma and wound infection.

Comparison of hospital stay, return to activity, cost, quality of life, pain and recurrence after laparoscopic and open ventral and incisional hernia repair.

**Section 8: Mesh technology:**

Do we have an ideal mesh in terms of prevention of adhesions? Are coated meshes really necessary? Are there data to support the manufacturers’ claims of superiority? Is a permanent or absorbable barrier preferred?

Role of biological meshes in laparoscopic incisional and ventral hernia repair? Are they advantageous in infected abdominal wall?

What happens to synthetic mesh after it is inserted into the body?

**Section 9: Hernia prophylaxis:**

Open abdominal surgery and stoma surgery. Indications for prophylactic mesh implantation and risk reduction strategies

**Section 10: New technologic developments:**

From Robotic Surgery to NOTES and Single Port Surgery: Is there any role today in ventral /incisional hernia repair?

**Section 11: Lumbar and other unusual hernias:**

Lumbar and unusual Hernias

**Section 12: Education:**

Education and training in laparoscopic ventral/incisional hernia repair

**Introduction**

Guidelines are increasingly determining the decision –making process in day-to-day clinical work. This role of guidelines remains not, however, undisputed. Critics fear a possible restriction of a doctor's freedom to continue to use diagnostic and therapeutic procedures which had been learned and in personal experience have shown beneficial. Fundamentally guidelines should not restrict medical therapeutic freedom. But guidelines describe the current, best possible standard in diagnostics and therapy. Divergence from them may be to the disadvantage of patients. Such divergence has to be explained. These days the statement frequently heard in former times “This has always worked well in my experience” is no longer automatically accepted. Personal solutions for procedures should be justified and documented. If the personal impression tallies with the objective results a surgeon will have no further problems even in legal cases.

A guideline reflects the current status of scientific research and clinical practice concerning the therapy for a disease. If at all possible a guideline should be developed by an international panel of experts, whereby alongside individual experience above all the results of comparative studies are decisive. According to the results of studies statements and recommendations are formulated and these are graded strictly following the criteria of Evidence Based Medicine (EBM). The value of a recommendation for decision -making in daily clinical work can be seen in the grading in transparent form (see below). This means that with the grading the level of evidence is determined and a diagnostic or therapeutic measure will be carried out corresponding to “must” (grade A), “should” (grade B) or “can” (grade C). A guideline can therefore be valuable in helping, in particular, the young surgeon in his or her work to find the best diagnostic or therapeutic option for the patient when confronted with an increasingly huge and confusing array of measures. But the older surgeon also benefits. Every guideline has to be updated every three years so that the latest insights can be incorporated. This means that it offers a useful orientation aid to the experienced surgeon, too, who, as a rule, has an extremely heavy workload and for whom it is generally difficult to keep up with the increasing flood of publications.

Incisional and ventral abdominal wall hernias are common. Their operative therapy forms a part of the daily routine of every surgeon in general and visceral surgery. In Germany alone 50 000 of these operations are carried out every year. Although the operation for abdominal wall hernia is comparatively unspectacular it can still be invasive in a major way for the individual patient bringing with it a long and painful period of illness and even leading in some cases to a lethal outcome. Findings and operation procedures can be extremely complex – as for instance in the size of defect or hernia sac, extent of intraabdominal adhesions, required operative competence, length of the operation and costs for the materials needed.

Guidelines for the operative removal of an inflamed appendix, of the gall bladder or of bowel in cases of sigmoid diverticulitis are redundant as these procedures are comparatively straightforward ablative interventions. The operation for an abdominal wall hernia is, however, plastic reconstructive as a rule and has become considerably more complex through the introduction and further development of laparoscopic techniques and of biocompatible materials.

For a surgeon who has not been trained in this specific area for, it is increasingly difficult to find the best treatment pathway for the patients. A guideline can be the solution to this problem. The fundamental precondition for a reliable guideline is, however, the availability of studies of high ranking in the classification of the EBM. At the beginning of the presented guideline process critics expressed fears that there was not yet sufficient evidence from studies to answer many important questions.

This argument deserves to be taken seriously, but on the other hand a PubMed search in the literature of the term “ventral hernias” produces 8000 and “incisional hernias” 2700 publications. To find answers to problems occurring in daily practice in this endless flood of information is difficult even for experts. The development of a guideline is positively a matter of obligation.

It must above all determine through careful study of the scientific literature which of our diagnostic and therapeutic measures is to be regarded as verified, where there are pointers to solutions but there is not yet convincing evidence, and where there are merely personal opinions.

This is a task which cannot be carried out by one expert alone. The preconditions for the development of a reliable guideline are therefore:

1. an international -if possible global - panel of experts
2. the experts to be qualified by publications in peer-review journals
3. if possible two experts to be available working up one specific topic
4. complete transparency of the process of development of the guideline and clear communication line between the experts
5. A consensus conference and agree process.

The development process of the following guideline ran in a form similar to the development of the “Guidelines for laparoscopic (TAPP) and endoscopic (TEP) treatment of inguinal Hernia [International Endohernia Society (IEHS)]“ (Surg Endosc 2011;25: 2773-2843).

**We started the guideline development process in January 2011 by collecting the most important questions and assembling the most qualified experts in laparoscopic hernia repair.** An invitation was sent to all well-known laparoscopic hernia specialists who have made outstanding contributions to ventral/incisional hernia surgery published in peer-review journals. Approximately 40 Experts from three continents were invited to participate in a Consensus Conference aimed at developing guidelines for laparoscopic treatment of ventral and incisional abdominal wall hernias. The conference was planned to be set up within the framework of the 5^th^ Meeting of the International Endohernia Society (IEHS), organized for October 2011 in Suzhou/China by Prof. Ji ZL/Nanjing, Prof. Yao QY/Shanghai and Prof. Wu HR/Suzhou. The following questions were asked:

1. Are you willing to participate?

2. Are you interested in an active participation?

3. In your opinion what are the most important questions in laparoscopic surgery of abdominal wall hernias?

4. What topic do you wish to prepare according to the criteria of Evidence Based Medicine - to be able to give a recommendation at the conference?

5. What other experts in incisional/ventral hernia repair do you suggest we should invite for active participation?

On the basis of the answers received, 38 topics were identified as most important and 25 surgeons declared their willingness to draft the respective guideline.

**In a second step, the experts were asked to:**

(1) search the literature available on the topic, and (2) grade the papers according to the **Oxford hierarchy of evidence** (following the advice of Dr. S. Sauerland) as outlined below consisting of the following five levels:

1A. Systematic review of RCTs (with consistent results from individual studies).

1B. RCTs (of good quality).

2A. Systematic review of 2B studies (with consistent results from individual studies).

2B. Prospective comparative studies (or RCT of poorer quality).

2C. Outcome studies (analyses of large registries, population based data, etc.).

3. Retrospective, comparative studies, case–control studies.

4. Case series (i.e., studies without control group).

5. Expert opinion, animal or lab experiments.

**For the recommendations the following grading scale are to be used:**

**A**    consistent level 1 studies => strict recommendations ("standard", "surgeons must do it.")

**B**   consistent level 2 or 3 studies or extrapolations from level 1 studies => less strict wording ("recommendation", "surgeons should do it.")

**C**    level 4 studies or extrapolations from level 2 or 3 studies => vague wording ("option", "surgeons can do it.")

**D**    level 5 evidence or worryingly inconsistent or inconclusive studies at any level => no recommendation at all, describe options.

However, there is often a need to upgrade or downgrade a recommendation because the outcome is so important or the clinical preference is so strong. This is possible, but needs to be explained in the commentary text .

The experts were requested to prepare a paper to present at the Consensus Conference in Suzhou.

In Suzhou ( Consensus Conference and 5^th^ Meeting of the International Endohernia Society (IEHS) 13.-16.10.2011), the papers were discussed first in the round of experts, and the most important one day later during the plenary session attended by several hundred participants. During the following months, the authors drafted the first version of their specific chapter including all the suggestions they had received during the conference. These first versions were distributed to all the other experts for criticisms, comments and supplements. During these weeks, countless mails and revisions of papers were exchanged to achieve definitive guidelines which all experts could agree upon.

The guidelines focus on technique and perioperative management of laparoscopic ventral hernia repair. They are the first comprehensive guidelines regarding this topic. The advantages of the guidelines presented here are: 1. The authors come from Europe, America, and Asia; thus the guidelines are, effectively, global. 3. The authors use the Oxford hierarchy of evidence comprising 5 levels; thus, big case series could be included, altogether giving a more realistic representation of generally applied practice.

**Coordination of the process of development and editing of the guidelines:**

**Reinhard Bittner**, MD, Professor of Surgery, Dr.h.c. mult.,FRCS; visceral surgeon, em. Director, Department of Visceral and General Surgery, Marienhospital Stuttgart. Seniordirector, Center of Minimally Invasive Surgery, Bethesda Krankenhaus Stuttgart.Currenty Director Hernia Center Rottenburg. More than 350 original articles and more than 600 scientific lectures. About 50 live demonstrations of TAPP, cholecystectomy, colonic resection in 19 countries in Europe and Asia. Former President of the German Society for Visceral and General Surgery. Former President of the German Association of Minimal Surgery. Vice-President and former Congress President of the German Hernia Society.

**Working group:**

**Dieter Berger,**M.D., Professor of Surgery, Chairman Department of Surgery Stadtklinik Baden-Baden. Numerous live demonstrations incisional and parastomal hernia repair. Numerous national and international meetings as speaker and moderator. 7 peer-revieved publications. Currently President of the German Hernia Society.

**Juliane Bingener-Casey,M.D.** Associate Professor of Surgery, College of Medicine, Mayo Clinic. She chairs the Department of Surgery Quality Committee and is Department of Surgery Vice Chair of Quality, Safety and Service at Mayo Clinic. She has taught laparoscopic ventral hernia repair for SAGES and was instrumental in standardizing laparoscopic hernia repair across all Mayo Clinic sites. She has over 50 peer-reviewed publications in the literature.

**Eli Chelala,** M.D., Associate professor in Digestive Surgery at University Hospital of Tivoli –Belgium. 11 publications in peer-revieved Journals. More than 60 live demonstrations in hernia surgery at the I.R.C.A.D- Strasbourg, Germany-IEHS, UK, Norway, UE, Austria-ICS, Kuwait, KSA. More than 150 national and international congresses as speaker and moderator. Board member of the Belgian section abdominal wall surgery. President Societe`Medicale EuroLibanaise.

**Pradeep Chowbey**, MD, Dr.h.c., FACS, Director of Minimal Access, Metabolic, and Bariatric Surgery, Max Healthcare Institute Ltd.,Saket, New Delhi, India. Honorary Surgeon to the President of India. Surgeon to His Holiness Dalai Lama. Founder President of the Asia-Pacific Hernia Society. Former President of the Obesity & Metabolic Surgery Society of India. Trustee & Former President of the Indian Association of Gastrointestinal Endo-Surgeons. President elect of the Asia Pacific Metabolic & Bariatric Surgical Society. 75 Original Articles. Two Books. Educational Set of 15 CD-ROMs. Editor: Journal of Minimal Access Surgery. Editorial Board: Hernia. Obesity Journal. Indian Journal of Surgery. Journal of Society of Endoscopic and Laparoscopic Surgeons of Asia.

**Ulrich Dietz,** M.D., Associated Professor Department of Surgery University of Wuerzburg. 40 Original Articles. Member of 5 national and international societies. Advisory Board of Study Affairs University of Wuerzburg.

**George S. Ferzli,** MD, FACS. Professor of Surgery. SUNY Health Science Center, Brooklyn, NY, USA. Chairman of the Department of Surgery at Lutheran Medical Center. Director of the Medical Fellowship Program. More than 100 Original Articles in Peer Reviewed Journals. More than 10 Chapters in Medical Textbooks.

**René Fortelny**, MD, Univ.-Lector. Chief Resident, 2nd Department of Surgery, Wilhelminenspital, Vienna, Austria. General , visceral and abdominal wall surgeon, Head of the Hernia Center at the Wilhelminenspital, Head of the Experimental Hernia Group at the Ludwig Boltzmann Institute for Experimental and Clinical Traumatology, Austria, Vienna. Former President of the Austrian Society for Minimal Invasive Surgery. President of the Austrian Hernia Society. 22 Publications in Peer Review Journals. 45 Scientific Lectures. 25 Live Demonstrations in Hernia Repair.

**Uwe Klinge**, MD. General and Visceral Surgeon. Principal Investigator of the Surgical Department, University of Aachen, and Institut for Applied Medical Engineering AME Helmholtz. Special fields of research: Biocompatibility of meshes. Visualization of meshes. Wound healing. 163 publications cited in PubMed, 53 Book Chapters, 127 invited lectures.

**Ferdinand Köckerling**, MD. Professor of Surgery. Chairman of the Department of Surgery and Center of Minimally Invasive Surgery at the Vivantes Hospital in Berlin, Teaching Hospital of CharitéMedical School. Former President of the German Society for Minimally Invasive Surgery. Former President of the German Society for General and Visceral Surgery. Former Congress President of the German Hernia Society. Editorial Board: Surg Endosc, Langenbeck`s Archives of Surgery. 173 Papers cited in PubMed, More than 400 presentations in national and international conferences.

**Jan Kukleta**, MD, general, visceral, abdominal wall surgeon. Klinik im Park, Zürich, Switzerland. Director of the Endoscopic Training Center Zürich. Lecturer at the ESI Hamburg and Elancourt Paris. More than 50 hernia-specific contributions at international meetings.

**Karl LeBlanc**, M.D. Director, Minimally Invasive Surgery Institute and the Fellowship Program, Baton Rouge, Managing Partner, Surgeons Group of Baton Rouge of Our Lady of the Lake Physician Group, Baton Rouge, LA**.** President of American Hernia Society Foundation, Founding member, American Society of General Surgeons, Past President American Hernia Society.

**Davide Lomanto**, MD, PhD ,FAMS. Associate Professor, Senior Consultant, Director of the Minimally Invasive Surgical Center; Director of the KTP Advanced Surgical Training Center; YYL School of Medicine, National University of Singapore. President of Asia-Pacific Hernia Society. General Secretary of the Asia Pacific Bariatric Surgery Society. President of the Endoscopic& Laparoscopic Surgeons of Asia(ELSA). Editorial Board: Asian Journal of Laparo-Endoscopic Surgery. Chinese Journal of Hernia and Abdominal Wall Surgery. 85 Original Articles in Peer Review Journals. 14 Book Chapters. 3 Books. 184 Scientific Lectures. Instructor/Mentor in 113 live surgery workshops.

**Mahesh Chandra Misra**, MD. Head of the Department of Surgical Disciplines and Chief; J P N Apex Trauma Center, All India Institute of Medical Sciences, New Delhi, India. General, Visceral, and Trauma Surgeon. 70 papers in Peer Review Journals. 50 Scientific Lectures. 25 Live Surgery Demonstrations.

**Salvador Morales-Conde**, MD. Chief of the Advanced Laparoscopic Unit of the University Hospital "Virgen del Rocío" (Sevilla, Spain). Head of the General, Digestive and Laparoscopic Surgery Unit of the USP-"Sagrado Corazón" Clinic (Sevilla, Spain). Associate Professor of the University of Sevilla (Spain). Director of the National program of training on Laparoscopic Surgery of the Spanish Association of Surgery. President of the Spanish Society of Abdominal Wall Surgery. Secretary of the Spanish Society of Endoscopic Surgery. Member of the Board of European Hernia Society. Authors of several papers and chapters of books and of the book entitled “Laparoscopic ventral hernia repair”. Former Congress President of the European Hernia Society.

**Filip Muysoms,** M.D. Doctorate School of Life Sciences and Medicine,University of Ghent,Belgium. Over 30 original articles. Organizer and presenter in more than 120 national and international meetings. Congress President 33^rd^ International Congress of the European Hernia Society Ghent2011. Chairman of the European Registry for Abdominal Wall Hernias (EuraHS). Member of 7 national and international societies.

**Bruce Ramshaw,**M.D**.** Chairman, Department of General Surgery, Transformative Care Institute,

Daytona Beach, Florida, Clinical Associate Professor, Florida State University. Member of 14 national

and international societies. Over 100 peer review publications and over 250 invited presentations.

Director of more than 50 courses for training laparoscopic surgery. Currently program director of the

American Hernia Society (AHS).

**Wolfgang Reinpold**, MD, general surgeon, Director of the Department of Surgery, Gross Sand Hospital Hamburg . Director of the Hernia Center in Hamburg Wilhelmsburg. Special interest: risk factors for pain and pain treatment after hernia repair. One randomized and 7 prospective studies on pain and new techniques in inguinal and incisional hernia repair, whether open or laparoscopic. More than 30 hernia specific presentations at international meetings. Former Congress President of the German Hernia Society.

**Matthias Rohr,**M.D. Consultant. Specialist General Surgery. Katutura State Hospital, Windhoek, Namibia. 8 papers in peer-reviewed journals; 82 scientific lectures; 7 live surgery demonstrations. Congress President IEHS 2013.

**Rudolf Schrittwieser,** M.D. Head of the Department of Surgery at Public Hospital of Bruck an der Mur, Austria Secretary of AHC (Austrian Hernia community), Member of Zürser Hernienforum. Scientific lectures (mainly about hernia surgery) in Austria, Germany, Belgium. Lectures and tutoring for Workshops in minimal invasive surgery since 2002 in Austria, Germany, France, Iran, China.

**Thomas Simon,**M.D. University Hospital Heidelberg , Department of General, Visceral and Transplantation Surgery, Im Neuenheimer Feld 110, D-69120 Heidelberg. Vice-Surgeon Department of Surgery, GRN-Klinik Sinsheim and GRN-Klinik Eberbach, affiliated hospitals to the University. Eight publications. Five lectures at the annual meetings of the German Society of Surgery. Board member of the German hernia registry “Herniamed”

**Bernd Stechemesser,**M.D., Director Herniacenter Cologne PAN-Klinik Zeppelinstraße 1, 50667 Cologne. Presentation, organization and active participation in numerous hernia

conferences in Germany and abroad. Founder and scientific director of the series "Berlin-Hernientage" and the continuing education unit “Hernie Kompakt”. Member of 7 national and international societies.

**Michael Timoney**, MD. Attending Surgeon/ Director of Quality Assurance, Lutherian Medical Center, Brooklyn NY, USA. 5 Papers and Abstracts in Peer Reviewed Journals and 1 Correspondence in N Engl J Med.

**Dirk Weyhe,** MD, PhD. General and Visceral Surgeon. Head of the Department of Surgery, Pius Hospital Oldenburg, Germany. Speciality: Biocompatibility of synthetic materials. 50 Publications in Peer Reviewed Journals. 2 Book Contributions. 99 Scientific Lectures. Former Congress President of the German Hernia Society.

In summary, the guidelines have been developed by leading hernia surgeons coming from Europe, America, and Asia, working in high spirits and in an atmosphere of deep friendship. The result is a truly global achievement pointing to the future. We wish to thank all contributors for their tireless efforts and their unwavering dedication to hernia surgery without any remuneration or compensation even for traveling expenses.

If you do a PubMed literature research using the term “ hernia surgery”, you will find 29939 publications. The Guidelines should assist the surgeon in his clinical practice to make the right decision and to improve his technical performance. For validation and agreement, every expert received at least twice all the chapters written by the other authors. All comments and critics were seriously discussed with the respective author and, if necessary, the statements and recommendations were revised accordingly.

The Guidelines are valid until December 2015. The update meeting will be organized in due time by the first and last author.

**Section 1: Basics**

**How comparable are incisional and ventral hernias in terms of operative technique and outcomes?**

Bruce Ramshaw MD

Acknowledgements: Uwe Klinge for review and editing of content, Jerome Berlin PhD for review and editing of content, Brandie Forman for review and clerical assistance
Search terms (publications identified as pertinent to this topic/total publications returned by search): variability of incisional hernia (3/5), variability of ventral hernia (2/8), laparoscopic ventral hernia variability (0/0), laparoscopic incisional hernia repair variability (0/1), complexity of ventral hernia repair (2/14), complexity of laparoscopic ventral hernia repair (2/8), complexity of incisional hernia repair (0/7), complexity of laparoscopic incisional hernia repair (0/5)

The search was performed in October, 2011 and a total of four unique publications were returned from this search. All four were clinical studies. A secondary search revealed an additional 22 publications pertinent to this topic, ten which were studies and twelve publications which were not clinical studies.

# Statements

| Level 4 | The level of complexity and variability for ventral/incisional hernia patients and techniques for repair is high. |
| --- | --- |
| Level 5 | The degree of complexity is growing higher at an increasing rate of change. The techniques and outcomes, therefore, cannot be considered comparable using current methods of analysis. This is due to the many complex ever changing variables, as well as, relationships between variables, which are not controllable. |

Recommendations:

| Grade C | Because of the increasing pace of change and the complexity of ventral/incisional hernia patients and techniques, use of traditional human subjects clinical research evidence-based methods and guidelines in healthcare should be considered a starting point, rather than a goal. |
| --- | --- |
| Grade C | The application of principles of complex adaptive systems science, particularly real-world clinical quality improvement methods, will likely be required to improve the value of care (quality outcomes measures, satisfaction, patient experience, costs, etc.) for the patient with a ventral/incisional hernia. |

**Introduction**

What once was considered a relatively simple problem by many physicians and patients, abdominal wall hernia disease, is clearly more complex than previously thought. In addition, the patient groups presenting with incisional and ventral hernias are becoming more complex as the treatment options, including the varieties of mesh, continue to grow. This increasing complexity as well as the variability of outcomes leads us to challenge the traditional application of evidence-based medicine, which until now does not include knowledge generated from clinical quality improvement studies. This is not to say that this understanding of evidence-based medicine does not have value for complex problems, such as abdominal wall hernia disease. It is, however incomplete, and is but a starting point rather than a goal towards the understanding of how to improve the value of care for both the patient who presents with a ventral/incisional hernia and for the system in which that care is provided. This chapter will describe the current evidence for the variability of ventral/incisional hernia patients and present a brief framework for understanding how to apply new thinking to the study of complex problems such as ventral/incisional hernia disease.

During the past 150 years, traditional clinical research methods have been based on reductionist scientific approaches, where the scientific method is applied to the study of one part, or variable (a drug or device, for example), within a complex system (a patient’s cycle of care, for example). This approach to medical research has led to significant improvements in healthcare. Without the ability to perform prospective, randomized controlled trials many improvements in health care would not have been achieved. However, a closer look at advances in healthcare reveals that many significant innovations did not come from well-planned studies based on the traditional application of the scientific method; they often were discovered by accident or by innovators outside of the traditional scientific community.(1,2) Many treatments that have been approved through rigorous scientific scrutiny have later been proven to cause unexpected and unintended harm or have been found to have unexpected benefits for other, unrelated diseases.(3,4) Even major medical initiatives, such as the human genome project, have emerged through loose collaborations and relationships between various individuals and often between various types of experts.(5) More recently, many health care research initiatives are being initiated by patients and family members who have been frustrated by the lack of medical knowledge generated by our traditional research mechanisms; e.g., the women who started studies on spontaneous coronary artery dissection because none were available, and the two mothers from Old Lyme, CT who initiated the studies elucidating the cause of Lyme Disease.(6,7)

A new field of medicine is forming, referred to as complex adaptive systems research.(8) Complex adaptive systems describe any biologic organism (the human body, for example) and any grouping of biologic organisms (our healthcare system, for example). Research conducted to generate evidence based on the study of complex adaptive systems includes clinical quality improvement methods, participatory research (sometimes led by patients and family members) and the documentation of data throughout the entire cycle of patient care including psychosocial and other non-traditional outcomes measures. This field recognizes that humans likely belong to many subgroups that must be identified, in order to better predict outcomes and improve value. These subgroups may be based on genetics, environment, disease states, age, sex, etc. Many researchers are realizing that the traditional application of reductionist methods of research is often inadequate in the search to improve the value of patient care. (9) One reason these traditional research methods are inadequate is the realization that, as our medical knowledge increases exponentially, an almost infinite number variables appear, having an almost infinite number of complex relationships between them. And these relational interactions can impact the outputs leading to an escalating degree of complexity in health care and our world in general.(10) In addition, these variables and relationships are constantly changing and are not controllable. In light of this increasing complexity, traditional research methods alone are not sufficient to improve the value of care for the patient or to improve the value of the overall healthcare system.(11)

**Research:**

This knowledge of complex adaptive systems and increasing complexity impacts our understanding of the variability we see for the patient with a ventral/incisional hernia. Variability that can impact outcomes for ventral/incisional hernia repair may include patient factors, technique variability, surgeon skill, variability in mesh characteristics, and also the variability in both the environmental conditions present in the patient’s home living conditions, as well as at the facility where treatment occurs. Studies on the variability of ventral/incisional hernias are few, but a comparison of studies of different types of ventral/incisional hernias clearly shows a large variety of outcomes based upon many complex factors. One study within the US Veterans Affairs system showed significant variation in the use of mesh for ventral/incisional hernia repair, which correlated with less recurrence in the facilities in which mesh was used more often (up to a four-fold increase in mesh utilization).(12) In a study using a similar VA data, the location of mesh placement also impacted outcomes, with laparoscopic and underlay mesh placement leading to lower recurrence rates compared with onlay and inlay mesh placement.(13)

One prospective clinical study attempted to define some of the complex variables involved in laparoscopic ventral/incisional hernia repair. (14) Jenkins et. al. documented significant variation for a number of variables from a group of 180 patients with data collected prospectively. Significant variation was documented for patient age, BMI, number of previous open abdominal procedures (0-13), previous laparoscopic procedures (0-6), number of prior hernia repairs (0-8) and many other patient factors. Significant variation was also documented for the actual operative procedure with wide variation in the time required for adhesiolysis, mesh placement and overall operative time. Variables that increased the time required for adhesiolysis included history of COPD, presence of bowel adhesions and a suprapubic hernia location. Suprapubic location and incarceration of hernia contents significantly increased the time for mesh placement and total operative time. Presence of bowel adhesions also significantly increased the total operative time. Another study looking at laparoscopic ventral/incisional hernia repair for hernias in a suprapubic location resulted in increased complication and recurrence rates compared to a large study of laparoscopic ventral/incisional hernia repair that included all locations. (15,16) Other location variability such as flank, subcostal, parastomal, etc. would also be expected to have an impact on surgical outcomes, especially if the surgeon has had little experience performing ventral/incisional hernia repair for hernias in these atypical locations.

BMI can also be a variable impacting the outcomes of laparoscopic ventral/incisional hernia repair. In one study of more than 1,000 patients by Tsereteli, et. al. morbidly obese patients had a four-fold increase in recurrence compared to non-morbidly obese patients.(17) In addition to obesity, another patient factor that can significantly impact outcomes is the size of the defect and the amount/volume of herniated contents. Outcomes such as operative time, complications and recurrence rates differ greatly for laparoscopic ventral/incisional hernia repair of small defects as opposed to loss of domain hernias. (18,19)

A variety of factors can also be seen to impact the post-operative course of patients undergoing ventral/incisional hernia repair. In studies evaluating factors related to need for mesh removal, post-operative complications, recurrence rates, surgical site infection and resource utilization patient demographics (male sex, history of smoking, etc.), hernia characteristics (size of defect, incarceration, etc.), and technique factors (laparoscopic, open, etc.) all had the potential to contribute to differences in outcomes.(20-24)

Another complex variable potentially impacting outcomes of ventral/incisional hernia repair is the choice of mesh material. Although most synthetic meshes used today produce good short-term results, any mesh could contribute to complications in a given subgroup of patients. A partial list of mesh related complications includes: infection requiring mesh removal, mesh mechanical failure, mesh bulging, chronic pain, chronic inflammatory reaction and mesh erosion into abdominal viscera. (25,26) With the number and variety of hernia meshes available for ventral/incisional hernia repair, this variable alone is enough to demonstrate that traditional research mechanisms (i.e. prospective randomized controlled clinical trials) will be inadequate to determine the mesh (or meshes) that is/are of best value for various patient groups, hernia types, techniques, surgeon skill levels, etc. With an understanding of complexity science, complex systems, continuous learning and continuous clinical quality improvement, we will begin to be able to understand and improve value for patients who present with a ventral/incisional hernia. The starting point for this endeavor is the best current available evidence, much of which is contained in the remaining chapters of this document.

**Summary:**

In summary, the traditional human subjects clinical research approach to generate evidence-based medicine guidelines alone is unable to produce improved value for patient care that will be significant and sustainable for our increasingly complex healthcare system. Specifically, the increasing variability in ventral/incisional hernia patients and technique options minimizes the value of applying traditional research methods to improve outcomes. We will need to change our thinking and learn how to understand and implement research methods designed to address this increasing complexity in order to fully address healthcare challenges, such as ventral/incisional hernia disease. This will not only include an evolution of traditional/current evidence-based medicine, but also an evolution of evidenced-based management in health care. Because complex systems research is most often applied in the real-world of patient care in the community, hospital, clinic and even the academic medical center, we will need to apply the principles of continuous learning and continuous clinical quality improvement to our regular patient care in addition to using traditional clinical research methods. As we apply these new principles (new to healthcare , although currently used in other industries) and learn how to utilize complexity science driven data analytics, the patient clusters that emerge will guide our treatment options and lead to improved value for our entire system. We should do this by including the patient in a shared decision process and with an entire medical team, caring for the person who is the patient. Our focus on improving value for the patient should be our uncompromising purpose.

**References:**

1. [Fleming A](http://www.ncbi.nlm.nih.gov.medezproxy.net.ucf.edu/pubmed?term=%22Fleming%20A%22%5BAuthor%5D) (1929,1980) Classics in infectious diseases: on the antibacterial action of cultures of a penicillium, with special reference to their use in the isolation of B. influenzae by Alexander Fleming, Reprinted from the British Journal of Experimental Pathology 10:226-236, 1929. [Rev Infect Dis.](http://www.ncbi.nlm.nih.gov.medezproxy.net.ucf.edu/pubmed/6994200) Jan-Feb;2(1):129-39. **(level 5)**
2. Litynski GS (1998) Kurt Semm and the fight against skepticism: endoscopic hemostasis, laproscopic appendectomy, and Semm’s impact on the “laproscopic revolution”. JSLS 2(3):309-313. **(level 5)**

Ito T, Handa H (2012) Deciphering the mystery of thalidomide teratogenicity. Congenit Anom (Kyoto) 52(1):1-7. **(level 5)**

Ban TA (2006) The role of serendipity in drug discovery. Dialogues Clin Neurosci 8(3):335-344. **(5)**

McKusick VA (2006) A 60-year tale of spots, maps, and genes. Annu Rev Genomics Hum Genet. 7:1-27. **(level 5)**

Campbell SF (2000) Science, art and drug discovery: a personal perspective. [Clin Sci (Lond).](http://www.ncbi.nlm.nih.gov.medezproxy.net.ucf.edu/pubmed/10995589) Oct;99(4):255-60. **(level 5)**

Elbaum-Garfinkle S (2011) Close to home: a history of Yale and Lyme disease. Yale J Biol Med. 84(2):103-108. **(level 5)**

Custers EJFM, Stuyt PMJ, De Vries Robbé PF (2000) Clinical Problem Analysis (CPA): A Systematic Approach to Teaching Complex Medical Problem Solving. Acad. Med 75: 291-297. **(level 5)**

Diez Roux AV (2011) Complex systems thinking and current impasses in health disparities research. Am J Public Health. 101(9):1627-1634. **(level 5)**

Tian Q, Price ND, Hood L (2012) Systems cancer medicine: towards realization of predictive, preventive, personalized and participatory (P4) medicine. J Intern Med. 271(2):111-121. **(level 5)**

1. Wierling C, Kuhn A, Hache H, Daskalaki A, Maschke-Dutz E, Peycheva S, Li J, Herwig R, Lehrach H (2012) Prediction in the face of uncertainty: A Monte Carlo-based approach for systems biology of cancer treatment. Mutat Res [Epub ahead of print] **(level 5)**
2. Hawn MT, Snyder CW, Graham LA, Gray SH, Finan KR, Vick CC (2011). Hospital level variability in incisional hernia repair technique affects patient outcomes. Surgery. Feb; 149(2): 185-91. **(level 4)**
3. Gray SH, Vick CC, Graham LA, Finan KR, Neumayer LA, Hawn MT (2008) Variation in mesh placement for ventral hernia repair: an opportunity for process improvement? Am J Surg. 196(2):201-206. **(level 4)**
4. Jenkins ED, Yom VH, Melman L, Pierce RA, Schuessler RB, Frisella MM , Eagon JC, Brunt LM, Matthews BD (2010) Clinical predictors of operative complexity in laparoscopic ventral hernia repair: a prospective study. Surg Endosc. 24:1872-1877. **(level 4)**
5. Varnell B, Bachman S, Quick J, Vitamvas M, Ramshaw B, Oleynikov D (2008) Morbidity associated with laparoscopic repair of suprapubic hernias. Am J Surg. 196(6):983-7. **(level 4)**
6. Heniford BT, Park A, Ramshaw BJ, Voeller G (2003) Laparoscopic repair of ventral hernias: nine years' experience with 850 consecutive hernias. Ann Surg. 238(3):391-9. **(level 4)**
7. Tsereteli Z, Pryor BA, Heniford BT, Park A, Voeller G, Ramshaw BJ (2008) Laparoscopic ventral hernia repair (LVHR) in morbidly obese patients. Hernia. 12(3):233-8. **(level 4)**
8. Garcea G, Ngu W, Neal CP, Robertson GS (2012) Results from a consecutive series of laparoscopic incisional and ventral hernia repairs. Surg Laparosc Endosc Percutan Tech. 22(2):131-5. **(level 4)**
9. Baghai M, Ramshaw BJ, Smith CD, Fearing N, Bachman S, Ramaswamy A (2009) Techniques of laparoscopic ventral hernia repair can be modified to successfully repair large defects in patients with loss of domain. Surg Innov. 16(1):38-45. **(level 4)**
10. Dunne JR, Malone DL, Tracy K, Napolitano L (2003) Abdominal wall hernias: risk factors for infection and resource utilization. J of Surg Research. 111, 78-84. **(level 4)**
11. Kaafarani H, Kaufman D, Reda D, Itani K (2010) Predictors of surgical site infection in laparoscopic and open ventral incisional herniorrhaphy. J of Surg Research. 163, 229-234. **(level 4)**
12. Blatnik JA, Harth KC, Aeder MI, Rosen MJ (2011) Thirty-day readmission after ventral hernia repair: predictable or preventable? Surg Endosc. 25:1446-1451. **(level 4)**
13. Hawn MT, Gray SH, Snyder CW, Graham LA, Finan KR, Vick CC (2011) Predictors of mesh explantation after incisional hernia repair. Am J of Surg. 202., 28-33. **(level 4)**
14. Bencini L, Sanchez LJ, Bernini M, Miranda E, Farsi M, Boffi B, Moretti R (2009) Predictors of recurrence after laparoscopic ventral hernia repair. Surg Laparosc Endosc Percutan Tech. 19(2):128-32. **(level 4)**
15. Robinson TN, Clarke JH, Schoen J, Walsh MD (2005) Major mesh-related complications following hernia repair: events reported to the Food and Drug Administration. Surg Endosc. 19(12):1556-60. **(5)**
16. Schoenmaeckers E, Wassenaar EB, Raymakers Johan, Rakic S (2010) Bulging of the mesh after laparoscopic repair of ventral and incisional hernias. JSLS. 14(4):541-546. **(level 4)**

**Is the routine application of CT and MRI recommended for the diagnosis of ventral hernias prior to laparoscopic ventral hernia repair?**

R Schrittwieser

Pubmed search: Search terms:

„CT-scan“ AND „ventral hernia“ AND „laparoscopy“

"hernia, ventral"[MeSH Terms] OR ("hernia"[All Fields] AND "ventral"[All Fields]) OR "ventral hernia"[All Fields] OR ("ventral"[All Fields] AND "hernia"[All Fields])) AND ("laparoscopy"[MeSH Terms] OR "laparoscopy"[All Fields]) AND ("tomography, x-ray computed"[MeSH Terms] OR ("tomography"[All Fields] AND "x-ray"[All Fields] AND "computed"[All Fields]) OR "x-ray computed tomography"[All Fields] OR ("CT"[All Fields] AND "scan"[All Fields]) OR "CT scan"[All Fields])

„MRI“ AND „ventral hernia“ AND „laparoscopy“

("hernia, ventral"[MeSH Terms] OR ("hernia"[All Fields] AND "ventral"[All Fields]) OR "ventral hernia"[All Fields] OR ("ventral"[All Fields] AND "hernia"[All Fields])) AND ("magnetic resonance imaging"[MeSH Terms] OR ("magnetic"[All Fields] AND "resonance"[All Fields] AND "imaging"[All Fields]) OR "magnetic resonance imaging"[All Fields] OR "mri"[All Fields]) AND ("laparoscopy"[MeSH Terms] OR "laparoscopy"[All Fields])

The search was performed in August 2011.

The first search detected 53 articles. There remained 21 relevant articles for the pre- and postoperative use of a CT scan and 3 relevant articles for the use of MRI.

**Key questions:**

**Is a CT scan routinely indicated in the diagnosis of a ventral hernia?**

**Is a MRI routinely indicated in the diagnosis of a ventral hernia**

**STATEMENT**

| Level 5 | There is insufficient evidence for the use of CT/MRI in the daily routine  In some cases, especially posttraumatic hernias, obese patients, large hernias with loss of domain or special rare entities like Lumbar hernias a CT scan or MRI can be helpful. |
| --- | --- |

**Recommendation**

| Grade D | In special cases like posttraumatic hernias, special, rare entities like lumbar hernias or Spieghelian hernias and also in connection with obesity a CT scan or MRI may be considered . |
| --- | --- |

**Key question**

**How important are CT scan and MRI in postoperative diagnosis?**

**Statement**

| Level 2b | In postoperative diagnosis of recurrent hernia a CT scan is superior to clinical examination |
| --- | --- |

**Recommendations**

| Grade B | To find a recurrence or associated pathologies a CT scan should be done. |
| --- | --- |
| Grade D | To find postoperative adhesions a functional cine MRI can be used. |

Clinical investigation ranks first for the diagnosis of ventral hernia.

There are however cases whereby a more extensive preoperative diagnosis with CT or MRI would be recommended.

The available literature is concerned above all with investigations involving specific entities (1-13). In most of the cases it is concerned more with case series. An investigation into the application of CT and MRI is lacking for all ventral hernia types.

With abdominal trauma a CT scan is recommended, amongst other things, to identify potential traumatic ventral hernias.

Killeen et al (1) investigated the CT scan results of patients with blunt abdominal trauma and traumatic lumbar hernias. 9 out of 14 patients had concomitant injuries and of the 14 patients only 1 had clinical signs of a hernia. Likewise Hickey et al (3) highlighted in a retrospective study of 15 traumatic abdominal wall hernias, which were all correctly diagnosed by a CT scan and subsequently intraoperatively confirmed, the high frequency of above all mesenterial and intestinal injuries.

The CT scan can therefore, alongside the diagnosis of traumatic abdominal wall hernias, provide valuable information concerning concomitant injuries, hernia condition or potential haematoma.

In some case series or case reports the significance of the CT scan for the diagnosis of uncommon abdominal wall hernias could be demonstrated (5, 7-13).

Gough et al (9) described the discovery of an incarcerated Spieghelian hernia as the cause of an acute abdominal pain within the context of a CT scan.

Skrekas et al (5) highlight the case of a patient with swelling in the left lumbar region without trauma or previous surgery. The CT scan showed a superior lumbar hernia (Grynfeltt Hernia).

In the case of obese patients a CT scan can also be helpful. Rose et al (4) reported concerning 3 obese patients whose clinical examination was not able to detect a hernia. The CT scan showed a ventral hernia as being the cause of the complaint.

In terms of the preoperative use of MRI in the diagnosis of ventral hernias there are currently no studies available.

The current view is against carrying out a CT scan for all ventral hernias. It is recommended to use it however in cases of obesity, repeated preliminary operations, large hernias with possible loss of domain, traumatic hernias and to diagnose uncommon ventral hernias.

In terms of the use of CT scans following LVHR there are currently a number of studies available (14-21).

Gutierrez de la Pena et al (14) described 50 patients with LVHR who 1 year after surgery underwent a clinical investigation, a CT scan and diagnostic laparoscopy. Relapses were correctly diagnosed in 98% of the cases by CT and in 88% of the cases by clinical investigation.

Wagenblast et al (15) highlighted in a prospective study of 35 patients with LVHR, of which 4 patients suffered swelling, that in every case the CT scan was able to differentiate exactly between a seroma and a relapse.

For MRI there are currently only studies concerning the formation of adhesions following LVHR with a cine-MRI (22-24)

The CT scan is the method of choice for the postoperative differential diagnosis of relapses, seroma, bulging or the condition of remaining hernias. An ultrasound investigation can be helpful in the detection of seromas, but cannot offer as many anatomical details as the CT scan (21).

**References:**

1. Killeen KL, Girad S, DeMeo JH, Shanmuganathan K, Mirvis SE. Using CT to diagnose traumatic lumbar hernia. AJR Am. Journal of Roentgenology; 2000 May;174(5):1413-15**(level 4)**

2. Palanivelu C, Rangarajan M, Jategaonkar PA, Amar V, GokulKS, Srikanth B. Laparoscopic Repair of diastasis recti using the “Venetian blinds” technique of plication with prosthetic reinforcement: a retrospectice study. Hernia 2009;13:287-92**(level 4)**

3. Hickey NA, Ryan MF, Hamilton PA, Bloom C, Murphy JP, Brenneman F. Compute tomography of traumatic abdominal wall hernia and associated deceleration injuries. Can AssocRadiol J. 2002 Jun;53(3):153-9**(level 4)**

4. Rose M, Eliakim R, Bar-Ziv Y, Vromen A, Rachmilewitz D. Abdominal wall hernias. The value of computed tomography diagnosis in the obese patient. J ClinGastroenterol. 1994 Sept;19(2):94-6 **(level 4)**

5.Skrekas G, Stafyla VK, Papalois VE. A Grynfeltt Hernia: Report of a case. Hernia 2005;9:188- 91**(level 5)**

6 .Iannitti DA, Biz WL. Laparoscopic repair of a traumatic lumbar hernia. Hernia 2007;11:537-40**(level 5)**

7. Habib E. Retroperitoneoscopic tension-free repair of a lumbar hernia. Hernia 2003;7:150-52**(level 5)**

8. Habib E, Elhadad A. Spieghelian hernia long considered as diverticulitis: CT scan diagnosis and laparoscopic treatment. Surgical Endoscopy 2003 Jan;17 (1) 159**(level 5)**

9. Gough VM, Vella M. Timely computed tomography scan diagnosis Spieghelian hernia: a case study. Ann R CollSurg Engl. 2009 Nov;91(8):676**(level 5)**

10 .Bathla L, Davies E, Fitzgibbons RJ Jr, Cemaj S. Timing of traumatic lumbar hernia repair: is delayed repair safe? Report of two cases and review of the literature. Hernia 2011 Apr;15(2):205-9**(level 5)**

11. Meinke AK. Totally extraperitoneal laparoendoscopic repair of lumbar hernia. Surg Endosc 2003;17 734-7**(level 5)**

12. Links DJR, Berney CR. Traumatic lumbar hernia repair: a laparoscopic technique for mesh fixation with an iliac crest suture anchor. Hernia 2011;15(6) 691-3**(level 5)**

13. Yavuz N, Ersoy YE, Demirkesen o, Tortum OB, Erguney S. Laparoscopic incisional lumbar hernia repair. Hernia 2009;13:281-6**(level 5)**

14. Gutierrez de la Pena C, Vargas Romero J, Dieguez Garcia JA.The value of CT diagnosis of hernia recurrence after prosthetic repair of ventral incisional hernias.EurRadiol. 2001:11(7):1161-4**(level 2b)**

15 .Wagenblast AL, Kristiansen VB, Fallentin E, Schulze S. Computed tomography scanning and recurrence after laparoscopic ventral hernia repair. Surg Laparosc Endosc Percutan Tech. 2004 Oct;14(5):254-6**(level 4)**

16. Sharma A, Mehrotra M, Khullar R, Soni V, Baijal M, Chowbey PK. Laparoscopic ventral/incisional hernia repair: a single center expreince of 1242 patients over a period of 13 years. Hernia 2011;15:131-9**(level 5)**

17. Raftopoulos I, Courcoulas AP. Outcome of laparoscopic ventral hernia repair in morbidly obese patients with a body mass index exceeding 35kg/m2. SurgEndosc. 2007 Dec;21(12):2293-7**(level 5)**

18. Wassenaar EB, Shoenmeckers EJP, Raymakers JTF, Rakic S. Recurrences after laparoscopic repair of ventral and incisional hernia: lessons learned from 505 repairs. Surg. Endosc. 2009:23:825-32**(level 5)**

19 .Tsomoyannis EC, Siakas P, Glantzounis G, Koulas S, Mavridou P, Gossios Kl. Seroma in laparoscopic ventral hernioplasty. Surg. Laparosc Endosc Percutan Tech. 2001 Oct;11(5):317-21**(level 5)**

20 .Gossios K, Zikou A, Vazakas P, Passas G, Glantzouni A, Glantzounis G, Kontogiannis D, Tsimoyannis E. Value of CT after laparoscopic repair of postsurgical ventral hernia. Abdom Imaging. 2003 Jan-Feb;28(1):99-102**(level 4)**

21 .Tse GH, Stuchfield BM, Duckworth AD, de Beaux AC, Tulloh B. Pseudo-recurrence following laparoscopic ventral and incisional hernia repair. Hernia 2010 Dec;14(6):583-7**(level 4)**

22. Mussak T, FischerT, Ladurner R, Gangkofer A, Bensler S, Hallfeldt KK, Reiser M, Lienemann A. Cinemagnetic resonance imaging vs high-resolution ultrasonography for detection of adhesions after laparoscopic and open incisional hernia repair: a matched pair pilot analysis. Surg. Endosc. 2005 Dec;19(12):1538-43**(level 2b)**

23. Fischer T, Ladurner R, Gangkofer A, Mussak T, Reiser M, Lienemann A. Functional cine MRI of the abdomen for the assessment of implanted synthetic mesh in patients after incisional hernia repair: initial results. Eur. Radiol. 2007 Dec;17(12)3123-9**(level 4)**

24 .Zinther NB, Zeuten A, Marinovskij E, Haislund M, Friis-Andersen H. Functional cine MRI and transabdominal ultrasonography for the assessment of adhesions to implanted synthetic mesh 5-7 years after laparoscopic ventral hernia repair. Hernia 2010 Oct;14(5):499-504**(level 4)**

**Classification**

U.A. Dietz, F. Muysoms, M. Rohr

Search terms: *"incisional_hernia" AND "classification", "ventral_hernia" AND "classification", "incisional_hernia" AND "randomized_controlled_trial".*

A systematic search of the available literature was performed in January 2012 using Embase, PubMed and Cochrane library as well as manual search of relevant references using the above listed search terms. The first search detected 70 articles in Embase, 112 articles in Pubmed and 14 articles by manual search of the literature regarding the utilization of classification criteria. After excluding duplicates and articles not relevant to the key questions, 30 articles were included for this review.

**2 Key questions**

**2.1 Is it necessary to classify ventral and incisional hernias?**

**Which classification is recommended?**

**Statements:**

| Level 5 | There is consensus among experts, that it is necessary to classify ventral and incisional hernias prospectively in order to create a useful dataset to improve the understanding of the disease, to allow comparability of results, to substantiate patients counseling and optimize therapeutic algorithms. |
| --- | --- |

**Recommendations:**

| Grade D | It is recommended to classify ventral and incisional hernias prior to surgical therapy.  It is recommended that the EHS classification for ventral and incisional hernias is used. |
| --- | --- |

**2.2 Are the classification criteria included in the EHS classification consistent?**

**Statements:**

| Level 2B | Number of previous repairs and reducibility have been demonstrated to increase the risk of postoperative seroma. |
| --- | --- |
| Level 2C | Risk factors have been shown to influence the incidence of repeat recurrences. |
| Level 3 | The incidence of SSI is increased in patients with recurrent incisional hernias, with chronic steroid use and in smokers.  Morphology and size of the hernia may influence the type of procedure.  Width of the hernia gap has been shown as a predictive factor for postoperative complications. Length of the hernia has been demonstrated as independent prognostic factor for repeat recurrences. |
| Level 4 | Risk factors, hernia gap size and morphology can influence the time needed for the surgical procedure.  Smoking, male gender, BMI, age, SSI and postoperative wound complications are risk factors for the development of an incisional hernia. |

**Recommendations:**

| Grade B | Number of previous repairs, morphology, size of the hernia gap, risk factors and reducibility should be part of any classification system and should be recorded in the patient files. |
| --- | --- |
| Grade C | Risk factors, hernia gap size and morphology should be part of any classification, they should be considered in planning (tailoring) the surgical procedure.  There is no algorithm yet known to reduce the incidence of SSI in patients with risk factors. These patients should be informed about the increased risk during preoperative counseling. |

**3 Comments**

**3.1 Is it necessary to classify ventral and incisional hernias?**

**Which classification is recommended?**

Classification systems are necessary to structure the way scientific knowledge is collected and analyzed. This is an essential part of science itself. Since the triumphal procession of the TNM-classification of tumors and the ICD-classification of diseases in general, classification systems have also shown their high and indispensable significance in diagnostic, therapeutic and prognostic decision making as well as in patients counseling. One may postulate, that the unfounded confidence of surgeons in the effectiveness of mesh-implantation to cure incisional hernias in the early 80ies has dazzled surgeons and kept them away from realize the importance of a classification system for incisional (and ventral) hernias also. In the meantime, the systematic tumor-follow-up regimens and the ageing of the population have increased the frequency of diagnosed incisional hernias. Additionally, the onset of the obesity epidemics and the development of laparoscopic techniques challenged new approach and therapeutic algorithms. As a result of these convergent historical phenomena, an awareness of the importance of the incisional hernia problem started to arise among surgeons. In chronological order, classifications for ventral and incisional hernias were proposed first by Chevrel and Rath (2000) [2], followed by Korenkov et al. (2001) [15], Ammaturo et al. (2005) [1], Chowbey et al (2006) [3], Dietz et al. (2007) [6], Muysoms et al. (2009) [22] and Hadeed et al (2011) [8]. In a comparative analysis of the criteria included in all these classification proposals, it becomes clear that there is some agreement regarding the basic criteria of morphology and size of the hernia gap, although not one of them experienced an appreciable acceptance in the literature. The classification proposed by the European Hernia Society (EHS) (Muysoms et al., 2009) is the result of a comprehensive discussion of the criteria to be included and also of how to precise and define them [22]. The consensus finding goes back to a conference in Ghent (Belgium) in October 2008. Participants were hernia surgeons from Belgium, France, Germany, Italy, The Netherlands, Poland, Spain, Sweden and the United Kingdom. The EHS classification can be seen as advancement to all the preceding ones.

**3.2 Are the classification criteria included in the EHS classification consistent?**

The following discussion has the scope to illustrate the clinical importance of the classification criteria [13, 30]. The scarceness of evidence is pictured in the chart below (Figure 1). As prospective clinical trials on the subject classification are missing, the discussion is intended to wake the awareness and interest to this topic.

Recurrence rating is an underappreciated clinical factor, although it provides the surgeon with important information on the patient’s hernia history. The term recurrence rating comprises first the differentiation between ventral and incisional hernias and secondly the further differentiation of incisional hernias into the subcategory of recurrent incisional hernias. It is of utmost importance to differentiate between primary ventral hernias and incisional hernias, since the etiology and the prognosis of surgical therapy are different. In an analogous manner, the prognosis of recurrent incisional hernias is poorer also. The number of previous repairs has been demonstrated to increase the risk of postoperative seroma [11]. The incidence of SSI is increased in patients with recurrent incisional hernias [7] and is related to the surgical technique [14]. The incidence of postoperative complications is twofold higher in patients with incisional hernias in comparison with ventral hernias [7].

The EHS classification includes the morphology as defined by the "localization of the hernia" and defines essentially median and lateral hernias. There is no clear correlation in the literature between the localization of the hernia and the occurrence of postoperative complication or of recurrence after repair. Nevertheless, morphology may influence the type of procedure, for example in the subxiphoidal area [4, 5, 7, 18] or in the suprapubic region [7, 28]. In a non- randomized clinical trial with 199 patients, lateral incisional hernias had a different clinical presentation than medial hernias, with more preoperative pain and more postoperative complications [21]. Most of all, the localization of the hernia is of utmost importance for the surgical strategy: proximity to bony structures, tension in closing the gap or the composition of the fascia layers are to be considered [7, 10, 17]. The localization of the hernia correlates with the operative time [10]. For future comparison of data regarding surgical approach, layer of mesh insertion and quality of life, the localization of the hernia will be an important criterion [23, 24].

There is agreement in the EHS classification to measure the gap size during the surgical procedure, since the clinical estimation may be compromised by BMI or by a non-evident Swiss-cheese morphology. It is consensus, that the length of the hernia gap should be the greatest longitudinal distance between the proximal and distal margins of the hernia gaps, as it should be for the width in the transversal axis [22, 23]. Hernia width is a useful intraoperative variable in tailoring surgical procedures [7, 24, 25, 28]. Width of the hernia gap has been shown as a predictive factor for postoperative complications; length of the hernia has been demonstrated as independent prognostic factor for repeat recurrences [7]. Hernia gap size can also influence the time needed for the surgical procedure and is a marker for operative complexity [10, 16]. Related to the hernia gap is the reducibility of the sac contents. Non-reducible incisional hernias have been shown to correlate significantly with a seroma [11, 12].

Risk factors for the incidence of a first incisional hernia as a complication of a laparotomy were studied in large cohort series [9, 29] and potential risk groups [25]. In analogy, the same risk factors have been correlated with the incidence of recurrence after previous hernia repair. Smoking, male gender, BMI, age, SSI and postoperative wound complications are risk factors for the development of an incisional hernia [7, 10, 19, 24, 25, 26, 27]. There is experimental evidence, that patients with incisional hernias have an imbalance in the collagen metabolism [14]. Risk factors have been shown to influence the incidence of repeat recurrences [7]. As risk factors and co-morbidities are not yet understood, the working group of the European Registry of Abdominal Wall Hernias (EuraHS at www.eurahs.eu) introduced the definition of the SOC-score (severity of comorbidity score) to further refine the influence of risk factors on the course of ventral and incisional hernias [23]. Risk factors should be considered in tailoring the surgical procedure and in counseling the patient regarding the expected postoperative course and prognosis of recurrence in late follow up.


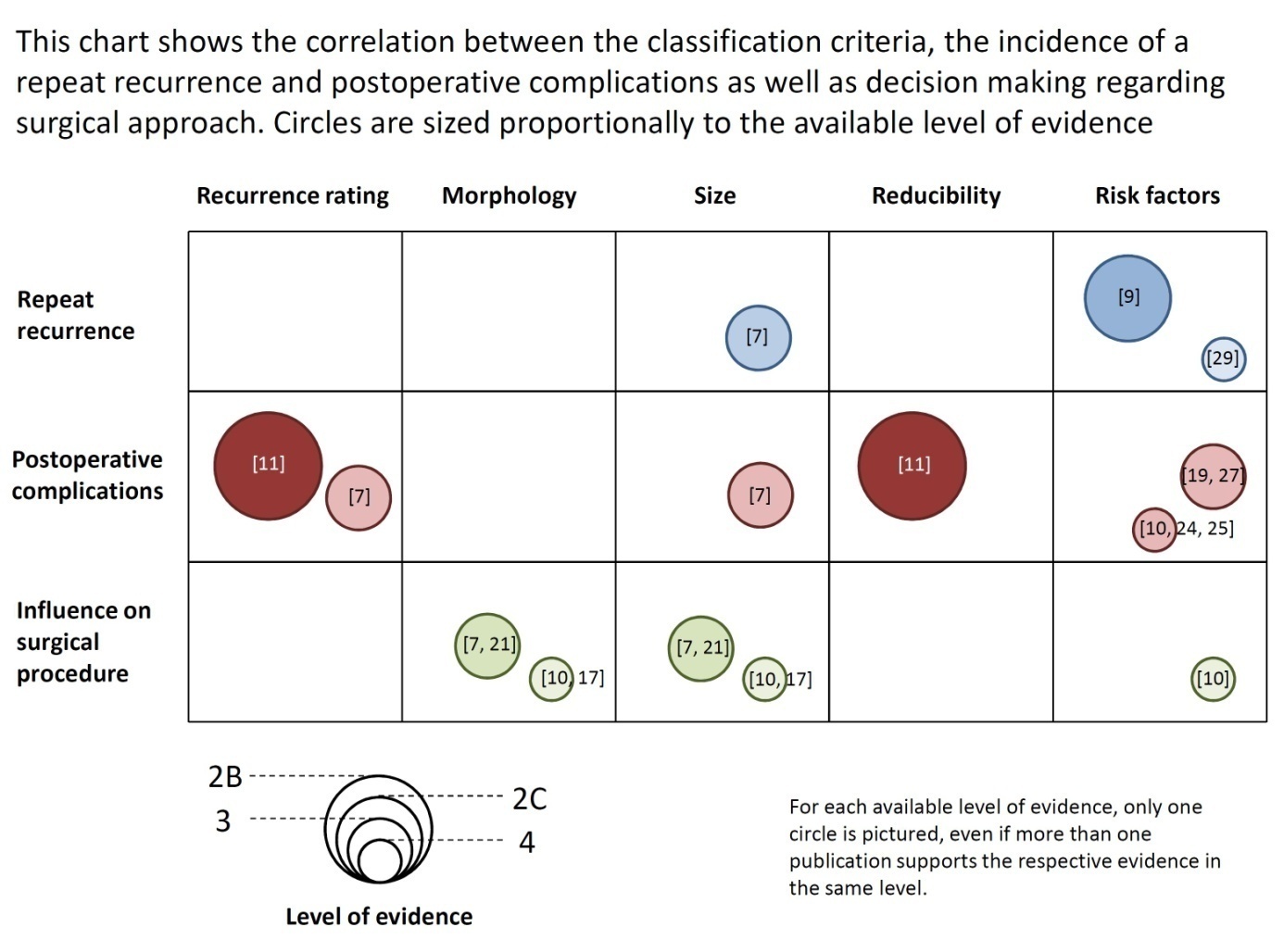


**Figure 1 –** Correlation between the classification criteria, the incidence of a repeat recurrence and postoperative complications as well as influence on decision-making regarding surgical approach. Circles are sized proportionally to the available level of evidence with respective references cited in each circle.

**Table 1 –** Literature overview on classification systems and the corresponding evidence on each criterion

| **Autor** | **Year** | **Type of study** | **Oxford** | **New classification** | **Utilization of a classification** | **Recurrence rating** | **Morphology** | **Size** | **Risk factoers** | **Surgical procedure** |
| --- | --- | --- | --- | --- | --- | --- | --- | --- | --- | --- |
| Ammaturo et al. [1] | 2005 | Case series | 4 | X |  |  | X | X |  |  |
| Chevrel et al. [2] | 2000 | Expert opinion | 5 | X |  |  |  |  |  |  |
| Chowbey et al. [3] | 2006 | Expert opinion | 5 | X |  |  |  |  |  |  |
| Conze et al. [4] | 2005 | Experimental | 5 |  |  |  | X |  |  | X |
| Conze et al. [5] | 2007 | Case series | 4 |  |  |  | X |  |  | X |
| Dietz et al. [6] | 2007 | Expert opinion | 5 | X |  |  |  |  |  |  |
| Dietz et al. [7] | 2012 | Retrospective case control | 3 |  | X | X | X | X | X |  |
| Hadeed et al. [8] | 2011 | Case series | 4 | X |  |  |  |  |  |  |
| Höer et al. [9] | 2002 | Outcome study | 2c |  |  |  |  |  | X |  |
| Jenkins et al. [10] | 2010 | Case series | 4 |  |  |  | X | X | X |  |
| Kaafarani et al. [11] | 2009 | RCT | 2B |  |  | X |  |  |  |  |
| Kaafarani et al. [12] | 2010 | RCT | 2B |  |  | X |  | X |  |  |
| Kingsnorth et al. [13] | 2006 | Review | 5 |  |  |  |  |  |  | X |
| Klinge et al. [14] | 2001 | Experimental | 5 |  |  |  |  |  | X |  |
| Korenkov et al. [15] | 2001 | Expert opinion | 5 | X |  |  |  |  |  |  |
| Leblanc et al. [16] | 2001 | Retrospective cohort | 4 |  |  |  |  | X |  |  |
| Licheri et al. [17] | 2008 | Case series | 4 |  | X |  | X |  |  |  |
| Losanoff et al. [18] | 2007 | Review | 5 |  |  |  | X |  |  | X |
| Martínez-S. et al. [19] | 2010 | Retrospective cohort | 3 |  |  |  |  |  | X |  |
| Moreno-Egea et al. [20] | 2007 | Review | 5 |  |  |  | X |  |  |  |
| Moreno-Egea et al. [21] | 2008 | NR-controlled trial | 3 |  |  |  | X |  |  |  |
| Muysoms et al. [22] | 2009 | Expert opinion | 5 | X | X | X | X | X | X |  |
| Muysoms et al. [23] | 2012 | Expert opinion | 5 |  | X |  |  |  | X |  |
| Parker et al. [24] | 2011 | Retrospective cohort | 4 |  |  |  |  | X |  |  |
| Piardi et al. [25] | 2010 | Retrospective cohort | 4 |  |  |  | X | X | X |  |
| Sanchez et al. [26] | 2011 | Review | 5 |  |  |  |  |  | X |  |
| Sorensen et al. [27] | 2005 | Retrospective cohort | 3 |  |  |  |  |  | X |  |
| Varnell et al. [28] | 2008 | Case series | 4 |  |  | X | X | X |  |  |
| Veljkovic et al. [29] | 2009 | Case series | 4 |  |  |  |  |  | X |  |
| Winkler et al. [30] | 2008 | Review | 5 |  | X | X | X | X |  | X |

**Literature**

1. Ammaturo C, Bassi G. The ratio between anterior abdominal wall surface/wall defect surface: a new parameter to classify abdominal incisional hernias. Hernia. 2005 Dec;9(4):316-21. **(level 4)**

2. Chevrel JP, Rath AM (2000) Classification of incisional hernias of the abdominal wall. Hernia 4:7-11. **(level 5)**

3. Chowbey PK, Khullar R, Mehrotra M, Sharma A, Soni V, Baijal M. Sir Ganga Ram Hospital classification of groin and ventral abdominal wall hernias. J Minim Access Surg. 2006 Sep;2(3):106-9**. (level 5)**

4. Conze J, Prescher A, Kisielinski K et al. (2005) Technical consideration for subxiphoidal incisional hernia repair. Hernia 9:84-87. **(level 4)**

5. Conze J, Krones CJ, Schumpelick V, Klinge U. Incisional hernia: challenge of re-operations after mesh repair. Langenbecks Arch Surg. 2007 Jul;392(4):453-7. **(level 4)**

6. Dietz UA, Hamelmann W, Winkler MS, Debus ES, Malafaia O, Czeczko NG, Thiede A, Kuhfuss I. An alternative classification of incisional hernias enlisting morphology, body type and risk factors in the assessment of prognosis and tailoring of surgical technique. J Plast Reconstr Aesthet Surg. 2007;60(4):383-8. **(level 5)**

7. Dietz UA, Winkler MS, Härtel RW, Fleischhacker A, Spor L, Isbert C, Jurowich Ch, Heuschmann P, Germer CT (2012) Importance of recurrence rating, morphology, hernial gap size and risk factors in ventral and incisional hernia classification. Hernia 2012; DOI 10.1007/s 10029-012-0999-x. (level 3)

8. Hadeed JG, Walsh MD, Pappas TN, Pestana IA, Tyler DS, Levinson H, Mantyh C, Jacobs DO, Lagoo-Deenadalayan SA, Erdmann D. Complex abdominal wall hernias: a new classification system and approach to management based on review of 133 consecutive patients. Ann Plast Surg. 2011 May;66(5):497-503*.* **(level 4)**

9. Höer J, Lawong G, Klinge U et al. (2002) Factors influencing the development of incisional hernia. A retrospective study of 2,983 laparotomy patients over a period of 10 years. Chirurg 73:474-480. **(level 2c)**

10. Jenkins ED, Yom VH, Melman L et al. (2010) Clinical predictors of operative complexity in laparoscopic ventral hernia repair: a prospective study. Surg Endosc 24:1872-1877. **(level4)**

11. Kaafarani HM, Hur K, Hirter A et al. (2009) Seroma in ventral incisional herniorrhaphy: incidence, predictors and outcome. Am J Surg 198:639-644. **(level 2b)**

12. Kaafarani HM, Kaufman D, Reda D, Itani KM. Predictors of surgical site infection in laparoscopic and open ventral incisional herniorrhaphy. J Surg Res. 2010 Oct;163(2):229-234. **(level 2b)**

13. Kingsnorth A (2006) The management of incisional hernia. Ann R Coll Surg Engl 88:252-260. **(5)**

14. Klinge U, Si ZY, Zheng H et al. (2001) Collagen I/III and matrix metalloproteinases (MMP) 1 and 13 in the fascia of patients with incisional hernias. J Invest Surg 14:47-54. **(level 5)**

15. Korenkov M, Paul A, Sauerland S, Neugebauer E, Arndt M, Chevrel JP, Corcione F, Fingerhut A, Flament JB, Kux M, Matzinger A, Myrvold HE, Rath AM, Simmermacher RK. Classification and surgical treatment of incisional hernia. Results of an experts' meeting. Langenbecks Arch Surg. 2001 Feb;386(1):65-73. **(level 5)**

16. LeBlanc KA, Booth WV, Whitaker JM, Bellanger DE. Laparoscopic incisional and ventral herniorraphy: our initial 100 patients. Kein PDF verfügbar Hernia. 2001 Mar;5(1):41-5. **(level 4)**

17. Licheri S, Erdas E, Pisano G, Garau A, Ghinami E, Pomata M. Chevrel technique for midline incisional hernia: still an effective procedure. Hernia. 2008 Apr;12(2):121-6. **(level 4)**

18. Losanoff JE, Basson MD, Laker S et al. (2007) Subxiphoid incisional hernias after median sternotomy. Hernia 11:473-479. **(level 5)**

19. Martínez-Serrano MA, Pereira JA, Sancho JJ, López-Cano M, Bombuy E, Hidalgo J; Study Group of Abdominal Hernia Surgery of the Catalan Society of Surgery. Risk of death after emergency repair of abdominal wall hernias. Still waiting for improvement. Langenbecks Arch Surg. 2010 Jun;395(5):551-6. **(level 3)**

20. Moreno-Egea A, Baena EG, Calle MC, Martínez JA, Albasini JL. Controversies in the current management of lumbar hernias. Arch Surg. 2007 Jan;142(1):82-8. **(level 5)**

21. Moreno-Egea A, Carrillo A, Aguayo JL. Midline versus nonmidline laparoscopic incisional hernioplasty: a comparative study. Surg Endosc. 2008 Mar;22(3):744-9. **(level 3)**

22. Muysoms FE, Miserez M, Berrevoet F, Campanelli G, Champault GG, Chelala E, Dietz UA, Eker HH, El Nakadi I, Hauters P, Hidalgo Pascual M, Hoeferlin A, Klinge U, Montgomery A, Simmermacher RK, Simons MP, Smietański M, Sommeling C, Tollens T, Vierendeels T, Kingsnorth A. Classification of primary and incisional abdominal wall hernias. Hernia. 2009 Aug;13(4):407-14. **(level 5)**

23. Muysoms F, Campanelli G, Champault GG, Debeaux AC, Dietz UA, Jeekel J, Klinge U, Köckerling F, Mandala V, Montgomery A, Morales Conde S, Puppe F, Simmermacher RK, Smietański M, Miserez M. EuraHS: the development of an international online platform for registration and outcome measurement of ventral abdominal wall hernia repair. Hernia. 2012. Jun;16(3):239-50. **(level 5)**

24. Parker M, Bray JM, Pfluke JM, Asbun HJ, Smith CD, Bowers SP. Preliminary experience and development of an algorithm for the optimal use of the laparoscopic component separation technique for myofascial advancement during ventral incisional hernia repair. J Laparoendosc Adv Surg Tech A. 2011 Jun;21(5):405-10. **(level 4)**

25. Piardi T, Audet M, Panaro F, Gheza F, Cag M, Portolani N, Cinqualbre J, Wolf P. Incisional hernia repair after liver transplantation: role of the mesh. Transplant Proc. 2010 May;42(4):1244-7. **(level 4)**

26. Sanchez VM, Abi-Haidar YE, Itani KM. Mesh infection in ventral incisional hernia repair: incidence, contributing factors, and treatment. Surg Infect (Larchmt). 2011 Jun;12(3):205-10. **(level 5)**

27. Sørensen LT, Hemmingsen UB, Kirkeby LT et al. (2005) Smoking is a risk factor for incisional hernia. Arch Surg 140:119-23. **(level 3)**

28. Varnell B, Bachman S, Quick J et al. (2008) Morbidity associated with laparoscopic repair of suprapubic hernias. Am J Surg 196:983-7. **(level 4)**

29. Veljkovic R, Protic M, Gluhovic A et al. (2010) Prospective clinical trial of factors predicting the early development of incisional hernia after midline laparotomy. J Am Coll Surg 210:210-9. **(level 4)**

30. Winkler MS, Gerharz E, Dietz UA (2008) Overview and evolving strategies of ventral hernia repair. Urologe Jun;47(6):740-7. **(level 5)**

**Section 2: Indication for Surgery**

**Indications for Treatment in dependence on size of defect or hernia sac,**

**hernia type, symptoms, age.**

Thomas Simon, MD

A systematic search was performed in Pubmed, Medline, Cochrane, Studyregister, relevant journals and reference lists including publications until 6^th^ of June 2012.

**Searchstrategy**

("delay"[ti] OR delaying[tiab])) OR (indication[tiab] AND surgery[tiab])) OR ("watchful waiting" OR "Watchful Waiting"[Mesh])) OR ("watch and wait" OR "wait and see" OR "wait and see policy")) OR (observation[mesh]) OR (observation[ti]) OR ("operation" AND compared AND "watchful waiting") AND ("Hernia"[Mesh]) OR ("Hernia, Inguinal"[Mesh] OR "Hernia, Diaphragmatic, Traumatic"[Mesh] OR "Hernia, Abdominal"[Mesh] OR "Hernia, Ventral"[Mesh] OR "Hernia, Umbilical"[Mesh] OR "Hernia, Obturator"[Mesh] OR (hernia OR hernias) OR ("Abdominal wall hernias") OR ("Abdominal wall hernia") OR ("ventral hernia") OR ("ventral hernias") OR ("umbilical hernia") OR ("umbilical hernias") OR ("primary hernia") OR ("primary hernias") OR ("epigastric hernia") OR ("epigastric hernias") OR ("lateral hernia") OR ("lateral hernias") OR ("incisional hernia" OR "incisional hernias") OR ("spieghelian hernia") OR ("spieghelian hernias")) OR ("flank hernia") OR ("flank hernias")AND (randomized controlled trial[pt] OR controlled clinical trial[pt] OR randomized[tiab] OR placebo[tiab] OR clinical trials as topic[mesh:noexp] OR randomly[tiab] OR trial[ti] NOT (animals[mh] NOT humans[mh])

The search produced 462 hits including inguinal hernias. 42 papers were relevant whereof 28 could be selected for this analysis. The only two Level 1b trials addressed inguinal hernias and where included with the intention to discuss the existing evidence in a related field. Regarding data addressing ventral and incisional hernias only one Level 3 study and 15 Level 4 uncontrolled studies could be found.

**Statements**

| **Level 4** | 33 – 78% of the patients with a ventral or incisional hernia develop symptoms |
| --- | --- |
| **Level 4** | 5 – 15% of the patients with a ventral or incisional hernia are operated on because of an acute complication (obstruction/strangulation)  Emergency repairs are associated with high morbidity  Umbilical hernias obstruct five times more than other ventral and incisional hernias |
| **Level 4** | Defect size of incisional hernias predicts recurrence rates |
| **Level 4** | There seems to be no difference in terms of morbidity and mortality regarding laparoscopic surgery on ventral hernias in advanced age.  Furthermore, the reduced risk of surgical site infections in laparoscopic techniques has an impact for elderly patients. |

**Recommendations**

| **Grade D** | Symptomatic ventral and incisional hernias should be treated surgically |
| --- | --- |
| **Grade D** | The laparoscopic technique for ventral and incisional hernias should preferable be reserved for defect sizes smaller than 10 cm in diameter |
| **Grade D** | The laparoscopic technique for ventral and incisional hernia repair can be used even in advanced age |

**Introduction**

There is no precise data available about the incidence and prevalence of ventral and incisional hernias.An epidemiological study showed an increasing proportion of midline abdominal wall hernias with a relative frequency of umbilical/paraumbilical hernias of 19 %, epigastric hernias of 8,6 % and incisional hernias of 4,8 % ^1^.The incidence for incisional hernias is 10 to 20 % ^2,3^,making it one of the most common surgical complication after laparotomies.

Ventral and incisional hernias are operated due to symptoms (pain and discomfort), to prevent complications (strangulation, respiratory dysfunction or skin problems) or when they present acute complications (incarceration and strangulation)^18^. It is still unclear, whether asymptomatic ventral and incisional hernias should be treated surgically and whether the indication for surgery should be influenced by the size of the hernia or the age of the patient.

**Symptoms**

The investigation regarding publications dealing with symptoms revealed 7 relevant papers whereas two are databases ^7^ and one a questionnaire ^5^. A study with long-term follow-up until 10 years including 564 patients, showed 11 % of patients developing an incisional hernia with 33 % having symptoms and 14 % suffering from obstruction ^3^.Vardanian et al published a retrospective review of 959 patients after liver transplantation. They found an incisional hernia rate of 4,6 % whereas 78 % suffered of pain and discomfort and 5 % presented incarceration or strangulation ^6^. In the series of Courtney et al also 78 % of patients were operated because of pain and 10 % presented acutely ^9^.In a series published by Hjaltason umbilical hernias incarcerated five times more than incisional hernias^10^.

**Acute hernia**

When an acute hernia occures, emergency repairs of abdominal hernias are associated with high morbidity ^11,4,16^. Davies et al demonstrated a significant proportion of patients presenting with acute hernia who were those managed by a 'watchful waiting' strategy before.The series of Alani et al presented an interestingly high rate of acute ventral hernia with nearly 50 % of their prospectively reviewed population. At the ratio of total hernias operated during the study period, the rate of acute ventral hernias of 12,2 % is still high ^12^ .For paediatric umbilical hernia, a retrospective review of 489 children presented 7 % acute hernias ^13^ .Earlier studies show an incarceration rate of 14,6 % and a strangulation rate of 2,4 % ^14^ .

**Indication in dependence on age**

Only one article providing evidence Level 4 included 155 patients in a retrospective analysis regarding the question whether advanced age is a contraindication for laparoscopic ventral hernia repair. They divided thestudy population in two groups with the threshold at 65 years and did not find a significant difference regarding morbidity and mortality^15^. Considering the results of the Cochrane review^29^comparing laparoscopic versus open surgical techniques for ventral and incisional hernia repair, the clear and consistent result of reduced risk for surgical site infections for the laparoscopic surgery has obviously great impact on elderly patients.

**Indication depending on size**

The systematic search revealed only one articlefocusing on defect size and outcome ^19^ . Moreno-Egea et al performed a prospective study without a control group, exluding hernias less than 5 cm diameter and those with ‘swiss-cheese’ defects. The average follow-up time in this single centre study was 60 month and recurrence was detected by clinical examination and computer tomography in unclear cases. The data analysis with a receiver operating characteristic curve analyzing the relation recurrence and defect size, showed that size predicts recurrence and they recommended to reserve the laparoscopic approach for a herniasize only up to 10 cm (Level 4). A retrospective single centre study of 302 patients, who underwent open repair with primary incisional hernia, analyzed several risk factors of recurrence and showed the size of the hernia as a significant risk factor for the development of recurrence^20^.

**Asymptomatic Hernias**

Regarding the natural course of ventral and incisional hernias, the search found no publication presenting any data. One long-term prospective study and one review showed 60 % of patients with incisional hernias do not have symptoms ^3,4^ .An international questionnaire among hernia specialists revealed a rate of 23 % of asymptomatic patients and more than 20 % of the patients did not receive surgery. The strangulation/incarceration rate was 5 % ^5^ .The group perceived that data describing the natural course of an incisional hernia is missing. Until now, patients with asymptomatic incisional hernias are operated to avoid complications. Precise data about the strangulation rate or the risk of acute incarceration of incisional hernias is missing. One small prospective case study disclosed an emergency operation rate of 3,2 % ^26^.The data from the Danish Ventral Hernia Database published by Helgstrand et al,showed a rate of acute hernias of 10 %, with the highest rate of umbilical herniaswith 57 % ^7^. There are no controlled trials analyzing the increase of size of incisional hernias over time, risk factors for strangulation or the development of discomfort and pain.

***Inguinal hernia – a different disease, a different approach ?***In contrast, the European Hernia Society published in the Guidelines for the treatment of inguinal hernias Level 1b evidence for a watchful waiting concept as an acceptable option ^21^. This is supported by two prospective randomized-controlled trials of the group of Fitzgibbons ^22^and the group of O’Dwyer^23^. The latter one demonstrated a very low acute incarceration rate for inguinal herias of 1.8 per 1000 patient-years. There was no difference between the ‘watchful waiting’ group and the surgery group regarding pain and discomfort in the fist two years. In a following analysis the group found no adverse effect on the final outcome when delaying surgery. In contrast inthe long-term follow up of 163 patients over 7.5 years, O’Dwyer demonstrated a crossover rate of 70 % from the watchful waiting group to surgery due to increasing pain ^24^. In a newly published systematic review the authors conclude that both, watchful waiting and surgery are treatment options for asymptomatic inguinal hernias, but most patients develop symptoms over time and will need surgical treatment^25^.

A prospective case study with consecutive patient series investigated whether patients benefit from surgery for incisional hernias with regard to pain ^26^. They could find no benefit regarding pain in the oligo-symptomatic group. To elucidate this unclear question about the indication for surgery for asymptomatic and oligosymptomatic incisional hernias there has been launched two prospective randomized controlled trials. The trial of Lauscher et al is the multicentre trial AWARE which is in the recruiting phase ^27^. A second trial finished already with data collection but is still unpublished^28^. In conclusion until now there is no conclusive data available regarding this issue and the publication of both trials has to be awaited.

**Studies analyzing incidence and rates of acute hernia**

|  | patients N | asymp-tomatic | acute | umbili-cal | incisio-nal | epiga-stric | morta-lity | morbi-dity |
| --- | --- | --- | --- | --- | --- | --- | --- | --- |
| Mudge M^3^ | 564 | 62 % | 14 % |  | 11 % |  |  |  |
| Helgstrand F^7^ | 6290 |  | 10 % | 45 % | 33 % | 16 % |  |  |
| Vardanian AJ^6^ | 959 | 17 % | 5 % |  | 4,6 % |  | 0 % | 20,5 % |
| Courtney CA^9^ | 120 | 22 % | 10 % | 26,6 % | 50 % | 23,3 % |  |  |
| McEntee GP^8^ | 79 |  | 100 % | 6,3 % | 2,5 % | 2,5 % | 9 % |  |
| Hjaltason E^10^ |  |  | 100 % | 17,7 % | 3,5 % |  | 25 % |  |
| Davies M^11^ | 39 |  | 100 % | 25,6 % | 12,8 % |  | 0 % | 46,2 % |
| Alani A^12^ | 91 |  | 100 % | 17 % | 20 % | 6,5 % | 3,8 % |  |
| Zendejas B^13^ | 34 |  | 100 % | 100 % |  |  |  | 2 % |
| Nieuwenhuizen J^16^ | 203 |  | 100 % | 25,6 % | 19,2 % | 6,9 % | 4,4 % |  |

**References**

1. Dabbas N, Adams K, Pearson K, RoyleG.Frequency of abdominal wall hernias: is classical teaching out of date?JRSM Short Rep. 2011 Jan 19; 2(1): 5. **.(Level 5)**

2 .Seiler CM, Bruckner T, Diener MK, Papyan A, Golcher H, Seidlmayer C, Franck A, Kieser M, Büchler MW, KnaebelHP.Interrupted or continuous slowly absorbable sutures for closure of primary elective midline abdominal incisions: a multicenter randomized trial (INSECT: ISRCTN24023541). Ann Surg. 2009 Apr; 249(4): 576-82.(**Level 1b)**

3.Mudge M, Hughes LE. Incisional hernia: a 10-year prospective study of incidence and attitudes.

Br J Surg. 1985 Jan; 72(1): 70-1.(**Level 4)**

4.Kingsnorth A, LeBlanc K. Hernias: inguinal and incisional. Lancet. 2003 Nov 8; 362(9395): 1561-71. (**Level 3)**

5.Nieuwenhuizen J, Kleinrensink GJ, Hop WC, Jeekel J, Lange JF.Indications for incisional hernia repair: an international questionnaire among hernia surgeons.

Hernia. 2008 Jun; 12(3): 223-5. (**Level 5)**

6.Vardanian AJ, Farmer DG, Ghobrial RM, Busuttil RW, Hiatt JR. Incisional hernia after liver transplantation. J Am CollSurg. 2006 Oct; 203(4): 421-5. ( **Level 4)**

7.Helgstrand F, Rosenberg J, Bay-Nielsen M, Friis-Andersen H, Wara P, Jorgensen LN, Kehlet H, Bisgaard T. Establishment and initial experiences from theDanish Ventral Hernia Database.

Hernia. 2010 Apr; 14(2): 131-5. (**Level 4)**

8.McEntee GP, O'Carroll A, Mooney B, Egan TJ, Delaney PV.Timing of strangulation in adult hernias.

Br J Surg. 1989 Jul; 76(7): 725-6.(**Level 4)**

9. Courtney CA, Lee AC, Wilson C, O'Dwyer PJ.Ventral herniarepair: a study of current practice.

Hernia. 2003 Mar; 7(1): 44-6.(**Level 4)**

10.[Hjaltason E](http://www.ncbi.nlm.nih.gov/pubmed?term=Hjaltason%20E%5BAuthor%5D&cauthor=true&cauthor_uid=7324753). Incarcerated hernia.ActaChir Scand. 1981; 147(4): 263-7.(**Level 4)**

11.[Davies M](http://www.ncbi.nlm.nih.gov/pubmed?term=Davies%20M%5BAuthor%5D&cauthor=true&cauthor_uid=17316522), [Davies C](http://www.ncbi.nlm.nih.gov/pubmed?term=Davies%20C%5BAuthor%5D&cauthor=true&cauthor_uid=17316522), Morris-Stiff G, Shute K. Emergency presentation of abdominal hernias: outcome and reasons for delay in treatment - a prospective study. Ann R CollSurgEngl2007 Jan; 89(1): 47-50. (**Level 4)**

12 .Alani A, Page B, O'Dwyer PJ. Prospective study on the presentation and outcome of patients with an acute hernia.Hernia. 2006Mar; 10(1): 62-5.(**Level 4)**

13. Zendejas B, Kuchena A, Onkendi EO, Lohse CM, Moir CR, Ishitani MB, Potter DD, Farley DR, Zarroug AE. Fifty-three-year experience with pediatric umbilical hernia repairs.J PediatrSurg. 2011 Nov; 46(11): 2151-6.(**Level 4)**

14. Read RC, Yoder G. Recenttrends in themanagement of incisionalherniation.ArchSurg. 1989 Apr; 124(4): 485-8.(**Level 4)**

15 .Saber AA, Elgamal MH, Mancl TB, Norman E, Boros MJ. Advanced age: is it an indication o rcontraindication for laparoscopic ventral hernia repair?JSLS. 2008 Jan-Mar; 12(1): 46-50.(**Level 4)**

16 .Nieuwenhuizen J, van Ramshorst GH, tenBrinke JG, de Wit T, van der Harst E, Hop WC, Jeekel J, Lange JF. The use of mesh in acute hernia: frequency and outcome in 99 cases.

Hernia. 2011 Jun;15(3):297-300. Epub 2011 Jan 23.(**Level 4)**

17. Bisgaard T, Kehlet H, Bay-Nielsen MB, Iversen MG, Wara P, Rosenberg J, Friis-Andersen HF, Jorgensen LN. Nationwide study of early outcomes after incisiona lhernia repair.

Br J Surg. 2009 Dec; 96(12): 1452-7.(**Level 4)**

18.Nieuwenhuizen J, Halm JA, Jeekel J, Lange JF. Natural course of incisional hernia and indications for repair.Scand J Surg. 2007; 96(4): 293-6. Review**.(Level 3)**

19. Moreno-Egea A, Carrillo-Alcaraz A, Aguayo-Albasini JL. Is the outcome of laparoscopic incisional hernia repair affected by defect size? A prospectivestudy.Am J Surg. 2012 Jan; 203(1): 87-94.(**Level 4)**

20 .Hesselink VJ, Luijendijk RW, de Wilt JH, Heide R, Jeekel J. An evaluation of risk factors in incisional hernia recurrence.SurgGynecolObstet. 1993 Mar; 176(3): 228-34.(**Level 4)**

21. Simons MP, Aufenacker T, Bay-Nielsen M, Bouillot JL, Campanelli G, Conze J, de Lange D, Fortelny R, Heikkinen T, Kingsnorth A, Kukleta J, Morales-Conde S, Nordin P, Schumpelick V, Smedberg S, Smietanski M, Weber G, Miserez M. European Hernia Society guidelines on the treatment of inguinal hernia in adult patients. Hernia. 2009 Aug;13(4):343-403. (**Level 1A)**

22 . Fitzgibbons RJ Jr, Giobbie-Hurder A, Gibbs JO, Dunlop DD, Reda DJ, McCarthy M Jr, Neumayer LA, Barkun JS, Hoehn JL, Murphy JT, Sarosi GA Jr, Syme WC, Thompson JS, Wang J, Jonasson O. Watchful waiting vs repairof inguinal hernia in minimally symptomatic men: a randomized clinical trial.JAMA. 2006 Jan 18;295(3):285-92. Erratum in: JAMA. 2006 Jun 21;295(23):2726.(**Level 1b)**

23.O’Dwyer PJ, Norrie J, Alani A, Walker A, Duffy F, Horgan P. Observation or operation for patients with an asymptomatic inguinal hernia: a randomized clinical trial. Ann Surg. 2006 Aug;244(2):167-73.(**Level 1b)**

24 .Chung L, Norrie J, O'Dwyer PJ. Long-term follow-up of patients with a painless inguinal hernia from a randomized clinical trial.Br J Surg. 2011 Apr;98(4):596-9. doi: 10.1002/bjs.7355. Epub 2010 Nov 30.(**Level 1b)**

25. Mizrahi H, Parker MC. Management of asymptomatic inguinal hernia: a systematic review of the evidence. Arch Surg. 2012 Mar; 147(3): 277-81. Review.(**Level 1a)**

26. Lauscher JC, Rieck S, Loh JC, Gröne J, Buhr HJ, Ritz JP. Oligosymptomatic vs. symptomatic incisional hernias--who benefits from open repair?Langenbecks Arch Surg. 2011 Feb; 396(2): 179-85. Epub 2010 Jun 28.(**Level 3)**

27 . Lauscher JC, Martus P, Stroux A, Neudecker J, Behrens U, Hammerich R, Buhr HJ, Ritz JP. The Development of a clinical trial to determine whether watchful waiting is an acceptable alternative to surgical repair for patients with oligosymptomatic incisional hernia: study protocol for a randomized controlled trial. Trials. 2012 Feb 7; 13:14**.(expected 1B)**

28 .Bellows C. Watchful waiting of incisional hernias. ClinicalTrials.gov NCT00351455**.(expected 1B)**

29 . Sauerland S, Walgenbach M, Habermalz B, Seiler CM, Miserez M.Laparoscopic versus open surgical techniques for ventral or incisional hernia repair.[Cochrane Database Syst Rev](http://www.ncbi.nlm.nih.gov/pubmed/21412910) 2011 Mar 16;(3): CD007781. (**Level 1a)**

**Is there still any place for open suture repair in dependence on defect size?**

J. Kukleta, Th.Simon, S.Morales-Conde

To answer the above question a systematic search of available literature was performed in August2011 and April 2012 using Pubmed, Medline, Cochrane Library and other relevant journals and reference lists with following search terms: “Small hernia” AND “non mesh repair” AND “suture repair” AND “recurrence” AND “infection” AND “umbilical hernia” AND “incisional hernia” AND “ventral hernia”.

The search detected 277 Metaanalysis, RCT’s and reviews on umbilical hernia (UB), UB and suture repair 100 articles, UB and recurrence 54, UB and infection 21 articles. For epigastric hernia (EH) we found 26 publications (metaanalysis, RCT’s and reviews). For small hernia (SM) 433 articles with filter Metaanalysis, RCT and review were found. From all above mentioned material and adding some important comparative studies 45 relevant articles were chosen for this review. Among these 19 with level of evidence la or lb, 4 with level ll, 14 with level lll and 6 with level 4.

**Questions to answer:**

**Do the outcomes of suture repair justify its use even in small hernias only?

Are there clear risk factors for recurrence identified that justify mesh repair in any size hernia?

Is the incidence of mesh infection relevant reason for suture repair of small hernias?**

**Statements**

| **Level 1B** | Suture herniorrhaphy is the simplest procedure among the open repair techniques.  The suture repair is associated with high recurrence rate.  Suture repair is accomplished in shorter operative time than mesh repair.  The mesh repair enables significantly lower recurrence rate than suture repair  The mesh repair seems to be safe method even in presence of non-viable bowel loops in case of incarcerated umbilical hernia  The wound complication rates can be slightly higher in mesh repair or are similar in both groups. |
| --- | --- |
| **Level 3** | Independent risk factors for recurrence in small hernias are not clearly defined. BMI, hernia size, wound infection in one study and smoking, obesity, size of hernia, type of repair, or chronic obstructive pulmonary disease in another study do not seem to predict recurrence in small hernia repair. In contrary to this one study reports a clear correlation between hernia size and/or BMI and the recurrence rate. |
| **Level 4** | Not every “small hernia” requires mesh repair.  Suture repair of hernias smaller than 2cm shows acceptable recurrence rate and low wound morbidity. |
| **Level 5** | Despite the existing evidence suture repair is still very popular in the surgical community |

**Recommendations**

| **Grade A** | In repair of primary defects bigger than 2cm or in recurrent hernias of any size the mesh repair should be considered as the first choice. |
| --- | --- |
| **Grade C** | The suture repair should be used only in very small primary defects of abdominal wall and in absence of any possible recurrence risk factor. |
| **Grade D** | Focussing on recurrence evidence is sufficiently strong to recommend that all defects of the abdominal wall, whether inguinal, incisional or umbilical hernias, and of whatever size, should be repaired with the use of prosthetic mesh. |

Comments:

The most studies on treatment of small abdominal wall hernias published between 2000 and 2012
would recommend mesh for the repair due to unacceptable high recurrence rate in suture repair.
The term “small hernia” is often used, although never precisely defined. A defect smaller or equal 2 cm, which continues to be repaired by suture by the vast majority of surgeons all over the world. Despite the clear message of Burger in 2004 [46] that “suture repair should be abandoned” the controversy remains.
Arroyo et al reports 2001 of a randomized controlled trial comparing suture and mesh repair in umbilical hernia in adults. The recurrence rate of suture repair was with 11% significantly higher (p=0.0015) than 1% in mesh repair [2].
Aslani et al presents 2010 a meta-analysis of RCT’s and an extensive review. All RCT’s favor mesh repair concerning recurrence and 8 of 10 cohort studies too. Wound complication rates are slightly higher for mesh repair in RCT’s and equal in cohort studies.
The retrospective study on comparison of mesh and suture repair by Sanjay (2005)[21] shows recurrence rates for mesh in 0% vs. 11.5% for suture repair. The infection rate for mesh repair was 0% vs. 11.5% for suture repair.
Stabilini et al [25]confirms 2009 after 10 years of experience recurrence rate of 14.7% in suture repair and 3.1% for mesh repair (p=0.0475).
Eryilmaz et al [19] concludes his prospective comparison concerning recurrence (2006) that all umbilical hernias regardless the size should be repaired by PP mesh.
As a contradiction to the above statements presents Dur et al [41] low recurrence rate in suture repair concluding that not every small hernia needs a mesh repair.
Risk factors:
Independent risk factors for recurrence in small hernia repair are not well defined.
Asolati et al [30] find that smoking, obesity, size of hernia, type of repair, or chronic obstructive pulmonary disease do not seem to predict recurrence of hernias. Halm et al [32] could not establish a relationship between a BMI over 30 kg/m2 and an increased recurrence rate but rather an increased recurrence rate from 5% to 18% with a BMI>25 kg/m2. ). Arroyo [2] did not find any significant relationship between recurrence rate and hernia size. The recurrence rates were similar for defects greater or smaller than 3 cm. The patient’s BMI of >30 kg/m2 was a risk factor for umbilical hernia recurrence. Schumacher [47] reported in his retrospective analysis of recurrence rate after mesh-free Spitzy’s repair a clear correlation between hernia size/ or BMI >30kg/m2 and the recurrence rate. According to their results a patient with BMI>30 /and /or hernia size >3cm should get a mesh repair.
Personal opinion: There is a lack of data on small hernia treatment in women in child-bearing age.

| **Author** | **Study** | **Nr. patients** | **OM / Rec** | **LM/Rec** | **ONM / Rec** | **Wound infection   OM / LM / ONM** |
| --- | --- | --- | --- | --- | --- | --- |
| **Abdel-Baki** | RCT | 42 | 21 / 0% |  | 21 / 19% |  |
| **Arroyo** | RCT | 200 | 1% |  | 11% | Similar |
| **Polat** | RCT | 50 | 17 PHS | 15 onlay | 18 Mayo |  |
| **Aslani** | Sys rev |  | 1% |  | 11% |  |
| **Asolati** | Retrosp | 229 | 132/ 3% |  | 97/ 7.7% |  |
| **Bowley** |  | 473 | 80/ 2.5% |  | 393/ 4% |  |
| **Ergul** | Case-series | 10+Lapchol | 0% |  |  |  |
| **Eryilmaz** | Prosp | 111 | 48/ 2% |  | 63/ 14% |  |
| **Farrow** | Retrosp | 152 | 1.5% |  | 9.2% | 19% |
| **Gonzales** | Retrosp | 76 | 20 / 20% | 32/ 0% | 24 / 8% | 15 0 0% |
| **Halm** | Retrosp | 131 | 12 / 0% |  | 119 / 13% |  |
| **Kamer** | Retrosp | 64 | 14 |  | 50 |  |
| **Lau** | Retrosp | 102 | 9/ 0% | 26/ 0% | 43 + 24 / 8.7% |  |
| **Malik** | Retrosp | 236 | 7.4% |  | 22.7% |  |
| **Solomon** | Retrosp | 724 | 227/ 1.8% | 301/1.0% | 146 / 30% | 1.3 2.2 5.5 % |
| **Sanjay** | Retrosp | 100 | 39/ 0.0% |  | 61 / 11.5% | 0.0 11.5% |
| **Stabilini** | Retrosp | 98 | 64/ 3.1% |  | 34 / 14.7% | 1.4 |
| **Venclauskas** | Retrosp | 97 | 5 |  | 92 |  |
| **Wright** | Retrosp | 116 | 20 | 30 | 66 |  |

**Tab.1** Umbilical hernia repair. Available number of patients and results.
**OM** open mesh repair **ONM** open non-mesh repair **LM** laparoscopic mesh repair **Rec** recurrence

| **Author** | **Mean F/U OM LM ONM** |
| --- | --- |
| **Abdel-Baki** | **16** |
| **Arroyo** | **64** |
| **Polat** | **22** |
| **Aslani** |  |
| **Asolati** | **40** |
| **Bowley** | **25 incomplete** |
| **Eryilmaz** | **37** |
| **Farrow** | **20** |
| **Halm** | **32** |
| **Kamer** | **25** |
| **Lau** | **24** |
| **Sanjay** | **33 61** |
| **Venclauskas** | **54** |
| **Wright** | **28** |
| **Solomon** | **47 56 54** |
| **Gonzales** | **25 22 28** |

**Tab. 2.** Duration of follow-up. **OM** open mesh repair, ONM open non-mesh repair
LM laparoscopic repair

References and Graduation of Evidence:

1. Abdel-Baki NA, Bessa SS, Abdel-Razek AH. Comparison of prosthetic mesh repair and tissue repair in the emergency management of incarcerated para-umbilical hernia: a prospective randomized study. Hernia. 2007 Apr;11(2):163-7. Epub 2007 Feb 2. (**level** **Ib)**

2. Arroyo A, García P, Pérez F, Andreu J, Candela F, Calpena R. Randomized clinical trial comparing suture and mesh repair of umbilical hernia in adults. Br J Surg. 2001 Oct;88(10):1321-3.  **(level Ib)**

3. Asencio F, Aguiló J, Peiró S, Carbó J, Ferri R, Caro F, Ahmad M. Open randomized clinical trial of laparoscopic versus open incisional hernia repair. Surgical Endoscopy 2009;23(7):1441–1448. [PUBMED: 19116750] **(level** **Ib)**

4. Aslani N, Brown CJ. Does mesh offer an advantage over tissue in the open repair of umbilical hernias? A systematic review and meta-analysis.

Hernia. 2010 Oct;14(5):455-62. Epub 2010 Jul 16. Review. PMID: 20635190 **(level** **Ia)**

5. Barbaros U, Asogulu O, Seven R, Erbil Y, Dinccag A, Deveci U,

Ozarmagan S, Mercan S. The comparison of laparoscopic and open

ventral hernia repairs : a prospective randomized study. Hernia

2006;11(1):51–56. [PUBMED: 17131072] **(level** **Ib )**

6. den Hartog D, Dur AHM, Tuinebreijer WE, Kreis RW (2008) Open surgical procedures for incisional hernias. Cochrane Database Syst Rev (3):CD006438 Meta-analysis. **(level** **Ia)**

7. Forbes SS, Eskicioglu C, McLeod RS, Okrainec A. Meta-analysis of randomized controlled trials comparing open and laparoscopic ventral and incisional hernia repair with mesh.

Br J Surg. 2009 Aug;96(8):851-8. Review. PMID: 19591158 **(level** **Ia)**

8. Goodney PP, Birkmeyer JD. Short term outcomes of laparoscopic and open ventral hernia repair: a meta-analysis. Archives of Surgery 2002;137(10):1161–5. [PUBMED: 12361426] **(level** **Ia)**

9. Polat C, Dervisoglu A, Senyurek G, Bilgin M, Erzurumlu K, Ozkan K. Umbilical hernia repair with the prolene hernia system. Am J Surg. 2005 Jul;190(1):61-4.  **(level Ib)**

10. Kapischke M, Schulz T, Schipper T, Tensfeld J, Caliebe A. Open versus laparoscopic incisional hernia repair: something different from a meta-analysis. Surgical Endoscopy 2008;22(10):2251–60. [PUBMED: 18320281] **(level** **Ia)**

11. Moreno-Egea A, Carrasco L, Girela E, Martín JG, Aguayo JL, Canteras M. Open vs laparoscopic repair of Spighelian hernia: a prospective randomized trial. Archives of Surgery 2002;137(11): 1266–8. [PUBMED: 12413315] RCT **(level Ib)**

12. Misra MC, Bansal VK, Kulkarni MP, Pawar DK. Comparison of laparoscopic and open repair of incisional and primary ventral hernia: results of a prospective randomized study. Surgical Endoscopy 2006;20(12):1839–45. [PUBMED: 17063290] **(level Ib** )

13. Navarra G, Musolino C, De Marco ML, Bartolotta M, Barbera A, Centorrino T. Retromuscular sutured incisional hernia repair: a randomized controlled trial to compare open and laparoscopic approach. Surgical Laparoscopy and Endoscopy 2007;17(2):86–90. [PUBMED: 17450086] **(level** **Ib)**

14. Olmi S, Scaini A, Cesana GC, Erba L, Croce E. Laparoscopic versus open incisional hernia repair: an open randomized controlled study. Surgical Endoscopy 2007;21(4):555–9. [PUBMED: 17364151] **(level** **Ib)**

15. Pring CM, Tran V, O’Rourke N, Martin IJ. Laparoscopic versus open ventral hernia repair: a randomized controlled trial. Australian and New Zealand Journal of Surgery 2008;78(10):903–6. [PUBMED: 18959646] **( level** **Ib)**

16. Sauerland S, Walgenbach M, Habermalz B, Seiler CM, Miserez M. Laparoscopic versus open surgical techniques for ventral or incisional hernia repair. Cochrane Database Syst Rev. 2011 Mar 16;(3):CD007781. Review. PMID: 21412910 **(level** **Ia)**

17. Itani KM, Hur K, Kim LT, Anthony T, Berger DH, Reda D, Neumayer L, for the Veterans Affairs Ventral Incisional Hernia Investigators. Comparison of laparoscopic and open repair with mesh for the treatment of ventral incisional hernia: a randomized trial. Archives of Surgery 2010;145(4):322–8. [PUBMED:20404280] **(level** **Ib)**

18. Korenkov M, Sauerland S, Arndt M, Bograd L, Neugebauer EA, Troidl H. Randomized clinical trial of suture repair, polypropylene mesh or autodermal hernioplasty for incisional hernia. Br J Surg. 2002 Jan;89(1):50-6. **(level** **IIa)**

19. Eryilmaz R, Sahin M, Tekelioglu MH. Which repair in umbilical hernia of adults: primary or mesh? Int Surg. 2006 Sep-Oct;91(5):258-61. **(level IIb)**

20. Wright BE, Beckerman J, Cohen M, Cumming JK, Rodriguez JL. Is laparoscopic umbilical hernia repair with mesh a reasonable alternative to conventional repair? Am J Surg. 2002 Dec;184(6):505-8; discussion 508-9. PMID:12488148**( level** **III)**

21. Sanjay P, Reid TD, Davies EL, Arumugam PJ, Woodward A. Retrospective comparison of mesh and sutured repair for adult umbilical hernias. Hernia 2005 Oct;9(3):248-51. Epub 2005 May 13. PMID:15891810 **(level III)**

22. Arroyo A, Pérez F, Serrano P, Costa D, Oliver I, Ferrer R, Lacueva J, Calpena R. Is prosthetic umbilical hernia repair bound to replace primary herniorrhaphy in the adult patient?

Hernia. 2002 Dec;6(4):175-7. Epub 2002 Oct 19. PMID: 18267162 **(level** **III)**

23. Thoman DS. Randomized clinical trial comparing suture and mesh repair of umbilical hernia in adults (Br J Surg 2001;88:1321-3). Br J Surg. 2002 May;89(5):627; author reply 628.

24. Vrijland WW, Jeekel J. Prosthetic mesh repair should be used for any defect in the abdominal wall. Curr Med Res Opin. 2003;19(1):1-3. **(level IV)**

25. Stabilini C, Stella M, Frascio M, De Salvo L, Fornaro R, Larghero G, Mandolfino F, Lazzara F, Gianetta E. Mesh versus direct suture for the repair of umbilical and epigastric hernias. Ten-year experience. Ann Ital Chir. 2009 May-Jun;80(3):183-7. **(level III)**

26. Farrow B, Awad S, Berger DH, Albo D, Lee L, Subramanian A, Bellows CF. More than 150 consecutive open umbilical hernia repairs in a major Veterans Administration Medical Center. Am J Surg. 2008 Nov;196(5):647-51. **(level** **III)**

27. Ergul Z, Ersoy E, Kulacoglu H, Olcucuoglu E, Devay AO, Gundogdu H. A simple modified technique for repair of umbilical hernia in patients undergo laparoscopic cholecystectomy. Report of 10 cases. G Chir. 2009 Oct;30(10):437-9. **(level** **IV)**

28. Kamer E, Unalp HR, Derici H, Tansug T, Onal MA. Laparoscopic cholecystectomy accompanied by simultaneous umbilical hernia repair: a retrospective study. J Postgrad Med. 2007 Jul-Sep;53(3):176-80. **( level** **III)**

29. Lau H, Patil NG (2003) Umbilical hernia in adults. Surg Endosc 17:2016–2020 **(level** **III)**

30. Asolati M, Huerta S, Sarosi G, Harmon R, Bell C, Anthony T. (2006) Predictors of recurrence in veteran patients with umbilical hernia: single center experience. Am J Surg 192:627–630 **(level** **IV)**

31. Bowley DMG, Kingsnorth AN (2000) Umbilical hernia, Mayo or mesh? Hernia 4:195–196 **(level** **IV)**

32. Halm JA, Heisterkamp J, Veen HF, Weidema WF (2005) Long term follow-up after umbilical hernia repair: are there risk factors for recurrence after simple and mesh repair. Hernia 9:334–337 **(level** **III)**

33. Venclauskas L, Silanskaite J, Kiudelis M (2008) Umbilical hernia: factors indicative of recurrence. Medicina (Kaunas, Lithuania) 44:855–859 **(level** **III)**

34. Solomon TA, Wignesvaran P, Chaudry MA, Tutton MG. A retrospective audit comparing outcomes of open versus laparoscopic repair of umbilical/paraumbilical herniae. Surg Endosc. 2010 Dec;24(12):3109-12. Epub 2010 May 20. PMID: 20490566 **(level**  **III)**

35. Malik AM, Jawaid A, Talpur AH, Laghari AA, Khan A. J Ayub. Mesh versus non-mesh repair of ventral abdominal hernias. Med Coll Abbottabad. 2008 Jul-Sep;20(3):54-6.PMID: 19610517

36. Martin DF, Williams RF, Mulrooney T, Voeller GR. Ventralex mesh in umbilical/epigastric hernia repairs: clinical outcomes and complications. Hernia. 2008 Aug;12(4):379-83. Epub 2008 Feb 29. PMID: 18309451 **(level** **IV)**

37. Gonzalez R, Mason E, Duncan T, Wilson R, Ramshaw BJ. Laparoscopic versus open umbilical hernia repair. JSLS. 2003 Oct-Dec;7(4):323-8. PMID: 14626398 **(level** **III)**

38. Franklin ME Jr, Gonzalez JJ Jr, Glass JL, Manjarrez A. Laparoscopic ventral and incisional hernia repair: an 11-year experience. Hernia 2004;8(1):23–7. [PUBMED: 14505237] **(level** **IV)**

39. Lomanto D, Iyer SG, Shabbir A, Cheah WK. Laparoscopic versus

open ventral hernia mesh repair: a prospective study. Surgical

Endoscopy 2006;20(7):1030–5. [PUBMED: 16703430**]( level** **IIb)**

40. Kamer E, Unalp HR, Derici H, Tansug T, Onal MA (2007) Laparoscopic cholecystectomy accompanied by simultaneous umbilical hernia repair: a retrospective study. J Postgrad Med

53:176–180 **( level** **III)**

41. Dur A, den Hartog D, Tuinebreijer WE, Kreis RW, Lange JF. Low recurrence rate of a two-layered closure repair for primary and recurrent midline incisional hernia without mesh. Hernia (2009) 13:421–426 DOI 10.1007/s10029-009-0487-0 **(level** **III)**

42. Itani KM, Neumayer L, Reda D, Kim L, Anthony T (2004) Repair of ventral incisional hernia: the design of a randomized trial to compare open and laparoscopic surgical techniques. Am J Surg 188(6A suppl):22S–29S.**(level** **lb)**

43. Itani K, Hur K, Kim L, Thomas A, Berger D, Reda D, Neumayer L. Ventral incisional hernia repair: comparison of laparoscopic and open repair with mesh . Hernia 2009;13(Suppl 1):S36. [: NCT00240188]

44. Halm JA, Heisterkamp J, Veen HF, Weidema WF (2005) Long term follow-up after umbilical hernia repair: are there risk factors for recurrence after simple and mesh repair. Hernia 9:334–337**(level** **III)**

45. Bowley DM, Kingsnorth AN (2000) Umbilical hernia, Mayo or mesh? Hernia 4:195–196 **(level** **IV)**

46. Burger JW, Luijendijk RW, Hop WC, Halm JA, Verdaasdonk EG, Jeekel J. Long-term follow-up of a randomized controlled trial of suture versus mesh repair of incisional hernia.

Ann Surg. 2004 Oct;240(4):578-83; discussion 583-5.**(level** **lb)**

47. Schumacher OP, Peiper C, Lörken M, Schumpelick V(2003). Long-term results after Spitzy's umbilical hernia repair. Chirurg. 2003 Jan;74(1):50-4. **(level** **III)**

**Limitations of laparoscopic intraperitoneal onlay mesh repair in terms of**

**defect size or body habitus**

Juliane Bingener, Matthias Rohr

*Search terms: “hernia” AND “ventral” AND “laparoscopy” AND “laparoscopic surgery” AND “postoperative complications or recurrence or pain” AND “postoperative or surgical wound infection” AND “prosthesis” AND “design/failure/implantation/device removal” AND “seroma” AND “pain” AND “limitations”.*

This resulted in a total of 946 citations from Ovid medliner 1948 – August 2011, PubMed including prepublication, Embase 1988 – 33^rd^ week of 2011, evidence-based medicine reviews and the Cochrane register, and the Web of Science from 1993 – 2011.

Out of these references, 17 full papers were reviewed to evaluate limitations for intraperitoneal onlay mesh repair.

From the review resulted the following statements and recommendations.

**Feasibility Regarding Obesity**

**Statements**

| Level 3 | Laparoscopic IPOM in obese patients is feasible (BMI >30) |
| --- | --- |
| Level 3 | Laparoscopic IPOM in morbidly obese patients is feasible (BMI >40) |
| Level 3 | Laparoscopic IPOM in super morbidly obese patients is feasible (BMI >50) |
| Level 4 | Laparoscopic IPOM is feasible in patients up to BMI 82 |

**IPOM Feasibility Hernia Size**

**Statements**

| Level 3 | Laparoscopic IPOM for defects >15 cm is feasible |
| --- | --- |
| Level 2B | Hernia recurrence in defects with a width >10 cm is more likely |
| Level 3 | Operating time is longer with defects >15 cm |
| Level 2B | Mesh size up to 1250 cm 2 is feasible |
| Level 4 | Mesh size up to 2400 cm 2 is feasible |
| Level 4 | LVHR is feasible up to 880 cm 2 defect size |

**Safety and Obesity**

**Statements**

| Level 3 | Complication rate for patients with BMI ≥40 undergoing LVHR is higher than for patients with BMI <40 |
| --- | --- |
| Level 2B | Recurrence rate is increased with BMI >30 |

**Recommendations**

| Grade B | Patients should be informed that LVHR is feasible in obese patients |
| --- | --- |
| Grade B | Patients should be informed that the risk of complications and hernia recurrence increases with BMI |
| Grade B | Patients should be informed that complications, wound infections are less likely for LVHR in obese patients compared to open |

**Large Hernia Compared to Open**

**Statements**

| Level 2B | LVHR results in the use of larger mesh sizes compared to open hernia repair |
| --- | --- |
| Level 2B | LVHR results in fewer superficial SSI than open repair in large hernias |
| Level 2B | LVHR results in decreased blood loss compared to open repair in large hernias |
| Level 3 | LVHR was associated with reduction in postoperative narcotics compared to open |
| Level 3 | LVHR was associated with shorter hospital stay compared to open |
| Level 3 | LVHR was associated with less ileus than open repair in large hernias |

**Recommendations**

| Grade B | Patients should be informed that LVHR is feasible in large hernia defects |
| --- | --- |
| Grade B | Patients should be informed that LVHR for large hernias compared to open repair results in fewer superficial SSI |
| Grade B | Patients should be informed that LVHR for large hernias compared to open repair results in less blood loss |
| Grade B | Patients should be informed that LVHR for large hernias compared to open repair results in shorter hospital stay |

This section evaluates the limitations of laparoscopic intraperitoneal onlay mesh repair. The two specific items researched were body habitus and defect size. The specific questions addressed are listed below.

**Measuring limitations of laparoscopic IPOM in terms of body habitus and defect size**

- What defect sizes have been described?
- What BMI levels have been described?
- Conversion rate?
- Complications?
- Comparisons with other patient cohorts

The statements made above are overall hampered by the paucity of studies with high quality study design. The majority of the studies encountered were retrospective in nature. Two were prospective studies, one was a cohort comparison and another a prospective cohort study. The remainders were retrospective studies out of which three were retrospective cohort comparisons. [1-21] Further, the definition of large hernia is very ill defined. There are classifications that do exist such as the classification from the European Hernia Society. Unfortunately, these classifications are not consistently used and definitions are often made for each study individually. Some studies consider large hernia >5 cm in diameter; some consider it >10 or 15 cm. In some studies, it is unclear in which dimension the hernia was measured; whether it was diameter versus length or width. One study referred to a hernia with size >20 cm as a giant hernia.

It is important to note that the level of recommendation in the statements and recommendations below for surgical site infection outcomes for laparoscopic ventral hernia repair versus open hernia repair in obese patients is extrapolated from consistent meta-analyses and randomized controlled trials for overall infection outcomes of laparoscopic versus open ventral hernia repairs.

**References**

1. Halm JA, de Wall LL, Steyerberg EW, Jeekel J, Lange JF (2007) Intraperitoneal polypropylene mesh hernia repair complicates subsequent abdominal surgery. World J Surg 31**:**423-429; discussion 430 **(level 4)**

2. Ferrari GC, Miranda A, Di Lernia S, Sansonna F, Magistro C, Maggioni D, Scandroglio I, Costanzi A, Franzetti M, Pugliese R (2008) Laparoscopic repair of incisional hernia: Outcomes of 100 consecutive cases comprising 25 wall defects larger than 15 cm. Surg Endosc 22**:**1173-1179 **(level 4)**

3. LeBlanc KA, Whitaker JM, Bellanger DE, Rhynes VK (2003) Laparoscopic incisional and ventral hernioplasty: lessons learned from 200 patients. Hernia 7**:**118-124 **(level 4)**

4. Perrone JM, Soper NJ, Eagon JC, Klingensmith ME, Aft RL, Frisella MM, Brunt LM (2005) Perioperative outcomes and complications of laparoscopic ventral hernia repair. Surgery 138**:**708-715; discussion 715-706 **(level 4)**

5. Varnell B, Bachman S, Quick J, Vitamvas M, Ramshaw B, Oleynikov D (2008) Morbidity associated with laparoscopic repair of suprapubic hernias. Am J Surg 196**:**983-987; discussion 987-988 **(level 4)**

6. Raftopoulos I, Vanuno D, Khorsand J, Ninos J, Kouraklis G, Lasky P (2002) Outcome of laparoscopic ventral hernia repair in correlation with obesity, type of hernia, and hernia size. J Laparoendosc Adv Surg Tech A 12**:**425-429 **(level 4)**

7. Raftopoulos I, Courcoulas AP (2007) Outcome of laparoscopic ventral hernia repair in morbidly obese patients with a body mass index exceeding 35 kg/m2. Surg Endosc 21**:**2293-2297 **(level 4)**

8. Novitsky YW, Cobb WS, Kercher KW, Matthews BD, Sing RF, Heniford BT (2006) Laparoscopic ventral hernia repair in obese patients: a new standard of care. Arch Surg 141**:**57-61 **(level 4)**

9. Muysoms F, Daeter E, Vander Mijnsbrugge G, Claeys D (2004) Laparoscopic intraperitoneal repair of incisional and ventral hernias. Acta Chir Belg 104**:**705-708 **(level 4)**

10. Gananadha S, Samra JS, Smith GS, Smith RC, Leibman S, Hugh TJ (2008) Laparoscopic ePTFE mesh repair of incisional and ventral hernias. ANZ J Surg 78**:**907-913 **(level 4)**

11. Ferrari GC, Miranda A, Sansonna F, Magistro C, Di Lernia S, Maggioni D, Franzetti M, Pugliese R (2008) Laparoscopic management of incisional hernias > or = 15 cm in diameter. Hernia 12**:**571-576 **(level 4)**

12. Carbajo MA, del Olmo JC, Blanco JI, de la Cuesta C, Martin F, Toledano M, Perna C, Vaquero C (2000) Laparoscopic treatment of ventral abdominal wall hernias: preliminary results in 100 patients. JSLS 4**:**141-145 **(level 4)**

13. Park A, Gagner M, Pomp A (1996) Laparoscopic repair of large incisional hernias. Surg Laparosc Endosc 6**:**123-128 **(level 4)**

14. Bower CE, Reade CC, Kirby LW, Roth JS (2004) Complications of laparoscopic incisional-ventral hernia repair: the experience of a single institution. Surg Endosc 18**:**672-675 **(level 4)**

15. Bamehriz F, Birch DW (2004) The feasibility of adopting laparoscopic incisional hernia repair in general surgery practice: early outcomes in an unselected series of patients. Surg Laparosc Endosc Percutan Tech 14**:**207-209 **(level 4)**

16. Birgisson G, Park AE, Mastrangelo MJ, Jr., Witzke DB, Chu UB (2001) Obesity and laparoscopic repair of ventral hernias. Surg Endosc 15**:**1419-1422 **(level 4)**

17. Kurmann A, Visth E, Candinas D, Beldi G (2011) Long-term follow-up of open and laparoscopic repair of large incisional hernias. World J Surg 35**:**297-301 **(3)**

18. Ballem N, Parikh R, Berber E, Siperstein A (2008) Laparoscopic versus open ventral hernia repairs: 5 year recurrence rates. Surg Endosc 22**:**1935-1940 **(level 4)**

19. Raftopoulos I, Vanuno D, Khorsand J, Kouraklis G, Lasky P (2003) Comparison of open and laparoscopic prosthetic repair of large ventral hernias. JSLS 7**:**227-232 **(level 3)**

20. Gonzalez R, Rehnke RD, Ramaswamy A, Smith CD, Clarke JM, Ramshaw BJ (2005) Components separation technique and laparoscopic approach: a review of two evolving strategies for ventral hernia repair. Am Surg 71**:**598-605 **(level 3)**

21. Muller-Riemenschneider F, Roll S, Friedrich M, Zieren J, Reinhold T, von der Schulenburg JM, Greiner W, Willich SN (2007) Medical effectiveness and safety of conventional compared to laparoscopic incisional hernia repair: a systematic review. Surg Endosc 21**:**2127-2136 **(level 2A)**

**Obese Patient and Incisional Hernia**

F. Köckerling, P. Chowbey

Search terms: "Incisional Hernia"; "Ventral Hernia"; "Incisional Hernia and Obesity"; "Ventral Hernia and Obesity"; "Laparoscopic Incisional Hernia Repair"; "Laparoscopic Ventral Hernia Repair (LVHR)"; LVHR and Obesity"; LVHR and Complications"; LVHR and Wound Infections"; LVHR and Defect Size"

A systematic search of the available literature was performed in July 2012 using Medline, PubMed, Cochrane library and relevant journals and reference lists using the above listed search terms.

The first search detected 35 relevant articles. In a second - level search no article was added. In Summery 9 articles and studies were used for this review.

**Key question:**

**What is Better, Open or Laparoscopic Approach?**

**Statements**

| | Level 1A  Level 2A  Level 2B  Level 3 | Laparoscopic ventral and incisional hernia repair is associated with fewer wound infections.  Laparoscopic ventral and incisional hernia repair is associated with significantly fewer wound complications.  Obese patients (BMI>30) have significantly larger defect sizes in laparoscopic incisional hernia repair.  A body mass index (BMI) >30 and/or a defect size greater than 8-10 cm lead significantly more often to a recurrence.  No significant differences were noted in terms of early outcome of laparoscopic ventral hernia repair between non-morbidly obese (BMI<35) and morbidly obese (BMI≥35) patients | | --- | --- | |
| --- | --- | --- |

**Recommendations**

| Grade A  Grade B | In obese patients presenting a ventral or incisional hernia, in order to reduce the rate of wound infections and complications the laparoscopic approach must be preferred.  In patients with a BMI ≥ 35 laparoscopic ventral and incisional hernia repair may be preferred.  In obese patients the defect sizes are significantly greater, something that has to be considered when indicating the laparoscopic approach.  In obese patients (BMI≥30) with a defect size greater than 8-10 cm, there may be a need for additional technical steps (greater mesh fixation, more overlap, suture closure of the defect), when the laparoscopic approach is indicated. |
| --- | --- |

Obesity is a risk factor for occurrence of incisional hernias and leads to a higher perioperative complication rate and a higher recurrence rate after open repair. There are multifactorial reasons for this, such as delayed wound healing, impaired pulmonary function and higher intraabdominal pressure (Birgisson et al 2001).

Meta-analyses of prospective randomized studies, which compared laparoscopic repair of incisional and ventral hernias with open repair, showed a significantly lower rate of wound infections, with no removal of the mesh, for the laparoscopic IPOM technique (Level 1A) and a trend towards lower infection rates with mesh removal (Level 1A) likewise for the minimally invasive technique (Forbes et al. 2009). In the metaanaIysis of Sauerland et al. (2011) the local infection rate in the laparoscopic group was 3,1 % versus 13,4 % in the open group (p < 0,00001). A local infection requiring mesh removal was found in 0,7 % in the laparoscopic and 3,5 % in the open group (p = 0,09). In an analysis of pooled data on 4,582 laparoscopic and 758 open repairs of incisional and ventral hernias, Pierce et al. (2007) found a wound complication rate of 3.8 % for the laparoscopic, and 16.8 % for the open, technique (p<0.0001) (Level 2A).

The significantly lower rate of wound complications attests to the benefits of using the minimally invasive technique, especially for obese persons, who in general are at higher risk for wound complications. In a metaanalysis of cohort studies, Mavros et al. (2011) observed a trend toward higher mesh infection rates in obese patients following open ventral hernia repair.

However, a larger abdominal wall defect must be expected in obese patients with an incisional hernia. In a study by Moreno-Egea et al. (2012) it was possible to demonstrate that in patients having a body mass index (BMI) >30, the proportion of defect sizes <10 cm was 35.1 %. But 60% of the patients with a defect size of 10-12 cm showed a BMI >30 , and in patients with defects > 12 cm this percentage was 73.5 % (Level 2B). Accordingly, a larger defect for an incisional hernia must always be expected in obese persons. In a mean follow-up of 5 years, following laparoscopic IPOM repair of incisional hernias defects < 10 cm recurrences were seen in 0.4 %, for defects of 10-12 cm in 20 %, and for defects > 12 cm in 41.2 % (Moreno-Egea et al. 2012). Accordingly, significant differences were noted in the defect sizes, in body mass index and in the proportion of patients with a BMI>30 between the recurrence group and the non-recurrence group. In the recurrence group the mean BMI was 36.3 ± 6.3, while in the non-recurrence group it was 29.5 ± 5.9 (p<0.001). The proportion of patients with a BMI>30 was 90 % in the recurrence group and 37.9 % in the non-recurrence group (p<0.001). The mean defect size was 14.4 ± 2.9 cm in the recurrence group and 7.9 ± 2.9 cm in the non-recurrence group (p<0.001).

As such, it must be noted that patients with a BMI>30 have significantly greater defects in the case of incisional hernias and that recurrences are significantly more common among these patients for a defect size of more than 8-10 cm. Accordingly, additional technical steps are needed to prevent recurrence, such as the use of a larger mesh to assure more extensive mesh overlap and stronger fixation of the mesh or even suture closure of the defect.

On comparing early postoperative outcome of patients with a BMI < 35 and those with a BMI of ≥ 35 (Level 3), no significant differences were discerned in the rate of enterotomies, haematomas, seromas, enterocutaneous fistulas or postoperative infections (Ching et al. 2008).

In 163 patients with a BMI > 30, Novitsky et al. (2006) found a mortality rate of 0 % after laparoscopic repair of incisional and ventral hernias, a conversion rate of 3.1 %, a postoperative complication rate of 12.3 %, a wound infection rate of 1.2 % and a mesh-related infection rate of 1.2 %. Raftopoulos et al. (2007) identified for patients with a BMI ≥ 35 likewise a mortality rate of 0 %, a wound infection rate of 3.7 %, a bladder injury rate of 3.7 % and a postoperative impaired passage rate of 11.1 %.

**References**

1. Birgisson G, Park AE, Mastrangelo MJ, Witzke DB, Chu UB

Obesity and laparoscopic repair of ventral hernias.

Surg Endosc (2001) 15:1419-1422 **(level 2 B)**

2. Forbes SS, Eskicioglu C, McLeod RS, Okrainec A

Meta-analysis of randomized controlled trials comparing open and laparoscopic ventral and

incisional hernia repair with mesh. British Journal of Surgery (2009) 96:851-858 **(level 1 A)**

3. Sauerland S, Walgenbach M, Habermalz B, Seiler CM, Miserez M

Laparoscopic versus open surgical techniques for ventral or incisional hernia repair (Review)

The Cochrane Collaboration, Published by John Wiley & Sons, Ltd. (2011**)(level Ia)**

4. Pierce RA, Spitler JA, Frisella MM, Matthews BD, Brunt LM

Pooled data analysis of laparoscopic vs. open ventral hernia repair: 14 years of patient data

accrual. Surg Endosc (2007) 21:378-386 **(level 2 A)**

5. Mavros MN, Athanasious S, Alexiou VG, Mitsikostas PK, Peppas G, Falagas ME

Risk Factors for Mesh-related Infections After Hernia Repair Surgery: A Meta-analysis of

Cohort Studies. World J Surg (2011) 35:2389-2398 **(level Ia)**

6. Moreno-Egea A, Carrillo-Alcaraz A, Aguayo-Albasini JL

Is the outcome of laparoscopic incisional hernia repair affected by defect size? A prospective

study. Am J Surg (2012), Jan; 203 (1): 87-94. Epub 2011 Jul 23 **(level 2 B)**

7. Ching SS, Sarela AI, Dexter SPL, Hayden JD, McMahon MJ

Comparison of early outcomes for laparoscopic ventral hernia repair between nonobese and

morbidly obese patient populations. Surg Endosc (2008) 22:2244-2250 **(level 3)**

8. Novitsky YW, Cobb WS, Kercher KW, Matthews BD, Sing RJ, Heniford BT

Laparoscopic Ventral Hernia Repair in Obese Patients.

Arch Surg (2006) 141:57-61 **(level 4)**

9. Raftopoulos, I, Courcoulas AP Outcome of laparoscopic ventral hernia repair in morbidly

obese patients with a body mass index excreeding 35 kg/m^2^. Surg Endosc (2007) 21:2293-

2297 **(level 4)**

**Recurrence after open surgery - re-do better laparoscopically?**

R Schrittwieser

Pubmed search

Search terms: (open[All Fields] AND ("hernia, ventral"[MeSH Terms] OR ("hernia"[All Fields] AND "ventral"[All Fields]) OR "ventral hernia"[All Fields] OR ("ventral"[All Fields] AND "hernia"[All Fields])AND ("recurrence"[MeSH Terms] OR "recurrence"[All Fields])

The search was performed in August 2011.

The first search detected 270 articles. For the review only 5 articles could be used.

**Key question:**

**Should a reoperation in case of a recurrent hernia after open surgery better be done laparoscopically?**

**Statement**

| Level 4 | There is some evidence if in case of a recurrence after open surgery the reoperation is done laparoscopically occult hernias can be detected. |
| --- | --- |

Recommendation

| Grade C | In some cases of recurrence after open repair the reoperation should better be done laparoscopically in presence of sufficient experience in laparoscopic ventral hernia repair. |
| --- | --- |

Reoperations are challenging interventions when reoccurrences appear following treatment of ventral hernias. In order to answer the question whether surgery on recurring conditions should be better carried out by an open operation or laparoscopically, there are currently no evidence based recommendations. In principle however it needs to be differentiated, whether or not a mesh was inserted during the primary intervention. In cases of suture repair the indication whether to opt for open or laparoscopic surgery is similar to that for the primary incisional hernia. The advantages and disadvantages of laparoscopic repair are described in numerous reviews. (1) (2) (3) When initially a mesh implant takes place then laparoscopic reoperation, if it is possible, does have some advantages. First of all the repeat operation is carried out at a different level of the abdominal wall, and furthermore in all cases the whole incisional scar can be covered by a mesh. In addition it is on the whole not necessary to remove the previously inserted mesh, whereby an expanded dissectionof the abdominal wall can be avoided.

Uranues et al (4) were able to highlight, that with sufficient expertise, laparoscopic reoperation can also be carried out following multiple preliminary operations with reasonable certainty and moderate recurrence rates.

A possible advantage of laparoscopic reoperation is the identification of previously undiscovered recurring hernias, which can immediately be taken care of during laparoscopic repair. Sharma et al (5) found 203 (16.3%) occult hernias amongst their patient sample of 1242 laparoscopic ventral hernia repair over 13 years.

**References:**

(1) [Sauerland S](http://www.ncbi.nlm.nih.gov/pubmed?term=Sauerland%20S%5BAuthor%5D&cauthor=true&cauthor_uid=21412910), [Walgenbach M](http://www.ncbi.nlm.nih.gov/pubmed?term=Walgenbach%20M%5BAuthor%5D&cauthor=true&cauthor_uid=21412910), [Habermalz B](http://www.ncbi.nlm.nih.gov/pubmed?term=Habermalz%20B%5BAuthor%5D&cauthor=true&cauthor_uid=21412910), [Seiler CM](http://www.ncbi.nlm.nih.gov/pubmed?term=Seiler%20CM%5BAuthor%5D&cauthor=true&cauthor_uid=21412910), [Miserez M](http://www.ncbi.nlm.nih.gov/pubmed?term=Miserez%20M%5BAuthor%5D&cauthor=true&cauthor_uid=21412910).Laparoscopic versus open surgical techniques for ventral or incisional hernia repair. Cochrane Database Syst Rev. 2011 Mar 16;(3):CD007781.**(level 1a)**

(2) [Müller-Riemenschneider F](http://www.ncbi.nlm.nih.gov/pubmed?term=M%C3%BCller-Riemenschneider%20F%5BAuthor%5D&cauthor=true&cauthor_uid=17763905), [Roll S](http://www.ncbi.nlm.nih.gov/pubmed?term=Roll%20S%5BAuthor%5D&cauthor=true&cauthor_uid=17763905), [Friedrich M](http://www.ncbi.nlm.nih.gov/pubmed?term=Friedrich%20M%5BAuthor%5D&cauthor=true&cauthor_uid=17763905), [Zieren J](http://www.ncbi.nlm.nih.gov/pubmed?term=Zieren%20J%5BAuthor%5D&cauthor=true&cauthor_uid=17763905), [Reinhold T](http://www.ncbi.nlm.nih.gov/pubmed?term=Reinhold%20T%5BAuthor%5D&cauthor=true&cauthor_uid=17763905), [von der Schulenburg JM](http://www.ncbi.nlm.nih.gov/pubmed?term=von%20der%20Schulenburg%20JM%5BAuthor%5D&cauthor=true&cauthor_uid=17763905), [Greiner W](http://www.ncbi.nlm.nih.gov/pubmed?term=Greiner%20W%5BAuthor%5D&cauthor=true&cauthor_uid=17763905), [Willich SN](http://www.ncbi.nlm.nih.gov/pubmed?term=Willich%20SN%5BAuthor%5D&cauthor=true&cauthor_uid=17763905).Medical effectiveness and safety of conventional compared to laparoscopic incisional hernia repair: a systematic review. Surg Endosc. 2007 Dec;21(12):2127-36. Epub 2007 Sep 1.**(level 1a)**

(3) [LeBlanc KA](http://www.ncbi.nlm.nih.gov/pubmed?term=LeBlanc%20KA%5BAuthor%5D&cauthor=true&cauthor_uid=18237502), [Elieson MJ](http://www.ncbi.nlm.nih.gov/pubmed?term=Elieson%20MJ%5BAuthor%5D&cauthor=true&cauthor_uid=18237502), [Corder JM 3rd](http://www.ncbi.nlm.nih.gov/pubmed?term=Corder%20JM%203rd%5BAuthor%5D&cauthor=true&cauthor_uid=18237502). Enterotomy and mortality rates of laparoscopic incisional and ventral hernia repair: a review of the literature. JSLS. 2007 Oct-Dec;11(4):408-14.**(level 1a)**

(4) [Uranues S](http://www.ncbi.nlm.nih.gov/pubmed?term=Uranues%20S%5BAuthor%5D&cauthor=true&cauthor_uid=18954777), [Salehi B](http://www.ncbi.nlm.nih.gov/pubmed?term=Salehi%20B%5BAuthor%5D&cauthor=true&cauthor_uid=18954777), [Bergamaschi R](http://www.ncbi.nlm.nih.gov/pubmed?term=Bergamaschi%20R%5BAuthor%5D&cauthor=true&cauthor_uid=18954777).Adverse events, quality of life, and recurrence rates after laparoscopic adhesiolysis and recurrent incisional hernia mesh repair in patients with previous failed repairs. J Am Coll Surg. 2008 Nov;207(5):663-9. Epub 2008 Aug 9.**(level 4)**

(5) [Sharma A](http://www.ncbi.nlm.nih.gov/pubmed?term=Sharma%20A%5BAuthor%5D&cauthor=true&cauthor_uid=21082208), [Mehrotra M](http://www.ncbi.nlm.nih.gov/pubmed?term=Mehrotra%20M%5BAuthor%5D&cauthor=true&cauthor_uid=21082208), [Khullar R](http://www.ncbi.nlm.nih.gov/pubmed?term=Khullar%20R%5BAuthor%5D&cauthor=true&cauthor_uid=21082208), [Soni V](http://www.ncbi.nlm.nih.gov/pubmed?term=Soni%20V%5BAuthor%5D&cauthor=true&cauthor_uid=21082208), [Baijal M](http://www.ncbi.nlm.nih.gov/pubmed?term=Baijal%20M%5BAuthor%5D&cauthor=true&cauthor_uid=21082208), [Chowbey PK](http://www.ncbi.nlm.nih.gov/pubmed?term=Chowbey%20PK%5BAuthor%5D&cauthor=true&cauthor_uid=21082208)Laparoscopic ventral/incisional hernia repair: a single centre experience of 1,242 patients over a period of 13 years.Hernia. 2011 Apr;15(2):131-9. Epub 2010 Nov 17.**(level 4)**

**Section 3: Perioperative Management**

**What is the evidence for antibiotic and thromboembolic prophylaxis**

**in laparoscopic ventral hernia surgery?**

Rudolf Schrittwieser

Is antibiotic prophylaxis routinely indicated for an elective laparoscopic ventral hernia operation? Is thromboembolic prophylaxis routinely indicated for an elective laparoscopic ventral hernia operation?

Search terms: „ ventral hernia“ AND „antibiotic prophylaxis“„ventral hernia“ AND „antibiotic prophylaxis“ AND „laparoscopy“„ventral hernia“ AND „antibiotic prophylaxis“ AND „randomized studies“„abdominal wall hernia“ AND „antibiotic prophylaxis“ „ ventral hernia“ AND „thromboembolic prophylaxis“„ hernia“ AND „ thromboembolic prophylaxis“ AND „laparoscopy“„ventral hernia“ AND „ thromboembolic prophylaxis“ AND „randomized studies“„abdominal wall hernia“ AND „ thromboembolic prophylaxis“

The search was performed in August 2011.

The first search detected 24 articles and there remained 13 articles which were used for this review.

**Key questions:**

**Is antibiotic prophylaxis routinely indicated for an elective laparoscopic ventral hernia operation?**

**Is thromboembolic prophylaxis routinely indicated for an elective laparoscopic ventral hernia operation?**

**Statements:**

| Level 2b | Antibiotic prophylaxis in ventral hernia repair is associated with significantly less local infections. |
| --- | --- |
| Level 5 | There is insufficient evidence for routine thrombembolic prophylaxis in laparoscopic ventral hernia repair |

**Recommendations:**

| Grade B | Routine antibiotic prophylaxis in ventral hernia repair is recommended. |
| --- | --- |
| Grade D | It is recommended that thromboembolic prophylaxis be given according to usual routines in patients with risk factors. |

**Antibiotic Prophylaxis:**

Antibiotic prophylaxis in relation to hernia surgery is a continuous a topic of discussion. As far as the inguinal hernia is concerned both grade D recommendations (1) and grade B recommendations (2) could be applied to laparoscopic inguinal hernia surgery to date. However in general it can be said that there is significantly more literature available concerning inguinal hernia surgery than there is concerning the ventral hernia. In fact there are very few relevant studies in existence that deal specifically with laparoscopic surgery of the ventral hernia and antibiotic prophylaxis.

The rate of infection with LVHR in specific studies can be as high as 16%, but normally is around 0,5to 4% (9).

2 studies are available at the level 2b.

Rios et al (3) were able to show, in a study published in 2001, a significant difference between with and without prophylactic antibiotics (P-value 0.00991). It dealt however with a non-randomised investigation of patients who had undergone open repair through mesh implantation, in which the patient groups differed in size (140 with prophylaxis, 76 without prophylaxis) and the rate of infection of 18.1% seemed overall somewhat on the high side.

Abromov et al (4) concluded from their investigation that single dose antibiotic prophylaxis has a positive effect on the wound infection rate after umbilical and incisional hernia operations. Also here it dealt with open hernia operations. The investigation was indeed conceived as a randomised controlled trial, however with a total of 35 patients it clearly lacks in impact. 1g of Cefonicid was given intravenously to every second patient 30 minutes before the operation. The wound infection rate was 1 out of 17 in the antibiotic prophylaxis group in comparison to 8 out of 18 in the non-antibiotic prophylaxis group, although once again the rate of infection within the non-antibiotic prophylaxis group does appear high.

3 studies are available at level 4.

White et al (5) investigated 250 hernia operations on 206 patients over a period of 14 years in terms of the wound complication rate and the influence of antibiotic prophylaxis, drainage and mesh implantation. Neither antibiotics nor drainage had any influence on the rate of wound complication.

Deysine et al (6) established, by means of a retrospective investigation of their own patients, an infection rate of 0.11% in more than 4,000 inguinal and 350 clean ventral hernia operations. Alongside antibiotic prophylaxis with 1g of Cefazolin being given intravenously one hour before the operation the approach comprised of additional frequent intraoperative wound flushing with a solution of 80mg Gentamicin in 250ml NaCl.

A further study by Edwards et al (7) in 2005 retrospectively investigated 65 cases where laparoscopic ventral hernia repair had been carried out in order to establish the rate of seroma associated cellulitis. Prior to surgery all of the patients had received a prophylactic antibiotic with a third generation cephalosporin, however in addition 45 of the 65 patients received 7 days after the operation either cephalosporin or fluoroquinolone orally over 7 days. The seroma rate amounted to a total of 33% in the post-surgery prophylactic antibiotic group and 30% in the pre-surgery only group, however in the pre-surgery only group 100% of the patients developed seroma associated cellulitis, resulting in the need to explant 2 meshes. On the other hand seroma associated cellulitis developed in only 40% of the post-surgery prophylactic antibiotic group. No side effects were observed as a result of taking the antibiotics. The authors concluded from this that taking the antibiotics for 7 days represented an effective means of reducing the seroma associated cellulitis in connection with laparoscopic ventral hernia repair. However the 100% cellulitis rate appears very high

once again within the pre-surgery only group and is questionable, and furthermore the study dealt with a small and very heterogeneous sample of patients.

Further studies concerning laparoscopic ventral hernia surgery can be identified, in which the methods used indicate the routine application of a prophylactic antibiotic. These range from the administration of Amoxicillin (1g) and Clavulanic acid (200mg) before surgery and 8 hours after the operation (8), the administration of a second generation cephalosporin at the start of the anaesthesia and 24 hours after the operation (10) to the administration of a first generation cephalosporin at the time of the skin incision and then repeated for operations lasting longer than 2 hours (11).

From the studies available a clear recommendation for or against the use of antibiotic prophylaxis cannot be drawn. It appears advisable however, along the lines of the recommendations for laparoscopic inguinal hernia repair, to consider administering a prophylactic antibiotic in the case of patients with risk factors (advanced age, administration of corticosteroids, immunosuppressive therapy, obesity, diabetes and malignant tumour) as well as in cases with surgical complications (contamination, long operation duration, drainage, urinary catheter). Furthermore with LVHR the intraperitoneal state of the mesh needs to be considered and therefore the possible use of a prophylactic antibiotic.

**Thromboembolic Prophylaxis**

Thromboembolic occurences represent serious complications within the context of surgical intervention in the abdomen. In addition some studies seem to suggest a higher risk within the context of laparoscopic interventions (12). The increased intraperitoneal pressure and the reversed Trendelenburg position possibly cause this.

There no randomised controlled trials available concerning the area of thrombosis prophylaxis in connection with LVHR.

In terms of thromboembolic prophylaxis and the incidences of thromboembolic complications following laparoscopic surgery, a prospective investigation was carried out (13). From a total of 2,384 patients 8 cases of deep vein thrombosis (DVT) were recorded, however there were no cases of pulmonary embolism. Of these in 6 cases pneumoperitoneum lastedfor more than 2 hours and in 2 cases for more than 3 hours. The authors concluded from their investigation that the heparin prophylaxis should be continued at least until discharge. Furthermore compression stockings are recommended for reduced intra-abdominal pressure, for occasional release of pneumoperitoneum and for a possible short–term anti-Trendelenburg position.

**References**

1 .Bittner R, Arregui ME, Bisgaard T,Dudai M, Ferzli GS,Fitzgibbons RJ, Fortelny RH, Klinge U, Kockerling F, Kuhry E, Kukleta J, Lomanto D,Misra MC,Montgomery A,Morales-Conde S, Reinpold W, Rosenberg J, Sauerland S,Schug-Paß C, Singh K, Timoney M, Weyhe D, Chowbey P. Guidelines for laparoscopic (TAPP) and endoscopic (TEP) treatment of inguinal Hernia [International Endohernia Society (IEHS)] Surg. Endosc. 2011 Sep;25(9):2773-843**(level 1a)**

2 .Simons MP, AufenackerT, Bay-Nielsen M, Bouillot JL,CampanelliG, Conze J, de Lange D, Fortelny R, Heikkinen T, Kingsnorth A, Kukleta J, Morales-Conde S, Nordin P, Schumpelick V, Smedberg S, Smietanski M, Weber G, Miserez M. European Hernia Society guidelines on the treatmentof inguinal hernia in adult patients. Hernia 2009 Aug; 13(4):343-403**(level 1a)**

3 .Ríos A, Rodríguez JM, Munitiz V, Alcaraz P, Pérez Flores D, Parrilla P. [Antibiotic prophylaxis in incisional hernia repair using a prosthesis.](http://www.ncbi.nlm.nih.gov/pubmed/11759801)Hernia. 2001 Sep;5(3):148-52.**(level 2b)**

4.Abramov D, Jeroukhimov I, Yinnon AM, Abramov Y, Avissar E, Jerasy Z, Lernau O. [Antibiotic prophylaxis in incisional hernia repair using a prosthesis.](http://www.ncbi.nlm.nih.gov/pubmed/11759801)

Eur J Surg. 1996 Dec;162(12):945-8; discussion 949.**(level 2b)**

5 .Edwards C, Angstadt J, Whipple O, Grau R. [Laparoscopic ventral hernia repair: postoperative antibiotics decrease incidence of seroma-related cellulitis.](http://www.ncbi.nlm.nih.gov/pubmed/16372611)

Am Surg. 2005 Nov;71(11):931-5; discussion 935-6.**(level 4)**

6.White TJ, Santos MC, Thompson JS. [Factors affecting wound complications in repair of ventral hernias.](http://www.ncbi.nlm.nih.gov/pubmed/9520825)Am Surg. 1998 Mar;64(3):276-80.**(level 4)**

7 .Deysine M. [Postmesh herniorrhaphy wound infections: can they be eliminated?](http://www.ncbi.nlm.nih.gov/pubmed/16463947)

Int Surg. 2005 Jul-Aug;90(3 Suppl):S40-4. Review.**(level4)**

8 .Bansal VK, Misra MC, Kumar S, Rao K, Singhal P, Goswami A,
Guleria S, Arora MK, Chabra A. A prospective randomized study comparing suture mesh fixation versus tacker mesh fixation for laparoscopic repair of incisional and ventral hernias.Surg Endosc 2011:25:1431–1438**(level 1b)**

9 . LeBlanc KA. Laparoscopic incisional and ventral hernia repair: Complications—how to avoid and handle Hernia 2004: 8: 323–331**(level 4)**

10 .Bellows CF, Berger DH. Infiltration of Suture Sites With Local Anesthesia for Management of Pain Following Laparoscopic Ventral Hernia Repairs: a Prospective Randomized TrialJSLS 2006:10:345–350**(level 2b)**

11 . Heniford T, Park A, Ramshaw B, MD, Voeller G. Laparoscopic Repair of Ventral Hernias Nine Years’ Experience With 850 Consecutive Hernias Ann Surg 2003;238: 391–400**(level 4)**

12. [Holzheimer RG](http://www.ncbi.nlm.nih.gov/pubmed?term=%22Holzheimer%20RG%22%5BAuthor%5D)**.** Laparoscopic procedures as a risk factor of deep venous thrombosis, superficial ascending thrombophlebitis and pulmonary embolism--case report and review of the literature.Eur J Med Res. 2004 Sep 29;9(9):417-22.**(level 4)**

13.[Catheline JM](http://www.ncbi.nlm.nih.gov/pubmed?term=%22Catheline%20JM%22%5BAuthor%5D), [Capelluto E](http://www.ncbi.nlm.nih.gov/pubmed?term=%22Capelluto%20E%22%5BAuthor%5D), [Gaillard JL](http://www.ncbi.nlm.nih.gov/pubmed?term=%22Gaillard%20JL%22%5BAuthor%5D), [Turner R](http://www.ncbi.nlm.nih.gov/pubmed?term=%22Turner%20R%22%5BAuthor%5D), [Champault G](http://www.ncbi.nlm.nih.gov/pubmed?term=%22Champault%20G%22%5BAuthor%5D).

Thromboembolism prophylaxis and incidence of thromboembolic complications after laparoscopic surgery.Int J Surg Investig. 2000;2(1):41-7.**(level 4)**

**Section 4: Key-points of technique**

**Positioning of the trocars and creating the capnopneumoperitoneum.**

Rohr, M. Trommer, Y

*Search terms : “laparoscopic hernia repair“ AND “LVHR“ AND “incisional hernia“ AND “ventral hernia“ AND “capno/peritoneum“ AND “trocar position“ AND “laparoscopic insufflation“ AND “CO^2n^ insufflations laparoscopic“*

A systemic search of the available literature was performed in August 2011 using Medline, PubMed, Cochrane library and relevant journals and reference lists using the above listed search terms

The search detected 15 relevant articles.

**Key questions:**

**Best way of trocar position? Best way of CO^2^ -insufflation?**

**Statements**

| Level 4 | A safe area for Veress needle insertion is usually in the right or left upper quadrant, but most surgeons prefer an open access (Hasson) in the left or right subcostal region in dependence on the previous operation and expected adhesions.  The location of the trocars will be influenced by the location of the hernia defect(s).  30°/45° scopes provide a better view to the inner part of the abdominal wall. |
| --- | --- |

**Recommendation**

| Grade D | It is considered to choose the left or right upper quadrant subcostally for the first access to the abdominal cavity.  It is considered to use a 30 degree angled laparoscope.  In dependence on the adhesions found inside as well as the size, site and number of existing wall defects the trocar entry points should be as far as possible to achieve triangulation of the site of the hernia. |
| --- | --- |

***Background:***

As incisional hernia is a frequent complication of abdominal surgery. To standardize the surgical techniques this article concentrates on content literature review concerning the optimal trocar position and preparation to create the capnopneumoperitoneum in the beginning of the operation.

***Methods:***

Systematic research of current guidelines, articles, reviews and case reports concerning laparoscopic treatment and positioning of trocars as well as creating the capnopneumoperitoneum.

**Results:**

Even though there is a large number of studies concerning ventral hernia management and surgical techniques, there are no articles to be found concerning only for preparing the capnopneumoperitoneum. There are merely a few studies investigating the technique for creation of progressive pneumoperitoneum in hernia patients presenting with a loss of domain [1].

But many articles can be found concerning the positions of the insertions of the trocars [2], however, most authors describe only their personal technique and recommend insertion of the trocars[3] in dependence on the adhesions found inside as well as the size, site and number of existing wall defects (2,4). A 3-trocar technique with primary a 10-12mm-trocar and then - depending on the intraabdominal anatomical situation - additional followed by one or two 5- or 10mm-trocars is mostly preferred [5,6]. Those can as well be positioned following the subcostal line on the left passing the rectus muscle or on the right side [7,8]. It is frequently necessary to place and manipulate instruments from the side of the patient in direct opposition to the viewing laparoscope to produce a mirror image for allowing a better viewing of all the adhesions(2). Moreover, an opposite 5mm trocar may provide a better fixation of the parts of the mesh near the optic trocar (9). Only in few cases in spite of the left subcostal area a subumbilical insertion is chosen, but there are no reliable results to generalize this decision.

The use of a 30°-scope is described because it seems to provide a good view of the inner part of the abdominal wall (2).

In contrast to groin hernia operations, in most of the patients the capnopneumoperitoneum is not created by using a Veress needle [10,11]. The left subcostal position is used to insert the first – mostly 10-12mm - trocar in open technique(Hasson) and to insufflate CO_2_ until a pressure of 12 to 14 mm Hg is reached [12,13]. When later the mesh is inserted into the abdomen, the pneumoperitoneum is reduced to 9 mmHg until it is fixed by suture, and to put in the tacks it will be increased onto 12 to 14 mmHg again [14].


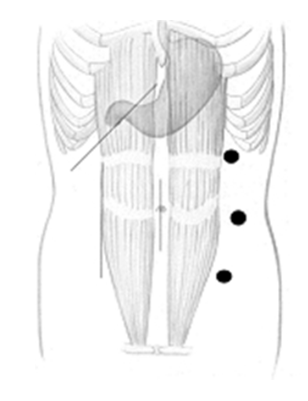

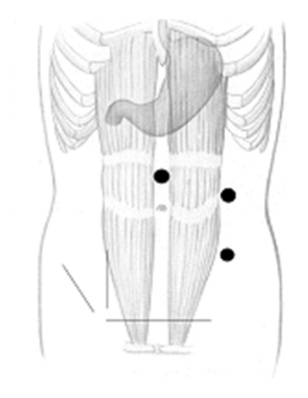


**References:**

1. Mcadory RS, Cobb WS,, Carbonell AM.; Progressive preoperative pneumoperitoneum for hernias with loss of domain: Am Surg 2009 Jun;75(6):508-9 **(level 4)**
2. LeBlanc K. Herniorrhaphy with the use of transfascial sutures. In Laparoscopic Hernia Surgery. Ed. K. LeBlanc , Arnold Publisher, London 2003, pp 115-124.
3. Olmi S., Magnone S., Erba L., Bertolini A., Croce E.; Results of Laparoscopic Versus Open Abdominal and Incisional Hernia Repair: JSLS 2005 Apr-Jun; 9(2): 189-195 **(level 4)**
4. Köckerling F, Schneider C, Rexmond MA, Scheidbach H, Konradt J, Bärlehner E, Bruch HP, Kuthe A, Troidl H, Hohenberger W.; Early results of a prospective multicenter study on 500 consecutive cases of laparoscopic colorectal surgery. Surg Endosc (1998) 12:37-41 **(level 4)**
5. Heniford BT, Park A, Ramshaw BJ, Voeller G.; Laparoscopic Ventral and Incisional Hernia Repair in 407 Patient: J Am Coll Surg 2000 **(level 4)**
6. Heniford BT, Park A, Ramshaw BJ, Voeller G.; Laparoscopic Ventral and Incisional Hernia Repair in 407 Patient: J Am Coll Surg 2000 **(level 4)**
7. Moreno-Egea A, Carillo-Alcaraz A.; Management of non-midline incisional hernia by the laparoscopic approach: results of a long-term follow-up prospective study: Surg Endosc (2012) 26:1069-1078 **(level 4)**
8. Baghai M, Ramshaw BJ, Smith CD, Fearing N, Bachmann S, Ramaswamy A.; Techniques of laparoscopic ventral hernia repair can be modified to successfully repair large defects in patients with loss of domain: Surg Innov 2009 Mar; 16(1):38-45 **(level4)**
9. Berger D. Laparoskopische Hernienoperationen. In Hernienchirurgie. Edt. Obermaier A, F. Pfeffer, U.T. Hopt. Urban & Fischer München 2009, pp 152-156.
10. Vettoretto N, Carrara A, Corradi A, De Vivo G, Lazzaro L, Ricciardelli L, Agresta F, Amodio C et al; Laparoscopic Adhesiolysis: Consensus Conference: Colorectal Dis. 2012 May; 14(5):e208-15 **(level 4)**
11. Nardi MJ, Millo P, Brachet Contul R, Fabozzi M, Persico F, Roveroni M, Laie Murix E, Bocchia P, Lorusso R, Gatti A, Grivon M, Allieta R.; Laparoscopic incisional and ventral hernia repair (LIVHR) with Parietex Composite mesh: Minimally Invasive Therapy 2012; 21:173-180 **(level 4)**
12. Tsai HW, Chen YJ, Ho CM, Hseu SS, Chao KC, Tsai SK, Wang PH. ; Maneuvers to decrease laparoscopy-induced shoulder and upper abdominal pain: a randomized controlles study: Arch Surg 2011 Dec; 146(12):1360-6 **(level 4)**
13. Zhu Q, Mao Z, Yu B, Jin J, Zheng M, Li J.; Effects of persistent CO_2_-insufflation during different laparoscopic inguinal hernioplasty: a prospective, randomized, controlled study: J Laparoendosc Adv Surg Tech A 2009 Oct; 19(5):611-4 **(level 4)**
14. Ghaderi I, Vaillancourt M, Sroka G, Kaneva PA, Vassiliou MC, Choy I, Okrainec A, Seagull FJ, Sutton E, George I, Park A, Brintzenhoff R, Stefanidis D, Fried GM, Feldmann LS.; Evaluation of surgical performance during laparoscopic incisional hernia repair: A multicenter study: Surg Endosc (2011) 25:2555-2563 **(level 4)**

**Port type, positions, and number in laparoscopic ventral hernia repair**

Rim, Sean; Yakoub, Danny; Ferzli, George

*Search Terms: “Laparoscopic” AND “ventral” AND “incisional” AND “abdominal wall” AND “hernia” AND “technique”*

A systematic search of the literature was performed in January 2012 using PubMed, Cochrane library and reference lists.

58 articles were found and analyzed, 4 were added. 6 articles were used for this review.

**Key questions:**

**-What types of trocars are available?**

**-What is the optimal position and number of trocars?**

**Statements:**

| **Level 2** | Visual entry trocars can minimize the size of the entry wound but do not decrease the incidence of visceral or vascular injury. |
| --- | --- |
| **Level 4** | Placement of trocars is dictated by the size and location of the defect.  Placement of additional trocars may be necessary. |

**Recommendations:**

| **Grade B** | Visual entry trocars should be utilized when attempting to decrease the size of the wound. |
| --- | --- |
| **Grade D** | The surgeon should utilize three inline ports on the left side when dealing with defects involving the midline or right side of the abdomen.  Left sided defects should be approached with three inline ports on the right.  The superior or inferior ports can be moved closer to midline depending on the location of the hernia.  When adding additional trocars, the principles of triangulation and maintenance of optimal distance should be held. |

**Introduction:**

Laparoscopic repair of ventral hernias is becoming the preferred approach with more and more literature confirming decreased morbidity and faster time to recovery. As in the traditional open approach, the keys components to the repair include tension free mesh placement, wide coverage of the defect, and meticulous adhesiolysis. [1] Perhaps less frequently discussed but just as important are the types of ports used as well as the appropriate number and positioning in relation to the hernia. These factors can have a significant affect on the surgeons strain and frustration if not utilitzed appropriately which in turn can lead to a sub-optimal repair.

**Port type:**

There are certainly many different approaches to establishing pneumoperitoneum and gaining visual access into the abdominal cavity. These include the use of Veress needle insufflation, the traditional open Hasson technique, or a modification of either of these techniques. The choice of approach is usually left to the surgeon’s preference and experience with these techniques. However, it is important to keep in mind that visual entry trocars have not been shown to decrease the incidence of visceral or vascular injury. They do have their advantage of decreasing the size of the port site wounds. [2] Level 2 evidence suggests that the surgeon must be cognizant of the risks and benefits of utilizing these trocars.

**Port positions and number:**

When planning for a laparoscopic ventral hernia repair, trocar positions should be determined by the site and size of the hernia. The fundamental principle of laparoscopic surgery still hold true which is the triangulation around the area of interest with an optimal distance from the target (16-18 cm). [3] The first trocar should always be placed as far as possible laterally from the defect to provide clear visualization of the defect margin. This allows the placement of subsequent trocars and to determine optimal mesh fixation points. Usually, a 10 mm optical trocar along with two 5 mm working trocars serve as the minimal number of ports necessary for a safe repair.

When dealing with midline and right -sided abdominal wall defects, three inline trocars in the left abdomen are ideal. Left sided abdominal defects are approached via three trocars on the right. [3-4] Small subxiphoid defects can be managed with the patient in a modified lithotomy position with the surgeon between the patient’s legs. The camera port is placed at the umbilicus and a five mm trocar on each side will provide excellent triangulation around the hernia. Larger subxiphoid defects eliminate the use of the umbilical port. In these situations, three trocars can be used in the left flank with the inferior most port closer to the midline. [3,5] Suprapubic defects can be dealt with in a similar fashion. Smaller defects can utilize the umbilicus as the camera port with two small working ports on either side. Larger suprapubic hernias can again be repaired with three left flank trocars with the uppermost port closer to the midline. [3,6] Additional ports should always be placed as needed, keeping in mind the principle of triangulation around the target. This will certainly be of benefit in difficult cases where extensive adhesiolysis is required or a large hernia sac is encountered.

**References**

1. Sauerland S, Walgenbach M, Habermalz B, Seiler CM, Miserez M. Laparoscopic versus open surgical techniques for ventral or incisional hernia repair. Cochrane Database Syst Rev. 2011 Mar;16(3). **(level 1A)**

2. Vilos GA, Ternamian A, Dempster J, Laberge PY. Laparoscopic entry: a review of techniques, technologies, and complications. J Obstet Gynaecol Can. 2007 May;29(5):433-65. **(level 2)**

3. Ferzli GS, Fingerhut A. Trocar placement for laparoscopic abdominal procedures: a simple standardized method. J Am Coll Surg. 2004 Jan;198(1):163-73. **(level 5)**

4. Carbajo MA, Martp del Olmo JC, Blanco JI, Toledano M, de la Cuesta C, Ferreras C, Vaquero C. Laparoscopic approach to incisional hernia. Surg Endosc. 2003 Jan;17(1):118-22. Epub 2002 Oct 29. **(level 4)**

5. Landau O, Raziel A, Matz A, Kyzer S, Haruzi I. Laparoscopic repair of poststernotomy subxiphoid epigastric hernia. Surg Endosc. 2001 Nov;15(11):1313-4. Epub 2001 Aug 16. **(level 4)**

6. Carbonell AM, Kercher KW, Matthews BD, Sing RF, Cobb WS, Heniford BT. The laparoscopic repair of suprapubic ventral hernias. Surg Endosc. 2005 Feb;19(2):174-7. Epub 2004 Dec 9. **(level 4)**

**Principles of adhesiolysis**

M. Rohr, J. Lang

*Search terms:*

*Hernia AND adhesiolysis (98)*

*Abdominal AND adhesiolysis (353)*

*Abdominal AND adhesiolysis AND treatment (316)*

A systemic search of the available literature was performed in August 2011 using Medline, PubMed, Cochrane library and relevant journals and reference lists using the above listed search terms

Without doubles there were 385 papers. During review of the literature we additionally obtained 9 papers connected to the topic and not covered by the search terms. Altogether 73 were considered to be relevant to the topic, of which 22 where cited in this paper.

**Key questions:**

**Best way of adhesiolysis? Sharp or bipolar or monopolar or ultrasonic dissection?**

**Statements:**

| Level 1b | Adhesiolysis offers no additional benefit in itself. |
| --- | --- |
| Level 3 | Adhesiolysis increases risk of enterotomy which increases mortality. |
| Level 4 | Age and number of previous operations increase risk of enterotomy during adhesiolysis. |
| Level 5 | Monopolar coagulation has a larger damage zone surrounding the coagulated tissue and produces higher temperatures.  Until today there is no reliable prevention of adhesions in abdominal surgery.  Use of monopolar electrocoaguation increases risk of enterotomy. |

**Recommendations**

| Grade B | Adhesiolysis should be limited to freeing the abdominal wall for overlapping mesh. |
| --- | --- |
| Grade C | Cold and sharp adhesiolysis is preferred, ultrasonic dissection or bipolar clamp is allowed, and monopolar coagulation should be avoided. |
| Grade D | Adhesiolysis should be done near the abdominal wall away from the adherent tissue. |

**Introduction**

Peritoneal adhesions are a frequent diagnosis during hernia operations. Although up to 25% have adhesions without previous operations ^1^ ^2^, adhesions form after nearly every invasive abdominal procedure ^3^. Adhesions are a major health problem, accounting for health costs in 1994 up to $2.3 billion ($1.4 billion for primary adhesiolysis; $926 million for secondary adhesiolysis) ^4^. Furthermore, adhesions are a cause for a lot of complications, even years after the original procedure. Adhesions are the number one cause for small bowel obstruction ^5^ and a cause for infertility^6 7^ while small bowel obstruction in absence of previous operations is a rare entity ^8^. Even more, adhesions are a cause for chronic abdominal pain and consequences of adhesions lead to a rather high rate of hospital readmission ^9^ while complicating future operations ^10^. Until today, there is no clinical therapy except for symptomatic treatment or surgical adhesiolysis, which is usually only recommended in emergencies as small bowel obstruction or during operations where adhesions are in the way of the surgeon since adhesions reformate in between 50% and 100% of the patients after adhesiolysis ^11^ ^3^. Another possibility is prevention of adhesions but again, until today there are no preventional therapies that are recommended without doubt, except for basic surgical procedures, e.g. powderless gloves ^12^.

In hernia operation, adhesiolysis is a basic part of the procedure since nearly all hernias show adhesions to the abdominal wall. It is part of this guideline to recommend principles of adhesiolysis during hernia operations.

**Discussion**

Overall evidence concerning adhesiolysis is unfortunately rather weak. The consequences of adhesions are well documentated but since there is no way to display adhesions short of a laparoscopy and adhesions start to form three days after the original procedure ^13^ a trial concerning the topic would need a second operation just for diagnostic purposes which is unethical.

An important topic is to decide how much adhesiolysis is useful. For hernia operations using a mesh adhesiolysis is needed to free the abdominal wall around the overlapping zone of the mesh. Should we continue and always attempt complete adhesiolysis? In the FINHYST trial (Level 2c) adhesiolysis was the strongest single risk factor for major complications as a whole [odds ratio (OR) 2.41, 95% (CI) 1.38–4.21] ^2^. We also found a Level 3 study that showed increasing enterotomies during relaparotomies when adhesiolysis was performed in the pelvis or between bowels, although no statistical significance was noted and the results have to be seen in connection with limited space during operations in the pelvis ^14^. However if an enterotomy happens, it increases mortality (Level 2a) ^15^. Age and number of previous operations are risk significant factors for enterotomy (Level 4) ^10^ and should be acknowledged when the extent of adhesiolysis is decided. On the other hand surgical adhesiolysis offers no additional benefit in e.g. chronic abdominal pain (Level 1b)^16^. Unfortunately we do not have studies comparing benefits of complete/partial adhesiolysis during hernia operation to a possible additional risk of enterotomy during extended adhesiolysis but the evidence points in the direction of a strategy that favors minimal adhesiolysis. Adhesiolysis should be done away from the adherent tissue and near the abdominal wall (Level 5) ^17^.

Adhesiolysis can be performed using several methods. Reformation of adhesions is unaffected by method of adhesiolysis in an animal model ^18^. There is level 4 evidence that ultrasonic dissection is safe in adhesiolysis ^19^ and a level 2c study showed less gallbladder perforations during laparoscopic cholecystectomy when harmonic scalpell was used (vs. Monopolar cautery) ^20^. We also know that a harmonic scalpell has a smaller damage zone and reaches lower temperatures than monopolar cautery ^21^. Again we found no study directly comparing different methods of adhesiolysis and their risks, although there is a italian consensus conference recommending cold and sharp adhesiolysis ^17^. In an animal model ultrasonic coagulating shears, electrothermal bipolar vessel sealer, titanium laparoscopic clips, and plastic laparoscopic clips all show sufficient hemostasis ^22^. Therefore to avoid enterotomy it is safer to refer to cold and sharp adhesiolysis or ultrasonic dissection for hemostasis.

**Conclusion**

Without additional benefits and certain well known risks, adhesiolysis should be done in a conservative way. High energy devices can be used with caution and monopolar cautery should be avoided.

**Literature:**

1. Weibel MA, Majno G. Peritoneal adhesions and their relation to abdominal surgery. A postmortem study. *American journal of surgery* 1973;126(3):345-53. **(level 5)**

2. Brummer TH, Jalkanen J, Fraser J, Heikkinen AM, Kauko M, Makinen J, et al. FINHYST, a prospective study of 5279 hysterectomies: complications and their risk factors. *Human reproduction (Oxford, England)* 2011;26(7):1741-51. **(level 2c)**

3. Diamond MP. Postoperative adhesion development after operative laparoscopy: evaluation at early second-look procedures. Operative Laparoscopy Study Group. *Fertility and sterility* 1991;55(4):700-4. **(level 4)**

4. Sikirica V, Bapat B, Candrilli SD, Davis KL, Wilson M, Johns A. The inpatient burden of abdominal and gynecological adhesiolysis in the US. *BMC surgery* 2011;11:13. **(level 2c)**

5. Menzies D, Ellis H. Intestinal obstruction from adhesions--how big is the problem? *Annals of the Royal College of Surgeons of England* 1990;72(1):60-3. **(level 2c)**

6. Hershlag A, Diamond MP, DeCherney AH. Adhesiolysis. *Clinical obstetrics and gynecology* 1991;34(2):395-402. **(level 5)**

7. Stovall TG, Elder RF, Ling FW. Predictors of pelvic adhesions. *The Journal of reproductive medicine* 1989;34(5):345-8. **(level 5)**

8. Butt MU, Velmahos GC, Zacharias N, Alam HB, de Moya M, King DR. Adhesional small bowel obstruction in the absence of previous operations: management and outcomes. *World journal of surgery* 2009;33(11):2368-71. **(level 2c)**

9. Parker MC, Ellis H, Moran BJ, Thompson JN, Wilson MS, Menzies D, et al. Postoperative adhesions: ten-year follow-up of 12,584 patients undergoing lower abdominal surgery. *Diseases of the colon and rectum* 2001;44(6):822-29; discussion 29-30. **(level 2c)**

10. Swank DJ, van Erp WF, Repelaer van Driel OJ, Hop WC, Bonjer HJ, Jeekel J. Complications and feasibility of laparoscopic adhesiolysis in patients with chronic abdominal pain. A retrospective study. *Surgical endoscopy* 2002;16(10):1468-73. **(level 4)**

11. Diamond MP, Daniell JF, Feste J, Surrey MW, McLaughlin DS, Friedman S, et al. Adhesion reformation and de novo adhesion formation after reproductive pelvic surgery. *Fertility and sterility* 1987;47(5):864-6. **(level 4)**

12. Ward BC, Panitch A. Abdominal adhesions: current and novel therapies. *The Journal of surgical research* 2011;165(1):91-111. **(level 5)**

13. Gomez-Gil V, Garcia-Honduvilla N, Pascual G, Rodriguez M, Bujan J, Bellon JM. Peritoneal adhesion formation and reformation tracked by sequential laparoscopy: optimizing the time point for adhesiolysis. *Surgery* 2010;147(3):378-91. **(level 5)**

14. Van Der Krabben AA, Dijkstra FR, Nieuwenhuijzen M, Reijnen MM, Schaapveld M, Van Goor H. Morbidity and mortality of inadvertent enterotomy during adhesiotomy. *The British journal of surgery* 2000;87(4):467-71. **(level 3)**

15. LeBlanc KA, Elieson MJ, Corder JM, 3rd. Enterotomy and mortality rates of laparoscopic incisional and ventral hernia repair: a review of the literature. *JSLS : Journal of the Society of Laparoendoscopic Surgeons / Society of Laparoendoscopic Surgeons* 2007;11(4):408-14. **(level 2a)**

16. Swank DJ, Swank-Bordewijk SC, Hop WC, van Erp WF, Janssen IM, Bonjer HJ, et al. Laparoscopic adhesiolysis in patients with chronic abdominal pain: a blinded randomised controlled multi-centre trial. *Lancet* 2003;361(9365):1247-51. **(level 1b)**

17. Vettoretto N, Carrara A, Corradi A, De Vivo G, Lazzaro L, Ricciardelli L, et al. Laparoscopic adhesiolysis: consensus conference guidelines. *Colorectal disease : the official journal of the Association of Coloproctology of Great Britain and Ireland* 2012;14(5):e208-15. **(level 1a)**

18. Tittel A, Treutner KH, Titkova S, Ottinger A, Schumpelick V. Comparison of adhesion reformation after laparoscopic and conventional adhesiolysis in an animal model. *Langenbeck's archives of surgery / Deutsche Gesellschaft fur Chirurgie* 2001;386(2):141-5. **(level 5)**

19. Swank DJ, Bonjer HJ, Jeekel J. Safe laparoscopic adhesiolysis with optical access trocar and ultrasonic dissection. A prospective study. *Surgical endoscopy* 2002;16(12):1796-801. **(level 4)**

20. Janssen IM, Swank DJ, Boonstra O, Knipscheer BC, Klinkenbijl JH, van Goor H. Randomized clinical trial of ultrasonic versus electrocautery dissection of the gallbladder in laparoscopic cholecystectomy. *The British journal of surgery* 2003;90(7):799-803. **(level 1b)**

21. Kinoshita T, Kanehira E, Omura K, Kawakami K, Watanabe Y. Experimental study on heat production by a 23.5-kHz ultrasonically activated device for endoscopic surgery. *Surgical endoscopy* 1999;13(6):621-5. **(level 5)**

22. Harold KL, Pollinger H, Matthews BD, Kercher KW, Sing RF, Heniford BT. Comparison of ultrasonic energy, bipolar thermal energy, and vascular clips for the hemostasis of small-, medium-, and large-sized arteries. *Surgical endoscopy* 2003;17(8):1228-30. **(level 5)**

**Laparoscopic ventral or incisional hernia repair- Importance of defining hernial defect margins and gauging size of the hernia preoperatively and intraoperatively.**

P. Chowbey

**Method-**The conclusion and recommendation for the significance of defining hernial defect size during incisional or ventral hernia repair are based on a systematic search and review of literature performed in Pubmed, Medline, Cochrane library, EMBASE, British journal of Surgery database, UK Pubmed Central, Google, Google scholar, Scirus, Ovid and Directory of Open Journal Access (DOAJ). Twenty -eight publications were found which covered the topic out of which eight statements were found useful for this research. In addition it is based on a consensus conference on guidelines for laparoscopic treatment of ventral and incisional hernia held on 13^th^ October 2011 in Suzhou, China.

**Search terms:** hernial defect size, hernial defect margins, hernial defect diameter, hernial defect area, laparoscopic contraindications, mesh size, measuring hernial defect size, incisional hernia, ventral hernia

**Statement**

| Level 2B | Size of hernia defect is a significant risk factor for recurrence in laparoscopic ventral/incisional hernia repair. |
| --- | --- |
| Level 3 | Accurate measurement of the size of the hernia defect is important, so that an appropriate surgical technique is chosen. |
| Level 3 | Accurate measurement of the defect is important, so that an appropriate sized mesh is chosen |
| Level 3 | The laparoscopic approach affords the surgeon the ability to clearly and definitively define the margins of the hernia defect and to identify additional defects that may not have been clinically apparent preoperatively. |

**Recommendations**

| **Grade B** | Accurate measurement of the hernial defect size should be done. |
| --- | --- |
| **Grade B** | Intracorporeal method of measurement of the size of hernial defect should be used. |

**Discussion**

Several decisions and outcomes related to laparoscopic incisional and ventral hernia (LIVHR) depend on the size of the hernial defect. In open surgery the size of the defect may play a minor role (1). But in laparoscopic repair its accurate measurement seems to be essential for estimating the proper size of the mesh to be used (2,3). Laparoscopic procedure is performed in patients with larger defect size (i.e. more than 15 cm)[4], however, this will not work without sufficient overlapping. The more overlapping the lower the recurrence rate will be (5). In so far precise measurement of defect size and correspondingly choose of an appropriate mesh size are indispensable preconditions for the success of the repair. To the determined size of the hernial defect, transverse and vertical dimension of 6- 10 cm is added and prosthesis slightly larger than these measurements is used for ensuring at least 3-5 cm overlap [6].

At present there is no standard and accurate method for measuring size of the hernial defect. Most commonly, measurement of the size of the hernial defect is estimated by physical examination which is not an accurate method. [7] Other methods include extracorporeally palpating hernial defect and marking it in distended abdominal cavity and then measuring it after deflation[6].

Intracorporeally, by placing spinal needles through the abdominal wall or placing intraperitoneal ruler after adhesiolyses. In addition the size of the hernia can be reported as the largest diameter of the hernial defect when measured directly intraperitoneally by a laparoscope [6,7].

Intracorporeal methods are more accurate and advantageous as compared to extracorporeal method. The laparoscopic approach defines the margins of the hernial defect clearly and definitively and helps in identifying additional defects that may have not been apparent preoperatively. In addition it prevents distortion of abdominal wall contour and the hernia sac[6,7,8].

In conclusion, gauging the size of the hernial defect is necessary for performing an optimal laparoscopic hernia repair.

**References** (in parentheses graduation of evidence)

1. Luijendijk RW, Hop WCJ, van den Tol MP, de Lange DCD, Braaksma MMJ, IJzermans JNM, et al. A Comparison of Suture Repair with Mesh Repair for Incisional Hernia. New England Journal of Medicine. 2000 Aug 10;343(6):392–8. **(level 1B)**
2. Birch DW. Characterizing laparoscopic incisional hernia repair. Can J Surg. 2007;50:195–201 **(level4)**
3. Moreno-Egea A, Camillo-alcaraz A, Aguayo-Albasini JL. Is the outcome of laparoscopic incisional hernia repair affected by defect size? A prospective study. Am J Surg 2012 Jan; 203(1):87-94 **(level 2B)**
4. Ferrari GC, Miranda A, Sansonna F, Magistro C, Di Lernia S, Maggioni D, Franzetti M, Pugliese R. Laparoscopic management of incisional hernias > or = 15 cm in diameter. Hernia. 2008 Dec;12(6):571-6. Epub 2008 Aug 8. **(level 4)**
5. Tsimoyiannis EC, Tsimogiannis KE, Pappas-Gogos G, Nikas K, Karfis E, Sioziou H. Seroma and recurrence in laparoscopic ventral hernioplasty. JSLS. 2008; 12(1):51-7.**(level 3)**
6. LeBlanc KA, Allain BW. Prevention and Management of Laparoendoscopic Surgical Complications, Third Edition. In: Kavic MS,Nezhat C, Winfield H, editors. Laparoscopic Repair of Ventral Wall Abdominal Hernia. World wide web: Society of Laparoendoscopic Surgeons (SLS); 2010.
7. Sharma A, Mehrotra M, Khullar R, Soni V, Baijal M, Chowbey P. Laparoscopic ventral/incisional hernia repair: a single centre experience of 1,242 patients over a period of 13 years. Hernia. 2011 Apr 1;15(2):131–9. **(level 3)**
8. Chowbey PK, Sharma A, Mehrotra M, Khullar R, Soni V, Baijal M. Laparoscopic repair of ventral / incisional hernias. J Min Access Surg 2006;2:192-8. **(level 5)**

**Bridging or augmentation? Reconstruction of the lineaalba – yes or no? Is it**

**necessary to close the defect before IPOM?**

Kukleta JF, Chelala E, Chowbey P

A systematic search of available literature was performed in August2011 and April 2012 using Pubmed, Medline, Cochrane Library and other relevant journals and reference lists with following search terms:
*„Augmentation repair“ AND „incisional hernia“ AND „bridging repair“ AND „ defect closure“, „hybrid repair“ AND „linea alba reconstruction “ AND „ incisional hernia“*.
Our search detected 53 articles for defect closure, 9 articles for augmentation repair, 3 for bridging repair, 1 for hybrid repair, 18 for linea alba reconstruction and 21 articles for linea alba and incisional hernia. Twenty seven articles were relevant although it’s the evidence was low (level 3, 4 and 5). We found no meta-analysis, no RCT, now comparative studies and no reviews.

The laparoscopic repair of ventral and incisional hernias was introduced by Karl LeBlanc in 1993 [1].
The IntraperitonealOnlay Mesh (IPOM) consists of reduction of the hernia content and patching the abdominal wall defect with an overlapping non absorbable synthetic mesh, which is tacked to the abdominal wall. The abdominal face of the intraperitoneally placed prosthetic is meant to prevent adhesions of the viscera to the mesh. In LeBlanc’s original technique the tacks were metallic. Due to the nature of the prosthetic (e-PTFE) the fixation material had to be of permanent character. The experience with the first 100 patients has lead to reinforcement of the tacked mesh with several additional transfascial mesh fixing sutures decreasing the recurrence rate from 9% to 4% in the next 100 patients [2].
 Such a “Bridging repair” may lead in larger hernias to a functional problem. The recti muscles detached from its origin at lineaalba not only lose the efficiency of their contraction, but compromise the function of the oblique muscles too. The balance between the anterior and posterior trunk muscles is disturbed.
The major goal of any open abdominal wall repair is not only reduction of hernia content and prevention of further herniation, but the restoration of integrity and restitution of abdominal wall functionality.
In case of the most frequent midline incisional hernias it is the restoration of the linea alba.

The laparoscopic version of such repair combines the transfascial transabdominal closure of the defect with the intraperitoneal onlay mesh placement. Such procedure is called “Augmentation repair” (or IPOM-Plus) in contrary to “Bridging repair” (the classical IPOM). The laparoscopicaly assisted transfascial suturing is achieved either transabdominaly with multiple interrupted sutures [3, 9,11] or intraabdominaly with a running suture [6].

The bridged area in IPOM is formed by mesh only (with no musculo-aponeurotic coverage) and as such functionally adynamic.

This creates the well known phenomenon of bulging and leaves space for seroma formation.
The sutured repair in IPOM-Plus reduces the hernia size to zero, eliminates bulging and decreases the seroma size and incidence, hence keeping the potential infection risk low. The defect closure enables bigger mesh overlap of 6 - 7 cm bilaterally, increases the total surface area of mesh contact with intact abdominal wall for future tissue in-growth and improves the solidity of fixation. Nevertheless the mesh size is laterally smaller which lowers the risk of nerve injury in problematic very lateral fixation.
The most important aspect of the augmentation repair is the uniform distribution of forces between the re-fixed defect, the tacked mesh margins, and the reinforcing transfascial sutures at the margins as well as the fixation lateral of the defect. Additional suture or tack fixation of the mesh around the defect (often called double crown technique) improves the mesh contact with the underlying abdominal wall and limits the dead space during the seroma formation. Although the straight defect closure is not feasible in every hernia due to inacceptable tension, the combination with endoscopic components separation technique will lower the tension and enable the closure increasing so the indication range. Hybrid techniques (combination of different approaches) can combine minilaparatomy for hernia closure followed by laparoscopic IPOM reinforcement with or without components separation.

Due to change of strategy from “tension free” to “non tension free” we have to answer following questions:

- Why to restore lineaalba or to close defects at all?
- Does IPOM-Plus offer clinical advantages?
- Does this technique reduce recurrence rate?
- Does it increase the risk of infection?
- Is IPOM-Plus functionally better than traditional IPOM?

**Statements**

| Level 3 | Reconstruction of Linea alba in laparoscopic incisional hernia repair improves the functionality of abdominal wall.  The reconstruction of the midline (even as open procedure) and the laparoscopic reinforcement through intraperitonealonlay mesh decrease the rate of wound complications.  Laparoscopic assisted transfascial of the midline defects is often feasible under “physiological tension”.  Despite of not being “tension-free”, the augmentation repair causes less pain in the early postoperative period than bridging repair.  Augmentation repair (due to combination of defect closure and extended mesh overlap), is stronger repair than bridging repair, if technically feasible. The usual overlap of 5cm can be extended to e.g. 8cm without increase of technical difficulty.  IPOM-Plus technique reduces the recurrence rate if compared with classical IPOM |
| --- | --- |
| Level 4 | Closing hernia defects in IPOM-Plus repair minimizes seroma incidence and prevents bulging, reducing patient’s discomfort   The augmentation repair decreases the recurrence rate and the incidence of chronic pain.  Reconstruction of linea alba without mesh reinforcement leads to high recurrence rates. |

**Recommendations**

| Grade B | The suture material for defect closure in IPOM-Plus should be non absorbable. |
| --- | --- |
| Grade C | Reconstruction of Linea alba (or any defect closure) in laparoscopic ventral or incisional hernia repair in combination with IPOM is recommendable in hernias of limited size. Additional component separation facilitates the closure and should be concerned in larger defects. |
| Grade D | The anterior transfascial suture technique should involve the hernia sack in order to obliterate the dead space as much as possible with the aim of prevention of seroma formation. |

**Discussion**

Chelala et al [8] presented in 2003 his “suturing concept for laparoscopic mesh fixation in ventral and incisional hernias”. An essential part of the concept was the defect closure with the U reverse stitches.
The same author [9, 11] reported improved outcomes based on growing experience (733 patients), longer follow-up and the experience with 85 redo surgeries [11].

Palanivelu et al [6] analyzed retrospectively 721 patients with laparoscopic incisional hernia repair. In a mean follow-up of 4.2 years only four recurrences (0.55%) were noted. The repair consisted of defect closure with running suture of polyamide and reinforcement with intraabdominalParietex composite mesh or Dualmesh.
Franklin et al [13] published in 2004 a retrospective analysis of 384 patients with laparoscopic abdominal wall hernia repair. In the mean follow-up time of 47.1 months 11 recurrences (2.9%) were found. Their standard repair was closure of large defects with non-absorbable interrupted sutures, even if only a limited closure was possible and reinforcement with non-absorbalemesh.

Banerjee et al [5] made a retrospective comparative study in 193 patients. His IPOM-Plus of interrupted non-absorbable sutures and intraperitoneal mesh reinforcement reached better recurrence rates than IPOM in primary and recurrent abdominal wall hernias ( 3% vs. 4.8% and 4.8% vs. 10.5% respectively).
Agarwal et al [4] describes a defect suturing technique using spinal needles as a threader needle and snare needle. He introduces the mesh through a 10mm port through the hernia defect, which is consecutively covered by the prosthetic mesh.

Sharma et al [19] proposes interrupted non-absorbable sutures with far-near-near-far stitching. It results in a kind of double-layered suture repair augmented with intraperitoneal mesh.
Orenstein et al [16] presents in 2011 the shoe-lacing technique for physiological abdominal wall reconstruction. The “figure-of-eight stitches” with non absorbable sutures close the defect. Non-absorbable cardinal sutures and additional absorbable transfascial sutures around the defect support the
circumferential fixation of the mesh margins with metallic or absorbable tacks. No infections were observed.
A systematic review on the outcomes of correction of diastasis of the recti by Hickey et al [17] demonstrates that the re-suturing without adequate support of mesh and sufficient fixation leads to unsatisfactory results.
To enable the defect closure in large hernias some additional operative steps may become necessary (hybrid procedures)[22, 26, 28].

**Comments**
Author’s personal experience with IPOM-Plus repair confirms the unexpected high rate of feasibility, the clinical advantages of restoration of lineaalba and the reduced infection rate. Seroma incidence and its size became unimportant in the most cases and the occasional bulging is not an issue anymore. It requires preferably 5 years of follow-up to confirm the actually decreased recurrence rates.
Some objective measurements need to be validated in the future in order to define more exactly the indications for IPOM-Plus repair.
The variability of the abdominal wall defects obviously demands a patient-oriented and hernia-oriented tailored approach. Only in theoretical and practical knowledge of variety of available operative steps the surgeon will be able to follow the strategy of tailored repair choosing the best solution for the individual well informed consenting patient.

**References and level of evidence**

1. LeBlanc KA, Booth WV (1993) Laparoscopic repair of incisional abdominal hernias using expanded polytetrafluoroethylene: preliminary findings. Surg Laparosc Endosc 3:39–41 (**level 5)**

2. LeBlanc KA, Whitaker JM, Bellanger DE, Rhynes VK. Laparoscopic incisional and ventral hernioplasty: lessons learned from 200 patients. Hernia. 2003 Sep;7(3):118-24. Epub 2003 Mar 21. (**level 3)**
3. Agarwal B, Agarwal S, Gupta M, Mishra A, Mahajan K (2008) Laparoscopic ventral hernia meshplasty with ‘Double breasted’ fascial closure of hernial defect. A new technique. J Laparoendosc Adv Surg Tech 18:222–229( **level 5)**

4. Agarwal BB, Agarwal S, Mahajan KC. Laparoscopic ventral hernia repair: innovative anatomical closure, mesh insertion without 10-mm transmyofascial port, and atraumatic mesh fixation: a preliminary experience of a new technique. Surg Endosc (2009) 23:900–905 DOI 10.1007/s00464-008-0159-7 ( **level 3B)**
5. Banerjee A, Narula VK, Mikami D. Laparoscopic ventral hernia repair – Does primary repair in addition to placement of mesh decrease recurrence? Surg Endosc (2012) 26:1264–1268 DOI 10.1007/s00464-011-2024-3 ( **level 2C -3)**

6. Palanivelu C, Jani KV, Senthilnathan P, Parthasarathi R, Madhankumar MV, Malladi VK (2007) Laparoscopic sutured closure with mesh reinforcement of incisional hernias. Hernia 11:223–228 (**level 4)**

7. Palanivelu C, Rangarajan M, Rajapandian S, Amar V, Parthasarathi R. Laparoscopic repair of adult diaphragmatic hernias and eventration with primary sutured closure and prosthetic reinforcement: A retrospective study. Surg Endosc (2009) 23:978–985 DOI 10.1007/s00464-008-0294-1 ( **level 4)**

8. Chelala et al (2003) Hernia 7:191-196. The suturing concept for laparoscopic mesh fixation in ventral and incisional hernias: preliminary results. **(level 5)**

9. Chelala E, Thoma M, Tatete B, Lemye AC, Dessily M, Alle JL (2007) The suturing concept for laparoscopic mesh fixation in ventral and incisional hernia repair: mid-term analysis of 400 cases. Surg Endosc 21(3):391–395 ( **level 3)**

10. Losanoff JE, Basson MD, Laker S, Weiner M, Webber JD, Gruber SA (2007) Sutured laparoscopic mesh fixation. Surg Endosc Jun 26 [Epub ahead of print]( **level 5)**

11. Chelala E, Debardemaeker Y, Elias B, Charara F, Dessily M, Allé JL. Eighty-five redo surgeries after

733 laparoscopic treatments for ventral and incisional hernia: adhesion and recurrence analysis. Hernia 2010 14(2):123-9. Epub 2010 Feb 14. ( **level 3)**

12. Misra MC, Bansal VK, Kulkarni MP, Pawar DK. Comparison of laparoscopic and open repair of incisional and primary ventral hernia: results of a prospective randomized study. Surg Endosc. (2006) 20(12):1839-45.( **level 1B)**

13. Franklin ME, Gonzalez JJ, Glass JL et al (2004) Laparoscopic ventral and incisional hernia repair: an 11-year experience. Hernia 8(1):23–27 (**level 4 )**

14. Carter JE. A new technique of fascial closure for laparoscopic incisions. *J Laparoendoscopic Surg*. 1994; 4: 143–148. **(level 5)**

15. Parker M, Goldberg RF, Dinkins MM, Asbun HJ, Daniel Smith C, Preissler S, Bowers SP. Pilot study on objective measurement of abdominal wall strength in patients with ventral incisional hernia. Surg Endosc. Surg Endosc (2011) 25:3503–3508 ( **level 2B)**16. Orenstein SB, Dumeer JL, Monteagudo J, Poi MJ, Novitsky YW. Outcomes of laparoscopic ventral hernia repair with routine defect closure using ‘‘shoelacing’’ technique Surg Endosc (2011) 25:1452–57. (**level 4)**

17. Hickey F, Finch JG, Khanna A. A systematic review on the outcomes of correction of diastasis of the recti. Hernia 2011 Jun 18. [Epub ahead of print]. 15:607 – 614 ) **level 2A)**

18. Alder AC, Alder SC, Livingston EH, Bellows CF. Current Opinions About Laparoscopic Incisional Hernia Repair - A Survey of Practicing Surgeons. Am J Surg. 2007 November; 194(5): 659–662. ( **level 4)**

19. Sharma D, Jindal V, Pathania OP, Thomas S. Novel technique for closure of defect in laparoscopic ventral hernia repair J Minim Access Surg. 2010 Jul-Sep; 6(3): 86–88. ( **level 4)**

20. Cox TC, Pearl JP, Ritter EM. Rives-Stoppa incisional hernia repair combined with laparoscopic separation of abdominal wall components: a novel approach to complex abdominal wall closure. Hernia (2010) 14(6):561-7. Epub 2010 Jul 27. (**level 4)**

21. Van Geffen HJAA, Simmermacher RKJ.Incisional Hernia Repair: Abdominoplasty, Tissue Expansion, and Methods of Augmentation. World J. Surg. 29,1080–1085 (2005) (**level 4)**

22. Barnes GS, Papasavas PK, O'Mara MS, Urbandt J, Hayetian FD, Gagn~ DJ, Newton ED, Caushaj PF Modified extraperitoneal endoscopic separation of parts for abdominal compartment syndrome Surg Endosc (2004) 18:1636-1639 ( **level 5)**

23. Espinosa-de-Los-Monteros A, de la Torre JI, Ahumada LA, Person DW, Rosenberg LZ, Vásconez LO.Reconstruction of the abdominal wall for incisional hernia repair. Am J Surg. 2006 Feb;191(2):173-7. (**level 4)**

24. Nguyen NT, Lee SL, Mayer KL, Furdui GL, Ho HS (2000) Laparoscopic umbilical herniorrhaphy.
J Laparoendosc Adv Surg Tech A 10: 151–153 (**level 4)**

25. Eid GE, Thodiyil PA, Collins JC, Bonanomi G, Mattar SG, Hughes SJ, Schauer PR,Wilson M. Laparoscopic Repair of Umbilical Hernias in Conjunction With Other Laparoscopic Procedures. JSLS. 2006 Jan-Mar; 10(1): 63–65. **(level 4)**

26. Griniatsos J, Yiannakopoulou E, Tsechpenakis A, Tsigris C, Diamantis T. A hybrid technique for recurrent incisional hernia repair. Surg Laparosc Endosc Percutan Tech. 2009 Oct;19(5):e177-80.( **level 4)**

27. Schug-Pass C, Trommer Y, Tamme C, Lippert H, Köckerling F. Dynamic patchplasty--a tension-free reconstruction of incisional hernias. Langenbecks Arch Surg. 2006 Aug;391(4):403-8; discussion 409-10. (**level 4)**

28. Mathes SJ, Steinwald PM, Foster RD, Hoffman WY, Anthony JP Complex Abdominal Wall Reconstruction: A Comparison of Flap and Mesh Closure Ann Surg Vol. 232, No. 4, 586–596 ( **level 4)**

29. Beldi G, et al. Mesh shrinkage and pain in laparoscopic ventral hernia repair: a randomized clinical trial comparing suture versus tack mesh fixation. Surg.Endosc.(2011) 25:749-755 (**level 2B )**

***HOW MUCH OVERLAP IS NECESSARY?***

Salvador Morales-Conde

***INTRODUCTION***

One of the technical aspects related to laparoscopic ventral hernia repair (LVHR) that have been widely discuss by experts on this field in different forum in the last decade, are those factors that may be related to recurrences. A low recurrence rate is one of the main challenges of surgeons who perform abdominal wall surgery. Recurrence rate after LVHR was widely analyzed by different authors at the beginning of this technique, establishing a direct relationship between the method of fixation and this fact, but different authors started to pay special attention to the fact that the overlap of the mesh initially used in the series was only of 2 cm. As experience has been gained, authors start recommended larger overlap of the mesh used during LVHR.

***METHOD***

A Medline search was performed until November 2011, using the following terms: laparoscopic repair, ventral hernia, ventral defect, overlapping, overlap and mesh size.

The numbers of papers identified were 78 (following the flow indicated in figure 1). The number of papers analyzed were 23, being excluded 55 for the following reasons (Figure 2): 3 were clinical studies not related to the topic being studied, 2 were experimental studies not related to overlap during LVHR, 41 just analyze mesh size related to the size of the defect, without describing the overlap of the mesh in the different directions, 4 describes overlap during open repair and 5 did not establish a number of centimeters when they describe the overlap, just mentioning the word a “sufficient” overlap.

Out of the 23 papers included in the final analysis, there were no papers with level of evidence 1 or 2, only 2 papers with level of evidence 3a (1, 2), 2 with level of evidence 3b (3, 4), 14 with level of evidence 4 (5-18) and 5 with level of evidence 5 (19-23).

***Statements***

| **Level 3** | - Recurrence will be increased by using an inadequate prosthetic OVERLAP of the fascial defect, existing a relationship between OVERLAP of the defect and recurrence  - Larger meshes with larger OVERLAP are related to lower recurrence rate  - To prevent recurrences, the surgeon must assure that there is at least a 3-4 cm OVERLAP in all directions of the hernia defect |
| --- | --- |
| **Level 4** | - Mesh must OVERLAP the fascial defect at least 5 cm in all directions  - If structures like the falciform ligament, the ligamentum teres and the prevesical fatty tissue are not dissected a proper fixation and incorporation of the mesh in the area where the mesh OVERLAP the fascial defect is hardly possible  - A larger OVERLAP of the prosthesis (5 vs 3 cm) is necessary if sutures are not used, being more important for the overlap than the use of transfascial sutures for fixation of the mesh  - Smaller defect need smaller OVERLAP than larger defect in order to avoid recurrences  -Recurrence after incisional hernia repair appears to be due primarily to disregard for the principles that the whole incision (not just the hernia) must be repaired |

***Recommendations***

| ***Grade B*** | - Mesh during laparoscopic repair of ventral hernia SHOULD OVERLAP the hernia defect at least 3-4 cm in all directions |
| --- | --- |
| ***Grade C*** | - It is recommended to OVERLAP the defect at least 5 cm in all directions  - For proper fixation and incorporation of the mesh removal of different anatomical structures like the falciform ligament, the ligamentum teres and the prevesical fatty tissue should be done.  - A larger OVERLAP could be necessary, minimum of 5 cm, if you fix the mesh without transfascial sutures  - It is recommended to use larger OVERLAP in larger hernias compared with the overlap used in smaller hernias  - In order to avoid recurrences, the entire incisional scar should be covered by the mesh, even if the defect is overlapped 3-5 cm in all direction. |

***DISCUSSION***

There is a low level of evidence and grade of recommendations to establish the proper overlap when a LVHR is performed. Initially, surgeons related recurrences to the method of fixation. In fact, LeBlanc (2), in 2004, established that the main reason of recurrence after LVHR was related to those cases in which transfascial sutures were not used, even when, at that time, an overlap of just 2 cm was used. Both studies with level of evidence 3a (1, 2), showed that a larger overlap of the prosthesis (5 vs 3 cm) was necessary if sutures were not used. If sutures were used, these studies recommended to place them no more than 5 cm apart. K LeBlanc recommended in his paper prospective randomized trials comparing techniques with and without of transfascial sutures using a consistent biomaterial to settle this issue. Further studies demonstrated that the method of fixation was important, but that it was not the key factor related to recurrences. The Double Crown technique, described by S Morales-Conde et al (13), demonstrated to have similar results using a technique without transfascial sutures, using two rows of spiral tacks. Technical reasons, together with a short overlap of the defect in all directions, has been demonstrated to be one of the key factor related to recurrences, being more important than the method of fixation.

EC Tsimoyiannis et al conducted a study with 78 patients who underwent 80 LVHR placing an ePTFE dual mesh intraperitoneally fixed using full-thickness stitches and endoscopic tacks. Patients included in this study were divided into 2 groups: group A, with 28 patients, overlapping the hernia defect 2.5 cm in 17 cases (subgroup A1) or 4 cm in 11 cases (subgroup A2). The second group included 52 patients following the same technique as in group A, but the hernia sac was cauterized by monopolar cautery (n=5) or Harmonic scalpel (n=47). The overlapping healthy margins were at least 2.5 cm, in 16 cases (subgroup B1) or 4 cm, 36 cases (subgroup B2). In subgroup A2 and B2, a full-thickness suture was placed in the center of the hernia defect to reduce the dead space. Regarding recurrences, the authors concluded that the combinations of a large patch to cover at least 4 cm of healthy margins and the surgeon's experience were sufficient to prevent recurrences in LVHR. Even that we can draw some conclusions out of this study, one of the main criticism of this paper is that many different technical factors were mixed, and the maneuvers performed to decrease seroma could have some influence in the rate of recurrences.

On the other hand, it is difficult to draw conclusions out of the 14 studies with level evidence 4, since 3 of them are based only in less than 10 cases (6, 11, 16).

But, even that the literature is not too useful to establish high grade of recommendations, what surgeons have learned in the last 15 years regarding this topic, is that the mesh should overlap the hernia defect at least 3 to 5 cm in all directions, and this distance should be larger as the defect is larger. The need of a large overlap is basically related to three factors: the first one is related to the intraabdominal pressure, that will attach the mesh better against the abdominal wall if its surface is larger. The second aspect, is that the mesh will have more surface to interact with the abdominal wall, increasing the in-growth and therefore the biological fixation of the prosthetic material. The third reason seems to be related to the shrinkage of the mesh, since all mesh reduce in size with time once they have been implanted, a larger surface of a mesh will reduce the possibility of exposing the defect and having a recurrence.

Another important issue is the importance of covering the whole previous scar in order to avoid a weak area in the abdominal wall, where a new hernia or a recurrence could occur (5).

But, even that there is no a strong level of evidence to clearly demonstrate the importance of the overlap, there is no place to run studies comparing an overlap less than 5 cm. One of the only doubts that remains is the relation between the size of the defect and the overlap, since the literature just recommend larger overlap with larger defect, without determining the proportion of the size of the hernia defect and the overlap.

***REFERENCES***

1.- LeBlanc KA. Laparoscopic incisional hernia repair: are transfascial sutures necessary? A review of the literature. Surg Endosc. 2007; 21(4):508-13.**(level 3a)**

2.-LeBlanc KA.Laparoscop icincisional and ventral herniarepair: complications-how to avoid and handle. Hernia. 2004; 8(4):323-31.**(level 3a)**

3.-Tsimoyiannis EC, Tsimogiannis KE, Pappas-Gogos G, Nikas K, Karfis E, Sioziou H. Seroma and recurrence in laparoscopic ventral hernioplasty. JSLS. 2008; 12(1):51-7.**(level 3b)**

4.- LeBlanc KA, Whitaker JM, Bellanger DE, Rhynes VK. Laparoscopic incisional and ventral hernioplasty: lessons learned from 200 patients. Hernia. 2003; 7(3):118-24.**(level 3b)**

5.- Wassenaar EB, Schoenmaeckers EJ, Raymakers JT, Rakic S. Recurrences after laparoscopic repair of ventral andincisionalhernia: lessons learned from 505 repairs. Surg Endosc. 2009;23(4):825-32. **(level4)**

6.-Abir F, Eisenberg D, Bell R. Laparoscopic ventral hernia repair using a two (5-mm) port technique.JSLS.2005;9(1):94-6.**(level4)**

7.-Ho J, Pigazzi A. Laparoscopic repair of extraction site ventral hernia after robotic prostatectomy: institutional experience with 42 consecutive cases. Hernia. 2011;15(6):673-6.**(level4)**

8.- Lambrecht J. Overlap-coefficient for the relationship between mesh size and defect size in laparoscopic ventral hernia surgery. Hernia. 2011;15(4):473-4.**(level4)**

9.- Rosen MJ. Polyester-based mesh for ventral herniarepair: is it safe? Am J Surg. 2009;197(3):353-9.**(level4)**

10.-Baccari P, Nifosi J, Ghirardelli L, Staudacher C.Laparoscopic incisional and ventral hernia repair without sutures: a single-center experiencewith 200 cases. J Laparoendosc Adv Surg Tech A. 2009 Apr;19(2):175-9.**(level4)**

11.- Zacharakis E, Hettige R, Purkayastha S, Aggarwal R, Athanasiou T, Darzi A, Ziprin. P.Laparoscopic parastomal hernia repair: a description of the technique and initial results. Surg Innov. 2008;15(2):85-9.**(level4)**

12.- Shah RH, Sharma A, Khullar R, Soni V, Baijal M, Chowbey PK. Laparoscopic repair of incarcerated ventral abdominal wall hernias. Hernia. 2008;12(5):457-63. **(level4)**

13.- Morales-Conde S, Cadet H, Cano A, Bustos M, Martín J, Morales-Mendez S. Laparoscopic ventral hernia repair without sutures—double crown technique: our experienceafter 140 cases with a mean follow-up of 40 months. Int Surg. 2005;90(3Suppl):S56-62.**(level4)**

14.- Jerabek J, Piskac P, Hnizdil L, Bucek J. The laparoscopic ventral and incisional hernia repair. Bratisl LekListy. 2005;106(3):147-8.**(level4)**

15.- Topart P, Ferrand L, Vandenbroucke F, Lozac'h P. Laparoscopic ventral hernia repair with the Goretex Dualmesh: long-term results and review of the literature. Hernia. 2005;9(4):348-52. **(level4)**

16.- Tagaya N, Mikami H, Aoki H, Kubota K.Long-term complications of laparoscopic ventral and incisional hernia repair. Surg Laparosc Endosc Percutan Tech. 2004;14(1):5-8.**(level4)**

17.- LeBlanc KA, Booth WV, Whitaker JM, Bellanger DE. Laparoscopic incisional and ventral herniorraphy: our initial 100 patients. Hernia. 2001;5(1):41-5.**(level4)**

18.- Alkhoury F, Helton S, Ippolito RJ.Cost and clinical outcomes of laparoscopic ventral hernia repair using intraperitoneal non heavyweight polypropylene mesh. Surg Laparosc Endosc Percutan Tech. 2011;21(2):82-5.**(level4)**

19.- Berger D. Laparoscopic IPOM technique. Chirurg. 2010;81(3):211-5.**(level5)**

20.- Bachman S, Ramshaw B.Prosthetic material in ventral herniarepair: how do I choose? Surg Clin North Am. 2008;88(1):101-12, ix.**(level5)**

21.- Cobb WS, Kercher KW, Heniford BT.Laparoscopic repair of incisional hernias.Surg Clin North Am. 2005;85(1):91-103, ix.**(level5)**

22.- Trap R, Schulze S, Kristiansen VB. Ventral herniotomy. Development of surgical technique and effect on the frequency of recurrence. Ugeskr Laeger. 2003;165(7):672-8.**(level5)**

23.- LeBlanc KA. The critical technical aspects of laparoscopic repair of ventral and incisional hernias. Am Surg. 2001;67(8):809-12.**(level5**

**Fixation**

R.H. Fortelny, M. Misra, F. Köckerling, J.Kukleta

*Search terms: “laparoscopic hernia repair“ AND “LVHR“ AND “incisional hernia“ AND “ventral hernia“ AND “fixation“ AND “sutures“ AND “tacks“ AN“staples“ AND “recurrences” AND “pain” AND “long term results”*

A systemic search of the available literature was performed in August 2011 using Medline, PubMed, Cochrane library and relevant journals and reference lists using the above listed search terms

The first search detected 64 relevant articles. In a second-level search 14 articles were added. In summary 78 articles including 36 studies were used for this review.

**Key questions:**

**Best type of fixation? Are permanent sutures needed?**

**Suture vs. Tacker – what is better?**

# Statements

| Level 1B | There is no significant difference of acute postoperative pain concerning the different type of mesh fixation by sutures, tacks or combination of both.  Suture fixation technique requires significantly longer operation time in comparison to tacker fixation.  The absorbability of the suture material used for mesh fixation is not related to the incidence of postoperative pain.  Tacker only fixation is associated with a significantly higher grade of mesh shrinkage in horizontal direction compared to transfascial suture fixation.  In case of umbilical hernias with a defect size up to 5 cm, mesh fixation by glue achieves less acute postoperative pain in comparison to tacker fixation in a short follow up. |
| --- | --- |
| Level 3 | The incidence of acute postoperative pain correlates significantly with the number of tacks used for mesh fixation. |
| Level 4 | The different fixation techniques - combination of sutures with tacks, tacker only and suture only fixation respectively are without significant difference concerning the recurrence rate.  The application intervals of staples/tacks in single or double crown technique of 1,5 cm are associated with low recurrence rate.  Irrespective of the type of fixation technique, there is no significant difference concerning the incidence of postoperative chronic pain.  The use of resorbable penetrating fixation devices achieves sufficient tensile strength and low recurrence rates.  The use of additional glue fixation increases the efficacy of fixation and implicates the reduction of penetrating devices as well as the risk of postoperative pain. |
| Level 5 | Penetrating fixation devices (e.g. transfascial sutures, protruding tacks) can cause incisional hernias and in the region of the pericard result in a cardiac tamponade­­­­­­­ |

**Recommendations:**

| Grade B | Suture fixation only or a combination with tacks should be performed to decrease the risk of mesh shrinkage. |
| --- | --- |
| Grade C | Fixation in laparoscopic repair for ventral and incisional hernias performed by sutures, tacks or combination of both can be recommended equivalently in terms of risk of recurrence and postoperative pain presupposing adequate technique (e.g. intervals of fixation and overlap of mesh).  Regarding the significant shorter operation time the tacker only fixation can be considered as technique of choice taking into account the increased risk of postoperative pain due to the number of devices and the need for an additional overlap of mesh (at least 5 cm) to prevent recurrence caused by shrinkage.  The reduction of penetrating fixation devices decreasing the risk of postoperative pain and device-induced hernia can be achieved by additional use of glue fixation. |

**Introduction:**

Since the introduction of laparoscopic surgery in ventral and incisional hernia (LVHR) by LeBlanc in 1991{57} an increased interest in technical aspects as well as in new mesh material and fixation devices has been observed, especially over the last decade. Nevertheless, one of the most controversially discussed topics in this field up to now is the type and technique of fixation. The majority of reports present the so-called traditional technique of transfascial sutures and tacker fixation e.g. Heniford et al. {58} and LeBlanc {6} achieving low recurrence rates of 4,7% and 4% respectively. LeBlanc demonstrated that additional transfascial suture fixation and an increased mesh overlap could reduce the recurrence rate from 9 to 4% {6}. On the other hand several studies certified the efficacy of tacker only fixation. Frantzides et al {17} and Carbajo et al. {16} performed this technique and obtained even very low recurrence rates of 1,4 and 4,4% respectively.

Another area of incremental interest is the correlation of fixation and incidence of postoperative pain. The discussion concerning the increased recurrence risk due to fewer fixation devices e.g. transfascial sutures, is still going on {77}. Finally new absorbable fixation devices like tacks, staples and glues were developed to reduce the risk of chronic postoperative pain. But there is still a lack of clinical studies comparing different fixation devices and different mesh-types (e.g. porosity, elasticity, coating).

**Fixation: Transfascial sutures with tacks, versus sutures only and versus tacks only fixation:**

**Recurrences:**

Concerning recurrence rate, the comparison of the usually performed 3 types of fixation techniques - transfascial sutures with tacks, sutures only and tacks only - were compared. Following the recommendation of Kapischke et al. {1} a meta analysis can only based on studies following surgical and statistical demands e.g. standardized operation-technique, specially fixation technique, postop evaluation after a standard protocol (e.g. pain assessment) including a minimum of 5 years follow up, for a sufficient judgement of the recurrence rate.

For this review we used a modification of the recommendation of Kapischke et al.{1} including only studies with a minimum of 100 patients and follow up of at least of 24 months – in total 23 studies were selected. In the group of transfascial sutures and tacks 10 studies {2-11}, in the suture group only 2 studies {12,13} and the tack only group 11 studies {8,10,13-22} fulfilled these criteria for inclusion (Tab.1).

The cumulative recurrence rate of all three groups including 5884 patients of 23 studies was median 3,95 % (2-5,6) at an cumulative follow up time of median 35,5 months (29-48) (Tab.1).

The recurrence rates of the suture and tack fixation groups comprising2211 patient revealed 3,65 % (2,45-5,75), sutures only fixation including 1121 patients1,05 % (0,82-1,27) and tacks only fixation involving 3473 patient4,5 % (2,4-6,17) respectively (Tab.2).

Comparing the results of the three groups (by Kruskal-Wallis and ANOVA test) no significant difference regarding the recurrence rate and the follow up time was detected (Tab.2). The two studies {12,13} using suture only repair based on the principle of suture closure of the defect and mesh-reinforcement of the abdominal wall in contrast to the usual IPOM technique obtained the lowest recurrence rate of 1,05 % but failed to show statistical significance compared to the other groups.

Due to the variability of patient characteristics and non-standardized technique of different fixation and different meshes-types used these results must be rated in awareness of a possible bias, but also considering the fact that data of randomized controlled trials is lacking.

**Tab. 1**

| Author | Study | Patients | Type of fixation | Recur-rences % | Follow up | Level of evidence |
| --- | --- | --- | --- | --- | --- | --- |
| Ballem  et al. 2008{2} | case-series | 119 | sutures + tacks | 28,6 | 90 | IV |
| Berger  et al. 2002 {3} | case-series | 296 | sutures + tacks | 0,3 | 24 | IV |
| Bingener  et al. 2007 {4} | case-series | 127 | sutures + tacks | 10,2 | 30 | IV |
| Franklin  et al. 2004 {5} | case-series | 335 | sutures + tacks | 2,4 | 47 | IV |
| LeBlanc  et al. 2003 {6} | case-series | 100 | sutures + tacks | 4 | 36 | IV |
| Mc Kinlay  et al. 2004{7} | case-series | 169 | sutures + tacks | 5,9 | 25 | IV |
| Sharma  et al. 2011 {8} | retro-  spective | 544 | sutures + tacks | 2,6 | 63 | III |
| Topart  et al. 2005 {9} | case-series | 151 | sutures + tacks | 5,3 | 27 | IV |
| Wassenaar  et al. 2009 {10} | retro-  spective | 299 | sutures + tacks | 1,7 | 31 | IV |
| Yavuz  et al. 2005 {11} | case-series | 150 | sutures + tacks | 3,3 | 32 | IV |
| Chelala  et al. 2007 {12} | case-series | 400 | sutures | 1,5 | 28 | IV |
| Palanivelu  et al. 2007 {13} | case-series | 721 | sutures | 0,6 | 50 | IV |
| Bencini  et al. 2009 {14} | case-series | 146 | tacks | 8,2 | 45 | IV |
| Bageacu  et al. 2002 {15} | retro-  spective | 146 | tacks | 13,9 | 40 | IV |
| Carbajo  et al. 2003 (16) | case-series | 269 | tacks | 4,5 | 44 | IV |
| Chowbey  et al. 2000 {17} | retro-  spective | 202 | tacks | 1,0 | 35 | IV |
| Frantzides  et al. 2004 {18} | retro-  spective | 208 | tacks | 1,4 | 24 | IV |
| Kirshtein  et al. 2002 {19} | retro-  spective | 100 | tacks | 4,0 | 26 | IV |
| Moreno-Egea  et al. 2008{20} | case-control | 199 | tacks | 5,5 | 64 | IIb |
| Morales  et al. 2005 {21} | case-series | 140 | tacks | 2,1 | 40 | IV |
| Olmi  et al. 2006{22} | case-series | 178 | tacks | 2,5 | 29 | IV |
| Sharma  et al. 2011 {8} | retro-  spective | 688 | stapler/tacks | 10/3,9 | 63 | III |
| Wassenaar  et al. 2009 {10} | retro-  spective | 206 | tacks | 4,3 | 31 | IV |
| **Total** |  | **5884***  **(143-297)** |  | **3,95***  **(2-5,6)** | **35,5***  **(29-48)** |  |

* median (IQR)

InterQuartile Range (IQR)

**Tab.2**

| **Type of fixation** | **Numberof studies** | **Total number of patients** | **Recurrence-rate in %**  median (IQR) | **Follow up**  **month**  median (IQR) |
| --- | --- | --- | --- | --- |
| **Sutures+ tacks** | 10 | 2211 | 3,65 (2,45-5,75)**^#^**” | 31,5 (27,75-38,25) |
| **Sutures only** | 2 | 1121 | 1,05 (0,82-1,27)**^#^**” | 39 (33,5-44,5) |
| **Tacks only** | 11 | 2473 | 4,5 (2,4-6,17)**^#^**” | 40 (30,5-49,5) |

**^#^ Kruskal-Wallis Test: p = 0,17**

**“ ANOVA: p= 0,535**

**Postoperative pain:**

**Acute postoperative pain**

Concerning the incidence of acute postoperative pain 4 RCT–1B studies {23,24,25,26,} and 1 prospective 2B study {27} were analysed.

In the study of Wassenaar et al. {23} 172 patients were included and randomized in 3 groups: absorbable sutures with tacks (n=56) vs tacks in double crown technique( n= 60) vs non absorbable sutures with tacks ( n= 56). The pain assessment by means of visual analog scoring was performed preoperatively, 2 weeks, 6 weeks and 3 months postoperatively. Additionally, QoL(SF36) was assessed preoperatively and 3 months postoperatively.

No significant differences among the different fixation techniques in terms of pain were detected at all timepoints.

68 patients were enrolled in the study of Bansal et al. {24} and randomized in 2 groups: tacks (n=36) versus nonabsorbable sutures (n=32). In the tacker-fixation group significantly higher pain scores were obtained at 1,6 and 24 hours, as well as at 1 week and 3 months postoperatively in comparison to the suture group.

Beldi et al. {25} included 40 patients in the trial and randomized in 2 groups: nonabsorbable sutures (n=20) versus tacks (n=20). The assessment of pain in the transfascial suture group revealed significant higher pain scores at 6 weeks but reached no significant difference at 6 months in comparison to the tacker group. Nguyen et al. {27} did another prospective study in terms of acute postoperative pain. The pain assessment in the 2 groups: sutures (n=29) versus tacks (n=21) revealed no significant difference at 1 week, 1 month and 2 months postoperatively.

Eriksen et al. {26} included 40patients with an umbilical hernia defect (1,5 to 5 cm) at three Danish hernia centers. Patients were assigned randomly (20/20) to fibrin sealant fixation (4 units/ml thrombin) or titanium tack fixation (double crown). The assessment of acute pain (days 0–2 postop) by VAS (0-10) detected significant less pain in the fibrin sealant group in comparison to the tacker group at rest (median 19 versus 47 mm; P = 0·025) and during activity (38 versus 60 mm; P = 0·014).

Bansal et al. {24} reported the cause of less pain in the suture group might be based on the technique of “loose tying of the sutures”. Although a significant difference to the tacker group is seen, the pain-scores in both groups are very low: at 1 week: 2,5/1,6; 1 month 1,5/0,6 and 3 month: 0,6 /0,14. Considering this fact pain scores of less than 2,5 and 1,5 respectively should not overestimated as a significant decrease in terms of quality of life.

Comment: In the laparoscopic treatment of small umbilical hernia defects the feasibility of fixation by glue could be an alternative to the penetrating fixation by tacks and sutures. Albeit the incidence of less acute postop. pain seems to be an remarkable advantage, the long term results remains to be seen.

| **Authors** | **Study** | **Pat.**  **total (groups)** | **Typ of fixation** | **Assess-ment**  **weeks**  **days*** | **Acute Pain**  **Sut./FS/Tack** | **p-value** | **Level of evidence** |
| --- | --- | --- | --- | --- | --- | --- | --- |
| Wassenaar et al. 2010 {23} | RCT | 172  (56/60/56) | sr+t vs tvs sn+t | 2/6/18 | ns/ns / ns | >0.05 | Ib |
| Bansal  et al. 2011  {24} | RCT | 68  (32/36) | sn vs t | 1***/1**/12 | s/s / s | <0.05 | Ib |
| Beldi et al. 2011  {25} | RCT | 40  (20/20) | sn vs t | 6/24 |  s/ns | 0.020 | Ib |
| Eriksen  et al. 2011  {26} | RCT | 38  (19/19) | fs vs t | 2*/10* |  s/s | 0.025 | Ib |
| Nguyen et al. 2008  {27} | prosp.  comp. | 50  (29/21) | sn vs t | 1/4/8 | ns/ns/ns | >0.05 | IIb |

suture non resorb. (sn), suture resorb. (sr), tacks (t) , fibrin sealant (fs)

significant (s)

non significant (ns)

**Chronic postoperative pain:**

Chronic pain is defined by pain lasting at least 6 months postoperatively.

To find a correlation of different fixation techniques and the incidence of chronic postoperative pain the main three different groups (transfascial sutures and tacks {2-11}, sutures only {12,13} tacks only {8,10,13-22}) were analysed.

The percentage of chronic pain in the group of sutures and tack fixation was median 2,75%, of sutures only 3,75% and 6,35% respectively. In comparison of the groups no significant differences were detected using the Kruskal-Wallis test (p = 0,845) and ANOVA test (p= 0,747) (Tab3.)

**Tab.3**

| **Type of fixation** | **Number of studies** | **Total number of patients** | **Chronic pain**  **%**  median (IQR) | **Follow up**  **month**  median (IQR) |
| --- | --- | --- | --- | --- |
| **Sutures + tacks** | 10 | 2211 | 2,75 (1,72-13,22)**^#^”** | 31,5 (27,75-38,25) |
| **Sutures only** | 2 | 1121 | 3,75(3,12-4,37)**^#^”** | 39(33,5-44,5) |
| **Tacks only** | 11 | 2473 | 6,35 (2,17-13,22)**^#^”** | 40(30,5-49,5) |

**^#^ Kruskal-Wallis Test: p = 0,845**

**“ANOVA: p= 0,747**

**Number of Tacks and postop.pain:**

Regarding correlation of postoperative pain and number of tacks used for mesh fixation a comparative study by Schoenmaeckers et al. {28} was performed. The assessment of pain by VAS revealed significant less pain (p= 0.001) at 3 months postoperatively in the group of 55% less tacks for fixation, whereas at 6 months no significant differences were found.

**Intervals of tacker fixation:**

Concerning the correlation of intervals of tacker fixation and recurrence rate 9 studies (Baccari et al. {30} Carbajo et al. {16} Ceccarelli et al.{29} Ferrari et al. {31}Morales et al. {20}Olmi et al. {22}Sharma et al. {8} Wassenaar et al. {10}) were selected and analysed (Tab.4).

The cumulative result of the mean intervals of tacker fixation was 1,5 cm (1-2) which correlated to a recurrence rate of 2,85% (2,1-3,8) at a follow up of 37 months (29-40) (Tab.4). The analysis of the different overlap of mesh revealed a mean of 4 cm (3,1-4,5).

**Comment**: The discussion of “are transfascial sutures necessary” in terms of strength of fixation and recurrences clinical {53,54,56,57} and experimental reports {69,71} were published. In conclusion the suture fixation achieves the highest tensile strength in comparison to alternative devices {69}. The implication made by Le Blanc is to use a combination of sutures and tacker fixation for minimizing the risk of recurrence.

**Tab.4**

| **Authors** | **Type of fixation SC(single crown)**  **DC (double crown** | **Intervals**  **cm** | **Overlap**  **cm** | **Recurrence rate in %** | **Follow up**  **month** |
| --- | --- | --- | --- | --- | --- |
| **Frantzides 2004 {18}** | SC | 1 | ≥ 3 | 1,4 | 24 |
| **Baccari 2009**  **{30}** | DC | 1 | 3-5 | 3,5 | 44 |
| **Carbajo 2003**  **{16}** | DC | 2 | 5 | 4,5 | 38 |
| **Ceccarelli 2008**  **{29}** | DC | 1-2 | 3-5 | 2,1 | 38 |
| **Ferrari 2008**  **{31}** | DC | 2 | 3-4 | 3,1 | 24 |
| **Olmi 2006**  **{22}** | DC | 2 | 4-5 | 2,6 | 29 |
| **Morales 2005**  **20}** | DC | 1 | ≥ 3 | 2,1 | 40 |
| **Sharma 2011**  **{8}** | SC/DC | 3 | 5 | 3,9 | 63 |
| **Wassenaar 2009**  **{10}** | DC | 1 | ≥ 3 | 1,9 | 31 |
| **total** |  | **1,5 ***  **(1-2)** | **4***  **(3,1-4,5)** | **2,85***  **(2,1-3,8)** | **37***  **(29-40)** |

* median (IQR

**Operation time – suture fixation vs tacker fixation:**

The correlation of type and time of fixation – suture versus tacker - was investigated in studies by Wassenaar et al.{23}**,**Bansal et al.{24} and Nguyen et al.{27}. In the randomized controlled trials of Wassenaar and Bansal the operation time in the suture group was significantly longer - 50.6 vs 41.1 min (p=0.002) and 77.5 vs 52.6 min. (p<0.0001). However in the prospective study of Nguyen et al. no significant difference between both groups was found.

**Permanent vs absorbable Suture Fixation and pain:**

Only one study published by Wassenaar et al. {23} analysed the correlation of suture material used for transfascial suture fixation and postoperative pain in a randomized controlled trial using absorbable sutures (Vicryl) versus nonabsorbable sutures (Mersilene). Comparing both groups no significant difference in terms of postoperative pain assessed by VAS (2 weeks, 6 weeks and 3 month postop.) was detected.

Concerning pain caused by suture sites a randomized study by Bellows et al.{65} was performed. Patients were randomized to receive local anesthesia (0.25% bupivacaine with epinephrine) into all layers of the abdominal wall to the level of the parietal peritoneum at suture fixation sites (non absorbable Gore-Tex sutures) immediately before suture placement and compared to a control group without local anesthesia The treated group had a statistically significant decrease in the postoperative pain scores (VAS: 0-10) at 1 hour postoperatively (2.2vs. 6.4; p<0.05). At the other timepoints (4 and 24 hours) the mean pain scores were decreased but not statistically significant.

**Fixation associated Complications:**

**Mesh Shrinkage:**

In the randomized controlled trial by Beldi et al. {25} tacker (Protack®, single crown technique, 2cm intervals) versus suture (polypropylene, 2-3cm intervals) fixation of a composite polyester mesh with an overlap of at least 5 cm was investigated by conventional abdominal X-ray examination in prone position, at the 2^nd^postoperative day, after 6 weeks and 6 months postoperatively respectively. A significant decrease of mesh size was detected in horizontal direction in the tacker group, whereas in vertical direction and mesh surface area no significant difference was found. In another study by Schoenmaeckers et al. {33} mesh shrinkage after double crown technique of ePTFE-meshes was investigated by CT measurements. At mean 17,9 month postoperatively shrinkage rate of 7,5% was found.

**Comment**: In conclusion, the suture fixation implies less risk of shrinkage in comparison to tacker only fixation. Taking into account the common shrinkage of all different mesh materials the overlap has to be estimated taking into account the correlation of mesh type and fixation technique.

**Fixation device induced incisional hernia:**

Several case reports, some recently, were published to cover the topic of fixation device induced incisional hernias. The first report in 2003 published by LeBlanc {34} referred to an incisional hernia in the site of a penetrating tacker fixation and described as a “tack hernia”. Further reports made by Muysoms et al {35}, Khandelwal et al. {37} and Barzana et al. {38} decribe incisional hernias after suture fixation.

The most severe complication of tacker fixation was reportedby Malmstroem et al. {36} leading to cardiac tamponade and finally to death.

**New fixation devices:**

**Resorbable fixation devices:**

Allthough resorbable devices for mesh fixation in LVHR have been available for some years, only one prospective multicenter clinical trial study by Lepere et al. {39}has been published. 29 patients in 11 centers were treated for incisional and umbilical hernia by LVHR. The mesh fixation was performed by I-Clip® (10 mm disposable instrument), which is resorbable within 1 year. Pain assessment by VAS (0-10) at 1 and 12 month revealed no pain at any timepoints.The recurrence rate at a follow up of 1 year was 0%.

Meanwhile the I-Clip® device was replaced by new resorbable tacker devices achieving higher tensile strength reported by Hollinsky et al.{40}.

In respect to the recently published experimental studies, new absorbable fixation devices (e.g. SorbaFix®, PermaFix®, AbsorbaTack®, Securestrap®) have been developed achieving a sufficient tensile fixation strengthin comparison to conventional non resorbable Tacker (Protack®) and transfascial suture repair {40,41} - randomized trials are required to verify these experimental results.

**Experimental articles:**

Hollinsky et al. {40} compared transfascial suture fixation versus tacker (non resorbable and resorbable) fixation in an experimental study in a rat model. Suture fixation achieved significant higher retention strength at 1 week and 2 months postop. in comparison to tacker (ProTack® , I-Clip®, AbsorbaTack®) fixation (8.7 N/cm2 versus 5.6 N/cm2 versus 5.7 N/cm2). Widespread anchorage of the mesh was achieved with ProTack® as well as AbsorbaTack®, whereas the I-Clip® obtained significant less retention strength than any other form of fixation at either time point because of poor tissue penetration. The incidence of adhesion formation was significantly higher in the ProTack® group than in any of the other groups (p<0.001) at all timepoints.

In an experimental pig study by Byrd et al. {41} the strength of tacker fixation in LVHR by double crown technique was investigated using the screw-type absorbable (SorbaFix®) and permanent (PermaFix®) fixation devices as well as titanium spiral tacks (TS-Protack®) in comparison to partial thickness polypropylene suture (PR) as a control group. The maximum pull-of forces were significantly higher (p< 0.001) in the Protack® group in comparison to all other groups (28.61 N versus 22.71 N (SF) and 16.98 N (PF) versus 20.83 N (PR)) respectively at 4 weeks postop. adhesions in the PF group were significantly less tenacious compared to the TS group (p = 0.01)

**Comment**: Summarized new permanent and resorbable fixation tacker devices achieve comparable results regarding tensile strength and less adhesion formation.

**Glue fixation:**

**Clinical studies:**

The first clinical report published by Olmi et al {42} in a prospective controlled trial included 40 Patients with a defect size 2-7cm in diameter, using diluted Tissucol® (50 U/ml Thrombin) by Duplotip®-application and temporary suture fixation . At a median follow up of 16 months neither hematoma, seroma, nor recurrences were detected. The painscore (VAS) after 7 days postoperatively was 0 in all patients.

Another case control study by Olmi et al. {43} included 19 patients with a defect size of <6cm in diameter. Again mesh fixation was performed by diluted Tissucol, applied by Duplotip®. In 2 cases transfascial suture fixation were added. No complication or recurrences were detected at mean follow up time of 20 months. The painscore (VAS 0-10) at 7 and 15 days postoperatively was 1 and at 30 days postoperatively 0.

Recently, Erikson et al {26} published a randomized controlled multicenter trial with inclusion of 40 patients suffering from an umbilical hernia defect of 1,5 to 5 cm. Patients were assigned randomly to FS (4 units/ml thrombin) or titanium tack fixation (double crown technique). In the FS group significant less pain (VAS; 0-100mm) on days 0–2 postop. in rest (median 19 versus 47 mm; p = 0·025) and during activity (38 versus 60 mm; p = 0·014) in comparison to the tacker group was measured. Patients in the FS group resumed normal daily activity earlier (after median 7 versus 18 days; p = 0·027) and reported significantly less discomfort.

**Comment**: In conclusion the mesh fixation in LVHR by fibrin sealant in small umbilical hernias(≤ 5 cm) was associated with less acute postoperative pain, discomfort and a shorter convalescence than tack fixation in the very short follow up of tendays. The results in the studies of Olmi {42,43} confirm the feasibility of glue fixation in small ventral hernias up to 7cm of defect size at a follow up of 16 and 20 months respectively without any recurrences*.*Thus these clinical results seems very promising further prospective studies with longer follow up are required

**Experimental articles:**

In several experimental studies published by Rieder et al. {44}, Clarke et al. {45}**,** Fortelny et al {46}, Eriksen et al. {47,78}, Melmanet al. {48}**,** Schug Pass et al. {49}, Eriksen et al. {50} and Jenkins et al.{66} mesh fixation by fibrin glue in comparison to sutures, tacks and combinations of fixation devices support the efficacy of glue fixation. In the study of Rieder et al.{44} the tangential detachment forces revealed that fibrin-glue attachment was not substantially different from that achieved with absorbable tacks (median Tension Force 7.8Newton (1.3 -15.8), but only when certain open porous meshes (e.g. polyvinylidene fluoride/polypropylene mesh or titanium-coated polypropylene mesh) were used. Another study by Clarke at al. {45} in pig model indicated that mesh fixation using fibrin glue has comparable tensile strength and adhesion rates to sutures with tacks. The combination of fibrin glue and tacker fixation showed similar biomechanical characteristics compared to the other groups at 4 weeks postop.

The glue fixation strength depends directly on the type of mesh used (e.g. resorbable or non resorbable coating and porosity) and the polymersation time of the glue (Jenkins et al.{66} Fortelny et al. {46}).

Additional experimental studies by Ladurner et al. {67,68} assessed cyanoacrylate glue for mesh fixation in LVHR in rabbits. In their first study {67} the fixation strength of polypropylene composite meshes with cyanoacrylate glue was equivalent to ePTFE mesh fixation with spiral tacks at 12 weeks postop. Whereas using only a polypropylene composite mesh in the second study the tensile strength analysis revealed significant less tensile strength of cyanoacrylate fixationin comparison to sutures or tacker fixation at 12 weeks postoperatively.

A drawback of glue fixation only technique in terms of migration and contraction described in the study of Schug Pass et al. {49} and Clarke et al. {45} should be an issue for the consideration of additional fixation (e.g. transfascial sutures or tacker) in the opposite to the results of Olmi et al. {42,43} and Eriksen et al. {26}.

**Comment**: Summarizing the experimental and clinical data, the use of glue, especially fibrin glue, in combination with penetrating fixation devices e.g. transfascial sutures or tacks seems to be feasible in terms of biomechanical strength presupposed the appropriate type of mesh (e.g.: porosity, elasticity, coating) is selected and use of glue application is adequate. The use of additional glue fixation increases the efficacy of mechanical fixation and leads to a possible reduction of penetrating and perforating devices {44,45} as well as the risk of postoperative pain respectively.

**References** (in parentheses graduation of evidence)

1. Kapischke M, Schulz T, Schipper T, Tensfeldt J, Caliebe A. Open versus laparoscopic incisional hernia repair: something different from a meta-analysis. Surg Endosc. 2008 Oct;22(10):2251-60. **(level 1A)**
2. Ballem N, Parikh R, Berber E, Siperstein A. Laparoscopic versus open ventral hernia repairs: 5 year recurrence rates. Surg Endosc. 2008 Sep;22(9):1935-40.**(level 4)**
3. Berger D, Bientzle M, Müller A. Postoperative complications afterlaparoscopic incisional hernia repair. Incidence and treatment. Surg Endosc. 2002Dec;16(12):1720-3. **(level 4)**
4. Bingener J, Buck L, Richards M, Michalek J, Schwesinger W, Sirinek K.Long-term outcomes in laparoscopic vs open ventral hernia repair. Arch Surg. 2007Jun;142(6):562-7. **(level 4)**
5. Franklin ME Jr, Gonzalez JJ Jr, Glass JL, Manjarrez A. Laparoscopic ventraland incisional hernia repair: an 11-year experience. Hernia. 2004 Feb;8(1):23-7. **(level 4)**
6. LeBlanc KA, Whitaker JM, Bellanger DE, Rhynes VK. Laparoscopic incisional andventral hernioplasty: lessons learned from 200 patients. Hernia. 2003Sep;7(3):118-24. **(level 4)**
7. McKinlay RD, Park A. Laparoscopic ventral incisional hernia repair: a moreeffective alternative to conventional repair of recurrent incisional hernia. JGastrointest Surg. 2004 Sep-Oct;8(6):670-4. **(level 4)**
8. Sharma A, Mehrotra M, Khullar R, Soni V, Baijal M, Chowbey PK. Laparoscopicventral/incisional hernia repair: a single centre experience of 1,242 patientsover a period of 13 years. Hernia. 2011 Apr;15(2):131-9. **(level 3)**
9. Topart P, Ferrand L, Vandenbroucke F, Lozach P. Laparoscopic ventral hernia repair with the Goretex Dualmesh: long-term results and review of the literature. Hernia. 2005 Dec;9(4):348-52. **(level 4)**
10. Wassenaar EB, Schoenmaeckers EJ, Raymakers JT, Rakic S. Recurrences afterlaparoscopic repair of ventral and incisional hernia: lessons learned from 505repairs. Surg Endosc. 2009 Apr;23(4):825-32. **(level 4)**
11. Yavuz N, Ipek T, As A, Kapan M, Eyuboglu E, Erguney S. Laparoscopic repair ofventral and incisional hernias: our experience in 150 patients. J LaparoendoscAdv Surg Tech A. 2005 Dec;15(6):601-5. **(level 4)**
12. Chelala E, Thoma M, Tatete B, Lemye AC, Dessily M, Alle JL. The suturingconcept for laparoscopic mesh fixation in ventral and incisional hernia repair:Mid-term analysis of 400 cases. Surg Endosc. 2007 Mar;21(3):391-5. **(level 4)**
13. Palanivelu C, Jani KV, Senthilnathan P, Parthasarathi R, Madhankumar MV,Malladi VK. Laparoscopic sutured closure with mesh reinforcement of incisionalhernias. Hernia. 2007 Jun;11(3):223-8. **(level 4)**
14. Bencini L, Sanchez LJ, Boffi B, Farsi M, Martini F, Rossi M, Bernini M, Moretti R. Comparison of laparoscopic and open repair for primary ventralhernias. Surg Laparosc Endosc Percutan Tech. 2009 Aug;19(4):341-4. **(level 4)**
15. Bageacu S, Blanc P, Breton C, Gonzales M, Porcheron J, Chabert M, Balique JG. Laparoscopic repair of incisional hernia: a retrospective study of 159 patients. Surg Endosc. 2002 Feb;16(2):345-8. **(level 4)**
16. Carbajo MA, del Olmo JC, Blanco JI, de la Cuesta C, Martín F, Toledano M, Perna C, Vaquero C. Laparoscopic treatment of ventral abdominal wall hernias:preliminary results in 100 patients. JSLS. 2000 Apr-Jun;4(2):141-5. **(level 4)**
17. Chowbey PK, Sharma A, Khullar R, Mann V, Baijal M, Vashistha A. Laparoscopic ventral hernia repair. J Laparoendosc Adv Surg Tech A. 2000 Apr;10(2):79-84.**(level 4)**
18. Frantzides CT, Carlson MA, Zografakis JG, Madan AK, Moore RE. Minimallyinvasive incisional herniorrhaphy: a review of 208 cases. Surg Endosc. 2004Oct;18(10):1488-91. **(level 4)**
19. Kirshtein B, Lantsberg L, Avinoach E, Bayme M, Mizrahi S. Laparoscopic repaof large incisional hernias. Surg Endosc. 2002 Dec;16(12):1717-9. **(level 4)**
20. Morales-Conde S, Cadet H, Cano A, Bustos M, Martín J, Morales-Mendez S. Laparoscopic ventral hernia repair without sutures--double crown technique: ourexperience after 140 cases with a mean follow-up of 40 months. Int Surg. 2005Jul-Aug;90(3 Suppl):S56-62. **(level 4)**
21. Moreno-Egea A, Cartagena J, Vicente JP, Carrillo A, Aguayo JL. Laparoscopicincisional hernia repair as a day surgery procedure: audit of 127 consecutivecases in a university hospital. Surg Laparosc Endosc Percutan Tech. 2008 Jun;18(3):267-71. **(level 4)**
22. Olmi S, Erba L, Magnone S, Bertolini A, Croce E. Prospective clinical studyof laparoscopic treatment of incisional and ventral hernia using a compositemesh: indications, complications and results. Hernia. 2006 Jun;10(3):243-7. **(level 3)**
23. Wassenaar E, Schoenmaeckers E, Raymakers J, van der Palen J, Rakic S. Mesh-fixation method and pain and quality of life after laparoscopic ventral orincisional hernia repair: a randomized trial of three fixation techniques. SurgEndosc. 2010 Jun;24(6):1296-302. **(level 1B)**
24. Bansal VK, Misra MC, Kumar S, Rao YK, Singhal P, Goswami A, Guleria S, AroraMK, Chabra A. A prospective randomized study comparing suture mesh fixationversus tacker mesh fixation for laparoscopic repair of incisional and ventralhernias. Surg Endosc. 2011 May;25(5):1431-8. **(level 1B)**
25. Beldi G, Wagner M, Bruegger LE, Kurmann A, Candinas D. Mesh shrinkage and pain in laparoscopic ventral hernia repair: a randomized clinical trial comparingsuture versus tack mesh fixation. Surg Endosc. 2011 Mar;25(3):749-55. **(level 1B)**
26. Eriksen JR, Bisgaard T, Assaadzadeh S, Jorgensen LN, Rosenberg J. Randomizedclinical trial of fibrin sealant versus titanium tacks for mesh fixation inlaparoscopic umbilical hernia repair. Br J Surg. 2011 Nov;98(11):1537-45.**(level 1B)**
27. Nguyen SQ, Divino CM, Buch KE, Schnur J, Weber KJ, Katz LB, Reiner MA, Aldoroty RA, Herron DM. Postoperative pain after laparoscopic ventral herniarepair: a prospective comparison of sutures versus tacks. JSLS. 2008Apr-Jun;12(2):113-6. **(level 1B)**
28. Schoenmaeckers EJ, de Haas RJ, Stirler V, Raymakers JT, Rakic S. Impact ofthe number of tacks on postoperative pain in laparoscopic repair of ventralhernias: do more tacks cause more pain? Surg Endosc. 2012 Fe;26(2):357-60**(level 3)**
29. Ceccarelli G, Patriti A, Batoli A, Bellochi R, Spaziani A, Pisanelli MC, Casciola L. Laparoscopic incisional hernia mesh repair with the "double-crown"technique: a case-control study. J Laparoendosc Adv Surg Tech A. 2008Jun;18(3):377-82. **(level 4)**
30. Baccari P, Nifosi J, Ghirardelli L, Staudacher C. Laparoscopic incisional and ventral hernia repair without sutures: a single-center experience with 200 cases.J Laparoendosc Adv Surg Tech A. 2009 Apr;19(2):175-9. **(level 4)**
31. Ferrari GC, Miranda A, Sansonna F, Magistro C, Di Lernia S, Maggioni D, Franzetti M, Pugliese R. Laparoscopic management of incisional hernias > or = 15cm in diameter. Hernia. 2008 Dec;12(6):571-6. **(level 4)**
32. Wassenaar EB, Raymakers JT, Rakic S. Impact of the mesh fixation technique on operation time in laparoscopic repair of ventral hernias. Hernia. 2008 Feb;12(1):23-5. **(level 4)**
33. Schoenmaeckers EJ, van der Valk SB, van den Hout HW, Raymakers JF, Rakic S. Computed tomographic measurements of mesh shrinkage after laparoscopic ventralincisional hernia repair with an expanded polytetrafluoroethylene mesh. SurgEndosc. 2009 Jul;23(7):1620-3. **(level 4)**
34. LeBlanc KA. Tack hernia: a new entity. JSLS. 2003 Oct-Dec;7(4):383-7. **(level 4)**
35. Muysoms FE, Cathenis KK, Claeys DA. "Suture hernia": identification of a new type of hernia presenting as a recurrence after laparoscopic ventral herniarepair. Hernia. 2007 Apr;11(2):199-201. **(level 4)**
36. Malmstrøm ML, Thorlacius-Ussing O. Cardiac tamponade as a rare complicationin laparoscopic incisional hernia repair. Hernia. 2010 Aug;14(4):421-2. **(level4)**
37. Khandelwal RG, Bibyan M, Reddy PK. Transfascial suture hernia: a rare form ofrecurrence after laparoscopic ventral hernia repair. J Laparoendosc Adv Surg TechA. 2010 Nov;20(9):753-5. **(level 4)**
38. Barzana D, Johnson K, Clancy TV, Hope WW. Hernia recurrence through acomposite mesh secondary to transfascial suture holes. Hernia. 2010 Sep 12. [Epubahead of print] PubMed PMID: 20835907. **(level 4)**
39. Lepere M, Benchetrit S, Bertrand JC, Chalbet JY, Combier JP, Detruit B,Herbault G, Jarsaillon P, Lagoutte J, Levard H, Rignier P. Laparoscopicresorbable mesh fixation. Assessment of an innovative disposable instrumentdelivering resorbable fixation devices: I-Clip(TM). Final results of aprospective multicentre clinical trial. Hernia. 2008 Apr;12(2):177-83. **(level 4)**
40. Hollinsky C, Kolbe T, Walter I, Joachim A, Sandberg S, Koch T, Rülicke T, Tuchmann A. Tensile strength and adhesion formation of mesh fixation systems usedin laparoscopic incisional hernia repair. Surg Endosc. 2010 Jun;24(6):1318-24. **(level 5)**
41. Byrd JF, Agee N, Swan RZ, Lau KN, Heath JJ, Mckillop IH, Sindram D, Martinie JB, Iannitti DA. Evaluation of absorbable and permanent mesh fixation devices: adhesion formation and mechanical strength. Hernia. 2011 Oct;15(5):553-8. **(level 5)**
42. Olmi S, Scaini A, Erba L, Croce E. Use of fibrin glue (Tissucol) inlaparoscopic repair of abdominal wall defects: preliminary experience. SurgEndosc. 2007 Mar;21(3):409-13. **(level 3)**
43. Olmi S, Cesana G, Sagutti L, Pagano C, Vittoria G, Croce E. Laparoscopic incisional hernia repair with fibrin glue in select patients. JSLS. 2010 Apr-Jun;14(2):240-5. Erratum in: JSLS. 2011 Jul-Sep;15(3):430. **(level 4)**
44. Rieder E, Stoiber M, Scheikl V, Poglitsch M, Dal Borgo A, Prager G, Schima H. Mesh fixation in laparoscopic incisional hernia repair: glue fixation providesattachment strength similar to absorbable tacks but differs substantially indifferent meshes. J Am Coll Surg. 2011 Jan;212(1):80-6. **(level 5)**
45. Clarke T, Katkhouda N, Mason RJ, Cheng BC, Algra J, Olasky J, Sohn HJ,Moazzez A, Balouch M. Fibrin glue for intraperitoneal laparoscopic mesh fixation:a comparative study in a swine model. Surg Endosc. 2011 Mar;25(3):737-48. **(level 5)**
46. Fortelny RH, Petter-Puchner AH, Ferguson J, Gruber-Blum S, Brand J, Mika K, Redl H. A comparative biomechanical evaluation of hernia mesh fixation by fibrin sealant. J Surg Res. 2011 Dec;171(2):576-81. **(level 5)**
47. Eriksen JR, Bech JI, Linnemann D, Rosenberg J. Laparoscopic intraperitonealmesh fixation with fibrin sealant (Tisseel) vs. titanium tacks: a randomisedcontrolled experimental study in pigs. Hernia. 2008 Oct;12(5):483-91. **(level 4)**
48. Melman L, Jenkins ED, Deeken CR, Brodt MD, Brown SR, Brunt LM, Eagon JC,Frisella M, Matthews BD. Evaluation of acute fixation strength for mechanicaltacking devices and fibrin sealant versus polypropylene suture for laparoscopicventral hernia repair. Surg Innov. 2010 Dec;17(4):285-90.**(level 5)**
49. Schug-Pass C, Lippert H, Köckerling F. Fixation of mesh to the peritoneum usingfibrin glue: investigations with a biomechanical model and an experimental laparoscopic porcine model. Surg Endosc. 2009 Dec;23(12):2809-15. **(level 5)**
50. Jenkins ED, Melman L, Frisella MM, Deeken CR, Matthews BD. Evaluation ofacute fixation strength of absorbable and nonabsorbable barrier coated meshsecured with fibrin sealant. Hernia. 2010 Oct;14(5):505-9.**(level 5)**
51. Schoenmaeckers EJ, Wassenaar EB, Raymakers JT, Rakic S. Bulging of the meshafter laparoscopic repair of ventral and incisional hernias. JSLS. 2010Oct-Dec;14(4):541-6. **(level 4)**
52. Wolter A, Rudroff C, Sauerland S, Heiss MM. Laparoscopic incisional herniarepair: evaluation of effectiveness and experiences. Hernia. 2009Oct;13(5):469-74. **(level 3)**
53. LeBlanc KA. Laparoscopic incisional hernia repair: are transfascial suturesnecessary? A review of the literature. Surg Endosc. 2007 Apr;21(4):508-13. **(level 1A)**
54. LeBlanc KA, Booth WV, Whitaker JM, Bellanger DE. Laparoscopic incisional and ventral herniorraphy: our initial 100 patients. Hernia. 2001 Mar;5(1):41-5.**(level 4)**
55. Berger D, Bientzle M, Müller A. [Laparoscopic repair of incisional hernias]. Chirurg. 2002 Sep;73(9):905-8. **(level 4)**
56. LeBlanc KA. Incisional hernia repair: laparoscopic techniques. World J Surg. 2005 Aug;29(8):1073-9. Review. **(level1B)**
57. LeBlanc KA. Laparoscopic incisional and ventral hernia repair:complications-how to avoid and handle. Hernia. 2004 Dec;8(4):323-31. Review.
58. Heniford BT, Park A, Ramshaw BJ, Voeller G. Laparoscopic repair of ventralhernias: nine years' experience with 850 consecutive hernias. Ann Surg. 2003Sep;238(3):391-9; **(level 4)**
59. Perrone JM, Soper NJ, Eagon JC, Klingensmith ME, Aft RL, Frisella MM, BruntLM. Perioperative outcomes and complications of laparoscopic ventral herniarepair. Surgery. 2005 Oct;138(4):708-15 **(level 4)**
60. Cobb WS, Kercher KW, Matthews BD, Burns JM, Tinkham NH, Sing RF, Heniford BT. Laparoscopic ventral hernia repair: a single center experience. Hernia. 2006Jun;10(3):236-42. **(level 4)**
61. Olmi S, Scaini A, Cesana GC, Erba L, Croce E. Laparoscopic versus open incisional hernia repair: an open randomized controlled study. Surg Endosc. 2007 Apr;21(4):555-9. **(level 1B)**
62. Olmi S, Magnone S, Erba L, Bertolini A, Croce E. Results of laparoscopicversus open abdominal and incisional hernia repair. JSLS. 2005 Apr-Jun;9(2):189-95. **(level 2B)**
63. Chelala E, Gaede F, Douillez V, Dessily M, Alle JL. The suturing concept for laparoscopic mesh fixation in ventral and incisional hernias: preliminaryresults. Hernia. 2003 Dec;7(4):191-6. **(level 4)**
64. Eriksen JR, Poornoroozy P, Jørgensen LN, Jacobsen B, Friis-Andersen HU, Rosenberg J. Pain, quality of life and recovery after laparoscopic ventral herniarepair. Hernia. 2009 Feb;13(1):13-21. **(level 4)**
65. Bellows CF, Berger DH. Infiltration of suture sites with local anesthesia for management of pain following laparoscopic ventral hernia repairs: a prospective randomized trial. JSLS. 2006 Jul-Sep;10(3):345-50.**(level 1B)**
66. Jenkins ED, Melman L, Desai S, Brown SR, Frisella MM, Deeken CR, Matthews BD.Evaluation of intraperitoneal placement of absorbable and non-absorbable barrier-coated mesh secured with fibrin sealant in a New Zealand white rabbit model. SurgEndosc. 2011 Feb;25(2):604-12. **(level 5)**
67. Ladurner R, Drosse I, Bürklein D, Plitz W, Barbaryka G, Kirchhoff C, Kirchhoff S, Mutschler W, Schieker M, Mussack T. Cyanoacrylate glue forintra-abdominal mesh fixation of polypropylene-polyvinylidene fluoride meshes in a rabbit model. J Surg Res. 2011 May 15;167(2):e157-62. **(level 5)**
68. Ladurner R, Drosse I, Seitz S, Plitz W, Barbaryka G, Siebeck M, Bürklein D, Kirchhoff C, Buhman S, Mutschler W, Schieker M, Mussack T. Tissue attachment strength and adhesion formation of intraabdominal fixed meshes with cyanoacrylat glues. Eur J Med Res. 2008 May 26;13(5):185-91. **(level 5)**
69. van't Riet M, de Vos van Steenwijk PJ, Kleinrensink GJ, Steyerberg EW, Bonjer HJ. Tensile strength of mesh fixation methods in laparoscopic incisional herniarepair. Surg Endosc. 2002 Dec;16(12):1713-6. **(level 5)**
70. Lerdsirisopon S, Frisella MM, Matthews BD, Deeken CR. Biomechanicalevaluation of potential damage to hernia repair materials due to fixation withhelical titanium tacks. Surg Endosc. 2011 Dec;25(12):3890-7. **(level 5)**
71. Winslow ER, Diaz S, Desai K, Meininger T, Soper NJ, Klingensmith ME. Laparoscopic incisional hernia repair in a porcine model: what do transfixionsutures add? Surg Endosc. 2004 Mar;18(3):529-35.**(level 5)**
72. Koehler RH, Voeller G. Recurrences in laparoscopic incisional hernia repairs:a personal series and review of the literature. JSLS. 1999 Oct-Dec;3(4):293-304. **(level 4)**
73. Cobb WS, Kercher KW, Heniford BT. Laparoscopic repair of incisional hernias. Surg Clin North Am. 2005 Feb;85(1):91-103. Review. **(level 1A)**
74. Rudmik LR, Schieman C, Dixon E, Debru E. Laparoscopic incisional herniarepair: a review of the literature. Hernia. 2006 Apr;10(2):110-9. **(level 1A)**
75. Bedi AP, Bhatti T, Amin A, Zuberi J. Laparoscopic incisional and ventral hernia repair. J Minim Access Surg. 2007 Jul;3(3):83-90. Review**(level 1A)**
76. Sauerland S, Walgenbach M, Habermalz B, Seiler CM, Miserez M. Laparoscopic versus open surgical techniques for ventral or incisional hernia repair. CochraneDatabase Syst Rev. 2011 Mar 16;(3) **(level 1A)**
77. Brill JB, Turner PL. Long-term outcomes with transfascial sutures versustacks in laparoscopic ventral hernia repair: a review. Am Surg. 2011Apr;77(4):458-65. Review. **(level 1A)**
78. Eriksen JR. Fixation of mesh to the peritoneum using a fibrin glue: investigations with a biomechanical model and an experimental laparoscopic porcine model. Surg Endosc. 2010 Jun;24(6):1501-2; **(level 5)**

**Fixation in suprapubic and subxiphoidal hernias**

R.H.Fortelny, M. Misra, F. Köckerling

Search terms: “laparoscopic hernia repair“ AND “LVHR“ AND “incisional hernia“ AND “suprapubic hernia“ AND “parapubic hernia” AND “subxiphoidal hernia“AND “fixation“AND “tacks“ AND “staples“ AND “recurrences” AND “pain” AND “long term results”

A systemic search of the available literature was performed in August 2011 using Medline, PubMed, Cochrane library and relevant journals and reference lists

The first search detected 19 relevant articles. In a second-level search 2 articles were added. In summary this review is based on 21 articles including 11 studies.

Key Question:

**How to fix the mesh in suprapubic and subxiphoidal hernias?**

The specification of the term suprapubic hernia is defined by Carbonell et al. {3} and Palanivelu et al. {6} ashernia defect located 3-4 cm above the symphysis pubis and by the EHS-classification {22} as hernia M5.The most common cause of suprapubic hernia is a postoperative incisional hernia (e.g. suprapubic radical prostatectomy {13}).Congenital malformations of the pelvis are a very rare issue {12}.

**Statements:**

| Level 4 | A retropubic dissection is necessary to achieve sufficient and safe mesh overlap of the suprapubic defect as well as an effective fixation.  A combination of mesh fixation by sutures and tacks includingfixation at Cooper’s ligament and a sufficient mesh overlap is associated with a low recurrence rate. |
| --- | --- |

**Recommendation:**

| Grade C | For safe positioning and sufficient overlap of mesh the retropubic space should be dissected.  The mesh fixation should include Cooper’s ligament preferably by penetrating devices. |
| --- | --- |

**Introduction:**

The first report of a mesh inforced repair of an incisional parapubic hernia was published by Bendavid in 1990 {1}. In an open approach via dissection of the space of Retzius and laterally thereof a polypropylene mesh (Marlex) was anchored to the ligaments of Cooperi and the arcuate pubic ligament by interrupted non absorbable sutures. Seven patients were treated in this specific technique which correlates to the laparoscopic technique used later on.

**Fixation in suprapubic hernia:**

In 1999 Matuszewski et al. {2} reported the first laparoscopic repair of an incisional suprapubic hernia 9 months postop.after suprapubic radical prostatectomy using a polypropylene mesh and fixation by clips.

Up to August 2011three case series {3,4,5} and four retrospective studies {6,7,8,9} regarding laparoscopicrepair in suprapubic hernia were published (Tab.1).

Hirasa et al {3} treated suprapubic hernias laparoscopically in 7 patients without dissection of the space of Retzius using dualsurface mesh with an overlap of 2-3 cm and fixation by tacks. At a mean follow up of 5,8 months 1 recurrence was detected.

All other studies describe a complete dissection of the retropubic space for appropriate mesh positioning and overlap. The overlap of mesh reported is at least 4 to 5 cm. Most fixation techniques are based on a combination of sutures and tacks. A new technique was described by Palanivelu et al. {6} performing a complete closure of the hernia defect by running sutures and mesh fixation (overlap of 5cm) by pretied and intracorporal sutures with 4-5 cm intervals circumferentially. Postoperative pain occurred in the studies of Carbonell et al. {5}, Palanivelu et al. {6}, Varnell et al.{7} and Sharma et al. {9} in a wide range of 2,7 – 9,7 %, possibly due to tight transfascial sutures. The highest number of patients (72) were analysed retrospectively by Sharma et al. {9} treated by a combination of devices (transfascial sutures and tacks) for mesh fixation with an overlap of 5 cm. At the longest follow up time of all studies of 4,9 years a recurrence rate of 0% and postop. pain of 9,7% occurred. In terms of a safe fixation of the mesh Carbonell et al. {10} reported a novel method of using a bone anchor eligible also for fixation in the region of the pubic bone in suprapubic hernia repair {11}.

The recurrence rate analyzed in a total number of 215 patients (included all studies) yield median 5,5% (2,7-6,0) at a follow up time of 21,1 month (13-36)(Tab1). The incidence of postop.pain was median 4,9 % (3,8-6,6)(Tab.1).

**Tab 1.**

| Author | Type of study | Pat. | Retro-pubic diss. | Overlap  of  mesh  cm | Type of fixation | Pain  % | Recurr.  rate  % | Follow  up  month | Level  of evidence |
| --- | --- | --- | --- | --- | --- | --- | --- | --- | --- |
| Hirasa  et al. 2001{3} | case-series | 7 | no | 2-3 | t | 0 | 14 | 5,8 | IV |
| Carbonell et al. 2005{4} | case-series | 36 | yes | 4-5 | ts | 2,7 | 5,5 | 21,1 | IV |
| McKay  et al. 2001{5} | case-series | 8 | yes | 3-4 | ts | - | 0 | 17,5 | IV |
| Palanivelu et al. 2008{6} | retro-spect. | 17 | yes | 5 | s^a^ | 5,8 | 5,8 | 9 | IV |
| Varnell  et al. 2008{7} | retro-spect. | 47 | yes | 4-5 | ts | 4,2 | 6,3 | 36 | IV |
| Ferrari  et al. 2009{8} | retro-spect. | 18 | yes | 4-5 | t | - | 5,5 | 37 | IV |
| Sharma  et al 2011{9} | retro-spect. | 72 | yes | 5 | ts | 9,7 | 0 | 57,6 | IV |
| total |  | 215 |  | 4,5*  (4-4,75) |  | 4,9*  (3,8-6,6) | 5,5*  (2,7-6,0) | 21,1*  (13-36) |  |

Tacks(t), Sutures (s) , Tacks+ sutures (ts)

^a^Closure of the defect by sutures

* median (IQR)

**Comment:**

Concerning operation technique in laparoscopic and open approach {14} it is common sense to perform a retropubic dissection for sufficient and safe overlap of the mesh. Without dissection the recurrence rate (Hirasa et al. {3}) seems to be significant higher. Combination of suture and tack fixation is associated with a low recurrence rate of median 5,5% at a mean follow up of 21,1 months. One study with closure of the defect and mesh fixation by suture only technique by Palanivelu et al. {9} obtained a similar recurrence rate of 5,8% at a follow up of 36 month, compared to the fixation by tacks only {3}.

**Fixation in subxiphoid hernia**

**Introduction:**

The specification of the term subxiphoid hernia is defined by the EHS-classification {22} as hernia M1. The reported incidence of subxiphoid incisional hernias after median sternotomy is between 1% and 4,2%{19}. Different types of open repair techniques (e.g. onlay mesh , sublay) are described {19} and since 2000 a laparoscopic techniqueis reported {20}.

In the technical considerations for the repair of subxyphoidal hernia Conze et al. {21} describes the importance of the landmarks for appropriate dissection of the retroxiphoidal space. Starting from the dorsal side of the xiphoid process, fatty tissue should be mobilized by blunt dissection followed by further detachment of the sternal portion of the diaphragm and finally separating the pericardium from the sternum. This special technique is mandatory independent of open or laparoscopic approach to achieve an opening of an extended retroxiphoidal space for safe and appropriate mesh positioning and sufficient overlap.

**Statements:**

| Level 4 | The dissection of the extended retroxiphoidal space up to 5cm behind the xiphoid process is mandatory for appropriate mesh positioning and overlap.  The fixation in the cephalad portion of the mesh has a high risk of a lesion to the pericard |
| --- | --- |

**Recommendation:**

| Grade C | The overlap of the mesh should be sufficient especially in the cephalad retroxiphoidal space.  The cephalad part of the mesh should be left without fixation. |
| --- | --- |

Only 4 studies - 1 retrosp.comp.study {16} and 3 retrospective studies {17,18, 8} are available for the analysis in this topic.

Muscarella et al. {20} in 2000 published the first report of a laparoscopic repair of a subxiphoidal hernia. A bi-layer permanent composite mesh was used and 4 transmural corner stitches and tacker to the posterior rectus abdominis sheath achieved the fixation.The first case series of Landau et al.{17} in 2001 included 10 patients repaired laparoscopically. For mesh fixation 3 pretied stay sutures and tacks were used.1 patient suffered from a recurrenceat a follow up 20-24 months.

Mackey et al.{16} performed aretrospect.comp.study concerning the risk of incisional hernia after median sternotomy for cardiothoracic procedure. In the hernia group 45 patients were enrolled for treatment - 35 in open approach (14 suture repair, 21 open-mesh repair) and10 laparoscopically. There are no details concerning specific technique used. At a mean follow up of 48 months in 3 patients - 1 patient after sternal wound infection -2 recurrences were detected.

In a case study published by Eisenberg et al. {18}4 patients - 3 with recurrent hernia after open repair - were included. Performing a mesh overlap of 3 cm fixated by 6-8 sutures and tacks omitting the cephalad portion 0 recurrences occurred at a follow up of 6 month.

In another retrospective study by Ferrari et al. 15 patientswere included (3 with recurrent hernia) and the mesh fixation was performed only with intracorporal suture to the peritoneal layer or xiphoidal periostium omitting the cephalad part of the mesh. The recurrence rate was 6,6% (1patient) at a follow up of 37 months.

The analysis of the total number of 39 patients revealed a recurrence rate of median 8,3%(4,95-15) at a follow up time of 29,5(18-39,75) months (Tab.2).

Tab.2

| Author | Type of study | Pat. | Over-  lap  cm | Type of fixation  Tacks(t)  Sutures (s)  Tacks+ sutures (ts) | Recurr.  rate  % | Follow  up  month | Level  of  evidence |
| --- | --- | --- | --- | --- | --- | --- | --- |
| Landau et al 2001 | retro-spective | 10 | - | ts | 10 | 20-24 | IV |
| Mackey et al 2005 | retro-spective | 10 | - | - | 30 | 48 | III |
| Eisenberg et al. 2008 | case- series | 4 | 3 | ts | 0 | 6 | IV |
| Ferrari et al. 2009 | retro-spective | 15 | - | s | 6,6 | 37 | IV |
| total |  | 39 |  |  | 8,3*  (4,95-15) | 29,5*  (18-39,75) |  |

Tacks(t), Sutures (s) , Tacks+ sutures (ts)

* median (IQR)

**Comment:**

Thus a low numbers of studiesand a low evidence of technique are available the mesh overlap especially in the cephalad retroxiphoidal space has to be adequate and protruding fixation in this region has to be omitted.

**References (in parentheses graduation of evidence)**

1. Bendavid R. Incisional parapubic hernias. Surgery. 1990 Nov;108(5):898-90**{level 4)**
2. Matuszewski M, Stanek A, Maruszak H, Krajka K. Laparoscopic treatment ofparapubic postprostatectomy hernia. Eur Urol. 1999 Nov;36(5):418-20. **{level 4}**
3. Hirasa T, Pickleman J, Shayani V. Laparoscopic repair of parapubic hernia.Arch Surg. 2001 Nov;136(11):1314-7**. {level 4}**
4. McKay R, Haupt D. Laparoscopic repair of low abdominal wall hernias by tackfixation to the cooper ligament. Surg Laparosc Endosc Percutan Tech. 2006Apr;16(2):86-90. **{level 4}**
5. Carbonell AM, Kercher KW, Matthews BD, Sing RF, Cobb WS, Heniford BT. Thelaparoscopic repair of suprapubic ventral hernias. Surg Endosc. 2005Feb;19(2):174-7. **{level4}**
6. Palanivelu C, Rangarajan M, Parthasarathi R, Madankumar MV, Senthilkumar K.Laparoscopic repair of suprapubic incisional hernias: suturing andintraperitoneal composite mesh onlay. A retrospective study. Hernia. 2008Jun;12(3):251-6. **{level 4}**
7. Varnell B, Bachman S, Quick J, Vitamvas M, Ramshaw B, Oleynikov D. Morbidityassociated with laparoscopic repair of suprapubic hernias. Am J Surg. 2008Dec;196(6):983-7**; {level 4}**
8. Ferrari GC, Miranda A, Sansonna F, Magistro C, Di Lernia S, Maggioni D,Franzetti M, Costanzi A, Pugliese R. Laparoscopic repair of incisional herniaslocated on the abdominal borders: a retrospective critical review. Surg Laparosc Endosc Percutan Tech. 2009 Aug;19(4):348-52. **{level 4}**
9. Sharma A, Dey A, Khullar R, Soni V, Baijal M, Chowbey PK. Laparoscopic repair of suprapubic hernias: transabdominal partial extraperitoneal (TAPE) technique.Surg Endosc. 2011 Jul;25(7):2147-52. Epub 2010 Dec 24**. {level 4}**
10. Carbonell AM, Kercher KW, Sigmon L, Matthews BD, Sing RF, Kneisl JS, Heniford BT. A novel technique of lumbar hernia repair using bone anchor fixation. Hernia. 2005 Mar;9(1):22-5**.(level 5)**
11. Craft RO, Harold KL. Laparoscopic repair of incisional and other complexabdominal wall hernias. Perm J. 2009 Summer;13(3):38-42. **{level 5}**
12. Moreno-Egea A, Campillo-Soto A, la Calle MC, Torralba-Martínez JA, Girela E,Albasini JL. Incisional pubic hernia: treatment of a case with congenitalmalformation of the pelvis. Hernia. 2006 Mar;10(1):87-9. **{level 4}**
13. Norris JP, Flanigan RC, Pickleman J. Parapubic hernia following radicalretropubic prostatectomy. Urology. 1994 Dec;44(6):922-3. PubMed PMID: 7985326.
14. Yao S, Li JY. Treatment for incisional parapubic hernia: an experience of 25cases. Am Surg. 2010 Dec;76(12):1420-2. **{level 4}**
15. Cohen MJ, Starling JR. Repair of subxiphoid incisional hernias with Marlexmesh after median sternotomy. Arch Surg. 1985 Nov;120(11):1270-1 **{level 4}**
16. Mackey RA, Brody FJ, Berber E, Chand B, Henderson JM. Subxiphoid incisionalhernias after median sternotomy. J Am Coll Surg. 2005 Jul;201(1):71-6. **{level 3}**
17. Landau O, Raziel A, Matz A, Kyzer S, Haruzi I. Laparoscopic repair ofpoststernotomy subxiphoid epigastric hernia. Surg Endosc. 2001 Nov;15(11):1313-4.Epub 2001 Aug 16. **{level 4}**
18. Eisenberg D, Popescu WM, Duffy AJ, Bell RL. Laparoscopic treatment ofsubxiphoid incisional hernias in cardiac transplant patients. JSLS. 2008Jul-Sep;12(3):262-6. **{level 4}**
19. Losanoff JE, Basson MD, Laker S, Weiner M, Webber JD, Gruber SA. Subxiphoid incisional hernias after median sternotomy. Hernia. 2007 Dec;11(6):473-9**.{level 2A}**
20. Muscarella P, Needleman BJ, Goldstein AH, Steinberg SM (2000)Laparoscopic repair of a subxiphoid incisional hernia followingmedian sternotomy. Surg Rounds 23:605–611 **{level 4}**
21. Conze J, Prescher A, Kisielinski K, Klinge U, Schumpelick V. Technicalconsideration for subxiphoidal incisional hernia repair. Hernia. 2005Mar;9(1):84-7. **{level 5}**

**MESH INSERTION**

M.C. Misra, V. K. Bansal, P.P. Prakash, D. Babu, P. Singhal, R.H. Fortelny

Search Engine used: Pubmed, Cochrane database, Medline and relevant journals and reference lists in the English language:

Mesh introduction/insertion AND “laparoscopic” AND “incisional hernia” AND “ventral hernia repair”

86 studies (level 3, 4, and 5) described the technique of mesh insertion. Only 12 of them primarily aimed to study mesh insertion technique. In 76 studies (> 6000 patients) mesh was inserted through 10 mm/12 mm port.

Theodoropoulou et al [1] described mesh insertion through the 10 mm ballon port or ballon port site. Hussain A et al [2] used a separate 10 to 15 mm port for mesh insertion at the center of the hernia, after reduction of the contents. Introduction of port at this site is very easy because there is only skin, subcutaneous tissue and peritoneum, while the muscle layer is attenuated.

Perry et al [3] used a 2-3 cm incision over the hernia site, in cases where there was incarcerated omentum, which could not be safely reduced. The omentum in such cases was carefully transected at the level of the abdominal wall and the hernia sac was incised to allow the complete dissection and excision of the previously identified incarcerated omentum, an appropriately sized piece of prosthetic mesh is prepared and inserted into the abdomen via the opened hernia sac.

Perrone et al [4], Nimeri et al [5] and Agrawal et al [6] also used similar skin incision over the defect for mesh insertion.

Carlson et al [7] described a technique of introduction of large mesh with stay sutures, slid into plastic sleeve and through the 10 mm trocar site without having the mesh come into contact with the skin.

The mesh itself should be treated in the same fashion as any vascular graft, in that any contact with the skin should be avoided (6,7,8). To avoid contact with the skin the mesh could be inserted with the help of a plastic sleeve (7).

Leiberman et al [9] rolled the mesh along its long axis and after every one-third roll a 4-0 chromic catgut suture placed around the roll. The mesh was then inserted through 10 mm trocar or 10 mm port site, if the mesh was too large.

**Rolling techniques and mesh introduction**

Walter et al [10] compared four specified insertion technique - Simple roll, a tight roll along the longest edge; Diagonal roll,a tight roll along the longest axis; Roll and bind, the optimal roll with an additional vicryl tie as binding; and Unprepared, grasped by the corner, the diagonal length of the mesh is presented head-on to the port. They documented the optimum insertion technique and minimum port sizes realistically needed for insertion of different types and sizes of mesh. They noted that the roll and bind technique allows optimal maximum mesh width (cm) to minimum port size (mm) ratio (M: P ratio) to be obtained from biological meshes as it overcomes their tendency to lose their roll. No advantage to using the roll and bind insertion technique was found with respect to the synthetic meshes nor was any value for rolling along the diagonal axis found for any mesh.

**Statements**

| Level 3 | Mesh insertion (up to 30 x 30 cm) through 10 – 12 mm port possible in majority laparoscopic incisional/ventral hernia repairs of varying sizes.  Mesh insertion through a 2 - 3cm skin incision at the center of the defect directly (inside of a plastic sleeve) or through 15mm port may be a viable alternative for larger defect requiring larger mesh size (> 30 cm). |
| --- | --- |
| Level 5 | Mesh-skin contact can impregnate the mesh with bacteria.  Largest size light weight mesh can be safely inserted through 10 – 12 mm port |

**Recommendations**

| Grade B | Large meshes should be tightly rolled up for safe and effective insertion. |
| --- | --- |
| Grade C | In very large meshes (35x30) a 15 mm port may be used.  Mesh-skin contact should be avoided. |

**References**

1. [Theodoropoulou K](http://www.ncbi.nlm.nih.gov/pubmed?term=Theodoropoulou%20K%5BAuthor%5D&cauthor=true&cauthor_uid=20529535), [Lethaby D](http://www.ncbi.nlm.nih.gov/pubmed?term=Lethaby%20D%5BAuthor%5D&cauthor=true&cauthor_uid=20529535), [Hill J](http://www.ncbi.nlm.nih.gov/pubmed?term=Hill%20J%5BAuthor%5D&cauthor=true&cauthor_uid=20529535), [Gupta S](http://www.ncbi.nlm.nih.gov/pubmed?term=Gupta%20S%5BAuthor%5D&cauthor=true&cauthor_uid=20529535), [Bradpiece H](http://www.ncbi.nlm.nih.gov/pubmed?term=Bradpiece%20H%5BAuthor%5D&cauthor=true&cauthor_uid=20529535) (2010) Laparoscopic hernia repair: a two-port technique.[JSLS](http://www.ncbi.nlm.nih.gov/pubmed?term=20529535) 14:103-5**(level 3b)**
2. [Hussain A](http://www.ncbi.nlm.nih.gov/pubmed?term=Hussain%20A%5BAuthor%5D&cauthor=true&cauthor_uid=18435900), [Mahmood H](http://www.ncbi.nlm.nih.gov/pubmed?term=Mahmood%20H%5BAuthor%5D&cauthor=true&cauthor_uid=18435900), [Shuaib S](http://www.ncbi.nlm.nih.gov/pubmed?term=Shuaib%20S%5BAuthor%5D&cauthor=true&cauthor_uid=18435900), [El-Hasani S](http://www.ncbi.nlm.nih.gov/pubmed?term=El-Hasani%20S%5BAuthor%5D&cauthor=true&cauthor_uid=18435900) (2008) Prevention of trocar site incisional hernia following laparoscopic ventral hernia repair.[JSLS](http://www.ncbi.nlm.nih.gov/pubmed?term=18435900) 12: 206-9 **(level 3b)**
3. [Perry KA](http://www.ncbi.nlm.nih.gov/pubmed?term=Perry%20KA%5BAuthor%5D&cauthor=true&cauthor_uid=18274823), [Millikan KW](http://www.ncbi.nlm.nih.gov/pubmed?term=Millikan%20KW%5BAuthor%5D&cauthor=true&cauthor_uid=18274823), [Huang WW](http://www.ncbi.nlm.nih.gov/pubmed?term=Huang%20WW%5BAuthor%5D&cauthor=true&cauthor_uid=18274823), [Myers JA](http://www.ncbi.nlm.nih.gov/pubmed?term=Myers%20JA%5BAuthor%5D&cauthor=true&cauthor_uid=18274823) (2008) A novel approach to extraction of incarcerated omentum and mesh insertion in laparoscopic ventral hernia repair. [Surg Endosc](http://www.ncbi.nlm.nih.gov/pubmed?term=18274823) 22: 798-801.**(level 5)**
4. [Perrone JM](http://www.ncbi.nlm.nih.gov/pubmed?term=%22Perrone%20JM%22%5BAuthor%5D), [Soper NJ](http://www.ncbi.nlm.nih.gov/pubmed?term=%22Soper%20NJ%22%5BAuthor%5D), [Eagon JC](http://www.ncbi.nlm.nih.gov/pubmed?term=%22Eagon%20JC%22%5BAuthor%5D), [Klingensmith ME](http://www.ncbi.nlm.nih.gov/pubmed?term=%22Klingensmith%20ME%22%5BAuthor%5D), [Aft RL](http://www.ncbi.nlm.nih.gov/pubmed?term=%22Aft%20RL%22%5BAuthor%5D), [Frisella MM](http://www.ncbi.nlm.nih.gov/pubmed?term=%22Frisella%20MM%22%5BAuthor%5D), [Brunt LM](http://www.ncbi.nlm.nih.gov/pubmed?term=%22Brunt%20LM%22%5BAuthor%5D) (2005) Perioperative outcomes and complications of laparoscopic ventral hernia repair.[Surgery](http://www.ncbi.nlm.nih.gov/pubmed?term=perrone%202005%20incisional%20hernia) 138: 708-15.**(level 3b)**
5. [Nimeri AA](http://www.ncbi.nlm.nih.gov/pubmed?term=%22Nimeri%20AA%22%5BAuthor%5D), [Brunt LM](http://www.ncbi.nlm.nih.gov/pubmed?term=%22Brunt%20LM%22%5BAuthor%5D) (2006)Laparoscopic ventral hernia repair: 5-mm port technique  and alternative mesh insertion method. [J Am Coll Surg](http://www.ncbi.nlm.nih.gov/pubmed/16571446)202: 708-10.**(level 4)**
6. [Agarwal BB](http://www.ncbi.nlm.nih.gov/pubmed?term=Agarwal%20BB%5BAuthor%5D&cauthor=true&cauthor_uid=18813981), [Agarwal S](http://www.ncbi.nlm.nih.gov/pubmed?term=Agarwal%20S%5BAuthor%5D&cauthor=true&cauthor_uid=18813981), [Mahajan KC](http://www.ncbi.nlm.nih.gov/pubmed?term=Mahajan%20KC%5BAuthor%5D&cauthor=true&cauthor_uid=18813981) (2009) Laparoscopic ventral hernia repair: innovative anatomical closure, mesh insertion without 10-mm transmyofascial port, and atraumatic mesh fixation: a preliminary experience of a new technique.[Surg Endosc](http://www.ncbi.nlm.nih.gov/pubmed?term=18813981) 23: 900-5.**(level 5)**
7. [Carlson MA](http://www.ncbi.nlm.nih.gov/pubmed?term=Carlson%20MA%5BAuthor%5D&cauthor=true&cauthor_uid=17318691), [Petersen A](http://www.ncbi.nlm.nih.gov/pubmed?term=Petersen%20A%5BAuthor%5D&cauthor=true&cauthor_uid=17318691) (2007) Technique for the insertion of large mesh during minimally invasive incisional herniorrhaphy.[Surg Endosc](http://www.ncbi.nlm.nih.gov/pubmed?term=%2017318691) 21:1243-4.**(level 5)**
8. Miller KS, Carey SD, Rodriguez FJ, Smoot RT.(2003) Complications and their management. In Laparoscopic Hernia Surgery. Edt. K. LeBlanc. Arnold London, pp 161-169.**(level 4)**
9. [Liberman MA](http://www.ncbi.nlm.nih.gov/pubmed?term=Liberman%20MA%5BAuthor%5D&cauthor=true&cauthor_uid=11800347), [Rosenthal RJ](http://www.ncbi.nlm.nih.gov/pubmed?term=Rosenthal%20RJ%5BAuthor%5D&cauthor=true&cauthor_uid=11800347), [Phillips EH](http://www.ncbi.nlm.nih.gov/pubmed?term=Phillips%20EH%5BAuthor%5D&cauthor=true&cauthor_uid=11800347) (2002) Laparoscopic ventral and incisional hernia repair: a simplified method of mesh placement.[J Am Coll Surg](http://www.ncbi.nlm.nih.gov/pubmed?term=11800347) 194: 93-5.**(level 5)**
10. [WalterCJ](http://www.ncbi.nlm.nih.gov/pubmed?term=%22Walter%20CJ%22%5BAuthor%5D), [Beral DL](http://www.ncbi.nlm.nih.gov/pubmed?term=%22Beral%20DL%22%5BAuthor%5D), [Drew P](http://www.ncbi.nlm.nih.gov/pubmed?term=%22Drew%20P%22%5BAuthor%5D). Optimum mesh and port sizes for laparoscopic incisional hernia repair. [J Laparoendosc Adv Surg Tech A.](http://www.ncbi.nlm.nih.gov/pubmed/17362181) 2007 Feb;17(1):58-63.**(level 5)**

**Section 5: Complications**

**Management of bowel injury during laparoscopic ventral incisional hernia**

**repair**

Timoney, Michael; Rim, Sean; Ferzli, George

*Search Terms: “Laparoscopic ventral hernia repair” AND “enterotomy” AND “mesh”*

A systematic search of the literature was performed in January 2012 using Medline, PubMed, Cochrane library and reference lists.

27 articles were found and analyzed, 9 were added. 12 articles were used for this review.

**Key questions:**

**-What are the incidences of bowel injury and what are the safest techniques to avoid them?**

**-Safest management in case of bowel injury and are alternatives?**

**Statements:**

| **Level 1** | The enterotomy rate for laparoscopic ventral hernia is 1.78%. The mortality rate for these patients is 2.8%.  In most cases (92%) the small bowel is injured.  Most frequent causes are rough adhesiolysis and the use of energy sources near adherent bowel. |
| --- | --- |
| **Level 4** | A bowel injury may occur during LVHR, particularly in cases where extensive adhesiolysis is performed by surgeons with less experience.    The extent of the bowel injury and contamination may dictate the type of repair.  Bowel injury does not mandate conversion to open repair.  LVHR can be delayed in patients who may have increased risk factors for developing a mesh infection.  Bowel injury does not preclude immediate LVHR. |

**Recommendations:**

| **Grade C** | Adhesiolysis should be done close to the abdominal wall and not to the bowel.  Sharp dissection techniques should be preferred and the use of energy sources must be avoided near bowel.  Conversion to laparotomy may be performed, especially if the surgeon is not proficient at laparoscopic bowel repair techniques.  A primary open repair may be performed in the setting of gross spillage. An open prosthetic repair may be undertaken if conditions remain sterile.  Small laparotomy away from the hernia defect may be used to repair a recognized enterotomy followed by continuation of LVHR.  In the event of a bowel injury repaired laparoscopically, LVHR may be performed with a delay of 3 to 7 days in which the patient is observed with administration of parenteral antibiotics and develops no signs and symptoms of infection.  LVHR may be completed in the setting of recognized bowel injury if repaired immediately with minimal spillage. This requires an advanced ability to laparoscopically repair bowel. |
| --- | --- |

**Introduction:**

The first laparoscopic repair of a ventral incisional hernia (LVHR) was described by LeBlanc in 1993. Approximately 90,000 ventral incisional hernia repairs are performed in the United States each year. Today, the LVHR continues to gain increasing popularity versus open repair [1]. Recurrence rates have been found to be similar between LVHR and open repairs. Complications of the laparoscopic technique tend to be fewer but may be more serious, mainly due to higher rates of enterotomies [2-3].

**Avoiding bowel injury during LVHR:**

The management of bowel injury during LVHR remains a vexing and controversial problem. A recent literature search by LeBlanc demonstrated an enterotomy rate of 1.78% for LVHR. The overall mortality rate for these patients was 2.8%. In the subset of patients where the injury was occult and recognition was delayed until after surgery (18%), the mortality rate was as high as 7.7%. Predictably, small bowel was injured 92% of the time [4]. A recent Cochrane review revealed an enterotomy rate of 1.55% with LVHR versus 0.63% with the open approach [2-6].

Bowel injury can be classified in one of three categories. Immediately recognized injuries tend to result from initial port entry or from bowel manipulation and adhesiolysis. Missed injuries can also occur during adhesiolysis. These are usually recognized as a septic response in the first 24 hours post-operatively. Delayed injuries are suspected to occur as a result of progression of a serosal injury from an energy source such as electrocautery or ultrasonic dissection. These present within the first 5 days postoperatively [7-9].

Avoiding bowel injury is of utmost importance when performing a LVHR. It is advisable to gain access to the abdominal cavity via an open technique, far from the hernia or scar. Sharp dissection should always be utilized in areas of dense adhesions, particularly when the presence of bowel is suspected. Again, the use of energy sources near bowel may be a source of delayed injuries with significantly increased morbidity and mortality [7].

**Conversion to laparotomy for managing bowel injury during LVHR:**

If a bowel injury does occur during a LVHR, management may be best dictated by the extent of injury and contamination as well as the level of the surgeon’s skills and comfort with laparoscopic procedures. Options include immediate conversion to open bowel repair and hernia repair with or without mesh. If the surgeon is adept at laparoscopic bowel repair and contamination is limited, the injury may be repaired and the LVHR may be performed immediately. An alternative is to repair the bowel and delay the hernia repair after a period of inpatient observation and administration of parenteral antibiotics [7,10].

If the surgeon is inexperienced or is uncomfortable with laparoscopic bowel repair, an immediate conversion to a laparotomy is advisable. The bowel injury should then be repaired and the hernia defect treated according to the amount of contamination. With gross spillage, the hernia should be repaired primarily without the use of mesh [6,11]. In a 2010 study by Itani, he describes 73 patients who underwent conversion to an open technique for bowel injury with minimal contamination during LVHR. In three patients, the enterotomy was repaired and the herniorrhaphy was performed with polypropylene mesh. None of the patients who underwent conversion to laparotomy, including those in whom a mesh was placed, developed a surgical site infection [3].

**Alternative methods for dealing with bowel injury during LVHR:**

There are several reasonable alternatives to conversion to laparotomy in the event of a bowel injury. Both Carbajo and Heniford have described a case in which a “mini-laparotomy” was made in order to repair the bowel injury. The incision was made away from the hernia and under direct visualization with the laparoscope, the injured bowel was brought through the incision and repaired extracorporeally. The incision was then closed and the LVHR was resumed [6,11].

If there is gross contamination, another viable option may be to repair the injury laparoscopically and to defer the herniorrhaphy. Lederman and Ramshaw reported a series of 9 patients who had an enterotomy during LVHR. After repair of the injury, the patients were admitted and observed for an average of three days on IV antibiotics. 7 of 9 patients then returned to the operating room for successful completion of their LVHR [7]. In 2005, Lederman identified several factors which put the patient at higher risk of enterotomy. These include adhesiolysis longer than three hours, chronic obstruction, inflamed bowel, and prior mesh incorporated into bowel [7]. The presence of these factors, or the recognition of a visceral injury, should prompt the surgeon to consider delaying the repair of the hernia until the patient shows no signs of intraabdominal infection.

Some authors advocate immediate repair of bowel injuries and completing the LVHR in the same setting. Carbajo reported 8 patients who underwent laparoscopic repair of enterotomies followed by immediate LVHR [11]. Similarly, Heniford noted 5 patients with hollow organ injuries that were repaired and the herniorrhaphy was completed laparoscopically [6]. The overriding principles here are that there must be minimal to no obvious gross contamination and the surgeon should be skilled at laparoscopic repair of bowel.

Finally, the use of biologic mesh has also been described as a safe method of completing a LVHR in the presence of contamination. Although synthetic mesh is generally preferred over biologic mesh in terms of recurrence prevention, biologic mesh has been successfully used in contaminated and infected fields. In 2004, Franklin described his experience with the use of porcine derived prosthetic mesh in 43 patients who underwent successful LVHR in a contaminated field. Details of the contamination are vague but included bowel resection, strangulation, and prior mesh infection. One patient developed a wound infection and a fistula. He described no recurrences [12].

**References**

1. LeBlanc KA, Booth WV. Laparoscopic repair of incisional abdominal hernias using expanded polytetrafluoroethylene: preliminary findings. Surg Laparosc Endosc. 1993;3:39-41. **(level 4)**

2. Sauerland S, Walgenbach M, Habermalz B, Seiler CM, Miserez M. Laparoscopic versus open surgical techniques for ventral or incisional hernia repair. Cochrane Database Syst Rev. 2011 Mar 16;(3). **(level 1A)**

3. Itani KM, Kim, LT, Anthony T, Berger DH, Reda D, Neumayer L; Veterans Affairs Ventral Incisional Hernia Investigators. Comparison of laparoscopic and open repair with mesh for the treatment of ventral incisional hernia: a randomized trial. Arch Surg. 2010 Apr;145(4):322-8. **(level 1B)**

4. LeBlanc KA, Elieson, MJ, Corder JM. Enterotomy and mortality rates of laparoscopic incisional and ventral hernia repair: A review of the literature. JSLS (2007)11:408–414. **(level 1A)**

5. Ramshaw BJ, Esartia P, Schwab Jet al: Comparison of laparoscopic and open ventral herniorrhaphy. Am Surg 65(9):827-831, 1999; discussion 831-2. **(level 2A)**

6. Heniford BT, Park A, Ramshaw BJ, Voeller G: Laparoscopic repair of ventral hernias: nine years' experience with 850 consecutive hernias. Ann Surg 238(3):391-399, 2003; discussion 399-400. **(level 4)**

7. Lederman AB, Ramshaw BJ. A Short-Term Delayed Approach to Laparoscopic Ventral Hernia When Injury Is Suspected. Surgical Innovation, Vol 12, No 1 (March), 2005: pp 31-35. **(level 4)**

8. Salameh JR, Sweeney JF, Graviss EA, et al: Laparoscopic ventral hernia repair during the learning curve. Hernia 6(4):182-187, 2002. **(level 4)**

9. Burger JWA, Luijendijk RW, Hop WCJ, Halm JA, Verdaasdonk EGG, Jeekel J. Long-term follow-up of a randomized controlled trial of suture versus mesh repair of incisional hernia. Annals of Surgery 2004;240(4):578–85. **(level 1B)**

10. LeBlanc KA. Laparoscopic incisional and ventral hernia repair: Complications—how to avoid and handle. Hernia (2004) 8: 323–331. **(level 5)**

11. Carbajo MA, Martın del Olmo JC, Blanco JI, Toledano M, de la Cuesta C, Ferreras C, Vaquero C. Laparoscopic approach to incisional hernia-lessons learned from 270 patients over 8 years. Surg Endosc (2003) 17: 118–122. **(level 4)**

12. Franklin ME, Gonzalez JJ, Glass JL. Use of porcine small intestinal submucosa as a prosthetic device for laparoscopic repair of hernias in contaminated fields: 2-year follow-up. Hernia. 2004 Aug;8(3):186-9. **(level 4)**

**Unrecognized Enterotomy**

Karl A. LeBlanc, MD, MBA, FACS

Matthias Rohr, MD

**Search Terms**

- Open abdomen AND enterotomy
- Damage control laparotomy AND enterotomy
- Laparoscopy AND enterotomy
- Enterotomy AND avoidance
- Inadvertent enterotomy AND hernia repair
- Enterotomy AND hernia repair
- Enterotomy AND hernia repair AND peritoneal contamination
- Required in English literature or English abstract

We queried the Pub Med and Embase databases as well as the Cochrane register using the search terms noted above from the time frame of 1960-2011.There were a total of 174 articles that met the search criteria but only 78 of these adequately dealt with the subject matter. Of these 32 qualified for the research by the evidence based medicine approach.

**Statements**

| Level 2A | Reoperation will be necessary  The recommendation of the method of repair or resection of the intestinal injury cannot be supported  Mesh explantation is recommended with primary repair of the hernia. |
| --- | --- |
| Level 4 | Evidence supports a laparotomy but not specific treatment of the intestinal injury.   - - Repair or resection are both appropriate   - Mesh explantation will be necessary   - Primary repair of the hernia is recommended |
| Level 5 | When this is suspected, repeat laparoscopy or laparotomy will be necessary   - - Repair or resection are both appropriate   - Mesh explantation will be necessary   - Primary hernia repair is recommended |

**Recommendations**

| Grade B | Surgeons should re-explore the patient, either open or laparoscopically, if there is a suspicion of a missed enterotomy or to treat the enterotomy with repair, resection and/or stomal creation based upon the injured organ and the clinical situation.  This is an upgraded recommendation but this condition is so critical that should be given this recommendation. |
| --- | --- |
| Grade C | Mesh explantation should be done  Primary repair of the hernia, if feasible, is deemed best at this time |

**Introduction**

The first report of the repair of incisional and ventral hernias by the laparoscopic method did not usher in a rapid adoption that the laparoscopic cholecystectomy did just a few years before(1). There have been many subsequent studies and publications that have supported the success of the technique. Despite its success, one of the most feared complications is that of an unrecognized enterotomy. The risk of this problem existed with the open procedure but its recognition postoperatively is more difficult due to the difference in the postoperative course of the patient and the fact that the laparoscopic procedure frequently results in an earlier discharge of the patient. The overall incidence of enterotomy ranges from1.78-6% (2,3,4). The reported rate of unrecognized enterotomy ranges from 0.68-25% (2,3,5). There is no statistically significant difference in the rate of an unrecognized enterotomy between the open or laparoscopic repair(2,6,7,8). The mortality of these events within any series ranges from 0.05-3.4% (2,3,4,5).However, in the patients that an unrecognized enterotomy occurred, the mortality within that group of patients ranges from 7.7-66%(2,3,4,5)Therefore, although enterotomy is not unavoidable in either the open or laparoscopic methods of these repairs, the consequences of this complication are significant.

**Discussion**

The findings of most of these reviews mentioned above were that there was very little specific discussion of unrecognized enterotomy, management of the enterotomy at reoperation or management of the hernia itself. Those reported herein dealt with these issues in some fashion.

Two Level 1A publications evaluated the laparoscopic repair of incisional and ventral hernias but did not specifically evaluate the subject of missed enterotomy but both concluded that there appeared to be a higher rate of injury to an intra-abdominal organ with the laparoscopic approach but not at a statistically significant higher level(6,7).

There were only two papers that discussed the method of repair of the intestinal injury and only one discussed the management of the hernia defect at Level 2A. One concluded that for unrecognized enterotomy “reoperation with closure/resection of the injury in conjunction with mesh explantation typically is necessary” (9). The other found that one method of repair was not superior to another. The conclusion was that primary repair of the injury by either suture or stapled closure was equally successful (2). It should be noted, however, that should the clinical condition of the patient and/or the reoperative findings require, an ostomy should be created. This was further corroborated by the single Level 2B study that created a stoma and left the hernia wound open in such an instance. The stoma was closed and the hernia was repaired primarily three months later(10).

**Table 1: Level 4 evidence studies.**

| Series | Incidence (%) | Laparoscopy/  Laparotomy | Primary Repair of Intestine/Resection | Mesh explant and Primary Hernia Repair |
| --- | --- | --- | --- | --- |
| Baccari(11) | 1 | Yes/Yes | Resection | Explant/Primary Repair |
| Ben-Haim(12) | 2 | No/Yes | Primary Repair | Explant/Hernia not repaired |
| Berger(13) | 1.3 | No/Yes | Repair (1), Resection(1) | Explant/Primary Repair |
| Binenbaum(14) | 0.3 | No/Yes | Resection | Not described |
| Heniford(15) | 1.7 | No/Yes | Resection | Primary repair |
| Koehler(4) | 6 | No/Yes | Resection | Explant/Primary repair |
| Moreno-Egea(3) | 1.1 | Not mentioned | Not discussed | Not discussed |
| Perrone(16) | 1.6 | No/Yes | Resection | Not mentioned/Primary hernia repair |
| Wara(17) | 1.4 | Not mentioned | Not discussed | Not discussed |
| Wright(5) | 0.68 | No/Yes | Not discussed | Explant/Not discussed |

Baccari (11) was the only author that attempted laparoscopic evaluation of the abdomen to ascertain the presence of an enterotomy. Once this was discovered, a formal laparotomy was undertaken. In the remaining papers, it is apparent that in the majority of instances in which this was discussed, mesh explantation, intestinal resection and primary repair of the hernia were the preferred management of the unrecognized enterotomy and the hernia. Regardless of the care that one undertakes it is clear that this occurrence not avoidable in all cases.

While there has been numerous published papers describing and reporting the repair in incisional hernias with the laparoscopic method, very few, even level 5 articles addressed this problem. Two have been identified. LeBlanc stated, “a laparotomy will generally be required with bowel resection and explant of the mesh”(18). Sarela recommended that “if there is a high index of suspicion for a missed enterotomy; a planned re-laparoscopy after 24-48 hours…”[should be done] but they did not provide any specific recommendations as to the management of either the intestinal injury or the hernia(19).

**Conclusion**

Based upon the relative paucity of high-level data as to the management of this serious problem, it seems that the safest approach is repair, resection of the injury, mesh explantation and primary repair of the fascial defect if it can be closed. If that is not possible or if the clinical condition warrants, treatment with an open abdomen is appropriate.

**References**

LeBlanc K, Booth WV. Laparoscopic repair of incisional abdominal hernias using expanded polytetrafluoroethylene: preliminary findings. SurgLaparoscEndosc. 1993;3:39-41.

LeBlanc KA,Elieson MJ, Corder JM. Enterotomy and Mortality Rates of Laparoscopic Incisional and Ventral Hernia Repair: A Review of the Literature. JSLS 2007;11:408–414**.(Level 2A)**

Moreno Egea DA, Torralba Martinez JA, Morales Cuenca G, De Miquel, Martín Lorenzo JG, AguayoAlbasini JL, CanterasJordana M. Mortality following laparoscopic ventral hernia repair: lessons from 90 consecutive cases and bibliographical analysis. Hernia (2004) 8: 208–212. **(level 3)**

Koehler RK, Voeller G. Recurrences in Laparoscopic Incisional Hernia Repairs: A Personal Series and Review of the Literature. JSLS (1999)3:293-304. **(level4)**

Wright BE, Niskanen BD, Peterson DJ, Ney AL, Odland MD, VanCamp J, Zera RT, Rodriquez JL. Laparoscopic ventral hernia repair: are there comparative advantages over traditional methods of repair? Am Surg 2002;68(3):291-296**.(level 3)**

Forbes SS, Eskicioglu C, McLeod RS, Okrainec A. Meta-analysis of randomized controlled trials comparing open and laparoscopic ventral and incisional hernia repair with mesh. British Journal of Surgery 2009; 96: 851–858 **(level 1A)**

Sauerland S, Walgenbach M, Habermalz B, Seiler CM, Miserez M. Laparoscopic versus open surgical techniques for ventral or incisional hernia repair (Review). <http://www.thecochranelibrary.com>, The Cochrane Database Syst Rev, 2011. 16(3): p. CD007781**(level 1A)**

Müller-Riemenschneider F, Roll S, Fredrich M, Zieren J, Reinhold T, von der Schulenburg JMG, Greiner W, Willich SN. Medical effectiveness and safety of conventional compared to laparoscopic incisional hernia repair: A systematic review. SurgEndosc (2007) 21:2127–2136. **(level 2A)**

Carlson MA, Frantzides CT, Shostrom VK, Laguna LE. Minimally invasive ventral herniorrhaphy: an analysis of 6,266 published cases. Hernia 2008;12:9-22**.(Level 2A)**

Barbaros U, Asoglu O, Seven R, Erbil Y, Dinccag A, Deveci U, Ozarmagan S, Mercan S. The comparison of laparoscopic and open ventral hernia repairs: a prospective randomized study. Hernia (2007) 11:51–56. **(level 1B)**

Baccari P, Nifosi J, Ghirardelli L, Staudacher C. Laparoscopic Incisional and Ventral Hernia Repair Without Sutures: A Single-Center Experience with 200 Cases. J LaparoendoAdvSurg Tech 2009;19(2):175-179**.(Level 4)**

Ben-Haim M, Kuriansky J, Tal R, Zmora O, Mintz Y, Rosin D, Ayalon A, Shabtai M. Pitfalls and complications with laparoscopic intraperitoneal expanded polytetrafluoroethylene patch repair of postoperative ventral hernia.Lessons from the first 100 consecutive cases.SurgEndosc (2002) 16:785-788. **(Level 4)**

Berger D, Bientzle M, Müller A. Postoperative complications after laparoscopic incisional hernia repair. Incidence and treatment.SurgEndosc (2002) 16: 1720–1723**.(Level 4)**

Binenbaum SJ, Goldfarb MA. Inadvertent Enterotomy in Minimally Invasive Abdominal Surgery. JSLS 2006;10(3):336-340**.(Level 4)**

Heniford BT, Park A, Ramshaw,BJ, Voeller G. Laparoscopic Repair of Ventral Hernias.Nine Years’ Experience With 850 Consecutive Hernias. Ann Surg 2003;238: 391–400**.(Level 4)**

Perrone JM, Soper NJ, Eagon C, Klingensmith ME, Aft RL, Frisella MM, Brunt M. Perioperative outcomes and complications of laparoscopic ventral hernia repair. Surgery 2005;138:708-16**.(Level 4)**

Wara P, Anderson LM.Long-term follow-up of laparoscopic repair of parastomal hernia using a bilayer mesh with a slit.SurgEndosc (2011) 25:526–530**.(Level 4)**

LeBlanc KA. Laparoscopic incisional and ventral hernia repair: Complications-how to avoid and handle. Hernia 2004;8:323-331. **(Level 5)**

Serala AI. Controversies in laparoscopic repair of incisional hernia. J Minim Access Surg 2006;2(1):7-11**.(Level 5)**

**Risk factors for infection in laparoscopic incisional / ventral hernia repair**

P. Chowbey

**Method-** The conclusion and recommendation for the risk factors for infection in laparoscopic ventral/incisional hernia are based on a systematic search and review of literature performed in Pubmed, Medline, Cochrane library, EMBASE, British journal of Surgery database, UK Pubmed Central, Google, Google scholar, Scirus, Ovid and Directory of Open Journal Access (DOAJ).

Thirty -eight publications were found which covered the topic out of which fifteen statements were found useful for this research. In addition it is based on a consensus conference on guidelines for laparoscopic treatment of ventral and incisional hernia held on 13th October 2011 in Suzhou, China.

**Search terms:** risk factors for SSI, risk factors for infection, causes of mesh infection, laparoscopic ventral/incisional hernia repair, perioperative risk factor for infection.

**Statements**

| Level 1 | Level Preoperative transfusion may also increase risk of SSI  Level 1 Laparoscopic operations lead to lower incidence of SSI than open operations as the total length of incisions is shorter which makes bacteria less likely to enter the subcutaneous space |
| --- | --- |
| Level 2 | Level 2 In elderly patients, COPD and low preoperative serum albumin were independent predictors of wound infections and CAD, COPD, low preoperative serum albumin, and steroid use were independent predictors of increased hospital length of stay.  Level 2 In patients who undergo ventral hernia repair with a simultaneous bowel resection, there is a higher incidence of infectious and noninfectious complications with mesh use  Level 2 Wound infection is lower in laparoscopic hernia repair compared to open, as there is decreased extent of tissue dissection in the former  Level 2 Mesh, wherever possible, should not be brought in contact with skin to avoid contamination by skin flora. Polyester meshes were found to have the highest incidence of infection, fistulisation and recurrence  Level 2 Patients given a prophylactic antibiotic have a lower incidence of SSI |
| Level 3 | Patien Operation time is the only significant risk factor associated with mesh graft infection following incisional hernia repair  Level 3 Patient age, ASA score, smoking, and the duration and emergency setting of the operation are found to be associated with the development of synthetic mesh infection  Level 3 There are significant associations between complications and larger hernias, previous herniorrhaphy, longer operating times, and longer hospital stays |
| Level 4 | Source o The patient characteristics that possibly increase the risk of SSI (surgical site infection) include administration of steroids, smoking, old age and underlying disorders like obesity, diabetes, malnutrition and remote site infection  Source of SSI is skin flora or bacteria contamination from a viscus  Level 4 The usage of the mesh does not increase the incidence of SSI, although the consequences of the mesh infection may be severe  Level 4 In regard to the position of the mesh, SSI is more common if the mesh is placed subcutaneously than in the case of sub-aponeurotic premuscular, pre-aponeurotic retromuscular or pre-peritoneal mesh placement. If the infection is present then tension free techniques using non-resorptive prosthetic implants are not recommended  Prolonged preoperative hospital stay, preoperative nares colonization with staphylococcus aureus  Level 4 The presence of drainage and its duration increases the incidence of SSI. If there is an indication for drainage it should be as short as possible |

# Recommendations

| Grade A | Regarding the risk of postoperative SSI laparoscopic procedure must be preferred  Before operation, known risk factors for SSI must be treated if possible.  Operation time and hospital stay must be as short as possible. |
| --- | --- |
| Grade B | Smoking cessation, glycemic control and treating remote infections before the surgery should be done before operation.  Prosthetic mesh insertion with simultaneous bowel resection should be avoided |
| Grade C | Grade C Preoperative clipping of hair is recommended  Weight loss befor operation may be considered |

# Discussion

After laparoscopic ventral and incisional hernia repair patients may develop surgical site infection (SSI). SSI significantly increases morbidity and mortality.[1] Reported incidence of infection in Open procedures is 10% and in laparoscopic procedures is 1.1 %.[ 2] Laparoscopic procedures lower the risk of infection by reducing wound size, hospital stay, operative time and probability of bacteria entering the subcutaneous space. [3, 4, 5, 6]

Pathogens that frequently cause SSI are Staphylococcus aureus, Enterococcus species and Escherichia coli which are usually sourced from patient’s skin, mucous membranes or bowel and rarely from another infected site in the body. [6, 7] The risk factors for infection can be divided into patient related risk factors and surgery related risk factors.

**Patient related risk factors** Gender and SSI cannot be correlated but the rate of wound infection for 15 to 24 year old patients is 10% and significantly increases for patients over 65 years of age.[8] Old age with greater likelihood of co morbid conditions weakens the immune system and increases risk for infection. Dunne et al reported CAD, COPD and low preoperative serum albumin as independent predictors for infection in elderly patients. [9] Patients on immunosuppressants, steroids and smokers also have greater chance of contracting infection. Risk of infection increases five fold for smokers and by nine percent in patients on steroids [8] A prospective study on 5031 patients conducted by Malone et al confirmed diabetes and malnutrition (defined as significant weight loss 6 months prior to surgery) as significant predictors for infection. [10] Obesity decreases the blood circulation in fat tissue and increases the risk of infection. [11] Other factors like history of infection, high ASA grades, hypoxia, hypothermia, radiation and peripheral vascular disease also contribute to SSI. [12, 13, 14, 15,]

**Surgery related risk factors** Preoperative factors increasing the risk of infection are shaving of the surgical site, duration of scrubbing, antiseptic use and blood transfusion. SSI rates were 5.6% in patients who had hair removed by razor compared with 0.6% in patients who had either their hair removed by depilatory agents or no hair removal. [16] Blood transfusion increases the risk two fold. [17]

Length of the operating time also predisposes to risk of infection. Procedures longer than 3–4 hours increase the risk [12]. In addition, mesh infection is a major contributing factor for infection. The reported incidence after laparoscopic repairs is 0–3.6% [18]. A mesh infection rate as low as 0.78% after laparoscopic repair was published in a systematic review by Carlson et al. [19] Polyester meshes and meshes positioned subcutaneously were found to have high incidence of infection.[18,20] The use of prosthetic mesh with bowel resection or injury increases the risk of infection many fold. [21] Also blood loss during the surgery is a significant predictor for infection. Post surgery complications like seroma, thromboembolism, pulmonary embolism, post procedure pneumonia and anemia makes the patient more susceptible to infection. [22]

To prevent infection, management of these risk factors is important. Some risk factors like old age, co-morbidities and immunosuppression cannot be modified. The ones that can be modified should be addressed and taken care of by adhering to established guidelines and protocols. [23] Minimizing smoking before the surgery improves postoperative SSI outcomes. Reports have not established how preoperative parenteral or enteral nutrition influences SSI outcome. [24] Strict preoperative glycemic control with maintenance of intraoperative normothermia is necessary. [25] Remote infection especially when mesh is being implanted should be treated and resolved completely before the surgery.

Preoperative hair removal should be avoided and clipping should be performed where possible. [16] Proper sterilization is of utmost importance. Antiseptic showering, use of antibiotics preoperatively and surgical hand hygiene significantly reduces chances of infection. The administration of antibiotics half an hour before surgery produces best results. [26] Intraoperatively, careful attention to proper surgical technique and timely completion of the operation also reduces the risk of SSI.

Knowledge of risk factors causing infection is very important as they help in identifying patients at risk of infection and initiating a strategy to minimize them.

**References** (in parentheses graduation of evidence)

1. National Nosocomial Infections Surveillance (NNIS) report, data summary from October 1986-April 1996, issued May 1996. A report from the National Nosocomial Infections Surveillance (NNIS) System. Am J Infect Control 1998;24(5):380–8**.(level 4)**
2. Franklin ME, Dorman JP, Glass JL, Balli JE, Gonzalez JJ. Laparoscopic Ventral and Incisional Hernia Repair. Surgical Laparoscopy Endoscopy & Percutaneous Techniques [Internet]. 1998;8(4). **(level 3)**
3. DenHartog D, Dur AHM, Tuinebreijer WE, Kreis RW. Open surgical procedures for incisional hernias. Cochrane Database of Systematic Reviews 2008, Issue 3. Art. No.: CD006438. **(level 1A)**
4. Zuvela M, Milićević M, Galun D, Lekić N, Basarić D, Tomić D, Petrović M, Palibrk I. Infection in hernia surgery. Acta Chir Iugosl. 2005;52(1):9-26.**(level 2)**
5. Kensarah AM. A Long-term Follow-up: Suture versus Mesh Repair for Adult Umbilical Hernia in Saudi Patients. A Single Center Prospective Study. Surgical Science. 2011;2(03):155–8**. (level 2)**
6. Chowbey PK, Sharma A, Mehrotra M, Khullar R, Soni V, Baijal M. Laparoscopic repair of ventral / incisional hernias. J Min Access Surg 2006;2:192-8 **(level 3)**
7. Perl TM., Golub JE. New approaches to reduce Staphylococcus aureus nosocomial infection rates: Treating S. aureus nasal carriage. Annals of Pharmacotherapy 1998;32(1): S7-16**.(level 4)**
8. Razavi S, Ibrahimpoor M, Sabouri Kashani A, Jafarian A. Abdominal surgical site infections: incidence and risk factors at an Iranian teaching hospital. BMC Surgery [Internet]. 2005;5(1):2**.(level 4)**
9. Dunne JR, Malone DL, Tracy JK, Napolitano LM. Abdominal wall hernias: risk factors for infection and resource utilization. Journal of Surgical Research. 2003 May 1;111(1):78–84. **(level 2)**
10. Malone DL, Genuit T, Tracy JK, Gannon C, Napolitano LM. Surgical site infections: reanalysis of risk factors. J Surg Res. 2002 Mar;103(1):89-95.**(level 3)**
11. Anaya DA, Dellinger EP. The Obese Surgical Patient. Surgical infections: 2006;7(5). 473-480 **(level 4)**
12. Kurz A, Sessler DI, Lenhardt R. Perioperative normathermia to reduce the incidence of surgical wound infection and shorten hospitalization. New England Journal of Medicine.1996; 334:1209–1215**.(level 1B)**
13. Cheadle WG. Risk factors for surgical site infection. Surg Infect (Larchmt).2006;7 Suppl 1:S7-11 **(level 4)**
14. Mangram AJ, Horan TC, Pearson ML, Silver LC, Jarvis WR; The Hospital Infection Control Practices Advisory Committee. Guideline for prevention of surgical site infection, 1999. Infect Control Hosp Epidemiol. 1999;20:247-278. **(level 4)**
15. Boni L, Benevento A, Rovera F, Dionigi G, Di Giuseppe M, Bertoglio C, Dionigi R. Infective complications in laparoscopic surgery. Surg Infect (Larchmt). 2006;7 Suppl 2:S109-11**.(level 2)**
16. Seropian R, Reynolds BM. Wound infection after preoperative depilation versus razor preparation. Am J Surg 1970 12: 251-254.**(level 4)**
17. Hill GE, Frawley WH, Griffith KE, et al. Allogeneic blood transfusion increases the risk of postoperative bacterial infection: a meta-analysis. J Trauma 2003;54(5):908–14.**(level 1A)**
18. Eriksen JR, Gogenur T, Rosenberg J. Choice of mesh for laparoscopic ventral hernia repair. Hernia 2007;11:481–492**.(level 3)**
19. Carlson MA, Frantzides CT, Shostrom VK, et al. Minimally invasive ventral herniorrhaphy: An analysis of 6,266 published cases. Hernia 2008;12:9–22**.(level 4)**
20. Franklin ME Jr, Gonzalez JJ Jr, Glass JL, Manjarrez A. Laparoscopic ventral and incisional hernia repair: an 11-year experience. Hernia. 2004 Feb;8(1):23-7.Epub 2003 Sep 20. **(level 4)**
21. Xourafas D, Lipsitz SR, Negro P, Ashley SW, Tavakkolizadeh A. Impact of mesh use on morbidity following ventral hernia repair with a simultaneous bowel resection. Archives of surgery.2010 Aug;145(8):739–44. Available from: [http://www.ncbi.nlm.nih.gov/pubmed/20713925**(level**](http://www.ncbi.nlm.nih.gov/pubmed/20713925(level) **3)**
22. Leber GE, Garb JL, Alexander AI, Reed WD. Long term complications associated with prosthetic repair of incisional hernias. *Arch Surg.* 1998;133:378–82 **(level 3)**
23. Kirby JP, Mazuski JE. Prevention of surgical site infection. The Surgical clinics of North America. 2009 Apr;89(2):365–89,9 **(level 5)**
24. Muller  JM, Brenner  U, Dienst  C , et al.  Preoperative parenteral feeding in patients with gastrointestinal carcinoma.  Lancet. 1982;1:68–71**.(level 3)**
25. Latham  R, Lancaster  AD, Covington  JF , et al.  The association of diabetes and glucose control with surgicalsite infections among cardiothoracic surgery patients.  Infection Control & Hospital Epidemiology. 2001;22:607–612.**(level 3)**
26. Yerdel  MA, Akin  EB, Dololan  S , et al.  Effect of singledose prophylactic ampicillin and sulbactam on wound infection after tension-free inguinal hernia repair with polypropylene mesh.  Ann Surg. 2001;233:26–33. **(level 1B)**

**Mesh Infection**

F. Köckerling, P. Chowbey, M. C. Misra

Search terms: "Incisional Hernia"; "Ventral Hernia"; "Laparoscopic Incisional Hernia Repair"; Laparoscopic Ventral Hernia Repair"; "Hernia Repair and Mesh Infection", "Mesh Infection"; "Hernia Repair and Wound Infection"; "Laparoscopic Ventral Hernia Repair and Mesh Infection"; Incisional Hernia Repair and Mesh Infection"

A systematic search of the available literature was performed in July 2012 using Medline, PubMed, Cochrane library and relevant journals and reference lists using the above listed search terms.

The first search detected 118 relevant articles. In a second - level search 4 articles were added. In Summery 15 articles and studies were used for this review.

**Key questions:**

**How to Deal with Mesh Infection? Removal of Mesh? Closure of the Hernia Defect? Biologic Mesh Implantation? When is it Safe to Place Synthetic Mesh Again? How Long Do We Wait for Reoperation? Vacuum-Assisted Therapy?**

**Statements**

| Level 1A  Level 3  Level 5 | The rate of mesh infections following laparoscopic ventral and incisional hernia repair is low at 1 %.  Not in all cases with wound infection following laparoscopic ventral and incisional hernia repair does the mesh need to be removed.  Infected ePTFE meshes need significantly more often removal in comparison to polypropylene based meshes.  There are case reports in the literature indicating that mesh salvage for infected meshes after laparoscopic ventral and incisional hernia repair is possible.  Conservative management of mesh infection following laparoscopic ventral and incisional hernia repair can be tried by percutaneous drainage, drain irrigation with gentamycin 80 mg in 20 ml saline 3 times a day and intravenous antibiotics.  If the conservative treatment of a mesh infection after laparoscopic ventral and incisional hernia repair has failed, all the same options as for mesh infection after open repair need to be considered depending on the individual findings of the patient.  The following options are reported for treatment of mesh infections following open repair: Mesh removal with primary skin closure – Repeat repair of the defect after 6-9 months.  Mesh removal - Component separation technique - Vacuum-assisted closure or open wound dressing.  Mesh removal - Repair with biological mesh - Vacuum-assisted closure or open wound dressing.  Mesh salvage - Vacuum-assisted closure or open wound dressing. |
| --- | --- |

**Recommendations**

| Grade B  Grade D | An infected ePTFE mesh after laparoscopic ventral and incisional hernia repair should be removed.  It can be attempted to preserve an infected composite mesh after laparoscopic ventral and incisional hernia by means of interventional and conservative treatment using percutaneous drainage, drain irrigation with gentamycin and intravenous antibiotics.  If the conservative treatment has failed or the finding does not justify conservative treatment, the options reported for treatment of mesh infections following open repair should be used.  As the options are only reported for cases, a decision must always be taken in accordance with the findings of the individual patient. |
| --- | --- |

An important advantage of laparoscopic IPOM technique compared with open repair of incisional and ventral hernias are the lower rates of wound and mesh infections. In a meta-analysis it has been demonstrated that after laparoscopic repair of incisional and ventral hernias significantly fewer wound infections or the need for mesh removal were noted (Level 1A) (Forbes et al. 2009). In a metaanalysis of Sauerland et al. (2011) the local infection rate in the laparoscopic group was 3.1 % versus 13,4 % in the open group (p < 0,00001). A local infection requiring mesh removal was found in 0,7 % in the laparoscopic group and in 3.5 % in the open group (P = 0,09). This trend is also seen for infections resulting in mesh removal. In that meta-analysis the rate of wound infections after laparoscopic repair was 2.23 %, whereby wound infections did not lead to mesh removal in 1.48 % of cases, but 0.74 % of wound infections did result in mesh removal (Forbes et al. 2009). In a pooled data analysis (Level 2A) by Pierce et al (2007) wound infections were found in 1.3 % of cases after laparoscopic repair and mesh infections in 0.9 %, whereas after open operation the wound infection rate was 10.9 % and mesh infections were 3.2 % (p<0.0001). In a large clinical case series and case analyses (Level 3), mesh infections were detected after laparoscopic IPOM in 0.78 % of patients,
n = 6, 206 (Carlson et al. 2008), in 0.90 %, with n = 4.582 (Pierce et al 2007) and in in 0.70 %, n = 850 patients (Heniford et al. 2009). In the literature there are case reports on treatment of mesh infections after laparoscopic repair of incisional and ventral hernias, whereby both mesh removal (Fortelny et al. 2010, Perrone et al. 2005) and mesh salvage are discussed (Aguilar et al. 2010, Trunzo et al. 2009).

For interventional and conservative treatment of a mesh infection after laparoscopic repair of incisional and ventral hernias, Aguilar et al (2010) and Trunzo et al. (2009) propose percutaneous drainage of accumulated pus around the mesh and insertion of a drain. Via this drain, irrigation with gentamycin 80 mg in 20 ml saline solution is then carried out three times daily as well as administration of antibiotic intravenous treatment.

But treatment of mesh infection also depends on the material used. In a comparative study Hawn et al (2011) demonstrated (Level 2B) that there was significantly less need to remove a polypropylene mesh than a PTFE - mesh because of a mesh infection (p<0.0001). Petersen et al. (2001) also showed that for mesh repair of incisional hernias, in which mesh infection occurred in 8.1 % of cases after using ePTFE and in 3.9 % on using polypropylene, in no case was it possible to salvage the infected ePTFE mesh, whereas all infected polypropylene meshes were preserved in the body. Hence the chances of mesh salvage after infection are greater in the case of polypropylene meshes than ePTFE meshes, which generally have to be explanted.

If an interventional conservative attempt at treating a mesh infection after laparoscopic IPOM has not proved successful or if from the outset the circumstances are such that it is no longer possible to preserve the mesh, there are various options that can be used for mesh infections after mesh repair of incisional and ventral hernias (Saettele et al. 2007, Sanchez et al. 2011, Baharestani al 2010, Tamhankar et al. 2009).

To that effect, the following options have been proposed:

- Mesh removal with primary skin closure – Repeat repair of the defect after 6-9 months.
- Mesh removal – component separation technique – Leave skin open - Vacuum-assisted wound closure or open wound dressing.
- Mesh removal – Repair of defect with a biological mesh - Leave skin open - Vacuum-assisted wound closure or open wound dressing.
- Mesh salvage - Leave skin open - Vacuum-assisted wound closure or open wound dressing.

Since the treatment options available in the literature relate only to individual cases or to small case series, at present no concrete recommendation invoking evidence-based data can be given as regards which method produces the best results. Rather, the treating surgeon must decide in the individual case which option is best for the individual patient. There is an absolute need for further studies.

**References**

1. Forbes SS, Eskicioglu C, McLeod RS, Okrainec A Meta-analysis of randomized controlled trials comparing open and laparoscopic ventral and incisional hernia repair with mesh. British Journal of Surgery (2009) 96:851-858 **(level 1A)**

2. Sauerland S, Walgenbach M, Habermalz B, Seiler CM, Miszerez M Laparoscopic versus open surgical techniques for ventral or incisional hernia repair (Review) The Cochrane Collaboration. Published by John Wiley & Sons, Ltd. (2011) **(level IA)**

3. Pierce RA, Spitler JA, Frisella MM, Matthews BD, Brunt LM Pooled data analysis of laparoscopic vs. open ventral hernia repair: 14 years of patient data accrual. Surg Endosc (2007), 21:378-386 **(level 2A)**

4. Carlson MA, Frantzides CT, Shostrom VK, Laguna LE Minimally invasive ventral herniorrhaphy: an analysis of 6.266 published cases. Hernia (2008) 12:9-22 **(level 2A)**

5. Heniford BT, Park A, Ramshaw BJ, Voeller G Laparoscopic Repair of Ventral Hernias. Nine Years Experience With 850 Consecutive Hernias. Annals of Surgery (2003), Volume 238, Number 3, 391-400 **(level 4)**

6. Fortelny RH, Petter-Puchner AH, Glaser KS, Offner F, Benesch T, Rohr M Adverse effects of polyvinylidene fluoride-coated polypropylene mesh used for laparscopic intraperitoneal onlay repair of incisional hernia. British Journal of Surgery (2010) 97:1140-1145 **(level 4)**

7. Perrone JM, Soper NJ, Eagon C, Klingensmith ME, Aft RL, Frisella MM, Brunt LM Perioperative outcomes and complications of laparoscopic ventral hernia repair. Surgery (2005), Volume 138, Number 4, 708-716 **(level 4)**

8. Aguila B, Chapital AB, Madura JA, Harold KL Conservative Management of Mesh-Site Infection in Hernia Repair. Journal of Laparoendoscopic & Advanced Surgical Techniques (2010), Volume 20, Number 3, 249-253 **(level 5)**

9. Trunzo JA, Ponsky JL, Jin J. Williams CP, Rosen MJ A novel approach for salvaging infected prosthetic mesh after ventral hernia repair. Hernia (2009), 13:545-549 **(level 5)**

10. Hawn MT, Gray SH, Snyder CW, Graham LA, Finan KR, Vick CC Predictors of mesh explantation after incisional hernia repair. The American Journal of Surgery (2011) 202:28-33 **(level 2B)**

11. Petersen S, Henke G, Freitag M, Faulhaber A, Ludwig K Deep Prosthesis Infection in Incisional Hernia Repair: predictive Factors and Clinical Outcome. Eur J Surg (2001),167:453-457 **(level 3)**

12. Saettele TM, Bachmann SL, Costello CR, Grant SA, Cleveland DS, Loy TS, Kolder DG, Ramshaw BJ Use of porcine dermal collagen as a prosthetic mesh in a contaminated field for ventral hernia repair: a case report. Hernia (2007), 11:279-285 **(level 5)**

13. Sanchez VM, Abi-Haidar YE, Itani KMF Mesh Infection in Ventral Incisional Hernia Repair: Incidence, Contributing Factors and Treatment. Surgical Infections (2011), Volume 12, Number 3, 205-210 **(level 5)**

14. Baharestani MM, Gabriel A Use of negative pressure wound therapy in the management of infected abdominal wounds containing mesh: an analysis of outcomes. International Wound Journal (2010), Vol 8, No 2, 118-125 **(level 4)**

15. Tamhankar AP, Ravi K, Everitt NJ Vacuum Assisted Closure Therapy In The Treatment Of Mesh Infection After Hernia Repair. Surgeon 7 (2009), 5:316-318 **(level 5)**

**Postoperative Seroma: Risk factors, prevention and best treatment**

J. Bingener, M. Rohr

*Search terms****: “****hernia” AND “ventral and laparoscopy” AND “laparoscopic surgery and seroma” AND “ incisional hernia and abdominal wall hernia and laparoscopy/or laparoscopic surgery/or hernioplasty”.*

This resulted in a total of 946 citations from Ovid medliner 1948 – August 2011, PubMed including prepublication, Embase 1988 – 33^rd^ week of 2011, evidence-based medicine reviews and the Cochrane register, and the Web of Science from 1993 – 2011.

The literature reviewed for the incidence, risk factors and treatment of seroma included 27 studies.[[1-27](#_ENREF_1)]

From the review resulted the following statements and recommendations:

**Incidence**

**Statements**

| Level 4 | Seroma can be found in up to 100% of patients by ultrasound |
| --- | --- |
| Level 4 | Seroma formation peaks around postoperative day 7 |
| Level 4 | Seroma resolution is almost complet4e at 90 days |
| Level 2B | Up to 30% of patients become symptomatic from the seroma |

**Recommendations**

| Grade B | Patients should be informed about likely (asymptomatic seroma formation) |
| --- | --- |

The majority of the studies encountered were retrospective in nature. Two were prospective studies, one was a cohort comparison and one a prospective cohort study. The remainder were retrospective studies out of which three were retrospective cohort comparisons. [[1-21](#_ENREF_1)]

The incidence of seroma after laparoscopic ventral hernia repair is reported with wide spectrum (3 – 100%) with a peak presentation at seven days postoperatively and almost complete resolution at 90 days postoperatively.[[1](#_ENREF_1), [3](#_ENREF_3), [5](#_ENREF_5), [6](#_ENREF_6), [8](#_ENREF_8), [25](#_ENREF_25)] This introduces the question whether all seromas are a complication or just a consequence of laparoscopic ventral incisional hernia repair. In the current surgical literature this is not well defined. Up to 35% of patients will become symptomatic with pain or pressure and erythema.[[6](#_ENREF_6)] Some will develop chronic seroma. Most studies reviewed for these guidelines do not distinguish between clinically significant or asymptomatic seroma.

While clinical retrospective studies often report the incidence of seroma to be 4 to 78%, [[14](#_ENREF_14), [18](#_ENREF_18), [22](#_ENREF_22), [26](#_ENREF_26)] a prospective study with close and ongoing ultrasound followup described the incidence of seroma 100% at seven days, with all but complete spontaneous resolution at 90 days.[[6](#_ENREF_6)] The study employed mesh, tacks and sutures. Up to 30% of patients become symptomatic from the seroma.[[11](#_ENREF_11)]

**Risk Factors**

**Statements**

| Level 2B | Laparoscopic vs open repair (trials with opposing results) |
| --- | --- |
| Level 2B | Non-reducible hernia is a risk factor |
| Level 3 | Seroma may be more common on IPOM compared to TAPP LVHR |
| Level 2B | Increased number of prior abdominal incisions |
| Level 2B | Hospital center (within VA system) with independent predictor of seroma |
| Level 5 | Sutures through hernia sac predispose for sustained seroma |

A large VA study identified the risk factors for seroma for both open and laparoscopic hernia repair: a non-reducible hernia, an increased number of prior abdominal incisions and the hospital center within the VA system as independent predictor of seroma.[[1](#_ENREF_1)] The finding of hospital centers being linked to seroma formation suggests that intraoperative technical factors may play a role. The transabdominal preperitoneal repair for primary ventral and umbilical hernias may decrease the likelihood of seroma formation.[[8](#_ENREF_8)] Randomized trials have conflicting results regarding the likelihood of seroma formation with the laparoscopic or open repair.[[3](#_ENREF_3), [4](#_ENREF_4)]

**Prevention**

**Statements**

| Level 2B | Cauterizing the hernia sac may lead to less seroma formation |
| --- | --- |
| Level 2B | Placing a quilting stitch did not affect seroma formation |
| Level 2B | Double crown stapling did not decrease seroma formation |
| Level 4 | No specific mesh is related to seroma formation |
| Level 4 | Compression dressing for 1 week reduces occurrence of seroma |

**Recommendations**

| Grade C | Surgeons can attempt cauterization of the hernia sac to prevent seroma formation |
| --- | --- |
| Grade C (D?) | Surgeons can attempt to place a pressure dressing |

To prevent the empty space created by the hernia repair from being filled with serous fluid, a number of strategies have been examined. A small randomized study determined that if the hernia sac was cauterized by electro-cautery or ultrasonic energy the seroma frequency was decreased from 25% to 4%. The trial had some methodological limitations (JADAD Score:0).[[27](#_ENREF_27)] Other similar trials have reported that placing a quilting stitch or double crown stapling to decrease the dead space did not affect seroma formation.[[7](#_ENREF_7)] Studies often suffer from small numbers. One study reported that the placement of a compression dressing for one week reduced the occurrence of seroma (24).

**Treatment**

**Statements**

|  | |
| --- | --- |
| Level 2B | The majority of seromas resolve spontaneously |
| Level 2B | Length of abdominal binder use does not affect seroma formation |
| Level 4 | Aspiration is often effective |
| Level 4 | Repeated aspiration may lead to mesh infection |

**Recommendations**

| Grade B | The majority of seromas should be expected to resolve spontaneously |
| --- | --- |
| Grade (B) | Patients may (should) be informed about the risk of infection if a seroma is repeatedly aspirated |

The recommendations are strongest for informing patients about the possible occurrence of seromas and the expectation that the majority will resolve spontaneously. ( 1,2,6, 25) Given the clinically important consequences of mesh infection as a possible complication of repeated seroma aspiration, this recommendation may also be considered stronger (level B) although it is based only on level 4 evidence. (1,14)

The importance of applying a pressure dressing was supported by one study with methodological limitations (24) and may be contradicted by the findings regarding binder placement, a circumferential pressure dressing. The strength of the recommendation may therefore be downgraded (level D).

**Commentary**

Seroma after laparoscopic ventral hernia repair can be demonstrated by ultrasound in up to 100% of patients with up to 30% of patients becoming symptomatic. However, high level evidence regarding the risk factors, prevention and treatment of postoperative seroma is missing. Contributing to this is the lack of a uniform definition (symptomatic vs asymptomatic seroma, timeline of seroma formation). Further, the detection of seroma can be difficult and subjective. Using imaging studies such as ultrasound provides a more quantitative assessment but may be too resource intense to use on a frequent basis.

**References**

1. Kaafarani HM, Hur K, Hirter A, Kim LT, Thomas A, Berger DH, Reda D, Itani KM (2009) Seroma in ventral incisional herniorrhaphy: incidence, predictors and outcome. Am J Surg 198**:**639-644 **(level 1B)**

2. Tsimoyiannis EC, Siakas P, Glantzounis G, Koulas S, Mavridou P, Gossios KI (2001) Seroma in laparoscopic ventral hernioplasty. Surg Laparosc Endosc Percutan Tech 11**:**317-321 **(level 2B)**

3. Forbes SS, Eskicioglu C, McLeod RS, Okrainec A (2009) Meta-analysis of randomized controlled trials comparing open and laparoscopic ventral and incisional hernia repair with mesh. Br J Surg 96**:**851-858 **(level 2A)**

4. Kapischke M, Schulz T, Schipper T, Tensfeldt J, Caliebe A (2008) Open versus laparoscopic incisional hernia repair: something different from a meta-analysis. Surg Endosc 22**:**2251-2260 **(level 2A)**

5. Palanivelu C, Jani KV, Senthilnathan P, Parthasarathi R, Madhankumar MV, Malladi VK (2007) Laparoscopic sutured closure with mesh reinforcement of incisional hernias. Hernia 11**:**223-228 **(level 4)**

6. Susmallian S, Gewurtz G, Ezri T, Charuzi I (2001) Seroma after laparoscopic repair of hernia with PTFE patch: is it really a complication? Hernia 5**:**139-141 **(level 4)**

7. Tsimoyiannis EC, Tsimogiannis KE, Pappas-Gogos G, Nikas K, Karfis E, Sioziou H (2008) Seroma and recurrence in laparoscopic ventral hernioplasty. JSLS 12**:**51-57 **(level 2B)**

8. Prasad P, Tantia O, Patle NM, Khanna S, Sen B (2011) Laparoscopic ventral hernia repair: a comparative study of transabdominal preperitoneal versus intraperitoneal onlay mesh repair. J Laparoendosc Adv Surg Tech A

21**:**477-483 **(level 3)**

9. Razman J, Shaharin S, Lukman MR, Sukumar N, Jasmi AY (2006) Initial experience of laparoscopic incisional hernia repair. Med J Malaysia

61**:**142-146 **(level 4)**

10. Sanchez LJ, Bencini L, Moretti R (2004) Recurrences after laparoscopic ventral hernia repair: results and critical review. Hernia 8**:**138-143 **(level 4)**

11. Toy FK, Bailey RW, Carey S, Chappuis CW, Gagner M, Josephs LG, Mangiante EC, Park AE, Pomp A, Smoot RT, Jr., Uddo JF, Jr., Voeller GR (1998) Prospective, multicenter study of laparoscopic ventral hernioplasty. Preliminary results. Surg Endosc 12**:**955-959 **(level 4)**

12. Uranues S, Salehi B, Bergamaschi R (2008) Adverse events, quality of life, and recurrence rates after laparoscopic adhesiolysis and recurrent incisional hernia mesh repair in patients with previous failed repairs. J Am Coll Surg

207**:**663-669 **(level 4)**

13. Bingener J, Buck L, Richards M, Michalek J, Schwesinger W, Sirinek K (2007) Long-term outcomes in laparoscopic vs open ventral hernia repair. Arch Surg 142**:**562-567 **(level 3)**

14. Olmi S, Erba L, Magnone S, Bertolini A, Croce E (2006) Prospective clinical study of laparoscopic treatment of incisional and ventral hernia using a composite mesh: indications, complications and results. Hernia

10**:**243-247 **(level 4)**

15. Tagaya N, Mikami H, Aoki H, Kubota K (2004) Long-term complications of laparoscopic ventral and incisional hernia repair. Surg Laparosc Endosc Percutan Tech 14**:**5-8 **(level 4)**

16. Lau H, Patil NG, Yuen WK, Lee F (2002) Laparoscopic incisional hernioplasty utilising on-lay expanded polytetrafluoroethylene DualMesh: prospective study. Hong Kong Med J 8**:**413-417 **(level 4)**

17. Ben-Haim M, Kuriansky J, Tal R, Zmora O, Mintz Y, Rosin D, Ayalon A, Shabtai M (2002) Pitfalls and complications with laparoscopic intraperitoneal expanded polytetrafluoroethylene patch repair of postoperative ventral hernia. Surg Endosc 16**:**785-788 **(level 4)**

18. Birch DW (2007) Characterizing laparoscopic incisional hernia repair. Can J Surg 50**:**195-201 **(level 4)**

19. Edwards C, Angstadt J, Whipple O, Grau R (2005) Laparoscopic ventral hernia repair: postoperative antibiotics decrease incidence of seroma-related cellulitis. Am Surg 71**:**931-935; discussion 935-936 **(level 3)**

20. Eid GM, Prince JM, Mattar SG, Hamad G, Ikrammudin S, Schauer PR (2003) Medium-term follow-up confirms the safety and durability of laparoscopic ventral hernia repair with PTFE. Surgery 134**:**599-603; discussion 603-594 **(level 4)**

21. Ferranti F, Passa G, Stefanuto A, Quintiliani A (2008) Laparoscopic incisional hernia repair: our experience with 105 consecutive cases. Chir Ital

60**:**249-255 **(level 4)**

22. Heniford BT, Park A, Ramshaw BJ, Voeller G (2003) Laparoscopic repair of ventral hernias: nine years' experience with 850 consecutive hernias. Ann Surg 238**:**391-399; discussion 399-400 **(level 4)**

23. Parker HH, 3rd, Nottingham JM, Bynoe RP, Yost MJ (2002) Laparoscopic repair of large incisional hernias. Am Surg 68**:**530-533; discussion 533-534 **(level 4)**

24. Sharma A, Mehrotra M, Khullar R, Soni V, Baijal M, Chowbey PK (2011) Laparoscopic ventral/incisional hernia repair: a single centre experience of 1,242 patients over a period of 13 years. Hernia 15**:**131-139 **(level 3)**

25. Sodergren MH, Swift I (2010) Seroma formation and method of mesh fixation in laparoscopic ventral hernia repair--highlights of a case series. Scand J Surg 99**:**24-27 **(level 4)**

26. Yavuz N, Ipek T, As A, Kapan M, Eyuboglu E, Erguney S (2005) Laparoscopic repair of ventral and incisional hernias: our experience in 150 patients. J Laparoendosc Adv Surg Tech A 15**:**601-605 **(level 4)**

27. Jadad AR, Moore RA, Carroll D, Jenkinson C, Reynolds DJ, Gavaghan DJ, McQuay HJ (1996) Assessing the quality of reports of randomized clinical trials: is blinding necessary? Controlled clinical trials 17**:**1-12

**Postoperative bulging**

Rohr, M.

*Search terms : “laparoscopic hernia repair“ AND “LVHR“ AND “incisional hernia“ AND “ventral hernia“ AND “postoperative bulging abdominal wall“ AND “abdominal wall bulging“ AND “abdominal wall hernia and bulging“ AND “complication bulging“ AND “incisional hernia and bulging” AND “bulging after hernia repair” AND “long term results”*

A systemic search of the available literature was performed in August 2011 using Medline, PubMed, Cochrane library and relevant journals and reference lists using the above listed search terms

54 articles were found, only 4 were fitting to the item „bulging” in laparoscopic hernia repair.

**Key questions:**

**A real problem? Is it avoidable?**

**Statements**

| **Level 2B** | Abdominal bulging is a specific problem associated with laparoscopic repair of large incisional hernias.  In 1.6%- 17.4 % of the patients bulging is to be observed after laparoscopic ventral/incisional hernia repair.  Symptomatic bulging is rare. |
| --- | --- |
| **Level 2C** | Symptomatic bulging, though not a recurrence, is an important negative outcome of laparoscopic ventral hernia repair. |

**Recommendations**

| **Grade B** | Symptomatic bulging, though not a recurrence, requires a new repair . |
| --- | --- |
| **Grade B** | In asymptomatic patients, “watch-full waiting” seems justified. |

**Introduction:**

Besides pain, patients sometimes complain about the presence of postoperative abdominal bulging, which can be cosmetically dissatisfying. The anatomical basis for this problem lies in the fact, that neither hernia orifice nor rectus diastasis (if present) is being closed during laparoscopic hernia repair. These issues which are relevant mainly in large hernias should be discussed with the patient preoperatively. (1)

**Bulging: is it a real problem and avoilable?**

To investigate the prevalence, diagnosis, clinical significance, and treatment strategies for bulging in the area of laparoscopic repair of ventral hernia that is caused by mesh protrusion through the hernia opening, but with intact peripheral fixation of the mesh and actually a still sufficient repair. (2) In the study of Schoenmaeckers (2) 765 patients who underwent laparoscopic ventral hernia repair were reviewed, and all patients with a swelling in the repaired area were identified and analyzed. He found twenty-nine patients with a swelling in the original hernia area. They all underwent a computed tomography assessment. Seventeen patients (2.2% of the total group) had a hernia recurrence; in an additional 12 patients (1.6%), radiologic examinations indicated only bulging of the mesh but no recurrence. Bulging was associated with pain in 4 patients who underwent relaparoscopy and got a new, larger mesh tightly stretched over the entire previous repair. Eight asymptomatic patients decided on “watchful waiting.” All patients remained symptom free during a median follow-up of 22 months. Symptomatic bulging requires a new repair and must be considered as an important negative outcome of laparoscopic ventral hernia repair. In asymptomatic patients, “watch-full waiting” seems justified. (2)

In the study of Kurmann et al. (3) long-term results after laparoscopic repair of large incisional hernias remain to be determined. The aim of this prospective study was to compare early and late complications between laparoscopic repair and open repair in patients with large incisional hernias. Comparison of 56 patients with a hernia diameter of ≥5 cm who underwent open incisional hernia repair with 69 patients who underwent laparoscopic repair. Prospectively followed with a median follow-up in the laparoscopic group of 32.5 months (1-62 months) vs. 65 months (1-80 months) in the open group. (3) The demographic parameters were not significantly different between the two groups. At long-term follow-up, the recurrence rate was not different between the two techniques but abdominal bulging is a specific problem associated with laparoscopic repair of large incisional hernias ( 17.4% in laparoscopic repair vs. 7.1% in open). (3)

To reduce frequencies of seromas or bulging Orenstein et al. (4) have modified their approach to LVHR to routinely utilize transabdominal defect closure ("shoelacing" technique) prior to mesh placement. Forty seven consecutive patients undergoing LVHR with shoelacing were reviewed retrospectively. Main outcome measures included patient demographics, previous surgical history, intraoperative time, mesh type and size, postoperative complications, length of hospitalization, and hernia recurrence. All the patients underwent LVHR with defect closure. LVHR with defect closure confers a strong advantage in hernia repair, shifting the paradigm towards more physiologic abdominal wall reconstruction. In their series, Orentsten et al. found this approach to be safe and comparable to historic controls. While providing reliable hernia repair, the addition of defect closure in their patients essentially eliminated postoperative seroma. Therefore the authors advocate routine use of the shoelace technique during laparoscopic ventral hernia repair.

**Comment:**

Symptomatic bulging, though not a recurrence, requires a new repair and must be considered as an important negative outcome of laparoscopic ventral hernia repair. In asymptomatic patients, “watch-ful waiting” seems justified. Abdominal bulging is a specific problem associated with laparoscopic repair of large incisional hernias. It occurs in between 2% and 20% of the patients, apparently in dependence on how careful it is looked for. But evidence is limited. There is an urgent need for more studies regarding this topic.

**Literature:**

1. Sauerland S, Walgenbach M, Habermalz B, Seiler CM, Miserez M Laparoscopic versus open surgical techniques for ventral or incisional hernia repair (Review) The Cochrane Collaboration. Published by John Wiley & Sons, Ltd. (2011) **(level IA)**
2. Schoenmaeckers EJ, Wassenaar EB, Raymarkers JT, Rakic S Bulging of the mesh after laparoscopic repair of ventral and incisional hernias . JSLS 2010 Oct-Dez 2010, 14 (4), 541-6 **(level 2C)**
3. Kurmann A, Visth E, Candinas D, Beldi G. Long-term follow-up of open and laparoscopic repair of large incisional hernias. World J Surg. 2011 Feb., 35(2):297-301. **(level 2B)**
4. Orenstein SB, Dumeer JL, Montegudo J, Poi MJ, Novitsky YW Outcome of laparoscopic ventral hernia repair with routine defect closure using “shoelacing” technique. Surg Endosc, 2011 25 (5):1452-7 **(level 4)**

**Complications: Chronic Pain – Risk Factors, Prevention and**

**Treatment**

J. Bingener, W. Reinpold, P. Chowbey

*Search terms: “hernia” AND “ventral laparoscopy” AND “laparoscopic surgery” AND “postoperative complications or recurrence or pain” AND “postoperative or surgical wound infection” AND “prosthesis design/failure/implantation/device removal” AND “pain”*

This resulted in a total of 946 citations from Ovid medliner 1948 – August 2011, PubMed including prepublication, Embase 1988 – 33^rd^ week of 2011, evidence-based medicine reviews and the Cochrane register, and the Web of Science from 1993 – 2011.

The topic chronic pain after laparoscopic ventral hernia repair was addressed by 3 meta analysis/systematic review, 13 RCT, 5 comparative cohort studies, 19 single cohort studies. [[4-43](#_ENREF_4)] The Oxford classification of trials was used (list A). The randomized trials were of fair to poor quality, which influenced the levels of evidence assigned to the statements and recommendations. In addition, 2 state-of-the-art perioperative pain factors reviews/study assessment reviews were accessed, [[2](#_ENREF_2), [3](#_ENREF_3)] which included non-procedure specific findings and recommendations. In some cases extrapolation from inguinal hernia trials may be appropriate. From the review result the following statements and recommendations.

**Risk Factors**

**Statements**

| Level 2A | LVHR results in chronic pain in 2-4% of patients |
| --- | --- |
| Level 2C | Recurrence is associated with chronic pain (open and laparoscopic) |
| Level 3 | Non-midline laparoscopic ventral hernia repair is more often associated with chronic pain |
| Level 4 | LVHR may lead to residual pain in up to 26% of patients |
| Level 2B | Acute postoperative pain (non-procedure specific) |

**Non-Procedure Specific Risk Factors**

**Statements**

| Level 2B | Age |
| --- | --- |
| Level 2B | Gender |
| Level 2B | Preoperative pain |
| Level 2B | Psychosocial factors |
| Level 2B | Catastrophizing |

**Prevention**

**Statements**

| Level 2B | Local anesthetic at suture sites during surgery significantly decreases acute early pain |
| --- | --- |
| Level 2B | No difference in acute or chronic pain with pain pump placement |
| Level 4 | Tissue glue resulted in “low levels of postoperative pain” |
| Level 2B | No difference in VAS between absorbable and permanent fixation sutures at 3 months, but QOL differences (physical activity) |
| Level 2B | No correlation with number of tacks |
| Level 3 | No consistent difference between PP and other LW meshes in pain scores |
| Level 4 | Absorbable fixation tacks were associated with few cases of chronic pain at 1 year |
| Level 2A | Transfascial sutures with tacks do not result in higher pain scores than tacks only |
| Level 2B | Permanent suture fixation at 2-3 cm intervals results in higher number of patients with pain 6 months postoperatively compared to tack only fixation |
| Level 2B | Permanent suture fixation pain frequency at 6 months was similar to tacks only fixation |
| Level 2B | Permanent corner suture plus double crown tacks resulted in higher VAS scores compared to permanent sutures only in <5 cm |

**Recommendations**

| Grade B | Patients should be informed that laparoscopic ventral hernia repair may lead to prolonged pain |
| --- | --- |
| Grade B | Surgeons should strive to limit acute pain as a risk factor for chronic pain |
| Grade B | Surgeons should use intraoperative suture site injection of local anesthetic |
| Grade D | Inconclusive evidence exists whether the type of suture, tacks, glue or mesh alters the likelihood of chronic pain |

**Treatment**

**Statements**

| Level 2B | Lidocaine patch did not significantly reduce postoperative acute or chronic pain |
| --- | --- |
| Level 4 | Local injection after surgery at suture sites can resolve pain |
| Level 4 | Suture removal can resolve chronic pain |
| Level 4 | Mesh removal can resolve chronic pain |
| Level 4 | Multimodality pain treatment can resolve chronic pain |

**Recommendations**

| Grade C | To treat chronic pain, local anesthetic injection at suture sites can be considered |
| --- | --- |
| Grade C | To treat chronic pain, suture, tack or mesh removal can be considered |
| Grade C | To treat chronic pain, multimodality pain treatment should be considered |

**Introduction**

It is well established that surgical injury can lead to chronic pain, which is defined as pain lasting for 3 months or more by the International Association for the Study of Pain (IASP).[[1](#_ENREF_1)]

The components and risk factors for postoperative pain can be subdivided into 1) patient factors, 2) intraoperative factors: tissue damage, mesh type, type of anesthesia 3) postoperative factors such as type of analgesia. Patient factors [[2](#_ENREF_2), [3](#_ENREF_3)] including catastrophizing contribute to postoperative pain perception but were not investigated in the studies available for review.

The studies that were reviewed for this topic showed substantial heterogeneity, with varying definitions of pain. The definition of chronic/prolonged pain was often vague, ranging between > 24 hrs and > 6 months. Furthermore, the trial designs and reporting were not uniform, further limiting the comparability of the outcomes. This is also noted in the meta-analysis available for laparoscopic ventral hernia repair. [[4-6](#_ENREF_4)]

Specific studies examining chronic pain in patients with ventral hernia repair are not frequent. We may be able to extrapolate some findings from other studies relevant to the assessment of pain syndromes and chronic pain. In inguinal hernia repair, e.g., other preoperative chronic pain conditions not related to the groin are a risk factor for chronic postoperative groin pain. In one controlled randomized trial comparing open and endoscopic groin hernia repair with a five- year follow-up, other previous pain syndromes were a significant risk factor for chronic pain (p<0.01).[[44](#_ENREF_44)] Two retrospective studies of patients with severe chronic postoperative groin pain mostly after open groin hernia repair are in accordance with these findings.[[45](#_ENREF_45), [46](#_ENREF_46)]

While several publications have reported that severe early postoperative pain after groin hernia repair is significantly associated with chronic pain,[[46](#_ENREF_46), [47](#_ENREF_47)] only few publications are available on high acute pain rates and chronic pain after endoscopic hernia repair. In a randomized controlled trial, Berndsen et al. [[48](#_ENREF_48)] found that severe early postoperative pain was a risk factor for chronic pain after Shouldice repair, but not after TAPP repair. However, two prospective non-randomized studies [[49](#_ENREF_49), [50](#_ENREF_50)] of 313 and 123 patients respectively reported that severe early postoperative pain was a significant risk factor for chronic pain after endoscopic hernia repair (p<0.05 and p<0.03 respectively). These findings may also be applicable in ventral hernia repair but have not been specifically studied.

**References**

1. Aasvang E KH (1986) Classification of chronic pain. Descriptions of chronic pain syndromes and definitions of pain terms. Prepared by the International Association for the Study of Pain. SUbcommittee on Taxonomy. Oaub Syook 3**:**1-226

2. Kehlet H, Rathmell JP (2010) Persistent postsurgical pain: the path forward through better design of clinical studies. Anesthesiology 112**:**514-515 **(level 5)**

3. Khan RS, Ahmed K, Blakeway E, Skapinakis P, Nihoyannopoulos L, Macleod K, Sevdalis N, Ashrafian H, Platt M, Darzi A, Athanasiou T (2011) Catastrophizing: a predictive factor for postoperative pain. Am J Surg 201**:**122-131 **(level 2A)**

4. Sauerland S, Walgenbach M, Habermalz B, Seiler CM, Miserez M (2011) Laparoscopic versus open surgical techniques for ventral or incisional hernia repair. Cochrane Database Syst Rev**:**CD007781 **(level 2)**

5. Muller-Riemenschneider F, Roll S, Friedrich M, Zieren J, Reinhold T, von der Schulenburg JM, Greiner W, Willich SN (2007) Medical effectiveness and safety of conventional compared to laparoscopic incisional hernia repair: a systematic review. Surg Endosc 21**:**2127-2136 **(level 2A)**

6. Sajid MS, Bokhari SA, Mallick AS, Cheek E, Baig MK (2009) Laparoscopic versus open repair of incisional/ventral hernia: a meta-analysis. Am J Surg 197**:**64-72 **(level 2A)**

7. Olmi S, Erba L, Magnone S, Bertolini A, Croce E (2006) Prospective clinical study of laparoscopic treatment of incisional and ventral hernia using a composite mesh: indications, complications and results. Hernia 10**:**243-247 **(level 4)**

8. Lau H, Patil NG, Yuen WK, Lee F (2002) Laparoscopic incisional hernioplasty utilising on-lay expanded polytetrafluoroethylene DualMesh: prospective study. Hong Kong Med J 8**:**413-417 **(level 4)**

9. Eid GM, Prince JM, Mattar SG, Hamad G, Ikrammudin S, Schauer PR (2003) Medium-term follow-up confirms the safety and durability of laparoscopic ventral hernia repair with PTFE. Surgery 134**:**599-603; discussion 603-594 **(level 4)**

10. Parker HH, 3rd, Nottingham JM, Bynoe RP, Yost MJ (2002) Laparoscopic repair of large incisional hernias. Am Surg 68**:**530-533; discussion 533-534 **(level 4)**

11. Sharma A, Mehrotra M, Khullar R, Soni V, Baijal M, Chowbey PK (2011) Laparoscopic ventral/incisional hernia repair: a single centre experience of 1,242 patients over a period of 13 years. Hernia 15**:**131-139 **(level 4)**

12. Wassenaar E, Schoenmaeckers E, Raymakers J, van der Palen J, Rakic S (2010) Mesh-fixation method and pain and quality of life after laparoscopic ventral or incisional hernia repair: a randomized trial of three fixation techniques. Surg Endosc 24**:**1296-1302 **(level 2B)**

13. Snyder CW, Graham LA, Vick CC, Gray SH, Finan KR, Hawn MT (2011) Patient satisfaction, chronic pain, and quality of life after elective incisional hernia repair: effects of recurrence and repair technique. Hernia 15**:**123-129 **(level 2C)**

14. Rosen MJ, Duperier T, Marks J, Onders R, Hardacre J, Ponsky J, Ermlich B, Laughinghouse M (2009) Prospective randomized double-blind placebo-controlled trial of postoperative elastomeric pain pump devices used after laparoscopic ventral hernia repair. Surg Endosc 23**:**2637-2643 **(level 1B)**

15. Nguyen SQ, Divino CM, Buch KE, Schnur J, Weber KJ, Katz LB, Reiner MA, Aldoroty RA, Herron DM (2008) Postoperative pain after laparoscopic ventral hernia repair: a prospective comparison of sutures versus tacks. JSLS 12**:**113-116 **(level 2B)**

16. Moreno-Egea A, Carrillo A, Aguayo JL (2008) Midline versus nonmidline laparoscopic incisional hernioplasty: a comparative study. Surg Endosc 22**:**744-749 **(level 3)**

17. Lepere M, Benchetrit S, Bertrand JC, Chalbet JY, Combier JP, Detruit B, Herbault G, Jarsaillon P, Lagoutte J, Levard H, Rignier P (2008) Laparoscopic resorbable mesh fixation. Assessment of an innovative disposable instrument delivering resorbable fixation devices: I-Clip(TM). Final results of a prospective multicentre clinical trial. Hernia 12**:**177-183 **(level 4)**

18. Johanet H, Dabrowski A, Hauters P (2006) Laparoscopic cure of small ventral hernias with composite mesh. Hernia 10**:**414-418 **(level 4)**

19. Itani KM, Hur K, Kim LT, Anthony T, Berger DH, Reda D, Neumayer L (2010) Comparison of laparoscopic and open repair with mesh for the treatment of ventral incisional hernia: a randomized trial. Arch Surg 145**:**322-328; discussion 328 **(level 1B)**

20. Hope WW, Lincourt AE, Newcomb WL, Schmelzer TM, Kercher KW, Heniford BT (2008) Comparing quality-of-life outcomes in symptomatic patients undergoing laparoscopic or open ventral hernia repair. J Laparoendosc Adv Surg Tech A 18**:**567-571 **(level 3)**

21. Eriksen JR, Poornoroozy P, Jorgensen LN, Jacobsen B, Friis-Andersen HU, Rosenberg J (2009) Pain, quality of life and recovery after laparoscopic ventral hernia repair. Hernia 13**:**13-21 **(level 4)**

22. Chelala E, Thoma M, Tatete B, Lemye AC, Dessily M, Alle JL (2007) The suturing concept for laparoscopic mesh fixation in ventral and incisional hernia repair: Mid-term analysis of 400 cases. Surg Endosc 21**:**391-395 **(level 4)**

23. Carbonell AM, Harold KL, Mahmutovic AJ, Hassan R, Matthews BD, Kercher KW, Sing RF, Heniford BT (2003) Local injection for the treatment of suture site pain after laparoscopic ventral hernia repair. Am Surg 69**:**688-691; discussion 691-682 **(level 4)**

24. Bellows CF, Berger DH (2006) Infiltration of suture sites with local anesthesia for management of pain following laparoscopic ventral hernia repairs: a prospective randomized trial. JSLS 10**:**345-350 **(level 2B)**

25. Beldi G, Wagner M, Bruegger LE, Kurmann A, Candinas D (2011) Mesh shrinkage and pain in laparoscopic ventral hernia repair: a randomized clinical trial comparing suture versus tack mesh fixation. Surg Endosc 25**:**749-755 **(level 2B)**

26. Bansal VK, Misra MC, Kumar S, Rao YK, Singhal P, Goswami A, Guleria S, Arora MK, Chabra A (2011) A prospective randomized study comparing suture mesh fixation versus tacker mesh fixation for laparoscopic repair of incisional and ventral hernias. Surg Endosc 25**:**1431-1438 **(level 2B)**

27. Bageacu S, Blanc P, Breton C, Gonzales M, Porcheron J, Chabert M, Balique JG (2002) Laparoscopic repair of incisional hernia: a retrospective study of 159 patients. Surg Endosc 16**:**345-348 **(level 4)**

28. Wolter A, Rudroff C, Sauerland S, Heiss MM (2009) Laparoscopic incisional hernia repair: evaluation of effectiveness and experiences. Hernia 13**:**469-474 **(level 3)**

29. Pring CM, Tran V, O'Rourke N, Martin IJ (2008) Laparoscopic versus open ventral hernia repair: a randomized controlled trial. ANZ J Surg 78**:**903-906 **(level 2B)**

30. Pierce RA, Spitler JA, Frisella MM, Matthews BD, Brunt LM (2007) Pooled data analysis of laparoscopic vs. open ventral hernia repair: 14 years of patient data accrual. Surg Endosc 21**:**378-386 **(level 2C)**

31. Moreno-Egea A, Bustos JA, Girela E, Aguayo-Albasini JL (2010) Long-term results of laparoscopic repair of incisional hernias using an intraperitoneal composite mesh. Surg Endosc 24**:**359-365 **(level4)**

32. Misiakos EP, Machairas A, Patapis P, Liakakos T (2008) Laparoscopic ventral hernia repair: pros and cons compared with open hernia repair. JSLS 12**:**117-125 **(level 5)**

33. Misra MC, Bansal VK, Kulkarni MP, Pawar DK (2006) Comparison of laparoscopic and open repair of incisional and primary ventral hernia: results of a prospective randomized study. Surg Endosc 20**:**1839-1845 **(level 2B)**

34. Lomanto D, Iyer SG, Shabbir A, Cheah WK (2006) Laparoscopic versus open ventral hernia mesh repair: a prospective study. Surg Endosc 20**:**1030-1035 **(level 2B)**

35. Levard H, Curt F, Perniceni T, Denet C, Gayet B (2006) [Laparoscopic incisional hernia repair: prospective non randomized trial in 51 cases]. Ann Chir 131**:**244-249 **(level 4)**

36. Antinori A, Moschella F, Maci E, Accetta C, Nunziata J, Magistrelli P (2008) [Immediate and long-term results after laparoscopic primary ventral hernia repair]. Ann Ital Chir 79**:**435-439 **(level 4)**

37. Asencio F, Aguilo J, Peiro S, Carbo J, Ferri R, Caro F, Ahmad M (2009) Open randomized clinical trial of laparoscopic versus open incisional hernia repair. Surg Endosc 23**:**1441-1448 **(level 2B)**

38. Barbaros U, Asoglu O, Seven R, Erbil Y, Dinccag A, Deveci U, Ozarmagan S, Mercan S (2007) The comparison of laparoscopic and open ventral hernia repairs: a prospective randomized study. Hernia 11**:**51-56 **(level 1B)**

39. Bencini L, Sanchez LJ, Scatizzi M, Farsi M, Boffi B, Moretti R (2003) Laparoscopic treatment of ventral hernias: prospective evaluation. Surg Laparosc Endosc Percutan Tech 13**:**16-19 **(level 4)**

40. Olmi S, Cesana G, Sagutti L, Pagano C, Vittoria G, Croce E (2010) Laparoscopic incisional hernia repair with fibrin glue in select patients. JSLS : Journal of the Society of Laparoendoscopic Surgeons / Society of Laparoendoscopic Surgeons 14**:**240-245 **(level 4)**

41. Olmi S, Scaini A, Erba L, Croce E (2007) Use of fibrin glue (Tissucol) in laparoscopic repair of abdominal wall defects: preliminary experience. Surg Endosc 21**:**409-413 **(level 4)**

42. Brill JB, Turner PL (2011) Long-term outcomes with transfascial sutures versus tacks in laparoscopic ventral hernia repair: a review. Am Surg 77**:**458-465 **(level 2A)**

43. Navarra G, Musolino C, De Marco ML, Bartolotta M, Barbera A, Centorrino T (2007) Retromuscular sutured incisional hernia repair: a randomized controlled trial to compare open and laparoscopic approach. Surg Laparosc Endosc Percutan Tech 17**:**86-90 **(level 2B)**

44. Wright D, Paterson C, Scott N, Hair A, O'Dwyer PJ (2002) Five-year follow-up of patients undergoing laparoscopic or open groin hernia repair: a randomized controlled trial. Annals of surgery 235**:**333-337 **(level 1B)**

45. Dennis R, O'Riordan D (2007) Risk factors for chronic pain after inguinal hernia repair. Annals of the Royal College of Surgeons of England 89**:**218-220 **(level 3B)**

46. Courtney C, Duffy K, Serpell M, O'Dwyer P (2002) Outcome of patients with chronic pain following repair of groin hernia. British Journal of Surgery 89**:**1310-1314 **(level 3B)**

47. Poobalan AS, Bruce J, Smith WC, King PM, Krukowski ZH, Chambers WA (2003) A review of chronic pain after inguinal herniorrhaphy. The Clinical journal of pain 19**:**48-54 **(level 1A)**

48. Berndsen F, Petersson U, Arvidsson D, Leijonmarck C-E, Rudberg C, Smedberg S, Montgomery A (2008) Discomfort five years after laparoscopic and Shouldice inguinal hernia repair: a randomised trial with 867 patients. A report from the SMIL study group. Hernia 11**:**307-313 **(level 1B)**

49. Callesen T, Bech K, Kehlet H (1999) Prospective study of chronic pain after groin hernia repair. The British journal of surgery 86**:**1528-1531 **(level 1B)**

50. Poobalan AS, Bruce J, King PM, Chambers WA, Krukowski ZH, Smith WC (2001) Chronic pain and quality of life following open inguinal hernia repair. The British journal of surgery 88**:**1122-1126

**Recurrence after laparoscopic ventral/incisional hernia repair- risk factors,**

**mechanism and prevention**

P. Chowbey

**Method-**The conclusion and recommendation for the risk factors, mechanism and prevention of recurrence following laparoscopic ventral/incisional hernia are based on a systematic search and review of literature performed in Pubmed, Medline, Cochrane library, EMBASE, British journal of Surgery database, UK Pubmed Central, Google, Google scholar, Scirus, Ovid and Directory of Open Journal Access (DOAJ).

Thirty- four publications were found which covered the topic out of which nineteen statements were found useful for this research. In addition it is based on a consensus conference on guidelines for laparoscopic treatment of ventral and incisional hernia held on 13^th^ October 2011 in Suzhou, China.

**Search terms:** risk factors for recurrence, mechanism of recurrence, preventing recurrence of hernia, recurrence after ventral / incisional hernia repair, recurrence rate.

**Statements- Risk factors for Recurrence**

| Level 1 | The existing literature does not show superiority of one mesh fixation technique over the other for recurrence |
| --- | --- |
| Level 3 | Size of the hernia (≥10 cm), BMI (≥30 kg/m2), history of previous open repair or failed hernia repair and perioperative complications like surgical site infection (SSI) are risk factors for hernia recurrence irrespective of the technique |
| Level 3 | The risk factors for recurrence include factors related to patient’s status, underlying disease and perioperative factors which include surgical techniques, postoperative complications, deep abscesses, and early reoperations |
| Level 3 | Smokers with earlier failed repair attempts have a higher risk of recurrence |
| Level 3 | Postoperative mesh infection requiring removal of mesh is a predictor of recurrence. |
| Level 3 | A higher incidences of seroma formation and recurrence is seen in cases treated with dual mesh |
| Level 3 | Repetition of a previously inadequate technique in recurrent hernia frequently fails. |

**Recommendations**

| Grade B | Risk factors predisposing to recurrence after laproscopic ventral or incisional hernia repair should be eliminated before operation as far as possible. |
| --- | --- |
| Grade B | Insufficient incision scar coverage with mesh, SSI and gastrointestinal complications should be avoided. |

**Statements- Mechanisms of Recurrence**

| Level 3 | Mechanism of recurrence of ventral hernia described in the literature in decreasing order of frequency are infection, lateral detachment of mesh, inadequate mesh fixation, inadequate mesh, inadequate overlap, missed hernias, increased intraabdominal pressure and trauma. |
| --- | --- |
| Level 4 | The mechanism of recurrence can be improperly placed transfascial sutures, overly large bites of mesh causing excessive tension and ultimately a hole in the mesh. |
| Level 4 | Mesh shift may be a precursor to hernia recurrence. Mesh tends to shift away from the operative side leading to recurrence. Recurrence may be a two-step process, beginning first with intra-operative mesh shift followed by additional factors (such as mesh contraction) that may accentuate the shift and lead to recurrence. |
| Level 4 | Recurrence can occur at defects at transfascial suture sites of previous laparoscopic ventral hernia mesh repair. |

**Recommendations**

| Grade B | A strictly standardized technique to avoid failures like mesh overlap less than 3 cm, improper fixation, mesh contraction and invagination into the hernial defect should be used. |
| --- | --- |
| Grade C | An optimal preoperative treatment in patients with an increased intraabdominal pressure in conditions like COPD, chronic cough and obesity should be considered. |

**Statements- Prevention of Recurrence**

| Level 1 | Recurrences can be prevented by using increased overlap of the biomaterial and placing dual methods of fixation (tacks and transfascial sutures). |
| --- | --- |
| Level 3 | Incisional hernias and ventral hernias larger than 2 cm are preferably repaired using prosthesis because primary repair has a high rate of recurrence. |
| Level 3 | Use of mesh in a repair of incisional hernia reduces the risk of recurrence. |
| Level 3 | A mesh overlap of at least 5 cm and fixation of the lower margin of the mesh under direct vision to Cooper's ligaments appears to confer increased strength and durability and contribute to low hernia recurrence rates in patients with suprapubic hernias. |
| Level 4 | Meticulous use of transfascial sutures with other fixation methods improves recurrence rates in high -risk obese patients. |
| Level 4 | Insufficient coverage of the incision scar is a risk factor for recurrence after laparoscopic repair of ventral and incisional hernia hence the entire incision and not just the hernia must covered with mesh. |
| Level 5 | Some surgeons believe that suture fixation of mesh is mandatory in laparoscopic ventral hernia repair to avoid higher recurrence rate |
| Level 5 | Some surgeons believe that total intraperitoneal fixation with tackers reduced the surgical time, avoided parietal vascular injuries and postoperative pain, and maintained a similar recurrence. |

**Recommendations**

| Grade B | A mesh repair should be used for all eligible patients with hernial defect size more than 2 cm. |
| --- | --- |
| Grade B | In suprapubic hernias the whole preperitoneal space should be dissected, a mesh overlap of at least 5 cm should be achieved, and fixation of the lower margin of the mesh under direct vision to Cooper’s ligaments should be done. |
| Grade B | Sufficient overlap of the mesh from the hernial margin and dual methods of fixation should be used. |

**Discussion**

Various factors can lead to recurrence of hernia after ventral or incisional hernia repair. According to some studies some patients are more susceptible to recurrence due to inherently weak native tissue and proven defect of collagen synthesis. [1, 2] Recurrence rate increases with the size of the primary hernial defect, larger the size (more than 10 cm) higher is the chance of recurrence. Patients with underlying disorders like obesity, chronic obstructive pulmonary disorder (COPD), chronic cough or diabetes mellitus are more predisposed to recurrence. [3, 4] Smokers with earlier failed repair attempts or [5] patients with a history of previous failed repair also contribute to recurrence rate. [6]

Conventional suture hernia repair has a high recurrence rate of 54-63% which reduces to 32% with the use of mesh. [7, 8]. Insufficient coverage of the incision scar is also a risk factor for recurrence after laparoscopic repair of ventral and incisional hernia. [9] Dual mesh reportedly increases the risk of recurrence. [10]

Post operative factors contributing to the recurrence after ventral or incisional hernia repair include surgical site infection (SSI), mesh infection, wound infection, deep abscesses, and gastrointestinal complications. [11]

According to a retrospective review of 1,242 patients, most common causes for the recurrence was mesh overlap less than 3 cm, displacement of the mesh, mesh contraction and invagination into the hernial defect. [12] Improperly placed transfascial sutures overlying large bites of mesh cause excessive tension and ultimately a hole in the mesh which results in recurrence. [13] Mesh shift may also be a precursor to hernia recurrence, beginning first with intra-operative mesh shift and then followed by additional accentuating factors like mesh contraction.[14] Most surgeons report using both transfascial sutures and laparoscopically placed tacks to secure prostheses in laparoscopic ventral hernia repair but no significant difference is found in rates of hernia recurrence.[15]

Increased intra-abdominal pressure also predisposes to recurrence. Therefore patients with conditions like morbid obesity, COPD, chronic cough have high risk for recurrence. [3, 4]

Incidence of recurrence after repair can be minimized by taking precautions in patients who are at high risk for recurrence. Patients with conditions like COPD, chronic cough should be treated preoperatively and in morbidly obese patients, larger mesh should be used. As mesh repair decreases the incidence of recurrence to half, it should be used for all eligible patients with hernial defect size more than 2 cm. [7, 8, 16] Laparoscopic approaches should be considered over open repair as it decreases the recurrence rate further. Recurrences can also be prevented by using increased overlap of the biomaterial and placing dual methods of fixation.[17] In cases of Suprapubic hernias mesh overlap of at least 5 cm and fixation of the lower margin of the mesh under direct vision to Cooper’s ligaments confers increased strength and durability and contributes to low hernia recurrence rates.[18] In addition the whole incision and not just the hernia must be repaired to lower the chances of recurrence. [9]

In conclusion, proper technique and addressing the patients’ underlying risk factors can significantly reduce hernia recurrence.

**References**

1. Chowbey PK, Sharma A, Mehrotra M, Khullar R, Soni V, Baijal M. Laparoscopic repair of ventral / incisional hernias. J Min Access Surg 2006;2:192-8 **(level 3)**
2. Klinge U, Conze J, Krones CJ, Schumpelik V. Incisional hernia: Open techniques. World J Surg 2005;29:1066-72**.(level 5)**
3. Kurmann A, Visth E, Candinas D, Beldi G. Long-term follow-up of open and laparoscopic repair of large incisional hernias. World J Surg. 2011 Feb;35(2):297-301.**(level 33)**
4. Heniford BT, Park A, Ramshaw BJ, Voeller G. Laparoscopic repair of ventral hernias: nine years' experience with 850 consecutive hernias. Ann Surg. 2003 Sep;238(3):391-9; discussion 399-400**.(level 3)**
5. Bencini L, Sanchez LJ, Bernini M, Miranda E, Farsi M, Boffi B, Moretti R. Predictors of recurrence after laparoscopic ventral hernia repair. Surg Laparosc Endosc Percutan Tech. 2009 Apr;19(2):128-32**.(level 3)**
6. Rosen M, Brody F, Ponsky J, Walsh RM, Rosenblatt S, Duperier F, Fanning A, Siperstein A. Recurrence after laparoscopic ventral hernia repair. Surg Endosc. 2003 Jan;17(1):123-8. Epub 2002 Sep 23**. (level 3)**
7. Paul A, Korenkov M, Peters S, Kohler L, Fischer S, Troidl H. Unacceptable results of the Mayo procedure for repair of abdominal incisional hernias. Eur J Surg. 1998;164:361-367. **(level 3)**
8. Burger JW, Luijendijk RW, Hop WC, Halm JA, Verdaasdonk EG, Jeekel J. Long-term follow-up of a randomized controlled trial of suture versus mesh repair of incisional hernia. Ann Surg.2004; 240:578-583**.(level 1B)**
9. Wassenaar EB, Schoenmaeckers EJ, Raymakers JT, Rakic S. Recurrences after laparoscopic repair of ventral and incisional hernia: lessons learned from 505 repairs. Surg Endosc. 2009 Apr;23(4):825-32. Epub 2008 Sep 24**.(level 4)**
10. Biondi A, Tropea A, Monaco G, Musmeci N, Zanghi G, Basile F. Complications in the laparoscopic treatment of primary and secondary hernias of the abdominal wall. Ann Ital Chir. 2010 May-Jun;81(3):193-8**. (level 3)**
11. P K Chowbey et al, Max institute of MAMBS, India SYMPOSIUM. 2006;l2(3):192-98 **(level 3)**
12. Sharma A, Mehrotra M, Khullar R, Soni V, Baijal M, Chowbey P. Laparoscopic ventral/incisional hernia repair: a single centre experience of 1,242 patients over a period of 13 years. Hernia [Internet]. 2011 Apr 1;15(2):131–9 **(level 3)**
13. Barzana D, Johnson K, Clancy TV, Hope WW. Hernia recurrence through a composite mesh secondary to transfascial suture holes. Hernia. 2012 Apr;16(2):219-21. Epub 2010 Sep 12.**(level 4)**
14. Liang MK, Clapp ML, Garcia A, Subramanian A, Awad SS. Mesh shift following laparoscopic ventral hernia repair. J Surg Res. 2012 Apr 12.**(4)**
15. Brill JB, Turner PL. Long-term outcomes with transfascial sutures versus tacks in laparoscopic ventral hernia repair: a review. Am Surg. 2011 Apr;77(4):458-65. **(level 4)**
16. Muysoms F, Daeter E, Mijnsbrugge GV, Claeys D. Laparoscopic intraperitoneal repair of incisional and ventral hernias. Acta Chirurgica Belgica. 2004; 104(6): 705-708 **(level 4)**
17. LeBlanc KA, Whitaker JM, Bellanger DE, Rhynes VK. Laparoscopic incisional and ventral hernioplasty : lessons learned from 200 patients. Hernia. 2003 Sep;7(3):118-24. Epub 2003 Mar 21**.(level 3)**
18. Sharma A, Dey A, Khullar R, Soni V, Baijal M, Chowbey PK. Laparoscopic repair of suprapubic hernias: transabdominal partial extraperitoneal (TAPE) technique. Surg Endosc. 2011 Jul;25(7):2147-52. Epub 2010 Dec 24**.(level 3)**

**Section 6: Technique – special questions**

**Is laparoscopic preperitoneal ventral and incisional hernia repair possible?**

W. Reinpold

The conclusions and recommendations on laparoscopic preperitoneal ventral and incisional hernia repair are based on a systematic review of the literature and a consensus conference on guidelines for the laparoscopic treatment of ventral and incisional hernias held in October 2011 Suzhou, China during the 5^th^ meeting of the International Endohernia Society (IEHS).

Pubmed, Medline, Embase, Br J Surg Database, Science Citation Index and the Cochrane database were searched for studies on laparoscopic preperitoneal ventral and incisional hernia repair. Search terms were “endoscopic preperitoneal repair” or “laparoscopic preperitoneal repair” or “endoscopic sublay repair” or “laparoscopic sublay repair” and “ventral hernia” or “incisional hernia” or “abdominal wall hernia” or “umbilical hernia”. Additionally experts in the field of abdominal wall hernia repair were contacted. The levels of evidence and grades of recommendation are based on the Oxford evidence-based medicine criteria (see above).

**Introduction:**

Today laparoscopic IPOM repair [1] and open sublay repair first described by Rives [2] are the most frequently used techniques for the cure of primary and incisional abdominal wall hernias. The advantages of minimal access surgery are evident. In the literature laparoscopic IPOM repair is associated with less infections and wound healing complications compared to open mesh repairs [1]. In contrast to all other laparoscopic procedures acute and chronic pain does not seem to be reduced after laparoscopic IPOM operations. The IPOM-technique is performed with expensive compound meshes whose bowel -facing surface is covered with adhesion preventing material. IPOM meshes have to be fixated thoroughly with transmural sutures, staples or clips which carry the risk of adhesions and acute and chronic postoperative pain. The long -term safety of IPOM meshes has not been proven in human clinical studies.

Other disadvantages of the laparoscopic IPOM repair are: 1. In most of the cases the hernia sac stays in situ, the defect is bridged, and the abdominal wall is not reconstructed. 2. All adhesions between the viscera and abdominal wall have to be taken down. 3. There seem to be more severe complications such as bowel lesions.

For a further improvement of abdominal wall hernia repair the advantages of the sublay repair and laparoscopic IPOM repair should be combined. Can a preperitoneal ventral and incisional hernia repair be achieved with reduced access trauma?

# Statements

| Level 4/5 | Laparoscopic transperitoneal and total extraperitoneal preperitoneal/ sublay repair are surgical options for the treatment of small and medium size ventral and incisional hernias (EHS Classification W1 and W2).  Both techniques allow the implantation of large standard alloplastic protheses.  The procedures are technically demanding with longer operating times than open preperitoneal/ sublay repair and laparoscopic IPOM repair but do not require compound meshes.  Laparoscopic preperitoneal repair combines the advantages of open preperitoneal repair and laparoscopic IPOM technique: small incisions and extraperitoneal mesh position.  Complication rates are low. |
| --- | --- |

### Recommendations

| Grade C | Laparoscopic transperitoneal and total extraperitoneal preperitoneal/ sublay repair may be considered for the cure of small and medium size ventral and incisional hernias (EHS classification W1 and W2) if expertise is present. |
| --- | --- |
| Grade D | Especially in the lower abdomen laparoscopic transperitoneal or extraperitoneal preperitoneal abdominal wall hernia repair can be considered if expertise is present. |

**Laparoscopic preperitoneal abdominal wall hernia repair**

There are only few literature reports on laparoscopic preperitoneal abdominal wall hernia repair [3-12]. As in inguinal hernia repair (TAPP and TEP) the laparoscopic preperitoneal mesh repair of ventral and incisional hernias can be performed via a transperitoneal or total extraperitoneal approach. The mesh may be separated from the abdominal cavity by the peritoneum only, the posterior rectus sheath and peritoneum or the urinary bladder.

**Laparoscopic transperitoneal preperitoneal mesh repair of ventral and incisional hernias (TAPP)**

In the literature there are 47 cases of laparoscopic transperitoneal preperitoneal abdominal wall hernia repair mainly in the lower abdomen reported [3-9]. Small and medium size suprapubic, umbilical, lumbar, epigastric and port site hernias have been operated on with a laparoscopic transperitoneal preperitoneal mesh repair. In the lower abdomen a modified TAPP technique can be used especially for the treatment of Spigelian hernias [4,6].

Since 2003 the author´s working group has performed 142 TAPP operations of primary and incisional epigastric, umbilical, combined umbilical and epigastric, lateral abdominal wall, Spigelian, and port-site hernias with the implantation of standard polypropylene meshes.

In a prospective cohort trial with a control group Schröder et al. [under review] report about a three port laparoscopic transperitoneal sublay repair (LTSR) technique via the left flank. In 43 small and medium size ventral and incisional hernias medium size and large pieces of standard polypropylene meshes (15 x 15 cm up to 30 x 20 cm) were implanted. The follow-up was 92% with a median of 16 months. Compared to the open sublay repair group there was less acute pain and the hospital stay was shorter. However, operating time was longer in the laparoscopic group. There were no differences in chronic pain and discomfort. In both groups were neither recurrence nor wound infection. The authors conclude that LTSR is a safe and effective method for the treatment of small and medium size primary and incisional abdominal wall hernias combining the advantages of open sublay and laparoscopic IPOM repair.

**Endoscopic total extraperitoneal preperitoneal abdominal wall hernia repair (TEP)**

Three publications with 17 cases of endoscopic total extraperitoneal mesh repair of abdominal wall hernias (abdominal wall TEP) were found [10-12]. Miserez et al. published 15 cases of abdominal wall TEP of the rectus compartment in 2002 [10]. There are two case reports about TEP Spieghelian hernia repair.

Reinpold et al. [in preparation; oral presentation EHS congress Istanbul 2010] developed a transhernial single port TEP technique for the treatment of primary and incisional abdominal wall hernias. Via a 3 to 4cm incision the hernia sac and midline defect are dissected. The extraperitoneal space around the defect is enlarged by separation of the peritoneum from the fascia. Large hernia sacs are removed and defects of the peritoneum are closed. A single port with three 5-mm trocars is inserted into the defect. With Capnopreperitoneum of 10mmHG the circumference of the defect is dissected endoscopically. A standard polypropylene mesh is inserted in the sublay position and fixated with sutures or clips at the lateral border. Alternatively a self- fixating mesh can be used. The midline defect is closed via the port incision. Twenty-four patients with an average defect size of 17cm² (9 - 61cm²) were operated on. The average mesh size was 232cm² (96cm² - 600cm²). Pain medication was stopped in all patients after a maximum of 4 days. Two small retromuscular hematomas were treated conservatively. After an average follow-up of 8 months (2 – 15 months) there was no chronic pain, no recurrence and no infection.

**Conclusion:**

Laparoscopic preperitoneal abdominal wall hernia repair in the TAPP and TEP technique in small and medium size primary and incisional abdominal wall hernia is feasible with a minimum morbidity. The advantages are: 1. Minimal access trauma 2. Standard meshes without thorough fixation can be used. 3. The Abdominal cavity is only minimally compromised. 4. The hernia sac is removed from the abdominal wall. 5. The hernia defect is closed and the abdominal wall is reconstructed anatomically. However, the technique is demanding and the operations take longer than standard procedures

**References**

1. Sajid MS, Bokhari SA, Mallick AS, Cheek E, Baig MK (2009) [Laparoscopic versus open repair of incisional/ventral hernia: a meta-analysis.](http://www.ncbi.nlm.nih.gov/pubmed/18614144) Am J Surg 197(1):64-72. **(level 1A)**

2. Rives J, Pire JC, Flament JB, Convers G (1977) [Treatment of large eventrations (apropos of 133 cases).](http://www.ncbi.nlm.nih.gov/pubmed/329161) Minerva Chir. 32(11):749-56. **(level 2C)**

# 3. Gagner M, Milone L, Gumbs A, Turner P (2010) Laparoscopic repair of left lumbar hernia after laparoscopic left nephrectomy. JSLS 14(3):405-9. (level 4)

# 4. Majeski J (2009) Open and laparoscopic repair of Spieghelian hernia. Int Surg 94(4):365-9. (level 4)

# 5. Hilling DE, Koppert LB, Keijzer R, Stassen LP, Oei IH (2009) Laparoscopic correction of umbilical hernias using a transabdominal preperitoneal approach: results of a pilot study

Surg Endosc 23(8):1740-4. **(level 4)**

# 6. Palanivelu C, Vijaykumar M, Jani KV, Rajan PS, Maheshkumaar GS, Rajapandian S (2006) Laparoscopic transabdominal preperitoneal repair of Spieghelian hernia.

JSLS 10(2):193-8. **(level 4)**

7. McKay R, Haupt D (2006) Laparoscopic repair of low abdominal wall hernias by tack fixation to the cooper ligament. Surg Laparosc Endosc Percutan Tech 16(2):86-90. **(level 4)**

8. Bhandarkar DS, Katara AN, Shah RS, Udwadia TE (2005) Transabdominal preperitoneal repair of a port-site incisional hernia. J Laparoendosc Adv Surg Tech A 15(1):60-2. **(level 4)**

# 9. Shekkariz B, Graziottin TM, Gholami S, Lu HF, Yamada H, Duh QY, Stoller ML (2001) Transperitoneal preperitoneal laparoscopic lumbar incisional herniorrhaphy. J Urol 166(4):1267-9. (level 4)

10. Miserez M, Penninckx F (2002) Endoscopic totally preperitoneal ventral hernia repair. Surg Endosc 16(8):1207-13. **(level 4)**

# 11. Koksal N, Altinli E, Celik A, Oner I (2004) Extraperitoneal laparoscopic approach to Spieghelian hernia combined with groin hernias. Surg Laparosc Endosc Percutan Tech 14(4):204-6. (level 4)

# 12. Tarnoff M, Rosen M, Brody F (2002) Planned totally extraperitoneal laparoscopic Spieghelian hernia repair. Surg Endosc 16(2):359. (level 4)

**The role of Endoscopic Component Separation (ECS) in the treatment of large**

**abdominal wall hernias**

W. Reinpold

The conclusions and recommendations on endoscopic component separation (ECS) are based on a systematic review of the literature and a consensus conference on guidelines for the laparoscopic treatment of ventral and incisional hernias held in October 2011 Suzhou, China during the 5^th^ meeting of the International Endohernia Society (IEHS).

Pubmed, Medline, Embase, Br J Surg Database, Science Citation Index and the Cochrane database were searched for studies on endoscopic component separation for the treatment of very large abdominal wall hernias. Search terms were “endoscopic component separation” or “laparoscopic component separation” and “ventral hernia” or “incisional hernia” or “abdominal wall hernia”. Additionally experts in the field of abdominal wall hernia repair were contacted. The levels of evidence and grades of recommendation are based on the Oxford evidence-based medicine criteria (see above).

Seventeen publications with 128 cases of ECS were identified.

**Introduction:**

Very large incisional hernias with a horizontal defect of more than 10 cm are a challenge in abdominal wall hernia surgery. In many of these giant incisional hernias standard open techniques and the laparoscopic IPOM repair are insufficient. The defect closure with reconstruction of the linea alba can often only be achieved with the open component separation (OCS) published by Oscar Ramirez in 1990 [1]. The open component separation gives an abdominal wall release of 10 to 15cm on every side but implies a very extended dissection of subcutaneous tissue of the abdominal wall with destruction of the deep perforating vessels. This leads to a high rate of wound infections and wound healing problems [2-6].

**Statements**

| Level 3 | The endoscopic component separation (ECS) is feasible with low morbidity.  The ECS can be combined with lap IPOM, open IPOM, open sublay and open onlay technique in complex hernias.  Abdominal wall release after ECS is less extensive than after OCS  There are less wound infections and wound healing problems after ECS compared to open component separation. |
| --- | --- |
| Level 4 | The question whether the lateral compartment should be augmented with mesh is unresolved. |

### Recommendations

| Grade C | In large and very large ventral and incisional hernias the endoscopic component separation can be considered in combination with open or laparoscopic mesh techniques if expertise is present. |
| --- | --- |

The ECS can be combined with other open or laparoscopic procedures [3-8]. Losanoff et al. were the first to report on endoscopic assisted component separation in 2002 [9]. In 2007 Rosen et al. [2] published a retrospective study of seven patients who underwent an ECS for abdominal wall reconstruction during the resection of an infected prosthetic material in complex abdominal wall hernias. The technique of ECST as described by Rosen et al. [2]:
Below the costal margin and lateral of the rectus compartment bilateral 15mm skin incision and insertion of a 10mm balloon dilator. Blunt dissection of the avascular space between the external and internal oblique muscle. Insertion of two trocars, insufflation of CO2 and further dissection of the space under camera vision. The fascia of the external oblique muscle is vertically incised lateral of the rectus compartment from the costal margen to the inguinal area. Residual defect size following the removal of all prosthetics was 338 cm2 (range 187-450). ECS enabled tension-free primary fascial reapproximation in all patients. There was one superficial surgical site infection. After an average follow-up period of 4.5 months, no recurrences were identified.

Harth et al [3,4] reported a retrospective study on 32 ECS compared to 22 open component separations (OCS). Open component separation had a 41% major wound morbidity rate compared with 19% in the endoscopic group (p = 0.07). Hernia recurrences rates were similar (open, 32%; endoscopic, 27%; p=0.99). Hospital length of stay was 11 days after OST vs 8 days after ECST (P=0.09). The median mesh costs differed significantly between ECS and OCS ($733 vs. $8,415; p = 0.05). The authors concluded that there were significantly less wound complications after ECS, and similar high rates of recurrence.

These findings were confirmed by the publications of Albright et al, Giurgius et al., Bachman et al, Parker et al. [5-8].

The ECS can be combined with laparoscopic IPOM, open IPOM, open sublay and open onlay technique in complex hernias. The abdominal wall release after ECS is less extensive than after open CST [4-7]. There are less wound infections and wound healing problems after ECS compared to open CS [3-8]. The question whether the lateral compartment should be augmented with mesh is unresolved. No long term data available. Further studies for the assessment of the ECS are necessary.

**References:**

1. Ramirez O, Ruas E, Dellon A (1990) Components Separation Method for closure of abdominal wall defects: an anatomic and clinical study. Plast Recontsr Surg 86:526. **(level 3)**

2. Rosen MJ, Jin J, McGee MF, Williams C, Marks J, Ponsky JL (2007) Laparoscopic component separation in the single-stage treatment of infected abdominal wall prosthetic removal. Hernia, 11(5):435-40**.(level 3)**

3. Harth KC, Rosen MJ (2010) Endoscopic versus open component separation in complex abdominal wall reconstruction. Am J Surg 199(3):342-6**.(level 3)**

4. Harth KC, Rose J, Delaney CP, Blatnik JA, Halaweish I, Rosen MJ (2011) Open versus endoscopic component separation: a cost comparison. Surg Endosc 25(9):2865-70**.(level 3)**

5. Albright E, Diaz D, Davenport D, Roth JS (2011) The component separation technique for hernia repair: a comparison of open and endoscopic techniques. Am Surg 77(7):839-43**.(level 3)**

6. Giurgius M, Bendure L, Davenport DL, Roth JS. (2012) The endoscopic component separation technique for hernia repair results in reduced morbidity compared to the open component separation technique. Hernia 16(1):47-51**.(level 3)**

7. Bachman SL, Ramaswamy A, Ramshaw BJ. (2009) Early results of midline hernia repair using a minimally invasive component separation technique. Am Surg 75(7):572-7**.(level 3)**

8. Parker M, Bray JM, Pfluke JM, Asbun HJ, Smith CD, Bowers SP (2011)
Preliminary experience and development of an algorithm for the optimal use of the laparoscopic component separation technique for myofascial advancement during ventral incisional hernia repair. J Laparoendosc Adv Surg Tech A 21(5):405-10**.(level 4)**

9. Losanoff JE, Richman BW, Jones JW (2002) Endoscopically assisted "component separation" method for abdominal wall reconstruction. J Am Coll Surg 195(2):288-291.(level 4)

**LAPAROSCOPIC PARASTOMAL HERNIA REPAIR**

Salvador Morales-Conde

A Medline search was performed until November 2011, using the following terms: lparoscopic, laparoscopy, paracolostomy, colostomy, paracolostomal, colostomal, paraileostomy, ileostomy, ileal conduit, urostomy, hernia, defect, repair, closure and reconstruction.

The numbers of papers identified were 73 (following the flow indicated in figure 1). The number of papers analyzed were 27, being excluded 46 for the following reasons (Figure 2): 17 were studies not related to hernia surgery, 7 were series of ventral hernia where parastomal hernias were not included, 2 where parastomal hernias are just mentioned as a case part of a series of ventral hernias treated by laparoscopy, 4 are series part of a larger series published later by the same author, 1 is a series published two time by the same author and 12 are clinical cases with just one case.

**INTRODUCTION**

Parastomal hernias are the most frequent complication that occurs after a stoma formation surgery. It can be described as an incisional hernia developed in the proximity of a stoma (ileostomy, colostomy, ureterostomy etc.).It´s incidence is not easy to be established, being an underestimated problem for the patients and even for the physicians. The incidence rate have been reported ranging from 2,8% up to 50% (1), being directly related to the time of follow up.Loop ileostomy has the lowest risk (0%-6.2%),followed by end ileostomy, and loop colostomy with a similar risk of 28% to 30%. End colostomy carries the highest risk for parastomal hernia of more than 50%. Eventhough most hernias occur within the first 2 years after stoma construction, the risk ofherniation extends up to 20 years (2).

Many risk factors have been related to the development of a parastomal hernia, being only considered the waist circumference, the age and the size of the stoma as independent risk factors for the presence of a parastomal hernia after a permanent colostomy (2,3).Parastomal hernia is asymptomatic most of the time, but it may be associated with serious complications such as strangulation and perforation;hence, elective repair is mandatory for carefully selected cases and surgical approaches.The diagnosis is performed by clinical examination,being CT-scan very useful in order to determine the content of the hernia sac, the size of the defect and the presence of a concomitant hernia at the midline incision.

Many different techniques have been described for the treatment of parastomal hernias.Non-mesh techniques are related to a high rate of recurrence (46-100%), and therefore should not be performed (4,5), offering mesh techniques significant better results. Meshes could be placed onlay or sublay through a local incision, close to the stoma, although these techniques are related to a high incidence of wound infection, up to 30%.(6,7). The underlay or IPOM (intraperitoneal onlay mesh) position have offered better results in terms of wound infections, and brings us the opportunity to repair a concomitant incisional hernia if present. Laparoscopic approach tries to join the advantages of a minimally invasive approach together with a low incidence of infection and recurrence rate that offers the intraabdominal placement of a mesh.

Out of the 27 papers included in the final analysis, there were no papers with level of evidence 1, 2 or 3a, only 3papers with level of evidence 3b (9-11), 16 with level of evidence 4 (12-27), and 8 with level of evidence 5 (28-35).Out of the three of the studies with level 3b of evidence, one of them compares two of the different techniques used to perform the repair of parastomal hernias by laparoscopy (11), while the other two compare the open approach versus the laparoscopic techniques (9,10). But if we analyze the two studies, we can find that one of them has a very poor quality since the authors compare the laparoscopic approach with a wide variety of open techniques, including no-mesh and mesh techniques (10). On the other hand, 8 of the studies of level 4 evidence are just series of cases with less than 10 cases included.

***II.-IS LAPAROSCOPIC APROACH OF PARASTOMAL HERNIAS SUPERIOR TO OPEN APPROACH?***

***II.a.-Statements***

| ***Level 3*** | -Laparoscopic repair of parastomal hernia can be performed safely |
| --- | --- |
| ***Level 4*** | -The rate of recurrences after laparoscopic repair of parastomal hernias are lower than after open approach |

***II.b.-Recommendations***

| ***Grade B*** | -Laparoscopic repair of parastomal hernia SHOULD BE considered a safe alternative to open approach |
| --- | --- |
| ***Grade C*** | -Laparoscopic parastomal hernia repair COULD be considered a valid option to open repair since the rate of recurrences of this approach SEEMS to be lower than open approach |

***II.3.-Discussion***

Open suture repair of the fascial defect or stoma resiting are both associated with high morbidity and unacceptably high recurrence rates and are no longer recommended for routine use.Primary closure of the aponeurosis at the hernia site, either via peristomal approach or through midline incision, is a simple procedure, but it carries a recurrence rate of 38% to 100%. Stoma relocation may result in a zero recurrence rate at the same hernia site, but the risk of a parastomal hernia after new stoma formation is as high as 46%. In addition, an incisional hernia at the previous colostomy site closure may also occur. For this reason, the use of polypropylene meshes has been applied to this repair, either to reinforce suture repair or to bridge the fascial gap. The recurrence rate with this open technique will still incur a failure rate of 20-33% (2).Additionally,complications related to polypropylene meshes have been described, such as obstruction, fistulization or mesh erosion with this biomaterial (36).Meshes can be placed in different anatomic positions: during the onlay repair, the mesh is subcutaneously placed and fixed to the fascia of anterior rectus muscles and to the aponeurosis of the external oblique abdominal muscle; a retromuscular technique indicates that the prosthesis is placed dorsally to the rectus muscle and anteriorly to the posterior rectus sheath; with an intraperitoneal position, the mesh is placed intra-abdominally being fixed to the peritoneum. Basically, two techniques are used to repair parastomal hernias with an intraperitoneally positioning of prosthesis: the ‘Sugarbaker’ technique and the keyhole technique. In 1985, Sugarbaker (37) described a new technique for parastomal hernia repair through a midline laparotomy; the bowel was lateralized passing from the hernia sac between the abdominal wall and the prosthesis, which was sutured to the fascial edge covering the opening.

The laparoscopic approach involves minimally invasive access to the abdominal cavity and intraperitoneal placement of prosthetic material with or without narrowing the defect. Similarly to the open intraperitoneal mesh repair, the Sugarbaker, the keyhole and a combination of both, technique described by D Berger et al (20) and known as the sandwich-technique, are used. laparoscopic approach makes peristomal incision unnecessary and decreases the potential risk of mesh infection as well.Published series on laparoscopic mesh repair of parastomal hernia, however, are few with relative short follow-up.

There are two studies with level 3b evidence which compare the open approach with the laparoscopic techniques to repair parastomal hernias. Both papers are retrospective studies, but the one conducted by McLemore et al (10) includes in the open group cases in which a suture repair was performed together with mesh techniques and relocation of the stoma, being the recurrence and the morbidity rates of this techniques very variable. On the other hand, this author also includes in the laparoscopic group cases performed following the keyhole and the modified-Sugarbaker technique and, as we will see later on the paper, both techniques are also associated to a different recurrence rate.

The most important message coming from the other study with level 3b of evidence, conducted by Pastor et al (9), is that the morbidity rate of the laparoscopic approach was 15%, while the complications after the open approach reach up to 33% of the cases.Regarding recurrences, even that this last study, by Pastor et al (9), shows a lower recurrence rate after the laparoscopic approach than after open techniques (33% vs 53,8%), the follow-up is different (13,9 month vs 21,4 month), it is has been mentioned before that this rate could increase with time.

In order to draw conclusions regarding recurrence, we have to analyze the studies with level 4 of evidence. Even there are some cases series with high recurrence rates, up to 56% (24), most of the studies report a recurrence rate below 10%, which represent an overall better recurrence rate than the results coming from the open approach.At present none of the methods of open or laparoscopic mesh repair has proved superior. In spite of this laparoscopic repair has gained increasing acceptance.

***III.- HAVE LAPAROSCOPIC PARASTOMAL HERNIA REPAIR SIMILAR RESULTS COMPARED TOLAPAROSCOPIC VENTRAL HERNIA REPAIR?***

***III.a.- Statements***

| ***Level 4*** | - Surgical time during parastomal hernia repair is longer than after laparoscopic ventral hernia repair, since the technique is more difficult, especially due to a more difficult process of adhesiolysis.  - Intraoperative complications during laparoscopic repair of parastomal hernias are more frequent than during standard laparoscopic ventral hernia repair  - A high percentage of parastomal hernia present an association of a midline incisional hernia what make the surgical procedure more complex and more demanding for the surgeon.  - The rate of recurrences and morbidity are higher after laparoscopic parastomal hernia repair than after laparoscopic ventral hernia repair. |
| --- | --- |

***III.b.- Recommendations***

| ***Grade C*** | - Laparoscopic approach of parastomal hernias SHOULD be considered a difficult technique with longer operating time, more intraoperative complications and more difficult adhesiolysisthan standard laparoscopic ventral hernia repair  -Results from laparoscopic repair of parastomal hernias COULD NOT be compared with the general results of lap ventral hernia repair, since the rate of recurrence and morbidity are higher  - Laparoscopic approach of parastomal hernias COULD be considered a more complex technique since a concomitant midline hernia must be repaired in a high percentage of cases |
| --- | --- |

***III.3.-Discussion***

If we compare the results after laparoscopic repair of parastomal hernias with the data published with level 1a of evidence on laparoscopic ventral hernia repair, the one from Sauerland et al published in 2011 (38), and the one from Kapischke et al in 2008 (39), we can observe that the surgical time associated to the repair of parastomal hernia by laparoscopy is longer and the morbidity higher. This data show that this technique seems to be more difficult than standard laparoscopic ventral hernia repair due to the presence of stronger adhesions and concomitant incisional hernias in the midline. The other reasonis related to the difficulties to free the adhesions of the bowel from the loop that goes to the ostomy, which need more surgical skills to avoid damaging this loop. On the other hand, even that the rate of infection during laparoscopic ventral hernia repair is close to zero, we can observe that this rate, or other late-mesh related complications, increases during laparoscopic parastomal hernia repair. In fact, series, like the one from Wara et al (15) and Craft et al (19), show a rate of infection of 7% and 9,5%, respectively. The conclusion of thiscomparison is that laparoscopic parastomal hernia repair seems to be a more complex surgery than standard laparoscopic ventral hernia repair. This fact leads us to think that this procedure needs to be performed by an expert surgeon in this field.

On the other hand, a comparison between the data observed in different studies with different level of evidence coming from laparoscopic ventral hernia repair, the overall recurrences rate of this technique is lower than the recurrence rate observed after laparoscopic repair of parastomal hernias. These facts are also related to the complexity of the surgery, together with the fact that the approach to this type of hernias has started to be published almost 10 years later after the first paper published on laparoscopic ventral hernia repair, what means that surgeons are still defining the best way to perform this technique.In fact, there are still doubts if the results of the laparoscopic modified-Sugarbaker technique would be better than the one offer by the other two techniques described in the literature, the Key-hole and the Sandwich technique. But, following the first anecdotalreports there are accumulating evidence that laparoscopic mesh repair is feasibleand has a promising potential in the management of parastomal hernia.

***IV.-WHICH LAPAROSCOPIC TECHNIQUE FOR PARASTOMAL HERNIAS IS SUPERIOR TO ANOTHER?***

***IV.a.- Statements***

| ***Level 3b*** | -Laparoscopic repair of parastomal hernia using a pure e-PTFE mesh (in modified Sugarbaker technique) is associated with better results than if the key-hole technique is performed |
| --- | --- |
| ***Level 4*** | -Using the key-hole technique after implantation of a pure e-PTFE mesh in the long term the recurrence rate is very high  -The results achieved after implantation of a e-PTFE-polypropylene mesh using the key-hole technique are better compared to pure e-PTFE |

***IV.b.- Recommendations***

| ***Grade B*** | - Laparoscopic repair of parastomal hernia using the modified-Sugarbaker technique SHOULD BE considered the one to be recommended WHEN a pure e-PTFE mesh is used |
| --- | --- |
| ***Grade C*** | - Although there is only one serie with the Sandwich technique, using two meshes, this technique COULD be considered a safe alternative to keyhole or Sugarbaker technique.  - The same laparoscopic technique COULD be performed for a hernia after a colostomy, ileostomy, urostomy or due to a ileal conduit |

***IV.3.-Discussion***

Laparoscopic parastomal hernia repair has become a viable option to overcome the challenges that face the hernia surgeon.Most series suffer from small sample size and controlled trials are lacking. The limited data, therefore, make it difficult to draw conclusions. Two laparoscopic techniques have emerged, the use of a mesh with a slit and a central keyhole and a mesh without a slit, the latter often termed as a modified-Sugarbaker. A third option has been also been described and consists in a combination of both techniques (known as the sandwich-technique).

Published series, however, are observational and often with a short length of follow-up. There is only one comparative study with level 3b of evidence, the one published by Muysons et al (11). In this study, the authors show that the modified-Sugarbaker technique offers significant better results in terms of recurrence than the key-hole technique (72,7% vs 15,4%), although the follow-up of those cases performed following the Sugarbaker technique is shorter than the rest of cases (30.7 vs 14 months). Together with this study, Hansson et al (40) show an very low recurrence rate (1,8%) once the key-hole technique is used with short-term follow-up (6 months). Latter publications from the same author (16), with longer follow-up of 36 months, show that the rate of recurrence with this technique becomes very high (37%). In these three studies the mesh used was a pure PTFE-e mesh, what leads us to conclude that when this mesh is used the key-hole technique should be avoided in favor to the modified-Sugarbaker one. If a PTFE mesh is used with the keyhole technique, parastomal hernia is likely to recur, and this mesh should preferably be used with the modified-Sugarbaker technique.

In this sense, series using other meshes following the key-hole technique, like the one from Liu et al(13) or Wara et al (15), using a e-PTFE-polypropylene mesh, show a low recurrence rate (4,1% and 3%, respectively), while the rate of the key-hole technique with a pure e-PTFE mesh is as high as 37%, in the report from Hansson et al (16),or 56% by Safadi et al (24) or even 72,7%, by Muysoms et al (11). On the other hand the results from Berger et al (20) following the sandwich-technique are also promising with a low recurrence rate, although there is only one series published using this technique.

In summary, the quality of evidence for the various surgical techniques for parastomal hernia repair is low and precludes firm conclusions. Randomized controlled trials would be ideal to compare the

various techniques of parastomal hernia repair, but none could be identified in the literature.

***REFERENCES***

1.- Tadeo-Ruiz G, Picazo-Yeste J.S, Moreno-Sanz C, Herrero-Bogajo ML. Parastomal Hernias: Background, current status and future prospects. Cir Esp2010;87(6):339-49.**(Level 5)**

2.-Hiranyakas A, Ho YH.Laparoscopic parastomal hernia repair. Dis Colon Rectum. 2010;53(9):1334-6.**(Level 5)**

3.- De Raet J, Delvaux G, Haentjens P, Van Nieuwenhave Y. Waist circunference is an independent risk factor for the development of a parastomal hernia after permanent colostomy. Dis Colon Rectum 2008;51(12):1806-9.**(level 4)**

4.- Pilgrim CH, McIntyre R, Bailey M. Prospective audit of parastomal hernia: prevalence and associated comorbidities. Dis Colon Rectum 2010;53(1):71-6.**(Level 2C)**

5.- Rubin MS, Schoetz JR DJ, Mathews J.B. Parastomal hernia. Is stoma relocation superior to fascial repair?.Arch Surg 1994;129:413-8.**(Level 3)**

6.- Cheung MT,ChiaNH,Chiu WY. Surgical treatment of parastomal hernia complicating sigmoid colostomies. Dis Colon Rectum 2001;44:266-70.**(Level 3)**

7.-Tekkis PP, Kocher HM, Payne JG. The continuing challenge of parastomal hernia: failure of a novel polypropylene mesh repair. Ann R CollSurgEngl 1998; 80:184-7.**(Level 5)**

8.- White TJ, Santos MC, Thmpson JS. Factors affecting wound complications in repair of ventral hernias. Am Surg 1998; 64:276-80.**(Level 3)**

9.-Pastor DM, Pauli EM, Koltun WA, Haluck RS, Shope TR, Poritz LS. Parastomal hernia repair: a single center experience. JSLS. 2009;13(2):170-5.**(Level 3b)**

10.-McLemore EC, Harold KL, Efron JE, Laxa BU, Young-Fadok TM, Heppell JP. Parastomal hernia: short-term outcome after laparoscopic and conventional repairs. SurgInnov. 2007;14(3):199-204. **(Level 3b)**

11.-Muysoms EE, Hauters PJ, Van Nieuwenhove Y, Huten N, Claeys DA. Laparoscopic repair of parastomal hernias: a multi-centre retrospective review and shift in technique. ActaChir Belg. 2008;108(4):400-4. **(Level 3b)**

12.-Mizrahi H, Bhattacharya P, Parker MC. Laparoscopic slit mesh repair of parastomal hernia using a designated mesh:long-term results. SurgEndosc2012; 26(1):267-70. **(Level 4)**

13.-Liu F, Li J, Wang S, Yao S, Zhu Y. Effectiveness analysis of laparoscopic repair of parastomal hernia using CK Parastomal patch. ZhongguoXiu Fu Chong JianWaiKeZaZhi. 2011;25(6):681-4.**(Level 4)**

14.-Jani K. Laparoscopic paracolostomy hernia repair: a retrospective case series at atertiary care center. SurgLaparoscEndoscPercutan Tech. 2010;20(6):395-8.**(Level 4)**

15.-Wara P, Andersen LM. Long-term follow-up of laparoscopic repair of parastomal hernia using a bilayer mesh with a slit. SurgEndosc. 2011; 25(2):526-30.**(Level 4)**

16.-Hansson BM, Bleichrodt RP, de Hingh IH. Laparoscopic parastomal hernia repair using a keyhole technique results in a high recurrence rate. SurgEndosc. 2009;23(7):1456-9. **(Level 4)**

17.-Zacharakis E, Hettige R, Purkayastha S, Aggarwal R, Athanasiou T, Darzi A, ZiprinP. Laparoscopic parastomal hernia repair: a description of the technique and initialresults. SurgInnov. 2008;15(2):85-9. **(Level 4)**

18.-Saber AA, Rao AJ, Rao CA, Elgamal MH. Simplified laparoscopic parastomal hernia repair: the scroll technique.Am J Surg. 2008; 196(3):e16-8.**(Level 4)**

19.-34. Craft RO, Huguet KL, McLemore EC, Harold KL. Laparoscopic parastomal hernia repair.Hernia. 2008; 12(2):137-40.**(Level 4)**

20.-Berger D, Bientzle M. Laparoscopic repair of parastomal hernias: a single surgeon's experience in 66 patients. Dis Colon Rectum. 2007;50(10):1668-73.**(Level 4)**

21.-Inan I, Gervaz P, Hagen M, Morel P. Multimedia article. Laparoscopic repair of parastomal hernia using a porcine dermal collagen (Permacol) implant.Dis Colon Rectum. 2007;50(9):1465.**(Level 4)**

22.-Mancini GJ, McClusky DA 3rd, Khaitan L, Goldenberg EA, Heniford BT, Novitsky YW, Park AE, Kavic S, LeBlanc KA, Elieson MJ, Voeller GR, Ramshaw BJ. Laparoscopic parastomal hernia repair using a nonslit mesh technique. SurgEndosc. 2007;21(9):1487-91. **(Level 4)**

23-.LeBlanc KA, Bellanger DE, Whitaker JM, Hausmann MG. Laparoscopic parastomal hernia repair.Hernia. 2005;9(2):140-4. **(Level 4)**

24.-Safadi B. Laparoscopic repair of parastomal hernias: early results. SurgEndosc. 2004;18(4):676-80. **(Level 4)**

25.-Kozlowski PM, Wang PC, Winfield HN.Laparoscopic repair of incisional and parastomal hernias after major genitourinary or abdominal surgery.J Endourol. 2001;15(2):175-9.**(Level 4)**

26.-Voitk A. Simple technique for laparoscopic paracolostomy hernia repair. Dis Colon Rectum. 2000;43(10):1451-3.**(Level 4)**

27.-Dumanian G. Laparoscopic repair of paraostomy hernias. J Am Coll Surg. 2002 Aug;195(2):293.**(Level 4)**

28.-Wara P. Parastomal hernia repair. An update. Minerva Chir. 2011; 66(2):123-8.**(Level 5)**

29.-Berger D. [Laparoscopic repair of parastomal hernia]. Chirurg. 2010; 81(11):988-92.**(Level 5)**

30.- Nagy A, Jánó Z. [Parastomal hernias].MagySeb. 2010; 63(5):335-9.**(Level 5)**

31.-Hiranyakas A, Ho YH. Laparoscopic parastomal hernia repair. Dis Colon Rectum. 2010; 53(9):1334-6.**(Level 5)**

32.-Israelsson LA. [Parastomal hernia treatment with prosthetic mesh repair].Chirurg. 2010;81(3):216-21. **(Level 5)**

33.-Berger D. [Laparoscopic IPOM technique]. Chirurg. 2010; 81(3):211-5. **(Level 5)**

34.-Israelsson LA. Parastomal hernias. SurgClin North Am. 2008; 88(1):113-25, ix.**(Level 5)**

35.-Voeller GR. Innovations in ventral hernia repair. SurgTechnol Int. 2007; 16:117-22.**(Level 5)**

36.-LeBlanc KA, Bellanger DE, Whitaker JM, Hausmann MG.Laparoscopic parastomal hernia repair.

Hernia. 2005 May;9(2):140-4. Epub 2004 Dec 16.**(Level 4)**

37.-Sugarbaker PH. Peritoneal approach to prosthetic mesh repair of paraostomy hernias. Ann Surg 1985;201:344-46.**(Level 4)**

38.-Sauerland S, Walgenbach M, Habermalz B, Seiler CM, Miserez M. Laparoscopic versus open surgical techniques for ventral or incisional hernia repair. Cochrane Database Syst Rev. 2011 Mar 16;(3):CD007781. **(Level 1A)**

39.-Kapischke M, Schulz T, Schipper T, Tensfeldt J, Caliebe A. Open versus laparoscopic incisional hernia repair: something different from a meta-analysis. Surg Endosc. 2008 Oct;22(10):2251-60. **(Level 1A)**

40.-Hansson BM, de Hingh IH, Bleichrodt RP.Laparoscopic parastomal hernia repair is feasible and safe: early results of aprospective clinical study including 55 consecutive patients.SurgEndosc. 2007; 21(6):989-93. **(Level 4)**

**Section 7: Comparison open vs. laparoscopic**

**Comparison of open vs. laparoscopic hernia repair: OR time, bowel lesion,**

**seroma and wound infection**

M. Rohr, J. Lang

A systemic search of the available literature was performed in August 2011 using Medline, PubMed, Cochrane library and relevant journals and reference lists using the above listed search terms

***Search terms:*** *open AND laparoscopic AND incisional AND hernia, open AND laparoscopic AND ventral AND hernia*

The first search detected 322 relevant articles. In a second-level search 339 articles were added. In summary 501 articles were founded, 59 articles were relevant and 38 were used for this review.

**Key questions:**

**OR time? Bowel lesion? Seroma? Wound infection?**

**Statements**

**OR-Time**

| Level 1A | There is no difference between open and laparoscopic techniques |
| --- | --- |
| Level 1B | Some studies shows longer, some shorter OR time for laparoscopic technique. The results are heterogeneous. |

**Bowel lesion**

| Level 1A | The laparoscopic approach seems to have a higher risk for bowel lesions. |
| --- | --- |

**Seroma:**

| Level 1 A | The results are heterogeneous, no significant difference between the open and laparoscopic techniques. |
| --- | --- |

**Wound infection:**

| Level 1 A | The laparoscopic approach has a significantly lower risk for wound  infections. |
| --- | --- |

**Recommendations**

| Grade A | Regarding the infection rate laparoscopic repair must be the preferred operative technique. |
| --- | --- |

No recommendations for OR Time or Seroma is possible.

**Introduction**

Since the introduction of laparoscopic hernia repair for ventral and incisional hernia, laparoscopic hernia repair has become the standard procedure. Over time enough RCTs comparing open vs. laparoscopic procedure have been published, so until now well-proved comparison of both techniques can be part of this guideline. Our question was to compare open and laparoscopic hernia repair concerning OR time, bowel lesions, seroma and wound infection.

**Discussion**

Concerning OR time we found very heterogenous results, with significance pointing both ways.

These heterogenous results were consistent through all levels of evidence. Altogether we found seven level 1a^1-3,6 13 14 22^, nine level 1b^4 5 7-12 16^, five level 2b^15 17-20^, one level 2c^22^, six level 3^23-28^ and one level 4^29^ studies that compared OR time lapaparoscopic vs. open with results ranging from significant shorter over same time up to significant longer for laparoscopic hernia repair. Since OR time is easy to measure, the problems seem to lie in incompatible standards be it different levels of experience, or normal evolution of a new surgical method from 1999 to 2011 with different operating techniques (suture / stapler). Other possible explanations would be different rates of adhesiolysis (partial vs. complete) and different patient collectives mixing spigelian, umbilical and incisional hernias.

Concerning bowel lesions we found similar results. Although Sauerland et al. ^1^ noted a possible bias for bowel lesion in the laparoscopic group, no significance was noted. We found three 1a^1 2 22^, four 1b^4 5 7 9^, four 2b^17-19 30^, one 2c^31^ and two 3^23 26^ comparing open vs. laparoscopic hernia repair and reporting bowel lesions. The level 2c study grouped bowel lesions together with visceral obstruction^31^ and a level 1a study only reported overall complications^6^. Only one study calculated a low significance (p=0,88) and reported more bowel lesions for the laparoscopic group (OR 2,19)^22^. The rest had too few enterotomies (0 – 5) due to small study numbers combined with excellent low complication rates. All in all nine studies reported more bowel lesion for laparoscopic surgery^1 2 4 5 9 19 22 26 30^, three had same rates^17 18 23^, one had no lesion at all^7^ and none reported more bowel lesion for open surgery. There may be a bias for bowel lesion in laparoscopic hernia repair but clearly more research is needed and the rates are low enough to be accepted as a bearable risk.

We found an entirely different picture for wound infections. Of all 29 studies (four 1a^1-3 22^, nine 1b^4 5 7-10 12 32 33^, seven 2b^15 17-20 30 34^, one 2c^21^, seven 3^23 25-27 35-37^ and one level 4^31^ one reported no infection^5^, one reported same rates for both methods^20^ one reported more infection for open surgery^30^ and the rest reported less wound infection (14 significant, including all level 1a studies). Therefore we can state that laparoscopic hernia repair has less wound infection than open hernia repair, which is quite important due to the delicate nature of a possible resulting net infection.

Seroma again shows heterogenous results.

Study Level	More seroma	Less seroma	Same rate

1a	3^1 22^ (1s^3^)	1^2^

1b	4^8 10^ (2s^5 9^)	3^4 7 12^ (1s^33^)

2b	2^17^ (1s^30^)	3 ^15 18 19^	1^20^

3	5^23 25-27 36^	1^35^

(s=significant, stated for laparoscopic hernia repair)

Unfortunately seroma was described using different standards (e.g. only reporting symptomatic seroma). With the new developed classification^38^ better research concerning this subject will be possible.

**Comment:**

Summarizing the experimental and clinical data, the data shows a heterogeneous result. The data are not comparable cause of differences in techniques (stapler, sutures) and experience account for different OR times.

There are too few high quality high volume studies.

The studies make no differentiation between ventral, umbilical or incisional hernias – several entities grouped together with very different patients.

There is no singular definition for seroma in studies (different times, symptomatic vs asymptomatic, need for aspiration. A definition for seroma is needed.

In all studies complications are often grouped together – difficult predictions for singular issues*.*

**Conclusion**

We can state that laparoscopic hernia repair has less wound infection compared to open repair. Concerning bowel lesions, OR time or seroma, no statement can be made due to contradicting evidence.

**References**

1. Sauerland S, Walgenbach M, Habermalz B, Seiler CM, Miserez M. Laparoscopic versus open surgical techniques for ventral or incisional hernia repair. *Cochrane database of systematic reviews (Online)* 2011(3):CD007781. **(1A)**

2. Forbes SS, Eskicioglu C, McLeod RS, Okrainec A. Meta-analysis of randomized controlled trials comparing open and laparoscopic ventral and incisional hernia repair with mesh. *The British journal of surgery* 2009;96(8):851-8. **(1A)**

3. Kapischke M, Schulz T, Schipper T, Tensfeldt J, Caliebe A. Open versus laparoscopic incisional hernia repair: something different from a meta-analysis. *Surgical endoscopy* 2008;22(10):2251-60. **(1B)**

4. Itani KM, Hur K, Kim LT, Anthony T, Berger DH, Reda D, et al. Comparison of laparoscopic and open repair with mesh for the treatment of ventral incisional hernia: a randomized trial. *Archives of surgery (Chicago, Ill. : 1960)* 2010;145(4):322-8; discussion 28. **(1B)**

5. Asencio F, Aguilo J, Peiro S, Carbo J, Ferri R, Caro F, et al. Open randomized clinical trial of laparoscopic versus open incisional hernia repair. *Surgical endoscopy* 2009;23(7):1441-8. **(1B)**

6. Sajid MS, Bokhari SA, Mallick AS, Cheek E, Baig MK. Laparoscopic versus open repair of incisional/ventral hernia: a meta-analysis. *American journal of surgery* 2009;197(1):64-72. **(1A)**

7. Pring CM, Tran V, O'Rourke N, Martin IJ. Laparoscopic versus open ventral hernia repair: a randomized controlled trial. *ANZ journal of surgery* 2008;78(10):903-6. **(1B)**

8. Olmi S, Scaini A, Cesana GC, Erba L, Croce E. Laparoscopic versus open incisional hernia repair: an open randomized controlled study. *Surgical endoscopy* 2007;21(4):555-9. **(1B)**

9. Barbaros U, Asoglu O, Seven R, Erbil Y, Dinccag A, Deveci U, et al. The comparison of laparoscopic and open ventral hernia repairs: a prospective randomized study. *Hernia : the journal of hernias and abdominal wall surgery* 2007;11(1):51-6. **(1B)**

10. Misra MC, Bansal VK, Kulkarni MP, Pawar DK. Comparison of laparoscopic and open repair of incisional and primary ventral hernia: results of a prospective randomized study. *Surgical endoscopy* 2006;20(12):1839-45. **(1B)**

11. Moreno-Egea A, Carrasco L, Girela E, Martin JG, Aguayo JL, Canteras M. Open vs laparoscopic repair of spigelian hernia: a prospective randomized trial. *Archives of surgery (Chicago, Ill. : 1960)* 2002;137(11):1266-8. **(1B)**

12. Carbajo MA, Martin del Olmo JC, Blanco JI, de la Cuesta C, Toledano M, Martin F, et al. Laparoscopic treatment vs open surgery in the solution of major incisional and abdominal wall hernias with mesh. *Surgical endoscopy* 1999;13(3):250-2. **(1B)**

13. Pham CT, Perera CL, Watkin DS, Maddern GJ. Laparoscopic ventral hernia repair: a systematic review. *Surgical endoscopy* 2009;23(1):4-15. **(1A)**

14. Goodney PP, Birkmeyer CM, Birkmeyer JD. Short-term outcomes of laparoscopic and open ventral hernia repair: a meta-analysis. *Archives of surgery (Chicago, Ill. : 1960)* 2002;137(10):1161-5. **(1A)**

15. Kurmann A, Visth E, Candinas D, Beldi G. Long-term follow-up of open and laparoscopic repair of large incisional hernias. *World journal of surgery* 2011;35(2):297-301. **(2B)**

16. Navarra G, Musolino C, De Marco ML, Bartolotta M, Barbera A, Centorrino T. Retromuscular sutured incisional hernia repair: a randomized controlled trial to compare open and laparoscopic approach. *Surgical laparoscopy, endoscopy & percutaneous techniques* 2007;17(2):86-90. **(1B)**

17. Lomanto D, Iyer SG, Shabbir A, Cheah WK. Laparoscopic versus open ventral hernia mesh repair: a prospective study. *Surgical endoscopy* 2006;20(7):1030-5. **(2B)**

18. Beldi G, Ipaktchi R, Wagner M, Gloor B, Candinas D. Laparoscopic ventral hernia repair is safe and cost effective. *Surgical endoscopy* 2006;20(1):92-5. **(2B)**

19. McGreevy JM, Goodney PP, Birkmeyer CM, Finlayson SR, Laycock WS, Birkmeyer JD. A prospective study comparing the complication rates between laparoscopic and open ventral hernia repairs. *Surgical endoscopy* 2003;17(11):1778-80. **(2B)**

20. Raftopoulos I, Vanuno D, Khorsand J, Kouraklis G, Lasky P. Comparison of open and laparoscopic prosthetic repair of large ventral hernias. *JSLS : Journal of the Society of Laparoendoscopic Surgeons / Society of Laparoendoscopic Surgeons* 2003;7(3):227-32. **(2B)**

21. Hwang CS, Wichterman KA, Alfrey EJ. Laparoscopic ventral hernia repair is safer than open repair: analysis of the NSQIP data. *The Journal of surgical research* 2009;156(2):213-6. **(2C)**

22. Sains PS, Tilney HS, Purkayastha S, Darzi AW, Athanasiou T, Tekkis PP, et al. Outcomes following laparoscopic versus open repair of incisional hernia. *World journal of surgery* 2006;30(11):2056-64. **(1A)**

23. Wolter A, Rudroff C, Sauerland S, Heiss MM. Laparoscopic incisional hernia repair: evaluation of effectiveness and experiences. *Hernia : the journal of hernias and abdominal wall surgery* 2009;13(5):469-74. **(3)**

24. Earle D, Seymour N, Fellinger E, Perez A. Laparoscopic versus open incisional hernia repair: a single-institution analysis of hospital resource utilization for 884 consecutive cases. *Surgical endoscopy* 2006;20(1):71-5. **(3)**

25. Olmi S, Magnone S, Erba L, Bertolini A, Croce E. Results of laparoscopic versus open abdominal and incisional hernia repair. *JSLS : Journal of the Society of Laparoendoscopic Surgeons / Society of Laparoendoscopic Surgeons* 2005;9(2):189-95. **(3)**

26. Bencini L, Sanchez LJ, Boffi B, Farsi M, Scatizzi M, Moretti R. Incisional hernia: repair retrospective comparison of laparoscopic and open techniques. *Surgical endoscopy* 2003;17(10):1546-51. **(3)**

27. Gonzalez R, Mason E, Duncan T, Wilson R, Ramshaw BJ. Laparoscopic versus open umbilical hernia repair. *JSLS : Journal of the Society of Laparoendoscopic Surgeons / Society of Laparoendoscopic Surgeons* 2003;7(4):323-8. **(3)**

28. Park A, Birch DW, Lovrics P. Laparoscopic and open incisional hernia repair: a comparison study. *Surgery* 1998;124(4):816-21; discussion 21-2. **(3)**

29. Ramshaw BJ, Esartia P, Schwab J, Mason EM, Wilson RA, Duncan TD, et al. Comparison of laparoscopic and open ventral herniorrhaphy. *The American surgeon* 1999;65(9):827-31; discussion 31-2. **(4)**

30. Bingener J, Buck L, Richards M, Michalek J, Schwesinger W, Sirinek K. Long-term outcomes in laparoscopic vs open ventral hernia repair. *Archives of surgery (Chicago, Ill. : 1960)* 2007;142(6):562-7. **(2B)**

31. Bisgaard T, Kehlet H, Bay-Nielsen MB, Iversen MG, Wara P, Rosenberg J, et al. Nationwide study of early outcomes after incisional hernia repair. *The British journal of surgery* 2009;96(12):1452-7. **(2C)**

32. Kaafarani HM, Kaufman D, Reda D, Itani KM. Predictors of surgical site infection in laparoscopic and open ventral incisional herniorrhaphy. *The Journal of surgical research* 2010;163(2):229-34. **(1B)**

33. Kaafarani HM, Hur K, Hirter A, Kim LT, Thomas A, Berger DH, et al. Seroma in ventral incisional herniorrhaphy: incidence, predictors and outcome. *American journal of surgery* 2009;198(5):639-44. **(1B)**

34. Zerey M, Heniford BT. Laparoscopic versus open surgery for ventral hernia repair--which is best? *Nature clinical practice. Gastroenterology & hepatology* 2006;3(7):372-3. **(2B)**

35. Solomon TA, Wignesvaran P, Chaudry MA, Tutton MG. A retrospective audit comparing outcomes of open versus laparoscopic repair of umbilical/paraumbilical herniae. *Surgical endoscopy* 2010;24(12):3109-12. **(3)**

36. Ballem N, Parikh R, Berber E, Siperstein A. Laparoscopic versus open ventral hernia repairs: 5 year recurrence rates. *Surgical endoscopy* 2008;22(9):1935-40. **(3)**

37. van't RM, Vrijland WW, Lange JF, Hop WC, Jeekel J, Bonjer HJ. Mesh repair of incisional hernia: comparison of laparoscopic and open repair. *The European journal of surgery = Acta chirurgica* 2002;168(12):684-9. **(3)**

38. Morales-Conde S. A new classification for seroma after laparoscopic ventral hernia repair. *Hernia : the journal of hernias and abdominal wall surgery* 2012;16(3):261-7. **(5)**

**COMPARISON OF HOSPITAL STAY, RETURN TO ACTIVITY, COST, QUALITY OF LIFE, PAIN AND RECURRENCE AFTER LAPAROSCOPIC AND OPEN VENTRAL AND INCISIONAL HERNIA REPAIR**

M. C. Misra, V. K. Bansal, P.P. Prakash, D. Babu, P. Singhal, R. Fortelny

Search Engine used: Pubmed, Cochrane database, Medline and relevant journals and reference lists in the English language till September 2011

**I)	HOSPITAL STAY**

Search term- “Hospital stay” AND “Laparoscopic incisional hernia repair” AND “Ventral hernia” AND “LIVHR” AND “Length of stay” AND “Laparoscopic vs open incisional hernia” AND “defect size” AND “primary ventral hernia” AND “fixation” AND “sutures” AND “tackers” AND “recurrent incisional hernia”

Total results	122

Relevant	25

Manual searches	40

Total	65

**Statements**

| 1a | Laparoscopic incisional and ventral hernia repair (LIVHR) significantly reduces hospital stay as compared to open repair |
| --- | --- |
| 1b | Hospital stay after suture fixation and tacker fixation are comparable |
| 2b | Hospital stay is significantly shorter after LIVHR in patients with hernias greater than 15 cm |
| 3 | The length of stay is shorter in LIVHR for primary ventral hernia as compared to incisional hernia |

**Recommendations**

| Grade A | Regarding hospital stay LIVHR must be the preferred operative technique. |
| --- | --- |

Hospital stay is an indirect indicator of many early postoperative variables like early postoperative pain, acute complications, and return to bowel functions. Minimal tissue dissection in laparoscopic incisional and ventral hernia repair has been reported to be associated with less postoperative pain and early return to bowel functions in many studies, leading to shorter hospital stay and eventually minimizing the overall cost of the procedure.

Three level 1a studies compared hospital stay between open repair and laparoscopic incisional and ventral hernia repair.In the Cochrane review 2011 [2], six [4, 5, 7-10]of the 9 trials [3-11]which reported length of hospital stay found a significant advantage for the laparoscopic group,the longest mean stay was 5.7 days for laparoscopic and 10 days for open surgery. Forbes et al [12](8 RCTs) and Sajid et al [13] (5 RCTs) reported similar findings in their meta-analysis. All of the studies included were later analyzed in the Cochrane review. One level 1b study compared the hospital stay between suture and tacker fixation and found the results to be comparable (1.13 vs 1.16 days; p = 0.77) [1].

Four level 2a studies [14-17] analyzed hospital stay. Mean hospital stay was shorter in 6 RCTs [4, 5, 7-10]and five nonrandomized studies. [18 - 22] Overall length of stay was shorter in laparoscopic incisional and ventral hernia repair (2 days vs 4 days; p = 0.02) [14].

There were ten level 3 studies [23 – 32] which reported on hospital stay with a follow up ranging from 4 to 44 months. The hospital stay ranged from 3-8.1 days in open repair and 2.1-6 days in laparoscopic repair. Five of them showed a significantly reduced hospital stay in laparoscopic repair group [26, 28-31].

There were 34 non-comparative studies (level 4) [33 – 66] in laparoscopic incisional and ventral hernia repair, which reported postoperative hospital stay to be ranging from 1 to 17 days. Kua et al [40] reported that 57% of their patients were discharged the next day of surgery while another 27% patients were discharged within 48 hours.Raftopoules et al [41] reported significantly less hospital stay after laparoscopic repair of primary ventral hernia as compared to incisional hernia (0.6 vs 2.2 days, p = 0.03). The possible explanation for this difference could be smaller defect size, fewer adhesions and a smaller size of prosthesis needing less number of tackers/sutures for fixation in primary ventral hernias leading to less dissection, and lesser pain leading to early discharge.

**Comments**

Hospital stay is shorter after laparoscopic incisional and ventral hernia repair, as compared to open repair.

Very few studies have comparedhospital stayinlaparoscopic incisional and ventral hernia repairin relation to defect size and no study is available reporting relation to mesh fixation, mesh type, and defect characteristics.

More RCTs are needed to study these aspects of LIVHR.

**II) RETURN TO ACTIVITY**

Search terms – “Return to work “AND “laparoscopic incisional hernia”AND “ventral hernia repair” AND “return to activity” AND “mesh fixation” AND “suture” AND “tacker” AND “defect size” AND “defect site” AND “recurrent incisional hernia” AND “type of mesh”

Total results	8

Relevant	3

Manual search	5

**Statements**

| 1a | There is no significant difference in time of return to activity in laparoscopic vs open repair |
| --- | --- |
| 1b | Laparoscopic incisional hernia repair is associated with a faster return to work as compared to open repair. |
|  | Suture fixation is associated with a faster return to work as compared to tacker fixation in laparoscopic repair |
| 2b | There is no difference in return to activity after suture or tacks fixation in laparoscopic incisional and ventral hernia repair |
| 4 | Smokers and patients with hard physical work demands takes significantly longer time for return to work |

**Recommendations**

| Grade A | Suture fixation is recommended in terms of early return to activity over tacker + suture |
| --- | --- |
| Grade B | Regarding faster return to work LIVHR must be the preferred operative technique. |

Return to daily activities and to work is an important measure for assessment of any surgical intervention. The patient contributes to social burden on his family and society till he is able to carry out his daily activities on his own and also the loss of work after the surgery has a definite economic impact on society. Thus, the time to return to activity and to work would indirectly measure the social and economic impact of the procedure on the society.

There are high expectations of early resumption of normal activities and returning to work by both the patients and the society after any laparoscopic procedure. But laparoscopic incisional and ventral hernia repair is different from other laparoscopic procedures, in terms of considerable early postoperative pain which may prolong the return to normal activities and return to work in many patients.

In the Cochrane review [2], there were 2 RCTs reporting on return to activity. Pring et al [11] showed no significant difference in return to activities, whereas Itani et al [6] showed that the time to resume work was shorter for the laparoscopic group as compared to the open repair group (median, 23.0 days vs 28.5 days, p = 0.06).

Olmi et al [10] in a RCT reported that, patients in laparoscopic group were able to return to work in significantly shorter time. The mean return to work was 13 days following laparoscopic incisional and ventral hernia repair as compared to 25 days following open repair (p = <0.005).

There is no level 2a evidence available on return to activity/work following open vs laparoscopic incisional and ventral hernia repair. Only one level 2b study available by Kurmann et al [22], reported earlier return to work after LIVHR, however the difference was not significant (21 vs 42 days, p > 0.05).

There is only one level 3 study by Raftopoulos et al [28], in which, although the mean return to activity was not significantly different, the mean return to work was significantly earlier following laparoscopic incisional and ventral hernia repair (25.9 vs 47.8 days, p = 0.036).

There are six level 4 studies [33, 39-40, 63, 67-8] reporting return to activities following laparoscopic incisional and ventral hernia repair. Kua et al[40] reported that 82% patients returned to household duties within a week following laparoscopic incisional and ventral hernia repair and most returned to normal activity within two weeks postoperatively.Eriksen et al [68] concluded that smokers and patients with heavy work demands took significantly longer time to return to household activities/work.

**Return to activities following suture or tacker fixation in laparoscopic incisional and ventral hernia repair**

Bansal et al in their RCT reported significantly shorter return to activity after suture fixation when compared to tacker fixation (p = <0.001) [1].

Nguyen et al(2b) [69] reported on return to activity after suture or tacker fixation in laparoscopic incisional and ventral hernia repair, and they reported no significant difference in return to activity after the two fixation techniques. They found that 50% and 42% patients returned to activities after one week following suture and tacker fixation respectively.

**Comments**

There are few RCTs comparing return to work comparing laparoscopic incisional and ventral hernia repair vs open repair.

Return to work is shorter or equivalent in laparoscopic incisional and ventral hernia repair compared to open repair.

No study is available which report return to activity in laparoscopic incisional and ventral hernia repair and mesh fixation, mesh type, and defect characteristics.

More RCTs are needed to analyze different aspects of laparoscopic incisional and ventral hernia repair such as fixation method, mesh and defect characteristics.

**III) COST**

Search terms – cost; laparoscopic incisional hernia repair; laparoscopic ventral hernia repair

**Statements**

| **Level 1A** | The cost of surgery is higher in laparoscopic group.  Shorter hospital stay may make laparoscopic surgery cost effective |
| --- | --- |
| **Level 1B** | Suture fixation is a cost-effective alternative to tacker fixation, for small and medium-sized defects in anatomically accessible areas.  Open repair is 9 times cheaper  Shorter hospital stay may reduce total cost |
| **Level 3** | Laparoscopic repair is costlier than open repair in terms of hospital cost but has decreased mean overall cost |

**Recommendations**

| Grade A | Suture fixation in Laparoscopic incisional hernia repair is recommended because of cost-effectiveness |
| --- | --- |
| Grade D | Laparoscopic incisional hernia repair can be recommended as a cost effective repair |

The rational allocation of scarce health care resources requires that the most cost-effective approach be used to deal with any clinical situation. It is necessary to analyze the cost of treatment modalities which have the same therapeutic potential. The laparoscopic approach to repair of incisional hernia has shown significantly lower mortality, reduced morbidity, fewer ICU admissions and 30-day readmissions, shorter hospital stay, and significantly reduced hospital costs for all the procedures.

Based on a systematic literature review in PUBMED, 42 studies analyzing costs involved in incisional and ventral hernia repair were identified. After studying the abstracts, 14 research papers were considered relevant and analyzed.

In Cochrane review [2], only one study by Misra et al [7] performed an economic analysis comparing open and laparoscopic repair and they found open repair to be 9 times cheaper compared to laparoscopic repair. While theoretical calculation by Olmi et al [10]showed the cost of operation in laparoscopic surgery to be higher as compared to open repair (1900 EURO vs 300 EURO), the overall cost was less than the open technique, probably due to shorter hospital stay.

There are 3 prospective studies [19, 67, 70] comparing cost of open and laparoscopic incisional hernia repair, all of which state that laparoscopic repair is cheaper. Demaria et al [19], attributed lower costs to lower readmission rates while Earle et al [70], attributed it to shorter hospital stay and lesser readmission cost.

There are 4 retrospective comparative studies comparing laparoscopic and open repair[23, 29, 31, 67]. In terms of cost, these studies have shown that the direct cost of surgery in terms of longer OR time, equipment and material cost, is higher in laparoscopy group but overall costs are lower or equivalent due to a decreased hospital stay and lower postoperative complication rate.

Bencini et al [29] showed that inspite of higher mesh cost in laparoscopic repair, it was cheaper due to the shorter hospital stay. Beldi et al [31] also showed similar results with direct cost of surgery higher in laparoscopy but lower overall hospital cost. These findings were complemented by 2 other studies by Holzman et al [23], and Wright et al [71].

Many variables such as mesh type, fixation technique, and technique of repair come into play in cost calculation. The modification of any of these cost variables will influence overall costs of either procedure. An RCT published by Bansal et al [1, 72] comparing suture mesh fixation with tacker mesh fixation showed that the overall cost of suture fixation was significantly lesser than tacker fixation for small to medium size defects ($575.42 more expensive, p = <0.001). Similarly the cost of mesh used dictates the overall cost involved. A prospective study by Alkhoury et al [73], comparing cost and clinical outcomes of laparoscopic ventral hernia repair using non-heavyweight polypropylene mesh and other meshes showed that polypropylene meshes were substantially cheaper with a cost saving of $436 per patient with Proceed, $770 per patient with Composix, and $931 per patient with ePTFE mesh. A retrospective comparative study published by Bencini et al [29], also showed that polypropylene mesh is significantly cheaper than PTFE mesh.

**Comments**

There were no studies with full economic evaluation focusing on the relevant alternatives.

The studies did not primarily aim to investigate costs or cost effectiveness.

The cost analysis was done in a highly insufficient manner and hence more studies are strongly recommended to address these issues.

**IV) QUALITY OF LIFE**

Search terms – “Quality of life” AND “laparoscopic” AND “incisional hernia” AND “ventral hernia repair” AND “open incisional hernia repair” AND “patient satisfaction” AND “cosmesis” AND “mesh fixation” and “suture” AND “tacker” AND “defect size” AND “recurrent incisional hernia”

Results	27

Relevant	7

Manual searches	4

Total	11

**Statements**

| 1a | There is no difference in quality of life after open and laparoscopic repair of incisional and ventral hernia. |
| --- | --- |
| 1b | Use of absorbable sutures with tacks leads to better quality of life as compared to tacks with non-absorbable sutures or tacks only |
|  | There is no difference in quality of life after suture or tacker fixation in laparoscopic repair of incisional and ventral hernia. |
| 2b | Laparoscopic repair leads to significant improvement in QOL as compared to open |
| 4 | Laparoscopic ventral hernia repair leads to a significant improvement in quality of life of the patient |
|  | Patient satisfaction is more after laparoscopic ventral hernia repair compared to open repair |
| 5 | Patients are satisfied cosmetically after suture fixation |

**Recommendations**

| Grade A | Laparoscopic repair is recommended with comparable QOL as open repair |
| --- | --- |

Quality of life (QOL) of patients has become a central evaluation parameter for chronic illness and chronic morbidity and also aids in decisions related to treatment strategies. Quality of life is not measured directly but is commonly sampled by using measurement scales in the form of questionnaires.

**QOL in Open vs laparoscopic ventral hernia repair**

In the Cochrane review [2], only 2 out of 9 RCTs compared quality of life and reported no significant difference in QOL in open vs laparoscopic ventral hernia repair [3, 6]. Patient satisfaction with the cosmetic appearance was examined in only one of the trials [7] and there was no significant difference in cosmetic outcomes also (p=0.26).

There are two level 2b studies which compared QOL between open and laparoscopic ventral hernia repair. Mussak et al [74] found no significant difference in any of the domains of SF-36 questionnaire. Patients were evaluated at 16 months after laparoscopic ventral hernia repair and 28 months after open repair. Whereas Hope et al [75] reported improvement in postoperative QOL in 4 of the 8 domains of SF-36 (general health, vitality, role emotional, mental health) in laparoscopic ventral hernia repair as compared to open repair. They also measured QOL on Carolinas Comfort Scale and reported improvement in all physical variables in laparoscopic ventral hernia repair as compared to open repair.

Uranues et al [76],and Eriksen et al [68],(level 4) reported substantially improved health-related QOL after laparoscopic incisional hernia repair. While Uraneus et al[76] reported significant improvement in 3 of the 5 GIQIL domains (symptoms, emotional function and physical function); Eriksen et al [68] measured the SF-36 domains and found improvement in general wellbeing, body pain and fatigue.

**QOL and technique of fixation**

Different fixation techniques (suture or tacker) may be associated with varying degrees of early postoperative and chronic pain and may affect the quality of life postoperatively.

Bansal et al in an RCT compared the QOL after fixation with either only sutures or with tackers with non-absorbable sutures and found no significant difference between the two groups [1]. A study by Wassener et al [77] which evaluated QOL in a RCT of three fixation techniques, tacks + absorbable sutures(TAS), double crown of tacks (DC) and tacks + non-absorbable sutures(TNS) and found that tacks + AS group was significantly better than double crown tacks in physical functioning and role limitation due to emotional problem (p=0.02).

**Patient satisfaction in laparoscopic ventral hernia repair**

Patient satisfaction is an indicator of postoperative quality of life and the cosmetic outcome of the patients. We found two studies commenting on the patient satisfaction. Bansal et al [1, 72], in their RCT of suture vs tacker fixation in laparoscopic ventral hernia repair, reported that patients were satisfied cosmetically after suture fixation, however no patient satisfaction scores were calculated. In a continuation of this study by the same author the patient satisfaction scores were calculated and found to be statistically insignificant between suture vs tacker fixation. Perrone et al(4) [55], reported that patient satisfaction score was high following laparoscopic incisional hernia repair.

**Comments**

There are few RCTs comparing QOL in laparoscopic vs open ventral hernia repair.

Very few studies have compared QOL in different aspects of laparoscopic ventral hernia repair like mesh, fixation and defect characteristics.

Different methods have been used for QOL assessment in different studies, making it difficult to compare and analyze them.

More RCTs are needed to evaluate different parameters of laparoscopic ventral hernia repair with one standardized method.

**V) PAIN**

Search terms: “pain” AND “laparoscopic” AND “incisional hernia repair” AND “ventral hernia” AND “LIHVR” AND “mesh fixation” AND “suture” AND “tackers” AND “type of mesh” AND “factors for pain” AND “defect size” AND “defect site” AND “pain” AND “acute pain” AND “chronic pain” AND “recurrent incisional hernia” AND “preoperative pain” AND “postoperative pain”.

Results	113

Relevant	39

Manual search	10

Total	49

**Statements**

| Level 1a | Incidence of pain, both acute& chronic, is not significantly different between open and laparoscopic ventral hernia repair. |
| --- | --- |
| Level 1b | Incidence of early postoperative pain and chronic pain is less in suture fixation as compared to tacker fixation in laparoscopic repair |
|  | There is no significant association of chronic pain in laparoscopic ventral hernia repair with preoperative pain. |
|  | There is no difference in pain between Heavyweight Polypropylene mesh and Lightweight barrier-coated meshes. |
| Level 2b | Chronic postoperative pain is more following laparoscopic ventral hernia repair of recurrent as compared to primary cases. |
| Level 4 | Fixation with both tacks and transfixation suture results in more pain. |
|  | Pain after laparoscopic ventral hernia repair is mostly at suture site. |
|  | Defect closure may lead to chronic pain. |
| Level 5 | Sutures cause ischemic injuries to anterior abdominal wall musculature or neurovascular bundle resulting in pain. Nerve entrapment in tacker is another possible explanation to the post-operative pain. |

**Recommendations**

| Grade A | Regarding pain scores laparoscopic ventral hernia repair must not be preferred. |
| --- | --- |
| Grade A | Only suture fixation in small and medium sized defect may result in less pain and can be recommended. |

Pain is one of the central outcomes after incisional hernia repair. While the big incision is the obvious cause of postoperative pain following open repair, the small incision in laparoscopic repair does not make it a minimally painful procedure, as in other laparoscopic procedures. The use of transfascial sutures and tacker causes substantial postoperative pain and in many patients may be a cause of chronic pain even after months or years of surgery. Increasingly post-operative pain rather than recurrences has become the most important outcome measure following laparoscopic incisional and ventral hernia repair. Consequently, current interest focuses increasingly on the genesis of pain after laparoscopic ventral hernia repair and methods to reduce such pain.

Various factors responsible for chronic pain have been cited like type of mesh fixation, defect closure, recurrent incisional hernias and type of mesh. An evidence based analysis was done to study pain in laparoscopic ventral hernia repair and also various factors predisposing to chronic pain.

There are two systematic reviews of RCTs (1a)which report on postoperative pain following laparoscopic vs open incisional hernia repair. In the Cochrane review [2], which was a meta-analysis of 10 RCT’s comprising 880 patients, there were 4 RCTs (Asencio et al [3], Barbaros et al [4], Misra et al [7] and Pring et al [11])which measured pain after surgery, and in all of them, the intensity of pain was similar between the two techniques. Sajid et al [13] analyzed 5 RCT’s which were also included in the Cochrane review and reported similar findings with no difference in overall postoperative pain between laparoscopic and open repairs (p = 0.84).

**Chronic pain**

The incidence of chronic pain following laparoscopic incisional/ventral hernia repair has been reported to be ranging from 1–3% in literature [78]. Chronic pain can be of significant concern in many patients, leading to prolonged consumption of analgesics and restriction in daily activities.

There are only two RCTs, which report chronic pain in laparoscopic ventral hernia repair vs open repair. Asencio et al (1b) [3],reported no significant difference in mean pain scores at 3 months and 1 year follow up. In fact the mean pain scores were higher in the laparoscopic ventral hernia repair group, although non-significant. Itani et al (1b) [6],reported that mean worst pain after 1 year was significantly less in the laparoscopic group (15.2 mm lower on a visual analogue score of 0-100 mm), but the article fails to contain mean values for both groups.

Three systematic review of non-randomized studies (2a) were identified, Pierce et al [17](review of 14 paired and 31 unpaired studies),Muller et al [79] (review of 14 comparative studies), and Cassar et al [80] (review of 19 studies), including a total of 9,244patients (open 2,102/LIVHR7,384) and mean follow up of 24 months following open repair and, 17.3 months following laparoscopic ventral hernia repair. Both Pierce et al [17]and Muller et al [79] reported no difference in chronic pain following laparoscopic or open incisional/ventral hernia repair. Cassar et al [80] reported the mean incidence of chronic pain as 1.8% in 4 out of 19 studies.

There were 15 level 4 studies [47-50, 54-5, 57, 59-62, 65-6, 76, 81] which reportedincidence of chronic pain comprising of 4236 patients and follow up ranging from 6-64 months. The incidence of chronic pain varied from 1 - 14.7% in various studies. Heniford et al [49]reported that pain was mostly at suture site. Sharma et al [65]reported that there was more pain after use of both tacks and transfixation sutures.

**Pain and type of fixation - Suture or tacks?**

The pain in laparoscopic incisional/ventral hernia repair is related to mesh fixation either with tacks or sutures. The pain due to fixation is different from that at the port sites. The postoperative pain produced by the fixation techniques could play an important role in deciding between sutures and tacks for mesh fixation.

There is no level 1a evidence regarding association of pain and method of fixation. There are 3 RCTs (1b) which studied the association of pain with the type of fixation. Wassener et al [77] conducted a RCT ofthree fixation techniques (tacks with absorbable sutures or nonabsorbable sutures or only tacks), and they found no significant difference in VAS scores at any time during a follow up of 3 months between the three techniques of mesh fixation. On the other hand, Bansal et al [72] reported higher pain scores in tacker fixation group compared to suture fixation group in early post-operative period and follow-up at 3 months, however on continuation of the study they found that chronic pain at 1 month was significantly more in tacker fixation and this became insignificant at 3 months [1]. However, there was no difference in the chronic pain at long term follow up. In another RCT, Beldi et al [82] 2011 did not find any significant difference in VAS scores following tacker vs suture fixation at 6 months follow up.

There are 3 nonrandomized comparative studies (2b) which reported chronic pain following suture and tacker fixation. Nguyen et al [69] reported no significant difference between the two fixation groups in their nonrandomized comparative trial comparing suture vstacker. Beldi et al [31] and Kurmann et al [22] reported during their assessment of pain following laparoscopic incisional and ventral hernia repair vs open repair, that pain following laparoscopic ventral hernia repair is mostly at the transfixation suture site.

Four non comparative trials (level 4) [49, 65, 83-4] were identified comprising of 2649 patients and follow up ranging 1-120 months. The chronic pain was seen in upto 16.4% in suture and 12.7% in tacker group and it was seen that chronic pain was highest in patientswhere both tacks and sutures were used (Sharma et al [65],p = 0.078).However, in a study by Chelala et al [57]using transfascial suture fixation only, 97.5% of their patients were pain free. Seven (1.75%) patients reported chronic pain, which gradually resolved, and three (0.75%) required excision of a neuroma at the suture fixation site.

Bedi et al [85] in a review of 34 original studies commented that sutures for mesh fixation may cause ischemic injuries to anterior abdominal wall musculature or neurovascular bundle which results in pain. Nerve entrapment in tacker is another possible explanation for postoperative pain. (Level 5)

**Association of chronic pain**

The association of seroma to chronic postoperative pain has not been studied very well in the literature. There has been only one study by Aura et al(4) [45]which has mentioned higher incidence of chronic postoperative pain following seroma formation.

Mckinlay et al [86](2b)analyzed the incidence of chronic pain following laparoscopic ventral hernia repair of primary and recurrent incisional hernias and reported chronic postoperative pain > 6 months in 2 of 69 of recurrent (2.8%) as compared to no chronic pain in 101 primary cases.

The mesh material may also play an important role in the causation of pain. Bansal et al [1, 72](1b) studied the association of chronic pain with type of mesh and did not find any difference in pain score between heavyweight polypropylene mesh and lightweight barrier coated meshes, either acute pain at 6hr, 24 hr and 7 days or chronic pain at 1 month and 3 months.

The efficacy of mesh repair is based on the formation of a strong mesh aponeurotic scar tissue complex (MAST complex). But inflammation beyond the optimum range may entrap neural structures leading to chronic pain. Today large numbers of lightweight composite meshes are available which are claimed to produce optimum fibrotic reaction and to decrease the incidence of chronic pain. However, there are not many studies available comparing the composite meshes with polypropylene meshes.

Chelala et al [57](4) and Franklin et al [53](4) reported incidence of chronic pain 2.5% and 3.1% respectively after defect closure. This may indicate that closure of the defect with subsequent traction may even contribute to chronic postoperative pain.

No study was found depicting the association of chronic pain with acute pain, preoperative pain and the site and size of defect.

**Comments**

None of the studies evaluated pain as the primary outcome

No study compares association of preoperative and postoperative pain.

Few RCTs have reported pain after laparoscopic incisional/ventral hernia repair, mainly comparing with open repair. Even less studies reported on chronic pain.

Sample size is small in all RCTs.

Very few studies evaluated association of pain with method of fixation and type of mesh.

No data available regarding relation with defect site, defect size, acute pain and recurrent hernias.

More RCTs are needed with more number of patients and with longer follow up.

Only short term data should not be recorded as chronic pain is not infrequent after hernia repair

Larger trials should also include separate analysis of primary ventral and incisional hernia.

Studies are strongly needed to assess the relation of pain with fixation, type of mesh, defect size and site, and recurrent hernias

**VI) RECURRENCE**

SEARCH TERMS- “laparoscopic incisional hernia repair“ AND “LIVHR“ AND “incisional hernia“ AND “ventral hernia“ AND “open hernia repair” AND “ recurrence rates“ AND “relapse”.

**Statements**

| Level 1a | No significant difference in recurrence between open and laparoscopic incisional/ventral hernia repair. |
| --- | --- |

**Recommendations**

| Grade A | Regarding recurrence rate laparoscopic ventral hernia repair must not be preferred. |
| --- | --- |
| Grade B | Suture and Tacker fixation are equally effective, however, all suture fixation is more cost effective in small and medium sized defects |

Recurrence is one of the most important outcomes of incisional and ventral hernia repair. Theoretically laparoscopic exploration allows us to inspect the whole of the previous incision and to cover it with a mesh, thus reducing the probability for a new hernia .On the other hand, laparoscopic repair does not include closure of the defect and therefore fully relies on the strength of the mesh and its fixation. Recurrence depends on various patient, hernia, tissue and technique related factors. Patient factors such as age, gender, body mass index, diabetes, malnutrition, collagen vascular disease, malignancy, etc influences recurrence rates.The type of hernia, location, number, and size of hernia along with the nature of index surgery and type of incision also dictates recurrence rates. It has been shown that those with altered collagen ratios and associated history of wound infection might have higher rates of recurrence after incisional hernia repair. Most of these risk factors are constant and cannot be altered but technical factors such as type of repair, type of mesh used, method of mesh fixation, margin of mesh overlap are certain factors that can significantly influence and bring about changes in the recurrencerates.

There are 3 meta-analysis [2, 12, 13] comparing recurrence rates in laparoscopic and open repair of incisional hernia, comprising of a total of 880 patients, 446 laparoscopic and 434 in open repairs. None of the studies demonstrated any significant difference in recurrence rates between the two techniques (RR 1.22; 95% CI 0.62 to 2.38; p=0.58) aftera follow up of 2-68 months. The recurrence rate was low probably due to short follow up in majority of trials.The follow up was less than two years in half of the trials [2].Forbes et al [12] in a meta-analysis of 8 RCTS consisting of 517 patients found no significant difference in recurrence between laparoscopic and open repair at a mean follow up of 23 months. The overall recurrence rate was low due to the small size of hernia in most of the studies and the lack of uniform definition of recurrence.

There are only 9 RCTs [3-11] comparing recurrence in laparoscopic and open incisional and ventral hernia repair and seven of these RCTs did not find any significant difference in recurrence between the two techniques whereas two (Carbajo et al [5]and Barbaros et al [4]) showed a lower recurrence rate with laparoscopic repair. Barbaros et al randomised 23 patients each to laparoscopic and open repair and found that the recurrence rate after laparoscopic repair was significantly lower ( p< 0.05). They had only one recurrence which was in the open group. They attributed their low recurrence rate in laparoscopic repair to the method of fixing mesh with both tackers and 4 corner sutures [4].Whereas Carbajo et al, who also showed a significantly lower recurrence in the laparoscopy group (p< 0.05) over a follow up period of two years but they had used different methods of fixation [5]

The various reasons for recurrence were discussed in these studies. Misra et al [7], attributed recurrence to inadequate space for mesh fixation in a low lying defect, Olmi et al [10],to inadequate mesh overlap and Itani et al [6],to post-operative surgical site infection.

Eight systematic reviews [15-17, 79-80, 85, 87-8] of prospective studies comparing laparoscopic and open ventral and incisional hernia repair were identified with 19,421 patients. The recurrence rates ranged from 0- 20.7 % in laparoscopic group and 0 -35% in open group with a follow up range of 1 -85 months. There was no statistically significant difference in recurrence rates in any of the studies except in one study reported by Pierce et al [17], which showed a significantly lower recurrence rate in laparoscopic repair .They published a pooled data analysis of 45 studies over a period of 12 years comparing laparoscopic versus open ventral hernia repair representing 5340 patients (4582 laparoscopic, 758 open). In the pooled analysis (combined paired and unpaired studies), laparoscopic repair was associated with significantly lower hernia recurrences (p < 0.0001).

Various probable causes for recurrence were also identified. Cassaret al [80] reviewed 19 prospective comparative studies comprising of a total of 1896 patients, 1598 in laparoscopic repair group and 298 in open group, and found higher recurrence rates in large hernias and those patients who had wound infection. They also found that staples alone were inadequate for fixation of mesh and that interval between 2 staples should be less than 1 cm.Bedi et al [85]stated that recurrence decreases with use of transfacial sutures and with experience.

Nine prospective comparative studies were identified [18-22,30,86,89-90]with total 1298 patients (773 laparoscopic group and 525 open group). The recurrence rate following laparoscopic repair ranged from 2-21% and following open repair was 0-16% with a follow up of 9 – 65 months. Recurrence rates were significantly lower in laparoscopic group in two studies. Bingener et al [21]compared laparoscopic repair with open repair prospectively with 127 patients in laparoscopic group and 233 patients in open group with a follow up of 25-36 months. Overall recurrence rates in laparoscopic group was 9% and 12% in open group (p=0.36).They also found that in patients with BMI > 30 ,there was a five-fold increase in recurrence rates and in those with post-operative abscess formation there was a 4.4 fold increase in recurrence rates. Ceccarelli et al [90], in a comparison of 94 patients of laparoscopic repair with 87 patient of open repair found a significantly lower recurrence rate following laparoscopic repair (p>0.05). They postulated that recurrence rates were lower because laparoscopy helps to identify defects not clinically identifiable. It was also found that recurrence was higher in morbidly obese and advocated a weight loss program before surgery.

It was also noted that lateral defects [18],larger defects [22, 86],BMI > 30 Kg/m^2^ [22] and those with peri-operative complications [18,22] had a significantly higher recurrence rate and Patients with recurrent or multiple hernia also had a higher rate of recurrence though not statistically significant [22].McKinlay et al [86],compared laparoscopic repair in 69 recurrent hernias and 101 primary hernias. The recurrence rate following repair of recurrent hernias was 7% while following non-recurrent hernias it was 5%.Though the difference was not statistically significant (p=0.53) but the mean time to recurrence was shorter in recurrent group, p = <0.0001.

Eight retrospective studies [24-6, 29, 32, 60, 74, and 90] were identified comprising of a total of 765 patients. The recurrence rate ranged from 0 - 15.7% in laparoscopic repair in a followup of 6-40 months. Zografos et al [32] analyzed 106 patients retrospectively comparing 30 laparoscopic repairs with 76 open repairs over 40 months. The recurrence rates following laparoscopic repair was 3.3 % compared to 2.6 % following open repair. The recurrences following laparoscopic repair were due to inadequate fixation of the mesh causing the mesh to slip out and only 4 corner sutures were insufficient to hold the mesh and so was only staplers, as staplers fix the mesh only to the peritoneum. Due to the abdominal pressure mesh migrates into the defect if not fixed to the fascia. Ceccarelli et al [90]postulated that the causes for recurrence in laparoscopic repair are rolling up of mesh, incomplete stretching of mesh and incomplete covering of the defect .

56 case series [24-6, 29, 32, 34-48, 50, 52-7, 59-68, 74, 76, 81, 83-4, 91-104] of laparoscopic repair including a total of 8677 patients were identified. The recurrence rates ranged from 0-20% over a follow up of 1-84 months. BMI> 37, defect size >10 cm, multiple Swiss cheese defects, recurrent hernias, long operating time, higher perioperative complication rate were identified to be associated with a significantly higher recurrence rate [47,64]. Inadequate mesh overlap, size, fixation and dissection were also identified to be risk factors for recurrence [38,47].

It has been noted that recurrences commonly occur at the mesh margins along the mesh-tissue interface. This finding has been validated by an experimental study which found that increasing the mesh overlap to 4 cm from the defect edges eliminated mesh disruption [92].In many of the studies, 3-5 cm overlap of the mesh has been used and recurrence rates have been reported to be less than 5 % [6,80,93] The study by Park et al [18]had a very high recurrence rate of 11% and on analysis it was realized that the mesh overlap in their patients was only 2.5 cm which was responsible for the high recurrence rates^.^ Theodoropoulous et al [94] had a recurrence in the periphery of the mesh despite a 3 cm overlap. LeBlanc et al [87], reviewing the literature on fixation techniques recommended that the minimum mesh overlap should be 4 to 5 cm if transfascial sutures are not used and at least 3 cm when trans-fascial sutures are used. Mesh size is equally important. Wassener et al [59] stated that the mesh should cover not just the defect but the entire incision in order to prevent recurrence. A larger sized mesh may protrude through the defect causing recurrence. Mesh contraction and migration into defect is common with a smaller size mesh.

Uraneus et al [76]studied recurrence rates following laparoscopic repair in recurrent hernias and reported that the risk was similar to that of primary repair (3.5%).Chelala et al [57]in their series of 400 cases noted that recurrence could be due to non-closure of the defect with extrusion of mesh into sac especially when mesh size is insufficient.

Mesh fixation is an important determinant of recurrence rates. Variable recurrence rates have been reported in the literature with the use of different mesh fixation techniques.

Three RCTs comparing various fixation devices and techniques were identified. None of them showed any significant difference in terms of recurrence between suture only, suture with tacker and tacker only fixation.

Similarly 2 systematic reviews with a total of 6824 patients were also identified which showed no significant difference between suture and tacker fixation[87, 88]. In a collective review of 23 studies and 12 comparative studies by LeBlanc et al [87],mesh fixation with sutures only resulted in the lowest recurrence rate (0.8%) compared to that by tacks alone (1.5%). Mesh fixation with tacks and sutures resulted in a recurrence rate of 3.5% at a mean follow up of 22 months.

**Studies using tacks and sutures for mesh fixation**

Bansal et al in their RCT randomized 106 patients, into suture and tacker fixation reported two recurrences both in the tacker fixation group over a mean follow-up period of 31 months [1]. Ben-haim et al [101] presented a retrospective study of 100 patients who underwent expanded polytetrafluoroethylene mesh fixation with both trans-fascial sutures and tacks. The exact mesh fixation technique and mesh overlap size were not mentioned. The recurrence rate was 2% over a mean follow-up period of 19 months. The reasons for recurrence were stated to be detachment of tacks and inadequate mesh overlap.

Heniford et al [83] have published the largest series of 850 patients undergoing laparoscopic ventral hernia repair. Mesh fixation was done by tacker and 4 corner sutures. Patients with a previous history of open repair comprised 34 % and a higher recurrence rate were seen in this group. The mean recurrence rate was 4.7% at a mean follow-up of 20 months. LeBlanc et al [47] published their series of 200 patients out of which 43 patients had multiple defects. With the use of trans-fascial sutures in the second half of their study, the recurrence rate decreased from 9% to 4%. Franklin et al [53] presented a large retrospective series of 384 patients in whom the mesh was fixed with tacks and trans-fascial sutures. 11 recurrences (2.9%) were seen over a follow-up period of 47 months. The recurrences were found to be most frequent (eight) in patients in whom trans-fascial sutures were not used. Ujiki et al [52] published a prospective series of 100 patients in 2004, fixed with tacks and a minimum of 4 trans-fascial polypropylene sutures, keeping a mesh overlap of at least 3 cm. The recurrence rate was 6 % over a mean follow up period of 3 months. The recurrences were attributed to intraoperative trans-fascial suture breaks and not being able to place the fourth trans-fascial suture in one patient. Bower et al [81] presented a series of 100 patients who underwent mesh fixation with both trans-fascial sutures and tacks and reported a recurrence rate of 2% at a mean follow-up of 6.5 months. Patients with a body mass index of greater than 30 Kg/m² accounted for 73% of the complications.

Perrone et al [55] presented a series of 116 patients with 28.9% of their cases being recurrent hernias, and the hernia recurrence rate was 9.3%.

Berrevoet et al [60] published a multicenter study in 2009 with 114 patients who underwent composite mesh (Proceed) fixation with tacks and trans-fascial sutures. The mean recurrence rate of 3.5 % over a mean follow-up of 27 months.

**Studies using tacks only for mesh fixation**

The largest study performed by Carbajo et al [48] followed 270 patients prospectively for a median follow-up of 44 months. Approximately 95% of patients had hernia defects greater than 5 cm, as 147 had defects between 5 and 10 cm while 108 patients had defects greater than 10 cm. They demonstrated a recurrence rate of 4.4%.

Another retrospective review performed by Frantzides et al [105] followed up 208 patients for a median of 24 months and demonstrated a low recurrence rate of 1.4%. Their operative technique involved only tacks placed 1 cm apart.

A long-term retrospective study by Bageacu et al [100] collected data on 159 patients with a median follow-up of 49 months. In contrast to the study by Carbajo et al [48], this study included smaller hernia defects, 46% were smaller than 5 cm, 24% between 5 and 10 cm, and 23% were greater than 10 cm. The recurrence rate was high at 15.7% and all recurrences were confirmed with a CT scan after clinical suspicion. The authors suggested that their higher recurrence rate might be attributable to a technical learning curve since their recurrence rate dropped from 20 to 10% between the periods of 1993–95 and 1996–98 respectively.

Another study using only tack fixation was performed by Kirshtein et al [44] which followed 103 patients with a mean of 26 months. They demonstrated a complication and recurrence rate of 4%. All four recurrences occurred within the first month suggesting a preventable etiology such as inappropriate fascial patch placement with mesh dislodgement. They concluded that using a double row of tacks might be required to prevent mesh migration. Gillian et al [102] presented a study of 100 patients with a mean follow-up of 27 months. Mesh fixation was by a double-crown technique, and the recurrence rate was 1%. While Chowbey et al [92] presented a series of 202 patients in whom mesh was fixed with a single crown of tacks. The recurrence rate was 1 % over a mean follow-up of 29 months.

Wassenaar et al [59]published a randomized controlled trial comparing mesh fixation with double crown tacks alone, tacks with non-absorbable sutures and tacks with absorbable sutures. They found no difference in recurrence rate at 2 weeks, 6 weeks and 3 months post-operatively among the three groups (p=0.38, 0.76, 0.41 respectively).

**Studies using only trans-fascial suture fixation**

Chelala et al [57] analyzed 400 cases where mesh was fixed with trans-fascial suture only. They closed the hernia defect with non-absorbable sutures. The mean operative time was 74 minutes and there was no recurrence detected over a mean follow-up of 28 months.

Varghese et al [42], reported that tacking of mesh to Coopers ligament is not sufficient. Berger et al [43] described a case of dislodgement of tackers when fixed to pubic symphysis.

There are no studies comparing recurrence rates and type of mesh.

**Comments**

The present data on recurrence does not give us any precise estimates. The total number of patients is small and the follow up period is short. Theoretically laparoscopic exploration allows us to inspect the whole of the previous incision and to cover it with a mesh, thus reducing the probability for a new hernia. On the other hand, laparoscopic repair does not include closure of the defect and therefore fully relies on the strength of the mesh and its fixation. More studies are required to consider these issues.

**References**

1. Bansal VK, Misra MC, Babu D, Singhal P, Rao K, Sagar R, Kumar S, Rajeshwari S, Rewari V (2012) Comparison of long-term outcome and quality of life after laparoscopic repair of incisional and ventral hernias with suture fixation with and without tacks: a prospective, randomized, controlled study. Surg Endosc. 2012, Dec; 26(12): 3476-85.**(1b)**
2. [Sauerland S](http://www.ncbi.nlm.nih.gov/pubmed?term=Sauerland%20S%5BAuthor%5D&cauthor=true&cauthor_uid=21412910), [Walgenbach M](http://www.ncbi.nlm.nih.gov/pubmed?term=Walgenbach%20M%5BAuthor%5D&cauthor=true&cauthor_uid=21412910), [Habermalz B](http://www.ncbi.nlm.nih.gov/pubmed?term=Habermalz%20B%5BAuthor%5D&cauthor=true&cauthor_uid=21412910), [Seiler CM](http://www.ncbi.nlm.nih.gov/pubmed?term=Seiler%20CM%5BAuthor%5D&cauthor=true&cauthor_uid=21412910), [Miserez M](http://www.ncbi.nlm.nih.gov/pubmed?term=Miserez%20M%5BAuthor%5D&cauthor=true&cauthor_uid=21412910).Laparoscopic versus open surgical techniques for ventral or incisional hernia repair.[Cochrane Database Syst Rev](http://www.ncbi.nlm.nih.gov/pubmed/21412910) 2011 Mar 16;(3): CD007781. **(1a)**
3. Asencio F, Aguiló J, Peiró S, Carbó J, Ferri R, Caro F, Ahmad M (2009)Open randomized clinical trial of laparoscopic versus open incisional hernia repair. SurgEndosc23: 1441–1448. **(1b)**
4. Barbaros U, Asogulu O, Seven R, Erbil Y, Dinccag A, Deveci U, Ozarmagan S, Mercan S (2006) The comparison of laparoscopic and open ventral hernia repairs: a prospective randomized study. Hernia11: 51–56. **(1b)**
5. Carbajo MA, Martín del Olmo JC, Blanco JI, de la Cuesta C, Toledano M, Martín F, Vaquero C, Inglada L (1999) Laparoscopic treatment vs open surgery in the solution of major incisional and abdominal wall hernias with mesh. SurgEndosc13: 250–2. **(1b)**
6. Itani KM, Hur K, Kim LT, Anthony T, Berger DH, Reda D, Neumayer L, for the Veternas Affairs Ventral Incisional Hernia Investigators.(2010) Comparison of laparoscopic and open repair with mesh for the treatment of ventral incisional hernia: a randomized trial. ArchSurg145: 322–8. **(1b)**
7. Misra MC, Bansal VK, Kulkarni MP, Pawar DK (2006) Comparison of laparoscopic and open repair of incisional and primary ventral hernia: results of a prospective randomized study. SurgEndosc20:1839–45. **(1b)**
8. Moreno-Egea A, Carrasco L, Girela E, Martín JG, Aguayo JL, Canteras M (2002) Open vs laparoscopic repair of spigelian hernia: a prospective randomized trial. Arch Surg137: 1266–8. **(1b)**
9. Navarra G, Musolino C, De Marco ML, Bartolotta M, Barbera A, Centorrino T (2007)Retromuscular sutured incisional hernia repair: a randomized controlled trial to compare open and laparoscopic approach. SurgLaparoscEndoscPercutan Tech17: 86–90. **(1b)**
10. Olmi S, Scaini A, Cesana GC, Erba L, Croce E (2007) Laparoscopic versus open incisional hernia repair: an open randomized controlled study.SurgEndosc21: 555–9. **(1b)**
11. Pring CM, Tran V, O’Rourke N, Martin IJ (2008) Laparoscopic versus open ventral hernia repair: a randomized controlled trial.ANZ J Surg78: 903–6**. (1b)**
12. [Forbes SS](http://www.ncbi.nlm.nih.gov/pubmed?term=%22Forbes%20SS%22%5BAuthor%5D), [Eskicioglu C](http://www.ncbi.nlm.nih.gov/pubmed?term=%22Eskicioglu%20C%22%5BAuthor%5D), [McLeod RS](http://www.ncbi.nlm.nih.gov/pubmed?term=%22McLeod%20RS%22%5BAuthor%5D), [Okrainec A](http://www.ncbi.nlm.nih.gov/pubmed?term=%22Okrainec%20A%22%5BAuthor%5D) (2009) Meta-analysis of randomized controlled trials comparing open and laparoscopic ventral and incisional hernia repair with mesh.[Br J Surg.](http://www.ncbi.nlm.nih.gov/pubmed?term=forbes%20incisional%20hernia)96: 851-8. **(1a)**
13. Sajid MS, Bokhari SA, Mallick AS, Cheek E, Baig MK (2009) Laparoscopic versus open repair of incisional/ventral hernia: a meta-analysis. Am J Surg197:64–72. **(1a)**
14. [Goodney PP](http://www.ncbi.nlm.nih.gov/pubmed?term=%22Goodney%20PP%22%5BAuthor%5D), [Birkmeyer CM](http://www.ncbi.nlm.nih.gov/pubmed?term=%22Birkmeyer%20CM%22%5BAuthor%5D), [Birkmeyer JD](http://www.ncbi.nlm.nih.gov/pubmed?term=%22Birkmeyer%20JD%22%5BAuthor%5D) (2002) Short-term outcomes of laparoscopic and open ventral hernia repair: a meta-analysis.[Arch Surg](http://www.ncbi.nlm.nih.gov/pubmed/12361426)137: 1161-5. **(2a)**
15. [Pham CT](http://www.ncbi.nlm.nih.gov/pubmed?term=%22Pham%20CT%22%5BAuthor%5D), [Perera CL](http://www.ncbi.nlm.nih.gov/pubmed?term=%22Perera%20CL%22%5BAuthor%5D), [Watkin DS](http://www.ncbi.nlm.nih.gov/pubmed?term=%22Watkin%20DS%22%5BAuthor%5D), [Maddern GJ](http://www.ncbi.nlm.nih.gov/pubmed?term=%22Maddern%20GJ%22%5BAuthor%5D) (2009) Laparoscopic ventral hernia repair: a systematic review.[SurgEndosc](http://www.ncbi.nlm.nih.gov/pubmed?term=pham%20laparoscopic%20ventral%20hernia)23: 4-15. **(2a)**
16. [Sains PS](http://www.ncbi.nlm.nih.gov/pubmed?term=Sains%20PS%5BAuthor%5D&cauthor=true&cauthor_uid=17058029), [Tilney HS](http://www.ncbi.nlm.nih.gov/pubmed?term=Tilney%20HS%5BAuthor%5D&cauthor=true&cauthor_uid=17058029), [Purkayastha S](http://www.ncbi.nlm.nih.gov/pubmed?term=Purkayastha%20S%5BAuthor%5D&cauthor=true&cauthor_uid=17058029), [Darzi AW](http://www.ncbi.nlm.nih.gov/pubmed?term=Darzi%20AW%5BAuthor%5D&cauthor=true&cauthor_uid=17058029), [Athanasiou T](http://www.ncbi.nlm.nih.gov/pubmed?term=Athanasiou%20T%5BAuthor%5D&cauthor=true&cauthor_uid=17058029), [Tekkis PP](http://www.ncbi.nlm.nih.gov/pubmed?term=Tekkis%20PP%5BAuthor%5D&cauthor=true&cauthor_uid=17058029), [Heriot AG](http://www.ncbi.nlm.nih.gov/pubmed?term=Heriot%20AG%5BAuthor%5D&cauthor=true&cauthor_uid=17058029) (2006) Outcomes following laparoscopic versus open repair of incisional hernia.[World J Surg](http://www.ncbi.nlm.nih.gov/pubmed?term=Sains%20PS%202006%20ventral%20hernia) 30: 2056-64. **(2a)**
17. Pierce RA, Spitler JA, Frisella MM, Matthews BD, Brunt LM (2007) Pooled data analysis of laparoscopic vs. open ventral hernia repair: 14 years of patient data accrual. SurgEndosc21: 378–86. **(2a)**
18. Park A, Birch DW, Lovrics P (1998)[Laparoscopic and open incisional hernia repair: a comparison study.](http://www.ncbi.nlm.nih.gov/pubmed/9781006)Surgery 124: 816-21. **(2b)**
19. [DeMaria EJ](http://www.ncbi.nlm.nih.gov/pubmed?term=%22DeMaria%20EJ%22%5BAuthor%5D), [Moss JM](http://www.ncbi.nlm.nih.gov/pubmed?term=%22Moss%20JM%22%5BAuthor%5D), [Sugerman HJ](http://www.ncbi.nlm.nih.gov/pubmed?term=%22Sugerman%20HJ%22%5BAuthor%5D) (2000)
20. Laparoscopic intraperitoneal polytetrafluoroethylene (PTFE) prosthetic patch repair of ventral hernia. Prospective comparison to open prefascial polypropylene mesh repair.[SurgEndosc](http://www.ncbi.nlm.nih.gov/pubmed?term=Demaria%20laparoscopic%20ventral%20hernia%202000)14: 326-9. **(2b)**
21. LomantoD, Iyer SG, Shabbir A, Cheah WK (2006) Laparoscopic versus open ventral hernia mesh repair: A prospective study. SurgEndosc20: 1030–5. **(2b)**
22. [Bingener J](http://www.ncbi.nlm.nih.gov/pubmed?term=Bingener%20J%5BAuthor%5D&cauthor=true&cauthor_uid=17576893), [Buck L](http://www.ncbi.nlm.nih.gov/pubmed?term=Buck%20L%5BAuthor%5D&cauthor=true&cauthor_uid=17576893), [Richards M](http://www.ncbi.nlm.nih.gov/pubmed?term=Richards%20M%5BAuthor%5D&cauthor=true&cauthor_uid=17576893), [Michalek J](http://www.ncbi.nlm.nih.gov/pubmed?term=Michalek%20J%5BAuthor%5D&cauthor=true&cauthor_uid=17576893), [Schwesinger W](http://www.ncbi.nlm.nih.gov/pubmed?term=Schwesinger%20W%5BAuthor%5D&cauthor=true&cauthor_uid=17576893), [Sirinek K](http://www.ncbi.nlm.nih.gov/pubmed?term=Sirinek%20K%5BAuthor%5D&cauthor=true&cauthor_uid=17576893) (2007) Long-term outcomes in laparoscopic vs open ventral hernia repair.[Arch Surg](http://www.ncbi.nlm.nih.gov/pubmed?term=Arch%20Surg.%202007%3B142%3A562-567) 142: 562-7. **(2b)**
23. Kurmann A, Visth E, Candinas D, Beldi G (2011) Long-term Follow-up of Open and Laparoscopic Repair of Large Incisional Hernias. World J Surg 35:297–301. **(2b)**
24. Holzman MD, Purut CM, Reintgen K, Eubanks S, Pappas TN (1997)[Laparoscopic ventral and incisional hernioplasty.](http://www.ncbi.nlm.nih.gov/pubmed/8994985) Surg Endosc 11:32-5. **(2b)**
25. [Ramshaw BJ](http://www.ncbi.nlm.nih.gov/pubmed?term=%22Ramshaw%20BJ%22%5BAuthor%5D), [Esartia P](http://www.ncbi.nlm.nih.gov/pubmed?term=%22Esartia%20P%22%5BAuthor%5D), [Schwab J](http://www.ncbi.nlm.nih.gov/pubmed?term=%22Schwab%20J%22%5BAuthor%5D), [Mason EM](http://www.ncbi.nlm.nih.gov/pubmed?term=%22Mason%20EM%22%5BAuthor%5D), [Wilson RA](http://www.ncbi.nlm.nih.gov/pubmed?term=%22Wilson%20RA%22%5BAuthor%5D), [Duncan TD](http://www.ncbi.nlm.nih.gov/pubmed?term=%22Duncan%20TD%22%5BAuthor%5D), [Miller J](http://www.ncbi.nlm.nih.gov/pubmed?term=%22Miller%20J%22%5BAuthor%5D), [Lucas GW](http://www.ncbi.nlm.nih.gov/pubmed?term=%22Lucas%20GW%22%5BAuthor%5D), [Promes J](http://www.ncbi.nlm.nih.gov/pubmed?term=%22Promes%20J%22%5BAuthor%5D) (1999) Comparison of laparoscopic and open ventral herniorrhaphy.[Am Surg](http://www.ncbi.nlm.nih.gov/pubmed/10484084) 65:827-31. **(3)**
26. [Chari R](http://www.ncbi.nlm.nih.gov/pubmed?term=%22Chari%20R%22%5BAuthor%5D), [Chari V](http://www.ncbi.nlm.nih.gov/pubmed?term=%22Chari%20V%22%5BAuthor%5D), [Eisenstat M](http://www.ncbi.nlm.nih.gov/pubmed?term=%22Eisenstat%20M%22%5BAuthor%5D), [Chung R](http://www.ncbi.nlm.nih.gov/pubmed?term=%22Chung%20R%22%5BAuthor%5D) (2000)A case controlled study of laparoscopic incisional hernia repair.[SurgEndosc](http://www.ncbi.nlm.nih.gov/pubmed?term=chari%20laparoscopic%20ventral%20hernia%202000) 14:117-9. **(3)**
27. [Zanghì A](http://www.ncbi.nlm.nih.gov/pubmed?term=Zangh%C3%AC%20A%5BAuthor%5D&cauthor=true&cauthor_uid=11347318), [Di Vita M](http://www.ncbi.nlm.nih.gov/pubmed?term=Di%20Vita%20M%5BAuthor%5D&cauthor=true&cauthor_uid=11347318), [Lomenzo E](http://www.ncbi.nlm.nih.gov/pubmed?term=Lomenzo%20E%5BAuthor%5D&cauthor=true&cauthor_uid=11347318), [De Luca A](http://www.ncbi.nlm.nih.gov/pubmed?term=De%20Luca%20A%5BAuthor%5D&cauthor=true&cauthor_uid=11347318), [Cappellani A](http://www.ncbi.nlm.nih.gov/pubmed?term=Cappellani%20A%5BAuthor%5D&cauthor=true&cauthor_uid=11347318) (2000)Laparoscopic repair vs open surgery for incisional hernias: a comparison study. [Ann Ital Chir](http://www.ncbi.nlm.nih.gov/pubmed/11347318) 71: 663-7. **(3)**
28. [Van't RM](http://www.ncbi.nlm.nih.gov/pubmed?term=%22van't%20RM%22%5BAuthor%5D), [Vrijland WW](http://www.ncbi.nlm.nih.gov/pubmed?term=%22Vrijland%20WW%22%5BAuthor%5D), [Lange JF](http://www.ncbi.nlm.nih.gov/pubmed?term=%22Lange%20JF%22%5BAuthor%5D), [Hop WC](http://www.ncbi.nlm.nih.gov/pubmed?term=%22Hop%20WC%22%5BAuthor%5D), [Jeekel J](http://www.ncbi.nlm.nih.gov/pubmed?term=%22Jeekel%20J%22%5BAuthor%5D), [Bonjer HJ](http://www.ncbi.nlm.nih.gov/pubmed?term=%22Bonjer%20HJ%22%5BAuthor%5D) (2002) Mesh repair of incisional hernia: comparison of laparoscopic and open repair.[Eur J Surg](http://www.ncbi.nlm.nih.gov/pubmed?term=Van't%20laparoscopic%20ventral%20hernia%202002)168: 684-9. **(3)**
29. [Raftopoulos I](http://www.ncbi.nlm.nih.gov/pubmed?term=Raftopoulos%20I%5BAuthor%5D&cauthor=true&cauthor_uid=14558710), [Vanuno D](http://www.ncbi.nlm.nih.gov/pubmed?term=Vanuno%20D%5BAuthor%5D&cauthor=true&cauthor_uid=14558710), [Khorsand J](http://www.ncbi.nlm.nih.gov/pubmed?term=Khorsand%20J%5BAuthor%5D&cauthor=true&cauthor_uid=14558710), [Kouraklis G](http://www.ncbi.nlm.nih.gov/pubmed?term=Kouraklis%20G%5BAuthor%5D&cauthor=true&cauthor_uid=14558710), [Lasky P](http://www.ncbi.nlm.nih.gov/pubmed?term=Lasky%20P%5BAuthor%5D&cauthor=true&cauthor_uid=14558710) (2003) Comparison of open and laparoscopic prosthetic repair of large ventral hernias.[JSLS](http://www.ncbi.nlm.nih.gov/pubmed/14558710)7: 227-32. **(3)**
30. [Bencini L](http://www.ncbi.nlm.nih.gov/pubmed?term=Bencini%20L%5BAuthor%5D&cauthor=true&cauthor_uid=12915971), [Sanchez LJ](http://www.ncbi.nlm.nih.gov/pubmed?term=Sanchez%20LJ%5BAuthor%5D&cauthor=true&cauthor_uid=12915971), [Boffi B](http://www.ncbi.nlm.nih.gov/pubmed?term=Boffi%20B%5BAuthor%5D&cauthor=true&cauthor_uid=12915971), [Farsi M](http://www.ncbi.nlm.nih.gov/pubmed?term=Farsi%20M%5BAuthor%5D&cauthor=true&cauthor_uid=12915971), [Scatizzi M](http://www.ncbi.nlm.nih.gov/pubmed?term=Scatizzi%20M%5BAuthor%5D&cauthor=true&cauthor_uid=12915971), [Moretti R](http://www.ncbi.nlm.nih.gov/pubmed?term=Moretti%20R%5BAuthor%5D&cauthor=true&cauthor_uid=12915971) (2003) Incisional hernia: repair retrospective comparison of laparoscopic and open techniques.[SurgEndosc](http://www.ncbi.nlm.nih.gov/pubmed/12915971) 17: 1546-51. **(3)**
31. [Olmi S](http://www.ncbi.nlm.nih.gov/pubmed?term=%22Olmi%20S%22%5BAuthor%5D), [Magnone S](http://www.ncbi.nlm.nih.gov/pubmed?term=%22Magnone%20S%22%5BAuthor%5D), [Erba L](http://www.ncbi.nlm.nih.gov/pubmed?term=%22Erba%20L%22%5BAuthor%5D), [Bertolini A](http://www.ncbi.nlm.nih.gov/pubmed?term=%22Bertolini%20A%22%5BAuthor%5D), [Croce E](http://www.ncbi.nlm.nih.gov/pubmed?term=%22Croce%20E%22%5BAuthor%5D) (2005)

Results of laparoscopic versus open abdominal and incisional hernia repair.[JSLS](http://www.ncbi.nlm.nih.gov/pubmed/15984708)9: 189-95. **(3)**

32. [Beldi G](http://www.ncbi.nlm.nih.gov/pubmed?term=%22Beldi%20G%22%5BAuthor%5D), [Ipaktchi R](http://www.ncbi.nlm.nih.gov/pubmed?term=%22Ipaktchi%20R%22%5BAuthor%5D), [Wagner M](http://www.ncbi.nlm.nih.gov/pubmed?term=%22Wagner%20M%22%5BAuthor%5D), [Gloor B](http://www.ncbi.nlm.nih.gov/pubmed?term=%22Gloor%20B%22%5BAuthor%5D), [Candinas D](http://www.ncbi.nlm.nih.gov/pubmed?term=%22Candinas%20D%22%5BAuthor%5D) (2006) Laparoscopic ventral hernia repair is safe and cost effective.[SurgEndosc](http://www.ncbi.nlm.nih.gov/pubmed/16333538) 20:92-5. **(3)**
33. [Zografos GN](http://www.ncbi.nlm.nih.gov/pubmed?term=%22Zografos%20GN%22%5BAuthor%5D), [Mitropapas G](http://www.ncbi.nlm.nih.gov/pubmed?term=%22Mitropapas%20G%22%5BAuthor%5D), [Vasiliadis G](http://www.ncbi.nlm.nih.gov/pubmed?term=%22Vasiliadis%20G%22%5BAuthor%5D), [Farfaras A](http://www.ncbi.nlm.nih.gov/pubmed?term=%22Farfaras%20A%22%5BAuthor%5D), [Ageli C](http://www.ncbi.nlm.nih.gov/pubmed?term=%22Ageli%20C%22%5BAuthor%5D), [Margaris E](http://www.ncbi.nlm.nih.gov/pubmed?term=%22Margaris%20E%22%5BAuthor%5D), [Tsipras I](http://www.ncbi.nlm.nih.gov/pubmed?term=%22Tsipras%20I%22%5BAuthor%5D), [Koliopanos A](http://www.ncbi.nlm.nih.gov/pubmed?term=%22Koliopanos%20A%22%5BAuthor%5D), [Pateras J](http://www.ncbi.nlm.nih.gov/pubmed?term=%22Pateras%20J%22%5BAuthor%5D), [Papastratis G](http://www.ncbi.nlm.nih.gov/pubmed?term=%22Papastratis%20G%22%5BAuthor%5D) (2007)Open and laparoscopic approach in incisional hernia repair with ePTFE prosthesis.[J Laparoendosc Adv Surg Tech A](http://www.ncbi.nlm.nih.gov/pubmed?term=zografos%20laparoscopic%20incisional%20hernia%20repair)  17: 277-81. **(3)**
34. [Brown RB](http://www.ncbi.nlm.nih.gov/pubmed?term=Brown%20RB%5BAuthor%5D&cauthor=true&cauthor_uid=8180760).Laparoscopic hernia repair: a rural perspective (1994) [SurgLaparoscEndosc](http://www.ncbi.nlm.nih.gov/pubmed?term=brown%20rb%20incisional%20hernia)4: 106-9. **(4)**
35. Franklin ME Jr, Dorman JP, Glass JL, Balli JE, Gonzalez JJ Jr (1998) Laparoscopic ventral and incisional hernia repair.SurgLaparoscEndosc 8: 294 – 9. **(4)**
36. [Tsimoyiannis EC](http://www.ncbi.nlm.nih.gov/pubmed?term=Tsimoyiannis%20EC%5BAuthor%5D&cauthor=true&cauthor_uid=9799145), [Tassis A](http://www.ncbi.nlm.nih.gov/pubmed?term=Tassis%20A%5BAuthor%5D&cauthor=true&cauthor_uid=9799145), [Glantzounis G](http://www.ncbi.nlm.nih.gov/pubmed?term=Glantzounis%20G%5BAuthor%5D&cauthor=true&cauthor_uid=9799145), [Jabarin M](http://www.ncbi.nlm.nih.gov/pubmed?term=Jabarin%20M%5BAuthor%5D&cauthor=true&cauthor_uid=9799145), [Siakas P](http://www.ncbi.nlm.nih.gov/pubmed?term=Siakas%20P%5BAuthor%5D&cauthor=true&cauthor_uid=9799145), [Tzourou H](http://www.ncbi.nlm.nih.gov/pubmed?term=Tzourou%20H%5BAuthor%5D&cauthor=true&cauthor_uid=9799145) (1998) Laparoscopic intraperitonealonlay mesh repair of incisional hernia.[SurgLaparoscEndosc](http://www.ncbi.nlm.nih.gov/pubmed/9799145) 8: 360-2. **(4)**
37. [Sanders LM](http://www.ncbi.nlm.nih.gov/pubmed?term=Sanders%20LM%5BAuthor%5D&cauthor=true&cauthor_uid=10219859), [Flint LM](http://www.ncbi.nlm.nih.gov/pubmed?term=Flint%20LM%5BAuthor%5D&cauthor=true&cauthor_uid=10219859), [Ferrara JJ](http://www.ncbi.nlm.nih.gov/pubmed?term=Ferrara%20JJ%5BAuthor%5D&cauthor=true&cauthor_uid=10219859) (1999) Initial experience with laparoscopic repair of incisional hernias.[Am J Surg](http://www.ncbi.nlm.nih.gov/pubmed/10219859) 177: 227-31. **(4)**
38. [Heniford BT](http://www.ncbi.nlm.nih.gov/pubmed?term=%22Heniford%20BT%22%5BAuthor%5D), [Ramshaw BJ](http://www.ncbi.nlm.nih.gov/pubmed?term=%22Ramshaw%20BJ%22%5BAuthor%5D) (2000)Laparoscopic ventral hernia repair: a report of 100 consecutive cases.[SurgEndosc](http://www.ncbi.nlm.nih.gov/pubmed/10858463)14:419-23. **(4)**
39. [LeBlanc KA](http://www.ncbi.nlm.nih.gov/pubmed?term=LeBlanc%20KA%5BAuthor%5D&cauthor=true&cauthor_uid=11387722), [Booth WV](http://www.ncbi.nlm.nih.gov/pubmed?term=Booth%20WV%5BAuthor%5D&cauthor=true&cauthor_uid=11387722), [Whitaker JM](http://www.ncbi.nlm.nih.gov/pubmed?term=Whitaker%20JM%5BAuthor%5D&cauthor=true&cauthor_uid=11387722), [Bellanger DE](http://www.ncbi.nlm.nih.gov/pubmed?term=Bellanger%20DE%5BAuthor%5D&cauthor=true&cauthor_uid=11387722) (2001) Laparoscopic incisional and ventral herniorraphy: our initial 100 patients.[Hernia](http://www.ncbi.nlm.nih.gov/pubmed/11387722) 5: 41-5. **(4)**
40. Moreno-Egea A, Castillo J, Girela E, Canteras M, Aguayo JL (2002) Outpatient laparoscopic incisional/ ventral hernioplasty: our experience in 55 cases. SurgLaparoscEndoscPercutan Tech 12:171-174. **(4)**
41. [Kua KB](http://www.ncbi.nlm.nih.gov/pubmed?term=%22Kua%20KB%22%5BAuthor%5D), [Coleman M](http://www.ncbi.nlm.nih.gov/pubmed?term=%22Coleman%20M%22%5BAuthor%5D), [Martin I](http://www.ncbi.nlm.nih.gov/pubmed?term=%22Martin%20I%22%5BAuthor%5D), [O'Rourke N](http://www.ncbi.nlm.nih.gov/pubmed?term=%22O'Rourke%20N%22%5BAuthor%5D) (2002)

Laparoscopic repair of ventral incisional hernia.[ANZ J Surg](http://www.ncbi.nlm.nih.gov/pubmed?term=kua%20laparoscopic%20ventral%20hernia) 72:296-9. **(4)**

42. [Raftopoulos I](http://www.ncbi.nlm.nih.gov/pubmed?term=Raftopoulos%20I%5BAuthor%5D&cauthor=true&cauthor_uid=12590723), [Vanuno D](http://www.ncbi.nlm.nih.gov/pubmed?term=Vanuno%20D%5BAuthor%5D&cauthor=true&cauthor_uid=12590723), [Khorsand J](http://www.ncbi.nlm.nih.gov/pubmed?term=Khorsand%20J%5BAuthor%5D&cauthor=true&cauthor_uid=12590723), [Ninos J](http://www.ncbi.nlm.nih.gov/pubmed?term=Ninos%20J%5BAuthor%5D&cauthor=true&cauthor_uid=12590723), [Kouraklis G](http://www.ncbi.nlm.nih.gov/pubmed?term=Kouraklis%20G%5BAuthor%5D&cauthor=true&cauthor_uid=12590723), [Lasky P](http://www.ncbi.nlm.nih.gov/pubmed?term=Lasky%20P%5BAuthor%5D&cauthor=true&cauthor_uid=12590723) (2002) Outcome of laparoscopic ventral hernia repair in correlation with obesity, type of hernia, and hernia size.[J Laparoendosc Adv Surg Tech A](http://www.ncbi.nlm.nih.gov/pubmed/12590723) 12: 425-9. **(4)**
43. [Varghese TK](http://www.ncbi.nlm.nih.gov/pubmed?term=Varghese%20TK%5BAuthor%5D&cauthor=true&cauthor_uid=12121696), [Denham DW](http://www.ncbi.nlm.nih.gov/pubmed?term=Denham%20DW%5BAuthor%5D&cauthor=true&cauthor_uid=12121696), [Dawes LG](http://www.ncbi.nlm.nih.gov/pubmed?term=Dawes%20LG%5BAuthor%5D&cauthor=true&cauthor_uid=12121696), [Murayama KM](http://www.ncbi.nlm.nih.gov/pubmed?term=Murayama%20KM%5BAuthor%5D&cauthor=true&cauthor_uid=12121696), [Prystowsky JB](http://www.ncbi.nlm.nih.gov/pubmed?term=Prystowsky%20JB%5BAuthor%5D&cauthor=true&cauthor_uid=12121696), [Joehl RJ](http://www.ncbi.nlm.nih.gov/pubmed?term=Joehl%20RJ%5BAuthor%5D&cauthor=true&cauthor_uid=12121696) (2002) Laparoscopic ventral hernia repair: an initial institutional experience.[J Surg Res](http://www.ncbi.nlm.nih.gov/pubmed/12121696) 105: 115-8. **(4)**
44. Berger D, Bientzle M, Muller A (2002) Postoperative complications after laparoscopic incisional hernia repair. Incidence and treatment. SurgEndosc 16: 1720–1723(4)
45. Kirshtein B, Lantsberg L, Avinoach E, Bayme M, Mizrahi S (2002) Laparoscopic repair of large incisional hernias. SurgEndosc 16: 1717–1719.
46. [Aura T](http://www.ncbi.nlm.nih.gov/pubmed?term=%22Aura%20T%22%5BAuthor%5D), [Habib E](http://www.ncbi.nlm.nih.gov/pubmed?term=%22Habib%20E%22%5BAuthor%5D), [Mekkaoui M](http://www.ncbi.nlm.nih.gov/pubmed?term=%22Mekkaoui%20M%22%5BAuthor%5D), [Brassier D](http://www.ncbi.nlm.nih.gov/pubmed?term=%22Brassier%20D%22%5BAuthor%5D), [Elhadad A](http://www.ncbi.nlm.nih.gov/pubmed?term=%22Elhadad%20A%22%5BAuthor%5D) (2002)Laparoscopic tension-free repair of anterior abdominal wall incisional and ventral hernias with an intraperitoneal Gore-Tex mesh: prospective study and review of the literature. [J Laparoendosc Adv Surg Tech A](http://www.ncbi.nlm.nih.gov/pubmed/12269494) 12:263-7**. (4)**
47. [Chowbey PK](http://www.ncbi.nlm.nih.gov/pubmed?term=Chowbey%20PK%5BAuthor%5D&cauthor=true&cauthor_uid=12709615), [Sharma A](http://www.ncbi.nlm.nih.gov/pubmed?term=Sharma%20A%5BAuthor%5D&cauthor=true&cauthor_uid=12709615), [Khullar R](http://www.ncbi.nlm.nih.gov/pubmed?term=Khullar%20R%5BAuthor%5D&cauthor=true&cauthor_uid=12709615), [Soni V](http://www.ncbi.nlm.nih.gov/pubmed?term=Soni%20V%5BAuthor%5D&cauthor=true&cauthor_uid=12709615), [Baijal M](http://www.ncbi.nlm.nih.gov/pubmed?term=Baijal%20M%5BAuthor%5D&cauthor=true&cauthor_uid=12709615) (2003) Laparoscopic ventral hernia repair with extraperitoneal mesh: surgical technique and early results.[SurgLaparoscEndoscPercutan Tech](http://www.ncbi.nlm.nih.gov/pubmed?term=Chowbey%202003%20incisional%20hernia) 13: 101-5. **(4)**
48. LeBlanc KA, Whitaker JM, Bellanger DE, Rhynes VK (2003) Laparoscopic incisional and ventral hernioplasty: lessons learned from 200 patients. Hernia 7: 118–124. **(4)**
49. Carbajo MA, Martp del Olmo JC, Blanco JI, Toledano M, de la Cuesta C, Ferreras C, Vaquero C (2003) Laparoscopic approach to incisional hernia. SurgEndosc 17:118-22. **(4)**
50. Heniford BT, Park A, Ramshaw BJ, Voeller G (2000) Laparoscopic ventral and incisional hernia repair in 407 patients. J Am CollSurg 190:645-50. **(4)**
51. Eid GM, Prince JM, Mattar SG, Hamad G, Ikrammudin S, Schauer PR (2003) Medium-term follow-up confirms the safety and durability of laparoscopic ventral hernia repair with PTFE. Surgery134:599-603. **(4)**
52. [Tagaya N](http://www.ncbi.nlm.nih.gov/pubmed?term=Tagaya%20N%5BAuthor%5D&cauthor=true&cauthor_uid=15259577), [Mikami H](http://www.ncbi.nlm.nih.gov/pubmed?term=Mikami%20H%5BAuthor%5D&cauthor=true&cauthor_uid=15259577), [Aoki H](http://www.ncbi.nlm.nih.gov/pubmed?term=Aoki%20H%5BAuthor%5D&cauthor=true&cauthor_uid=15259577), [Kubota K](http://www.ncbi.nlm.nih.gov/pubmed?term=Kubota%20K%5BAuthor%5D&cauthor=true&cauthor_uid=15259577)(2004)Long-term complications of laparoscopic ventral and incisional hernia repair. [SurgLaparoscEndoscPercutan Tech](http://www.ncbi.nlm.nih.gov/pubmed/15259577) 14: 5-8. **(4)**
53. Ujiki MB, Weinberger J, Varghese TK, Murayama KM, Joehl RJ (2004) One hundred consecutive laparoscopic ventral hernia repairs. Am J Surg 188: 593–597. **(4)**
54. Franklin ME Jr, Gonzalez JJ Jr, Glass JL, Manjarrez A (2004) Laparoscopic ventral and incisional hernia repair: an 11-year experience. Hernia 8: 23-7. **(4)**
55. [Bamehriz F](http://www.ncbi.nlm.nih.gov/pubmed?term=Bamehriz%20F%5BAuthor%5D&cauthor=true&cauthor_uid=15472549), [Birch DW](http://www.ncbi.nlm.nih.gov/pubmed?term=Birch%20DW%5BAuthor%5D&cauthor=true&cauthor_uid=15472549)(2004)The feasibility of adopting laparoscopic incisionalhernia repair in general surgery practice: early outcomes in an unselected series of patients. [SurgLaparoscEndoscPercutan Tech](http://www.ncbi.nlm.nih.gov/pubmed?term=bamehriz%20incisional%20hernia) 14: 207-9. **(4)**
56. [Perrone JM](http://www.ncbi.nlm.nih.gov/pubmed?term=%22Perrone%20JM%22%5BAuthor%5D), [Soper NJ](http://www.ncbi.nlm.nih.gov/pubmed?term=%22Soper%20NJ%22%5BAuthor%5D), [Eagon JC](http://www.ncbi.nlm.nih.gov/pubmed?term=%22Eagon%20JC%22%5BAuthor%5D), [Klingensmith ME](http://www.ncbi.nlm.nih.gov/pubmed?term=%22Klingensmith%20ME%22%5BAuthor%5D), [Aft RL](http://www.ncbi.nlm.nih.gov/pubmed?term=%22Aft%20RL%22%5BAuthor%5D), [Frisella MM](http://www.ncbi.nlm.nih.gov/pubmed?term=%22Frisella%20MM%22%5BAuthor%5D), [Brunt LM](http://www.ncbi.nlm.nih.gov/pubmed?term=%22Brunt%20LM%22%5BAuthor%5D) (2005) Perioperative outcomes and complications of laparoscopic ventral hernia repair.[Surgery](http://www.ncbi.nlm.nih.gov/pubmed?term=perrone%202005%20incisional%20hernia) 138:708-15. **(4)**
57. [Motson RW](http://www.ncbi.nlm.nih.gov/pubmed?term=Motson%20RW%5BAuthor%5D&cauthor=true&cauthor_uid=17048281), [Engledow AH](http://www.ncbi.nlm.nih.gov/pubmed?term=Engledow%20AH%5BAuthor%5D&cauthor=true&cauthor_uid=17048281), [Medhurst C](http://www.ncbi.nlm.nih.gov/pubmed?term=Medhurst%20C%5BAuthor%5D&cauthor=true&cauthor_uid=17048281), [Adib R](http://www.ncbi.nlm.nih.gov/pubmed?term=Adib%20R%5BAuthor%5D&cauthor=true&cauthor_uid=17048281), [Warren SJ](http://www.ncbi.nlm.nih.gov/pubmed?term=Warren%20SJ%5BAuthor%5D&cauthor=true&cauthor_uid=17048281) (2006)Laparoscopic incisionalhernia repair with a self-centring suture. [Br J Surg](http://www.ncbi.nlm.nih.gov/pubmed/17048281) 93: 1549-53. **(4)**
58. Chelala E, Thoma M, TateteB, Lemye AC, Dessily M, Alle JL (2007) The suturing concept for laparoscopic mesh fixation in ventral and incisional hernia repair: Mid-term analysis of 400 cases. SurgEndosc 21:391-5. **(4)**
59. [Raftopoulos I](http://www.ncbi.nlm.nih.gov/pubmed?term=Raftopoulos%20I%5BAuthor%5D&cauthor=true&cauthor_uid=17522922), [Courcoulas AP](http://www.ncbi.nlm.nih.gov/pubmed?term=Courcoulas%20AP%5BAuthor%5D&cauthor=true&cauthor_uid=17522922) (2007)Outcome of laparoscopic ventral hernia repair in morbidly obese patients with a body mass index exceeding 35 kg/m2. [SurgEndosc](http://www.ncbi.nlm.nih.gov/pubmed/17522922) 21: 2293-7. **(4)**
60. Wassenaar EB, Raymakers JTFJ, Rakic S (2008) Impact of the mesh fixation technique on operative time in laparoscopic repair of ventral hernias. Hernia 12: 23-25. **(4)**
61. [Berrevoet F](http://www.ncbi.nlm.nih.gov/pubmed?term=%22Berrevoet%20F%22%5BAuthor%5D), [Fierens K](http://www.ncbi.nlm.nih.gov/pubmed?term=%22Fierens%20K%22%5BAuthor%5D), [De Gols J](http://www.ncbi.nlm.nih.gov/pubmed?term=%22De%20Gols%20J%22%5BAuthor%5D), [Navez B](http://www.ncbi.nlm.nih.gov/pubmed?term=%22Navez%20B%22%5BAuthor%5D), [Van Bastelaere W](http://www.ncbi.nlm.nih.gov/pubmed?term=%22Van%20Bastelaere%20W%22%5BAuthor%5D), [Meir E](http://www.ncbi.nlm.nih.gov/pubmed?term=%22Meir%20E%22%5BAuthor%5D), [Ceulemans R](http://www.ncbi.nlm.nih.gov/pubmed?term=%22Ceulemans%20R%22%5BAuthor%5D) (2009)Multicentric observational cohort study evaluating a composite mesh with incorporated oxidized regenerated cellulose in laparoscopic ventral hernia repair. [Hernia](http://www.ncbi.nlm.nih.gov/pubmed/18682886) 13:23-7.**(4)**
62. Baccari P, Nifosi J, Ghirardelli L, Staudacher C (2009) Laparoscopic incisional and ventral hernia repair without sutures: a single-center experience with 200 cases. J Laparoendosc Adv Surg Tech A 19:175-9. **(4)**
63. [Edwards C](http://www.ncbi.nlm.nih.gov/pubmed?term=Edwards%20C%5BAuthor%5D&cauthor=true&cauthor_uid=19462203), [Geiger T](http://www.ncbi.nlm.nih.gov/pubmed?term=Geiger%20T%5BAuthor%5D&cauthor=true&cauthor_uid=19462203), [Bartow K](http://www.ncbi.nlm.nih.gov/pubmed?term=Bartow%20K%5BAuthor%5D&cauthor=true&cauthor_uid=19462203), [Ramaswamy A](http://www.ncbi.nlm.nih.gov/pubmed?term=Ramaswamy%20A%5BAuthor%5D&cauthor=true&cauthor_uid=19462203), [Fearing N](http://www.ncbi.nlm.nih.gov/pubmed?term=Fearing%20N%5BAuthor%5D&cauthor=true&cauthor_uid=19462203), [Thaler K](http://www.ncbi.nlm.nih.gov/pubmed?term=Thaler%20K%5BAuthor%5D&cauthor=true&cauthor_uid=19462203), [Ramshaw B](http://www.ncbi.nlm.nih.gov/pubmed?term=Ramshaw%20B%5BAuthor%5D&cauthor=true&cauthor_uid=19462203) (2009) Laparoscopic transperitoneal repair of flank hernias: a retrospective review of 27 patients.[SurgEndosc](http://www.ncbi.nlm.nih.gov/pubmed?term=Laparoscopic%20transperitoneal%20repair%20of%20flank%20hernias%3A%20a%20retrospective%20review%20of%2027%20patients) 23: 2692-6. **(4)**
64. [Moreno-Egea A](http://www.ncbi.nlm.nih.gov/pubmed?term=Moreno-Egea%20A%5BAuthor%5D&cauthor=true&cauthor_uid=18574413), [Cartagena J](http://www.ncbi.nlm.nih.gov/pubmed?term=Cartagena%20J%5BAuthor%5D&cauthor=true&cauthor_uid=18574413), [Vicente JP](http://www.ncbi.nlm.nih.gov/pubmed?term=Vicente%20JP%5BAuthor%5D&cauthor=true&cauthor_uid=18574413), [Carrillo A](http://www.ncbi.nlm.nih.gov/pubmed?term=Carrillo%20A%5BAuthor%5D&cauthor=true&cauthor_uid=18574413), [Aguayo JL](http://www.ncbi.nlm.nih.gov/pubmed?term=Aguayo%20JL%5BAuthor%5D&cauthor=true&cauthor_uid=18574413) (2008)Laparoscopic incisionalhernia repair as a day surgery procedure: audit of 127 consecutive cases in a university hospital. [SurgLaparoscEndoscPercutan Tech](http://www.ncbi.nlm.nih.gov/pubmed/18574413) 18: 267-71. **(4)**
65. [Olmi S](http://www.ncbi.nlm.nih.gov/pubmed?term=Olmi%20S%5BAuthor%5D&cauthor=true&cauthor_uid=20932376), [Cesana G](http://www.ncbi.nlm.nih.gov/pubmed?term=Cesana%20G%5BAuthor%5D&cauthor=true&cauthor_uid=20932376), [Sagutti L](http://www.ncbi.nlm.nih.gov/pubmed?term=Sagutti%20L%5BAuthor%5D&cauthor=true&cauthor_uid=20932376), [Pagano C](http://www.ncbi.nlm.nih.gov/pubmed?term=Pagano%20C%5BAuthor%5D&cauthor=true&cauthor_uid=20932376), [Vittoria G](http://www.ncbi.nlm.nih.gov/pubmed?term=Vittoria%20G%5BAuthor%5D&cauthor=true&cauthor_uid=20932376), [Croce E](http://www.ncbi.nlm.nih.gov/pubmed?term=Croce%20E%5BAuthor%5D&cauthor=true&cauthor_uid=20932376) (2010)Laparoscopic incisionalhernia repair with fibrin glue in select patients. [JSLS](http://www.ncbi.nlm.nih.gov/pubmed/20932376) 14: 240-5. **(4)**
66. Sharma A, Mehrotra M, Khullar R, Soni V, Baijal M, Chowbey PK (2011) Laparoscopic ventral/incisional hernia repair: a single centre experience of 1,242 patients over a period of 13 years. Hernia 15:131-9. **(4)**
67. SturtNJH,Christopher CL, Engledow AH, Menzies D, Motson R (2011) Results of Laparoscopic Repair of Primary and Recurrent Incisional Hernias at a Single UK Institution. SurgLaparoscEndoscPercutan Tech 21:86–89. **(4)**
68. [Engledow AH](http://www.ncbi.nlm.nih.gov/pubmed?term=Engledow%20AH%5BAuthor%5D&cauthor=true&cauthor_uid=17111283), [Sengupta N](http://www.ncbi.nlm.nih.gov/pubmed?term=Sengupta%20N%5BAuthor%5D&cauthor=true&cauthor_uid=17111283), [Akhras F](http://www.ncbi.nlm.nih.gov/pubmed?term=Akhras%20F%5BAuthor%5D&cauthor=true&cauthor_uid=17111283), [Tutton M](http://www.ncbi.nlm.nih.gov/pubmed?term=Tutton%20M%5BAuthor%5D&cauthor=true&cauthor_uid=17111283), [Warren SJ](http://www.ncbi.nlm.nih.gov/pubmed?term=Warren%20SJ%5BAuthor%5D&cauthor=true&cauthor_uid=17111283) (2007) Day case laparoscopic incisional hernia repair is feasible, acceptable, and cost effective.[SurgEndosc](http://www.ncbi.nlm.nih.gov/pubmed/17111283) 21:84-6. **(4)**
69. [Eriksen JR](http://www.ncbi.nlm.nih.gov/pubmed?term=Eriksen%20JR%5BAuthor%5D&cauthor=true&cauthor_uid=18670733), [Poornoroozy P](http://www.ncbi.nlm.nih.gov/pubmed?term=Poornoroozy%20P%5BAuthor%5D&cauthor=true&cauthor_uid=18670733), [Jørgensen LN](http://www.ncbi.nlm.nih.gov/pubmed?term=J%C3%B8rgensen%20LN%5BAuthor%5D&cauthor=true&cauthor_uid=18670733), [Jacobsen B](http://www.ncbi.nlm.nih.gov/pubmed?term=Jacobsen%20B%5BAuthor%5D&cauthor=true&cauthor_uid=18670733), [Friis-Andersen HU](http://www.ncbi.nlm.nih.gov/pubmed?term=Friis-Andersen%20HU%5BAuthor%5D&cauthor=true&cauthor_uid=18670733), [Rosenberg J](http://www.ncbi.nlm.nih.gov/pubmed?term=Rosenberg%20J%5BAuthor%5D&cauthor=true&cauthor_uid=18670733) (2009) Pain, quality of life and recovery after laparoscopic ventral hernia repair.[Hernia](http://www.ncbi.nlm.nih.gov/pubmed/18670733) 13: 13-21. **(4)**
70. [Nguyen SQ](http://www.ncbi.nlm.nih.gov/pubmed?term=%22Nguyen%20SQ%22%5BAuthor%5D), [Divino CM](http://www.ncbi.nlm.nih.gov/pubmed?term=%22Divino%20CM%22%5BAuthor%5D), [Buch KE](http://www.ncbi.nlm.nih.gov/pubmed?term=%22Buch%20KE%22%5BAuthor%5D), [Schnur J](http://www.ncbi.nlm.nih.gov/pubmed?term=%22Schnur%20J%22%5BAuthor%5D), [Weber KJ](http://www.ncbi.nlm.nih.gov/pubmed?term=%22Weber%20KJ%22%5BAuthor%5D), [Katz LB](http://www.ncbi.nlm.nih.gov/pubmed?term=%22Katz%20LB%22%5BAuthor%5D), [Reiner MA](http://www.ncbi.nlm.nih.gov/pubmed?term=%22Reiner%20MA%22%5BAuthor%5D), [Aldoroty RA](http://www.ncbi.nlm.nih.gov/pubmed?term=%22Aldoroty%20RA%22%5BAuthor%5D), [Herron DM](http://www.ncbi.nlm.nih.gov/pubmed?term=%22Herron%20DM%22%5BAuthor%5D) (2008) Postoperative pain after laparoscopic ventral hernia repair: a prospective comparison of sutures versus tacks.[JSLS](http://www.ncbi.nlm.nih.gov/pubmed/18435881) 12: 113-6. **(2b)**
71. [Earle D](http://www.ncbi.nlm.nih.gov/pubmed?term=Earle%20D%5BAuthor%5D&cauthor=true&cauthor_uid=16333530), [Seymour N](http://www.ncbi.nlm.nih.gov/pubmed?term=Seymour%20N%5BAuthor%5D&cauthor=true&cauthor_uid=16333530), [Fellinger E](http://www.ncbi.nlm.nih.gov/pubmed?term=Fellinger%20E%5BAuthor%5D&cauthor=true&cauthor_uid=16333530), [Perez A](http://www.ncbi.nlm.nih.gov/pubmed?term=Perez%20A%5BAuthor%5D&cauthor=true&cauthor_uid=16333530) (2006) Laparoscopic versus open incisional hernia repair: a single-institution analysis of hospital resource utilization for 884 consecutive cases.[SurgEndosc](http://www.ncbi.nlm.nih.gov/pubmed/16333530) 20:71-5**. (3)**
72. [Wright BE](http://www.ncbi.nlm.nih.gov/pubmed?term=Wright%20BE%5BAuthor%5D&cauthor=true&cauthor_uid=11893110), [Niskanen BD](http://www.ncbi.nlm.nih.gov/pubmed?term=Niskanen%20BD%5BAuthor%5D&cauthor=true&cauthor_uid=11893110), [Peterson DJ](http://www.ncbi.nlm.nih.gov/pubmed?term=Peterson%20DJ%5BAuthor%5D&cauthor=true&cauthor_uid=11893110), [Ney AL](http://www.ncbi.nlm.nih.gov/pubmed?term=Ney%20AL%5BAuthor%5D&cauthor=true&cauthor_uid=11893110), [Odland MD](http://www.ncbi.nlm.nih.gov/pubmed?term=Odland%20MD%5BAuthor%5D&cauthor=true&cauthor_uid=11893110), [VanCamp J](http://www.ncbi.nlm.nih.gov/pubmed?term=VanCamp%20J%5BAuthor%5D&cauthor=true&cauthor_uid=11893110), [Zera RT](http://www.ncbi.nlm.nih.gov/pubmed?term=Zera%20RT%5BAuthor%5D&cauthor=true&cauthor_uid=11893110), [Rodriguez JL](http://www.ncbi.nlm.nih.gov/pubmed?term=Rodriguez%20JL%5BAuthor%5D&cauthor=true&cauthor_uid=11893110) (2002) Laparoscopic ventral hernia repair: are there comparative advantages over traditional methods of repair?[Am Surg](http://www.ncbi.nlm.nih.gov/pubmed/11893110) 68:291-5. **(3)**
73. Bansal VK, Misra MC, Kumar S, Rao YK, Singhal P, Goswami A, Guleria S, Arora MK, Chabra A (2011) A prospective randomized study comparing suture mesh fixation versus tacker mesh fixation for laparoscopic repair of incisional and ventral hernias. SurgEndosc25: 1431-8. **(1b)**
74. [Alkhoury F](http://www.ncbi.nlm.nih.gov/pubmed?term=Alkhoury%20F%5BAuthor%5D&cauthor=true&cauthor_uid=21471797), [Helton S](http://www.ncbi.nlm.nih.gov/pubmed?term=Helton%20S%5BAuthor%5D&cauthor=true&cauthor_uid=21471797), [Ippolito RJ](http://www.ncbi.nlm.nih.gov/pubmed?term=Ippolito%20RJ%5BAuthor%5D&cauthor=true&cauthor_uid=21471797) (2011) Cost and clinical outcomes of laparoscopic ventral hernia repair using intraperitonealnonheavyweight polypropylene mesh.[SurgLaparoscEndoscPercutan Tech](http://www.ncbi.nlm.nih.gov/pubmed/21471797) 21: 82-5. **(3)**
75. [Mussack T](http://www.ncbi.nlm.nih.gov/pubmed?term=Mussack%20T%5BAuthor%5D&cauthor=true&cauthor_uid=16424985), [Ladurner R](http://www.ncbi.nlm.nih.gov/pubmed?term=Ladurner%20R%5BAuthor%5D&cauthor=true&cauthor_uid=16424985), [Vogel T](http://www.ncbi.nlm.nih.gov/pubmed?term=Vogel%20T%5BAuthor%5D&cauthor=true&cauthor_uid=16424985), [Lienemann A](http://www.ncbi.nlm.nih.gov/pubmed?term=Lienemann%20A%5BAuthor%5D&cauthor=true&cauthor_uid=16424985), [Eder-Willwohl A](http://www.ncbi.nlm.nih.gov/pubmed?term=Eder-Willwohl%20A%5BAuthor%5D&cauthor=true&cauthor_uid=16424985), [Hallfeldt KK](http://www.ncbi.nlm.nih.gov/pubmed?term=Hallfeldt%20KK%5BAuthor%5D&cauthor=true&cauthor_uid=16424985) (2006) Health-related quality-of-life changes after laparoscopic and open incisional hernia repair: a matched pair analysis. [SurgEndosc](http://www.ncbi.nlm.nih.gov/pubmed?term=Surg%20Endosc%20%282006%29%2020%3A%20410%E2%80%93413) 20: 410-3. **(3)**
76. [Hope WW](http://www.ncbi.nlm.nih.gov/pubmed?term=Hope%20WW%5BAuthor%5D&cauthor=true&cauthor_uid=18721006), [Lincourt AE](http://www.ncbi.nlm.nih.gov/pubmed?term=Lincourt%20AE%5BAuthor%5D&cauthor=true&cauthor_uid=18721006), [Newcomb WL](http://www.ncbi.nlm.nih.gov/pubmed?term=Newcomb%20WL%5BAuthor%5D&cauthor=true&cauthor_uid=18721006), [Schmelzer TM](http://www.ncbi.nlm.nih.gov/pubmed?term=Schmelzer%20TM%5BAuthor%5D&cauthor=true&cauthor_uid=18721006), [Kercher KW](http://www.ncbi.nlm.nih.gov/pubmed?term=Kercher%20KW%5BAuthor%5D&cauthor=true&cauthor_uid=18721006), [Heniford BT](http://www.ncbi.nlm.nih.gov/pubmed?term=Heniford%20BT%5BAuthor%5D&cauthor=true&cauthor_uid=18721006) (2008) Comparing quality-of-life outcomes in symptomatic patients undergoing laparoscopic or open ventral hernia repair.[J Laparoendosc Adv Surg Tech A](http://www.ncbi.nlm.nih.gov/pubmed/18721006) 18: 567-71. **(2b**)
77. Uranues S, Salehi B, Bergamaschi R (2008) Adverse events, quality of life, and recurrence rates after laparoscopic adhesiolysis and recurrent incisional hernia mesh repair in patients with previous failed repairs. J Am CollSurg207:663-9. **(4)**
78. Wassenaar E, Schoenmaeckers E, Raymakers J, van der Palen J (2010) Mesh-fixation method and pain and quality of life after laparoscopic ventral or incisional hernia repair: a randomized trial of three fixation techniques. SurgEndosc 24:1296-302. **(1b)**
79. [Cobb WS](http://www.ncbi.nlm.nih.gov/pubmed?term=%22Cobb%20WS%22%5BAuthor%5D), [Kercher KW](http://www.ncbi.nlm.nih.gov/pubmed?term=%22Kercher%20KW%22%5BAuthor%5D), [Heniford BT](http://www.ncbi.nlm.nih.gov/pubmed?term=%22Heniford%20BT%22%5BAuthor%5D) (2005) Laparoscopic repair of incisional hernias.[SurgClin North Am](http://www.ncbi.nlm.nih.gov/pubmed/15619531) 85:91-103. **(5)**
80. Müller-Riemenschneider F, Roll S, Friedrich M, Zieren J, Reinhold T, von der Schulenburg JM, Greiner W, Willich SN (2007) Medical effectiveness and safety of conventional compared to laparoscopic incisional hernia repair: a systematic review. SurgEndosc21:2127–36. **(2a)**
81. [Cassar K](http://www.ncbi.nlm.nih.gov/pubmed?term=Cassar%20K%5BAuthor%5D&cauthor=true&cauthor_uid=11972542), [Munro A](http://www.ncbi.nlm.nih.gov/pubmed?term=Munro%20A%5BAuthor%5D&cauthor=true&cauthor_uid=11972542) (2002) Surgical treatment of incisional hernia.[Br J Surg](http://www.ncbi.nlm.nih.gov/pubmed/11972542)89: 534-45**. (2a)**
82. Bower CE, Reade CC, Kirby LW, Roth JS (2004) Complications of laparoscopic incisional-ventral hernia repair: the experience of a single institution. SurgEndosc 18: 672–675. **(4)**
83. [Beldi G](http://www.ncbi.nlm.nih.gov/pubmed?term=%22Beldi%20G%22%5BAuthor%5D), [Wagner M](http://www.ncbi.nlm.nih.gov/pubmed?term=%22Wagner%20M%22%5BAuthor%5D), [Bruegger LE](http://www.ncbi.nlm.nih.gov/pubmed?term=%22Bruegger%20LE%22%5BAuthor%5D), [Kurmann A](http://www.ncbi.nlm.nih.gov/pubmed?term=%22Kurmann%20A%22%5BAuthor%5D), [Candinas D](http://www.ncbi.nlm.nih.gov/pubmed?term=%22Candinas%20D%22%5BAuthor%5D) (2011) Mesh shrinkage and pain in laparoscopic ventral hernia repair: a randomized clinical trial comparing suture versus tack mesh fixation.[SurgEndosc](http://www.ncbi.nlm.nih.gov/pubmed?term=beldi%20mesh%20shrinkage%20and%20pain) 25:749-55. **(1b**)
84. [Heniford BT](http://www.ncbi.nlm.nih.gov/pubmed?term=Heniford%20BT%5BAuthor%5D&cauthor=true&cauthor_uid=14501505), [Park A](http://www.ncbi.nlm.nih.gov/pubmed?term=Park%20A%5BAuthor%5D&cauthor=true&cauthor_uid=14501505), [Ramshaw BJ](http://www.ncbi.nlm.nih.gov/pubmed?term=Ramshaw%20BJ%5BAuthor%5D&cauthor=true&cauthor_uid=14501505), [Voeller G](http://www.ncbi.nlm.nih.gov/pubmed?term=Voeller%20G%5BAuthor%5D&cauthor=true&cauthor_uid=14501505) (2003) Laparoscopic repair of ventral hernias: nine years' experience with 850 consecutive hernias.[Ann Surg](http://www.ncbi.nlm.nih.gov/pubmed?term=Laparoscopic%20Repair%20of%20Ventral%20Hernias%20Nine%20Years%E2%80%99%20Experience%20With%20850%20Consecutive%20Hernias)238: 391-9. **(4)**
85. Yavuz N, Ipek T, As A,Kapan M, Eyuboglu E, Erguney S (2005) [Laparoscopic repair of ventral and incisional hernias: our experience in 150 patients.](http://www.ncbi.nlm.nih.gov/pubmed/16366866) J Laparoendosc Adv Surg Tech A 15:601-5. **(4)**
86. Bedi AP, Bhatti T, Amin A, Zuberi J (2007) Laparoscopic incisional and ventral hernia repair. J Minim Access Surg 3:83-90. **(2a)**
87. [McKinlay RD](http://www.ncbi.nlm.nih.gov/pubmed?term=%22McKinlay%20RD%22%5BAuthor%5D), [Park A](http://www.ncbi.nlm.nih.gov/pubmed?term=%22Park%20A%22%5BAuthor%5D) (2004)Laparoscopic ventral incisional hernia repair: a more effective alternative to conventional repair of recurrent incisional hernia.[J GastrointestSurg](http://www.ncbi.nlm.nih.gov/pubmed/15358326) 8:670-4. **(2b)**
88. [LeBlanc KA](http://www.ncbi.nlm.nih.gov/pubmed?term=LeBlanc%20KA%5BAuthor%5D&cauthor=true&cauthor_uid=17287923) (2007) Laparoscopic incisional hernia repair: are transfascial sutures necessary? A review of the literature.[SurgEndosc](http://www.ncbi.nlm.nih.gov/pubmed/17287923) 21: 508-13. **(2a)**
89. [Rudmik LR](http://www.ncbi.nlm.nih.gov/pubmed?term=Rudmik%20LR%5BAuthor%5D&cauthor=true&cauthor_uid=16453075), [Schieman C](http://www.ncbi.nlm.nih.gov/pubmed?term=Schieman%20C%5BAuthor%5D&cauthor=true&cauthor_uid=16453075), [Dixon E](http://www.ncbi.nlm.nih.gov/pubmed?term=Dixon%20E%5BAuthor%5D&cauthor=true&cauthor_uid=16453075), [Debru E](http://www.ncbi.nlm.nih.gov/pubmed?term=Debru%20E%5BAuthor%5D&cauthor=true&cauthor_uid=16453075) (2006) Laparoscopic incisional hernia repair: a review of the literature.[Hernia](http://www.ncbi.nlm.nih.gov/pubmed?term=Rudmik%20LR%20ventral%20hernia) 10: 110-9. **(2a)**
90. [Ching SS](http://www.ncbi.nlm.nih.gov/pubmed?term=Ching%20SS%5BAuthor%5D&cauthor=true&cauthor_uid=18622552), [Sarela AI](http://www.ncbi.nlm.nih.gov/pubmed?term=Sarela%20AI%5BAuthor%5D&cauthor=true&cauthor_uid=18622552), [Dexter SP](http://www.ncbi.nlm.nih.gov/pubmed?term=Dexter%20SP%5BAuthor%5D&cauthor=true&cauthor_uid=18622552), [Hayden JD](http://www.ncbi.nlm.nih.gov/pubmed?term=Hayden%20JD%5BAuthor%5D&cauthor=true&cauthor_uid=18622552), [McMahon MJ](http://www.ncbi.nlm.nih.gov/pubmed?term=McMahon%20MJ%5BAuthor%5D&cauthor=true&cauthor_uid=18622552) (2008) Comparison of early outcomes for laparoscopic ventral hernia repair between nonobese and morbidly obese patient populations.[SurgEndosc](http://www.ncbi.nlm.nih.gov/pubmed/18622552) 22: 2244-50. **(2b)**
91. [Ceccarelli G](http://www.ncbi.nlm.nih.gov/pubmed?term=Ceccarelli%20G%5BAuthor%5D&cauthor=true&cauthor_uid=17623245), [Casciola L](http://www.ncbi.nlm.nih.gov/pubmed?term=Casciola%20L%5BAuthor%5D&cauthor=true&cauthor_uid=17623245), [Pisanelli MC](http://www.ncbi.nlm.nih.gov/pubmed?term=Pisanelli%20MC%5BAuthor%5D&cauthor=true&cauthor_uid=17623245), [Bartoli A](http://www.ncbi.nlm.nih.gov/pubmed?term=Bartoli%20A%5BAuthor%5D&cauthor=true&cauthor_uid=17623245), [Di Zitti L](http://www.ncbi.nlm.nih.gov/pubmed?term=Di%20Zitti%20L%5BAuthor%5D&cauthor=true&cauthor_uid=17623245), [Spaziani A](http://www.ncbi.nlm.nih.gov/pubmed?term=Spaziani%20A%5BAuthor%5D&cauthor=true&cauthor_uid=17623245), [Biancafarina A](http://www.ncbi.nlm.nih.gov/pubmed?term=Biancafarina%20A%5BAuthor%5D&cauthor=true&cauthor_uid=17623245), [Stefanoni M](http://www.ncbi.nlm.nih.gov/pubmed?term=Stefanoni%20M%5BAuthor%5D&cauthor=true&cauthor_uid=17623245), [Patriti A](http://www.ncbi.nlm.nih.gov/pubmed?term=Patriti%20A%5BAuthor%5D&cauthor=true&cauthor_uid=17623245)(2008) Comparing fibrin sealant with staples for mesh fixation in laparoscopic transabdominal hernia repair: a case control-study. [SurgEndosc](http://www.ncbi.nlm.nih.gov/pubmed?term=Surg%20Endosc%20%282008%29%2022%3A668%E2%80%93673)22: 668-73. **(2b)**
92. Chowbey PK, Sharma A, Khullar R Mann V, Baijal M, Vashistha A (2000) Laparoscopic ventral hernia repair. J Laparoendosc Adv Surg.Tech A 10: 79–84. **(4)**
93. Binnebosel M, Rosch R, Junge K, Flanagan TC, Schwab R, Schumpelick V, Klinge U (2007) Biochemical analysis of overlap and mesh dislocation in an incisional hernia model in vitro. Surgery 142: 365-71. **(4)**
94. [Verbo A](http://www.ncbi.nlm.nih.gov/pubmed?term=Verbo%20A%5BAuthor%5D&cauthor=true&cauthor_uid=17907969), [Petito L](http://www.ncbi.nlm.nih.gov/pubmed?term=Petito%20L%5BAuthor%5D&cauthor=true&cauthor_uid=17907969), [Manno A](http://www.ncbi.nlm.nih.gov/pubmed?term=Manno%20A%5BAuthor%5D&cauthor=true&cauthor_uid=17907969), [Coco C](http://www.ncbi.nlm.nih.gov/pubmed?term=Coco%20C%5BAuthor%5D&cauthor=true&cauthor_uid=17907969), [Mattana C](http://www.ncbi.nlm.nih.gov/pubmed?term=Mattana%20C%5BAuthor%5D&cauthor=true&cauthor_uid=17907969), [Lurati M](http://www.ncbi.nlm.nih.gov/pubmed?term=Lurati%20M%5BAuthor%5D&cauthor=true&cauthor_uid=17907969), [Pedretti G](http://www.ncbi.nlm.nih.gov/pubmed?term=Pedretti%20G%5BAuthor%5D&cauthor=true&cauthor_uid=17907969), [Rizzo G](http://www.ncbi.nlm.nih.gov/pubmed?term=Rizzo%20G%5BAuthor%5D&cauthor=true&cauthor_uid=17907969), [Sermoneta D](http://www.ncbi.nlm.nih.gov/pubmed?term=Sermoneta%20D%5BAuthor%5D&cauthor=true&cauthor_uid=17907969), [Lodoli C](http://www.ncbi.nlm.nih.gov/pubmed?term=Lodoli%20C%5BAuthor%5D&cauthor=true&cauthor_uid=17907969), [Nunziata J](http://www.ncbi.nlm.nih.gov/pubmed?term=Nunziata%20J%5BAuthor%5D&cauthor=true&cauthor_uid=17907969), [D'Ugo D](http://www.ncbi.nlm.nih.gov/pubmed?term=D%27Ugo%20D%5BAuthor%5D&cauthor=true&cauthor_uid=17907969) (2007) Laparoscopic approach to recurrent incisional hernia repair: a 3-year experience.[J Laparoendosc Adv Surg Tech A](http://www.ncbi.nlm.nih.gov/pubmed/17907969) 17:591-5. **(4)**
95. [Theodoropoulou K](http://www.ncbi.nlm.nih.gov/pubmed?term=Theodoropoulou%20K%5BAuthor%5D&cauthor=true&cauthor_uid=20529535), [Lethaby D](http://www.ncbi.nlm.nih.gov/pubmed?term=Lethaby%20D%5BAuthor%5D&cauthor=true&cauthor_uid=20529535), [Hill J](http://www.ncbi.nlm.nih.gov/pubmed?term=Hill%20J%5BAuthor%5D&cauthor=true&cauthor_uid=20529535), [Gupta S](http://www.ncbi.nlm.nih.gov/pubmed?term=Gupta%20S%5BAuthor%5D&cauthor=true&cauthor_uid=20529535), [Bradpiece H](http://www.ncbi.nlm.nih.gov/pubmed?term=Bradpiece%20H%5BAuthor%5D&cauthor=true&cauthor_uid=20529535) (2010) Laparoscopic hernia repair: a two-port technique.[JSLS](http://www.ncbi.nlm.nih.gov/pubmed?term=Theodoropoulou%202010%20incisional%20hernia) 14: 103-5. **(4)**
96. [Costanza MJ](http://www.ncbi.nlm.nih.gov/pubmed?term=Costanza%20MJ%5BAuthor%5D&cauthor=true&cauthor_uid=9843329), [Heniford BT](http://www.ncbi.nlm.nih.gov/pubmed?term=Heniford%20BT%5BAuthor%5D&cauthor=true&cauthor_uid=9843329), [Arca MJ](http://www.ncbi.nlm.nih.gov/pubmed?term=Arca%20MJ%5BAuthor%5D&cauthor=true&cauthor_uid=9843329), [Mayes JT](http://www.ncbi.nlm.nih.gov/pubmed?term=Mayes%20JT%5BAuthor%5D&cauthor=true&cauthor_uid=9843329), [Gagner M](http://www.ncbi.nlm.nih.gov/pubmed?term=Gagner%20M%5BAuthor%5D&cauthor=true&cauthor_uid=9843329) (1998) Laparoscopic repair of recurrent ventral hernias.[Am Surg](http://www.ncbi.nlm.nih.gov/pubmed/9843329)64: 1121-5. **(4)**
97. Toy F K, Bailey R W, Carey S, Chappuis C W, Gagner M, Josephs L G, Mangiante E C, Park A E, Pomp A, Smoot R T et al (1998)Prospective, multicenter study of laparoscopic ventral hernioplasty - Preliminary results. SurgEndosc12:955-959. **(4)**
98. [Koehler RH](http://www.ncbi.nlm.nih.gov/pubmed?term=Koehler%20RH%5BAuthor%5D&cauthor=true&cauthor_uid=10694076), [Voeller G](http://www.ncbi.nlm.nih.gov/pubmed?term=Voeller%20G%5BAuthor%5D&cauthor=true&cauthor_uid=10694076) (1999) Recurrences in laparoscopic incisional hernia repairs: a personal series and review of the literature.[JSLS](http://www.ncbi.nlm.nih.gov/pubmed?term=Koehler%20RH%201999) 3: 293-304. **(4)**
99. [Szymanski J](http://www.ncbi.nlm.nih.gov/pubmed?term=Szymanski%20J%5BAuthor%5D&cauthor=true&cauthor_uid=10890970), [Voitk A](http://www.ncbi.nlm.nih.gov/pubmed?term=Voitk%20A%5BAuthor%5D&cauthor=true&cauthor_uid=10890970), [Joffe J](http://www.ncbi.nlm.nih.gov/pubmed?term=Joffe%20J%5BAuthor%5D&cauthor=true&cauthor_uid=10890970), [Alvarez C](http://www.ncbi.nlm.nih.gov/pubmed?term=Alvarez%20C%5BAuthor%5D&cauthor=true&cauthor_uid=10890970), [Rosenthal G](http://www.ncbi.nlm.nih.gov/pubmed?term=Rosenthal%20G%5BAuthor%5D&cauthor=true&cauthor_uid=10890970) (2000)Technique and early results of outpatient laparoscopic mesh onlay repair of ventral hernias. [SurgEndosc](http://www.ncbi.nlm.nih.gov/pubmed/10890970) 14:582-4. **(4)**
100. [Lau H](http://www.ncbi.nlm.nih.gov/pubmed?term=Lau%20H%5BAuthor%5D&cauthor=true&cauthor_uid=12459597), [Patil NG](http://www.ncbi.nlm.nih.gov/pubmed?term=Patil%20NG%5BAuthor%5D&cauthor=true&cauthor_uid=12459597), [Yuen WK](http://www.ncbi.nlm.nih.gov/pubmed?term=Yuen%20WK%5BAuthor%5D&cauthor=true&cauthor_uid=12459597), [Lee F](http://www.ncbi.nlm.nih.gov/pubmed?term=Lee%20F%5BAuthor%5D&cauthor=true&cauthor_uid=12459597) (2002) Laparoscopic incisional hernioplastyutilising on-lay expanded polytetrafluoroethylene DualMesh: prospective study.[Hong Kong Med J](http://www.ncbi.nlm.nih.gov/pubmed/12459597)ournal 8: 413-7. **(4)**
101. Bageacu S, Blanc P, Breton C, Gonzaled M, Porcheron J, Chabert M, Balique JG (2002) Laparoscopic repair of incisional hernia: A retrospective study of 159 patients. SurgEndosc 16: 345-348. **(4)**
102. Ben-Haim M, Kuriansky J, Tal R, Zmora A, MintzY, Rosin D, Ayalon A, Shabtai M (2002) Pitfalls and complications with laparoscopic intraperitoneal expanded polytetrafluoroethylene patch repair of postoperative ventral hernia. SurgEndosc 16: 785–788. **(4)**
103. [Gillian GK](http://www.ncbi.nlm.nih.gov/pubmed?term=Gillian%20GK%5BAuthor%5D&cauthor=true&cauthor_uid=12500829), [Geis WP](http://www.ncbi.nlm.nih.gov/pubmed?term=Geis%20WP%5BAuthor%5D&cauthor=true&cauthor_uid=12500829), [Grover G](http://www.ncbi.nlm.nih.gov/pubmed?term=Grover%20G%5BAuthor%5D&cauthor=true&cauthor_uid=12500829) (2002) Laparoscopic incisional and ventral hernia repair (LIVH): an evolving outpatient technique. [JSLS](http://www.ncbi.nlm.nih.gov/pubmed?term=gillian%20ventral%20hernia) 6:315-22. **(4)**
104. [Eitan A](http://www.ncbi.nlm.nih.gov/pubmed?term=Eitan%20A%5BAuthor%5D&cauthor=true&cauthor_uid=12470403), [Bickel A](http://www.ncbi.nlm.nih.gov/pubmed?term=Bickel%20A%5BAuthor%5D&cauthor=true&cauthor_uid=12470403) (2002)Laparoscopically assisted approach for postoperative ventral hernia repair. [J Laparoendosc Adv Surg Tech A](http://www.ncbi.nlm.nih.gov/pubmed/12470403) 12:309-11. **(4)**
105. [Moreno-Egea A](http://www.ncbi.nlm.nih.gov/pubmed?term=Moreno-Egea%20A%5BAuthor%5D&cauthor=true&cauthor_uid=19533233), [Bustos JA](http://www.ncbi.nlm.nih.gov/pubmed?term=Bustos%20JA%5BAuthor%5D&cauthor=true&cauthor_uid=19533233), [Girela E](http://www.ncbi.nlm.nih.gov/pubmed?term=Girela%20E%5BAuthor%5D&cauthor=true&cauthor_uid=19533233), [Aguayo-Albasini JL](http://www.ncbi.nlm.nih.gov/pubmed?term=Aguayo-Albasini%20JL%5BAuthor%5D&cauthor=true&cauthor_uid=19533233) (2010)Long-term results of laparoscopic repair of incisional hernias using an intraperitoneal composite mesh. [SurgEndosc](http://www.ncbi.nlm.nih.gov/pubmed/19533233) 24: 359-65. **(4)**
106. Frantzides CT, Carlson MA, Zografakis JG, Madan AK, Moore RE (2004) Minimally invasive incisional herniorrhaphy: a review of 208 cases. SurgEndosc 18: 1488–1491.**(4)**

**Table 1 Level 1a Studies Comparing Laparoscopic vs Open repair of Incisional and Ventral Hernia**

| **Author** | **No of patient (Lap/**  **open)** | **Follow up (months)**  **Mean/**  **range** | **Hospital stay (Lap/open)**  **Range (days)** | **Return to activity/**  **work(days)**  **(Lap/**  **open)** | **Cost (Lap/**  **open)** | **QOL (Lap/**  **open)** | **Acute Pain (Lap/**  **open)** | **Chronic pain**  **(Lap/**  **open)** | **Recur-**  **rence (Lap/**  **open)** |
| --- | --- | --- | --- | --- | --- | --- | --- | --- | --- |
| Sajid et al, 2008 [13]  5 RCTs | 366  183/183 | 2-27 | Shorter hospital stay in lap, SMD -1.82; 95% CI -3.21 to -0.44; p = 0.01 | NR | NR | NR | No difference  CI -0.41 TO 0.33; p = 0.84  (2 trials) | NR | RR-1.0;95% CI 0.31-3.2  (p = 1) |
| Forbes SS et al, 2009 [12]  8 RCTs | 517  269/253 | 23 | 1.1 – 5.7/1.33 - 9.06  6 RCTs less hospital stay in lap | 13/25, p = <0.005  (1 study) | NR | NR | NR | NR | 3.4%/3.6%  RR-1.02;95% CI 0.41 to 2.54  (p = 0.56) |
| Sauer-  land et al, 2011 [2]  9 RCTs | 880  446/434 | 2-27 | 1.1 – 5.7/1.33 - 9.06  (p = 0.56)  6 RCTs less hospital stay in lap | No difference  CI -2.1 to 0.7,  p = 0.33  (2 studies) | CI 1.84 – 3.14, p < 0.00001  (1 study) | No difference  p = 0.11  (1 study) | CI -0.45 to 0.62. p = 0.75  (4 studies) | CI -0.24 TO 1.11, p = 0.2  (1 study) | RR-1.22;95% CI 0.62 to 2.38  (p = 0.56) |

| **Table 2 Level 1b Studies Comparing Laparoscopic vs Open repair of Incisional and Ventral Hernia** | | | | | | | | | |
| --- | --- | --- | --- | --- | --- | --- | --- | --- | --- |
| **Author** | **No of patient (Lap/**  **open)** | **Follow up**  **(months)**  **Mean/**  **range** | **Hospital stay (Lap/**  **open)**  **Range (days)** | **Return to activity/work (days)**  **(Lap/**  **open)** | **Cost (Lap/**  **open)** | **QOL (Lap/**  **open)** | **Acute Pain (Lap/**  **open)** | **Chronic pain**  **(Lap/**  **open)** | **Recur-rence (Lap/**  **open)** |
| Carbajo et al , 1999 [5] | 60, 30/30 | 24 | 2.23/9.06  ( p = <0.05) | NR | NR | NR | NR | NR | 0/2  (p = 0.05) |
| Moreno –egea et al, 2002 [6] | 22, 11/11 | 24 | 1.1/5.2  ( p = <0.001) | NR | NR | NR | NR | NR | 0/0 |
| Barbaros et al, 2005 [4] | 46, 23/23 | 24 | 2.5/6.3  (p = <0.05) | NR | NR | NR | 1.53/1.61 (p = >0.05) | NR | 0/1  (p = <0.05) |
| Misra et al,  2006 [7] | 62, 32/30 | 12.17 | 1.47/3.43  (p = 0.007) | NR | Rs13786.90  ($ 288)/ Rs1536.66  ($ 32)  (p = 0.01) | Patient satisfaction score 8.27/7.6  (p = 0.26) | Day1 5.95/6.05  Day3 2.33/2.16 (p = 0.857) | 3 months 6/3 (pts)  12 months 1/1 | 2/1 [7.4%/4%]  (p = 0.95) |
| Navarra et al ,2007 [9] | 24,12/12 | < 12 | 5.7/10  (p = 0.006) | NR | NR | NR | NR | NR | 0/0 |
| Olmi et al, 2007 [10] | 170, 85/85 | 24 | 2.7/9.9  (p = <0.005) | 13 (6-15)/ 25 (16-30)  (p = <0.005) | € 2700/  € 3100  (theoretical calculation) | NR | NR | NR | 2/1 [2.3%/1.1%]  (p = NS) |
| Pring et al ,2008 [11] | 54, 30/24 | 27.5 | 1.46/1.33  (p = 0.43) | 3.9 (SD 1.3)/ 4.6 (SD 3.3)  (p = 0.33) | NR | NR | 6.067/6.292  (p = >0.05) | NR | 1/1  (p = NS) |
| Asencio et al, 2009 [3] | 84, 45/39 | 12 | 3.46/3.33  (p = 0.78) | NR | NR | RR -0.04, CI -0.10 to 0.01, p = 0.11 | Day1 47.66/42.86  (p = 0.285)  Day7  28.93/22.55  (p = 0.205) | 3 months  13.51/7.10  (p = 0.056)  12 months  10.31/6.00  (p = 0.201) | 9.7%/7.9%  (p = 0.77) |
| Itani et al, 2010 [6] | 146, 73/73 | 24 | 4.0/3.9  (p = 0.91) | Return to work 23.0/28.5  (p = 0.06) | NR | No difference in improvement at 8 wks (p = 0.17 for pain at rest and p = 0.07 for worst pain | NR | NR | 9/6 [12.5%/8.2%]  (p = 0.44) |

| **Table 3 Level 2a Studies Comparing Laparoscopic vs Open repair of Incisional and Ventral Hernia** | | | | | | | | | | |
| --- | --- | --- | --- | --- | --- | --- | --- | --- | --- | --- |
| **Author** | **No of patient (Lap/open)** | **Follow up (months)**  **Mean/**  **range** | **Hospital stay (Lap/open)**  **Range (days)** | **Return to activity/**  **work (days)**  **(Lap/open)** | **Cost (Lap/**  **open)** | **QOL (Lap/open)** | **Acute Pain (Lap/**  **open)** | **Chronic pain**  **(Lap/open)** | **Recurrence (Lap/open)** |  |
| Cassar et al ,2002 [80]  (19 studies) | 1896,  1598/298 | 6-53 | NR | NR | NR | NR | NR | 1.8%  (4 studies) | 0-9%/  0-10% |  |
| Goodney et al, 2002 [14] | 712  322/ 390 | NR | 2/4  (p = 0.02) | NR | NR | NR | NR | NR | NR |  |
| Rudmik et al, 2006 [88]  (10 studies) | 2060  All lap | 34 | 3.2/2.2 tack vs suture | NR | NR | NR | NR | NR | 4.5/4.4  (tack vs  Suture) |  |
| Sains et al, 2006 [16]  (4 studies) | 351,  148/203 | 1-85 | RR 3.2, CI -5.4 to 1.15  (p = 0.003) | NR | NR | NR | NR | NR | 0.06 |  |
| Pierce et al , 2006 [17]  (45 studies) | 5340, 4582/758 | 17-25 | 2.4/4.3 (p = 0.0004) | NR | NR | NR | NR | 1.0%/0.9%  (p = 0.93) | 4.3%/12.1%  p = <0.0001 |  |
| LeBlanc et al, 2007 [87]  (12 studies) | 3434  All lap  With or without sutures | 6-49 | NR | NR | NR | NR | NR | NR | 1.8%/ 4%  With and  without  sutures |  |
| Bedi et al, 2007 [85]  (34 studies) | 3266  All lap | 29.7 | NR | NR | NR | NR | 2.75% | NR | 3.67% |  |
| Mullar-Riemenschneider et al, 2007 [79]  (15 studies) | 2008  906/1102 | 1- 24 | 3.4 | NR | NR | NR | NR | 3.6%/4.1%  (p >0.05) | 0-20.7%/  0-35% |  |
| Pham et al, 2009 [15]  (9 studies) | 1066  497/569 | 2 -24 | 0.8 – 3.4/1.5-9.06 | NR | NR | NR | NR | 6.1%/4.1% | 0-13%  0-20.7% |  |
| Total | 20073  16753/3320 | 1-85 | 2.4/4.3 | NR | NR | NR | NR | 3.6%/3% | 0-20.7%/  0-35% |  |

**Table 4 Level 2b Studies Comparing Laparoscopic vs Open repair of Incisional and Ventral Hernia**

| **Author** | **No of patient (Lap/**  **open)** | **Follow up (months)**  **Mean/**  **range** | **Hospital stay (Lap/**  **open)**  **Range (days)** | **Return to activity/work (days)**  **(Lap/**  **open)** | **Cost (Lap/open)** | **QOL (Lap/**  **open)** | **Acute Pain (Lap/**  **open)** | **Chronic pain**  **(Lap/**  **open)** | **Recur-rence (Lap/**  **open)** |
| --- | --- | --- | --- | --- | --- | --- | --- | --- | --- |
| Park et al ,1998 [18] | 105  49/56 | 24.1/53.7 | 3.4/6.5  (p < 0.001) | NR | NR | NR | NR | NR | 21%/3% |
| Demaria et al, 2000 [19] | 39  21/18 | 12-24 mth | 0.8/4.4  (p < 0.05) | NR | $8273±$2950/  $12461±$5987  (p < 0.05)  Initial and readmission cost | NR | Analgesic use 10% vs 79%  (p < 0.05) | NR | 5%/0% |
| Mckinlay et al ,2004 [86] | 170  69-recurrent  101-Primary | 19/27 | NR | NR | NR | NR | NR | 2.8%/0 in recurrent vs Primary | 7%-recur-rent  5%-Primary  (p = 0.53) |
| Lomanto et al , 2005 [20] | 100  50/50 | 20.8 | 2.7/4.7  (p = 0.044) | NR | NR | NR | VAS 2.9/4.1  (p = 0.001) | NR | 2%/10% |
| Olmi S et al, 2005 [30] | 50  25/25 | 9/24.5 | NR | NR | NR | NR | NR | NR | 2%/0% |
| Bingener et al, 2007 [21] | 360  127/233 | 36/25 | 0.9 ± 1.4/1.4 ± 2.0  (p = 0.01) | NR | NR | NR | NR | NR | 9%/12%  (p = 0.36) |
| Chings et al, 2008 [89] | 168  All lap  (42 mor-bidly-obese/  124 non obese) | 19 | NR | NR | NR | NR | NR | Obese/ non obese  5%/  10%  (p = 0.5) | Obese/ non obese  10%/  13%  (p = 0.78) |
| Ceccarrelli et al, 2008  [90] | 181  94/87 | 38 | NR | NR | NR | NR | NR | NR | 2.1%/6.9%  (p > 0.05) |
| Kurmann et al, 2011 [22] | 125  69/56 | 32.5/65 | 6 (1-23)/7 (1-67)  p = 0.001 | Return to work 21/42  p > 0.05 | NR | NR | NR | VAS 0.5/0.6  P > 0.05 | 18%/16%  (p = 0.6) |
| Total | 960  435/525 | 24.8/34.4  (7 studies) | 2.7/4.6  (5 studies) | Return to work 21/42  (1 study) | $8273±$2950/  $12461±$5987  (1 study) | NR | 2.9/4.1  (p = 0.001)  (1 study) | 0.5/0.6  P > 0.05  (1 study) | 8.4%/6.8%  (7 studies) |

**Table 5 Level 3 Studies Comparing Laparoscopic vs Open repair of Incisional and Ventral Hernia**

| **Author** | **No of patient (Lap/**  **open)** | **Follow up (months)**  **Mean/**  **range** | **Hospital stay (Lap/**  **open)**  **Range (days)** | **Return to activity/work (days)**  **(Lap/**  **open)** | **Cost (Lap/**  **open)** | **QOL (Lap/**  **open)** | **Acute Pain (Lap/**  **open)** | **Chronic pain**  **(Lap/**  **open)** | **Recur-rence (Lap/**  **open)** |
| --- | --- | --- | --- | --- | --- | --- | --- | --- | --- |
| Holzman et al, 1997 [23] | 37, 21/16 | 20/18.8 | 2/5  (p = NS) | NR | $4395/  $7299  (p < 0.05) | NR | NR | NR | 9.5%/12.5% |
| Ramshaw et al ,1999 [24] | 253, 174/79 | 21 | 2/3  (p = NS) | NR | NR | NR | NR | NR | 21%/3% |
| Chari et al, 2000 [25] | 28, 14/14 | 6-27 | -0.4  (p = NS) | NR | NR | NR | NR | NR | 0%/0% |
| Zanghi et al, 2000[26] | 26, 15/11 | 25 | 2/5  P< 0.05 | NR | NR | NR | NR | NR | 0%/0% |
| Van’t et al, 2002 [27] | 101, 25/76 | 15/17 | 4/5  (p = 0.28) | NR | NR | NR | NR | NR | 15%/ 8%  (p = 0.91) |
| Raftopou-los et al, 2003 [28] | 72, 50/22 | NR | 1.88 VS 5.38  P< 0.001 | 22.1/37.5  (p = NS) | NR | NR | NR | NR | 2%/18%  P<0.028 |
| Bencini et al, 2003 [29] | 91,42/49 | 17/18 | 5/8  P<0.0001 | NR | € 3091/  € 3936  (p = 0.017) | NR | NR | NR | 0 / 6%  (p = 0.3) |
| Olmi et al, 2005 [30] | 100, 50/50 | 9/24.5 | 2.1/8.1  (p < 0.05) | NR | NR | NR | NR | NR | 2%/0%  P value ns |
| Beldi et al, 2006 [31] | 141, 49/92 | 2.5/16 | 6 (3–32)/7 (2–87)  (p = 0.02) | NR | € 9787/  € 7654  (p = 0.02) | NR | 6.1%/5.4%  (p = 1) | NR | 4%/0  NS |
| Mussack et al, 2006 [74] | 48, 24/24 | 16-28 | NR | NR | NR | NS | NR | NR | 4% / 0%  (p = NS) |
| Zografos et al, 2007 [32] | 106  30/76 | 44 | 5.5/3.5  (p = NS) | NR | NR | NR | NR | NR | 3.3%/2.6% |
| Engledow et al, 2007 [67] | 31 lap only (day care vs in-patient) | 15 | NR | NR | £ 908/  £ 1514 | NR | NR | NR | NR |
| Total | 997  494/503 | 6-44 | 3.4/5.5 | 22.1/37.5  (1 study) | $ 6798/  $ 7232 | NR | 6.1%/5.4%  (p = 1)  (1 study) | NR | 6.7%/5.6% |

**Table 6 Level 4 Studies in Laparoscopic repair of Incisional and Ventral Hernia**

| **Author** | **No. of patient** | **Follow up (months)** | **Hospital stay (days)** | **Return to activity/work**  **(days)** | **Chronic pain (%)** | **Recurrence (%)** |
| --- | --- | --- | --- | --- | --- | --- |
| Brown et al, 1994 [33] | 118 | 42 | 1 | 7 | NR | 3.5% |
| Park et al, 1996 [18] | 30 | 8 | NR | NR | NR | 1% |
| Franklin et al, 1998 [34] | 176 | 1 – 84 | 1-12 | NR | NR | 1.1 % |
| Tsimoyiannis et al, 1998 [35] | 11 | 15 | 3 (2-6) | NR | NR | 0 |
| Costanza et al, 1998 [94] | 16 | 18 | NR | NR | NR | 5% |
| Toy et al, 1998 [96] | 144 | 7 | NR | NR | NR | 4% |
| Koehler et al, 1999 [97] | **32** | **20** | NR | NR | NR | **9%** |
| sanders et al, 1999 [36] | 12 | 12.5 | 3.5 | NR | NR | 8.5% |
| Heniford et al, 2000 [37] | 407 | 23 | 1.6 (1-4) | NR | NR | 3.4% |
| Chowbey et al, 2000 [92] | 202 | 33 | NR | NR | NR | 0.9% |
| Szymanski et al, 2000 [98] | 44 | 6 | NR | NR | NR | 5% |
| Lau et al, 2001 [99] | 10 | NR | NR | NR | NR | No early recurrence |
| Le blanc et al, 2001 [38] | 100 | 51 | 1 | NR | NR | 9% |
| Moreno-Egea et al, 2002 [39] | 55 | 18 | 3-6 | NR | NR | 2% |
| Bageacu et al, 2002 [100] | 159 | 49 | NR | NR | NR | 15.7% |
| Kua et al, 2002 [40] | 30 | 1 to 69 | 0-11 (57%-1 day, 27%-2 days) | 82% within a week | NR | 10% |
| Raftopoules et al, 2002 [41] | 50 | NR | 2.2 | NR | NR | 0 |
| Varghese et al, 2002 [42] | 32 | 8 | 1.8 | NR | NR | 6.7% |
| Berger et al, 2002 [43] | 150 | 15 | 9/10, primary vs recurrent | NR | NR | 2.7% |
| Kirshtein et al, 2002 [44] | 103 | 26 | 2-3 | NR | NR | 4% |
| Ben-haim et al, 2002 [101] | 100 | 19 | NR | NR | NR | 2% |
| Aura et al, 2002 [45] | 86 | 37 | 4.8 | NR | NR | 7% |
| Gillian et al, 2002 [102] | 100 | 27 | NR | NR | NR | 5% |
| Eitan et al, 2002 [103] | 62 | 36 | NR | NR | NR | 0 |
| Chowbey et al, 2003 [46] | 34 | 16.5 | 1 | NR | NR | 2.9% |
| Leblanc et al, 2003 [47] | 200 | 36 | 1.2 | NR | NR | 6.5% |
| Carbajo et al, 2003 [48] | 270 | 44 | 1.5 | NR | 7.4% | 4.4% |
| Heniford et al, 2003 [83] | 850 | 20.2 | 2.3 | NR | 1.6% | 4.7% |
| Eid et al, 2003 [50] | 79 | 72 | 1.7 | NR | 3.8% | 5% |
| Tagaya nobumi et al, 2004 [51] | 10 | 64 | 9.4 | NR | NR | 20% |
| Bower et al, 2004 [81] | 100 | 6.5 | NR | NR | 3% | 2% |
| Ujiki et al, 2004 [52] | 100 | 3 | 2 | NR | NR | 6% |
| Franklin et al, 2004 [53] | 384 | 47.1 | 2.9 | NR | NR | 2.9% |
| Bamehriz et al, 2004 [54] | 28 | 10.7 | 3.7 ± 0.3 | NR | 7% | 3.5% |
| Perrone et al, 2005 [55] | 121 | 6 | 1.7 ± 1 | NR | 3.3% | 9.3% |
| Yavuz et al, 2005 [84] | 150 | 32 | NR | NR | NR | 3% |
| Motson et al, 2006 [56] | 117 | 42 | 1 | NR | 2.5% | 7.7% |
| Verbo et al, 2007 [93] | 41 | 38 | NR | NR | NR | 2.4% |
| Chelala et al, 2007 [57] | 400 | 28 | 3 | NR | 2.5% | 1.5% |
| Raftopoulos et al, 2007 [58] | 27  BMI>35  Men BMI-46.9 | 14.9 | 3.6 | NR | NR | 18.5% |
| Uranues et al, 2008 [76] | 85 | 41 | NR | NR | 7% | 3.5% |
| Moreno-egea et al, 2008 [63] | 199  127-day cases  72-in-patients | NR | NR | < 10 | NR | 4.4% |
| Engledow et al, 2007 [67] | 31Day care | 15 | NR | 21 (2-180) | NR | 0 |
| Wassener et al, 2008 [59] | 505 | 19.8 | 3.2 ± 1 | NR | 2.2% | 1.8% |
| Berrevoet et al, 2009 [60] | 114 | 27 | 2.4 | NR | 1.8% | 3.5% |
| Baccari et al, 2009 [61] | 200 | 22 | 3 | NR | 1% |  |
| Edwards et al 2009 [62]  (Flank hernia) | 27 | 3.6 | 3.1 | NR | 9% | - |
| Eriksen et al, 2009 [68] | 35 | 6 | NR | 14 (1-38) | NR | NR |
| Moreno –Egae et al, 2010 [104] | 200 | 12 | 2.6 | NR | 0 | 6.2% |
| Theodoropoulous et al , 2010 [94] | 40 | 23.5 | NR | NR | NR | 2.5% |
| Olmi et al, 2010 [64]  (Glue fixation) | 19 | 20 | 1.5 | NR | NR | 0 |
| Sharma et al, 2011 [65] | 1242 | 64 | 1.9 | NR | 14.7% | 4.4 % |
| Sturt et al, 2011 [66] | 227 | 53 | 1 (0-17) | NR | 3.5% | 11% |
| Total | 8301 | 26.2 | 2.4 | 17.5 (return to work)  (2 studies) | 4.7% | 5.4% |

**Section 8: Mesh technology**

**Do we have an ideal mesh in terms of prevention of adhesions? Are coated meshes really necessary? Are there data to support the manufacturers’ claims of superiority? Is a permanent or absorbable barrier preferred?**

F. Köckerling, D. Weyhe, M. C. Misra, U. Klinge, J. Kukleta

Search terms: "Incisional Hernia", "Ventral Hernia", "Laparoscopic Incisional Hernia Repair", "Laparoscopic Ventral Hernia Repair", "Hernia Repair and Meshes", "Meshes", "Mesh Repair", "Laparoscopic Ventral Hernia Repair and Meshes", "Incisional Hernia Repair and Meshes"

A systematic search of the available literature was performed in July 2012 using Medline, PubMed, Cochrane Library and relevant journals and reference lists using the above listed search terms.

The first search detected 78 relevant articles. In a second - level search 2 articles were added. In Summary 26 articles and studies were used for this review.

**Key questions:**

**Do we have an ideal mesh in terms of prevention of adhesions? Are coated meshes really necessary? Are there data to support the manufacturers’ claims of superiority? Is a permanent or absorbable barrier preferred?**

**Introduction**

- In general, clinical studies usually do not have sufficient power to confirm any claim of superiority of any device. Considering the heterogeneity of the patients, the surgeons and the procedures, a specific impact of the device to change the outcome is hardly possible with study cohorts of < 1000 patients per group and a survey of many years. Thus, postmarket surveillance of devices should always (and compulsory) be supplemented by documentation in clinical registries. These will not be able to prove any superiority, but at least will help to identify devices with poor performance more easy. Because of the principally limited power of clinical studies to confirm the suitability of any device preclinical and experimental tests often are the best way to design appropriate devices.

- Adhesions after laparoscopic ventral hernia repair is a common phenomenon, due to trauma of surgery and to reaction to the mesh and/or fixation devices. There is no technique and no device that prevents completely and for sure the formation of adhesions.

- Direct contact of visceral organs to polypropylene and polyester is followed by dense adhesions to the mesh, subsequently leading to high risk of bowel resection in case of revision operations, and suspected to be followed by a higher risk for development of fistula to the bowels. This risk is decreased when using films (PTFE), or textile meshes made either of PVDF, polypropylene or polyester, but then with an additional coating/barrier function (titanium, collagen, cellulose, hyaluronic acid, PDS).

- Any film-like long term barrier covering a textile will initiate a tissue response comparable to those of a pure film-like device with encapsulation of the entire prosthesis. As any demage to peritoneum is healed within days, a temporary protection of the polymer surface should be sufficient. However, as outlined before, whether a sufficient protection is provided by a temporary or permanent barrier is widely influenced by the textile material and construction, and has to be verified for each device

**Statements**

| Level 4  Level 5 | Laparoscopic ventral and incisional hernia repair can be performed with the use of e-PTFE, PVDF or composite meshes with permission for use in the abdominal cavity.  The results of experimental studies on large animals with laparoscopic ventral hernia repair and comparison of meshes show advantages of light-weight polypropylene meshes vs. heavy-weight meshes, ePTFE and composite meshes vs. pure polypropylene meshes, composite meshes vs. ePTFE meshes and composite meshes vs. composite meshes.  Following laparoscopic incisional hernia repair adhesions will develop in at least 2/3 of the patients. Adhesions can not be prevented for sure by any of the materials used as IPOM, adhesions have to be expected in most of the patients.  Materials for use within the abdominal cavity can be made of ePTFE, PVDF, polyester or polypropylene, whereas the latter, if not with film-like structure, needs further barrier to prevent any direct contact to the bowels (composite meshes). Unprotected porous polypropylene and polyester meshes, which were placed in direct contact to the bowels, show a higher risk for bowel erosion or for bowel resection at subsequent surgery.  With every material similarly a low recurrence rate can be achieved if adequate technique is applied.  Film-like materials tend to show encapsulation and sometimes extensive shrinkage, and need a permanent fixation.  Enterocutaneous fistula after laparoscopic ventral hernia repair are rare events, in particularly for ePTFE.  Experimental studies in animals showed contradictory results and are hardly comparable.  Tissue integration of the various devices with different design characteristics differ and require different fixation techniques.  There is no ideal mesh, but every mesh has to be considered as a compromise in regard to strength, elasticity, tissue ingrowth and cellular response with specific advantages and disadvantages in some regards.  Most devices demonstrate a lack of stretchability, so that folding of the fixed mesh after release of the Pneumoperitoneum may be unvavoidable. |
| --- | --- |

**Recommendations**

| Grade C  Grade D | For laparoscopic incisional and ventral hernia repair only materials with permission for use in the abdominal cavity (PTFE, PVDF and composite meshes) should be used. Meshes without permission for use in the abdominal cavity should not be used outside of studies.  ePTFE is prone for persisting presence of bacteria and therefore should be explanted in case of severe contamination (see chapter "Mesh Infection").  The final choice of mesh will therefore for the moment typically be based on the surgeon's preference while awaiting further data from controlled clinical trials.  Based on today's knowledge plain polypropylene (without a protective layer) cannot be recommended for intra-abdominal use at this time.  Fixation has to consider the specific flexibility and tissue integration of the device.  Quality control of outcome requires a long follow up and should use registries with standardized set of variables with an open end option for surveillance. |
| --- | --- |

When meshes are inserted intraperitoneally during laparoscopic IPOM they must meet stringent requirements because of their direct contact with the abdominal intestines. Eriksen et al (2007) formulated the following characteristics for an optimal mesh to be used for laparoscopic repair of ventral and Incisional hernias:

- Minimal adhesion formation
- Excellent tissue ingrowth
- Minimal shrinkage
- No infection or fistula formation
- Minimal pain
- Minimal seroma formation
- No change in abdominal wall compliance
- Low price
- Easy to manipulate

Typically, meshes are made of the basic materials polypropylene, polyester, polivinylidenfluoride or polytetrafluoroethylene (PTFE). The use of pure polypropylene meshes and polyester meshes are not recommended for laparoscopic IPOM (Eriksen et al. 2007, Halm et al. 2007, Shankaran et al. 2011). It is accepted in general that polypropylene and polyester meshes are coated either with a protective membrane or a protective film (absorbable or nonabsorbable) or with a titanium layer to protect the viscera. These ‘composite meshes’, as they are known, and PTFE meshes are generally recommended for intraperitoneal use (Eriksen et al. 2007, Deeken et al. 2007, Shankaran et al. 2011, Huber et al. 2011) (Tab. 1). It is assumed that when using these meshes few adhesions occur and the risk of intestinal damage and fistula formation is low.

**Clinical studies**

To date, there is a paucity of clinical case series and only one randomized trial offering general recommendations for specific meshes. Only few clinically relevant differences, which could be deemed to be clinically relevant outcome parameters, have been discerned in studies between the meshes.

In a prospective randomized trial Moreno-Egea et al. (2012) compared in laparoscopic incisional hernia repair the use of a light-weight titanium-coated mesh (n = 51) with a collagen-polyester composite mesh (n = 51). The primary end points were pain and recurrence. The secondary end points were morbidity and patient outcomes (analgesic consumption, return to everyday activities). The postoperative complication rates were similar for the two meshes. Pain was significantly less common in the titanium-coated mesh group at 1 month (p = 0,029). There was a significant differences between the two groups in the average use of analgesics in favor of the titanium-coated mesh group (p < 0,001). The titanium-coated mesh group returned to everyday activities after 6.9 days versus 9.7 days for the collagen-polyester composite mesh group (p < 0,001). The rate of recurrence did not differ between the two groups at the 2-years follow-up evaluation. The authors concluded, that the light titanium-covered polypropylene mesh was associated with less postoperative pain in the short term, lower analgesic consumption, and a quicker return to everyday activities than the Parietex composite medium-weight mesh.

In a retrospective comparative study, Colon et al. (2011) compared, on average 12 months after the operation, 116 patients who had undergone laparoscopic ventral hernia repair, of whom 66 patients received a polyester-based composite mesh and 50 patients a PTFE mesh. No significant differences were noted in terms of recurrence rate, wound complications, mesh-related infections or persistent pain.

Chelala et al (2010) reported on the intraoperative findings of 85 reoperations following laparoscopic repair of ventral and incisional hernias with the polyester-based mesh Parietex Composite®. They detected after an average of 52 months no adhesions in 47 % of cases, few adhesions in 42 % and serosa adhesions in 11 %.

Jenkins et al. (2010) presented 69 patients, who underwent laparoscopic surgery after prior intraperitoneal mesh placement for ventral hernia repair. Previous meshes were absorbable-barrier-coated mesh in 18 cases (Proceed®, Sepamesh®, C-Qur®, Parietex® Composite), permanent-barrier composite meshes in 17 cases (Composix®), permanent-barrier non composite mesh in 14 cases (DualMesh®), uncoated polypropylene mesh in 12 cases and biologic mesh in 8 cases. Indications for laparoscopic re-exploration were recurrent vental hernia (n = 58), chronic pain (n = 3), cholecystectomy (n = 3), parastomal hernia (n = 2), small bowel obstruction (n = 1), nephrectomy (n = 1) and Nissen fundoplication (n = 1). Adhesions to DualMesh were less tenacious (p < 0,05) compared to all other meshes. Surface area of adhesions to DualMesh was less (p < 0,05) then to Composix and to uncoated polypropylene mesh, but not to absorbable-barrier-coated and biologic mesh. Adhesiolysis time: mesh surface area was less (p < 0,05) for DualMesh® compared to Composix®, uncoated polyprophylene, and biologic mesh, but not to absorbable-barrier-coated mesh. Adhesiolysis-related complications occurred in two (16,7 %) (p = ns) patients with uncoated polypropylene mesh, one cystotomy and one enterotomy; both were repaired laparoscopically. There were two (16,7 %) (p = ns) conversions to an open procedure: one converted patient had Composix (6,7 %) and one had absorbable-barrier-coated mesh (5,9 %). There were no adhesiolysis-related complications with these meshes. There were no adhesiolysis-related complications or conversions to open in the DualMesh or biologic mesh groups.

Wassenaar et al. (2010) presented a series of 65 patients who had a subsequent abdominal operation after more than 1 month after laparoscopic ventral and incisional hernia repair (n = 65 / 695; 9,4 %) with DualMesh®. Only one patient required acute surgical intervention due to a laparoscopic ventral and incisional hernia repair related adhesion (0,15 %). Laparoscopy was performed in 83 % and laparotomy in 17 % of patients. Adhesions against the implant were present in 83 % of patients; in 65 %; the adhesions involved omentum only, and in 18 %, they involved the bowel. Adhesiolysis was always easy and caused no inadvertent enterotomies.

Heniford et al. (2003) reported on a consecutive series of 850 cases that had undergone laparoscopic IPOM for ventral and incisional hernias with ePTFE (Dual Mesh®). They identified a complication rate of 13.2 %. Ileus was seen in 3.0 % and long-term seroma in 2.6 %. A recurrence was noted on average after 20 months in 4.7 %.

Koehler et al. (2003) reported on 65 reoperations after laparoscopic IPOM with ePTFE (Dualmesh®). No adhesions were seen in 23 %, avascular adhesions in 68 % and stronger adhesions in 9 %.

Berger et al. (2009) reported on their experiences with 297 laparoscopic repairs of incisional hernias with polypropylene / polyvinylidenefluoride (DynaMesh®). In that series mesh-related infections occurred in 1 %, but did not result in removal of the mesh. The rate of intestinal fistulas was 0.34 %. A recurrence rate of 0.6 % was found but no mesh-related long-term complications.

As opposed to the good experiences reported by Berger et al. (2009) with DynaMesh®, Fortelny et al. (2010) reported a higher complication rate after laparoscopic IPOM repair of incisional hernias with DynaMesh®. After a follow-up examination period of one year, adhesions necessitating reintervention occurred in 5 out of 29 patients, and in 3 of out 29 cases the mesh had to be explanted, and this because of an early infection in one case.

At present, there are no other large clinical case series with usage of defined e-PTFE, PVDF or composite meshes. There is limited evidence that pure polypropylene mesh has been used without serious complications.

In 2000 Chowbey et al. reported about 202 laparoscopic ventral hernia repairs with the use of pure polypropylene meshes without given the product name. In his series, there were two postoperative hernia recurrences at a mean follow-up of 2,9 years. The incidence of seroma formation postoperatively was 32 % in the first 3 years, but declined to 18 % subsequently with postoperative abdominal-wall pressure dressings. There were no postoperative sequelae related to bowel adhesions.

Halm et al. (2006) reported about 39 cases, which underwent subsequent laparotomy / laparoscopy after prosthetic incisional hernia repair with intraperitoneal placed polypropylene meshes. The perioperative course was complicated in 76 % of procedures. Small bowel resections were necessary in 21 % of the cases. In 26 % the patients developed a surgical site infection. The authors concluded, that the intraperitoneal positioning of polypropylene mesh for incisional and ventral hernia repair should be avoided.

Though not published as yet, 60-70 % of laparoscopic ventral and incisional hernia repairs are undertaken with pure polypropylene meshes in India because of the cost and affordability issues (personal communication of M. Misra 2012).

**Experimental studies**

By means of several animal experimental studies, attempts were made to identify differences between the meshes. To that effect, investigations were conducted on both small animals and large animals. According to Penttinen et al. (2008), the closest models to surgical practice are those using large animals, swine or sheep, which allow constructions of hernias that resemble human anatomy. Based on Eriksen et al (2007), only a few experimental studies have been performed in large animals with proper mesh size and laparoscopic technique.

Conze et al. (2004) performed a study comparing heavy-weight (90g/m^2^; pore-size: 0.6mm), medium-weight (45g/m^2^, pore-size: 2.5mm) and light-weight (29g/m^2^, pore-size: 4mm) pure polypropylene meshes in laparoscopic ventral hernia repair. The heavy-weight, small porous polypropylene mesh showed significantly stronger adhesion formation. Granuloma formation was lowest in large-pore-size monofilament meshes.

Borrazzo et al (2004) compared pure polypropylene(PP) mesh, ePTFE (Dual Mesh®) and polypropylene coated on one side with a bioabsorbable adhesion barrier (Sepramesh®). The mean area of adhesion formation was 14 % in the Sepramesh®-group, 40 % in the pure PP group and 41 % in the ePTFE group. The difference between Sepramesh® and pure PP was significant (p=0.013).

Another study by Jacob et al. (2007) compared a pure polypropylene mesh with a mesh made of a polyester parietal layer and antiadhesive collagen visceral layer (Parietex Composite®) and a polypropylene soft mesh encapsulated in a polydioxanone polymer film covered by a layer of absorbable oxidized regenerated cellulose (Proceed®) . The mean area of adhesion to Parietex Composite® (11 %) was significantly less than for Proceed® (48 %; p<0.008) or pure polypropylene (46 %; p<0.008). Adhesion peel strength was significantly less for Parietex Composite® (5.9N) than for Proceed® (12.1N; p<0.02) or pure polypropylene (12.9; p<0.02).

Comparison of the composite mesh TiMesh® with titanium coating of the lightweight polypropylene and ePTFE (Dual Mesh®) showed a significantly higher shrinkage rate for ePTFE (p=0.006). Determination of the partial volume of the inflammatory cells showed significantly lower median figures for TiMesh® (p=0.009). Measurements of the proliferation marker Ki 67 showed significantly higher volumes for ePTFE (p=0.011). The apoptosis index was significantly higher for the ePTFE Mesh (p<0.002) (Schug-Pass et al. 2006).

Comparison of collagen-coated polyester (Parietex Composite®) and Composite ePTFE / polypropylene mesh (Composix®) indicates that collagen-coated polyester (Parietex Composite®) induces fewer adhesions (14.5 % vs. 53.4 %; p=0.007) (Duffy et al. 2004).

Comparison of the two composite meshes Parietex Composite® and DynaMesh® showed a significant reduction of intraabdominal adhesion formation for Parietex Composite® (Zinther et al. 2010).

Another comparison (Schug-Pass et al. 2009) of a polypropylene mesh with collagen coating (Parietene Composite®) with a polypropylene mesh with polyvinylidene fluoride on the visceral side (DynaMesh®) and a polypropylene mesh with polydioxanone and cellulose coating exhibited a markedly lower value of 12.8 % for Parietene Composite® in respect of adhesions to the greater omentum, and 31.7 % for Proceed® and 33.2 % for DynaMesh® (p=0.01). A similar value of 14 % was obtained for shrinkage of DynaMesh® and Parietene Composite®, while Proceed® showed a 25 % reduction in surface area (p=0.029 vs DynaMesh® and p=0.041 vs Parietene Composite®).

Deeken et al. (2012) compared the novel absorbable barrier coated mesh Ventrio®ST with other absorbable barrier meshes (Sepramesh® and Proceed®) and a permanent barrier mesh Ventrio®. A significant greater percent area contracture was demonstrated for Proceed® (26,9 %) compared to Ventrio® (14,5 %), to Ventrio®ST (8,8 %), and Sepramesh (9,2 %). Ventrio®ST demonstrated similiar adhesion area, tenacity and tissue ingrowth compared to all other meshes.

| **Group** | **Name of mesh** | **Material** | **Company name** |
| --- | --- | --- | --- |
| **PTFE** | | | |
|  | Mycromesh® | ePTFE | W. L. Gore |
|  | Dualmesh® | ePTFE | W. L. Gore |
|  | MotifMESH® | cPTFE | Proxy Biomedical |
|  | Omyramesh® | cPTFE | Aesculap AG |
| **PVDF** | | | |
|  | Dynamesh® | Polypropylene / polyvinyliden fluoride | FEG Fextiltechnik /  Dahlhausen |
| **Composite mesh with absorbable-barrier-coated** | | | |
|  | Glucamesh® | Polypropylene with beta glucan coat | Genzyme |
|  | Proceed® | Polypropylene with ORC layer | Ethicon |
|  | Sepramesh® | Polypropylene with resorbable layer | Genzyme |
|  | Parietene Composite® | Polypropylene with collagen-coating | Sofradim |
|  | Parietex Composite® | Polyester with collagen-coating | Sofradim |
|  | Physiomesh® | Polypropylene with poliglecaprone 25 | Ethicon |
|  | C-Qur® | Polypropylene with omega 3 fatty acid coat | Atrium Medical Corp. |
|  | Ventrio ST® | Polypropylene with PGA fibers and PDO  Filaments and hydrogel barrier | C. R. Bard |
| **Composite mesh with permanent-barrier-coated** | | | |
|  | TiMesh® | Polypropylene with titanium coat | pfm medical AG |
|  | Composix® | Polypropylene/ePTFE | C. R. Bard |
|  | Dulex® | Polypropylene/ePTFE | C. R. Bard |
|  | Ventrio® Hernia Patch | Polypropylene/ePTFE | C. R. Bard |
|  | Intramesh T1® | Polypropylene/ePTFE | Cousin Biotech |
|  | Intramesh W3® | Polyester mesh with siliconce layer | Cousin Biotech |

**Table 1**	Meshes with permission for use in the abdominal cavity modified after Eriksen et al. (2007) (1)

**References**

1.	Eriksen JR, Gögenur I, Rosenberg J

Choice of mesh for laparoscopic ventral hernia repair.

Hernia (2008) 11:481-492 **(level 4)**

2.	Shankaran V, Weber DJ, Reed, RL, Luchette FA

A Review of Available Prosthetics for Ventral Hernia Repair.

Annals of Surgery (2011), Volume 253, Number 1:16-26 **(level 4)**

3.	Halm JA, de Wall LL, Steyenberg MC, Jeekel J, Lange JF

Intraperitoneal polypropylene mesh hernia repair complicates subsequente abdominal surgery.

World J Surg (2007) Feb.; 31 (2): 423-429 **(level 4)**

4.	Deeken CR, Faucher KM, Matthews BD

A review of the composition, characteristics and effectiveness of barrier mesh protheses utilized for laparoscopic ventra hernia repair.

Surg Endosc (2011); Published online: 05 September 2011; DOI 10.1007/s00464-011-1899-3

**(level 4)**

5.	Huber A, McCabe GP, Boruch AV, Medberry C, Honerlaw, M, Badylak SF

Polypropylene-containing synthetic mesh devices in soft tissue repair: A meta-analysis.

Wiley Online Library (2011); Published online: 18 November 2011; DOI: 10.1002/jbm.b.31932

**(level 4)**

6.	Moreno-Egea A, Carrillo-Alcaraz A, Soria-Aledo V

Randomized clinical trial of laparoscopic hernia repair comparing titanium-coated lightweight mesh and medium-weight composite mesh

Surg Endosc (2012) DOI 10.1007/s00464-012-2425-y **(level 1B)**

7.	Colon MJ, Telem DA, Chin E, Weber K, Divino CM, Nguyen SQ

Polyester Composite Versus PTFE in Laparoscopic Ventral Hernia Repair

JSLS (2011); Jul-Sep; 15 (3):305-308 **(level level 3)**

8.	Chelala E, Debardemaeker Y, Elias B, Charara F, Dessily M, Allé JL

Eighty-five redo surgeries after 733 laparscopic treatments for ventral and incisional hernia: adhesion and recurrence analysis.

Hernia (2010) 14:123-129 **(level 4)**

9.	Jenkins ED, Yom V, Melman L, Brunt LM, Eagon JC, Frisella MM, Matthews BD

Prospective evaluation of adhesion characteristics to intraperitoneal mesh and adhesiolysis-related complications during laparoscopic re-exploration after prior ventral hernia repair

Surg Endosc (2010) 24:3002-3007 **(level 4)**

10.	Wassenaar EB, Schoenmaeckers EJP, Raymakers JTFJ, Rakic S

Subsequent abdominal surgery after laparoscopic ventral and incisional hernia repair with an expanded polytetrafluoroethylene mesh: a single institution experience with 72 reoperations

Hernia (2010) 14:137-142 **(level 4)**

11.	Heniford BT, Park A, Ramshaw BJ, Voeller G

Laparoscopic Repair of Ventral Hernias.

Nine Years Experience With 850 Consecutive Hernias.

Annals of Surgery (2003), Volume 238, Number 3, 391-400 **(level 4)**

12.	Koehler RH, Begons D, Berger D, Carey S, LeBlanc K, Park A, Ramshaw B, Smoot R, Voeller G

Minimal Adhesions to ePTFE Mesh After Laparoscopic Ventral Incisional Hernia Repair: Reoperative Findings in 65 Cases.

Journal of the Society of Laparoscopic Surgeons (2003) 335-340 **(level 4)**

13.	Berger D, Bientzle M

Polyvinylidene fluoride: a suitable mesh material for laparoscopic incisional and parastomale hernia repair!

Hernia (2009) 13:167-172 **(level 4)**

14.	Fortelny RH, Petter-Pucher AH, Glaser KS, Offner F, Benesch T, Rohr M

Adverse effects of polyvinylidene fluoride-coated polypropylene mesh used for laparscopic intraperitoneal onlay repair of incisional hernia

British Journal of Surgery (2010); 97:1140-1145 **(level 4)**

15.	Chowbey PK, Sharma A, Khullar R, Mann V, Baijal M, Vashistha A

Laparoscopic Ventral Hernia Repair

Journal of Laparoendoscopic & Advanced Surgical Techniques (2000); Volume 10, Number 2:79-84 **(level 4)**

16.	Halm JA, de Wall LL, Steyerberg EW, Jeekel J, Lange JF

Intraperitoneal Polypropylene Mesh Hernia Repair Complicates Subsequent Abdominal Surgery

World J Surg (2007) 31:423-429 **(level 3)**

17.	Misra M, personal communication (2012) **(level 5)**

18.	Penttinen R, Grönroos JM

Mesh repair of common abdominal hernias: a review on experimental and clinical studies.

Hernia (2008) 12:337-344 **(level 4)**

19.	Conze J, Rosch R, Klinge U, Weiss C, Anurov M, Titkowa S, Oettinger A, Schumpelick V

Polypropylene in the intra-abdominal position: Influence of pore size and surface area.

Hernia (2004) 8:365-372 **(level 5)**

20.	Borrazzo EC, Belmont MF, Boffa D, Fowler DL

Effect of prosthetic material on adhesion formation after laparoscopic ventral hernia repair in a porcine model

Hernia (2004) 8:108-112 **(level 5)**

21.	Jacob BP, Hogle NJ, Durak E, Kim T, Fowler DL

Tissue ingrowth and bowel adhesion formation in an animal comparative study: polypropylene versus Proceed versus Parietex Composite.

Surg Endosc (2007) 21:629-633 **(level 5)**

22.	Schug-Paß C, Tamme C, Tannapfel A, Köckerling F

A lightweight polypropylene mesh (TiMesh) for laparoscopic intraperitoneal repair of abdominal wall hernias

Surg Endosc (2206) 20: 402-409 **(level 5)**

23.	Duffy AJ, Hogle NJ, LaPerle KM, Fowler DL

Compüarison of two composite meshes using two fixation divices in a procine laparoscopic ventral hernia repair model

Hernia (2004) 8:358-364 **(level 5)**

24.	Zinther NB, Wara P, Friis-Andersen H

Intraperitoneal onlay mesh: an experimental study of adhesion formation in a sheep model

Hernia (2010) 14:283-289 **(level 5)**

25.	Schug-Pass C, Sommerer F, Tannapfel A, Lippert H, Köckerling F

The use of composite meshes in laparoscopic repair of abdominal wall hernias: are there differences in biocompatibily?

Surg Endosc (2009) 23:487-495 **(level 5)**

26.	Deeken CR, Matthews BD

Comparison of contracture, adhesion, tissue ingrowth, and histologic response characteristics of permanent and absorbable barrier meshes in a procine model of laparoscopic ventral hernia repair

Hernia (2011); DOI 10.1007/s10029-011-0854-5; Published online: 12. Juliy 2011

**Role of biological meshes in laparoscopic incisional and ventral hernia repair?**

**Are they advantageous in infected abdominal wall?**

B. Stechemesser, D. Weyhe, B. Ramshaw, F. Köckerling, G. S. Ferzli

Search terms: "Incisional Hernia", "Ventral Hernia", “Laparoscopic Incisional Hernia Repair", "Laparoscopic Ventral Hernia Repair", "Biological Meshes", "Meshes and Hernia Repair", "Biological Meshes and Hernia Repair".

A systematic search of the available literature was performed in July 2012 using Medline, PubMed, Cochrane Library and relevant journals and reference lists using the above listed search terms.

The first search detected 45 relevant articles. In a second - level search 1 article was added. In Summary 7 articles and studies were used for this review.

**Statements**

| Level 1B  Level 3  Level 4 | Using non-cross-linked biological meshes for elective laparoscopic bridging repair of incisional and ventral hernias shows a high recurrence rate.  Recurrence rate in elective laparoscopic repair of incisional and ventral hernias using a cross-linked acellular porcine demal collagen implant is not significantly higher compared to synthetic composite mesh.  Biological meshes are not impervious to infection.  Laparoscopic repair of incisional and ventral hernias in an infected or potentially contaminated surgical field can be performed with non-cross-linked biological meshes after closing the defect with a suture. |
| --- | --- |

**Recommendations**

| Grade A  Grade D | Elective laparoscopic repair of incisional and ventral hernias should not be performed with the use of non-cross-linked biological mesh in bridging technique.  Caution is advised to use biological meshes in a contaminated field.  Laparoscopic repair of incisional and ventral hernias in an infected or potentially contaminated surgical field with non-cross-linked biological meshes while additionally closing the defect with a suture is an option.  Elective laparoscopic repair of incisional and ventral hernias with cross-linked biological meshes is an option. |
| --- | --- |

In a systematic review of the meshes and implants available for treatment of incisional and ventral hernias by Shankaran et al (1), biological meshes are listed as a possible alternative. In this respect, biological meshes can be used in an extraperitoneal as well as an intraperitoneal position. The main advantage cited for biological meshes is their suitability for use in contaminated and infected surgical fields. Since biological meshes are revascularized and incorporated into the host tissue they provoke a markedly less pronounced foreign body reaction compared with synthetic meshes. The relatively low concentration of inflammatory cells around a biological mesh appears to explain their successful use in a contaminated field. According to Shankaran et al. (1), numerous studies have demonstrated that biological meshes can be used in contaminated fields. However, a study of the publications included in the clinical review by Shankaran et al. (1) reveals that only 6 publications were actually consulted for the review. All publications are retrospective case series. Only two of the publications explicitly focus on use in a contaminated setting. The number of cases varies between 9-75. Overall, the patient collective compared is so heterogeneous that extreme caution is advised when it comes to endorsing the statement made by Shankaran et al. (1) on the use of biological meshes in a contaminated situation.

Another systematic review from Bellows et al. (2) shows "that a paucity of high quality evidence exists in the peer-reviewed medical literature on the use of biological tissue grafts for incisional hernia repair. Although the rationale for using biological prosthesis for complex and contaminated incisional hernias is related to surgeons' concerns regarding the potential dire consequences of using permant mesh in contaminated fields, there are yet to any published prospective clinical trials justifying their preference over conventional mesh materials. Until such evidence is forthcoming, the use of biological prosthetics in complex incisional hernia repairs should proceed with caution. There may very well be a solid place for the use of these materials, but for them to add true value to complex hernia repair, better-designed and reported studies are necessary to help guide clinical practice." ( 2).

Although most xenografts are used bei surgeons in the setting of contamination, none of these biological meshes has received a U. S. Food and Drug Adminstation (FDA) indication for use in this situation (3). One particular interesting study reviewed the FDA database of adverse events associated with biological mesh. One hundred fifty adverse events were identified, with 80 % described as infection and 90 % necessitating reoperation (3,4).

**Elective laparoscopic repair of incisional and ventral hernias with biological meshes in a non-contaminated field.**

The LAPSIS study compared open retromuscular (mesh augmentation technique) with laparoscopic repair (mesh bridging technique) in a prospective randomized trial. Here the non-cross-linked Surgisis Gold® biological mesh was compared with a classic synthetic mesh. The defect sizes were between 4-10 cm. The number of cases calculated for the trial was 660. The primary target criteria were recurrence rate and reoperation rate. In a letter to the editor the study directors then announced premature termination of the trial (5). The reasons given for premature termination of the trial by the directors was too low a recruitment rate, incomplete trial data and a higher recurrence rate in the group with the biological meshes. Four years after starting the trial, only 265 patients, i.e. 40.2 % of the total number of cases, had been recruited. For 257 patients 1-year follow-up was recorded. In the laparoscopic group a recurrence rate of 19 % was noted for the biological mesh and of 5 % for the classic synthetic mesh. This was also comparable after open retromuscular augmentation (11 % vs 3 %). No significant differences were found for the other endpoints.

The conclusion drawn by the authors from the available data was that caution should be exercised when using non-cross-linked biological meshes for elective laparoscopic bridging repair of incisional and ventral hernias, if synthetic meshes could be used alternatively. Likewise, in a contaminated setting bridging of defects with biological meshes should be avoided.

In a retrospective comparative study, Cobb et al (6) compared elective laparoscopic repair of incisional and ventral hernias using a bridging technique and a composite mesh, made of polypropylene and ePTFE (Bard Composix Mesh®), with the biological mesh Permacol®. Permacol® is a cross-linked acellular porcine dermal collagen matrix. All operations were performed by a single surgeon. Eighty-four procedures were carried out with the Bard Composix Mesh® and 55 with Permacol®. In the Permacol group 15 % of procedures were conducted because of recurrences, while in the composite group 20 % of procedures were for recurrences (p = 0.655). Postoperative wound infections occurred in 3.3 % of cases in the Permacol group and in 2.4 % of the composite group. Mean follow-up in the Permacol group was 14 months and 31 months in the composite group. The recurrence rate in the Permacol group was 6.6 % and 1.2 % in the composite group and, as such, was not statistically different (p = 0.17).

The authors concluded that cross-linked acellular porcine dermal collagen was a safe alternative to composite meshes made of polypropylene and ePTFE for elective laparoscopic repair of incisional and ventral hernias using a bridging technique.

**Laparoscopic repair of incisional and ventral hernias with biological meshes in an infected or potentially contaminated field.**

In a prospective trial with 116 patients, Franklin et al (7) reported on the use of the biological mesh Surgisis® in potentially or grossly contaminated fields. All procedures were performed laparoscopically with two techniques: intraperitoneal onlay mesh (IPOM) and two-layered "sandwich" repair. Once the defect was totally freed of adhesions and had been closed with number 1 Tycra sutures whenever possible, the mesh was then introduced into the abdomen and stapled securely in place with an intracorporeal stapler. Most hernia repairs were performed by means of the intraperitoneal onlay mesh (IPOM) technique, except for three patients in whom the two-layered "sandwich" technique with laparoscopic and open implantation, with reinforcement with Surgisis anteriorly and posteriorly by laparoscopy, was used. Thirty-nine cases were carried out in an infected field and the rest in a potentially contaminated field. Ninety-one procedures were performed concurrently with a contaminated procedure. Twenty-five presented as intestinal obstruction, 16 as strangulated hernias and 17 required small bowel resection, 29 were inguinal hernias, 57 incisional and 38 umbilical hernias. In 13 patients more than two different hernias were repaired. The mean follow-up was 52± 20.9 months. Eighty-five cases were followed up for 5 years, during which 7 recurrences (6%), 11 seromas (all resolved) and 10 patients reporting mild pain were identified. Six second looks were performed and in all cases except one the mesh was found to be totally integrated into the tissue with strong scar tissue corroborated macro- and microscopically.

The authors concluded that the use of small intestine submucosa mesh (Surgisis®) in contaminated or potentially contaminated fields is a safe and feasible alternative to hernia repair with minimal recurrence rate and satisfactory results in long-term follow-up.

**References**

1. Shankaran V, Weber DJ, Reed II, L, Luchette FA

A Review of Available Prosthetics for Ventral Hernia Repair. Annals of Surgery, Volume 253, Number 1, January 2011**(level 2A)**

2. Bellows CF, Smith A, Malsbury J, Helton WS

Repair of incisional hernia with biological prosthesis: a systematic review of current evidence

The American Journal of Surgery , 2012, DOI: 10.1016/j.amjsurg.2012.02.019**(level 2A)**

3. Cavasco M, Itani KMF

Ventral Hernia Repair with Synthetic, Composite, and Biologic Mesh: Characteristics, Indication, and

Infection Profile. Surgical Infections, 2012, Volume 13: 209-215**(level 2A)**

4. Harth KC, Rosen MJ

Major Complications Associated With Xenograft Biologic Mesh Implantation in Abdominal Wall Reconstruction. Surgical Innovation 16: 324-329**(level 2C)**

5. Miserez M, G. Grass, C. Weiss, H. Stützer, S. Sauerland, Neugebauer EAM

Closure of the LAPSIS trial. British Journal of Surgery, 2010, 97, 1598**(level 1B)**

6. Cobb GA, Shaffer J

Cross-Linked Acellular Porcine Dermal Collagen Implant in Laparoscopic Ventral Hernia Repair:

Case-Controlled Study of Operative Variables and Early Complications

Int Surg, 2005, 90, 24-29**(level 3)**

7. Franklin Jr ME, Trevino JM, Portillo G, Vela I, Glass JL, Gonzalez JJ

The use of procine small intestinal submucosa as a prosthetic matieral for laparoscopic hernia repair in infected and potentially contaminated fields: long-term follow-up

Surg Endosc, 2008, 22, 19-41-1946**(level 4)**

**What happens to synthetic mesh after it is inserted into the body?**

Michael Fabian, MD, Bruce Ramshaw MD

Acknowledgement: Uwe Klinge added comments and critique

Search Terms (publications identified as pertinent to this topic/total publications returned by search): Mesh explant (0/25), materials characterization of hernia mesh (2/6), hernia mesh explant (0/9), hernia mesh interaction (0/13), hernia mesh analysis (0/39)

The search was performed in October 2011 and a total of two unique publications were returned from this search. Both were clinical studies. A secondary search revealed an additional 10 publications pertinent to this topic. Additional information on this topic was searched for on UpToDate.

**Statements**

| Level 4 | It appears that permanent synthetic (plastic) mesh used for hernia repair is not inert when placed in the patient’s body. |
| --- | --- |
| Level 4 | This biologic interaction is complex and the effects can be quite variable. |

**Recommendations:**

| Grade D | Because there is no way to predict the biologic interaction of each patient to each available hernia mesh, the patient should be informed of potential interactions and complications. The complexity and variability of the biologic interaction also would argue against the standardization of mesh within a hospital or outpatient surgery center, allowing surgeons and patients to have options between a variety of mesh choices. |
| --- | --- |

**Introduction:**
Hernia repair is one of the most common surgical procedures currently performed. There are over one million hernias repaired in the United States alone each year, and of these, over 150,000 are for incisional hernias. The vast majority of hernias are repaired with a permanent synthetic (plastic) mesh material. We are now only beginning to realize the changes that occur to the mesh and the body after placing mesh into a dynamic biologic organism.(1) The potential advantages of synthetic mesh are that mesh is accessible (easy to manufacture and maintain), consistent (materials are reproducible), durable, and cost-effective (less expensive than biological materials).
The first synthetic mesh was placed by Aquaviva in Marseille, France in 1944, and then reported widely by Dr. Francis Usher in 1958 (2,3). For over four decades, it was assumed that the mesh material remained inert after placement in the body. This analysis of current evidence will challenge that belief. Until recently, heavyweight polypropylene was by far the most commonly utilized mesh material. There are now a variety of polypropylene based meshes with varying densities and pore sizes as well as many meshes produced from other types of polymers. It should be noted that despite synthetic mesh reactions in the body based on current mesh explant analysis, most patients who have had mesh hernia repair have not developed mesh related complications.

**Research:**

In the late 1990's, and continuing in the last decade, mesh that had been explanted for a variety of reasons was studied by a number of techniques. Histological analysis, scanning electron microscope analysis, chemical analysis, infrared spectroscopy, differential scanning calorimetry, thermogravimetric analysis, and compliance testing have all been used to test and examine synthetic mesh, mostly from prior abdominal wall hernia repair, but also after pelvic floor reinforcement.(4)
The meshes have been found to undergo changes as a result of the body's defense against foreign objects, as well as complex changes due to chemical attack on the polymer structure.(5) There have also been many complications related to mesh hernia repair and the result of this mesh-body interaction may be a contributing factor to these complications. Complications related to mesh interaction with the body include recurrence due to mesh contraction and/or migration, mesh erosion into viscera and/or through skin, chronic pain, functional issues due to lack of mesh compliance, acute and delayed mesh infection, acute and chronic inflammatory reactions including chronic active seroma, and rare systemic symptoms, such as flu-like symptoms, potentially related to synthetic mesh. The variety of methods used to study mesh after explantation from the body are now presented.

**Histology:**
At the cellular level, the body will attempt to wall-off, digest or expel the foreign material. Cellular immunity is critical for survival, yet it creates problems in some (but not all) hernia patients. Polypropylene seems to have the greatest inflammatory reaction of the synthetic meshes, but this appears to decrease over time (6).
Neutrophils, lymphocytes, macrophages and foreign-body giant cells are stimulated upon injury (surgery) and implantation of mesh material. These cells release enzymes and oxidants to degrade the foreign body, in this case the mesh.(7)  Study of the mesh has shown oxidative breakdown, in addition to encasement with inflammatory cells. Lymphocytes and foreign-body giant cells are present, and these can bath the mesh in a continuous environment of oxidants, while progressively encasing the mesh in a fibrous scar that can become increasingly rigid. This may be a contributing factor to chronic and in some cases debilitating pain (8).
The foreign body response has been classified as having four distinct phases: acute inflammation, chronic inflammation, foreign body reaction with development of granulation tissue, and fibrosis (7). Heavyweight polypropylene meshes exhibit more collagen deposition and fibrosis, while lightweight meshes exhibit minimal fibrotic tissue with better neovascularization around the mesh (9).
The oxidants released by lysosomes can create superoxide anions, as well as hydrogen peroxide and hypochlorous acid. (10). Polypropylene has been shown to undergo chain scission, and overall degradation with fissures, micro cracks, build-up of hydroxyl and carbonyl groups on the surface of the material, changes in thermal properties (see below), and changes in mechanical properties such as embrittlement and reduced compliance.

There has also been discussion that the meshes generally shrink due to the above changes. But this contraction or shrinkage appears to be a very complex and irregular process. Coda et al studied multiple types of mesh and discovered that the explanted mesh pore sizes could have expanded up to 58% as well as shrunk by 40%.(11)
**Scanning Electron Microscopy:**
Most micrographs have demonstrated changes to the polypropylene mesh that include micro cracks in the transverse direction, as well as peeling of the top layer of fibers (9). Other changes included superficial or deep flaking, and fractures in the threads of varying lengths and depths (4). Interestingly, polyethylene terephthalate did not appear degraded in two separate studies (4, 12). These findings are contrary to other reports on degradation of vascular grafts and much more study of this complex biologic interaction is needed.

 Fourier Transform Infrared Spectroscopy:

 	FTIR Spectroscopy is a spectroscopic technique widely used to facilitate determination of chemical functional groups by their absorption frequency. Both Clave and Cozad in separate papers in 2010 examined multiple types of mesh.(4,13) They discovered that in virtually all types of synthetic mesh, peaks representing hydroxyl and carbonyl groups were present. This has even been noted in e-PTFE, one of the meshes thought to be the least affected by alterations.

This indicated a chemical breakdown of the “inert” mesh that has potential implications for the strength of the polymer. Many of the hydrocarbon propylenes depend on Van Der Vaals forces, and the alteration of the chemical groups can weaken these bonds. The overall effect may explain the changes in mesh seen in the tests mentioned below.

Differential Scanning Calorimetry:

This test measures melting temperature and heat of fusion in materials, and was tested in a variety of explanted meshes. This showed a shift toward lower melting temperature and broader melting peak. The clinical implication is not clear, but demonstrates a change in the physical properties of the mesh.

Thermogravimetric Analysis:

This measures weight loss of the material versus a pristine piece of mesh. This was lower for all mesh tested. This is now intuitive, as the material has been assaulted by the body, exposed to oxidative forces, and broken down chemically. This would also explain the mechanical failure of some lightweight meshes, which have been designed to lessen the host response with fibrosis and scarring, but sacrifice strength to achieve this.

**Compliance Testing**:

This measures the mean value of work to bend the mesh in half using a constant force. Nearly all materials tested, even after removing all organic material, required more work and were less compliant than the pristine control mesh. However, this compliance testing revealed tremendous variability between explant samples.(8,9).

**Summary:**

Since the early 1990’s, a diverse group of individuals, including materials engineers, chemical engineers, pathologists, device company representatives, and surgeons have made early attempts to begin to understand the changes that occur after mesh implantation in human beings. Animal experiments have not been able to show the long -term consequences of foreign body implantation into biologic organisms. The host response is variable, and we have only begun to realize the individualization that will be needed to find the best mesh for a particular cluster of individuals. There will likely be groups of patients that will have a better outcome with certain types of mesh as well as certain groups of patients who will be at risk for increased mesh-related complications with certain types of mesh. To attempt to define these groups, an evolved understanding of clinical research, based on principles of complex systems science, will likely be needed.

**References:**

1. UpToDate Jun 11, 2010. Reconstructive Materials used in surgery: Classification and host response. **(level 5)**

2. Usher, FC, Gannon JP (1959) A new plastic mesh for replacing tissue defects, experimental studies. Arch Surg 78:131**(level 5)**

3. Usher, FC, Ochsner J, Tuttle LLD Jr. (1958) Use of Marlex in incisional hernias Am Surg 24:969 **(5)**

4. Clave A, Yahi H, Hammou JC, Montanari S, Gounon P, Clave H (2010) Polypropylene as a reinforcement in pelvic surgery is not inert: comparative analysis of 100 explants. Int Urogynecol J 21:261-270 **(level 4)**

5. Klinge U, Klosterhalfen B, Muller M, Schumpelick V (1999) Foreign body reaction to meshes used for the repair of abdominal wall hernias Eur J Surg 165: 665-673 **(level 4)**

6. Klosterhalfen B, Junge K, Hermanns B, Klinge U (2002) Influence of implantation interval on the long-term biocompatibility of surgical mesh Br J Surgery 89(8): 1043-1048 **(level 4)**

7. Offner FA (2004) Pathophysiology and pathology of the foreign-body reaction to mesh implants. In: Schumpelick V, Nyhus LM, eds. *Meshes: Benefits and Risks*. New York, NY: Springer; (2004) 161-169 **(level 5)**

8. Costello CR, Bachman SL, Grant SA, Cleveland DS, Loy TS, Ramshaw BJ (2007) Characterization of heavyweight and lightweight polypropylene prosthetic mesh explants from a single patient Surg Innov 14(3) 168-176 **(level 4)**

9. Costello CR, Bachman SL, Ramshaw BJ, Grant SA (2007) Materials characterization of explanted polypropylene hernia meshes J of Biomedical Materials Research Part B: Applied Biomaterials DOI 10.1002/jbmb 44-49 **(level 4)**

10. Ratner B, Hoffman AS, Schoen FJ, Lemons JE (1996) Biomaterials Science. San Diego, CA: Academic Press 243-254 **(level 5)**

11. Coda A, Bendavid R, Botto-Micca F, Bossotti M, Bona A (2003) Structural alterations of prosthetic meshes in humans **(level 4)**

12. Bracco P, Brunella V, Trossarelli L, Coda A, Botto-Micca F (2005 Comparison of polypropylene and polyethylene terephthalate (Dacron) meshes for abdominal wall hernia repair: A chemical and morphological study Hernia 9: 51-55 **(level 4)**

13. Cozad MJ, Grant DA, Bachman SL, Grant DN, Ramshaw BJ, Grant SA (2010) Materials characterization of explanted polypropylene, polyethylene terephthalate, and expanded polytetrafluoroethylene composites: Spectral and thermal analysis. J of Biomedical Materials Research B: Applied Biomaterials 455-462 **(level 4)**

**Section 9: Hernia prophylaxis**

**Open abdominal surgery and stoma surgery**

**Indications for prophylactic mesh implantation and risk reduction strategies**

Thomas Simon, Dieter Berger

A systematic search was performed in PubMed, Medline, Cochrane, Study register, relevant journals and reference lists including publications until 6^th^ of June 2012.

Search strategy

(indic* AND prophyl* AND mesh)) OR ("Hernia, Ventral/prevention and control"[Mesh] OR "Hernia/prevention and control"[Mesh] OR “incisional hernia" AND (prevention OR prophyl*) OR “abdominal wall hernia” AND (prevention OR prophyl*) OR "Hernia, Abdominal/prevention and control"[mesh]) OR “hernia prevention” OR "hernia prophylaxis" OR "prophylactic mesh" OR “mesh implantation” OR (mesh AND “risk reduction” [tiab]) AND (randomized controlled trial [pt] OR controlled clinical trial [pt] OR randomized [tiab] OR placebo [tiab] OR clinical trials astopic [mesh: no exp] OR randomly [tiab] OR trial [ti] NOT (animals [mh] NOT humans [mh]))

The search produced 895 hits; with RCT filter 128 and Systematic Review filter 39 hits. Regarding open abdominal surgery and the indication for prophylactic mesh six relevant publications were identified, whereas two level 2a, one level 2b, one level 3, three level 4 and one experimental study were stratified. For stoma surgery and the indication for prophylactic mesh four systematic reviews and one protocol for a Cochrane review could be identified. There were 21 publications included dealing with risk reduction strategies to prevent incisional hernias.

**Statements**

| **Level 2** | Prophylactic mesh placement reduce the rate of incisional hernia in risk groups with morbid obesity or aortic aneurysm |
| --- | --- |
| **Level 1** | Prophylactic mesh placement in primary stoma formation reduce the rate of parastomal hernia without increasing morbidity, although this is based on small patient populations |
| **Level 2** | There is no relevant difference between midline and transverse incisions regarding the incidence for incisional hernia formation |
| **Level 1** | Fascia closure with a continuous suture technique using slowly-resorbable suture material reduces the incidence for incisional hernia after elective median laparotomy relevantly |
| **Level 4** | Achieving a suture length to wound length ratio equal or higher than 4 reduces the incidence of incisional hernia after midline incision significantly |

**Recommendations**

| **Grade B** | A prophylactic mesh should be placed after open abdominal surgery in risk groups with morbid obesity or aortic aneurysm |
| --- | --- |
| **Grade A** | A prophylactic mesh should be placed at the primary stoma operation, although this is based on small patient populations |
| **Grade B** | The access to the abdominal cavity can be reached via a transverse or a midline incision taking into account the surgeons preference with respect to the patient’s disease and anatomy |
| **Grade A** | After elective median laparotomy the fascia should be closed with a continuous suture technique using slowly-resorbable suture material |
| **Grade D** | An equal or higher suture length to wound length ratio than 4 should be accomplished |

**Introduction**

The incidence for incisional hernias has been reported between 5 % and 20 %, addressing it as the most common surgical complication after laparotomies^1-5^. With this burden of disease for the patient suffering from complications after surgical repair,the increasing risk for recurrence ^6^, and the economical consequences,the need for studies dealing with risk reduction strategies is obvious. For parastomal hernias, defined as an “incisional hernia related to an abdominal wall stoma”^7^, the incidence is even up to 48%. Beside technical aspects of wound closure and stoma formation, the use of biological and synthetic meshes for prophylaxis of incisional and parastomal hernia is subject of several studies.

**Indication for prophylactic mesh implantation for open abdominal surgery**

Observing the above mentioned unacceptable high incidence rates of incisional hernia for all patients undergoing laparotomy; several studies have identified two major risk groups with even higher rates. For patients with an abdominal aortic aneurysm (AAA) a meta-analysis has shown a five-fold increased risk of incisional hernia development^8^. It is assumed that a systemic connective tissue disorder in these patients may be responsible for the high rates of incisional hernias ^9^^,^^10^.
The second risk group for postoperative wound complications, especially the development of incisional hernia after laparotomy are morbidly obese patients ^11^. Other studies reported rates of postoperative hernias for obese patients up to 50 % ^12^^,^^13^.
The objective of this study is to find evidence for the use of prophylactic mesh to minimize the risk for incisional hernia.

A small case series with a prophylactic mesh placed in the preperitoneal space after open AAA repair resulted in a low rate of incisional hernia after a median follow-up time of 47 months^14^. A well conducted randomized controlled trial with a 3-year follow-up showed a significant reduction of postoperative incisional hernia after AAA repair without increasing the rate of complications, even though patients with previous abdominal surgery were not excluded^15^.

The first randomized controlled trial with long-term results after prophylactic mesh to prevent incisional hernia in obese patients could not reveal an advantage for the mesh group ^16^. However it has to be taken into account that the study group used a resorbable polyglactin mesh.

A case series with 60 patients undergoing gastric-bypass surgery and a midline incision closure with a non-resorbable polypropylene mesh demonstrated an effective prevention of incisional hernia^17^. The same group conducted a randomized controlled trial for the prophylactic use of a mesh with a mean follow-up of 28 month and found over 20 % incisional hernia in the non-mesh group and none in the mesh group ^18^. Beside the small number of patients, the missing blinding is a weakness of this study. A prospective study with no clear randomization of 100 high-risk patients (including neoplastic pathology, age over 70, respiratory failure, malnutrition, obesity and smokers) revealed as well a significant reduction of the development of incisional hernia with the use of a prophylactic polypropylene mesh ^19^. In a two institutional non-randomized prospective trial a biologic mesh was applied to one patient group after gastric bypass and showed a reduction of the incidence of incisional hernia compared with the non-mesh group in the other institution ^20^. All of the above mentioned randomized controlled trial studies show substantial weaknesses regarding study design and methods resulting in downgrading their evidence level.The on-going double blind randomized controlled multi-centre trial PRIMA conducted by Lange et al. is including both high risk groups with patients being operated for AAA or other median laparotomies with BMI over 27^21^. The recruitment process is accomplished and the publication of the trial has to be awaited.

| **Author** | **N** | **Population** | **Comments** |
| --- | --- | --- | --- |
| Pans A (1998) | 288 | Obesity | Resorbable mesh |
| Gutierez de la Pena C (2003) | 100 | Mixed | Unclear randomization |
| Strzelczyk JM (2006) | 74 | Obesity | No blinding |
| Bevis PM (2010) | 80 | AAA | Including patients with previous laparotomies |

**Tab 1: RCT studies for open abdominal surgery**

**Indication for prophylactic mesh implantation for stoma formation**

The repair of parastomal hernias results in high complication and recurrence rates ^7^^,^^22^^,^^23^. Although the approach of laparoscopic repair of parastomal hernias with intraperitoneal meshes have shown better results with recurrence rates under 12 %, the complication rates are still high ^24^^,^^25^. Hence, the prevention of the parastomal herniation by placing a prophylactic mesh in the abdominal wall at the primary operation has been subject of several studies.

The first study of prophylactic mesh implantation to prevent paracolostomal hernia formation, was published by Bayer et al in 1986^26^. The first randomized controlled trial was stopped by the authors, after the inclusion of 21 patients, due to a significant difference between the groups^27^. After a mean follow-up of 24 months, 13 of 27 patients in the non-mesh group showed a parastomal hernia whereas in the mesh group only one patient had a hernia. A systematic review including the above study and some case reports concluded that the preliminary results of a prophylactic mesh in stoma formation were promising ^28^. In the randomized study of Hammond only ileostoma-loop stomas were included and reinforced with a biologic mesh in the treatment group and with only 10 patients in each group^29^. The results of the five-year follow-up published by Janes in 2009 confirmed the initial results of the above-mentioned trial with a significant reduction of hernia rates^30^. A third randomized controlled trial was conducted by Serra-Aracil including 27 patients scheduled for permanent end colostomy surgery in each group ^31^. After a median follow-up of 29 months a hernia rate of 40,7% in the non-mesh group versus 14,8% in the treatment group was significantly lower (p<0,05) with no mesh-related complication.

Three further systematic reviews and one protocol for a Cochrane review were published, including the same three randomized controlled trials (Tam, Wijeyekoon, Shabbir)^32-35^. A total of 128 patients (Mesh=64, Non-Mesh=64) were eligible and analysed in the latest review by Shabbir et al. They conclude that despite a small patient population it could be demonstrated that the use of a prophylactic mesh at the primary stoma operation reduces the incidence of parastomal herniation with a very low specific morbidity. It is recommended to confirm these findings with large randomized controlled trials and to focus on mesh material and anatomic location.

**Risk reduction strategies**

Against the background of high incidences of incional hernias after laparotomies, efforts to reduce the risk should be taken^36^. Beside patient related risk factors, technical aspects such as suture material, suture length, suture technique and the access to the abdominal cavity are subjects of several studies.

**Access to the abdominal cavity: midline versus transverse incision**

The most commonly used incisions to gain access to the abdominal cavity for major abdominal surgery are midline or transverse incisions. Related complications and relevant outcomes are incisional hernia, wound infection and pulmonary complication. In 2005 the systematic review of the Cochrane Collaboration showed a slightly advantage for the transverse incision with respect to postoperative pain and a negative influence on pulmonary function^37^. Due to inadequate blinding, unclear randomization procedures and small sample sizes of the underlying study populations, these results are not conclusive. Comparing the one-sided transverse incision with midline incision for open cholecystectomies, the randomised controlled trial conducted by Halm et al. showed significant fewer incisional hernia for transverse incision for this selective indication ^38^. Another prospective randomized trial revealed an incidence of incisional hernia of over 90 % for midline incision compared to 40 % for transverse incision for aortic aneurysm repair ^39^. However, the results have to be seen critically with 22 patients in the midline and 15 patients in the transverse group.
The randomized controlled double-blind equivalence trial POVATI comparing both incisions revealed no significant difference regarding pain, pulmonary complications and incisional hernias after one year ^40^. Significantly more wound infections occurred in the transverse group.

**Closure technique**
Regarding wound closure techniques after laparotomies there is no consensus in the surgical community as shown in a cross-sectional cohort study ^41^. A lot of randomised controlled trials are available focusing on surgical aspects and five systematic reviews pooled the available data without defining homogenous study populations and follow-ups ^3^^,^^42-45^.
With precisely defined study populations and follow-up periods, the INLINE systematic review and meta-analysis revealed the highest available evidence ^46^. The risk for incisional hernia after elective median laparotomy is relevantly lower when closing the fascia with a continuous suture technique using slowly absorbable suture material. For emergency settings the results of the randomized controlled multicentre trial CONTINT have to be awaited ^47^.
Technical aspects of suture techniques are suture length and stich width. In a prospective trial with 363 patients after midline laparotomy in elective and emergency settings Israelsson found an overall incidence of incisional hernia of 18,7 % after 12 months ^48^. When stratified for a suture length to wound length ratio, the group with a ratio below 4 revealed an incisional hernia rate of 23.7 % whereas the group with a ratio equal or higher than 4 the incidence was 9.0 % (p>0.001). These results could be confirmed in several cohort studies ^49-51^. Subject of several studies and ongoing trials is the question of stich technique^52^^,^^53^. In an experimental study wound closure with small stiches placed 3 mm from the wound edge was stronger compared with those placed at least 10 mm^54^. Another experimental study achieved simmilar results^55^. A randomized controlled trial with 381 patients in the long stich group and 356 patients in the short stich group, the latter showed an incidence of incisional hernias 12 months after operation of 5.6 % compared with 18 % in the long stich group (p>0.001) ^56^. Furthermore, long stich length was identified as an independent risk factor for surgical site infection.The authors recommended the use of a 150 cm long USP 2-0 suture with a small needle to accomplish a suture length to wound length ratio of at least or more than four. The small needle is suggested to not allow making big stiches.
Gaining 1b evidence in the question wether the “small bite” stich technique is superior to the commonly used “big bite” technique in terms of costs and effectiveness, the ongoing randomized controlled multicentre trial ‘STICH’ was initiated ^57^.

**References**

1.	Anthony T, Bergen P, Kim L, Henderson M, Fahey T, Rege R, Turnage R (2000) Factors affecting recurrence following incisional herniorrhaphy. World journal of surgery 24**:**95-101(**Level 4)**

2.	Manninen MJ, Lavonius M, Perhoniemi VJ (1991) Results of incisional hernia repair. A retrospective study of 172 unselected hernioplasties. The European journal of surgery = Acta chirurgica 157**:**29-31(**Level 4)**

3.	van 't Riet M, Steyerberg EW, Nellensteyn J, Bonjer HJ, Jeekel J (2002) Meta-analysis of techniques for closure of midline abdominal incisions. The British journal of surgery 89**:**1350-1356(**Level 1a)**

4.	Paul A, Korenkov M, Peters S, Kohler L, Fischer S, Troidl H (1998) Unacceptable results of the Mayo procedure for repair of abdominal incisional hernias. EUROPEAN JOURNAL OF SURGERY 164**:**361-367(**Level 4)**

5.	READ R, YODER G (1989) RECENT TRENDS IN THE MANAGEMENT OF INCISIONAL HERNIATION. ARCHIVES OF SURGERY 124**:**485-488(**Level 4)**

6.	Flum DR, Horvath K, Koepsell T (2003) Have outcomes of incisional hernia repair improved with time? A population-based analysis. Annals of surgery 237**:**129-135(**Level 3)**

7.	Pearl RK (1989) Parastomal hernias. World journal of surgery 13**:**569-572(**Level 4)**

8.	Takagi H, Sugimoto M, Kato T, Matsuno Y, Umemoto T (2007) Postoperative incision hernia in patients with abdominal aortic aneurysm and aortoiliac occlusive disease: a systematic review. European journal of vascular and endovascular surgery : the official journal of the European Society for Vascular Surgery 33**:**177-181(**Level 3 )**

9.	Hall KA, Peters B, Smyth SH, Warneke JA, Rappaport WD, Putnam CW, Hunter GC (1995) Abdominal wall hernias in patients with abdominal aortic aneurysmal versus aortoiliac occlusive disease. American journal of surgery 170**:**572-575; discussion 575-576(**Level 4)**

10.	Adye B, Luna G (1998) Incidence of abdominal wall hernia in aortic surgery. American journal of surgery 175**:**400-402(**Level 4)**

11.	Christou NV, Jarand J, Sylvestre JL, McLean AP (2004) Analysis of the incidence and risk factors for wound infections in open bariatric surgery. Obesity surgery 14**:**16-22(**Level 4)**

12.	Arribas D, Elia M, Artigas C, Jimenez A, Aguilella V, Martinez M (2004) Incidence of incisional hernia following vertical banded gastroplasty. Hernia : the journal of hernias and abdominal wall surgery 8**:**135-137(**Level 4)**

13.	Sugerman HJ, Kellum JM, Jr., Reines HD, DeMaria EJ, Newsome HH, Lowry JW (1996) Greater risk of incisional hernia with morbidly obese than steroid-dependent patients and low recurrence with prefascial polypropylene mesh. American journal of surgery 171**:**80-84(**Level 4)**

14.	O'Hare JL, Ward J, Earnshaw JJ (2007) Late results of mesh wound closure after elective open aortic aneurysm repair. European journal of vascular and endovascular surgery : the official journal of the European Society for Vascular Surgery 33**:**412-413(**Level 4)**

15.	Bevis PM, Windhaber RA, Lear PA, Poskitt KR, Earnshaw JJ, Mitchell DC (2010) Randomized clinical trial of mesh versus sutured wound closure after open abdominal aortic aneurysm surgery. The British journal of surgery 97**:**1497-1502(**Level 2a)**

16.	Pans A, Elen P, Dewe W, Desaive C (1998) Long-term results of polyglactin mesh for the prevention of incisional hernias in obese patients. World journal of surgery 22**:**479-482; discussion 482-473(**Level 3)**

17.	Strzelczyk J, Czupryniak L, Loba J, Wasiak J (2002) The use of polypropylene mesh in midline incision closure following gastric by-pass surgery reduces the risk of postoperative hernia. Langenbeck's archives of surgery / Deutsche Gesellschaft fur Chirurgie 387**:**294-297(**Level 4)**

18.	Strzelczyk JM, Szymanski D, Nowicki ME, Wilczynski W, Gaszynski T, Czupryniak L (2006) Randomized clinical trial of postoperative hernia prophylaxis in open bariatric surgery. The British journal of surgery 93**:**1347-1350(**Level 2b)**

19.	Gutierrez de la Pena C, Medina Achirica C, Dominguez-Adame E, Medina Diez J (2003) Primary closure of laparotomies with high risk of incisional hernia using prosthetic material: analysis of usefulness. Hernia : the journal of hernias and abdominal wall surgery 7**:**134-136(**Level 3)**

20.	Llaguna OH, Avgerinos DV, Nagda P, Elfant D, Leitman IM, Goodman E (2011) Does prophylactic biologic mesh placement protect against the development of incisional hernia in high-risk patients? World journal of surgery 35**:**1651-1655(**Level 4)**

21.	<http://www.clinicaltrials.gov(2009)Primary_mesh_closure_of_abdominal_midline_wounds_(PRIMA)(NCT00761475).http://clinicaltrials.gov/ct2/show/record/NCT00761475?term=PRIMA&rank=3> (2009) (Expected **Level 1b)**

22.	Carne PW, Robertson GM, Frizelle FA (2003) Parastomal hernia. The British journal of surgery 90**:**784-793(**Level 4)**

23.	Rieger N, Moore J, Hewett P, Lee S, Stephens J (2004) Parastomal hernia repair. Colorectal disease : the official journal of the Association of Coloproctology of Great Britain and Ireland 6**:**203-205(**Level 4)**

24.	Hansson BM, de Hingh IH, Bleichrodt RP (2007) Laparoscopic parastomal hernia repair is feasible and safe: early results of a prospective clinical study including 55 consecutive patients. Surgical endoscopy 21**:**989-993(**Level 3)**

25.	Berger D, Bientzle M (2009) Polyvinylidene fluoride: a suitable mesh material for laparoscopic incisional and parastomal hernia repair! A prospective, observational study with 344 patients. Hernia : the journal of hernias and abdominal wall surgery 13**:**167-172(**Level 3)**

26.	Bayer I, Kyzer S, Chaimoff C (1986) A new approach to primary strengthening of colostomy with Marlex mesh to prevent paracolostomy hernia. Surgery, gynecology & obstetrics 163**:**579-580(**Level 4)**

27.	Janes A, Cengiz Y, Israelsson LA (2004) Preventing parastomal hernia with a prosthetic mesh. Archives of surgery (Chicago, Ill : 1960) 139**:**1356-1358(**Level 2a)**

28.	Helgstrand F, Gogenur I, Rosenberg J (2008) Prevention of parastomal hernia by the placement of a mesh at the primary operation. Hernia : the journal of hernias and abdominal wall surgery 12**:**577-582(**Level 2a)**

29.	Hammond TM, Huang A, Prosser K, Frye JN, Williams NS (2008) Parastomal hernia prevention using a novel collagen implant: a randomised controlled phase 1 study. Hernia : the journal of hernias and abdominal wall surgery 12**:**475-481(**Level 2b)**

30.	Janes A, Cengiz Y, Israelsson LA (2009) Preventing parastomal hernia with a prosthetic mesh: a 5-year follow-up of a randomized study. World journal of surgery 33**:**118-121; discussion 122-113(**Level 3)**

31.	Serra-Aracil X, Bombardo-Junca J, Moreno-Matias J, Darnell A, Mora-Lopez L, Alcantara-Moral M, Ayguavives-Garnica I, Navarro-Soto S (2009) Randomized, controlled, prospective trial of the use of a mesh to prevent parastomal hernia. Annals of surgery 249**:**583-587(**Level 2a)**

32.	Shabbir J, Chaudhary BN, Dawson R (2012) A systematic review on the use of prophylactic mesh during primary stoma formation to prevent parastomal hernia formation. Colorectal disease : the official journal of the Association of Coloproctology of Great Britain and Ireland 14**:**931-936(**Level 1a)**

33.	Tam KW, Wei PL, Kuo LJ, Wu CH (2010) Systematic review of the use of a mesh to prevent parastomal hernia. World journal of surgery 34**:**2723-2729(**Level 1b)**

34.	Wijeyekoon SP, Gurusamy K, El-Gendy K, Chan CL (2010) Prevention of parastomal herniation with biologic/composite prosthetic mesh: a systematic review and meta-analysis of randomized controlled trials. Journal of the American College of Surgeons 211**:**637-645(**Level 1b)**

35.	Wijeyekoon Sanjaya P, Gurusamy Kurinchi S, El-Gendy K, Chan Christopher LH, Williams Norman S (2010) Prosthetic mesh for prevention of parastomal herniation. Cochrane Database of Systematic Reviews, DOI: 10.1002/14651858.CD008905, (**Expected Level 1a)**

36.	Seiler CM, Bruckner T, Diener MK, Papyan A, Golcher H, Seidlmayer C, Franck A, Kieser M, Buchler MW, Knaebel HP (2009) Interrupted or continuous slowly absorbable sutures for closure of primary elective midline abdominal incisions: a multicenter randomized trial (INSECT: ISRCTN24023541). Annals of surgery 249**:**576-582(**Level 2a)**

37.	Brown Steven R, Tiernan J (2005) Transverse verses midline incisions for abdominal surgery. Cochrane Database of Systematic Reviews, DOI: 10.1002/14651858.CD005199.pub2, (**Level 1a)**

38.	Halm JA, Lip H, Schmitz PI, Jeekel J (2009) Incisional hernia after upper abdominal surgery: a randomised controlled trial of midline versus transverse incision. Hernia : the journal of hernias and abdominal wall surgery 13**:**275-280(**Level 2b)**

39.	Fassiadis N, Roidl M, Hennig M, South LM, Andrews SM (2005) Randomized clinical trial of vertical or transverse laparotomy for abdominal aortic aneurysm repair. The British journal of surgery 92**:**1208-1211(**Level 2b)**

40.	Seiler CM, Deckert A, Diener MK, Knaebel HP, Weigand MA, Victor N, Buchler MW (2009) Midline versus transverse incision in major abdominal surgery: a randomized, double-blind equivalence trial (POVATI: ISRCTN60734227). Annals of surgery 249**:**913-920(**Level 1b)**

41.	Rahbari NN, Knebel P, Diener MK, Seidlmayer C, Ridwelski K, Stoltzing H, Seiler CM (2009) Current practice of abdominal wall closure in elective surgery - Is there any consensus? BMC surgery 9**:**8(**Level 5)**

42.	Hodgson NC, Malthaner RA, Ostbye T (2000) The search for an ideal method of abdominal fascial closure: a meta-analysis. Annals of surgery 231**:**436-442(**Level 1b)**

43.	Weiland DE, Bay RC, Del Sordi S (1998) Choosing the best abdominal closure by meta-analysis. American journal of surgery 176**:**666-670(**Level 2b)**

44.	Rucinski J, Margolis M, Panagopoulos G, Wise L (2001) Closure of the abdominal midline fascia: meta-analysis delineates the optimal technique. The American surgeon 67**:**421-426(**Level 2b)**

45.	Gupta H, Srivastava A, Menon GR, Agrawal CS, Chumber S, Kumar S (2008) Comparison of interrupted versus continuous closure in abdominal wound repair: a meta-analysis of 23 trials. Asian journal of surgery / Asian Surgical Association 31**:**104-114(**Level 1b)**

46.	Diener MK, Voss S, Jensen K, Buchler MW, Seiler CM (2010) Elective midline laparotomy closure: the INLINE systematic review and meta-analysis. Annals of surgery 251**:**843-856(**Level 1a)**

47.	Rahbari NN, Knebel P, Kieser M, Bruckner T, Bartsch DK, Friess H, Mihaljevic AL, Stern J, Diener MK, Voss S, Rossion I, Buchler MW, Seiler CM (2012) Design and current status of CONTINT: continuous versus interrupted abdominal wall closure after emergency midline laparotomy - a randomized controlled multicenter trial [NCT00544583]. Trials 13**:**72(**Expected Level 1b)**

48.	Israelsson LA, Jonsson T (1993) Suture length to wound length ratio and healing of midline laparotomy incisions. The British journal of surgery 80**:**1284-1286(**Level 4)**

49.	Israelsson LA, Jonsson T (1996) Incisional hernia after midline laparotomy: a prospective study. The European journal of surgery = Acta chirurgica 162**:**125-129(**Level 3)**

50.	Millbourn D, Cengiz Y, Israelsson LA (2011) Risk factors for wound complications in midline abdominal incisions related to the size of stitches. Hernia : the journal of hernias and abdominal wall surgery 15**:**261-266(**Level 2a)**

51.	Gruppo M, Mazzalai F, Lorenzetti R, Piatto G, Toniato A, Ballotta E (2012) Midline abdominal wall incisional hernia after aortic reconstructive surgery: a prospective study. Surgery 151**:**882-888(**Level 3)**

52.	Millbourn D, Israelsson LA (2004) Wound complications and stitch length. Hernia : the journal of hernias and abdominal wall surgery 8**:**39-41(**Level 3)**

53.	Israelsson LA, Jonsson T (1997) Overweight and healing of midline incisions: the importance of suture technique. The European journal of surgery = Acta chirurgica 163**:**175-180(**Level 4)**

54.	Cengiz Y, Blomquist P, Israelsson LA (2001) Small tissue bites and wound strength: an experimental study. Archives of surgery (Chicago, Ill : 1960) 136**:**272-275(Level 5)

55.	Harlaar JJ, van Ramshorst GH, Nieuwenhuizen J, Ten Brinke JG, Hop WC, Kleinrensink GJ, Jeekel H, Lange JF (2009) Small stitches with small suture distances increase laparotomy closure strength. American journal of surgery 198**:**392-395

56.	Millbourn D, Cengiz Y, Israelsson LA (2009) Effect of stitch length on wound complications after closure of midline incisions: a randomized controlled trial. Archives of surgery (Chicago, Ill : 1960) 144**:**1056-1059(**Level 2a)**

57.	Harlaar JJ, Deerenberg EB, van Ramshorst GH, Lont HE, van der Borst EC, Schouten WR, Heisterkamp J, van Doorn HC, Cense HA, Berends F, Stockmann HB, Vrijland WW, Consten EC, Ottow RT, Go PM, Hermans JJ, Steyerberg EW, Lange JF (2011) A multicenter randomized controlled trial evaluating the effect of small stitches on the incidence of incisional hernia in midline incisions. BMC surgery 11**:**20(**Expected Level 1b)**

**Section 10: New technologic developments**

**Role of Single Port Surgery in Laparoscopic Hernia Repair? Place for NOTES**

**and is Robotic Surgery necessary?**

D. Lomanto

**Robotic**

**Pubmed. Embase and Medline (2003-2011)**

- KEYWORDS: Animal, Hernia, abdominal surgery/Ventral hernia, Umbilical, Incisional hernia, prosthesis implantation,Laparoscopy, Suture technique and Instrumentations, Swine, Endoscopy/methods, Endoscopy/trends, Endoscopy Gastrointestinal Methods, Surgical Procedures, Minimally invasive, Robot, Robotic Surgery, Robotic Device, Endoscopic Surgery, Laparoscopy

# Statements

| **Level 4** | (case series: i.e. studies without control group) |
| --- | --- |
|  | - Robot Assisted Ventral hernia repair is a feasible alternative to laparoscopic repair of ventral hernia. - Intracorporeal suturing under direct visualization allowed stable suture fixation of the mesh under direct visualization. - Helicoid tackers and transabdominal sutures contribute to post operative pain |

### Recommendations

| **Grade C** | - More studies must be conducted on the feasibility, practicality, costs and success of the Robotic-assisted Ventral Hernia Repair*.* |
| --- | --- |

Laparoscopic surgery requires a high degree of special resolution, dexterity, and technical skills due to the lack in depth, tactile sensation and force-feedback. New technologies have been developed to improve the ergonomics and the drawback of MIS like robotic devices. In any surgical procedure and especially in laparoscopic surgery technical skills, experience in deciding to act and manual skills as major predictors of outcomeand having surgical manipulator computer-controlled that may improve the performance and the outcome can ultimately benefits the patients (1,2) especially in procedure where the learning curve is stiff like hernia repair (3,4).Since the first successful laparoscopic repair in 1993 (5), and subsequently the advent of this surgical manipulator, many groups worldwide have tried to experience and the benefits of the use of robotic device in ventral hernia repair (6-8).

**Comments**

- Only few studies have been published to analyze the benefits of robotic devices in ventral hernia repair
- More studies must be conducted on the feasibility, practicality, costs and success of the Robotic-assisted Ventral Hernia Repair.
- Schluender S, et al, (7) showed that the robot-assisted laparoscopic repair of ventral hernia using intracorporeal suturing allowed for stable suture fixation under direct visualization and eliminated the need for tackers.
- Tayar et al (8), confirmed the benefits of the Da Vinci for intracorporeal suturing in human

**References**

1. Ramsay CR et al. Statistical assessment of the learning curves of health technologies. Health Technol Assess 2001; 5: 1-79. (4)
2. Simons MP, Aufenacker T, Bay-Nielsen M, Bouillot JL, Campanelli G, Conze J, de Lange D, Fortelny R, Heikkinen T, Kingsnorth A, Kukleta J, Morales-Conde S, Nordin P, Schumpelick V, Smedberg S, Smietanski M, Weber G, Miserez M. European Hernia Society guidelines on the treatment of inguinal hernia in adult patients. Hernia 2009; 13: 343-403. (2A)
3. Voitk AJ. The learning curve in laparoscopic inguinal hernia repair for the community general surgeon. Can J Surg 1998; 41: 446-50 (3)
4. Bencini L, Sánchez LJ: Learning curve for laparoscopic ventral hernia repair. Am J Surg 2004, 187; 378-382 (3)
5. LeBlanc KA, Booth WV. Laparoscopic repair of incisional abdominal hernias using expanded polytetrafluoroethylene: preliminary findings. Surg Laparosc Endosc. 1993;3:39–41.(3)
6. Ballantyne GH, Hourmont K, Wasielewski A. Telerobotic laparoscopic repair of incisional ventral hernias using intraperitoneal prosthetic mesh. JSLS. 2003 Jan-Mar;7(1):7-14. PubMed PMID: 12722992 (3)
7. Schluender S, Conrad J, Divino CM, Gurland B. Robot-assisted laparoscopic repair of ventral hernia with intracorporeal suturing. Surg Endosc. 2003 Sep;17(9):1391-5. Epub 2003 Jun 25. PubMed PMID: 12820058 (3)
8. Tayar C, Karoui M, Cherqui D, Fagniez PL. Robot-assisted laparoscopic mesh repair of incisional hernias with exclusive intracorporeal suturing: a pilot study. Surg Endosc. 2007 Oct;21(10):1786-9. Epub 2007 Mar 13. PubMed PMID: 17353984. (3)

**NOTES**

**Pubmed. Embase and Medline (2003-2011)**

- KEYWORDS: Animal, Colon, Hernia, Ventral, Incisional, Umbilical hernia, Prosthesis Implantation, Surgical Mesh, Surgical instrumentation, Swine, Endoscopy/methods, Endoscopy/trends, Endoscopy Gastrointestinal Methods, Surgical Procedures, Minimally invasive surgery, Natural Orifice Transluminal Endoscopic Surgery, Natural Orifice Surgery, Surgical Wound Infection/prevention, Intraperitoneal Infection, Laparoscopy

# 11 papers were relevant

# 10 papers - confidence level 4

# 1 paper - confidence level 1B

# Statements

| **Level 1 (RCT)** | - The mesh placement via NOTES is technically feasible but has a high Infection rate |
| --- | --- |
| **Level 4 (case series)** | - The risk of infection is much higher than in open or laparoscopic transabdominal ventral hernia repair; - The vaginal wall seems to be a safer entry site, compared to the gastric wall |

### Recommendations

| Grade C | - Access and development of an effective delivery device (that eliminates the contamination of the mesh through a colonized route) is necessary before trials can be started in humans. - Comparative studies are necessary to verify feasibility and success rate |
| --- | --- |

Surgery and especially endo-laparoscopic surgery has gone through a fast-pacing revolution in the last two decades. The refinement of flexible endoscopy with additional features like NBI, high-definition; the wide clinical use of robotic devices like Zeus, Da Vinci; the development of new and combined energy sealing devices like Ultracision (Ethicon Endosurgey, USA), Ligasure (covidien, USA) and lately Thunderbeat (Olympus, Japan), the use of more IT like wireless technology are completely changing the way the surgery will be performed in the near future. In 2004, a new concenpt of Natural Orifice Transluminal Endoscopic Surgery (NOTES) started fascinating all the surgeons, scientists, and industries around the world. The innovative concept of performing surgery inside the abdominal cavity accessing through natural orifice like mouth, vagina and more was an innovation (1-5).

The actual benefits of NOTES, however, has yet to be proven since most research into this exciting new field is focused on small trials involving animal models.[6] Although substantial knowledge has been gained from these studies in a relatively short time, many safety issues have to be considered especially when challenging the time honored basic surgical principle of avoiding unnecessary enterotomies by going beyond the natural borders.[7] Some human experience has been gained but is currently considered experimental and receiving much criticism and skepticism amidst the enthusiasm.[6] A review of human NOTES experiences show that so far, all have been performed under the guidance, assistance, or monitoring of concomitant laparoscopy in a hybrid setting. Multiple constraints in performance of NOTES have been identified [11]. Principally, present endoscopic systems are not designed with sufficient dexterity for NOTES procedures. Performing NOTES with today’s endoscopic instrumentations is technically difficult due to the limited endoscopic field of visualization and considerable constraints in maneuvering the instruments within the small confines of the peritoneal cavity. In NOTES, off-axis operation is often necessary. Tasks such as tissue approximation and dissection require independent coordination of two instruments approaching from different angles. But the parallelism of standard endoscopic fixtures limits the degree of freedom for optimal surgical maneuvers and does not permit much triangulationof endoscopically deployed instruments to approach surgical target. For which reasons, experimental NOTES in human have thus far focused on technically less challenging procedures. Hypothetical benefits of NOTES are: entire abdominal fascia at risk for herniation can be visualized; a reduction in port-site hernias; a better cosmetiss because of miminal or nil scar ; less pain and this especuially in Ventral hernia where different factiors may affect the healing process (12)

**Comments**

- Platform\Technology necessary to perform NOTES is still under development
- Most of the reported surgical procedures are hybrid procedures
- Comparison shold look at both simple and difficult procedures
- Delivery of a foreign body (mesh) through a colonized natural orifice may increase chronic mesh infection compared with laparoscopic techniques. Results from studies showed different conclusions; Few studies reported and increased mesh infection rate in their subjects (13-15) while another study (16-18) )showed that bacterial contamination and intra-abdominal morbidities were not encountered during surgeries when using the transvaginal approach compared to the transgastric route.
- Ventral Hernia repair using NOTES approach seems tob e safe and feasible in both experiemental and few intial report in human (12-14;18-21).

**References**

1. Kalloo AN, Singh VK, Jagannath SB, Niiyama H, Hill SL, Vaughn CA, Magee CA, Kantsevoy SV (2004). Flexible transgastric peritoneoscopy: a novel approach to diagnostic and therapeutic interventions in the peritoneal cavity. Gastrointest Endosc 60:114–117. **(level 4)**
2. McGee M, Rosen M, Marks J, et al. A primer on Natural Orifice Transluminal Endoscopic Surgery: building a new paradigm. Surg Innov 2006; 13-2: 86-93. **(level 4)**
3. Merrifield B, Wagh M, Thompson C. Peroral transgastric organ resection: a feasibility study in pigs. Gastrointest Endosc 2006;63:693-97. **(level 4)**
4. Kantsevoy SV, Hu B, Jagannath CA, et al. Transgastric endoscopic splenectomy – Is it Possible? Surg Endosc 2006; 20: 522-525. **(level 4)**
5. Wagh M, Merrifield B, Thompson C. Survival studies after endoscopic transgastric oophorectomy and tubectomy in a porcine model. Gastrointest Endosc 2006;63:473-78. **(level 4)**
6. Kalloo AN, Singh VK, Jagannath SB, et al. Flexible Transgastric Peritoneoscopy: A novel approach to diagnostic and therapeutic interventions in the peritoneal cavity. Gastrointest Endosc 2004;60:114-7. **(level 4)**
7. Rattner D, Hawes R. Notes: Gathering Momentum. Gastroint Endosc 2006;63:838-839
8. Hochberger J, Lamade W. Transgastric surgery in the the abdomen: the dawn of a new era? Gastroint Endosc 2005;62:293-296. **(level 4)**
9. Jagannath SB, Kantsevoy SV, Vaughn CA, et al. Peroral transgastric endoscopic ligation of fallopian tubes with long-term survival in a porcine model. Gastrointest Endosc 2005;61:449-53. **(level 4)**
10. Pai R, Fong D, Bundga M. Transcolonic endoscopic cholecystectomy: a NOTES survival study in a porcine model. Gastroint Endoscopy 2006;64:428-434. **(level 4)**
11. ASGE/SAGES Working Group on Natural Orifice Translumenal Endoscopic Surgery White Paper October 2005. ASGE; SAGES. Gastrointest Endosc. 2006 Feb;63(2):199-203. **(level 4)**
12. Powell B, Whang SH, Bachman SL, Astudillo JA, Sporn E, Miedema BW, Thaler K. Transvaginal repair of a large chronic porcine ventral hernia with synthetic mesh using NOTES. JSLS. 2010 Apr-Jun;14(2):234-9. **(level 4)**
13. 2. Earle DB, Desilets DJ, Romanelli JR. NOTES transgastric abdominal wall hernia repair in a porcine model. Hernia. 2010 Oct;14(5):517-22. **(level 4)**
14. 3. Jacobsen GR, Thompson K, Spivack A, Fischer L, Wong B, Cullen J, Bosia J, Whitcomb E, Lucas E, Talamini M, Horgan S. Initial experience with transvaginal incisional hernia repair. Hernia. 2010 Feb;14(1):89-91. **(level 4)**
15. 4. Buck L, Michalek J, Van Sickle K, Schwesinger W, Bingener J. Can gastric irrigation prevent infection during NOTES mesh placement? J Gastrointest Surg.2008 Nov;12(11):2010-4. **(level 4)**
16. Lomanto D, Chua HC, Myat MM, So J, Shabbir A, Ho L. Microbiological contamination during transgastric and transvaginal endoscopic techniques. J Laparoendosc Adv Surg Tech A. 2009 Aug;19(4):465-. **(level 4)**
17. 6. Miedema BW, Bachman SL, Sporn E, Astudillo JA, Thaler K. Transgastric placement of biologic mesh to the anterior abdominal wall. Surg Endosc. 2009Jun;23(6):1212-8. Epub 2009 Mar. **(level 4)**
18. Lomanto D, Dhir U, So JB, Cheah WK, Moe MA, Ho KY. Total transvaginal endoscopic abdominal wall hernia repair: a NOTES survival study. Hernia. 2009 Aug;13(4):415-9. **(level 4)**
19. Sporn E, Astudillo JA, Bachman SL, Mayfield TP, Thaler K, Miedema BW. Transgastric biologic mesh delivery and abdominal wall hernia repair in a porcine model. Endoscopy. 2009 Dec;41(12):1062-8. **(level 4)**
20. Fong DG, Ryou M, Pai RD, Tavakkolizadeh A, Rattner DW, Thompson CC. Transcolonic ventral wall hernia mesh fixation in a porcine model. Endoscopy. 2007 Oct;39(10):865-9. **(level 4)**
21. Fortelny RH, Petter-Puchner AH, Gruber-Blum S, Mika K, Brand J, Keibl C,Glaser KS, Redl H. The Feasibility of FS Mesh Fixation by a Transgastric Approach-An Important Benefit in Future NOTES Procedures? J Surg Res. 2010 Feb 4. **(level 1B)**

**Single Port Surgery**

**Pubmed. Embase and Medline (2005-2011)**

- Hernia, Ventral, Umbilcal, Incisional Hernia,Laparoscopy/methods Surgical Instruments, SILS, Single port, Surgical Mesh, SPA, Single Port Access, Surgical Mesh, laparoscopic surgery, minimally invasive surgery.

5 Papers are relevant: level 4

**Statements**

| **Level 4** | (case series) |
| --- | --- |
|  | - Single Port Access Ventral Hernia Repair appears to be safe for experienced endolaparoscopic surgeons. It may decrease parietal trauma and scarring in patients prone to incisional hernia and may be associated with a decrease in rate of port-site incisional hernia compared with multiport laparoscopy |

### Recommendations

| Grade C | - Single Port Access Ventral Hernia Repair seems to be a safe and feasible alternative option to conventional laparoscopy in selected cases but further RCTs are needed. |
| --- | --- |

In the last few years, minimally invasive surgery has continued to develop by further reducing surgical aggression and scars hence Natural Orifice Transluminal Endoscopic Surgery (NOTES) came into light. This new approach created a lot of enthusiasm but still several issues and challenges have arised and need to be resolved before a full clinical acceptance (1-3). While improving on these procedures, the idea of reducing the number and size of ports, so-called single incision access surgery came into limelight. In the beginning by using multiple fascial punctures and later using dedicated devices that were ad hoc developed and marketed. Through a small wound incision between 1.5 and 2.5 cm, the single port device can be inserted and allow multiple access for telescope and instrumentations to carried out the surgery. Early reports of different procedures have been published and the cosmetic advantage offered by the Single Port Endo-laparoscopic Surgery (SPES) make this approach attractive option for patients who require additional benefit of cosmesis. Further clinical studies involving large series of patients, are needed to confirm the benefits and advantages of SPES over standard procedure. Few case reports have been published on inguinal (4-5) and ventral hernia repair with promising results (6-10).

**Comments**

- Literatures reviewed demonstrated that the procedure is feasible, safe and reproducible.
- No intraoperative complications were observed.
- Standard instruments were used.
- Patients were discharged on the 1^st^ post op day

**References**

1. Romanelli JR, Earle DB. Single-port laparoscopic surgery: an overview. Surg Endosc. 2009 Jul;23:1419-27.**(level 5)**
2. Allemann P, Schafer M, Demartines N. Critical appraisal of single port access cholecystectomy. Br J Surg. 2010;97:1476-80.**(level 2C)**
3. Tracy CR, Raman JD, Cadeddu JA, Rane A. Laparoendoscopic single-site surgery in urology: where have we been and where are we heading? Nat Clin Pract Urol. 2008;5:561-8.**(level 5)**
4. Goo TT,Goel R, Lawenko M, Lomanto D. Laparoscopic transabdominal preperitoneal (TAPP) hernia repair via a single port. Surg Laparosc Endosc Percutan Tech. 2010;20:389-90.**(level 3)**
5. Jacob BP, Tong W, Reiner M, Vine A, Katz LB. Single incision total extraperitoneal (one SITE) laparoscopic inguinal hernia repair using a single access port device. Hernia. 2009;13:571-2.**(level 3)**
6. Bower CE, Love KM. Single incision laparoscopic ventral hernia repair. JSLS 2011 Apr-Jun;15(2):165-8. PubMed PMID: 21902968; PubMed Central PMCID: PMC3148864. **(level 3)**
7. Tsivian A, Tsivian M, Sidi AA. Laparoendoscopic Single-site Repair of Incisional Hernias After Urological Surgery. Urology. 2011 Sep;78(3):715-8. Epub 2011 Jul 8. PubMed PMID: 21741693**.(level 3)**
8. Bucher P, Pugin F, Morel P. Single-port access prosthetic repair for primary and incisional ventral hernia: toward less parietal trauma. Surg Endosc. 2011 Jun;25(6):1921-5. Epub 2010 Dec 7. **(level 3)**
9. Roberts KE, Panait L, Duffy AJ, Bell RL. Single-port laparoscopic umbilical hernia repair. Surg Innov. 2010 Sep;17(3):256-60, **(level 3)**
10. Podolsky ER, Mouhlas A, Wu AS, Poor AE, Curcillo PG 2nd. Single Port Access (SPA) laparoscopic ventral hernia repair: initial report of 30 cases. Surg Endosc. 2010 Jul;24(7):1557-61. Epub 2010 Jan 1**.(level 3)**

**Section 11: Lumbar and other unusual hernias**

**Lumbar and Unusual Hernias**

Karl A. LeBlanc,

René H. Fortelny

**Search Terms**

- Flank hernia repair, Flank hernia repair with mesh, Lumbar hernia repair, Lumbar hernia repair with mesh, Unusual hernias of the abdominal wall, Spigelian hernia, Spigelian hernia repair, Lateral incisional hernia, Traumatic lumbar hernia, Grynfelt OR Grynfelt’s hernia, Petit OR Petit’s hernia, Above AND repair, Above AND laparoscopy, Lumbar hernia AND lumbar muscles AND paralysis, Lumbar hernia AND lumbar muscles AND paralysis AND bulge, Lumbar hernia AND lumbar muscles AND paralysis AND nephrectomy, Lumbar hernia AND nephrectomy

We queried the Pub Med and Embase databases as well as the Cochrane register using the search terms noted above from the time frame of 1960-2011. (for number of articles found and used respectively see discussion)

**Statements – Lumbar Hernia**

| Level 2b | Laparoscopic repair of lumbar hernia (with mesh) is superior to open repair with mesh in terms of morbidity but not recurrence rate |
| --- | --- |
| Level 4 | There does not appear to be any distinct advantage of any method of repair of the “standard” fascial defect for lumbar hernias |

**Recommendations – Lumbar Hernia**

| Grade B | **-** Options for repair of lumbar hernias include: open repair with or without mesh in any position and laparoscopic repair with mesh in any position. However, the laparoscopic repair is preferred due to the improvement in the postoperative morbidity. |
| --- | --- |

**Statements – Spigelian Hernia**

| Level 2b | Laparoscopic repair is superior in morbidity rates and length of hospital stay |
| --- | --- |
| Level 4 | The placement of mesh is preferred either by the laparoscopic or the open method |

**Recommendations – Spieghelian Hernia**

| Grade B | The use of mesh to repair these hernias is recommended by either the laparoscopic approach but the latter method seems superior in terms of morbidity and length of stay. This represents an “upgraded” recommendation due to the clear superiority of the use of mesh for these hernias. |
| --- | --- |

**Introduction**

These two types of hernias are rather rare in the presentation to the surgeon. While most surgeons will have an opportunity to repair a Spieghelian hernia within their careers, many will never see a true lumbar hernia due to its rarity. The latter problem has increased somewhat in incidence because of the more frequent use of the lumbar approach to anterior fusion of the lumbar spine but many of these bulges are the result of intercostal nerve injury and subsequent paralysis of the flat muscles of the abdominal wall.

The first suggestion of the existence of the lumbar hernias was by Barbette in 1672 but the first publication regarding these entities was by Garangeot in 1731. It is believed that the first surgical repair of a strangulated lumbar hernia occurred in 1750 by Ravaton. However, Petit and Grynfeltt’s names are associated with these hernias rather than the other surgeons. That is because they provided the first anatomic description of the inferior lumbar space (Petit in 1783) and the superior lumbar space (Grynfeltt in 1866). The boundaries of the inferior lumbar hernia are the latissimusdorsi muscle posteriorly, the external oblique muscle anteriorly and the iliac crest inferiorly. The boundaries of the superior lumbar hernia are the 12^th^ rib superiorly, the internal oblique muscle anteriorly and the erector spinae muscle posteriorly.

Selby described traumatic acquired lumbar hernia in 1906 and Kelton noted incisional acquired lumbar hernia in 1939. Kretchmer published the first study of 11 of these latter hernias following renal surgery in 1951(1). The ratio of congenital and acquired hernias has remained stable over time, with 80% in the latter category. The etiology of the acquired defects has changed, however. Infectious etiology has declined from 17% to two percent whereas incisional hernias have increased from 10% to 31%(2) The laparoscopic approach to the repair of the lumbar hernia was first described by Burick and Parascandola in 1996(3) Currently there are many methods and meshes to repair all of these defects.

Similar to the lumbar hernias, the name of the Spieghelian hernia is credited to someone who clarified the anatomic description of the entity, Spieghel (1578-1625). This hernia occurs at the level of the semicircular line where the fascias of the oblique and transversus muscles begin to split to for the two separate layers of the abdominal musculature. Generally the overlying external oblique fascia remains intact making this herniation interstitial and more difficult to diagnose. These entities are more common than that of the lumbar hernias.

**Discussion**

Our task was to research the unusual hernias of the abdominal wall. In that effort we separated these into lumbar hernias, Spieghelian hernias, and unusually located hernias other than these two entities in the English literature. Not unexpectedly, in the latter category there was very little available information that could be used in an evidence based approach to treatment. In our search, we chose to limit the “acceptable” articles to those that included at least five cases, no case reports, and those that dealt with the repair of these hernias in some fashion.

We were also charged to investigate the unusual hernias that were located in other locations. With these we were able to identify 48 articles but all were either single case series or did not really deal with the repair of the hernia. Therefore none of these could be used for this study. There were 35 articles that were located under the “flank hernia” search but they, too, either were a case report or did not address any aspect of hernioplasty. No articles were found that dealt with the lateral bulging after a denervation injury following nephrectomy or anterior lumbar disc surgery.

Seventy-nine publications were found that described lumbar hernias or their repair. Fourteen were case series less than five cases. None were non-English publications. Two were solely anatomic descriptive articles and one was a publication that duplicated already published data. We were able to include 12 papers that included more than five patients and one prospective randomized study. There were no papers that were identified that could be used as evidentiary levels of 1A, 1B, 2A, 2C, or 3.

Moreno-Egea et al had the only prospective, non-randomized study that involved 16 patients(4). Fifteen were following nephrectomy and one occurred after trauma. Mesh was used in all of the repairs with seven done by the open method technique and nine were laparoscopic. They found that the open repair was associated with a longer operative time, a longer length of stay, higher morbidity and more recurrences. There were no recurrences in the laparoscopic group and three in the open group (p=0.4). The concluded that the laparoscopic repair was “more efficient and profitable.” This level 2B evidence supports the laparoscopic repair.

Twelve articles provided evidence at Level 4. Of these, there were six that were performed with the open technique only (5,6,7,8,9,10) Four were performed solely laparoscopically (11,12,13,14) One paper included patients that were treated both with the open or laparoscopic method (15) Of these papers, a total of 123 patients could be evaluated in all of these series. Four patients underwent a repair but the method could not be determined in the article. The method of repair of the other 119 patients are shown in Table 1.

**Table 1. Lumbar Repair – Method and Number of Patients**

| Method | Sutured repair | Preperitoneal mesh | Onlay mesh | Intraperitoneal mesh | Mesh location not stated |
| --- | --- | --- | --- | --- | --- |
| Open | 28 | 17 | 11 | 6 | 8 |
| Laparoscopic | 0 | 0 | 0 | 32 | 17 |

Of the patients listed in Table 1, only 108 had adequately follow-up described in the article. This resulted in 28 patients with an open sutured repair, 31 patients with an open repair with mesh in any location and 49 patients with a laparoscopic repair with mesh in any location. No recurrences were noted in any group of patients but the length of follow-up varied from one month to 40 months in the entire patient population. Given these results, it would appear that any method of repair for the lumbar hernia, sutured or with mesh placed by any method or location would be an acceptable operation.

Bathla et al performed a review of the literature and reported on two cases of their own (16). Their opinion was that a combined open and laparoscopic repair using transfascial sutures with or without bone anchors would be the best method to approach these difficult hernias. Stumpf et al used cadaver dissections to conclude that mesh should be used and placed in the sublay position between the internal and external oblique muscles (17)

The “Spigelian” search revealed 397 articles. Of these, the Spieghelian hernia was noted in 391 but only 95 of these described a repair of these defects with a sufficient number of patients. The “Spieghelian hernia repair AND adult” search found 263 articles. Of course, there was an overlap of a significant number of these papers. In summary, there were 95 case reports. Non- English papers that were excluded were 90. Forty-five articles dealt with the radiologic work-up or diagnosis alone. Sixteen publications were not related to our topic sufficiently to be included. Therefore, we were able to assess 16 articles that included five patients or more and described a repair of the hernia and, additionally, one study from a database. Of these 17 papers no usable data could be found at levels 1A, 1B, 2A, 2C, 3, or 5.

Only one article was found that presented a prospective randomized trial of open vs laparoscopic repair of the Spieghelian hernia (18). There were eleven patients randomized into either an open or laparoscopic repair arm. All mesh was placed in the preperitoneal space except for three in the laparoscopic group where the mesh was used in the intraperitoneal space. The laparoscopic repair was superior in incidence in postoperative morbidity (p<.05) and length of hospital stay (p<.001). The authors opined that the laparoscopic extraperitoneal repair should be the preferred approach to these hernias.

The majority of the level four evidence articles were series of patients with an open sutured repair. Several were identified that included the diagnosis and treatment of the hernia but could not be included because no morbidity or follow-up data was provided.Length of follow up varied greatly amongst the series. The cumulative data is shown in Table 2. It is rather obvious that the use of mesh is preferred. In the three series that included patients that were repaired without the use of a mesh product, the recurrence rate was 4-14%. There were no recurrence in any series that included mesh in the repair either with the open or laparoscopic technique. The mesh was placed in the intraperitoneal, extraperitoneal or intra-aponeurotic locations without the development of a recurrence.

**Table 2. Summary of Spieghelian Hernia Data**

|  | **No. of repairs** | **Open**  **Sutured** | **Open**  **Mesh** | **Lap**  **Mesh** | **Recurrence**  **Rate (%)** |
| --- | --- | --- | --- | --- | --- |
| **Artioukh** (19) | 19 | 19 |  |  | 0 |
| **Campanelli** (20) | 32 |  | 32 |  | 0 |
| **Celdrán** (21) | 9 |  | 9 |  | 0 |
| **Larson** (22) | 81 | 75 | 5 | 1 | 3/75 (4) No mesh |
| **Malazgirt** (23) | 34 |  | 34 |  | 0 |
| **Mittal** (24) | 10 |  |  | 10 | 0 |
| **Moreno-Egea**(25) | 28 (17 open but no stated if mesh was used) |  |  | 11 | 0 |
| **Mouton** (26) | 35 | 21 | 14 |  | 3/21(14)  No mesh |
| **Palanivelu** (27) | 8 |  |  | 8 | 0 |
| **Patie** (28) | 6 |  |  | 6 | 0 |
| **Saber** (29) | 8 |  |  | 8 | 0 |
| **Sanchez-Montes** (30) | 6 |  | 6 |  | 0 |
| **Singer** (31) | 8 | 8 |  |  | 0 |
| **Vos** (32) | 25 | 20 | 5 |  | 1/20(5)  No mesh |
| **Weiss** (33) | 9 | 9 |  |  | 0 |
| **TOTAL** | 318 | 152 | 105 | 44 | 7/318 (2.2) |
| **RECURRENCE RATE** | 7/318 (2.2%) | 7/152 (4.6%) | 0 | 0 |  |

**References**

1) Kretchmer HL. Hernia of the Kidney. J Urol. 1951;65:944-949.
2) Moreno-Egea A, Baena EG, Calle MC, Martınez JAT, Albasini JLA.Controversies in the Current Management of Lumbar Hernia.Arch Surg. 2007;142:82-88
3) Burick AJ, Parascandola SA. Laparoscopic repair of a traumatic lumbar hernia: a case report. J LaparoendoscSurg 1996;6:259–262

4) Moreno-Egea A,Torralba-Martinez JA,Morales G,Fernández T,Girela E,Aguayo-Albasini JL. Open vs laparoscopic repair of secondary lumbar hernias.SurgEndosc (2005) 19: 184–187. **(Level 2B)**

5) A. M. Carbonell, K. W. Kercher, L. Sigmon B. D. Matthews, R. F. Sing, J. S. Kneisl B. T. Heniford. A novel technique of lumbar hernia repair using bone anchor fixation.Hernia (2005) 9: 22–26. **(Level 4)**
6) Cavallaro G, Sadighi A, Micelli M, Burza A, Carbone G, Cavallaro A. Primary Lumbar Hernia Repair: The open approach. EurSurg Res 2007;39:88-92. **(Level 4)**
7) Hafner CD, Wylie JH, Brush BE. Petit’s lumbar hernia: repair with Marlex mesh. Arch Surg 1963 Feb;86:180-186**.(Level 4)**
8) Light HG. Hernia of the inferior lumbar space. Arch Surg 1983;118:1077-1080**.(Level 4)**
9) Alfredo Moreno-Egea, MD, Monica Mengual-Ballester, MD, Mar ́ıa Jose ́ Cases-Baldo ́, MD, and Jose ́ Luis Aguayo-Albasini, MD, Murcia.Repair of complex incisional hernias using double prosthetic repair: Single-surgeon experience with 50 cases. Surgery. 2010 Jul;148(1):140-4. **(Level 4)**
10) X. Zhou, J. O. Nve, G. Chen Lumbar hernia: Clinical analysis of 11 cases. Hernia (2004) 8: 260–263**.(Level 4)**
11) Arca MJ, Heniford BT, Pokorny R, Wilson MA, Mayes J, Gagner M. Laparoscopic repair of lumbar hernias. JACS 1998;187(2):147-152. **(Level 4)**
12) [Palanivelu C](http://www.ncbi.nlm.nih.gov/pubmed?term=%22Palanivelu%20C%22%5BAuthor%5D), [Rangarajan M](http://www.ncbi.nlm.nih.gov/pubmed?term=%22Rangarajan%20M%22%5BAuthor%5D), [John SJ](http://www.ncbi.nlm.nih.gov/pubmed?term=%22John%20SJ%22%5BAuthor%5D), [Madankumar MV](http://www.ncbi.nlm.nih.gov/pubmed?term=%22Madankumar%20MV%22%5BAuthor%5D), [Senthilkumar K](http://www.ncbi.nlm.nih.gov/pubmed?term=%22Senthilkumar%20K%22%5BAuthor%5D). Laparoscopic transperitoneal repair of lumbar incisional hernias: a combined suture and 'double-mesh' technique. Hernia. 2008 Feb;12(1):27-31**.(Level 4)**
13) N. Yavuz · Y. E. Ersoy · O. Demirkesen · O. B. Tortum · S. Erguney Laparoscopic incisional lumbar hernia repair Hernia (2009) 13:281–286. **(Level 4)**
14) Tobias-Machado M, Rincon FJ, Lasmar MT, Zambon JP, Juliano RV, Wroclawski ER. Laparoscopic surgery for treatment of incisional lumbar hernia.IntBraz J Urol 2005;31(4):309-314. **(Level 4)**
15) Alfredo Moreno-Egea, MD; Enrique G. Baena, MD; Miquel C. Calle, MD; Jose ́ Antonio T. Martınez, MD; Jose ́ Luis A. Albasini, MD.Controversies in the Current Management of Lumbar Hernias Arch Surg. 2007;142:82-88**. (Level 4)**

16) L. Bathla • E. Davies • R. J. Fitzgibbons Jr. • S. Cemaj. Timing of traumatic lumbar hernia repair: is delayed repair safe? Report of two cases and review of the literature. Hernia 2011;15(2):205-9.

17) M. Stumpf · J. Conze · A. Prescher · K. Junge · C. J. Krones · U. Klinge · V. Schumpelick. The lateral incisional hernia: anatomical considerations for a standardized retromuscularsublay repair Hernia (2009) 13:293–297.

18) Moreno-Egea A, Carrasco L, Girela E, Mart ́ın JG, Aguayo JL, Canteras M. Open vs Laparoscopic Repair of Spieghelian Hernia. A Prospective Randomized Trial. Arch Surg. 2002;137:1266-1268. **(Level 2B)**

19) Artioukh DY, Walker SJ. Spieghelianherniae: presentation, diagnosis and treatment. JRCollSurgEdinb 1996:41:241-243**.(Level 4)**
20) Campanelli G, Pettinari D, Nicolosi FM, ContessiniAvesani E. Spieghelian Hernia. Hernia 2005;9:3-5. **(Level 4)**
21) Celdrán A, Señaris J, Mañas J, Frieyro O. The open mesh repair of Spieghelian hernia. Am J Surg 2007;193:111-113**.(Level 4)**
22) Larson DW, Farley DR. Spieghelian Hernias: Repair and Outcome for 81 patients. World J Surg 2002;26:1277-1281**. (Level 4)**
23) Malazgirt Z, Topgul K, Sokmen S, ErsinS,Turkcapar AG, Gok H, Gonullu N, Paksoy M, Ertem M. Spieghelian hernias: a prospective analysis of baseline parameters and surgical outcome of 34 consecutive patients. Hernia 2006;10:326-330**.(Level 4)**
24) Mittal T, Kumar V, Khullar R, Sharma A, Soni V, Baijal M, Chowbey PK. Diagnosis and management of Spieghelian hernia: A review of literature and our experience. J Minim Access Surg 2008;4(4):95-98**.(Level 4)**
25) Moreno-Egea, Flores B, Girela E, Martin JG, Aguayo JL, Canteras M.Spieghelian hernia: bibliographical study and presentation of a series of 28 patients. Hernia 2002;6:167-170**.(Level 4)**
26) Mouton WG, Otten KT, Weiss D, Naef M, Wagner HE.Preperitoneal mesh repair in Spieghelian hernia.IntSurg 2006;91(5):262-264.
27) Palanivelu C, Vijaykumar M, Jai KV, Rajan PS, Maheshkumaar GS, Rajapandian S.Laparoscopic TransabdominalPreperitoneal Repair of Spieghelian Hernia. JSLS 2006;10:193-198.
28) Patie NM, Tantia O, Sasmal PK, Khanna S, Sen B. Laparoscopic Repair of Spieghelian Hernia: Our Experience. JSLS 2010;20(2):2010.
29) Saber AA, Elgamal MH, Rao AJ, Osmer RL, Itawi EA. Laparoscopic Spieghelian Hernia Repair: The Scroll Technique. Am Surg 2008;74(2):108-112.
30) Sanchez-Montes I, Deysine M. Spieghelian hernias: a new repair technique using preshaped polypropylene umbrella plugs. Arch Surg 1998;133(6):670-672.
31) Singer JA, Mansberger AR. Spieghelian hernia. Arch Surg 1973;107(4):515-517.
32) Vos DI, Scheltinga MRM. Incidence and outcome of surgical repair of Spighelian hernia. Br J Surg 2004;91:640-644.
33) Weiss Y, Lernau OZ, Nissan S. Spieghelian Hernia. Ann Surg 1974;180(6):836-839

**Section 12: Education**

**Education and Training in Laparoscopic Ventral Hernia Repair**

D. Lomanto

**Pubmed. Embase and Medline (2000-2011)**

- KEYWORDS: Hernia/ abdominal surgery/Ventral hernia, Umbilical, Incisional hernia, Learning curve, Education/Laparoscopy, General surgery/education, Surgical procedures/ operative education, Surgical procedures/ operative psychology, Teaching/methods, Internship/residency, Competency based education, Computer assisted instruction.

**Statements**

| **Level 1 (RCT)** | - A Structured laparoscopic training programme in hernia repair improves operator outcome in operation room |
| --- | --- |
| **Level 2C** | - Specialist centers seem to perform better than general surgical units, especially for endoscopic repairs. |
| **Level 4 (case series)** | - Positive correlation between LVHR simulator training and performance in operating room - Operative performance can be greatly affected by surgical judgment and intra-operative decision making - Surgeons with advanced laparoscopic skills are more likely to perform LVHR. Most with limited experience will begin after working with a preceptor - The Global Operative Assessment of Laparoscopic Skills –incisional hernia (GOALS-IH) is easy to use, valid and reliable for assessment of simulated LIHR - A one-day course may impacts surgeon’s practice . - 20 LVHR performed by surgeons experienced in laparoscopic surgery leads to a plateau in recurrence rates and intra-operative complications |

### Recommendations

| Grade A | In departments performing incisional/ventral hernia repair a structured laparoscopic training programme should be introduced. |
| --- | --- |
| Grade B | Complex hernia repairs should be done in specialized centers. |

| Grade C | - Laparoscopic training by virtual reality simulators may be done. - Added focus on decision making skills in LVHR significantly affect operative performance - Advanced laparoscopic skills should be acquired before mastery of LVHR - Around 20 cases should be done to reach a plateau in performance of LVHR - More studies must be conducted on the learning curve and on the best approach to integrate training in LVHR |
| --- | --- |

| Grade D | - All surgeons graduating as general surgeon should acquire a profound knowledge of the commonly performed surgical repair for conventional abdominal wall hernia repair both onlay, sublay and inlay. - Performing under supervision about 15-20 cases is ideal and necessary before a surgeon works independently. - A structured laparoscopic hernia-training program might improve surgical outcome. - Complex abdominal wall hernia surgery (multiple recurrences, chronic pain, mesh infection) should be performed by a hernia specialist. |
| --- | --- |

Medical education is undergoing a paradigm shift, from the traditional experience-based model to a program that requires documentation of proficiency (*1)*

Laparoscopic surgery requires a high degree of special resolution, dexterity, and technical skills. An initial training period is usually required for the majority of surgeons to become proficient in complex procedures by continuous repetition of these tasks (2-6)

*Clinical Outcome and Complication rates are dependent on operator experience in the procedures, surgeons who are less experienced in laparoscopic surgery and in laparoscopic ventral hernia repair will have higher complication rates.*

- Smaller scars
- Less post operative pain
- Shorter hospital stay
- Lower recurrence rates
- Fewer infectious complications comparatively with open repair
- Less overall cost

Surgeons recognize technical issues, experiences in deciding to act and manual skills as major predictors of outcome (7-8). A learning curve for a specific procedure can be evaluated by means of operative times, but mainly rate of conversions (for endo-laparoscopic surgery) and complications. In case of hernia repair, it is generally accepted that the learning curve for performing endoscopic inguinal hernia repair is longer than for open Lichtenstein repair, although the Lichtenstein technique also has a learning curve with respect to prevention of recurrence and and prevention of chronic groin pain. However, this learning curve seems to be more favourable than that for the endoscopic techniques (8-9). This is especially the case for endoscopic totally extraperitoneal repair (TEP), due to a limited working space and different appreciation of the usual anatomical landmarks seen from inside the peritoneal cavity or through an anterior approach. There appears to be a higher rate of rare but serious complications with endoscopic repair, especially during the learning curve period. Adequate patient selection and training might minimise these risks of infrequent but serious complications in the learning curve period (10-14).

Similarly for Ventral Hernia the surgical repair undergone a paradigm shift in term of repair: from simple suture repair, to mesh repair to the first successful laparoscopic repair in 1993 (15)

LVHR like any other minimally invasive procedure also offers advantages but has its own challenges: challenges of any other minimally invasive procedure, familiarity of new instruments (meshes, tackers, suture passers, etc), and familiarity of laparoscopic anatomy (though minimal for an experienced laparoscopic surgeon) (15-18). The exact definition of learning curve in laparoscopic procedures is unclear (7). The possible factors which may influence learning curve can be; surgeon’s experience with other laparoscopic procedures and instrumentation, knowledge of laparoscopic anatomy, standardization of surgical technique and stabilization of operative time and complication rate. Based on limited or no data on training, learning curve and ventral hernia repair we should suggest that a minimal training of 15-20 cases are required by an experienced laparoscopic surgeons to tackle the difficulties of the technique and achieve camparable clinical outcome in term of complications, operating time and recurrence (19-21). Supervision by an experienced surgeons may help to reduce the learning curve as suggested in several studies for other procedures including inguinal hernia repair (4,13-14).

For complex abdominal wall hernia repair these should be performed in specialist centers. These centres seem to perform better than general surgical units, especially for endoscopic repairs and complex inguinal hernia surgery (multiple recurrences, chronic pain, mesh infection…) should thus best be performed by a hernia specialist (3, 24, 32). It is unclear whether subspeciality training, center volume and/or surgeon volume are equally important to determine the outcome (22), but for many procedures, the observed associations between hospital volume and operative mortality are largely mediated by surgeon volume (23).

A structured laparoscopic training programme in hernia repair improves operator outcome in operation room and surgical outcome because allow the surgeons to learn directly from experts about the challenges encountered durig the procedures and even on how to overcome the difficulties. This followed by supervision and/or proctoring can be useful in achieving good clinical results and to shorten the learning curve. Even a one-day course may impacts surgeon’s practice especially regarding hernia repair (24-25).

In the era of IT and computer simulation, also training in ventral hernia has been positively influence by these new toys (26-28). Laparoscopic training by virtual reality simulators have shown proven benefit in terms of improved operator performance in operating room even in LVHR (29-31).

**Comments**

- Only few studies have been made to analyze learning curve
- A learning curve is needed to decrease conversions and intra-operative complications in LVHR
- Although the laparoscopic technique of ventral hernia is conceptually straight forward, the adhesiolysis requires more advanced skills.

**References**

1) Reznick RK, MacRae H: Teaching surgical skills – changes in the wind. NEJM 2006, 21;355 (25)2664-9. **(5)**
2) DeTurris SV, Cacchione RN, Mungara A, Pecoraro A, Ferzli GS. Laparoscopic herniorrhaphy: beyond the learning curve. J Am Coll Surg 2002; 194: 65-73 **(3).**
3) Edwards CC, Bailey RW. Laparoscopic hernia repair: the learning curve. Surg Laparosc Endosc Percutan Tech 2000; 10: 149-53 **(3).**
4) Fallon WF, Jr., Wears RL, Tepas JJ, III. Resident supervision in the operating room: does this impact on outcome? J Trauma 1993; 35: 556-60 **(3).**
5) Feliu-Pala X, Martin-Gomez M, Morales-Conde S, Fernandez-Sallent E. The impact of the surgeon's experience on the results of laparoscopic hernia repair. Surg Endosc 2001; 15: 1467-70 **(level 3).**
6) Miserez M, Arregui M, Bisgaard T, Huyghe M, Van Bruwaene S, Peeters E, Penninckx F. A standardized resident training program in endoscopic surgery in general and in laparoscopic totally extraperitoneal (TEP) inguinal hernia repair in particular. Surg Laparosc Endosc Percutan Tech 2009; 19: e125-e129 **(3).**
7) Ramsay CR et al. Statistical assessment of the learning curves of health technologies. Health Technol Assess 2001; 5: 1-79. **(3)..**
8) Simons MP, Aufenacker T, Bay-Nielsen M, Bouillot JL, Campanelli G, Conze J, de Lange D, Fortelny R, Heikkinen T, Kingsnorth A, Kukleta J, Morales-Conde S, Nordin P, Schumpelick V, Smedberg S, Smietanski M, Weber G, Miserez M. European Hernia Society guidelines on the treatment of inguinal hernia in adult patients. Hernia 2009; 13: 343-403. **(2C).**
9) Voitk AJ. The learning curve in laparoscopic inguinal hernia repair for the community general surgeon. Can J Surg 1998; 41: 446-50 **(3).**
10) Haidenberg J, Kendrick ML, Meile T, Farley DR. Totally extraperitoneal (TEP) approach for inguinal hernia: the favorable learning curve for trainees. Curr Surg 2003; 60: 65-8. **(3).**
11) Lau H, Patil NG, Yuen WK, Lee F. Learning curve for unilateral endoscopic totally extraperitoneal (TEP) inguinal hernioplasty. Surg Endosc 2002; 16: 1724-8. **(3).**
12) Liem MS et al. The learning curve for totally extraperitoneal laparoscopic inguinal hernia repair. Am J Surg 1996; 171: 281-5**. (3).**
13) Neumayer LA et al. Proficiency of surgeons in inguinal hernia repair: effect of experience and age. Ann Surg 2005; 242: 344-8. **(3).**
14) Robson AJ, Wallace CG, Sharma AK, Nixon SJ, Paterson-Brown S. Effects of training and supervision on recurrence rate after inguinal hernia repair. Br J Surg 2004; 91: 774-7**. (3).**
15) LeBlanc KA, Booth WV. Laparoscopic repair of incisional abdominal hernias using expanded polytetrafluoroethylene: preliminary findings. Surg Laparosc Endosc. 1993;3:39–41 **(3)..**
16) Lomanto D, Iyer S, Shabbir A, Cheah WK: Laparoscopic versus open ventral hernia repair: a prospective study. Surg Endosc 2006, 20(7);1030-5 **(2B).**
17) Heniford BT, Matthews BD, Box EA, et al: Optimal teaching environment for laparoscopic ventral herniorrhaphy. Hernia 2002, 6 (1) :17-20 **(3).**
18) Le Blanc K: Laparoscopic Incisional and Ventral Hernia: Complications – How to avoid and handle it. Hernia 2004, 8:323-31 **(3).**
19) Bencini L, Sánchez LJ: Learning curve for laparoscopic ventral hernia repair. Am J Surg 2004, 187; 378-382 **(3).**
20) Salameh JR, Sweeney JF, Graviss EA et al: Laparoscopic ventral hernia repair during the learning curve. Hernia 2002, 6(1):182-187 **(3).**
21) Arwa AL-Harazi, Vipan K, Simran S, Iwena W, Cheah WK, Lomanto D: Laparoscopic Ventral Hernia Reapir: there is a learning curve. Proceedings Asia Pacific Hernia Society Congress, Hua Hin, Thailand 2011. Abstract book pp. 21. **(3).**
22) Halm EA, Lee C, Chassin MR. Is volume related to outcome in health care? A systematic review and methodologic critique of the literature. Ann Intern Med 2002; 137: 511-20 **(1A).**
23) Birkmeyer JD et al. Surgeon volume and operative mortality in the United States. N Engl J Med 2003; 349: 2117-27. **(2C).**
24) Zerey M, Kercher KW, Sing RF, Ramshaw BJ, Voeller G, Park A, Heniford BT: Does a one-day course influence surgeon adoption of laparoscopic ventral herniorrhaphy? Surg Res. 2007 Apr;138(2):205-8. **(3)**
25) Hwang J, Telem DA, Nguyen SQ, et al: A novel laparoscopic ventral herniorrhaphy training system Surg Laparosc Endosc Percutan Tech 2010 20 (1): 16-18. **(3)**
26) Pugh C, Plachta S, Auyang E, Pryor A, Hungness E: Outcome measures for surgical simulators: is the focus on technical skills the best approach? Surgery. 2010 May;147(5):646-54 **(3)**
27) Seagull FJ, George I, Ghaderi I,et al: Surgical Abdominal Wall (SAW): A Novel Simulator for Training in Ventral Hernia Repair. Surg Innov. 2009 Dec;16(4):330-6.**(3)**
28) Jain M, Tantia O, Khanna S, et al: Hernia endotrainer: results of training on self-designed hernia trainer box. J Lap Adv Surg Tech (2009, 19:4; 535-540**(3)**
29) Vaillancourt M, Ghaderi I, Kaneva et al: LS GOALS-incisional hernia: a valid assessment of simulated laparoscopic incisional hernia repair. Surg Innov. 2011 Mar;18(1):48-54. **(3)**
30) Ghaderi I, Vaillancourt M, Sroka G, et al: Performance of simulated laparoscopic incisional hernia repair correlates with operating room performance. Am J Surg. 2011 Jan;201(1):40-5.**(3)**
31) Pugh CM, DaRosa DA, Santacaterina S, Clark RE: Faculty evaluation of simulation-based modules for assessment of intraoperative decision making. Surgery 2010, 149:4; 534-542. **(3)**

Acknowledgments:

We are very grateful to Professor Ji ZL/Nanjing, Professor Yao QY/Shanghai and Professor Wu HR/Suzhou for their outstanding hospitality and excellent organization of the Consensus Conference for development of “Guidelines for laparoscopic treatment of ventral and incisional abdominal wall hernias” within the frame of the 5^th^ Meeting of the international Endohernia Society (IEHS).”

We are very grateful to Max Healthcare Institute Ltd., Saket, New Delhi (India) for supporting open access to this publication.
