# Supplementary material for: Guidelines for laparoscopic treatment of ventral and incisional abdominal wall hernias (International Endohernia Society (IEHS)—Part 1
Source: Surg Endosc. 2013 Oct 11;28(1):2–29. doi: 10.1007/s00464-013-3170-6 (PMC3872300; doi:10.1007/s00464-013-3170-6)
Supplement: Supplementary file 2 — Supplementary material 2 (DOCX 87 kb) [file 464_2013_3170_MOESM2_ESM.docx]

**References**

1. [Fleming A](http://www.ncbi.nlm.nih.gov.medezproxy.net.ucf.edu/pubmed?term=%22Fleming%20A%22%5BAuthor%5D) (1929,1980) Classics in infectious diseases: on the antibacterial action of cultures of a penicillium, with special reference to their use in the isolation of B. influenzae by Alexander Fleming, Reprinted from the British Journal of Experimental Pathology 10:226-236, 1929. [Rev Infect Dis.](http://www.ncbi.nlm.nih.gov.medezproxy.net.ucf.edu/pubmed/6994200) Jan-Feb;2(1):129-39. **(level 5)**
2. Litynski GS (1998) Kurt Semm and the fight against skepticism: endoscopic hemostasis, laproscopic appendectomy, and Semm’s impact on the “laproscopic revolution”. JSLS 2(3):309-313. **(level 5)**

Ito T, Handa H (2012) Deciphering the mystery of thalidomide teratogenicity. Congenit Anom (Kyoto) 52(1):1-7. **(level 5)**

Ban TA (2006) The role of serendipity in drug discovery. Dialogues Clin Neurosci 8(3):335-344. **(5)**

McKusick VA (2006) A 60-year tale of spots, maps, and genes. Annu Rev Genomics Hum Genet. 7:1-27. **(level 5)**

Campbell SF (2000) Science, art and drug discovery: a personal perspective. [Clin Sci (Lond).](http://www.ncbi.nlm.nih.gov.medezproxy.net.ucf.edu/pubmed/10995589) 99(4):255-60. **(level 5)**

Elbaum-Garfinkle S (2011) Close to home: a history of Yale and Lyme disease. Yale J Biol Med. 84(2):103-108. **(level 5)**

Custers EJFM, Stuyt PMJ, De Vries Robbé PF (2000) Clinical Problem Analysis (CPA): A Systematic Approach to Teaching Complex Medical Problem Solving. Acad. Med 75: 291-297. **(level 5)**

Diez Roux AV (2011) Complex systems thinking and current impasses in health disparities research. Am J Public Health. 101(9):1627-1634. **(level 5)**

Tian Q, Price ND, Hood L (2012) Systems cancer medicine: towards realization of predictive, preventive, personalized and participatory (P4) medicine. J Intern Med. 271(2):111-121. **(level 5)**

1. Wierling C, Kuhn A, Hache H, Daskalaki A, Maschke-Dutz E, Peycheva S, Li J, Herwig R, Lehrach H (2012) Prediction in the face of uncertainty: A Monte Carlo-based approach for systems biology of cancer treatment. Mutat Res 746(2):163-70. **(level 5)**
2. Hawn MT, Snyder CW, Graham LA, Gray SH, Finan KR, Vick CC (2011). Hospital level variability in incisional hernia repair technique affects patient outcomes. Surgery. 149(2): 185-91. **(level 4)**
3. Gray SH, Vick CC, Graham LA, Finan KR, Neumayer LA, Hawn MT (2008) Variation in mesh placement for ventral hernia repair: an opportunity for process improvement? Am J Surg. 196(2):201-206. **(level 4)**
4. Jenkins ED, Yom VH, Melman L, Pierce RA, Schuessler RB, Frisella MM , Eagon JC, Brunt LM, Matthews BD (2010) Clinical predictors of operative complexity in laparoscopic ventral hernia repair: a prospective study. Surg Endosc. 24:1872-1877. **(level 4)**
5. Varnell B, Bachman S, Quick J, Vitamvas M, Ramshaw B, Oleynikov D (2008) Morbidity associated with laparoscopic repair of suprapubic hernias. Am J Surg. 196(6):983-7. **(level 4)**
6. Heniford BT, Park A, Ramshaw BJ, Voeller G (2003) Laparoscopic repair of ventral hernias: nine years' experience with 850 consecutive hernias. Ann Surg. 238(3):391-9. **(level 4)**
7. Tsereteli Z, Pryor BA, Heniford BT, Park A, Voeller G, Ramshaw BJ (2008) Laparoscopic ventral hernia repair (LVHR) in morbidly obese patients. Hernia. 12(3):233-8. **(level 4)**
8. Garcea G, Ngu W, Neal CP, Robertson GS (2012) Results from a consecutive series of laparoscopic incisional and ventral hernia repairs. Surg Laparosc Endosc Percutan Tech. 22(2):131-5. **(level 4)**
9. Baghai M, Ramshaw BJ, Smith CD, Fearing N, Bachman S, Ramaswamy A (2009) Techniques of laparoscopic ventral hernia repair can be modified to successfully repair large defects in patients with loss of domain. Surg Innov. 16(1):38-45. **(level 4)**
10. Dunne JR, Malone DL, Tracy K, Napolitano L (2003) Abdominal wall hernias: risk factors for infection and resource utilization. J of Surg Research. 111, 78-84. **(level 4)**
11. Kaafarani H, Kaufman D, Reda D, Itani K (2010) Predictors of surgical site infection in laparoscopic and open ventral incisional herniorrhaphy. J of Surg Research. 163, 229-234. **(level 4)**
12. Blatnik JA, Harth KC, Aeder MI, Rosen MJ (2011) Thirty-day readmission after ventral hernia repair: predictable or preventable? Surg Endosc. 25:1446-1451. **(level 4)**
13. Hawn MT, Gray SH, Snyder CW, Graham LA, Finan KR, Vick CC (2011) Predictors of mesh explantation after incisional hernia repair. Am J of Surg. 202., 28-33. **(level 4)**
14. Bencini L, Sanchez LJ, Bernini M, Miranda E, Farsi M, Boffi B, Moretti R (2009) Predictors of recurrence after laparoscopic ventral hernia repair. Surg Laparosc Endosc Percutan Tech. 19(2):128-32. **(level 4)**
15. Robinson TN, Clarke JH, Schoen J, Walsh MD (2005) Major mesh-related complications following hernia repair: events reported to the Food and Drug Administration. Surg Endosc. 19(12):1556-60. **(5)**
16. Schoenmaeckers E, Wassenaar EB, Raymakers Johan, Rakic S (2010) Bulging of the mesh after laparoscopic repair of ventral and incisional hernias. JSLS. 14(4):541-546. **(level 4)**
17. Killeen KL, Girad S, DeMeo JH, Shanmuganathan K, Mirvis SE. (2000) Using CT to diagnose traumatic lumbar hernia. Am. Journal of Roentgenology. 174(5):1413-15**(level 4)**
18. Palanivelu C, Rangarajan M, Jategaonkar PA, Amar V, GokulKS, Srikanth B. (2009) Laparoscopic Repair of diastasis recti using the “Venetian blinds” technique of plication with prosthetic reinforcement: a retrospectice study. Hernia 13:287-92**(level 4)**
19. Hickey NA, Ryan MF, Hamilton PA, Bloom C, Murphy JP, Brenneman F. (2002) Compute tomography of traumatic abdominal wall hernia and associated deceleration injuries. Can Assoc Radiol J. 53(3):153-9**(level 4)**
20. Rose M, Eliakim R, Bar-Ziv Y, Vromen A, Rachmilewitz D. (1994) Abdominal wall hernias. The value of computed tomography diagnosis in the obese patient. J Clin Gastroenterol. 19(2):94-6 **(level 4)**
21. Skrekas G, Stafyla VK, Papalois VE. (2005) A Grynfeltt Hernia: Report of a case. Hernia 9:188- 91**(level 5)**
22. Iannitti DA, Biz WL. (2007)Laparoscopic repair of a traumatic lumbar hernia. Hernia 11:537-40**(level 5)**
23. Habib E. (2003) Retroperitoneoscopic tension-free repair of a lumbar hernia. Hernia 7:150-52**(level 5)**
24. Habib E, Elhadad A. (2003) Spieghelian hernia long considered as diverticulitis: CT scan diagnosis and laparoscopic treatment. Surgical Endoscopy 17 (1) 159**(level 5)**
25. Gough VM, Vella M. (2009) Timely computed tomography scan diagnosis Spieghelian hernia: a case study. Ann R Coll Surg Engl. 91(8):676**(level 5)**
26. Bathla L, Davies E, Fitzgibbons RJ Jr, Cemaj S. (2011) Timing of traumatic lumbar hernia repair: is delayed repair safe? Report of two cases and review of the literature. Hernia 15(2):205-9**(level 5)**
27. Meinke AK. (2003)Totally extraperitoneal laparoendoscopic repair of lumbar hernia. Surg Endosc 17: 734-7**(level 5)**
28. Links DJR, Berney CR. (2011) Traumatic lumbar hernia repair: a laparoscopic technique for mesh fixation with an iliac crest suture anchor. Hernia 15(6) 691-3**(level 5)**
29. Yavuz N, Ersoy YE, Demirkesen O, Tortum OB, Erguney S. (2009) Laparoscopic incisional lumbar hernia repair. Hernia 13:281-6**(level 5)**
30. Gutierrez de la Pena C, Vargas Romero J, Dieguez Garcia JA. (2001)The value of CT diagnosis of hernia recurrence after prosthetic repair of ventral incisional hernias.Eur Radiol. 11(7):1161-4**(level 2b)**
31. Wagenblast AL, Kristiansen VB, Fallentin E, Schulze S. (2004) Computed tomography scanning and recurrence after laparoscopic ventral hernia repair. Surg Laparosc Endosc Percutan Tech. 14(5):254-6**(level 4)**
32. Sharma A, Mehrotra M, Khullar R, Soni V, Baijal M, Chowbey PK. (2011) Laparoscopic ventral/incisional hernia repair: a single center experience of 1242 patients over a period of 13 years. Hernia 15:131-9 **(level 4)**
33. Raftopoulos I, Courcoulas AP. (2007) Outcome of laparoscopic ventral hernia repair in morbidly obese patients with a body mass index exceeding 35kg/m2. SurgEndosc. 21(12):2293-7**(level 5)**
34. Wassenaar EB, Shoenmeckers EJP, Raymakers JTF, Rakic S. (2009) Recurrences after laparoscopic repair of ventral and incisional hernia: lessons learned from 505 repairs. Surg. Endosc. 23:825-32**(level 5)**
35. Tsomoyannis EC, Siakas P, Glantzounis G, Koulas S, Mavridou P, Gossios Kl. (2001)Seroma in laparoscopic ventral hernioplasty. Surg. Laparosc Endosc Percutan Tech. 11(5):317-21**(level 5)**
36. Gossios K, Zikou A, Vazakas P, Passas G, Glantzouni A, Glantzounis G, Kontogiannis D, Tsimoyannis E. (2003) Value of CT after laparoscopic repair of postsurgical ventral hernia. Abdom Imaging. 28(1):99-102**(level 4)**
37. Tse GH, Stuchfield BM, Duckworth AD, de Beaux AC, Tulloh B. (2010) Pseudo-recurrence following laparoscopic ventral and incisional hernia repair. Hernia 14(6):583-7**(level 4)**
38. Mussak T, FischerT, Ladurner R, Gangkofer A, Bensler S, Hallfeldt KK, Reiser M, Lienemann A. (2005) Cinemagnetic resonance imaging vs high-resolution ultrasonography for detection of adhesions after laparoscopic and open incisional hernia repair: a matched pair pilot analysis. Surg. Endosc. 19(12):1538-43**(level 2b)**
39. Fischer T, Ladurner R, Gangkofer A, Mussak T, Reiser M, Lienemann A. (2007) Functional cine MRI of the abdomen for the assessment of implanted synthetic mesh in patients after incisional hernia repair: initial results. Eur. Radiol. 17(12)3123-9**(level 4)**
40. Zinther NB, Zeuten A, Marinovskij E, Haislund M, Friis-Andersen H. (2010) Functional cine MRI and transabdominal ultrasonography for the assessment of adhesions to implanted synthetic mesh 5-7 years after laparoscopic ventral hernia repair. Hernia 14(5):499-504**(level 4)**
41. Ammaturo C, Bassi G. (2005) The ratio between anterior abdominal wall surface/wall defect surface: a new parameter to classify abdominal incisional hernias. Hernia. 9(4):316-21. **(level 4)**
42. Chevrel JP, Rath AM (2000) Classification of incisional hernias of the abdominal wall. Hernia 4:7-11. **(level 5)**
43. Chowbey PK, Khullar R, Mehrotra M, Sharma A, Soni V, Baijal M. (2006) Sir Ganga Ram Hospital classification of groin and ventral abdominal wall hernias. J Minim Access Surg. 2(3):106-9**. (level 5)**
44. Conze J, Prescher A, Kisielinski K et al. (2005) Technical consideration for subxiphoidal incisional hernia repair. Hernia 9:84-87. **(level 4)**
45. Conze J, Krones CJ, Schumpelick V, Klinge U. (2007) Incisional hernia: challenge of re-operations after mesh repair. Langenbecks Arch Surg. 392(4):453-7. **(level 4)**
46. Dietz UA, Hamelmann W, Winkler MS, Debus ES, Malafaia O, Czeczko NG, Thiede A, Kuhfuss I. (2007)An alternative classification of incisional hernias enlisting morphology, body type and risk factors in the assessment of prognosis and tailoring of surgical technique. J Plast Reconstr Aesthet Surg. 60(4):383-8. **(level 5)**
47. Dietz UA, Winkler MS, Härtel RW, Fleischhacker A, Spor L, Isbert C, Jurowich Ch, Heuschmann P, Germer CT (2012) Importance of recurrence rating, morphology, hernial gap size and risk factors in ventral and incisional hernia classification. Hernia Oct 16. [Epub ahead of print] DOI 10.1007/s 10029-012-0999-x. (level 3)
48. Hadeed JG, Walsh MD, Pappas TN, Pestana IA, Tyler DS, Levinson H, Mantyh C, Jacobs DO, Lagoo-Deenadalayan SA, Erdmann D. (2011) Complex abdominal wall hernias: a new classification system and approach to management based on review of 133 consecutive patients. Ann Plast Surg. 66(5):497-503*.* **(level 4)**
49. Höer J, Lawong G, Klinge U et al. (2002) Factors influencing the development of incisional hernia. A retrospective study of 2,983 laparotomy patients over a period of 10 years. Chirurg 73:474-480. **(level 2c)**
50. Kaafarani HM, Hur K, Hirter A et al. (2009) Seroma in ventral incisional herniorrhaphy: incidence, predictors and outcome. Am J Surg 198:639-644. **(level 2b)**
51. Kingsnorth A (2006) The management of incisional hernia. Ann R Coll Surg Engl 88:252-260. **(5)**
52. Klinge U, Si ZY, Zheng H et al. (2001) Collagen I/III and matrix metalloproteinases (MMP) 1 and 13 in the fascia of patients with incisional hernias. J Invest Surg 14:47-54. **(level 5)**
53. Korenkov M, Paul A, Sauerland S, Neugebauer E, Arndt M, Chevrel JP, Corcione F, Fingerhut A, Flament JB, Kux M, Matzinger A, Myrvold HE, Rath AM, Simmermacher RK. (2001) Classification and surgical treatment of incisional hernia. Results of an experts' meeting. Langenbecks Arch Surg. 386(1):65-73. **(level 5)**
54. LeBlanc KA, Booth WV, Whitaker JM, Bellanger DE. (2001) Laparoscopic incisional and ventral herniorraphy: our initial 100 patients. Hernia 5(1):41-5. **(level 4)**
55. Licheri S, Erdas E, Pisano G, Garau A, Ghinami E, Pomata M. (2008)Chevrel technique for midline incisional hernia: still an effective procedure. Hernia 12(2):121-6. **(level 4)**
56. Losanoff JE, Basson MD, Laker S et al. (2007) Subxiphoid incisional hernias after median sternotomy. Hernia 11:473-479. **(level 5)**
57. Martínez-Serrano MA, Pereira JA, Sancho JJ, López-Cano M, Bombuy E, Hidalgo J (2010) Study Group of Abdominal Hernia Surgery of the Catalan Society of Surgery. Risk of death after emergency repair of abdominal wall hernias. Still waiting for improvement. Langenbecks Arch Surg. 395(5):551-6. **(level 3)**
58. Moreno-Egea A, Baena EG, Calle MC, Martínez JA, Albasini JL. (2007) Controversies in the current management of lumbar hernias. Arch Surg. 142(1):82-8. **(level 5)**
59. Moreno-Egea A, Carrillo A, Aguayo JL. (2008)Midline versus non midline laparoscopic incisional hernioplasty: a comparative study. Surg Endosc. 22(3):744-9. **(level 3)**
60. Muysoms FE, Miserez M, Berrevoet F, Campanelli G, Champault GG, Chelala E, Dietz UA, Eker HH, El Nakadi I, Hauters P, Hidalgo Pascual M, Hoeferlin A, Klinge U, Montgomery A, Simmermacher RK, Simons MP, Smietański M, Sommeling C, Tollens T, Vierendeels T, Kingsnorth A. (2009)Classification of primary and incisional abdominal wall hernias. Hernia. 13(4):407-14. **(level 5)**
61. Muysoms F, Campanelli G, Champault GG, Debeaux AC, Dietz UA, Jeekel J, Klinge U, Köckerling F, Mandala V, Montgomery A, Morales Conde S, Puppe F, Simmermacher RK, Smietański M, Miserez M. (2012) EuraHS: the development of an international online platform for registration and outcome measurement of ventral abdominal wall hernia repair. Hernia. 16(3):239-50. **(level 5)**
62. Parker M, Bray JM, Pfluke JM, Asbun HJ, Smith CD, Bowers SP. (2011) Preliminary experience and development of an algorithm for the optimal use of the laparoscopic component separation technique for myofascial advancement during ventral incisional hernia repair. J Laparoendosc Adv Surg Tech A. 21(5):405-10. **(level 4)**
63. Piardi T, Audet M, Panaro F, Gheza F, Cag M, Portolani N, Cinqualbre J, Wolf P. (2010) Incisional hernia repair after liver transplantation: role of the mesh. Transplant Proc. 42(4):1244-7. **(level 4)**
64. Sanchez VM, Abi-Haidar YE, Itani KM. (2011) Mesh infection in ventral incisional hernia repair: incidence, contributing factors, and treatment. Surg Infect (Larchmt). 12(3):205-10. **(level 5)**
65. Sørensen LT, Hemmingsen UB, Kirkeby LT et al. (2005) Smoking is a risk factor for incisional hernia. Arch Surg 140:119-23. **(level 3)**
66. Veljkovic R, Protic M, Gluhovic A et al. (2010) Prospective clinical trial of factors predicting the early development of incisional hernia after midline laparotomy. J Am Coll Surg 210:210-9. **(level 4)**
67. Winkler MS, Gerharz E, Dietz UA (2008) Overview and evolving strategies of ventral hernia repair. Urologe 47(6):740-7. **(level 5)**
68. Dabbas N, Adams K, Pearson K, RoyleG. (2011)Frequency of abdominal wall hernias: is classical teaching out of date? JRSM Short Rep. 19; 2(1): 5. **.(Level 5)**
69. Seiler CM, Bruckner T, Diener MK, Papyan A, Golcher H, Seidlmayer C, Franck A, Kieser M, Büchler MW, KnaebelHP. (2009)Interrupted or continuous slowly absorbable sutures for closure of primary elective midline abdominal incisions: a multicenter randomized trial (INSECT: ISRCTN24023541). Ann Surg. 249(4): 576-82.(**Level 1b)**
70. Mudge M, Hughes LE. (1985) Incisional hernia: a 10-year prospective study of incidence and attitudes. Br J Surg. 72(1): 70-1.(**Level 4)**
71. Kingsnorth A, LeBlanc K. (2003) Hernias: inguinal and incisional. Lancet. 362(9395): 1561-71. (**Level 3)**
72. Nieuwenhuizen J, Kleinrensink GJ, Hop WC, Jeekel J, Lange JF. (2008)Indications for incisional hernia repair: an international questionnaire among hernia surgeons. Hernia. 12(3): 223-5. (**Level 5)**
73. Vardanian AJ, Farmer DG, Ghobrial RM, Busuttil RW, Hiatt JR. (2006) Incisional hernia after liver transplantation. J Am CollSurg. 203(4): 421-5. ( **Level 4)**
74. Helgstrand F, Rosenberg J, Bay-Nielsen M, Friis-Andersen H, Wara P, Jorgensen LN, Kehlet H, Bisgaard T. (2010) Establishment and initial experiences from theDanish Ventral Hernia Database. Hernia. 14(2): 131-5. (**Level 4)**
75. McEntee GP, O'Carroll A, Mooney B, Egan TJ, Delaney PV. (1989)Timing of strangulation in adult hernias. Br J Surg. 76(7): 725-6.(**Level 4)**
76. Courtney CA, Lee AC, Wilson C, O'Dwyer PJ. (2003)Ventral hernia repair: a study of current practice. Hernia. 7(1): 44-6.(**Level 4)**
77. [Hjaltason E](http://www.ncbi.nlm.nih.gov/pubmed?term=Hjaltason%20E%5BAuthor%5D&cauthor=true&cauthor_uid=7324753). (1981) Incarcerated hernia.ActaChir Scand. 147(4): 263-7.(**Level 4)**
78. [Davies M](http://www.ncbi.nlm.nih.gov/pubmed?term=Davies%20M%5BAuthor%5D&cauthor=true&cauthor_uid=17316522), [Davies C](http://www.ncbi.nlm.nih.gov/pubmed?term=Davies%20C%5BAuthor%5D&cauthor=true&cauthor_uid=17316522), Morris-Stiff G, Shute K. (2007) Emergency presentation of abdominal hernias: outcome and reasons for delay in treatment - a prospective study. Ann R CollSurgEngl 89(1): 47-50. (**Level 4)**
79. Alani A, Page B, O'Dwyer PJ. (2006)Prospective study on the presentation and outcome of patients with an acute hernia.Hernia. 10(1): 62-5.(**Level 4)**
80. Zendejas B, Kuchena A, Onkendi EO, Lohse CM, Moir CR, Ishitani MB, Potter DD, Farley DR, Zarroug AE. (2011)Fifty-three-year experience with pediatric umbilical hernia repairs.J Pediatr Surg. 46(11): 2151-6.(**Level 4)**
81. Read RC, Yoder G. (1989) Recent trends in the management of incisional herniation. ArchSurg. 124(4): 485-8.(**Level 4)**
82. Saber AA, Elgamal MH, Mancl TB, Norman E, Boros MJ. (2008)Advanced age: is it an indication or contraindication for laparoscopic ventral hernia repair? JSLS. 12(1): 46-50.(**Level 4)**
83. Nieuwenhuizen J, van Ramshorst GH, tenBrinke JG, de Wit T, van der Harst E, Hop WC, Jeekel J, Lange JF. (2011)The use of mesh in acute hernia: frequency and outcome in 99 cases. Hernia. 15(3):297-300. Epub 2011 Jan 23.(**Level 4)**
84. Bisgaard T, Kehlet H, Bay-Nielsen MB, Iversen MG, Wara P, Rosenberg J, Friis-Andersen HF, Jorgensen LN. (2009)Nationwide study of early outcomes after incisiona lhernia repair. Br J Surg. 96(12): 1452-7.(**Level 4)**
85. Nieuwenhuizen J, Halm JA, Jeekel J, Lange JF. (2007)Natural course of incisional hernia and indications for repair.Scand J Surg. 96(4): 293-6. Review**.(Level 3)**
86. Moreno-Egea A, Carrillo-Alcaraz A, Aguayo-Albasini JL. (2012) Is the outcome of laparoscopic incisional hernia repair affected by defect size? A prospective study. Am J Surg. 203(1): 87-94.(**Level 2B)**
87. Hesselink VJ, Luijendijk RW, de Wilt JH, Heide R, Jeekel J. (1993) An evaluation of risk factors in incisional hernia recurrence.SurgGynecolObstet. 176(3): 228-34.(**Level 4)**
88. Simons MP, Aufenacker T, Bay-Nielsen M, Bouillot JL, Campanelli G, Conze J, de Lange D, Fortelny R, Heikkinen T, Kingsnorth A, Kukleta J, Morales-Conde S, Nordin P, Schumpelick V, Smedberg S, Smietanski M, Weber G, Miserez M. (2009)European Hernia Society guidelines on the treatment of inguinal hernia in adult patients. Hernia. 13(4):343-403. (**Level 1A)**
89. Fitzgibbons RJ Jr, Giobbie-Hurder A, Gibbs JO, Dunlop DD, Reda DJ, McCarthy M Jr, Neumayer LA, Barkun JS, Hoehn JL, Murphy JT, Sarosi GA Jr, Syme WC, Thompson JS, Wang J, Jonasson O. (2006) Watchful waiting vs repair of inguinal hernia in minimally symptomatic men: a randomized clinical trial. 18;295(3):285-92. Erratum in: JAMA. 2006 Jun 21;295(23):2726.(**Level 1b)**
90. O’Dwyer PJ, Norrie J, Alani A, Walker A, Duffy F, Horgan P. (2006) Observation or operation for patients with an asymptomatic inguinal hernia: a randomized clinical trial. Ann Surg. 244(2):167-73.(**Level 1b)**
91. Chung L, Norrie J, O'Dwyer PJ. (2011)Long-term follow-up of patients with a painless inguinal hernia from a randomized clinical trial.Br J Surg. 98(4):596-9. doi: 10.1002/bjs.7355. Epub 2010 Nov 30.(**Level 1b)**
92. Mizrahi H, Parker MC. (2012) Management of asymptomatic inguinal hernia: a systematic review of the evidence. Arch Surg. 147(3): 277-81. Review.(**Level 1a)**
93. Lauscher JC, Rieck S, Loh JC, Gröne J, Buhr HJ, Ritz JP. (2011) Oligosymptomatic vs. symptomatic incisional hernias--who benefits from open repair? Langenbecks Arch Surg. 396(2): 179-85. Epub 2010 Jun 28.(**Level 3)**
94. Lauscher JC, Martus P, Stroux A, Neudecker J, Behrens U, Hammerich R, Buhr HJ, Ritz JP. (2012)The Development of a clinical trial to determine whether watchful waiting is an acceptable alternative to surgical repair for patients with oligosymptomatic incisional hernia: study protocol for a randomized controlled trial. Trials. 13:14**.(expected 1B)**
95. Bellows C. Watchful waiting of incisional hernias. ClinicalTrials.gov NCT00351455**.(expected 1B)**
96. Sauerland S, Walgenbach M, Habermalz B, Seiler CM, Miserez M. (2000) Laparoscopic versus open surgical techniques for ventral or incisional hernia repair.[Cochrane Database Syst Rev](http://www.ncbi.nlm.nih.gov/pubmed/21412910) 2011 Mar 16;(3): CD007781. (**Level 1a)**
97. Abdel-Baki NA, Bessa SS, Abdel-Razek AH. (2007)Comparison of prosthetic mesh repair and tissue repair in the emergency management of incarcerated para-umbilical hernia: a prospective randomized study. Hernia. 11(2):163-7. Epub 2007 Feb 2. (**level** **Ib)**
98. Arroyo A, García P, Pérez F, Andreu J, Candela F, Calpena R. (2001) Randomized clinical trial comparing suture and mesh repair of umbilical hernia in adults. Br J Surg. 88(10):1321-3.  **(level Ib)**
99. Asencio F, Aguiló J, Peiró S, Carbó J, Ferri R, Caro F, Ahmad M. (2009) Open randomized clinical trial of laparoscopic versus open incisional hernia repair. Surg Endosc 23(7):1441–1448. [PUBMED: 19116750] **(level** **Ib)**
100. Aslani N, Brown CJ. (2010) Does mesh offer an advantage over tissue in the open repair of umbilical hernias? A systematic review and meta-analysis. Hernia. 14(5):455-62. Epub 2010 Jul 16. Review. PMID: 20635190 **(level** **Ia)**
101. Barbaros U, Asogulu O, Seven R, Erbil Y, Dinccag A, Deveci U, Ozarmagan S, Mercan S. (2006) The comparison of laparoscopic and open ventral hernia repairs : a prospective randomized study. Hernia 11(1):51–56. [PUBMED: 17131072] **(level** **Ib )**
102. den Hartog D, Dur AHM, Tuinebreijer WE, Kreis RW (2008) Open surgical procedures for incisional hernias. Cochrane Database Syst Rev (3):CD006438 Meta-analysis. **(level** **Ia)**
103. Forbes SS, Eskicioglu C, McLeod RS, Okrainec A. (2009) Meta-analysis of randomized controlled trials comparing open and laparoscopic ventral and incisional hernia repair with mesh. Br J Surg. 96(8):851-8. Review. PMID: 19591158 **(level** **Ia)**
104. Goodney PP, Birkmeyer JD. (2002)Short term outcomes of laparoscopic and open ventral hernia repair: a meta-analysis. Archives of Surgery 137(10):1161–5. [PUBMED: 12361426] **(level** **Ia)**
105. Polat C, Dervisoglu A, Senyurek G, Bilgin M, Erzurumlu K, Ozkan K. (2005) Umbilical hernia repair with the prolene hernia system. Am J Surg. 190(1):61-4.  **(level II b)**
106. Kapischke M, Schulz T, Schipper T, Tensfeld J, Caliebe A. (2008)Open versus laparoscopic incisional hernia repair: something different from a meta-analysis. Surgical Endoscopy 22(10):2251–60. [PUBMED: 18320281] **(level** **Ia)**
107. Moreno-Egea A, Carrasco L, Girela E, Martín JG, Aguayo JL, Canteras M. (2002) Open vs laparoscopic repair of Spieghelian hernia: a prospective randomized trial. Archives of Surgery 137(11): 1266–8. [PUBMED: 12413315] RCT **(level Ib)**
108. Misra MC, Bansal VK, Kulkarni MP, Pawar DK. (2006) Comparison of laparoscopic and open repair of incisional and primary ventral hernia: results of a prospective randomized study. Surg Endosc 20(12):1839–45. [PUBMED: 17063290] **(level Ib** )
109. Navarra G, Musolino C, De Marco ML, Bartolotta M, Barbera A, Centorrino T. (2007) Retromuscular sutured incisional hernia repair: a randomized controlled trial to compare open and laparoscopic approach. Surgical Laparoscopy and Endoscopy 17(2):86–90. [PUBMED: 17450086] **(level** **Ib)**
110. Olmi S, Scaini A, Cesana GC, Erba L, Croce E. (2007)Laparoscopic versus open incisional hernia repair: an open randomized controlled study. Surg Endosc 21(4):555–9. [PUBMED: 17364151] **(level** **Ib)**
111. Pring CM, Tran V, O’Rourke N, Martin IJ. (2008) Laparoscopic versus open ventral hernia repair: a randomized controlled trial. Australian and New Zealand Journal of Surgery 78(10):903–6. [PUBMED: 18959646] **( level** **Ib)**
112. Itani KM, Hur K, Kim LT, Anthony T, Berger DH, Reda D, Neumayer L for the Veterans Affairs Ventral Incisional Hernia Investigators. (2010) Comparison of laparoscopic and open repair with mesh for the treatment of ventral incisional hernia: a randomized trial. Archives of Surgery 145(4):322–8. [PUBMED:20404280] **(level** **Ib)**
113. Korenkov M, Sauerland S, Arndt M, Bograd L, Neugebauer EA, Troidl H. (2002) Randomized clinical trial of suture repair, polypropylene mesh or autodermal hernioplasty for incisional hernia. Br J Surg. 89(1):50-6. **(level** **IIa)**
114. Eryilmaz R, Sahin M, Tekelioglu MH. (2006) Which repair in umbilical hernia of adults: primary or mesh? Int Surg. 91(5):258-61. **(level IIb)**
115. Wright BE, Beckerman J, Cohen M, Cumming JK, Rodriguez JL. (2002) Is laparoscopic umbilical hernia repair with mesh a reasonable alternative to conventional repair? Am J Surg. 184(6):505-8; discussion 508-9. PMID:12488148**( level** **III)**
116. Sanjay P, Reid TD, Davies EL, Arumugam PJ, Woodward A. (2005) Retrospective comparison of mesh and sutured repair for adult umbilical hernias. Hernia 9(3):248-51. Epub 2005 May 13. PMID:15891810 **(level III)**
117. Arroyo A, Pérez F, Serrano P, Costa D, Oliver I, Ferrer R, Lacueva J, Calpena R. (2002) Is prosthetic umbilical hernia repair bound to replace primary herniorrhaphy in the adult patient? Hernia. 6(4):175-7. Epub 2002 Oct 19. PMID: 18267162 **(level** **III)**
118. Thoman DS. (2001/2002) Randomized clinical trial comparing suture and mesh repair of umbilical hernia in adults Br J Surg 88:1321-3 ; Br J Surg. 89(5):627; author reply 628.
119. Vrijland WW, Jeekel J. (2003) Prosthetic mesh repair should be used for any defect in the abdominal wall. Curr Med Res Opin. 19(1):1-3. **(level IV)**
120. Stabilini C, Stella M, Frascio M, De Salvo L, Fornaro R, Larghero G, Mandolfino F, Lazzara F, Gianetta E. (2009) Mesh versus direct suture for the repair of umbilical and epigastric hernias. Ten-year experience. Ann Ital Chir. 80(3):183-7. **(level III)**
121. Farrow B, Awad S, Berger DH, Albo D, Lee L, Subramanian A, Bellows CF. (2008) More than 150 consecutive open umbilical hernia repairs in a major Veterans Administration Medical Center. Am J Surg. 196(5):647-51. **(level** **III)**
122. Ergul Z, Ersoy E, Kulacoglu H, Olcucuoglu E, Devay AO, Gundogdu H. (2009)A simple modified technique for repair of umbilical hernia in patients undergo laparoscopic cholecystectomy. Report of 10 cases. G Chir. 30(10):437-9. **(level** **IV)**
123. Kamer E, Unalp HR, Derici H, Tansug T, Onal MA. (2007)Laparoscopic cholecystectomy accompanied by simultaneous umbilical hernia repair: a retrospective study. J Postgrad Med. 53(3):176-80. **( level** **III)**
124. Lau H, Patil NG (2003) Umbilical hernia in adults. Surg Endosc 17:2016–2020 **(level** **III)**
125. Asolati M, Huerta S, Sarosi G, Harmon R, Bell C, Anthony T. (2006) Predictors of recurrence in veteran patients with umbilical hernia: single center experience. Am J Surg 192:627–630 **(level** **IV)**
126. Bowley DMG, Kingsnorth AN (2000) Umbilical hernia, Mayo or mesh? Hernia 4:195–196 **(level** **IV)**
127. Halm JA, Heisterkamp J, Veen HF, Weidema WF (2005) Long term follow-up after umbilical hernia repair: are there risk factors for recurrence after simple and mesh repair. Hernia 9:334–337 **(level** **III)**
128. Venclauskas L, Silanskaite J, Kiudelis M (2008) Umbilical hernia: factors indicative of recurrence. Medicina (Kaunas, Lithuania) 44:855–859 **(level** **III)**
129. Solomon TA, Wignesvaran P, Chaudry MA, Tutton MG. (2010) A retrospective audit comparing outcomes of open versus laparoscopic repair of umbilical/paraumbilical herniae. Surg Endosc. 24(12):3109-12. Epub 2010 May 20. PMID: 20490566 **(level**  **III)**
130. Malik AM, Jawaid A, Talpur AH, Laghari AA, Khan A. J Ayub. (2008) Mesh versus non-mesh repair of ventral abdominal hernias. Med Coll Abbottabad. 20(3):54-6.PMID: 19610517
131. Martin DF, Williams RF, Mulrooney T, Voeller GR. (2008) Ventralex mesh in umbilical/epigastric hernia repairs: clinical outcomes and complications. Hernia. 12(4):379-83. Epub 2008 Feb 29. PMID: 18309451 **(level** **IV)**
132. Gonzalez R, Mason E, Duncan T, Wilson R, Ramshaw BJ. (2003) Laparoscopic versus open umbilical hernia repair. JSLS. 7(4):323-8. PMID: 14626398 **(level** **III)**
133. Franklin ME Jr, Gonzalez JJ Jr, Glass JL, Manjarrez A. (2004) Laparoscopic ventral and incisional hernia repair: an 11-year experience. Hernia 8(1):23–7. [PUBMED: 14505237] **(level** **IV)**
134. Lomanto D, Iyer SG, Shabbir A, Cheah WK. (2006) Laparoscopic versus open ventral hernia mesh repair: a prospective study. Surgical Endoscopy 20(7):1030–5. [PUBMED: 16703430**]( level** **IIb)**
135. Dur A, den Hartog D, Tuinebreijer WE, Kreis RW, Lange JF. (2009) Low recurrence rate of a two-layered closure repair for primary and recurrent midline incisional hernia without mesh. Hernia 13:421–426 DOI 10.1007/s10029-009-0487-0 **(level** **III)**
136. Itani KM, Neumayer L, Reda D, Kim L, Anthony T (2004) Repair of ventral incisional hernia: the design of a randomized trial to compare open and laparoscopic surgical techniques. Am J Surg 188(6A suppl):22S–29S.**(level** **lb)**
137. Itani K, Hur K, Kim L, Thomas A, Berger D, Reda D, Neumayer L. (2009) Ventral incisional hernia repair: comparison of laparoscopic and open repair with mesh . Hernia 13(Suppl 1):S36. [: NCT00240188]
138. Burger JW, Luijendijk RW, Hop WC, Halm JA, Verdaasdonk EG, Jeekel J. (2004) Long-term follow-up of a randomized controlled trial of suture versus mesh repair of incisional hernia. Ann Surg. 240(4):578-83; discussion 583-5.**(level** **lb)**
139. Schumacher OP, Peiper C, Lörken M, Schumpelick V(2003). Long-term results after Spitzy's umbilical hernia repair. Chirurg. 74(1):50-4. **(level** **III)**
140. Halm JA, de Wall LL, Steyerberg EW, Jeekel J, Lange JF (2007) Intraperitoneal polypropylene mesh hernia repair complicates subsequent abdominal surgery. World J Surg 31**:**423-429; discussion 430 **(level 4)**
141. Ferrari GC, Miranda A, Di Lernia S, Sansonna F, Magistro C, Maggioni D, Scandroglio I, Costanzi A, Franzetti M, Pugliese R (2008) Laparoscopic repair of incisional hernia: Outcomes of 100 consecutive cases comprising 25 wall defects larger than 15 cm. Surg Endosc 22**:**1173-1179 **(level 4)**
142. LeBlanc KA, Whitaker JM, Bellanger DE, Rhynes VK (2003) Laparoscopic incisional and ventral hernioplasty: lessons learned from 200 patients. Hernia 7**:**118-124 **(level 4)**
143. Perrone JM, Soper NJ, Eagon JC, Klingensmith ME, Aft RL, Frisella MM, Brunt LM (2005) Perioperative outcomes and complications of laparoscopic ventral hernia repair. Surgery 138**:**708-715; discussion 715-706 **(level 4)**
144. Raftopoulos I, Vanuno D, Khorsand J, Ninos J, Kouraklis G, Lasky P (2002) Outcome of laparoscopic ventral hernia repair in correlation with obesity, type of hernia, and hernia size. J Laparoendosc Adv Surg Tech A 12**:**425-429 **(level 4)**
145. Novitsky YW, Cobb WS, Kercher KW, Matthews BD, Sing RF, Heniford BT (2006) Laparoscopic ventral hernia repair in obese patients: a new standard of care. Arch Surg 141**:**57-61 **(level 4)**
146. Muysoms F, Daeter E, Vander Mijnsbrugge G, Claeys D (2004) Laparoscopic intraperitoneal repair of incisional and ventral hernias. Acta Chir Belg 104**:**705-708 **(level 4)**
147. Gananadha S, Samra JS, Smith GS, Smith RC, Leibman S, Hugh TJ (2008) Laparoscopic ePTFE mesh repair of incisional and ventral hernias. ANZ J Surg 78**:**907-913 **(level 4)**
148. Ferrari GC, Miranda A, Sansonna F, Magistro C, Di Lernia S, Maggioni D, Franzetti M, Pugliese R (2008) Laparoscopic management of incisional hernias > or = 15 cm in diameter. Hernia 12**:**571-576 **(level 4)**
149. Carbajo MA, del Olmo JC, Blanco JI, de la Cuesta C, Martin F, Toledano M, Perna C, Vaquero C (2000) Laparoscopic treatment of ventral abdominal wall hernias: preliminary results in 100 patients. JSLS 4**:**141-145 **(level 4)**
150. Park A, Gagner M, Pomp A (1996) Laparoscopic repair of large incisional hernias. Surg Laparosc Endosc 6**:**123-128 **(level 4)**
151. Bower CE, Reade CC, Kirby LW, Roth JS (2004) Complications of laparoscopic incisional-ventral hernia repair: the experience of a single institution. Surg Endosc 18**:**672-675 **(level 4)**
152. Bamehriz F, Birch DW (2004) The feasibility of adopting laparoscopic incisional hernia repair in general surgery practice: early outcomes in an unselected series of patients. Surg Laparosc Endosc Percutan Tech 14**:**207-209 **(level 4)**
153. Birgisson G, Park AE, Mastrangelo MJ, Jr., Witzke DB, Chu UB (2001) Obesity and laparoscopic repair of ventral hernias. Surg Endosc 15**:**1419-1422 **(level 4)**
154. Kurmann A, Visth E, Candinas D, Beldi G (2011) Long-term follow-up of open and laparoscopic repair of large incisional hernias. World J Surg 35**:**297-301 **(3)**
155. Ballem N, Parikh R, Berber E, Siperstein A (2008) Laparoscopic versus open ventral hernia repairs: 5 year recurrence rates. Surg Endosc 22**:**1935-1940 **(level 4)**
156. Raftopoulos I, Vanuno D, Khorsand J, Kouraklis G, Lasky P (2003) Comparison of open and laparoscopic prosthetic repair of large ventral hernias. JSLS 7**:**227-232 **(level 3)**
157. Gonzalez R, Rehnke RD, Ramaswamy A, Smith CD, Clarke JM, Ramshaw BJ (2005) Components separation technique and laparoscopic approach: a review of two evolving strategies for ventral hernia repair. Am Surg 71**:**598-605 **(level 3)**
158. Muller-Riemenschneider F, Roll S, Friedrich M, Zieren J, Reinhold T, von der Schulenburg JM, Greiner W, Willich SN (2007) Medical effectiveness and safety of conventional compared to laparoscopic incisional hernia repair: a systematic review. Surg Endosc 21**:**2127-2136 **(level 2A)**
159. Pierce RA, Spitler JA, Frisella MM, Matthews BD, Brunt LM. (2007) Pooled data analysis of laparoscopic vs. open ventral hernia repair: 14 years of patient data accrual. Surg Endosc 21:378-386 **(level 2 A)**
160. Mavros MN, Athanasious S, Alexiou VG, Mitsikostas PK, Peppas G, Falagas ME. (2011) Risk Factors for Mesh-related Infections After Hernia Repair Surgery: A Meta-analysis of Cohort Studies. World J Surg 35:2389-2398 **(level Ia)**
161. Ching SS, Sarela AI, Dexter SPL, Hayden JD, McMahon MJ. (2008) Comparison of early outcomes for laparoscopic ventral hernia repair between nonobese and morbidly obese patient populations. Surg Endosc 22:2244-2250 **(level 3)**
162. Novitsky YW, Cobb WS, Kercher KW, Matthews BD, Sing RJ, Heniford BT. (2006) Laparoscopic Ventral Hernia Repair in Obese Patients. Arch Surg 141:57-61 **(level 4)**
163. [LeBlanc KA](http://www.ncbi.nlm.nih.gov/pubmed?term=LeBlanc%20KA%5BAuthor%5D&cauthor=true&cauthor_uid=18237502), [Elieson MJ](http://www.ncbi.nlm.nih.gov/pubmed?term=Elieson%20MJ%5BAuthor%5D&cauthor=true&cauthor_uid=18237502), [Corder JM 3rd](http://www.ncbi.nlm.nih.gov/pubmed?term=Corder%20JM%203rd%5BAuthor%5D&cauthor=true&cauthor_uid=18237502). (2007) Enterotomy and mortality rates of laparoscopic incisional and ventral hernia repair: a review of the literature. JSLS. 11(4):408-14.**(level 1a)**
164. [Uranues S](http://www.ncbi.nlm.nih.gov/pubmed?term=Uranues%20S%5BAuthor%5D&cauthor=true&cauthor_uid=18954777), [Salehi B](http://www.ncbi.nlm.nih.gov/pubmed?term=Salehi%20B%5BAuthor%5D&cauthor=true&cauthor_uid=18954777), [Bergamaschi R](http://www.ncbi.nlm.nih.gov/pubmed?term=Bergamaschi%20R%5BAuthor%5D&cauthor=true&cauthor_uid=18954777). (2008) Adverse events, quality of life, and recurrence rates after laparoscopic adhesiolysis and recurrent incisional hernia mesh repair in patients with previous failed repairs. J Am Coll Surg. 207(5):663-9. Epub 2008 Aug 9.**(level 4)**
165. Bittner R, Arregui ME, Bisgaard T,Dudai M, Ferzli GS,Fitzgibbons RJ, Fortelny RH, Klinge U, Kockerling F, Kuhry E, Kukleta J, Lomanto D,Misra MC,Montgomery A,Morales-Conde S, Reinpold W, Rosenberg J, Sauerland S,Schug-Paß C, Singh K, Timoney M, Weyhe D, Chowbey P. (2011) Guidelines for laparoscopic (TAPP) and endoscopic (TEP) treatment of inguinal Hernia [International Endohernia Society (IEHS)] Surg. Endosc. 25(9):2773-843**(level 1a)**
166. Ríos A, Rodríguez JM, Munitiz V, Alcaraz P, Pérez Flores D, Parrilla P. (2001) [Antibiotic prophylaxis in incisional hernia repair using a prosthesis.](http://www.ncbi.nlm.nih.gov/pubmed/11759801)Hernia. 5(3):148-52.**(level 2b)**
167. Abramov D, Jeroukhimov I, Yinnon AM, Abramov Y, Avissar E, Jerasy Z, Lernau O. (1996) [Antibiotic prophylaxis in incisional hernia repair using a prosthesis.](http://www.ncbi.nlm.nih.gov/pubmed/11759801) Eur J Surg. 162(12):945-8; discussion 949.**(level 2b)**
168. Edwards C, Angstadt J, Whipple O, Grau R. (2005) [Laparoscopic ventral hernia repair: postoperative antibiotics decrease incidence of seroma-related cellulitis.](http://www.ncbi.nlm.nih.gov/pubmed/16372611) Am Surg. 71(11):931-5; discussion 935-6.**(level 4)**
169. White TJ, Santos MC, Thompson JS. (1998) [Factors affecting wound complications in repair of ventral hernias.](http://www.ncbi.nlm.nih.gov/pubmed/9520825)Am Surg. 64(3):276-80.**(level 4)**
170. Deysine M. (2005) [Postmesh herniorrhaphy wound infections: can they be eliminated?](http://www.ncbi.nlm.nih.gov/pubmed/16463947) Int Surg. 90(3 Suppl):S40-4. Review.**(level4)**
171. Bansal VK, Misra MC, Kumar S, Rao K, Singhal P, Goswami A, Guleria S, Arora MK, Chabra A. (2011) A prospective randomized study comparing suture mesh fixation versus tacker mesh fixation for laparoscopic repair of incisional and ventral hernias.Surg Endosc 25:1431–1438**(level 1b)**
172. LeBlanc KA. (2004) Laparoscopic incisional and ventral hernia repair: Complications—how to avoid and handle. Hernia 8: 323–331**(level 4)**
173. Bellows CF, Berger DH. (2006) Infiltration of Suture Sites with Local Anesthesia for Management of Pain Following Laparoscopic Ventral Hernia Repairs: a Prospective Randomized Trial. JSLS 10:345–350**(level 2b)**
174. [Holzheimer RG](http://www.ncbi.nlm.nih.gov/pubmed?term=%22Holzheimer%20RG%22%5BAuthor%5D)**.** (2004) Laparoscopic procedures as a risk factor of deep venous thrombosis, superficial ascending thrombophlebitis and pulmonary embolism--case report and review of the literature.Eur J Med Res. 29;9(9):417-22.**(level 4)**
175. [Catheline JM](http://www.ncbi.nlm.nih.gov/pubmed?term=%22Catheline%20JM%22%5BAuthor%5D), [Capelluto E](http://www.ncbi.nlm.nih.gov/pubmed?term=%22Capelluto%20E%22%5BAuthor%5D), [Gaillard JL](http://www.ncbi.nlm.nih.gov/pubmed?term=%22Gaillard%20JL%22%5BAuthor%5D), [Turner R](http://www.ncbi.nlm.nih.gov/pubmed?term=%22Turner%20R%22%5BAuthor%5D), [Champault G](http://www.ncbi.nlm.nih.gov/pubmed?term=%22Champault%20G%22%5BAuthor%5D). (2000) Thromboembolism prophylaxis and incidence of thromboembolic complications after laparoscopic surgery.Int J Surg Investig. 2(1):41-7.**(level 4)**
176. Mcadory RS, Cobb WS,, Carbonell AM. (2009) Progressive preoperative pneumoperitoneum for hernias with loss of domain: Am Surg 2009 75(6):508-9 **(level 4)**
177. LeBlanc K. (2003) Herniorrhaphy with the use of transfascial sutures. In Laparoscopic Hernia Surgery. Ed. K. LeBlanc , Arnold Publisher, London, pp 115-124.
178. Olmi S., Magnone S., Erba L., Bertolini A., Croce E. (2005) Results of Laparoscopic Versus Open Abdominal and Incisional Hernia Repair: JSLS 9(2): 189-195 **(level 4)**
179. Köckerling F, Schneider C, Rexmond MA, Scheidbach H, Konradt J, Bärlehner E, Bruch HP, Kuthe A, Troidl H, Hohenberger W. (1998) Early results of a prospective multicenter study on 500 consecutive cases of laparoscopic colorectal surgery. Surg Endosc 12:37-41 **(level 4)**
180. Heniford BT, Park A, Ramshaw BJ, Voeller G. (2000) Laparoscopic Ventral and Incisional Hernia Repair in 407 Patient: J Am Coll Surg 190(9): 645-650. **(level 4)**
181. Moreno-Egea A, Carillo-Alcaraz A. (2012) Management of non-midline incisional hernia by the laparoscopic approach: results of a long-term follow-up prospective study: Surg Endosc 26:1069-1078 **(level 4)**
182. Berger D. (2009) Laparoskopische Hernienoperationen. In Hernienchirurgie. Edt. Obermaier A, F. Pfeffer, U.T. Hopt. Urban & Fischer München, pp 152-156.
183. Vettoretto N, Carrara A, Corradi A, De Vivo G, Lazzaro L, Ricciardelli L, Agresta F, Amodio C (2012) Laparoscopic Adhesiolysis: Consensus Conference: Colorectal Dis. 14(5):e208-15 **(level 4)**
184. Nardi MJ, Millo P, Brachet Contul R, Fabozzi M, Persico F, Roveroni M, Laie Murix E, Bocchia P, Lorusso R, Gatti A, Grivon M, Allieta R. (2012) Laparoscopic incisional and ventral hernia repair (LIVHR) with Parietex Composite mesh: Minimally Invasive Therapy 21:173-180 **(level 4)**
185. Tsai HW, Chen YJ, Ho CM, Hseu SS, Chao KC, Tsai SK, Wang PH. (2011) Maneuvers to decrease laparoscopy-induced shoulder and upper abdominal pain: a randomized controlles study: Arch Surg 146(12):1360-6 **(level 4)**
186. Zhu Q, Mao Z, Yu B, Jin J, Zheng M, Li J. (2009) Effects of persistent CO_2_-insufflation during different laparoscopic inguinal hernioplasty: a prospective, randomized, controlled study: J Laparoendosc Adv Surg Tech A 19(5):611-4 **(level 2B)**
187. Ghaderi I, Vaillancourt M, Sroka G, Kaneva PA, Vassiliou MC, Choy I, Okrainec A, Seagull FJ, Sutton E, George I, Park A, Brintzenhoff R, Stefanidis D, Fried GM, Feldmann LS. (2011) Evaluation of surgical performance during laparoscopic incisional hernia repair: A multicenter study: Surg Endosc 25:2555-2563 **(level 3)**
188. Vilos GA, Ternamian A, Dempster J, Laberge PY. (2007) Laparoscopic entry: a review of techniques, technologies, and complications. J Obstet Gynaecol Can. 29(5):433-65. **(level 2)**
189. Ferzli GS, Fingerhut A. (2004) Trocar placement for laparoscopic abdominal procedures: a simple standardized method. J Am Coll Surg. 198(1):163-73. **(level 5)**
190. Carbajo MA, Martp del Olmo JC, Blanco JI, Toledano M, de la Cuesta C, Ferreras C, Vaquero C. (2003) Laparoscopic approach to incisional hernia. Surg Endosc. 17(1):118-22. Epub 2002 Oct 29. **(level 4)**
191. Landau O, Raziel A, Matz A, Kyzer S, Haruzi I. (2001) Laparoscopic repair of poststernotomy subxiphoid epigastric hernia. Surg Endosc. 15(11):1313-4. Epub 2001 Aug 16. **(level 4)**
192. Carbonell AM, Kercher KW, Matthews BD, Sing RF, Cobb WS, Heniford BT. (2005) The laparoscopic repair of suprapubic ventral hernias. Surg Endosc. 19(2):174-7. Epub 2004 Dec 9. **(level 4)**
193. Weibel MA, Majno G. (1973) Peritoneal adhesions and their relation to abdominal surgery. A postmortem study. Am J Surg 126(3):345-53. **(level 5)**
194. Brummer TH, Jalkanen J, Fraser J, Heikkinen AM, Kauko M, Makinen J (2011) FINHYST, a prospective study of 5279 hysterectomies: complications and their risk factors. Human reproduction (Oxford, England) 26(7):1741-51. **(level 2c)**
195. Diamond MP. (1991) Postoperative adhesion development after operative laparoscopy: evaluation at early second-look procedures. Operative Laparoscopy Study Group. Fertility and sterility 55(4):700-4. **(level 4)**
196. Sikirica V, Bapat B, Candrilli SD, Davis KL, Wilson M, Johns A. (2011) The inpatient burden of abdominal and gynecological adhesiolysis in the US. BMC surgery 11:13. **(level 2c)**
197. Menzies D, Ellis H. (1990) Intestinal obstruction from adhesions--how big is the problem? Annals of the Royal College of Surgeons of England 72(1):60-3. **(level 2c)**
198. Hershlag A, Diamond MP, DeCherney AH. (1991) Adhesiolysis. Clinical obstetrics and gynecology 34(2):395-402. **(level 5)**
199. Stovall TG, Elder RF, Ling FW. (1989) Predictors of pelvic adhesions. *The* Journal of reproductive medicine 34(5):345-8. **(level 5)**
200. Butt MU, Velmahos GC, Zacharias N, Alam HB, de Moya M, King DR. (2009) Adhesional small bowel obstruction in the absence of previous operations: management and outcomes. World journal of surgery 33(11):2368-71. **(level 2c)**
201. Parker MC, Ellis H, Moran BJ, Thompson JN, Wilson MS, Menzies D (2001) Postoperative adhesions: ten-year follow-up of 12,584 patients undergoing lower abdominal surgery. Diseases of the colon and rectum 44(6):822-29; discussion 29-30. **(level 2c)**
202. Swank DJ, van Erp WF, Repelaer van Driel OJ, Hop WC, Bonjer HJ, Jeekel J. (2002) Complications and feasibility of laparoscopic adhesiolysis in patients with chronic abdominal pain. A retrospective study. Surgical Endoscopy 16(10):1468-73. **(level 4)**
203. Diamond MP, Daniell JF, Feste J, Surrey MW, McLaughlin DS, Friedman S. (1987) Adhesion reformation and de novo adhesion formation after reproductive pelvic surgery. Fertility and sterility 47(5):864-6. **(level 4)**
204. Ward BC, Panitch A. (2011) Abdominal adhesions: current and novel therapies. The Journal of surgical research 165(1):91-111. **(level 5)**
205. Gomez-Gil V, Garcia-Honduvilla N, Pascual G, Rodriguez M, Bujan J, Bellon JM. (2010) Peritoneal adhesion formation and reformation tracked by sequential laparoscopy: optimizing the time point for adhesiolysis. Surgery 147(3):378-91. **(level 5)**
206. Van Der Krabben AA, Dijkstra FR, Nieuwenhuijzen M, Reijnen MM, Schaapveld M, Van Goor H. (2000) Morbidity and mortality of inadvertent enterotomy during adhesiotomy. The British Journal of Surgery 87(4):467-71. **(level 3)**
207. Swank DJ, Swank-Bordewijk SC, Hop WC, van Erp WF, Janssen IM, Bonjer HJ. (2003) Laparoscopic adhesiolysis in patients with chronic abdominal pain: a blinded randomised controlled multi-centre trial. Lancet 361(9365):1247-51. **(level 1b)**
208. Tittel A, Treutner KH, Titkova S, Ottinger A, Schumpelick V. (2001) Comparison of adhesion reformation after laparoscopic and conventional adhesiolysis in an animal model. Langenbeck's archives of surgery 386(2):141-5. **(level 5)**
209. Swank DJ, Bonjer HJ, Jeekel J. (2002) Safe laparoscopic adhesiolysis with optical access trocar and ultrasonic dissection. A prospective study. Surgical Endoscopy 16(12):1796-801. **(level 4)**
210. Janssen IM, Swank DJ, Boonstra O, Knipscheer BC, Klinkenbijl JH, van Goor H. (2003) Randomized clinical trial of ultrasonic versus electrocautery dissection of the gallbladder in laparoscopic cholecystectomy. The British Journal of Surgery 90(7):799-803. **(level 1b)**
211. Kinoshita T, Kanehira E, Omura K, Kawakami K, Watanabe Y. (1999) Experimental study on heat production by a 23.5-kHz ultrasonically activated device for endoscopic surgery. Surgical Endoscopy 13(6):621-5. **(level 5)**
212. Harold KL, Pollinger H, Matthews BD, Kercher KW, Sing RF, Heniford BT. (2003) Comparison of ultrasonic energy, bipolar thermal energy, and vascular clips for the hemostasis of small-, medium-, and large-sized arteries. Surgical Endoscopy 17(8):1228-30. **(level 5)**
213. Luijendijk RW, Hop WCJ, van den Tol MP, de Lange DCD, Braaksma MMJ, IJzermans JNM. (2000) A Comparison of Suture Repair with Mesh Repair for Incisional Hernia. New England Journal of Medicine. 343(6):392–8. **(level 1B)**
214. Birch DW. (2007) Characterizing laparoscopic incisional hernia repair. Can J Surg. 50:195–201 **(level4)**
215. Tsimoyiannis EC, Tsimogiannis KE, Pappas-Gogos G, Nikas K, Karfis E, Sioziou H. (2008) Seroma and recurrence in laparoscopic ventral hernioplasty. JSLS. 12(1):51-7.**(level 3)**
216. LeBlanc KA, Allain BW. (2010) Prevention and Management of Laparoendoscopic Surgical Complications, Third Edition. In: Kavic MS,Nezhat C, Winfield H, editors. Laparoscopic Repair of Ventral Wall Abdominal Hernia. World wide web: Society of Laparoendoscopic Surgeons (SLS).
217. Chowbey PK, Sharma A, Mehrotra M, Khullar R, Soni V, Baijal M. (2006) Laparoscopic repair of ventral / incisional hernias. J Min Access Surg 2:192-8. **(level 5)**
218. LeBlanc KA, Booth WV (1993) Laparoscopic repair of incisional abdominal hernias using expanded polytetrafluoroethylene: preliminary findings. Surg Laparosc Endosc 3:39–41 (**level 5)**
219. Agarwal B, Agarwal S, Gupta M, Mishra A, Mahajan K (2008) Laparoscopic ventral hernia meshplasty with ‘Double breasted’ fascial closure of hernial defect. A new technique. J Laparoendosc Adv Surg Tech 18:222–229( **level 5)**
220. Agarwal BB, Agarwal S, Mahajan KC. (2009)Laparoscopic ventral hernia repair: innovative anatomical closure, mesh insertion without 10-mm transmyofascial port, and atraumatic mesh fixation: a preliminary experience of a new technique. Surg Endosc 23:900–905 DOI 10.1007/s00464-008-0159-7 ( **level 3B)**
221. Banerjee A, Narula VK, Mikami D. (2012) Laparoscopic ventral hernia repair – Does primary repair in addition to placement of mesh decrease recurrence? Surg Endosc 26:1264–1268 DOI 10.1007/s00464-011-2024-3 ( **level 2C -3)**
222. Palanivelu C, Jani KV, Senthilnathan P, Parthasarathi R, Madhankumar MV, Malladi VK (2007) Laparoscopic sutured closure with mesh reinforcement of incisional hernias. Hernia 11:223–228 (**level 4)**
223. Palanivelu C, Rangarajan M, Rajapandian S, Amar V, Parthasarathi R. (2009) Laparoscopic repair of adult diaphragmatic hernias and eventration with primary sutured closure and prosthetic reinforcement: A retrospective study. Surg Endosc 23:978–985 DOI 10.1007/s00464-008-0294-1 ( **level 4)**
224. Chelala E, Gaede F, Douillez V, Dessily M, Alle JL. (2003) The suturing concept for laparoscopic mesh fixation in ventral and incisional hernias: preliminary results. Hernia 7:191-196. . **(level 5)**
225. Chelala E, Thoma M, Tatete B, Lemye AC, Dessily M, Alle JL (2007) The suturing concept for laparoscopic mesh fixation in ventral and incisional hernia repair: mid-term analysis of 400 cases. Surg Endosc 21(3):391–395 ( **level 3)**
226. Losanoff JE, Basson MD, Laker S, Weiner M, Webber JD, Gruber SA (2008) Sutured laparoscopic mesh fixation. Surg Endosc Surg Endosc. 22(3):804-805. Jun 26 [Epub ahead of print]( **level 5)**
227. Chelala E, Debardemaeker Y, Elias B, Charara F, Dessily M, Allé JL.( 2010) Eighty-five redo surgeries after 733 laparoscopic treatments for ventral and incisional hernia: adhesion and recurrence analysis. Hernia 14(2):123-9. Epub 2010 Feb 14. ( **level 3)**
228. Misra MC, Bansal VK, Kulkarni MP, Pawar DK. (2006) Comparison of laparoscopic and open repair of incisional and primary ventral hernia: results of a prospective randomized study. Surg Endosc. 20(12):1839-45.( **level 1B)**
229. Carter JE. (1994) A new technique of fascial closure for laparoscopic incisions. J Laparoendoscopic Surg. 4: 143–148. **(level 5)**
230. Parker M, Goldberg RF, Dinkins MM, Asbun HJ, Daniel Smith C, Preissler S, Bowers SP. (2011) Pilot study on objective measurement of abdominal wall strength in patients with ventral incisional hernia. Surg Endosc. 25:3503–3508 ( **level 2B)**
231. Orenstein SB, Dumeer JL, Monteagudo J, Poi MJ, Novitsky YW. (2011) Outcomes of laparoscopic ventral hernia repair with routine defect closure using ‘‘shoelacing’’ technique Surg Endosc 25:1452–57. (**level 4)**
232. Hickey F, Finch JG, Khanna A. (2011) A systematic review on the outcomes of correction of diastasis of the recti. Hernia 15:607 – 614 **(** **level 2A)**
233. Alder AC, Alder SC, Livingston EH, Bellows CF. (2007) Current Opinions About Laparoscopic Incisional Hernia Repair - A Survey of Practicing Surgeons. Am J Surg 194(5): 659–662. ( **level 4)**
234. Sharma D, Jindal V, Pathania OP, Thomas S. (2010) Novel technique for closure of defect in laparoscopic ventral hernia repair J Minim Access Surg. 6(3): 86–88. ( **level 4)**
235. Cox TC, Pearl JP, Ritter EM. (2010) Rives-Stoppa incisional hernia repair combined with laparoscopic separation of abdominal wall components: a novel approach to complex abdominal wall closure. Hernia 14(6):561-7. Epub 2010 Jul 27. (**level 4)**
236. Van Geffen HJAA, Simmermacher RKJ. (2005) Incisional Hernia Repair: Abdominoplasty, Tissue Expansion, and Methods of Augmentation. World J. Surg. 29,1080–1085 (**level 4)**
237. Barnes GS, Papasavas PK, O'Mara MS, Urbandt J, Hayetian FD, Gagn~ DJ, Newton ED, Caushaj PF (2004) Modified extraperitoneal endoscopic separation of parts for abdominal compartment syndrome Surg Endosc 18:1636-1639 ( **level 5)**
238. Espinosa-de-Los-Monteros A, de la Torre JI, Ahumada LA, Person DW, Rosenberg LZ, Vásconez LO. (2006) Reconstruction of the abdominal wall for incisional hernia repair. Am J Surg. 191(2):173-7. (**level 4)**
239. Nguyen NT, Lee SL, Mayer KL, Furdui GL, Ho HS (2000) Laparoscopic umbilical herniorrhaphy.
     J Laparoendosc Adv Surg Tech A 10: 151–153 (**level 4)**
240. Eid GE, Thodiyil PA, Collins JC, Bonanomi G, Mattar SG, Hughes SJ, Schauer PR,Wilson M. (2006) Laparoscopic Repair of Umbilical Hernias in Conjunction With Other Laparoscopic Procedures. JSLS. 10(1): 63–65. **(level 4)**
241. Griniatsos J, Yiannakopoulou E, Tsechpenakis A, Tsigris C, Diamantis T. (2009) A hybrid technique for recurrent incisional hernia repair. Surg Laparosc Endosc Percutan Tech. 19(5):e177-80.( **level 4)**
242. Schug-Pass C, Trommer Y, Tamme C, Lippert H, Köckerling F. (2006) Dynamic patchplasty--a tension-free reconstruction of incisional hernias. Langenbecks Arch Surg. 391(4):403-8; discussion 409-10. (**level 4)**
243. Mathes SJ, Steinwald PM, Foster RD, Hoffman WY, Anthony JP (2006) Complex Abdominal Wall Reconstruction: A Comparison of Flap and Mesh Closure Ann Surg 232, No. 4, 586–596 ( **level 4)**
244. Beldi G, Wagner M, Bruegger LE, Kurmann A, Candinas D. (2011) Mesh shrinkage and pain in laparoscopic ventral hernia repair: a randomized clinical trial comparing suture versus tack mesh fixation. Surg.Endosc. 25:749-755 (**level 2B )**
245. LeBlanc KA. (2007) Laparoscopic incisional hernia repair: are transfascial sutures necessary? A review of the literature. Surg Endosc. 21(4):508-13.**(level 3a)**
246. Abir F, Eisenberg D, Bell R. (2005) Laparoscopic ventral hernia repair using a two (5-mm) port technique.JSLS. 9(1):94-6.**(level4)**
247. Ho J, Pigazzi A. (2011) Laparoscopic repair of extraction site ventral hernia after robotic prostatectomy: institutional experience with 42 consecutive cases. Hernia. 15(6):673-6.**(level4)**
248. Lambrecht J. (2011) Overlap-coefficient for the relationship between mesh size and defect size in laparoscopic ventral hernia surgery. Hernia. 15(4):473-4.**(level4)**
249. Rosen MJ. (2009) Polyester-based mesh for ventral hernia repair: is it safe? Am J Surg. 197(3):353-9.**(level4)**
250. Baccari P, Nifosi J, Ghirardelli L, Staudacher C. (2009) Laparoscopic incisional and ventral hernia repair without sutures: a single-center experiencewith 200 cases. J Laparoendosc Adv Surg Tech A. 19(2):175-9.**(level4)**
251. Zacharakis E, Hettige R, Purkayastha S, Aggarwal R, Athanasiou T, Darzi A, Ziprin. P. (2008) Laparoscopic parastomal hernia repair: a description of the technique and initial results. Surg Innov. 15(2):85-9.**(level4)**
252. Shah RH, Sharma A, Khullar R, Soni V, Baijal M, Chowbey PK. (2008) Laparoscopic repair of incarcerated ventral abdominal wall hernias. Hernia. 12(5):457-63. **(level4)**
253. Morales-Conde S, Cadet H, Cano A, Bustos M, Martín J, Morales-Mendez S. (2005) Laparoscopic ventral hernia repair without sutures—double crown technique: our experienceafter 140 cases with a mean follow-up of 40 months. Int Surg. 90(3Suppl):S56-62.**(level4)**
254. Jerabek J, Piskac P, Hnizdil L, Bucek J. (2005) The laparoscopic ventral and incisional hernia repair. Bratisl LekListy. 106(3):147-8.**(level4)**
255. Topart P, Ferrand L, Vandenbroucke F, Lozac'h P. (2005) Laparoscopic ventral hernia repair with the Goretex Dualmesh: long-term results and review of the literature. Hernia. 9(4):348-52. **(level4)**
256. Tagaya N, Mikami H, Aoki H, Kubota K. (2004) Long-term complications of laparoscopic ventral and incisional hernia repair. Surg Laparosc Endosc Percutan Tech. 14(1):5-8.**(level4)**
257. Alkhoury F, Helton S, Ippolito RJ. (2011) Cost and clinical outcomes of laparoscopic ventral hernia repair using intraperitoneal non heavyweight polypropylene mesh. Surg Laparosc Endosc Percutan Tech. 21(2):82-5.**(level4)**
258. Berger D. (2010) Laparoscopic IPOM technique. Chirurg. 81(3):211-5.**(level5)**
259. Bachman S, Ramshaw B. (2008) Prosthetic material in ventral herniarepair: how do I choose? Surg Clin North Am. 88(1):101-12, ix.**(level5)**
260. Cobb WS, Kercher KW, Heniford BT. (2005) Laparoscopic repair of incisional hernias.Surg Clin North Am. 85(1):91-103, ix.**(level5)**
261. Trap R, Schulze S, Kristiansen VB. (2003) Ventral herniotomy. Development of surgical technique and effect on the frequency of recurrence. Ugeskr Laeger. 165(7):672-8.**(level5)**
262. LeBlanc KA. (2001) The critical technical aspects of laparoscopic repair of ventral and incisional hernias. Am Surg. 67(8):809-12.**(level5)**
263. Berger D, Bientzle M, Müller A. (2002) Postoperative complications after laparoscopic incisional hernia repair. Incidence and treatment. Surg Endosc. 16(12):1720-3. **(level 4)**
264. Bingener J, Buck L, Richards M, Michalek J, Schwesinger W, Sirinek K. (2007) Long-term outcomes in laparoscopic vs open ventral hernia repair. Arch Surg. 142(6):562-7. **(level 4)**
265. McKinlay RD, Park A. (2004) Laparoscopic ventral incisional hernia repair: a more effective alternative to conventional repair of recurrent incisional hernia. J Gastrointest Surg. 8(6):670-4. **(level 4)**
266. Yavuz N, Ipek T, As A, Kapan M, Eyuboglu E, Erguney S. (2005) Laparoscopic repair of ventral and incisional hernias: our experience in 150 patients. J LaparoendoscAdv Surg Tech A. 15(6):601-5. **(level 4)**
267. Chelala E, Thoma M, Tatete B, Lemye AC, Dessily M, Alle JL. (2007) The suturing concept for laparoscopic mesh fixation in ventral and incisional hernia repair: Mid-term analysis of 400 cases. Surg Endosc. 21(3):391-5. **(level 4)**
268. Bencini L, Sanchez LJ, Boffi B, Farsi M, Martini F, Rossi M, Bernini M, Moretti R. (2009) Comparison of laparoscopic and open repair for primary ventral hernias. Surg Laparosc Endosc Percutan Tech. 19(4):341-4. **(level 4)**
269. Bageacu S, Blanc P, Breton C, Gonzales M, Porcheron J, Chabert M, Balique JG. (2002) Laparoscopic repair of incisional hernia: a retrospective study of 159 patients. Surg Endosc. 16(2):345-8. **(level 4)**
270. Chowbey PK, Sharma A, Khullar R, Mann V, Baijal M, Vashistha A. (2000) Laparoscopic ventral hernia repair. J Laparoendosc Adv Surg Tech A. 10(2):79-84.**(level 4)**
271. Frantzides CT, Carlson MA, Zografakis JG, Madan AK, Moore RE. (2004) Minimally invasive incisional herniorrhaphy: a review of 208 cases. Surg Endosc. 18(10):1488-91. **(level 4)**
272. Kirshtein B, Lantsberg L, Avinoach E, Bayme M, Mizrahi S. (2002) Laparoscopic repair of large incisional hernias. Surg Endosc. 16(12):1717-9. **(level 4)**
273. Morales-Conde S, Cadet H, Cano A, Bustos M, Martín J, Morales-Mendez S. (2005) Laparoscopic ventral hernia repair without sutures--double crown technique: our experience after 140 cases with a mean follow-up of 40 months. Int Surg. 90(3 Suppl):S56-62. **(level 4)**
274. Moreno-Egea A, Cartagena J, Vicente JP, Carrillo A, Aguayo JL. (2008) Laparoscopic incisional hernia repair as a day surgery procedure: audit of 127 consecutive cases in a university hospital. Surg Laparosc Endosc Percutan Tech. 18(3):267-71. **(level 4)**
275. Olmi S, Erba L, Magnone S, Bertolini A, Croce E. (2006) Prospective clinical study of laparoscopic treatment of incisional and ventral hernia using a composite mesh: indications, complications and results. Hernia. 10(3):243-7. **(level 3)**
276. Wassenaar E, Schoenmaeckers E, Raymakers J, van der Palen J, Rakic S. (2010) Mesh-fixation method and pain and quality of life after laparoscopic ventral or incisional hernia repair: a randomized trial of three fixation techniques. Surg Endosc. 24(6):1296-302. **(level 1B)**
277. Eriksen JR, Bisgaard T, Assaadzadeh S, Jorgensen LN, Rosenberg J. (2011) Randomized clinical trial of fibrin sealant versus titanium tacks for mesh fixation in laparoscopic umbilical hernia repair. Br J Surg. 98(11):1537-45.**(level 1B)**
278. Nguyen SQ, Divino CM, Buch KE, Schnur J, Weber KJ, Katz LB, Reiner MA, Aldoroty RA, Herron DM. (2008) Postoperative pain after laparoscopic ventral hernia repair: a prospective comparison of sutures versus tacks. JSLS. 12(2):113-6. **(level 1B)**
279. Schoenmaeckers EJ, de Haas RJ, Stirler V, Raymakers JT, Rakic S. (2012) Impact of the number of tacks on postoperative pain in laparoscopic repair of ventralhernias: do more tacks cause more pain? Surg Endosc. 26(2):357-60**(level 3)**
280. Ceccarelli G, Patriti A, Batoli A, Bellochi R, Spaziani A, Pisanelli MC, Casciola L. (2008) Laparoscopic incisional hernia mesh repair with the "double-crown"technique: a case-control study. J Laparoendosc Adv Surg Tech A. 18(3):377-82. **(level 4)**
281. Wassenaar EB, Raymakers JT, Rakic S. (2008) Impact of the mesh fixation technique on operation time in laparoscopic repair of ventral hernias. Hernia. 12(1):23-5. **(level 4)**
282. Schoenmaeckers EJ, van der Valk SB, van den Hout HW, Raymakers JF, Rakic S. (2009) Computed tomographic measurements of mesh shrinkage after laparoscopic ventral incisional hernia repair with an expanded polytetrafluoroethylene mesh. SurgEndosc. 23(7):1620-3. **(level 4)**
283. LeBlanc KA. (2003) Tack hernia: a new entity. JSLS. 7(4):383-7. **(level 4)**
284. Muysoms FE, Cathenis KK, Claeys DA. (2007) "Suture hernia": identification of a new type of hernia presenting as a recurrence after laparoscopic ventral herniarepair. Hernia. 11(2):199-201. **(level 4)**
285. Malmstrøm ML, Thorlacius-Ussing O. (2010) Cardiac tamponade as a rare complication in laparoscopic incisional hernia repair. Hernia. 14(4):421-2. **(level4)**
286. Khandelwal RG, Bibyan M, Reddy PK. (2010) Transfascial suture hernia: a rare form of recurrence after laparoscopic ventral hernia repair. J Laparoendosc Adv Surg TechA. 20(9):753-5. **(level 4)**
287. Barzana D, Johnson K, Clancy TV, Hope WW. (2012) Hernia recurrence through a composite mesh secondary to transfascial suture holes. Hernia. 16(2):219-21. 12. [Epub ahead of print] PubMed PMID: 20835907. **(level 4)**
288. Lepere M, Benchetrit S, Bertrand JC, Chalbet JY, Combier JP, Detruit B,Herbault G, Jarsaillon P, Lagoutte J, Levard H, Rignier P. (2008)Laparoscopic resorbable mesh fixation. Assessment of an innovative disposable instrument delivering resorbable fixation devices: I-Clip(TM). Final results of aprospective multicentre clinical trial. Hernia. 12(2):177-83. **(level 4)**
289. Hollinsky C, Kolbe T, Walter I, Joachim A, Sandberg S, Koch T, Rülicke T, Tuchmann A. (2010)Tensile strength and adhesion formation of mesh fixation systems usedin laparoscopic incisional hernia repair. Surg Endosc. 24(6):1318-24. **(level 5)**
290. Byrd JF, Agee N, Swan RZ, Lau KN, Heath JJ, Mckillop IH, Sindram D, Martinie JB, Iannitti DA. (2011)Evaluation of absorbable and permanent mesh fixation devices: adhesion formation and mechanical strength. Hernia. 15(5):553-8. **(level 5)**
291. Olmi S, Scaini A, Erba L, Croce E. (2007) Use of fibrin glue (Tissucol) in laparoscopic repair of abdominal wall defects: preliminary experience. SurgEndosc. 21(3):409-13. **(level 3)**
292. Olmi S, Cesana G, Sagutti L, Pagano C, Vittoria G, Croce E. (2010)Laparoscopic incisional hernia repair with fibrin glue in select patients. JSLS. 14(2):240-5. Erratum in: JSLS. 2011 Jul-Sep;15(3):430. **(level 4)**
293. Rieder E, Stoiber M, Scheikl V, Poglitsch M, Dal Borgo A, Prager G, Schima H. (2011)Mesh fixation in laparoscopic incisional hernia repair: glue fixation provides attachment strength similar to absorbable tacks but differs substantially indifferent meshes. J Am Coll Surg. 212(1):80-6. **(level 5)**
294. Clarke T, Katkhouda N, Mason RJ, Cheng BC, Algra J, Olasky J, Sohn HJ, Moazzez A, Balouch M. (2011) Fibrin glue for intraperitoneal laparoscopic mesh fixation: a comparative study in a swine model. Surg Endosc. 25(3):737-48. **(level 5)**
295. Fortelny RH, Petter-Puchner AH, Ferguson J, Gruber-Blum S, Brand J, Mika K, Redl H. (2011)A comparative biomechanical evaluation of hernia mesh fixation by fibrin sealant. J Surg Res. 171(2):576-81. **(level 5)**
296. Eriksen JR, Bech JI, Linnemann D, Rosenberg J. (2008) Laparoscopic intraperitoneal mesh fixation with fibrin sealant (Tisseel) vs. titanium tacks: a randomised controlled experimental study in pigs. Hernia. 12(5):483-91. **(level 4)**
297. Melman L, Jenkins ED, Deeken CR, Brodt MD, Brown SR, Brunt LM, Eagon JC,Frisella M, Matthews BD. (2010) Evaluation of acute fixation strength for mechanical tacking devices and fibrin sealant versus polypropylene suture for laparoscopicventral hernia repair. Surg Innov. 17(4):285-90.**(level 5)**
298. Schug-Pass C, Lippert H, Köckerling F. (2009) Fixation of mesh to the peritoneum usingfibrin glue: investigations with a biomechanical model and an experimental laparoscopic porcine model. Surg Endosc. 23(12):2809-15. **(level 5)**
299. Jenkins ED, Melman L, Frisella MM, Deeken CR, Matthews BD. (2010) Evaluation of acute fixation strength of absorbable and non absorbable barrier coated mesh secured with fibrin sealant. Hernia. 14(5):505-9.**(level 5)**
300. Wolter A, Rudroff C, Sauerland S, Heiss MM. (2009) Laparoscopic incisional hernia repair: evaluation of effectiveness and experiences. Hernia. 13(5):469-74. **(level 3)**
301. LeBlanc KA. (2007)Laparoscopic incisional hernia repair: are transfascial sutures necessary? A review of the literature. Surg Endosc. 21(4):508-13. **(level 1A)**
302. Berger D, Bientzle M, Müller A. (2002) Laparoscopic repair of incisional hernias. Chirurg. 73(9):905-8. **(level 4)**
303. LeBlanc KA. (2005) Incisional hernia repair: laparoscopic techniques. World J Surg. 29(8):1073-9. Review. **(level1B)**
304. Cobb WS, Kercher KW, Matthews BD, Burns JM, Tinkham NH, Sing RF, Heniford BT. (2006) Laparoscopic ventral hernia repair: a single center experience. Hernia. 10(3):236-42. **(level 4)**
305. Chelala E, Gaede F, Douillez V, Dessily M, Alle JL. (2003)The suturing concept for laparoscopic mesh fixation in ventral and incisional hernias: preliminary results. Hernia. 7(4):191-6. **(level 4)**
306. Eriksen JR, Poornoroozy P, Jørgensen LN, Jacobsen B, Friis-Andersen HU, Rosenberg J. (2009) Pain, quality of life and recovery after laparoscopic ventral hernia repair. Hernia. 13(1):13-21. **(level 4)**
307. Jenkins ED, Melman L, Desai S, Brown SR, Frisella MM, Deeken CR, Matthews BD. (2011)Evaluation of intraperitoneal placement of absorbable and non-absorbable barrier-coated mesh secured with fibrin sealant in a New Zealand white rabbit model. SurgEndosc. 25(2):604-12. **(level 5)**
308. Ladurner R, Drosse I, Bürklein D, Plitz W, Barbaryka G, Kirchhoff C, Kirchhoff S, Mutschler W, Schieker M, Mussack T. (2011) Cyanoacrylate glue forintra-abdominal mesh fixation of polypropylene-polyvinylidene fluoride meshes in a rabbit model. J Surg Res. 167(2):e157-62. **(level 5)**
309. Ladurner R, Drosse I, Seitz S, Plitz W, Barbaryka G, Siebeck M, Bürklein D, Kirchhoff C, Buhman S, Mutschler W, Schieker M, Mussack T. (2008) Tissue attachment strength and adhesion formation of intraabdominal fixed meshes with cyanoacrylat glues. Eur J Med Res. 13(5):185-91. **(level 5)**
310. van't Riet M, de Vos van Steenwijk PJ, Kleinrensink GJ, Steyerberg EW, Bonjer HJ. (2002)Tensile strength of mesh fixation methods in laparoscopic incisional herniarepair. Surg Endosc. 16(12):1713-6. **(level 5)**
311. Lerdsirisopon S, Frisella MM, Matthews BD, Deeken CR. (2011) Biomechanical evaluation of potential damage to hernia repair materials due to fixation with helical titanium tacks. Surg Endosc. 25(12):3890-7. **(level 5)**
312. Winslow ER, Diaz S, Desai K, Meininger T, Soper NJ, Klingensmith ME. (2004) Laparoscopic incisional hernia repair in a porcine model: what do transfixion sutures add? Surg Endosc. 18(3):529-35.**(level 5)**
313. Koehler RH, Voeller G. (1999) Recurrences in laparoscopic incisional hernia repairs: a personal series and review of the literature. JSLS. 3(4):293-304. **(level 4)**
314. Rudmik LR, Schieman C, Dixon E, Debru E. (2006) Laparoscopic incisional hernia repair: a review of the literature. Hernia. 10(2):110-9. **(level 1A)**
315. Bedi AP, Bhatti T, Amin A, Zuberi J. (2007) Laparoscopic incisional and ventral hernia repair. J Minim Access Surg. 3(3):83-90. Review**(level 1A)**
316. Brill JB, Turner PL. (2011) Long-term outcomes with transfascial sutures versus tacks in laparoscopic ventral hernia repair: a review. Am Surg. 77(4):458-65. Review. **(level 1A)**
317. Eriksen JR. (2010) Fixation of mesh to the peritoneum using a fibrin glue: investigations with a biomechanical model and an experimental laparoscopic porcine model. Surg Endosc. 24(6):1501-2; **(level 5)**
318. Bendavid R. (1990) Incisional parapubic hernias. Surgery. 108(5):898-90**{level 4)**
319. Matuszewski M, Stanek A, Maruszak H, Krajka K. (1999) Laparoscopic treatment of parapubic post prostatectomy hernia. Eur Urol. 36(5):418-20. **{level 4}**
320. Hirasa T, Pickleman J, Shayani V. (2001) Laparoscopic repair of parapubic hernia.Arch Surg. 136(11):1314-7**. {level 4}**
321. McKay R, Haupt D. (2006) Laparoscopic repair of low abdominal wall hernias by tack fixation to the cooper ligament. Surg Laparosc Endosc Percutan Tech. 16(2):86-90. **{level 4}**
322. Palanivelu C, Rangarajan M, Parthasarathi R, Madankumar MV, Senthilkumar K. (2008) Laparoscopic repair of suprapubic incisional hernias: suturing and intraperitoneal composite mesh onlay. A retrospective study. Hernia. 12(3):251-6. **{level 4}**
323. Ferrari GC, Miranda A, Sansonna F, Magistro C, Di Lernia S, Maggioni D,Franzetti M, Costanzi A, Pugliese R. (2009) Laparoscopic repair of incisional hernias located on the abdominal borders: a retrospective critical review. Surg Laparosc Endosc Percutan Tech. 19(4):348-52. **{level 4}**
324. Sharma A, Dey A, Khullar R, Soni V, Baijal M, Chowbey PK. (2011) Laparoscopic repair of suprapubic hernias: transabdominal partial extraperitoneal (TAPE) technique.Surg Endosc. 25(7):2147-52. Epub 2010 Dec 24**. {level 4}**
325. Carbonell AM, Kercher KW, Sigmon L, Matthews BD, Sing RF, Kneisl JS, Heniford BT. (2005) A novel technique of lumbar hernia repair using bone anchor fixation. Hernia. 9(1):22-5**.(level 5)**
326. Craft RO, Harold KL. (2009)Laparoscopic repair of incisional and other complexabdominal wall hernias. Perm J. 13(3):38-42. **{level 5}**
327. Moreno-Egea A, Campillo-Soto A, la Calle MC, Torralba-Martínez JA, Girela E,Albasini JL. (2006) Incisional pubic hernia: treatment of a case with congenital malformation of the pelvis. Hernia. 10(1):87-9. **{level 4}**
328. Norris JP, Flanigan RC, Pickleman J. (1994)Parapubic hernia following radical retropubic prostatectomy. Urology. 44(6):922-3.
329. Yao S, Li JY. (2010) Treatment for incisional parapubic hernia: an experience of 25 cases. Am Surg. 76(12):1420-2. **{level 4}**
330. Cohen MJ, Starling JR. (1985)Repair of subxiphoid incisional hernias with Marlex mesh after median sternotomy. Arch Surg. 120(11):1270-1 **{level 4}**
331. Mackey RA, Brody FJ, Berber E, Chand B, Henderson JM. (2005)Subxiphoid incisional hernias after median sternotomy. J Am Coll Surg. 201(1):71-6. **{level 3}**
332. Eisenberg D, Popescu WM, Duffy AJ, Bell RL. (2008) Laparoscopic treatment ofsubxiphoid incisional hernias in cardiac transplant patients. JSLS. 12(3):262-6. **{level 4}**
333. Muscarella P, Needleman BJ, Goldstein AH, Steinberg SM (2000) Laparoscopic repair of a subxiphoid incisional hernia followingmedian sternotomy. Surg Rounds 23:605–611 **{level 4}**
334. [Theodoropoulou K](http://www.ncbi.nlm.nih.gov/pubmed?term=Theodoropoulou%20K%5BAuthor%5D&cauthor=true&cauthor_uid=20529535), [Lethaby D](http://www.ncbi.nlm.nih.gov/pubmed?term=Lethaby%20D%5BAuthor%5D&cauthor=true&cauthor_uid=20529535), [Hill J](http://www.ncbi.nlm.nih.gov/pubmed?term=Hill%20J%5BAuthor%5D&cauthor=true&cauthor_uid=20529535), [Gupta S](http://www.ncbi.nlm.nih.gov/pubmed?term=Gupta%20S%5BAuthor%5D&cauthor=true&cauthor_uid=20529535), [Bradpiece H](http://www.ncbi.nlm.nih.gov/pubmed?term=Bradpiece%20H%5BAuthor%5D&cauthor=true&cauthor_uid=20529535) (2010) Laparoscopic hernia repair: a two-port technique.[JSLS](http://www.ncbi.nlm.nih.gov/pubmed?term=20529535) 14:103-5**(level 3b)**
335. [Hussain A](http://www.ncbi.nlm.nih.gov/pubmed?term=Hussain%20A%5BAuthor%5D&cauthor=true&cauthor_uid=18435900), [Mahmood H](http://www.ncbi.nlm.nih.gov/pubmed?term=Mahmood%20H%5BAuthor%5D&cauthor=true&cauthor_uid=18435900), [Shuaib S](http://www.ncbi.nlm.nih.gov/pubmed?term=Shuaib%20S%5BAuthor%5D&cauthor=true&cauthor_uid=18435900), [El-Hasani S](http://www.ncbi.nlm.nih.gov/pubmed?term=El-Hasani%20S%5BAuthor%5D&cauthor=true&cauthor_uid=18435900) (2008) Prevention of trocar site incisional hernia following laparoscopic ventral hernia repair.[JSLS](http://www.ncbi.nlm.nih.gov/pubmed?term=18435900) 12: 206-9 **(level 3b)**
336. [Perry KA](http://www.ncbi.nlm.nih.gov/pubmed?term=Perry%20KA%5BAuthor%5D&cauthor=true&cauthor_uid=18274823), [Millikan KW](http://www.ncbi.nlm.nih.gov/pubmed?term=Millikan%20KW%5BAuthor%5D&cauthor=true&cauthor_uid=18274823), [Huang WW](http://www.ncbi.nlm.nih.gov/pubmed?term=Huang%20WW%5BAuthor%5D&cauthor=true&cauthor_uid=18274823), [Myers JA](http://www.ncbi.nlm.nih.gov/pubmed?term=Myers%20JA%5BAuthor%5D&cauthor=true&cauthor_uid=18274823) (2008) A novel approach to extraction of incarcerated omentum and mesh insertion in laparoscopic ventral hernia repair. [Surg Endosc](http://www.ncbi.nlm.nih.gov/pubmed?term=18274823) 22: 798-801.**(level 5)**
337. [Nimeri AA](http://www.ncbi.nlm.nih.gov/pubmed?term=%22Nimeri%20AA%22%5BAuthor%5D), [Brunt LM](http://www.ncbi.nlm.nih.gov/pubmed?term=%22Brunt%20LM%22%5BAuthor%5D) (2006) Laparoscopic ventral hernia repair: 5-mm port technique and alternative mesh insertion method. [J Am Coll Surg](http://www.ncbi.nlm.nih.gov/pubmed/16571446)202: 708-10.**(level 4)**
338. [Carlson MA](http://www.ncbi.nlm.nih.gov/pubmed?term=Carlson%20MA%5BAuthor%5D&cauthor=true&cauthor_uid=17318691), [Petersen A](http://www.ncbi.nlm.nih.gov/pubmed?term=Petersen%20A%5BAuthor%5D&cauthor=true&cauthor_uid=17318691) (2007) Technique for the insertion of large mesh during minimally invasive incisional herniorrhaphy.[Surg Endosc](http://www.ncbi.nlm.nih.gov/pubmed?term=%2017318691) 21:1243-4.**(level 5)**
339. Miller KS, Carey SD, Rodriguez FJ, Smoot RT.(2003) Complications and their management. In Laparoscopic Hernia Surgery. Edt. K. LeBlanc. Arnold London, pp 161-169.**(level 4)**
340. [Liberman MA](http://www.ncbi.nlm.nih.gov/pubmed?term=Liberman%20MA%5BAuthor%5D&cauthor=true&cauthor_uid=11800347), [Rosenthal RJ](http://www.ncbi.nlm.nih.gov/pubmed?term=Rosenthal%20RJ%5BAuthor%5D&cauthor=true&cauthor_uid=11800347), [Phillips EH](http://www.ncbi.nlm.nih.gov/pubmed?term=Phillips%20EH%5BAuthor%5D&cauthor=true&cauthor_uid=11800347) (2002) Laparoscopic ventral and incisional hernia repair: a simplified method of mesh placement. [J Am Coll Surg](http://www.ncbi.nlm.nih.gov/pubmed?term=11800347) 194: 93-5.**(level 5)**
341. [WalterCJ](http://www.ncbi.nlm.nih.gov/pubmed?term=%22Walter%20CJ%22%5BAuthor%5D), [Beral DL](http://www.ncbi.nlm.nih.gov/pubmed?term=%22Beral%20DL%22%5BAuthor%5D), [Drew P](http://www.ncbi.nlm.nih.gov/pubmed?term=%22Drew%20P%22%5BAuthor%5D). (2007) Optimum mesh and port sizes for laparoscopic incisional hernia repair. [J Laparoendosc Adv Surg Tech A.](http://www.ncbi.nlm.nih.gov/pubmed/17362181)  17(1):58-63.**(level 5)**
